# 40th International Symposium on Intensive Care & Emergency Medicine: Brussels, Belgium. 24-27 March 2020

**DOI:** 10.1186/s13054-020-2772-3

**Published:** 2020-03-24

## P001 Physiological emergence of amyloid-β1-40 with mechanical ventilation-induced cerebral immunochallenge

### S Lahiri^1^, N Sparrow^2^, L Mangiacotti^2^, PS Rajput^2^, M Koronyo^2^

#### ^1^Cedars-Sinai Medical Center, Neurocritical Care, Los Angeles, United States; ^2^Cedars-Sinai Medical Center, Departments of Neurology, Neurosurgery, and Biomedical Sciences, Los Angeles, United States

**Introduction:**

A long-term cognitive impairment that resembles Alzheimer’s disease (AD) is a known complication of acute critical illnesses that affects up to 2 million individuals annually in the US. Mechanical ventilation (MV) is a hallmark critical care intervention that is strongly associated with cognitive decline.

**Methods:**

We subjected double transgenic Alzheimer’s disease (Adtg) (APP/PSEN1) and wild-type (WT) mice to MV for 4 hours and compared to spontaneously breathing (SB) controls. Cerebral soluble/insoluble amyloid-β (Aβ) and neurological and systemic markers of inflammation were quantified. Hippocampal blood-brain barrier permeability was quantified using a novel methodology that enabled assessment of small and large molecule permeability across the blood-brain barrier. Immunohistochemistry was used to assess the regional relationship between amyloid-β1–40 and acute vascular disruption and neuronal injury.

**Results:**

See image

**Conclusions:**

Short-term MV resulted in increased cerebral soluble Aβ1-40 and increased cerebral TNF-α and IL-6 concentrations. BBB permeability and neuronal injury were decreased in mechanically ventilated ADtg mice, whereas BBB permeability and neuronal injury were increased in mechanically ventilated WT mice compared to their respective SB controls. There was increased distribution of Aβ1-40 in regions of acute vascular disruption, resulting in lower BBB permeability. Overall, these results support a possible physiological role for Aβ1-40 to decrease BBB permeability and neuronal injury during the acute stress of MV, however it is expected that long-term sustained of this putative protective pathway will contribute to neurodegeneration and cognitive impairment.

**Fig. 1 (abstract P001).**
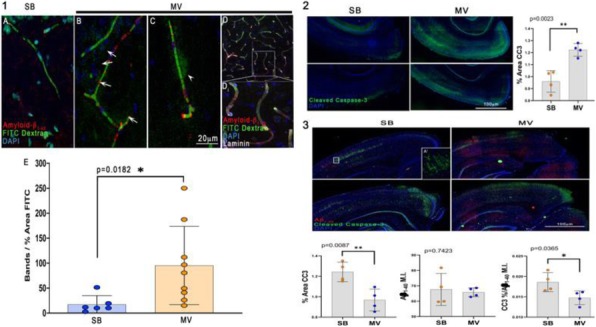
(1) Representative images from ADtg mouse showing markedly increased distribution of Aβ1-40 in a “banding” pattern within a region of acute vascular disruption. Graph showing significant increase in Aβ1-40 bands/% area of FITC in ADtg mice subjected to MV compared to SB ADtg mice. (2) Increased cleaved caspase-3 in the entorhinal cortices of WT mice subjected to MV compared to SB controls. (3) Significantly decreased activation of cleaved caspase-3 in the entorhinal cortices of ADtg mice subjected to MV compared to SB ADtg controls

## P002

**Withdrawn**

## P003 Molecular mechanisms of xenon neuroprotection (experimental data)

### A Kuzovlev^1^, O Grebenchikov^1^, A Shabanov^2^, I Kasatkina^1^, L Nikolaev^3^, I Molchanov^4^

#### ^1^Federal Research and Clinical Center of Intensive Care Medicine and Rehabilitology, Moscow, Russia; ^2^Federal Research and Clinical Center of Intensive Care Medicine and Rehabilitology; N.V. Sklifosofsky Research Institute of Emergency Medicine, Moscow, Russia; ^3^Russian Academy of Postgraduate Education, Moscow, Russia; ^4^Federal Research and Clinical Center of Intensive Care Medicine and Rehabilitology; Russian Academy of Postgraduate Education, Moscow, Russia

**Introduction:**

The aim of the study was to investigate into the molecular mechanisms of neuroprotection with 50 vol% xenon in an in vivo model experiments.

**Methods:**

Eight rats were anesthestized (Combi-Vet machine; induction chloralhydrate 300 mg/kg intraabdominally; then 30 mins of 50 vol% xenon inhalation (95% O2 0.5 l/min, 100% xenon 0.5 l/min; O2 50%, Xe 50%); 8 rats were in the control group (Combi-Vet anesthesia machine; induction chloralhydrate 300 mg/kg intraabdominally; then 30 mins of 95% O2 0.5 l/min). Rats were euthanized and brain homogenates were made. Content of the phosphorylated (inactivated form) of the GSK-3 beta enzyme and key antioxidant enzymes (hemoxygenase, superoxide dismutase, catalase) in rat brain homogenates was assessed by western - immunoblotting. Statistica 6.0, parametric methods were used for data analysis.

**Results:**

The research results showed that xenon inhalation anesthesia resulted in a 2-fold increase of the phosphorylated (inactivated form) of the GSK-3 beta enzyme (р<0.05); increased the content of the key antioxidant enzymes (hemoxygenase (by 50%, р<0.05), superoxide dismutase (by 60%, р<0.05), catalase (by 20%, р>0.05) in rat brain homogenates compared to the controls.

**Conclusions:**

An increase of the phosphorylated GSK-3 beta enzyme and pool of antioxidant enzymes (hemoxigenase, superoxide dismutase, catalase) in the brain under the xenon anesthesia was proved which suggests a new molecular mechanism for the realization of its neuroprotective properties and has a great clinical outlook.

## P004 Procalcitonin and C-reactive protein are not increased in ventriculostomy-related infections in patients with hemorrhagic stroke

### S Wang, E Pietrzko, E Keller, G Brandi

#### University Hospital Zurich, Neurocritical Care Unit, Dept. of Neurosurgery and Institute of Intensive Care Medicine, Zurich, Switzerland

**Introduction:**

Ventriculostomy-related infection (VRI) is a serious complication in patients with hemorrhagic stroke. In such patients, diagnosis of VRIs is complicated by blood contamination of CSF following ventricular hemorrhage. We aimed to evaluate the diagnostic potential of white blood cells count (WBC), C-reactive protein (CRP), and procalcitonin (PCT) to identify VRIs in patients with hemorrhagic stroke during the time of external ventricular drain (EDV) in situ.

**Methods:**

This retrospective study was conducted at the Neurosurgical-ICU, University Hospital of Zurich. A total of 347 patients with hemorrhagic stroke and an external ventricular drain (EVD) were admitted over a 6 years period at the ICU. Of those, 14 patients with VRIs (“VRI”), defined by positive CSF bacterial culture and increased WBC in CSF (>250/ul), and 115 patients without VRIs and with serial CSF sampling (“no-VRI”) were analyzed. Patients with CSF-contamination or suspected VRI (negative CSF cultures but antibiotic treatments) were excluded. WBC, CRP, and PCT were measured daily. CSF was sampled routinely twice a week or by T>38°C. For the analysis, mean peak values of WBC, CRP, PCT during the time of EVD in situ were compared between groups (t test). Data are expressed as mean with CI 95%.

**Results:**

Between groups, WBC and CRP were similar (WBC: 15.13 G/L and 14.55 G/L, p=0.68 and CRP: 115.93 mg/l and 129.44 mg/l, p=0.56 in the group VRI and no-VRI, respectively) (Figure 1, panel A and B). In the group VRI, PCT was low and significantly lower than in the group no-VRI (0.16 ug/l and 2.61ug/l, p=0.03 in the group VRI and no-VRI, respectively) (panel C). WBC in CSF were similar between groups (710.14/ul and 675.16/ul p=0.93 in the group VRI and no-VRI, respectively).

**Conclusions:**

In this study, serum-inflammatory markers were not able to screen patients with VRIs. Their routine measurement should be carefully evaluated.

**Fig. 1 (abstract P004).**
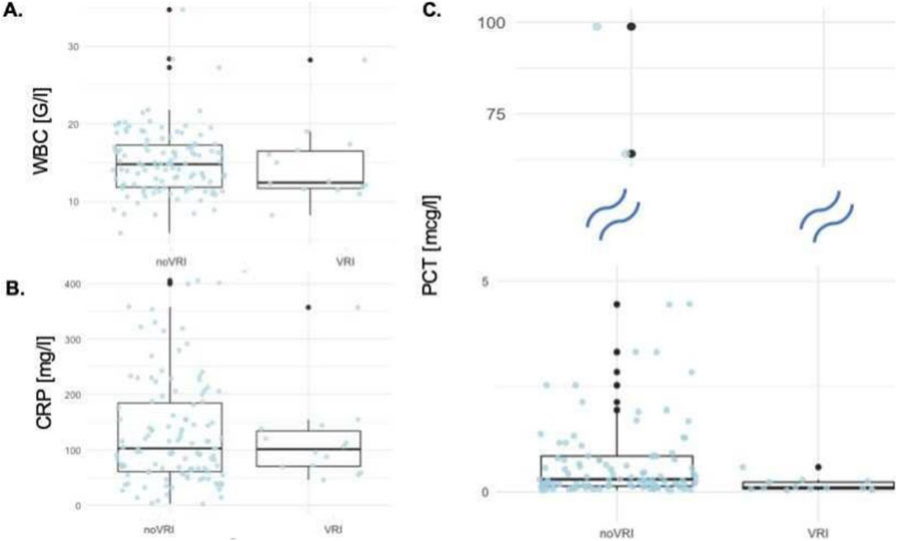
Box-plots of WBC (panel A), CRP (panel B), and PCT (panel C) in the groups

## P005 Central nervous system infections in an intensive care unit: a retrospective study

### A Martinho, E Trigo, M Miranda, P Martins

#### Centro Hospitalar e Universitário de Coimbra - CHUC, Medicina Intensiva, Coimbra, Portugal

**Introduction:**

Central nervous system (CNS) infections constitute a potentially life-threatening neurological emergency. Patients admitted to the intensive care unit (ICU) usually present with a severe disease and organ failure, leading to high mortality and morbidity.

**Methods:**

We have performed a retrospective analysis during a 5-year period of patients admitted to a polyvalent ICU. Clinical, demographic and outcome data were collected to evaluate its clinical impact on the outcome of patients with CNS infections.

**Results:**

We identified 30 patients with the diagnosis of meningitis, meningoencephalitis and ventriculitis, where the median age was 57,6 years (range 24-80). Upon clinical presentation, their most frequent signs were fever (70%), meningeal signs (40%), seizures (30%), and a Glasgow Coma Scale score <8 (66%). All needed ventilation support and 66% needed cardiovascular support. A definitive microbiological diagnosis was achieved on 22 patients and antibiotic therapy was adjusted on 18 of them. Most common microorganisms were *Streptococcus pneumoniae* (n=7), *Listeria* (n=5) and *Pseudomonas aeruginosa* (n=4) (Figure 1). Other gram negative microorganisms were detected and lead to more adverse outcomes. Meningitis was the cause of admission on 26 patients and on a minority (n=4) meningitis was considered to be a secondary diagnosis on patients admitted for other causes (traumatic brain injury, subarachnoid or intraparenchymal hemorrhage, postoperatively of neurosurgical tumor). Patients that eventually died had at least one risk factor (age>65, immunocompromised due to diabetes, corticotherapy, HIV or heart transplantation).

**Conclusions:**

Patients admitted to the ICU were not so aged, but had some comorbidities and risk factors leading to more uncommon microorganisms, increasing the risk of adverse outcomes. This lead to an increase of mortality: 23% in the ICU and an overall of 43%.

**Fig. 1 (abstract P005).**
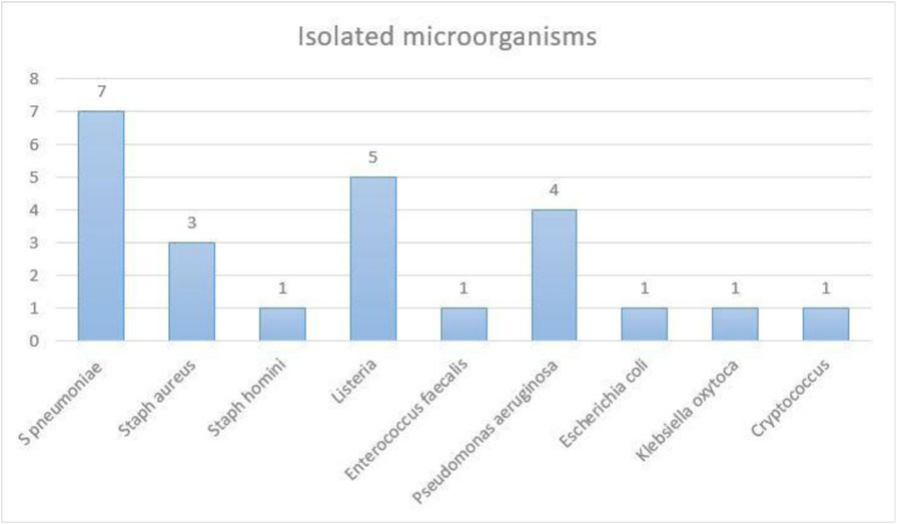
Isolated microorganisms

## P006 Study of selenium levels in unresponsive wakefullness (UWS) patients with systemic inflammatory response syndrome (SIRS)

### E Kondratyeva^1^, S Kondratyev^2^, N Dryagina^2^

#### ^1^Almazov National Medical Reseach Centre, Minimally Conscious Research Group, St Petersburg, Russia; ^2^Almazov National Medical Reseach Centre, Minimally Conscious Science Group, St Petersburg, Russia

**Introduction:**

Patients in UWS  are characterized by a persistent systemic inflammatory response syndrome that can lead to selenium deficiency

**Methods:**

Ànalysis of selenium level was investigated in  54 UWS patients with signs of SIRS:  31patients with TBI and 23 patients with hypoxia. Duration of UWS was 2-4 month.  Selenium blood level was performed once every 10 days for 30 days (three times on the whole). After first blood level measurement, 46 patients (27 TBI and 19 hypoxia) received sodium selenite pentahydrate (Selenase) at a dose of 6 mkg / kg / day for 7 days; In 8 patients (4 TBI and% hypoxia, selenase was not administrated (control group).

**Results:**

In patients who were treated with selenase, maximum concentration of selenium was observed on the 20th day and was on 34.74% higher on average than in the initial study. In the initial study, the average value in patients with  hypoxia was 24.24 ± 10.90 mkg/l, the maximum value was 61.30 mkg/l, between 2-9 days 28.83 ± 15.46 mkg/l (max 70.00 mkg/l), from 10-19 day - 30.22 ± 8.54 mkg/l (max 50.70 mkg/l), from 20-30 days 34.76 ± 16.75 mkg/ l (57.10 mkg/l). At the first measurement the average value in patients with a consequence of TBI was 24.81 ± 8.83 mkg/l, maximum value was 53.00 mkg/l, between 2-9 days 39.26 ± 12.39 mkg/l (max 64.50 mkg/l), from 10-19 day - 37.41 ± 12.88 mkg/l (max 59.50 mcg/l), from 20-30 days 37.35 ± 12.75 mcg/l (max 64.30 mcg/l). Original selenium blood level are 23-190 mkg/l. The average value of the initial values in the control group was 21.4 ± 1.6, mkg/l on day 30 in the control group 17.8 ± 3.8 mkg/l.

**Conclusions:**

Therapy with selenase showed a statistically significant increase of selenium blood concentration (p = 0.00) compared with the control group. Selenase administration did not show a statistically significant value on severity of SIRS process. Average values of selenium levels did not depend on the etiology of brain damage in UWS patients.

**Funding :**

The study was funded by RFBR project number 19-29-01066/2019

## P007 Pharmacokinetics of levetiracetam in critically ill patients with normal and augmented renal clearance

### H Barrasa^1^, I Bilbao-Meseguer^2^, M Solinís^3^, A Rodríguez-Gascón^3^, J Maynar^4^, E Uson^5^, G Balziskueta^4^, F Fonseca^4^, A Martín^4^, A Isla^6^

#### ^1^University Hospital of Alava, Intensive Care, Vitoria, Spain; ^2^Cruces University Hospital, Department of Pharmacy, Cruces University Hospital, Barakaldo, Spain; ^3^Faculty of Pharmacy, PharmaNanoGene Group, Vitoria, Spain; ^4^University Hospital of Alava, intensive Care, Vitoria, Spain; ^5^Can Misses Hospital, intensive Care, Ibiza, Spain; ^6^Faculty of Pharmacy, pharmaNanoGene Group, Vitoria, Spain

**Introduction:**

The objective of this study was to evaluate the pharmacokinetics (PK) of levetiracetam (LEV) in critically ill patients with normal and augmented renal clearance (ARC), and determine if the recommended dosage regimen provides concentrations in the therapeutic range (12-46 mg/L) [1].

**Methods:**

A prospective observational study was conducted in a tertiary hospital. Six blood samples were taken during a dose interval at steady state and LEV was quantified by HPLC. A population PK study was carried out. Statistical analysis was conducted to evaluate the differences in PK between patients with and without ARC. The suitability of drug concentrations was also assessed.

**Results:**

Seventeen patients were included, 13 with normal creatinine clearance (CrCL) (80-129 mL/min) and 4 with CrCl≥130 mL/min (ARC). Ten patients received 500mg q12h, one 1000mg q12h and two 1500mg q12h. The data were best fitted to a two-compartment model. Figure 1 shows LEV concentrations during the dosing interval. Mean clearance (CL) was 4 L/h and mean volume of distribution of central compartment (V) was 44 L. Interindividual variability was 38 and 61% for CL and V, respectively. No differences were identified between both groups (p>0.05) in PK parameters. No correlation was found between LEV CL and CrCL. Trough levels were below the minimum concentration (C_min_) 12 mg/L of the therapeutic range in all patients except 1. Furthermore, between 3-5 h 50% of samples were below the C_min_.

**Conclusions:**

Administered doses were not able to maintain LEV concentrations in the recommended therapeutic range. Other dosage strategies, such the extension of infusion time with higher doses, could be evaluated in order to obtain a more favourable profile. No correlation between LEV CL and CrCL was found.

**Acknowledgements**

We thank the Department of Education of the Basque Government (PIBA 2019-57) and the University of the Basque Country UPV/EHU (PPG17/65, GIU 17/32) for their financial support.

**References:**

1. Reimers A et al. Drug Des Devel Ther. 12: 271–280, 2018.

**Fig. 1 (abstract P007).**
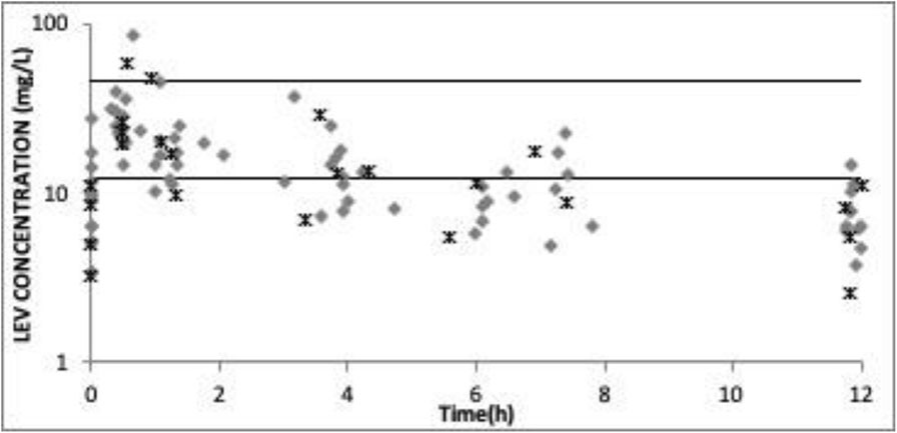
LEV concentrations during the dosing interval

## P008 Feasibility of quantitative assessment of mechanical properties of muscles in chronic critically ill patients

### N Beloborodova, M Petrova, I Buyakova, E Chernevskaya

#### Federal Research and Clinical Center of Intensive Care Medicine and Rehabilitology, Moscow, Russia

**Introduction:**

The mechanical properties of muscles such as tone, elasticity, and stiffness are often affected in chronic critical ill (CCI) patients. A hand-held device known as the MyotonPRO demonstrated acceptable relative and absolute reliability in a ward setting for patients with acute stroke [1]. The technology works on the principle of applying multiple short impulses over the muscle bulk via the testing probe. The aim of our study is to assess the feasibility of objective measurement of muscle tone in CCI patients with neurological dynamics and serum biomarkers.

**Methods:**

The study included 23 CCI patients with neurological disorders (stroke, traumatic brain injury, neurosurgical intervention for brain tumors) with more than a 3-weeks stay in ICU. Dynamic measurements of the muscle properties were taken on the deltoideus, brachioradialis, quadriceps femoris, gastrocnemius using the MyotonPRO. To identify the leading factor in impaired muscle tone also were measured neurological (S100, NSE), inflammatory (IL-6), bacterial load (PCT) biomarkers using Elecsys immunoassay and the serum level of microbial metabolites using GC-MS (Thermo Scientific).

**Results:**

All patients were divided into groups depending on positive and negative clinical dynamics. Significant differences were obtained in parameters characterizing changes in muscle tone of lower limbs - F_gastrocnemius_(tone) -15.5 vs 18.5 Hz, R_quadriceps femoris_(the mechanical stress relaxation time) - 16.5 vs 13.6 ms (p < 0.01, respectively). Some significant correlations between five parameters of muscle tone biomarkers and microbial metabolites were revealed.

**Conclusions:**

The results of a quantitative measurement of muscle tone objectively reflect the dynamics of neurological status, which in the future may be promising technique for the personalized approach CCI in patients.

**References:**

1. Lo WLA et al Biomed Res Int. 2017: 4294028, 2017

## P009 Prognosis of unresponsive wakefulness syndrome outcome based on the determination of certain hormones and natriuretic peptide

### E Kondratyeva, N Dryagina, S Kondratyev

#### Almazov National Medical Reseach Centre, Minimally Conscious Research Group, St Petersburg, Russia

**Introduction:**

Changes in hormonal status in patients with unresponsive wakefulness syndrome (UWS) remains poorly understood.

**Methods:**

275 patients in UWS were examined at the period from 2007 to 2017. 152 patients (115 men) with TBI and 123 patients (63 men) after hypoxia. ACTH, cortisol, TSH, free T3 and T4, STH, prolactin and natriuretic peptide were studied in the period from 2 to 4 months UWS. In men, the level of total testosterone, LH and FSH was additionally studied. The obtained data was compared with the UWS outcome in 6-12 months (CRS-R scale assessment).

**Results:**

None of the studied hormones of the hypothalamic-pituitary-adrenal axis were a reliable criterion for predicting the outcome of UWS. Most often and consistently was revealed a tendency of disrupt the rhythm of cortisol secretion, with higher rates in the evening hours. The average value of STH was higher in men with the consequences of head injury who had recovered consciousness than in those who remained in UWS. Significant decrease in testosterone levels, regardless of age, was found in patients with a consequence of TBI. Mean levels of LH were higher in patients with TBI and hypoxia who remained unconscious than in patients who later restored consciousness. The average level of FSH was higher in patients who had recovered consciousness . The increase of natriuretic peptide level was observed both in patients who remained in chronic UWS and in those who restored consciousness.

**Conclusions:**

No certain endocrine background, characterising this category of patients was found.  Violations of some hormones secretion rhythms, in particular, cortisol can be considered usual for UWS patients, especially in patients with TBI.

**Acknowledgement:** The study was funded by the Russian Foundation For Basic Research (RFBR) project number 19-29-01066/2019

## P010 Craniocerebral hypothermia as a neuromodulation method in chronically critically ill patients

### M Petrova

#### Federal Research and Clinical Center of Intensive Care Medicine and Rehabilitology, Moscow, Russia

**Introduction:**

Therapeutic hypothermia has not been used before our research in chronically critically ill (CCI) patients. Temperature decrease in neuronal cells is a strong signal that triggers endogenic cytoprotection programs using early response genes expression. Our goal is to determine influences of craniocerebral hypothermia (CCH) on level of consciousness in CCI patients.

**Methods:**

We examined 98 patients with different types of brain injuries. 54 males and 44 females, mean age 45.56 ±16.03. Patients were divided into 2 groups: main group - 47 patients (Vegetative State (VS) – 28, minimally conscious state (MCS) – 19), comparison group – 51 patient (VS – 32, MCS –19), groups were equal on main parameters (severity, functional state, comorbidity). Patients from main group received courses of CCH, duration -180 minutes, scalp temperature 5-8 °С, cerebral cortex cooling up to 32-34 ^o^C, session end was without slow reheating period, and session’s amount was set - until signs of consciousness recovery. Cortex temperature check done noninvasively by using detection of brain tissue EMI in SHF- range. Consciousness recovery in VS and MCS patients controlled using CRS-R scale.

**Results:**

CCH sessions significantly increased level of consciousness in VS and MCS patient groups. In VS patients vegetative state increased until minimally conscious state and MCS +, and in MCS group until lucid consciousness (p <0.05) (Figure 1).

**Conclusions:**

Craniocerebral Hypothermia is used in chronically critically ill patients for the first time. Our research results demonstrated effectiveness of CCH as an additive treatment tool in such patients. This let us optimistically determine the perspective of inclusion of CCH method in chronically critically ill patient’s rehabilitation to increase level of consciousness.

**Fig. 1 (abstract P010).**
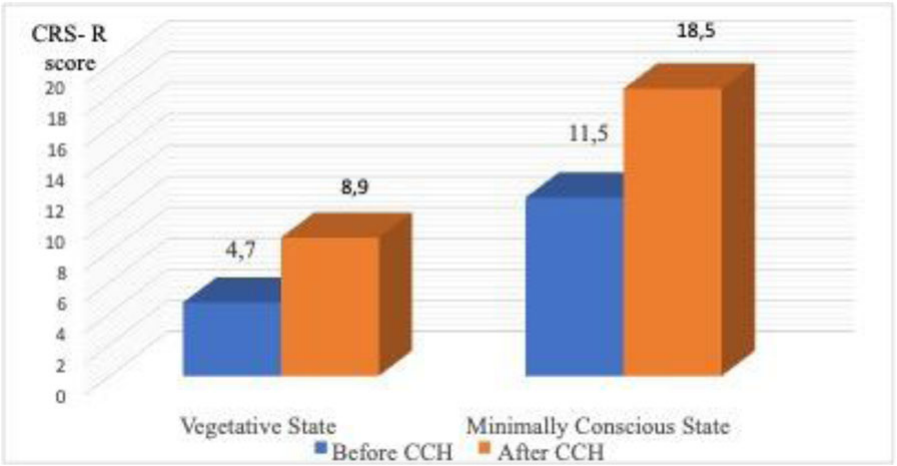
Consciousness change after CCH sessions using CRS-R scale

## P011 Transcranial Doppler assessment of cerebral perfusion in pediatric severe sepsis and septic shock

### A Mostafa^1^, A Elbeleidy^2^, S Awad^2^, R Hamdi^2^, H Elgebaly^2^

#### ^1^Children Cancer Hospital, Pediatric ICU, Giza, Egypt; ^2^Cairo University Hospital, Cairo, Egypt

**Introduction:**

Sepsis associated encephalopathy (SAE) may manifest in up to 70% of adult septic patients. ^**[1]**^  Transcranial Doppler (TCD) may provide important information about cerebral hemodynamics in various disorders. ^[2]^ Two indices reflecting cerebro-vascular resistance (CVR) can be calculated; the pulsatility index (PI), and the resistive index (RI). ^[3]^ In this study, we aimed to assess the cerebrovascular resistance in septic shock paediatric cases with encephalopathy admitted to the PICU and compare them to a group of critically ill conscious non septic patients.

**Methods:**

This case-control study included 45 critically ill children with sepsis associated encephalopathy and 30 critically ill non-septic and non-encephalopathic children admitted to a tertiary care paediatric hospital. PRISM III score and neurological assessment, including level of consciousness assessed using the Full Outline of Unresponsiveness (FOUR) score were determined. PI and RI of the middle meningeal artery were done on both sides using TCD to all included patients.

**Results:**

The study showed that mean PI and RI values of the septic critical cases were significantly higher than those of the control group, CVR increases with the more deepening of coma (the decline in FOUR score). Cerebral CO2 reactivity in septic patients were found disturbed. There were positive correlations between both PI and RI and PaCO2 with high significance.

**Conclusions:**

Cerebrovascular resistance is increased in cases of sepsis - associated encephalopathy. Carbon Dioxide vasoreactivity is impaired in cases of septic shock.

**References:**

1. Pytel P et al. Curr Opin Neurol 22: 283-287, 2009.

2. Purkayastha S et al. Semin Neurol 32:411-20, 2013

3. Iyer VN et al. Mayo Clin Proc 84:694-701, 2009.

## P012 Pupillometry in the follow up of patients undergoing EVT. Could it replace repetitive CT-control

### J Koehn, A Giede-Jeppe, M Hagen, JB Kuramatsu, S Schwab, HB Huttner

#### University of Erlangen-Nuremberg, Department of Neurology, Erlangen, Germany

**Introduction:**

Despite the clinical benefit of endovascular treatment (EVT) for large vessel occlusion (LVO) in ischemic stroke, space-occupying brain edema (BE) represents a common complication during the course of disease. Routinely, CT imaging is used for monitoring of these patients, notably in the critical care setting, yet novel and easy bed-side techniques with the potential to reliably predict BE without repetitive imaging would be valuable for a time and cost effective patient care. We assessed the significance of automated pupillometry for the identification of BE patients after LVO-EVT.

**Methods:**

We enrolled 40 patients admitted to our neurocritical-care unit who received EVT after anterior circulation large vessel occlusion. We monitored parameters of pupillary reactivity [light-reflex latency (Lat; s), constriction and re-dilation velocities (CV, DV; mm/s), and percentage change of apertures (per-change; %)] using a portable pupilometer (NeurOptics®) up to every 60 minutes during the first 72 hours of ICU stay. BE was defined as midline-shift ≥5mm on follow-up imaging within 3-5 days after EVT. We assessed differences in pupillary reactivity between patients with and without BE (U-test) and evaluated prognostic performance of pupillometry for development of BE (ROC analysis).

**Results:**

In 32 patients (19 women, 74.3±12.0 years) without BE, 1,224 assessments were compared to 207 assessments in 6 patients (3 women, 71.7±11.8 years) with BE. On day 1, day 2, and day 3 after EVT, patients with BE had significantly lower CVs and DVs, and smaller per-changes than patients without BE, whereas Lat did not differ between both groups. ROC-analyses revealed a significant negative association of CV, DV, and per-change with development of BE.

**Conclusions:**

Automated pupillometry seems to identify patients at risk for BE after EVT. A prospective study should validate whether automated pupillometry harbors the potential to reduce unnecessary follow-up CT imaging.

## P013 Automated infrared pupillometry: possible hypotheses of extended application in intensive care unit

### F Marturano, F Socci, F Marchi, F Disalvo, G Branchetti, M Bonizzoli, M Ciapetti, A Peris

#### Careggi Teaching Hospital, Intensive Care Unit and ECMO Referral Center, Florence, Italy

**Introduction:**

The aim of this preliminary analysis is to detect differences between the qualitative and quantitative evaluation of the pupillary function carried out by doctors and nurses of an intensive care unit (ICU) of a tertiary level hospital. Secondary purpose is to investigate new indications for the use of pupillometry in a population admitted in ICU

**Methods:**

The study has been conducted (currently in progress) at the intensive care unit and ECMO Referral Center at Careggi Teaching Hospital (Florence; Italy). The enrolled patients are adult subjects (> 18 years) with alteration of consciousness defined by a Glasgow Coma Scale (GCS) < 9, following a primary brain injury and/or the use of sedative drugs. The studied parameters, obtained with NeuroLight pupillometer ® (ID-Med, Marseille, France) are analyzed, integrated and matched with clinical evaluations, biochemical laboratory test and neurophysiological investigations according to internal protocols.

**Results:**

Visual/qualitative evaluation of the pupil function shows a lower reliability if compared to automated pupillometry. The estimated error in the proper determination of photomotor reflex is 34.9% (p< 0.01). No significant difference is reported between quantitative and qualitative pupillometry in the detection of anisocoria.

**Conclusions:**

Our preliminary results are compatible with previously reported data [1-3], even if there was no difference in anisocoria determination. Interestingly, a longer latency period among patients treated with opioids has been observed. Other results are still in progress.

**References:**

1. Morelli P et al. Minerva Anestesiol 85:995-1002, 2019

2. Oddo M et al. Intensive Care Med 44:2102-2111, 2018

3. Couret D et al. Crit Care 13;20:99, 2016

## P014 Use of color-coded duplex sonography in neuromonitoring of critical care patients. Experience at Pirovano Hospital in Buenos Aires

### DN Gauna, L Macchiavello

#### Hospital Pirovano, Critical Care, City of Buenos Aires, Argentina

**Introduction:**

Due to the dynamic of critical care disease, a rapid bedside, non-invasive and highly sensitive and specific method is required for diagnosis. In this study we set out our experience with trancranial color-coded duplex ultrasound (DXT) [1]. The DXT study identifies cerebral arteries as well as hemorrhagic phenomenon, hydrocephalus, mass-occupying lesions and midline shift. This is the main difference between DXT and conventional Transcranial Doppler (DTC) which is a blind study and do not provide any image.

**Methods:**

Descriptive, cross-sectional and observational study from December 2018 to June 2019. 21 patients were included. Inclusion criteria: Neurocritical patients. Exclusion criteria: No acoustic window, presence of ultrasound artifacts. Data collection was performed. It was used a low-frequency transducer from 1.5-3.5 MHz with trancranial duplex preset (Figure). The patterns were defined as normal, vasospasm, high resistance, hypermedia and cerebral circulatory arrest, depending on the cerebral flow velocity, Lindegaard Ratio (LR) and Pulsatility Index (IP).

**Results:**

12 men (57.1%) and 9 women (42.9%). Average age 55.6(20-79). Patients diseases: Subarachnoid hemorrhage 6, traumatic brain injury 5, AV malformation 4, stroke 2, hemorrhagic cerebrovascular accident 2 and mass occupying lesions 2. Normal Pattern: 10 patients (rel. freq 0.47). Vasospasm: 5 patients (rel. freq 0.23). High resistance: 4 patients (rel. freq 0.19). Hyperemia: 1 patient (real. freq 0.04). Cerebral circulatory arrest: 1 patient (rel. freq 0.04)

**Conclusions:**

DXT should be part of the routine of neuromonitoring, it allows real time images especially useful in unstable conditions. Although it will be needed a large amount of patients to be statistical significant, DXT is useful considering a non invasive study, bedside and it allows early identification of different clinic conditions.

**References:**

1. Lau VI. Crit Ultrasound J 9:21, 2017

**Fig. 1 (abstract P014).**
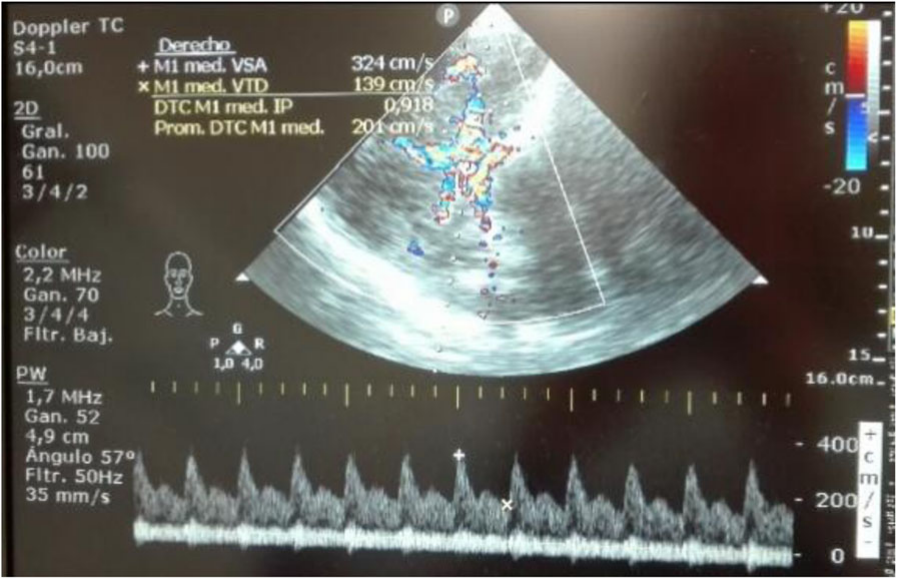
Circle of Willis and Pulsed-wave Doppler mode of middle cerebral artery

## P015

**Withdrawn**

## P016

**Withdrawn**

## P017 Use of adenosine-induced cardioplegia in anesthesia during endovascular treatment of arteriovenous malformation of the brain

### N Lesteva, A. Kondratyev, M Aibazova, D Vasilyev, G Ryibakov, S Lesina

#### Polenov Neurosurgical Institute - Almazov National Medical Research Centre, Anesthesiology and Intensive Care, Saint-Petersburg, Russia

**Introduction:**

Embolization of the draining vein during endovascular treatment of arteriovenous malformation (AVM) may result in venous outflow obstruction and hemorrhage. Anaesthesiologist can use deliberate hypotension to reduce blood flow through AVM which may be somehow helpful to prevent this scenario. Adenosine-induced cardiac arrest may facilitate the embolization too. The goal of our study was to improve the results of endovascular treatment of AVM using adenosine-induced cardiac arrest.

**Methods:**

After obtaining informed consent 13 patients (8 male, 5 female) were selected for adenosine-induced cardiac arrest during endovascular AVM embolization. Main age was 40,8±6 years old. 9 of them were evaluated as III class ASA, 4 as IV. Endovascular treatment in all cases was performed under general anaesthesia. Propofol, fentanyl, rocuronium were used to induce anaesthesia, then all the patients were intubated and ventilated with parameters to keep EtCO2 32-35 mm Hg. Sevoflurane 2,1-2,6 vol% (12 cases) or desflurane 6 vol% (1 case) were used to maintain anaesthesia. Hemodynamic monitoring consisted of ECG, pulsoximetry, non-invasive blood pressure measurement. Onyx or/and Squid were used as embolic agents. CT was performed to every patient just after procedure as well as neurological examination.

**Results:**

Adenosine dosage was 0.875-1.26 mg/kg. Time of consequent cardiac arrest was 10-40 sec. There were 10 cases we administered adenosine for 1 time, in one case we had to administer it twice, in one - 3 times and 4 times in one more case as well. Hemodynamic parameters recovered without any particular treatment in all the patients. Embolization has been performed in all the cases uneventfully. Postoperative CT showed no hemorrhage. Nobody from investigated group had neurological deterioration in postoperative period.

**Conclusions:**

Our study shows that adenosine-indused cardiac arrest is not very difficult to perform method and it can be useful during AVM embolization.

## P018 Incidence of postoperative major adverse cardiac events in patients undergoing carotid endarterectomy: a single-center retrospective study

### A Suphathamwit, C Leewatchararoongjaroen

#### Faculty of Medicine Siriraj Hospital, Mahidol University, Department of Anesthesiology, Bangkok, Thailand

**Introduction:**

Major adverse cardiac events (MACE) is an important causes of morbidity and mortality after carotid endarterctomy (CEA)[1,2,3]. The investigators aim to determine the incidence of MACE in patients undergoing CEA in the tertiary university hospital.

**Methods:**

This retrospective study recruited 175 patients between January 1999 – June 2018. The patients received CEA with coronary artery bypass graft surgery were excluded. Primary outcome was the incidence of MACE at 7 days, 30 days and 1 year postoperatively. MACE was defined as myocardial infarction, congestive heart failure, significant arrythmia and cardiac arrest. Secondary outcome was the incidence of postoperative stroke. The patients’ chart was reviewed and direct contact was performed for patient’s information.

**Results:**

The incidences of MACE were 7.5%, 1.1% and 1.1% at 7 days, 30 days and 1 year, respectively. MACE at 7 days were 1.1% of myocardial infarction, 2.9% arrhythmia, 1.1% congestive heart failure and 2.3% cardiac arrest. There were no significant differences in age, BMI, baseline hemoglobin level, creatinine level and severity of carotid artery stenosis between those with and without MACE. The incidence of stroke at 7 days postoperatively was 4.6%. There was no new stroke occurred at 30 days and 1 year after surgery.

**Conclusions:**

The patients with carotid artery stenosis were at high risk from major cardiac disease. The overall incidence was 7.5% within 7 days after surgery. Significant arrythmia was the most common adverse cardiac event.

**References:**

1. Mutirangura P et al. J Med Assoc Thai 99:785-93, 2016.

2. Go C et al. Ann Vasc Surg 29:1265-71, 2015.

3. Boulanger M et al. Stroke 46:2843-8, 2015

## P019 The antithrombotic effects of different oral anticoagulants in the monitoring of acute stroke

### M Lawrence^1^, V Evans^2^, J Whitley^2^, S Pillai^2^, P Slade^3^, M Krishnan^3^, K Power^4^, PA Evans^2^

#### ^1^Welsh Center of Emergency Medicine, HBRU, Swansea, United Kingdom; ^2^Welsh Center of Emergency Medicine, Swansea, United Kingdom; ^3^Swansea Bay UHB, Stroke Department, Swansea, United Kingdom; ^4^Swansea Bay UHB, Pharmarcy, Swansea, United Kingdom

**Introduction:**

A major risk factor for stroke is atrial fibrillation (AF). To treat AF anticoagulation is needed. There are now several anticoagulants available. However, a lack of head to head data as well as the absence of accurate techniques makes it difficult to compare them and measure determine there efficacy. Stroke is known to produce an abnormal clot microstructure which is a common factor in many thrombotic diseases. This pilot study aims to use a functional biomarker of clot microstructure (d_f_) and clotting time (TGP) to investigate the therapeutic effects of different anticoagulants in stroke and AF.

**Methods:**

We recruited 114 patients (59 AF and 55 stroke & AF). Two samples of blood were taken: before anticoagulation (baseline) and post anticoagulation (6-10weeks). Patients were either given warfarin (31%) or axipaban (69%). d_f_ and TGP were measured and compared before and after anticoagulation.

**Results:**

Warfarin increased T_GP_ (267±56 secs to 332±78 secs (p<0.05)), and decreased d_f_ (1.71±0.05 to 1.65±0.06 (p<0.05)). Apixaban increased TGP (235±66 sec to 410±105 sec (p<0.05)) but did not change df (1.72±0.04 & 1.72±0.05). Interestingly we found that in the apixaban group TGP significantly correlated (p=0.05) with blood drug concentration levels.

**Conclusions:**

In this study we show that d_f_ and TGP can quantify and differentiate between the therapeutic effects of two different oral anticoagulants. Showing that warfarin prolongs clotting and weakens the ability of the blood to form stable clots. Conversely apixaban prolongs clotting time but does not affect the bloods ability to form stable clots. This shows the utility of the d_f_ and TGP biomarkers in comparing two different treatment options, something no other current marker has proven able to do. Where d_f_ and TGP may prove useful tools in a personalized approach to anticoagulation treatment and monitoring in an acute setting.

## P020 HAIR-score optimalisation in subarachnoidal bleeding

### L Capiau^1^, H Vanoverschelde^1^, A Decruyenaere^2^, J Decruyenaere^1^, K Colpaert^1^

#### ^1^Ghent University Hospital, Intensive Care Unit, Ghent, Belgium; ^2^Ghent University Hospital, Internal Medicine, Ghent, Belgium

**Introduction:**

The HAIR-score stratifies the risk for in-hospital mortality after spontaneous subarachnoid hemorrhage (SAH) [1]. Maximal early lactate is associated with increased risk for poor outcome [2]. We investigated whether the combination of both HAIR-score and early abnormal lactate (<24h), gives a better association with mortality after spontaneous SAH.

**Methods:**

In this retrospective cohort study at a tertiary university hospital, data was collected for all patients with spontaneous SAH during a period of eleven years (2007-2017). Multiple binary logistic regression models were fitted for each outcome (ICU mortality and hospital mortality). The first one included the individual components of the HAIR-score, the second model also included an indicator for abnormal (>18 mg/dl) lactate within the first 24h (La-HAIR-score). In addition, two simple binary logistic regression models were fitted as continuous covariates: The HAIR-score (theoretical range 0-8) and the La-HAIR-score, with one extra point to patients with abnormal lactate concentration (theoretical range 0-9). These models were compared with a likelihood ratio test and Nagelkerke’s R² test.

**Results:**

A total of 470 patients were included. In our sample, not all original HAIR components were associated with ICU or hospital mortality. The multiple logistic regression model of the La-HAIR-score had a significantly better fit to the data, compared to the original HAIR-score. The p-value of the likelihood ratio test was 0.01 for ICU mortality and 0.025 for hospital mortality. A score of 8 on the La-HAIR-score had a 100% specificity for both ICU and in-hospital mortality.

**Conclusions:**

Combination of HAIR-score and abnormal lactate within the first 24h, the La-HAIR-score, significantly improved the model fit for ICU and hospital mortality compared to the model with the original HAIR-score.

**References:**

1. Lee VH et al. Neurocrit Care 21:14, 2014

2. Imo P et al. Am J Emerg Med 34:708-712, 2016

## P021 South American experience with urapidil in neurocritical care patients

### M Canitrot^1^, J Ramirez^2^, J Serra^3^, L Brunetto^3^, Y Santos^3^, R Villa^4^, I Previgliano^4^, S Ugarte^3^

#### ^1^INDISA Clinic, Critical Care, Santiago, Chile; ^2^Andres Bello University, NeuroICU, Santiago, Chile; ^3^INDISA Clinic, NeuroICU, Santiago, Chile; ^4^Hospital General J. A. Fernandez, NeuroICU, Buenos Aires, Argentina

**Introduction:**

Urapidil is an antihypertensive, vasodilatory drug with no tachycardia or increased ICP effect.  Recommended for neurocritical patients in European and Asian guidelines. OBJECTIVE: To evaluate the efficacy and tolerance of injectable urapidil for the treatment of hypertensive emergencies in neurocritical patients.

**Methods:**

We carried out a prospective study of 175 cases in 2 South American Neuro ICU (Chile and Argentina), with clinical and biological monitoring. Treatment was initiated if the blood pressure was higher than 180/110mmHg with organ disfunction. Efficacy was defined as a lowering of blood pressure to 150/100 mmHg or below. The drug was administered according to protocol by IV bolus (by PVC) followed by continuous infusion. Sex, age, SAP, MAP, DAP and CR were evaluated at the beginning and end of bolus therapy, as well as  response to the drug at 5, 10, 15 minutes, and maintenance dose.  Statistical analysis: Wilcoxon test for paired samples.

**Results:**

175 patients (average age : 63). Ischemic stroke (86), ICH (59), SAH (16), HELLP / PEE (5), ACS (3), HE (2). Changes in SAP (198 to 130), DAP (116 to 78) and MAP (155 to 94) (p two tails <0.001) (Figure 1). No significant changes in CR (median 76 to 72). 9 deaths, none attributable to administration of urapidil. 124 (71%) responded to 1st dose at 5 min, 50 (28%) to 2nd dose at 10 min and 1 (1%) required a 3rd dose at 15 min. All responded favourably. Urapidil was effective in all cases, with a significant decrease in BP. 99% within 10 minutes. There was no lack of response or complications.

**Conclusions:**

Injectable urapidil seems to be an antihypertensive agent that is easy to use and effective in all the cases of this first South-American experience. Further comparative studies are required.

**Fig. 1 (abstract P021).**
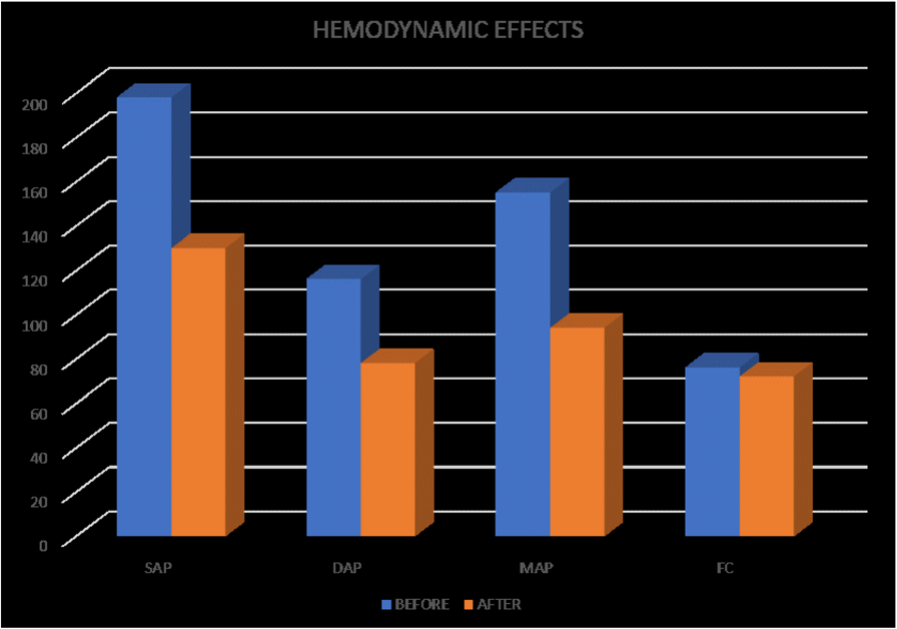
Hemodynamic effects of urapidil

## P022 Poor-grade aneurysm subarachnoid hemorrhage in neuro ICU: how poor is their prognosis?

### M Canitrot^1^, J Ramirez^2^, J Serra^3^, Y Santos^3^, V Munoz^3^, JL Sufan^3^, J Contreras^3^, F Paravic^4^, C Droguett^4^, S Ugarte^2^

#### ^1^INDISA Clinic, Critical Care, Santiago, Chile; ^2^Andres Bello University, NeuroICU, Santiago, Chile; ^3^INDISA Clinic, NeuroICU, Santiago, Chile; ^4^INDISA Clinic, Neurorehabilitation, Santiago, Chile

**Introduction:**

Patients with poor-grade aneurysm subarachnoid hemorrhage (aSAH) World Federation of Neurological Surgeons (WFNS) Grades IV and V, have commonly been considered to have a poor prognosis (70-100% mortality). Though early intervention and aggressive treatment in NeuroICU has improved outcome in the past years, it is controversial because most of the patients left hospital severely disabled. The objective of this study was to investigate the clinical and social outcomes in intracranial aneurysm patients with poor-grade aSAH underwent different intervention therapies.

**Methods:**

A single center observational registry of 25 poor-grade aSAH consecutive patients, defined as WFNS Grades IV and V, treated at tertiary chilean referral center from December 2013 to March 2019 were enrolled in this study. The clinical data including patient characteristics on admission and during treatment course, treatment modality, aneurysm size and location, radiologic features, signs of cerebral herniation (dilated pupils), and functional neurologic outcome were collected. Clinical outcomes were assessed via GOSE and and socio-occupational outcome, both at discharge and at 6 months.

**Results:**

883 admissions, 190 were SAH. 84 patients Fisher III and IV. 25 patients WFNS 4 or 5: 20% mortality (5/25). Every death was declared out of therapeutic reach during admission by neurosurgeons. Mortality per year (3/3 2014, 0/4 2015, 1/3 2016, 0/4 2017, 0/6 2018, 1/5 2019), suggesting reduction of mortality rates while expertise increased. Of the 20 survivors, 3 were released in poor neurological conditions (GOSE ≥4) at the neuro ICU discharge. After 6 months, all patients (20/20) achieved GOSE ≤ 3, with adequate family, social and labor reintegration (Figure 1). 20% mortality is less than previously reported, and survivors had a favorable recovery, confirmed with neuro psychological test.

**Conclusions:**

Poor-grade aSAH patients in our study shows a more positive outcome than previously considered.

**Fig. 1 (abstract P022).**
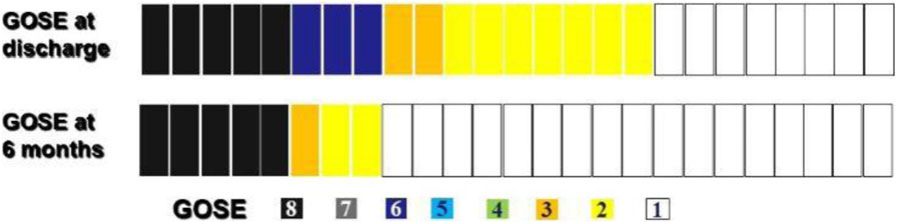
GOSE WFNS 4 - 5 SAH patients

## P023 Success in intravenous thrombolysis with alteplase for acute ischemic stroke: a matter of distance?

### P Papamichalis^1^, AL Skoura^1^, P Katsiafylloudis^1^, E Neou^1^, S Karagiannis^1^, E Dardiotis^2^, M Karvouniaris^1^, D Papadopoulos^1^, T Zafeiridis^1^, A Komnos^1^

#### ^1^General Hospital of Larissa, Department of Intensive Care, Larissa, Greece; ^2^University Hospital of Larissa, Department of Neurology, Larissa, Greece

**Introduction:**

Pre-hospital management of acute ischemic strokes focuses on factors associated with success in intravenous thorombolysis (IVT)with alteplase [1]. Distance of patient’s residence from stroke ready hospitals could be one such factor.

**Methods:**

Retrospective study from our department’s thrombolysis database with 157 patients (mean age 66 years). Demographic data, thrombolysis time (from symptoms onset to drug administration, thombolysis within 3 hours or between 3-4.5 hours), complications during hospital stay (symptomatic intracranial hemorrhage or death or new embolic episode or other major hemorrhage), functional outcome at 3 months (modified Rankin Scale-mRS), mortality at 3 months were retrieved and analyzed in association with distance of patient’s home from our hospital. Distance was calculated with the use of an international web mapping service. Those factors were also compared at patients according to residence in a rural, medium urban or large urban area.

**Results:**

Shorter distance was associated with less time spent for thrombolysis, fewer in-hospital complications and better functional outcome (mRS ≤ 2) at 3 months time (Table 1). Living in a rural area was associated with worse functional and neurologic outcome (p< 0.05, Mann-Whitney U test for both comparisons).

**Conclusions:**

Unlike previous studies [2] our study showed association of thrombolysis time with distance from stoke centre and furthermore better results with less complications when distance is short. Long distance from stroke centres and residence in rural areas probably play a negative role in thrombolysis outcome and should be taken into consideration when national health systems organize pre-hospital and hospital network for strokes.

**References:**

1) Kobayashi A et al. Eur J Neurol 25:425-433, 2018

2) Acharya AB et al. J Stroke Cerebrovasc Dis 20:295-301, 2011

**Table 1 (abstract P023).** Correlation of safety and efficacy markers of thrombolysis and thrombolysis time with distance from stroke centre

	Distance	p Value
Thrombolysis time	(Spearman´s rho: 0.41)	< 0.001^a b^
Thrombolysis time: 3 hours (n= 121) / 3-4.5 (n=36)	16400 (400 - 72600) / 37500 (1400 – 88600)	< 0.001^c b^
Complications: NO (n= 148) / YES (n= 9)	20300 (400 - 88600) / 37000 (2700 - 72600)	0.04^c b^
Death (3 months): NO (n= 136) / YES (n= 21)	20200 (400 - 88600) / 27700 (450 - 72600)	0.24 ^c d^
Functional outcome: mRS≤2 (n= 103) / mRS>2 (n= 54)	18800 (400 - 88600) / 25900 (450 - 77100)	0.03^c b^

Results are expressed as mean (range). a: Spearman Correlation Coefficient. b: Statistically significant. c: Mann-Whitney U test. d: not statistically significant. Complications (during hospital stay): symptomatic intracranial hemorrhage, death or new embolic episode or other major hemorrhage. mRS: modified Rankin Scale

## P024 Influence of pneumonia on delayed cerebral infarction after subarachnoid hemorrhage: a retrospective study

### T Gargadennec^1^, V Vermeersch^1^, J Ognard^2^, M Geslain^1^, P Ariès^1^, J Le Roy^1^, H Floch^1^, A De Tinténiac^3^, X Chapalain^1^, O Huet^1^

#### ^1^Brest University Hospital, Département d´Anesthésie et Réanimation Chirurgicale, Brest, France; ^2^Brest University Hospital, Service de Radiologie et Imagerie Médicale, Brest, France; ^3^Brest University Hospital, Brest, France

**Introduction:**

Prognosis of subarachnoid hemorrhage (SAH) is scarce, indeed almost half patients die or become severely disable after SAH. Outcome is related to the severity of the initial bleeding and delayed cerebral infarction (DCI). Infection and more precisely pneumonia have been associated with poor outcome in SAH. However, the interaction between the two pathologic events remains unclear. Therefore, we hypothesized that DCI may be associated to pneumonia in SAH patients. Thus the aim of our study was to analyze the association between delayed cerebral infarction and pneumonia in patients with SAH.

**Methods:**

In this retrospective, observational, monocentric cohort study, patients included in the analysis were admitted in Neurosurgical Intensive Care Unit or Surgical Intensive Care Unit in the University Hospital of Brest (France) for non-traumatic SAH. Primary outcome was diagnosis of DCI on CT scan or MRI 3 months after SAH. Multivariate analysis was used to identify factors independently associated with DCI.

**Results:**

A total of 224 patients were included in the analysis (female male ratio 141/83, median age 57 [47-65] years). Multivariate analysis was adjusted on sedation, intracranial surgery, Fisher classification of SAH severity, pneumonia occurrence and non-pneumonia infectious event occurrence (Figure 1). Pneumonia occurred in 66 patients (29.5%) and other causes of infections in 45 patients (20.1%). DCI was found in 108 patients (48.2%). Factors independently associated with DCI were pneumonia (OR 3.10 [1.41-7.06]; p=0.006) and non-pneumonia infectious events (OR 2.50 [1.20-5.39]; p=0.016). Interestingly severity of initial bleeding evaluated by Fisher scale was not independently associated with DCI.

**Conclusions:**

DCI is independently associated with the occurrence of pneumonia or other cause of sepsis. Those results may highlight the need for rigorous approach for prevention protocol, early diagnosis and treatment of hospital acquired infectious diseases in SAH patients.

**Fig. 1 (abstract P024).**
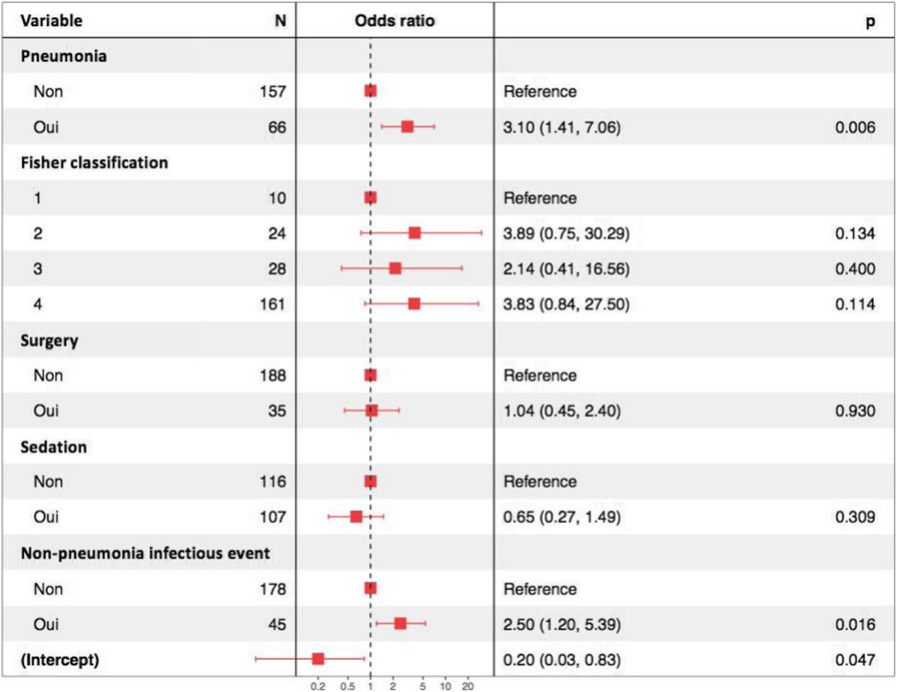
Forest plot representation of results of multivariate analysis for occurrence of delayed cerebral infarction. Results expressed as odds ratio with 95% confidence interval

## P025 Association of prehospital hypocarbia and hypercarbia with outcomes with severe traumatic brain injury patients

### Z Weisner^1^, D Salcido^1^, F Guyette^1^, H Siddiqui^1^, J Salerno^1^, J Elmer^2^

#### ^1^UPMC Presbyterian, Emergency Medicine, Pittsburgh, United States; ^2^UPMC Presbyterian, Emergency Medicine and Critical Care, Pittsburgh, United States

**Introduction:**

Hypocarbia and hypercarbia alter cerebral blood flow and intracranial pressure. Both might cause secondary brain injury after traumatic brain injury (TBI).

**Methods:**

We performed a retrospective observational study from Jan 2012 to Dec 2017 including adult blunt trauma patients with initial Glasgow Coma Scale (GCS) ≤ 14, clinical suspicion for TBI, endotracheal intubation and continuous capnography. We excluded interfacility transfers, penetrating TBI and cases treated for herniation (management includes hyperventilation). All patients were treated by a single air medical transport service in southwest Pennsylvania. Our primary exposure was depth-duration of EtCO2 <35mmHg (hypocarbia). Secondary outcomes were depth-duration of hypercarbia >45mmHg, and maximum depths of hypocarbia and hypercarbia. Our primary outcome was survival to discharge. Covariates were age, sex, initial GCS, shock index and first prehospital serum lactate. We performed unadjusted and adjusted logistic regression testing relationships between EtCO2 and outcome.

**Results:**

Overall, 148 patients were included and 113 (76%) survived. Overall, 104 (70%) were hypocarbic at least once. Median hypocarbia depth was 26 [IQR 18-30] mmHg, median hypocarbia duration was 15 [4 – 28] min, and median depth-duration was 60 [14 – 141] mmHg*min. Hypercarbia occurred in 56 (38%) patients. Median duration of hypercarbia short (0.5 [0 – 5] min) and median depth duration was only 2 [IQR 0 – 18] mmHg*min. Neither hypocarbia nor hypercarbia (maximum, duration or depth-duration) were associated with outcome in unadjusted or adjusted models. Age [OR; 0.96, 95% CI 0.94-0.98], initial GCS scores [OR; 1.12, 95% CI 1.01 to 1.25] and shock index [OR; 0.41 95% CI 0.19 to 0.88] were independent predictors of mortality.

**Conclusions:**

Among intubated blunt TBI patients, prehospital hyper- and hypoventilation do not appear to worsen outcomes.

## P026 Psychiatric drug burden after intensive care unit treated pediatric traumatic brain injury

### E Mikkonen^1^, M Skrifvars^2^, R Raj ^2^

#### ^1^Helsinki University Hospital & University of Helsinki, Department of Anesthesiology, Intensive Care and Pain Medicine and Department of Emergency Care and Services, Helsinki, Finland; ^2^Helsinki University Hospital & University of Helsinki, Helsinki, Finland

**Introduction:**

Traumatic brain injury (TBI) can have devastating neurological, psychological and social sequelae. Increased psychiatric morbidity after TBI has been shown in both adult and the pediatric population. Also, critical illness as such is a risk factor for psychiatric problems in youth. Our aim was to assess risk factors for later being prescribed psychiatric medication in survivors of intensive care unit (ICU)-treated pediatric TBI.

**Methods:**

We used the Finnish Intensive Care Consortium (FICC) database to identify patients 5-17 years of age, treated for TBI in four ICU in Finland during the years 2003-2013. We examined electronic health records and CT scans and collected data on drug prescription after discharge. We used multivariable logistic regression models to find statistically significant risk factors for psychiatric drug reimbursement.

**Results:**

We identified 248 patients of which 46 patients received psychiatric drug prescription (19%) during follow up. The median time to prescription was 14 months after TBI (interquartile range [IQR] 5-31 months). 33 patients received antidepressants, 9 received stimulants and 18 received antipsychotics. Increasing age showed a positive association with all drug prescriptions except for stimulants, where an inverse relationship was observed (Table 1). Using multivariable analyses, we could not find any admission or treatment related factors that significantly associated with being prescribed psychiatric medications. Teenage survivors with moderate disability (Glasgow outcome scale [GOS] 4) showed high numbers of psychotropic drug utilization (45% received any medication, 36% received antidepressants, 24% received antipsychotics).

**Conclusions:**

Our data suggests, that the risk of psychotropic drug prescription after TBI depends on factors other than those related to injury severity or treatment measures. The incidence of drug prescription is especially high in patients with moderate disability.

**Table 1 (abstract P026).** Prescription frequencies in patients categorized by GOS score and age group

GOS, 5-11 years	Any prescription	Antidepressant	Stimulant	Antipsychotic
3, n= 5	2 (40%)	0 (0%)	1 (20%)	1 (20%)
4, n= 10	3 (30%)	1 (10%)	3 (30%)	0 (0%)
5, n= 63	3 (5%)	1 (2%)	1 (2%)	1 (2%)
GOS, 12-17 years				
3, n= 16	5 (31%)	4 (25%)	0 (0%)	2 (13%)
4, n= 42	19 (45%)	15 (36%)	1 (2%)	10 (24%)
5, n= 107	14 (13%)	12 (11%)	3 (3%)	3 (3%)

## P027 The effects of 1-adamantylethyloxy-3-morpholino-2-propanol hydrochloride on the formation of steroid neurotoxicity in rats with brain injury

### A. Semenenko^1^, S. Semenenko^2^, A. Solomonchuk^3^, N. Semenenko^3^

#### ^1^National Pirogov Memorial Medical University, Department of Anesthesiology, Intensive Care and Emergency Medicine, Vinnytsya, Ukraine; ^2^National Pirogov Memorial Medical University, Departament of Clinical Pharmacy and Clinical Pharmacology, Vinnytsya, Ukraine; ^3^National Pirogov Memorial Medical University, Vinnytsya, Ukraine

**Introduction:**

Depending on the nature of the brain injury and the severity of the victims, mortality in traumatic brain injury (TBI) ranges from 5 to 65% [1]. One of the targets for pathogenetic influence on the course of TBI is the use of pharmacological agents that are able to counteract the negative effects of excess concentrations of glucocorticoids on brain.

**Methods:**

The therapeutic effect of new pharmacological derivative 1-adamantylethyloxy-3-morpholino-2-propanol hydrochloride (ademol) in rats with TBI was evaluated for 8 days. The pseudoperated animals and control group received 0.9% NaCl solution and the comparison group received amantadine sulfate. Cortisol levels were used to determine the efficacy of the test drugs in TBI.

**Results:**

In rats treated with ademol, the level of cortisol in the blood ranged from 179 to 188 ng/ml (P5-P95) and was 2.58-fold lower (p<0.05) compared to control pathology group on the 8 day of therapy. Instead, the effect of amantadine sulfate on the level of cortisol in the blood was significantly less than that of ademol. The concentration of cortisol in rats with amantadine sulfate in the blood ranged from 271-280 ng/ml (P5-P95), was 1.73 times lower (p<0.05), compared with the control pathology group, and by 49.2% (p<0.05) exceeded the corresponding value in animals treated with ademol.

**Conclusions:**

Therapeutic treatment of rats with severe TBI with a solution of ademol, preferably better than rats in the group with 0.9% NaCl and amantadine sulfate protect the brain from the formation of steroid neurotoxicity by cortisol (p<0.05).

**References:**

1. Rickels E. Eur J Trauma Emerg Surg 43:729–730, 2017.

## P028 YKL-40 and neuron-specific enolase as prognostic biomarkers in traumatic brain injury

### G Pavlov^1^, M Kazakova^2^, V Dichev^2^, D Vasilev^3^, K Simitchiev^4^, C Stefanov^3^, V Sarafian^2^

#### ^1^Medical University of Plovdiv, Department of Anesthesiology, Emergency and Intensive Care Medicine *University Hospital “St. George” Plovdiv, Bulgaria, Plovdiv, Bulgaria; ^2^Medical University of Plovdiv, Department of Medical Biology, Medical Faculty, Medical University- Plovdiv, Bulgaria, Plovdiv, Bulgaria; ^3^Medical University of Plovdiv, Department of Anesthesiology, Emergency and Intensive Care Medicine, Medical Faculty, Medical University- Plovdiv, Bulgaria, Plovdiv, Bulgaria; ^4^University of Plovdiv, Department of Analytical Chemistry and Computer Chemistry, Faculty of Chemistry, University of Plovdiv, Bulgaria, Plovdiv, Bulgaria

**Introduction:**

Severe traumatic brain injury (TBI) is the leading cause of morbidity and mortality among young people [1]. The aim of the study is to assess how plasma and cerebrospinal levels of YKL-40 [2], independently or in combination with NSE, IL-6, TNF-α and CRP affect the clinical models and the outcome in TBI.

**Methods:**

30 patients with isolated severe TBI admitted to the Intensive Care Unit at the University Hospital “St. George”- Plovdiv from 2017 to 2018 were included. Cerebrospinal fluid (CSF) and plasma were collected on the 24^th^ and 96^th^ hour after the injury. CSF samples were also collected from 15 adult cadavers and served as an age-matched and gender-matched control group. Levels of YKL-40, NSE, IL-6 and TNF-α were analyzed with ELISA. GCS, APACHE III and Marshall Classification were determined prior to randomization and at the 96^th^ hour after admission. Outcome was assessed as in-hospital mortality and at six months mortality.

**Results:**

The CSF level of YKL-40 in the TBI group was significantly higher compared to the control group (p=0.005). CSF levels of NSE are significantly higher compared to plasma concentrations on 24^-th^ hour in TBI patients. Mean plasma levels of YKL-40 and TNF-α were higher compared to CSF, but the difference was feeble. We found statistically significant difference in plasma levels of YKL-40 (p<0.001) and NSE (p<0.001) in patients with short survival. A significant correlation between plasma concentrations of YKL-40, TNF-α and NSE was determined.

**Conclusions:**

Plasma NSE concentration is the major independent variable which influenced the survival of TBI patients. Plasma and CSF YKL-40 levels along with NSE, reflect the inflammatory process and would provide new information about its dynamics in TBI patients.

Acknowledgements: The financial support by the National Science Fund of Bulgaria (Contract DM 03/2 12.12.2016) is gratefully acknowledged.

**References:**

1. Peeters W, et al. Acta Neurochir 157: 1683–1696, 2015.

2. Bonneh-Barkay D, et al. J Neurotrauma 27:1215–1223, 2010.

## P029 Cerebro-vascular perfusion correlation index (CPC) predicts outcomes in severe traumatic brain injury

### D Colombo^1^, G Berdin^2^, A Scerrati^3^, G Golino^4^, E Bonaldi^2^, E Roman-Pognuz^5^, P Persona^6^, MA Martin^2^, S De Rosa^7^

#### ^1^SS. Trinità Hospital,Borgomanero, Borgomanero, Novara, Italy; ^2^San Bortolo Hospital, Department of Anesthesia and Intensive Care Medicine, Vicenza, Italy; ^3^San Bortolo Hospital, Department of Neurosurgery, Vicenza, Italy; ^4^San Bortolo Hospital, Department of Medicine – DIMED, Section of Anesthesiology and Intensive Care Medicine, Vicenza, Italy; ^5^Azienda Sanitaria Universitaria Integrata di Trieste, Hospital of Trieste, Department of Anesthesia and Intensive Care Medicine, Trieste, Italy; ^6^University of Padova, Department of Medicine – DIMED, Section of Anesthesiology and Intensive Care Medicine, Padova, Italy; ^7^San Bortolo Hospital, Department of Intensive Care, Vicenza, Italy

**Introduction:**

Although cerebrovascular pressure reactivity (PRx) well correlate to patient’s outcome [1], it requires continuous monitoring and mobile average calculation for its determination. We therefore hypothesized that a simplified model of variation between mean arterial pressure (MAP) and intracranial pressure ICP over the first three days of admission would have been able to predict patient outcome: we call this new parameter Cerebrovascular Pressure Correlation index (CPC).

**Methods:**

We performed a retrospective observational study of all adult patients with severe TBI admitted to ICU from January 2017 to April 2018 inclusive. All consecutive patients with a clinical need for ICP monitoring were included for analysis. Both for ICP and MAP data were mean value over 2-hours registration, for a total of 12 observations/day, CPC was therefore calculated as the Pearson Correlation Coefficient between ICP values (x axis) and MAP values (y axis), obtaining one single value every 24 hours. Variables included in the model (i.e. CPC, CPP, ICP, systemic glucose, arterial lactate, PaCO_2_, ICP, and internal body temperature) were collected for the first 3 days since trauma.

**Results:**

For the main outcome only the minimum value of CPC fit the regression analysis (P = 0.004). The correspondent ROC curve showed an AUC of 0.80. The associated Youden criterion was ≤0.26 (Sensitivity = 0.90; Specificity = 0.68). Of all the variables considered for the secondary outcome only CPCmin fit the regression model (P =0.03). Table 1 reports the median and IQR range for SG and NSG of all the variables considered in the model.

**Conclusions:**

This observational study suggests that CPC could be a simplified model of variation between MAP and intracranial pressure ICP over the first three days of admission predicting patient outcome.

**References:**

1. Donnelly J, et al. Neurocrit Care 22: 20–25, 2015

**Table 1 (abstract P029).** Odds ratio and significance level for each variable

Variable	Odds ratio	95%CI	P
CPC	0.0029	0.0000 to 0.8336	0.0431
ICP	1.0614	0.9300 to 1.2115	0.3768
CPP	1.0128	0.8519 to 1.2041	0.8854
PaCO2	1.1137	0.8738 to 1.4195	0.3843
Glucose	1.0322	0.9905 to 1.0757	0.1319
Lactate	0.2381	0.0237 to 2.3900	0.2227
Internal body temperature	7.2637	0.4385 to 120.3210	0.1662

## P030 The brain heart interaction - dynamic relationship between variability of heart rate, interventions and outcome in traumatic brain injury

### BA Ianosi^1^, V Rass^1^, A Lindner^1^, P Smielewski^2^, A Ercole^3^, R Beer^1^, A Schiefecker^1^, B Pfausler^1^, N Stocchetti^4^, R Helbok^1^

#### ^1^Medical University of Innsbruck, Neurological Intensive Care Unit, Department of Neurology, Innsbruck, Austria; ^2^Cambridge University, Division of Neurosurgery, Cambridge, United Kingdom; ^3^Cambridge University, Division of Anaesthesia, Cambridge, United Kingdom; ^4^Ospedale Maggiore Policlinico, Department of Anesthesia and Critical Care, Milano, Italy

**Introduction:**

In severe traumatic brain injury (TBI) the heart rate variability (HRV) could inform about severity and predict outcome. In this study we depicted the dynamic response of HRV to iatrogenic interventions.

**Methods:**

Prospectively collected high resolution data of patients from multiple centers with severe TBI from the Intensive Care Unit (ICU) cohort of the CENTER-TBI study were analyzed. Bad outcome was defined as having a Glasgow-Outcome-Scale GOSE between 1-3 at 6 months. HRV parameters were calculated according to international guidelines using a 5-minute sliding window, updated every 10s. Hourly and daily medians were further analyzed

**Results:**

We included 273 patients (11403 h analyzed).80% were male, 46±19 years old and had an initial GCS of 6 (IQR 3-10). 72 had at least one unresponsive pupil. Outcome data was available for 90.1% of patients with 50.8% (N=125/246) having a bad outcome. The ratio of LF (low frequency) to HF (high frequency) power (LF/HF ratio) was lower in patients with at least one unreactive pupil (1.2±1.1 vs.1.5±1.3, p=0.03) and who had a bad outcome (1.1±0.9)vs. good outcome (1.8±1.4) (mean difference 0.69; 95%CI 0.38-0.99, p<0.001) suggesting parasympathetic dominance. During deep sedation we found a lower LF/HF Ratio (0.9±1.1 vs.1.6±1.7, p<0.001), a lower heart rate standard deviation(HR-SD)(p<0.001) and root mean square of successive differences heartbeats(RMSSD) (p=0.006), compared to patients that were not suppressed. During 151 sedation boli the HR-SD increased (mean diff.0.71 95%CI 0.9-0.4, p<0.002) compared to baseline (30min before bolus). During days with induced hypothermia (63d-mild hypothermia; 23d≤35°C, N=42) the LF/HF Ratio was lower (1.6±1.7 vs 1.1±1.1, p=0.02), as were the HR-SD(p=0.01)&RMSSD (p=0.01).Patients with a good outcome but not those with poor outcome showed increased RMSSD during suctioning(reflecting better vagal response) (Figure 1).

**Conclusions:**

HRV reflects severity and ICU interventions. A dynamic analysis during interventions may offer prognostic information.

**Fig. 1 (abstract P030).**
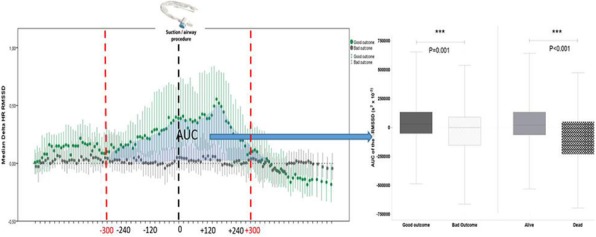
The patients with a good 6-month outcome (GOSE>3) after severe traumatic brain injury showed an increase in root mean square of successive differences between normal heartbeats (RMSSD) (compared to baseline 30 -minutes before tracheal succtioning)

## P031 Temperature and cerebral autoregulation in severe TBI patients

### BA Ianosi^1^, V Rass^1^, A Lindner^1^, M Kofler^1^, A Schiefecker^1^, R Beer^1^, B Pfausler^1^, N Stocchetti^2^, A Ercole^3^, R Helbok^1^

#### ^1^Medical University of Innsbruck, Neurological Intensive Care Unit, Department of Neurology, Innsbruck, Austria; ^2^Ospedale Maggiore Policlinico, Department of Anesthesia and Critical Care, Milano, Italy; ^3^Cambridge University, Division of Anaesthesia, Cambridge, United Kingdom

**Introduction:**

Impaired cerebrovascular reactivity (CAR) after traumatic brain injury (TBI) is a marker for disease severity and poor outcome. It is unclear how dynamic changes in body temperature and fever impact CAR and outcome.

**Methods:**

We calculated the pressure reactivity index (PRx) using the CENTER-TBI high-resolution intensive care unit cohort, as a moving correlation coefficient between intracranial pressure (ICP) and mean arterial pressure (MAP). Minute and hourly values of PRx and temperature were averaged in patients with simultaneous recording of ICP and ABP. Demographic data was based the Core Registry (V2.0). Linear mixed models were calculated based on minute-by-minute data using R with lme4 V1.1-21 and ggeffects V0.9.0. Generalized estimating equation models were used to analyze changes during effervescence (increase of temperature of >1°C within 3 hours).

**Results:**

We assessed high frequency physiological data during 567 days of 102 patients admitted to the ICU with predominantly a closed injury type (N=94/102). Median age was 46 years (IQR 29-62), baseline GCS was 6 (IQR 3-9), and 27% had at least one unreactive pupil. The main measurement site for temperature was the urinary bladder 55/102(54%). Half of the patients (49/102) developed fever(>1h with mean T ≥ 38.3°C) with a total of 834h fever and a median of 9h fever(IQR 4-24) per patient. Of 110 effervescence episodes 30(27%) reached the febrile threshold of 38.3°C which was associated with an increase in PRx from 0.09 (±SD 0.25) at baseline (2h before) to 0.26 (±SD 0.3) during the febrile peak (p=0.014) (Figure 1-A). Linear mixed models showed a quadratic relationship between PRx and temperature (p<0.001) with an increase in predicted PRx with febrile and hypothermic temperatures (Figure 1B).

**Conclusions:**

The association of increasing body temperature with worsening of CAR supports prevention of fever in severe TBI. Prospective studies are needed to further differentiate between mechanisms involved (i.e. inflammation) and central autonomic dysregulation.

**Fig. 1 (abstract P031).**
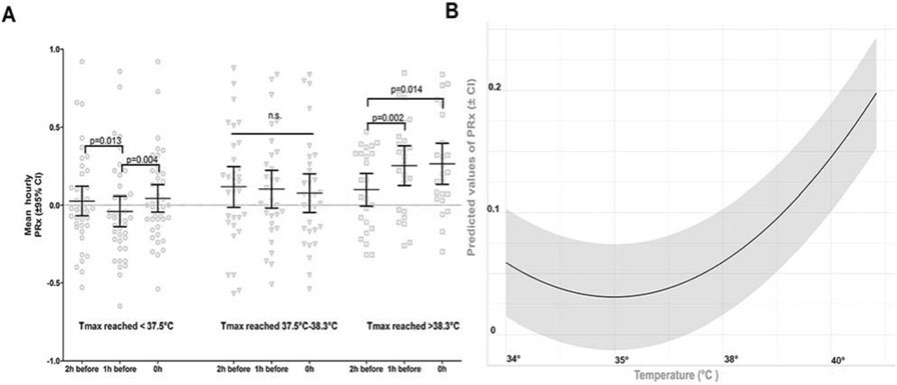
A) Changes in mean PRx during episodes of effervescence (1°C within 3 hours); B) Predicted relationship between PRx and temperature based on mixed linear models

## P032 Brain temperature after traumatic injury: a center-TBI study

### T Birg^1^, F Ortolano^1^, M Carbonara^1^, T Zoerle^1^, R Helbok^2^, Y Savchenko^3^, BA Ianosi^2^, N Stocchetti^1^

#### ^1^Fondazione IRCCSC a’Granda Ospedale Maggiore Policlinico, Neurointensive care unit, Milan, Italy; ^2^University of Innsbruck, Department of Neurology, Innsbruck, Austria; ^3^Burdenko Neurosurgical Center, Neurointensive care unit, Moscow, Russia

**Introduction:**

After traumatic brain injury (TBI) fever is common and may cause secondary brain damage. In this study we investigated the association between changes in brain temperature (BT), intracranial pressure (ICP), and cerebral perfusion pressure (CPP).

**Methods:**

CENTER-TBI is a co-operative effort of several neurotrauma centers in Europe and includes a repository of monitored data sampled at high-frequency (from 200 Hz to 500 Hz). Simultaneous BT, ICP and CPP recordings were studied, focusing on episodes of BT changes (delta BT ≥0.5 °C, lasting 15 minutes - 3 hours) up or down-ward. Using the linear correlation coefficient episodes were coded as responding (R), when there was a clear association between ICP and BT (Pearson’s R value ≥0.5) or non-responding (NR) when the correlation coefficient was <0.5.

**Results:**

Twenty-one patients were selected for the study for a total of 2435 monitoring hours. The mean duration of monitoring was 86 hours. All patients reached a BT above 38° and experienced at least one episode of ICP above 20 mmHg. A CPP lower than 60 mmHg (often of very short duration) has been recorded in all patients. 149 episodes (79 for BT elevation and 70 for BT reduction) were identified. During BT elevations, the increase in temperature was accompanied by ICP increases in slightly more than half episodes (43 R (54%) vs 37 NR (46%)). During BT reduction there were 32 R (46%) and 38 NR (54%) episodes.

**Conclusions:**

Patients after TBI usually develop BT> 38° soon after the injury. The BT may influence brain physiology, as reflected by ICP, through vascular responses (respectively vasodilation/vasoconstriction). Our analysis identified potential ICP and CPP variations connected to BT changes in approximately half of episodes. However, it has to be noted that both ICP and CPP have been actively manipulated in the ICU, potentially blunting the biological responses due to BT.

## P033 Subclinical AKI in severe traumatic brain injury

### S De Rosa^1^, G Golino^2^, G Villa^3^, S Samoni^4^, M De Cal^4^, MA Martin^5^, G Berdin^5^, P Persona^2^, P Navalesi^2^, C Ronco^6^

#### ^1^San Bortolo Hospital, Department of Intensive Care, Vicenza, Italy; ^2^University of Padova, Department of Medicine – DIMED, Section of Anesthesiology and Intensive Care Medicine, Padova, Italy; ^3^University of Florence, Department of Anesthesia and Intensive Care Medicine, Florence, Italy; ^4^San Bortolo Hospital, Department of Nephrology, Vicenza, Italy; ^5^San Bortolo Hospital, Department of Anesthesia and Intensive Care Medicine, Vicenza, Italy; ^6^San Bortolo Hospital, Department of Medicine, University of Padova, Padova-Italy, Vicenza, Italy

**Introduction:**

Acute kidney injury (AKI) is relatively common in patients with severe traumatic brain injury (sTBI) and it can contribute to morbidity and mortality [1]. NephroCheck is a point-of-care urine test that flags two biomarkers that indicate if a critically ill patient is at risk for AKI. We investigated the incidence of subclinical AKI in patients with sTBI.

**Methods:**

We performed a prospective observational study of all adult patients with severe TBI admitted to ICU from January 2017 to April 2018 inclusive. All consecutive patients with a clinical need for ICP monitoring were included for analysis. Urine samples of severe TBI patients was collected at ICU admission from 33 patients to measure NephroCheck (NC) test [IGFBP7]x[TIMP-2] was performed using the NephroCheck® Astute1 40 ™ meter. Serum creatinine was collected at admission, during the first three days, at ICU dismission and 60-days follow up to assess renal recovery. The diagnosis of AKI was based on KDIGO criteria. Hemodynamics, electrolytes, PEEP, P/F, kind of fluid administered, Fluid Balance, % Fluid overload, length of stay, The Sequential Organ Failure Assessment score, injury severity scores and mortality were collected.

**Results:**

A total of 15 patients (45%) presented a median NC higher values at ICU admission. One patient with positive NC value experienced AKI at 24 hrs. The positive NC group had more plasma transfusion (p-value 0.03) and a lower median hematocrit at 24 hrs (p-value 0.013), but similar hospital length of stay (p=0.17) and mortality rate (p=0.80)

**Conclusions:**

NC at ICU admission identifies subclinical AKI in TBI patients and it maight be used to predictclinical AKI. Hemodilution (but not fluid overload) seems to be associated with development of subclinical AKI. Higher NC at ICU admission is not associated with worst long-term outcome in TBI patients.

**References:**

1. Moore EM et al. Ren Fail 32:1060-5, 2010

## P034 Benchmarking outcomes after severe traumatic brain injury with the CRASH and IMPACT prediction models: a single center analysis in the Netherlands

### WR Van de Peppel^1^, HF Lingsma^2^, I Haitsma^3^, M Van der Jagt^4^

#### ^1^Erasmus MC - University Medical Center, Departments of Intensive Care Adults, Rotterdam, Netherlands; ^2^Erasmus MC - University Medical Center, Department of Public Health, Rotterdam, Netherlands; ^3^Erasmus MC - University Medical Center, Department of neurosurgery, Rotterdam, Netherlands; ^4^Erasmus MC - University Medical Center, Department of Intensive Care Adults, Rotterdam, Netherlands

**Introduction:**

Severe traumatic brain injury (TBI) is considered a serious public health problem in Europe. Partly because of the heterogeneity of TBI, considerable uncertainty may exist in the expected outcome of patients. The International Mission for Prognosis and Analysis of Clinical Trials in TBI (IMPACT) and the Corticosteroid Randomization After Significant Head Injury (CRASH) prediction models are considered the most widely validated prognostic models [1,2]. However, studies using these prediction models for benchmarking of outcomes have been scarce. We aimed to compare actual outcomes in a TBI cohort of critically ill TBI patients with predicted outcomes in a quality of care initiative in an Academic Hospital.

**Methods:**

In this retrospective cohort study, we included consecutively admitted TBI patients to the ICU Adults of Erasmus MC, University Medical Center, Rotterdam, The Netherlands between January 2018 and February 2019.

**Results:**

We included 87 patients with TBI. 14-day mortality was 25%, six-month mortality was 36% and six-month unfavourable outcome was 50%. The IMPACT core+CT+lab model predicted 34% 6-month mortality (vs 35% actual, p=0.89) and 51% unfavourable outcome (vs 50% actual, p=0.9). The 14-day mortality prediction by CRASH prognosis calculator was 43% versus actual 14-day mortality of only 25% (p=0.01), whereas 6-month unfavourable outcome prediction by CRASH was 67% (vs. 50% actual, p=0.02) (Figure 1).

**Conclusions:**

The IMPACT model, although developed more than a decade ago, seemed appropriate for benchmarking purposes in this single center cohort in the Netherlands, while CRASH predictions were less applicable to our setting.

**References:**

1. Perel P et al. BMJ 23 :425-429, 2003

2. Steyerberg EW et al. PLoS Med 5:e165, 2008

**Fig. 1 (abstract P034).**
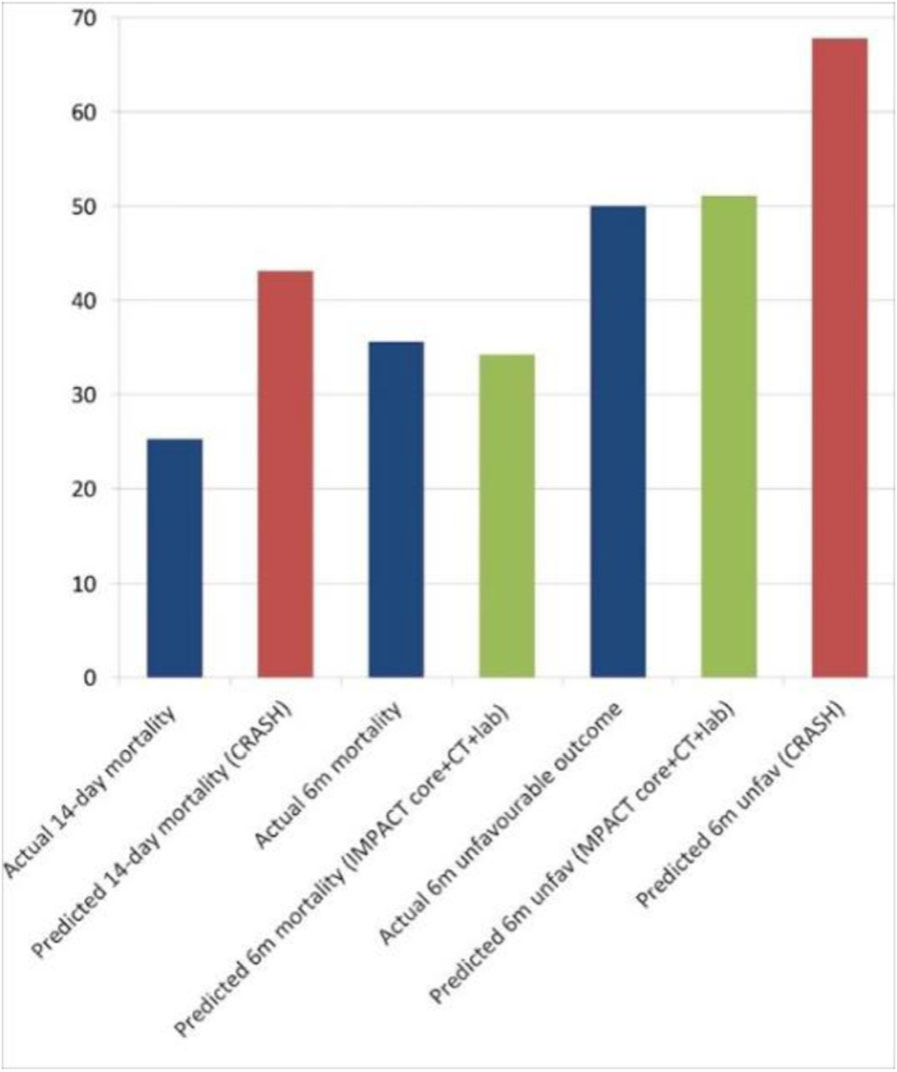
Actual and predicted outcome according to CRASH and IMPACT

## P035 Neuroprognostication following out of hospital cardiac arrest

### R Davidson^1^, T Bagnall^2^, M Ahmed^3^

#### ^1^St George´s Hospital Cardiothoracic Intensive Care, Atkinson Morley Wing, London, United Kingdom; ^2^Kingston Hospital, Cardiology, London, United Kingdom; ^3^St George´s Hospital Cardiothoracic Intensive Care, London, United Kingdom

**Introduction:**

Out of hospital cardiac arrest (OHCA) continues to be associated with significant mortality and morbidity. Centralisation of care has considerably improved patient survival but has resulted in increased morbidity in the form of neurological deficit. Accurate neurological prognostication remains challenging incorporating repeated clinical examination and ancillary investigations [1, 2].

**Methods:**

Data was collected retrospectively and analysed for 96 patients admitted post OHCA from October 2018 to October 2019. Patient arrest demographics were collected in conjunction with extensive inpatient investigation findings including CT, traditional pupil assessment, pupillometry and EEG.

**Results:**

50% of patients survived to hospital discharge. Patients presenting in a shockable rhythm continue to have higher survival rates (Table 1). 53% of patients who received immediate CPR survived to hospital discharge in comparison to 41% of patients who did not receive immediate CPR. 73% of patients underwent non-contrast CT head. 74% of patients had traditional pupillary examination performed on arrival. Pupillometry was introduced in December 2018; 31 out of a possible 85 patients had pupillometry during their inpatient stay. EEG was undertaken in 11% of cases.

**Conclusions:**

Our data shows receiving immediate CPR and presenting with a shockable rhythm remain positive prognostic factors. CT head as a stand-alone prognostic modality is unreliable with 14% of patients who survived to discharge, with intact neurology, had an admission CT head reported as hypoxic brain injury. A new neuroprognostic strategy is required in our unit that adds further certainty to likely clinical outcome. This includes increased use of tests such as EEG and pupillometry and the introduction of biomarkers such as neuron specific enolase, somatosensory evoked potential testing and magnetic resonance imaging.

**References:**

1. Sandroni et al. Crit Care 22:150, 2018

2. Eertmans et al. Scand J Trauma Resusc Emerg Med 26:93, 2018

**Table 1 (abstract P035).** Survival to discharge based on presenting rhythm

Presenting rhythm	Total patients	Survival to discharge
VF/VT	62	65%
PEA/asystole	33	24%

## P036

**Withdrawn**

## P037 Audit of end of life care and neuroprognostication in the intensive care department at Bristol Royal Infirmary

### KJ Richardson, M Thomas

#### Bristol Royal Infirmary, Intensive Care Unit, Bristol, United Kingdom

**Introduction:**

Post-resuscitation care of patients following an out-of-hospital cardiac arrest (OOHCA) is set out by the UK Resuscitation Council [1]. This is in line with the European Resuscitation Council guideline [2]. The aim of this audit was to review compliancy to this guideline at the Intensive care unit at the Bristol Royal Infirmary .

**Methods:**

A retrospective audit was performed over a six-month period in adults who were admitted to the intensive care unit at the BRI following an OOHCA whom later died during that admission (41 patients). The focus was on whether the Neuroprognostication and End-of-life (EOL) care received was as per the standards set by the UK Resuscitation Council.

**Results:**

The main neuroloical examinations documented were pupillary reflex (100%), corneal reflex (75%) and motor response to pain (100%). 61.5% of patients received an SSEP analysis >72 hours post-ROSC, 81.5% underwent an EEG and 66.7% had >2 serum neuron-specific enolase measurements recorded. All patients (100%) underwent a CT Head during their admission. 5.6% of patients were referred to Palliative Care during their admission. 22% of patients were prescribed all EOL medications. Most common prescriptions included Alfentanil (90.2%) and Midazolam (58.5%). Finally, 100% of appropriate patients were referred to be potential organ donors.

**Conclusions:**

The audit reflected our local practice and that some parameters were not being maintained as set by UK Resuscitation guideline. Multiple modalities of documentation posed a problem in analysing the care that these patients received.

**References:**

1. Resuscitation council UK BHF, NHS England. Consensus Paper on out-of-hospital cardiac arrest in England. 2015.

2. Nolan JP et al. Resuscitation 95:202-22, 2015

## P038 Target temperature management after out of hospital cardiac arrest

### P Liddicoat, M Ahmed, A Obhrai

#### St George's Hospital, Intensive Care, Tooting, United Kingdom

**Introduction:**

Targeted temperature management (TTM) and fever avoidance improves mortality and neurological outcome post out of hospital cardiac arrest (OHCA) [1]. Patients follow a protocol from admission: TTM for 72 hours (36°C for 24h and then normothermia for 48h). We looked at the rationale for commencing TTM, temperature control devices, if we met temperature guidelines and the reasons for TTM failure.

**Methods:**

We retrospectively analysed patients who had been coded OHCA on iCLIP (our electronic patient record) between 30/06/2018 to 30/06/19. We analysed ICU charts for recorded temperatures (< 72hrs) for TTM patients. We then accessed notes through iCLIP and assessed rationale for TTM commencement and reasons for failure. We compared our results to our ICU standards, based on current consensus and the TTM1 results [1]. We also looked at the effectiveness of a clerking proforma (Feb 2019) on documentation.

**Results:**

95 patients were admitted post OHCA. 76 were initiated on TTM; of the 19 not: 10 patients were critically unstable, while 9 patients had good GCS on ROSC. Of recorded arrest rhythms 57/71 (80%) of TTM patients were shockable while 9/15 (60%) of non TTM patients were shockable. Of the TTM patients: 6 had surface cooling devices and 70 had intravascular cooling devices. 21/73 (29%) of TTM patients failed TTM adherence by either having a mean of >0.5°C OR a spike of >1°C above target temperature. 13/ 21 (62%) had predictable medical causes of TTM failure: Aspiration pneumonia or myoclonic jerks. The other 8 patients had process-based failures: going for PCI or being warmed early. The proforma has had a marked improvement on documentation.

**Conclusions:**

These results highlight that raised temperatures in TTM are predictable. To improve this we will focus on recognition of these risk factors and establish means to minimise their impact. We have also shown the benefit of an established clerking proforma.

**References:**

1. Nielsen N et al. N Engl J Med 369:2197-2206, 2013.

## P039 Serum GFAP and UCH-L1 for the prediction of long term neurological outcome in comatose out-of-hospital cardiac arrest patients

### F Ebner^1^, M Moseby-Knappe^2^, N Mattsson^2^, J Undén^3^, S Ullén^4^, T Cronberg^2^, N Nielsen^5^

#### ^1^Helsingborg Hospital, Department of Clinical Sciences Lund, Anesthesia and Intensive Care, Helsingborg, Sweden; ^2^Skane University Hospital, Lund University, Skane University Hospital, Department of Clinical Sciences Lund, Neurology, Lund, Sweden; ^3^Halland Hospital, Lund University, Halland Hospital, Department of Clinical Sciences Lund, Anesthesia and Intensive Care, Halmstad, Sweden; ^4^Skane University Hospital, Clinical Studies Sweden, Skane University Hospital, Lund, Sweden; ^5^Helsingborg Hospital, Lund University, Helsingborg Hospital, Department of Clinical Sciences Lund, Anesthesia and Intensive Care, Helsingborg, Sweden

**Introduction:**

The prognostication of neurological outcome in comatose out-of-hospital cardiac arrest (OHCA) patients is an integral part of post cardiac arrest care. Biochemical biomarkers released from cerebral cells after hypoxic-ischemic injury represent potential tools to increase accuracy in predicting outcome after OHCA. Currently, only neuron-specific enolase (NSE) is recommended in European prognostication guidelines. In this study, we present the release dynamics of GFAP and UCH-L1 after OHCA and evaluate their prognostic performance for long-term neurological outcome in OHCA patients.

**Methods:**

Serum GFAP and UCH-L1 were collected at 24, 48 and 72 h after OHCA. The primary outcome was neurological function at 6-month follow-up assessed by cerebral performance category scale (CPC), dichotomized into good (CPC 1-2) and poor (CPC 3-5). Outcome prognostic performance was investigated with receiver operating characteristics (ROC) by calculating the area under the receiver operating curve (AUROC) and compared to NSE.

**Results:**

717 of 819 included patients had at least one serum GFAP or UCH-L1 value at 24, 48 or 72 h after OHCA. GFAP and UCH-L1 levels were significantly elevated in patients with poor outcome. GFAP and UCH-L1 discriminated excellently between good and poor neurological outcome at all time-points (AUROC GFAP 0.88-0.89; UCH-L1 0.86-0.87) and overall predictive performance measured by AUROC of GFAP and UCH-L1 was superior to NSE (AUROC 0.76-0.85) (Figure 1). However, the ROC at the highest specificities of UCH-L1 and GFAP overlap those of NSE and comparing the sensitivities for UCH-L1 and GFAP with those of NSE for the highest specificities (>95%) revealed higher sensitivities for NSE than for UCH-L1 and GFAP at 48 and 72 h.

**Conclusions:**

GFAP and UCH-L1 predict poor neurological outcome in patients after OHCA excellently and with a higher overall accuracy than NSE, but both biomarkers perform inferior to NSE at specificities over 95% at 48 and 72 h limiting their clinical use to guide decisions on prognosis.

**Fig. 1 (abstract P039).**
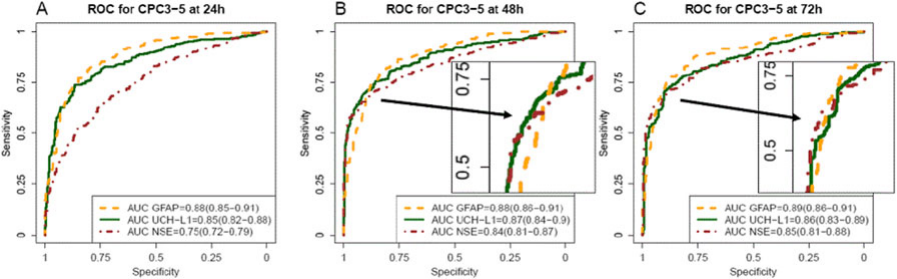
A-C, Receiver-operating characteristic analyses for prediction of Cerebral Performance Category (CPC) Scale 1-2 vs CPC 3-5 at 6-months follow-up for serum samples collected at 24, 48 and 72 hours. The enlarged sections in B and C show the overlap of GFAP, UCH-L1 and NSE at high specificities at 48 and 72 hours

## P040 Blood pressure after cardiac arrest and severity of hypoxic-ischemic encephalopathy

### C Endisch^1^, S Preuß^2^, C Storm^3^, CJ Ploner^1^, C Leithner^1^

#### ^1^Charité Universitätsmedizin Berlin, Department of Neurology, AG Emergency and Critical Care Neurology, Berlin, Germany; ^2^Charité Universitätsmedizin Berlin, Departement of Cardiology, Berlin, Germany; ^3^Charité Universitätsmedizin Berlin, Department of Nephrology and Internal Intensive Care Medicine, Berlin, Germany

**Introduction:**

Blood pressure management in post cardiac arrest (CA) patients ensures sufficient cerebral perfusion to avoid secondary brain injury. In previous studies, lower mean arterial pressure (MAP) was associated with poor clinical outcome. However, the relationship between hypotension and severity of hypoxic-ischemic encephalopathy (HIE) remains uncertain.

**Methods:**

We retrospectively analyzed MAP during the first week in three CA patient groups: (1) Patients surviving with no/mild HIE – cerebral performance category (CPC) 1. (2) Patients surviving with severe HIE – unresponsive wakefulness syndrome (CPC 4). (3) Deceased patients with post-mortem brain autopsy. In these autopsy patients, we used the Selective Eosinophilic Neuronal Death (SEND) classification to quantify the histopathological severity of HIE.

**Results:**

We included 354 autopsy patients, 118 had histopathological severe and 236 patients no/mild HIE. Between autopsy patients with no/mild HIE and severe HIE, there was no difference in mean MAP during the first week. However, no/mild HIE autopsy patients with post-CA regain of consciousness had a mean MAP of 75 mmHg compared to 65 mmHg in those never conscious before death. We found no difference in MAP between 216 patients with full neurological recovery (CPC 1) and 57 surviving with unresponsive wakefulness syndrome (CPC 4). Hypothermia induced small reversible hypotension in all groups.

**Conclusions:**

Contradicting previous studies, we found no difference in post-CA MAP of patients with histopathological and clinical evidence of presence and absence of HIE. No/mild HIE patients with post-CA regain of consciousness before death showed high-normal blood pressure stability. This may indicate a blood pressure effect on neuronal function rather than structural integrity. Importantly, extracerebral causes for loss of consciousness and mortality need to be considered.

## P041

**Withdrawn**

## P042

**Withdrawn**

## P043 P25/30 somatosensory evoked potentials and neurological outcome in comatose survivors of out of hospital cardiac arrest

### N Nikitas^1^, L Nye^2^, C Bigham^1^, N Broomfield^2^, A MacCormick^1^, L Lankester^1^, N Gourd^1^, R Squire^3^, E Dunn^2^, N Hudson^2^

#### ^1^University Hospitals Plymouth NHS Trust, Department of Intensive Care Medicine, Plymouth, United Kingdom, ^2^University Hospitals Plymouth NHS Trust, Department of Neurophysiology, Plymouth, United Kingdom, ^3^University Hospitals Plymouth NHS Trust, Department of Emergency Medicine, Plymouth, United Kingdom

**Introduction:**

The objective of the study is to analyse the 20-30msec complex of the somatosensory evoked potentials [SSEP] in comatose survivors of out of hospital cardiac arrest [OHCA], to identify the patterns of this complex and to test whether the addition of P25/30 in analysis could improve N20’s positive predictive value for favourable outcome. Previously it was suggested that P25/30 have a role in the prognosis of neurological outcome after OHCA [1].

**Methods:**

Single-centre, prospective, observational study. Patients: Adult comatose survivors of OHCA treated with organ support, neuroprotection and targeted temperature [T] management for normothermia. SSEP recorded at 24-36h post ROSC and interpreted by two interpreters for P25/30 and N20 in different amplitude thresholds. Neurological outcome at hospital discharge was assessed by the cerebral performance category [CPC] score.

**Results:**

First 30 patients: 18 male, 12 female, mean age 62.3 years. OHCA rhythm VF in 17, asystole in 13. Cause of OHCA: cardiac in 20 and non-cardiac in 10. Mean anoxic time 30min. Mean T in the first 24-36 hours 35.9. Among survivors with unfavourable outcome [CPC 3-5] 14 had bilaterally absent P25/30 and N20, 2 bilaterally present P25/30 and bilaterally absent N20, 1 bilaterally present P25/30 and N20, 1 bilaterally present 25/30 and unilaterally present N20 and 1 unilaterally present P25/30 and N20. All 11 survivors with favourable outcome [CPC 1-2] had bilaterally present P25/30 and N20.

**Conclusions:**

Early results suggest the presence of variable patterns of 20-30msec SSEP complex in comatose survivors of OHCA. As the number of enrolled participants increase, it is yet to be determined whether the presence of P25/30 could potentially increase the positive predictive value of N20 for the favourable neurological outcome.

**References:**

1. Kim SW et al. Crit Care Med 46:545-551, 2018

## P044 Relationship between shockable rhythm and long-term outcome in pediatric out-of-hospital cardiac arrest in Rotterdam, the Netherlands: a 16 year observational study.

### M Albrecht^1^, RCJ De Jonge^1^, M Hunfeld^1^, JAE Kammeraad^1^, XRJ Moors^1^, VM Nadkarni^2^, L van Zellem^1^, CMP Buysse^1^

#### ^1^Erasmus MC – Sophia Children's Hospital, Intensive Care and Department of Pediatric Surgery, Rotterdam, the Netherlands; ^2^Department of Anesthesiology and Critical Care Medicine, The Children's Hospital of Philadelphia, University of Pennsylvania, Philadelphia, PA., USA

**Introduction:**

Local chain-of-survival improvements affect P-OHCA survival [1-5]. Also initial rhythm in P-OHCA is an important predictor of survival [1,4]. Little is known about the relationship between initial rhythm in P-OHCA and long-term outcome [6-8]. Our aim was to establish the relation between shockable rhythm and favorable long-term outcome in POHCA.

**Methods:**

All children aged 1 day-18 years who experienced non-traumatic OHCA between 2002-2017 and were admitted to the Sophia Children’s Hospital in Rotterdam were included. Long-term outcome was determined using a Pediatric Cerebral Performance Category score at the longest available follow-up interval. The primary outcome measure was survival with favorable neurologic outcome, defined as PCPC 1-2 or no difference between pre- and postarrest PCPC. The association between shockable rhythm and the primary outcome measure was calculated in a multivariable regression model, adjusted for the pre-defined variables.

**Results:**

From the 329 patients included in the 16 year study period 126 (38%) patients survived to hospital discharge of which 99 patients (30%) had favorable neurologic outcome (median follow-up duration of 24 months). The rate of favorable neurologic outcome rose from 17% in 2002 to 52% in 2017 (p < 0.001 for trend) (Fig. 1). In adjusted analysis initial shockable rhythm (OR 7.2 [95%CI 2.2-23.7]), initial unknown rhythm (OR 10.2 [95%CI 3.5-29.2]) and the year of event (OR 1.2 [95%CI 1.2-1.4]) were associated with favorable long-term neurologic outcome.

**Conclusions:**

The odds of favorable neurologic outcome at the longest follow-up duration were significantly higher after a shockable initial and unknown rhythm. Secondly, trend analysis showed an increase in AED defibrillation and shorter CPR duration. This was followed, finally, by a rise in ROSC, survival to hospital discharge and favorable neurologic outcome rate.

**References:**

1. Atkins DL et al. Circulation 2009;119:1484-91.

2. Kitamura T et al. Lancet 2010;375:1347-54.

3. Tijssen JA et al. Resuscitation 2015;94:1-7.

4. Fukuda T et al. Resuscitation 2017;111:1-7.

5. Naim MY et al. JAMA Pediatr 2017;171:133-41.

7. Michiels E et al. Resuscitation 2016;102:122-6.

8. Meert KL et al. Pediatr Crit Care Med 2016;17:e543-e50.

**Fig. 1 (abstract P044).**
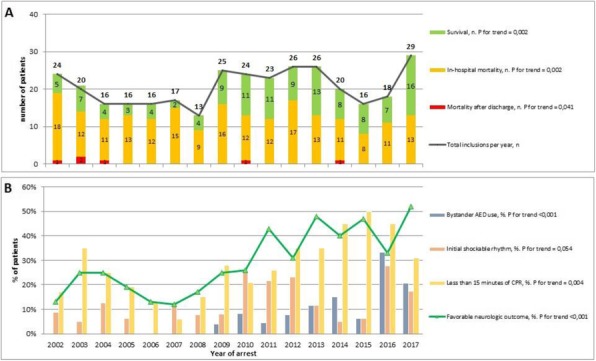
Patient and arrest characteristics over time. A. The total amount of mortality as a stacked bar: in light-red the number of patients who deceased at scene, in green the number of patients deceased during admission, in red patients who died after discharge. The grey line is the total number of inclusions. B. The rate of bystander AED use, rate of initial shockable rhythm, rate of less than 15 minutes of CPR and rate of favorable neurologic outcome over time. P for trend significant for bystander AED use, less than 15 minutes of CPR and favorable neurologic outcome. Trend analysis performed using binary logistic regression for dichotomous data (and a Kruskal-Wallis test for non-normally distributed continuous data)

## P045 Acute kidney injury in cardiac arrest patients

### S Bazzano^1^, G Villa^2^, G Golino^3^, S Samoni^4^, M De Cal^4^, S Marcante^5^, M Martin^5^, R Vaschetto^1^, C Ronco^4^, S De Rosa^6^

#### ^1^Università degli studi del PIemonte Orientale, Department of Anesthesia and Intensive Care Medicine, Novara, Italy; ^2^University of Florence, Department of Anesthesia and Intensive Care Medicine, Florence, Italy; ^3^University of Padova, Department of Medicine – DIMED, Section of Anesthesiology and Intensive Care Medicine, Padova, Italy; ^4^San Bortolo Hospital, Department of Nephrology, Vicenza, Italy; ^5^San Bortolo Hospital, Department of Anesthesia and Intensive Care Medicine, Vicenza, Italy; ^6^San Bortolo Hospital, Department of Intensive Care, Vicenza, Italy

**Introduction:**

Cardiac arrest (CA) is a global ischemia-reperfusion injury syndrome [1]. The aim of the study is to evaluate the incidence of AKI in comatose patients resuscitated from CA.

**Methods:**

We performed retrospective study of consecutive comatose patients resuscitated from CA and admitted to our ICU from January 2013 to May 2019 inclusive. Data obtained at baseline, 24 hrs, 48 hrs, and 72 hrs included: temperature trend and rate, serum creatinine,MAP, VIS, PEEP, diuretic use, urine output, fluid balance (FB). AKI was defined according to KDIGO criteria.

**Results:**

Thirty-six patients were treated with TH 33°C out of 46 ICU admissions (78%). Fifty-four patients were treated with TTM 35°C out of 106 ICU admission (51%) while fifty-two were not treated with temperature management (49%). The incidence of AKI was 17.35 at 24h for NO TTM group, 13.9% for TH group and 14.9% for TTM group. At 48h the incidence of AKI increased at 41.7% for TH, 37.1% for TTM while in NO TTM group increased less (18.8%). At 72h the incidence of AKI was 33.3% for TH group, 27.8% for TTM group and 32.7% for NO TTM group.SCr at baseline, at admission at 24 and 48h was not significant different in the three groups. Median cumulative FB was significant different in the TH group at 48h 1522 (488 – 2236) compared to 382 (- 397 – 1553) in TTM group and 228 ( -393 – 1530) in NO TTM group (p< 0.003). Twenty-four patients (16.9%) required RRT in the overall population. Urinary TIMP-2 e IGFBP7 was 1.54 (0.8 – 3.46) in TTM group and 1 (0.19 – 2.18) in TH group (p = 0.105).

**Conclusions:**

AKI occurred in more then 30% of patient after CA even if there are no clear impairment of serum creatinine, AKI can show in a subclinical way and determine an alteration of FB, hemodynamic function, and chronic renal loss of function

**References:**

1. De Rosa S et al. BMC Nephrol 18:376, 2017.

## P046 Socioeconomic status is associated with early coronary angiography after out-of-hospital cardiac arrest

### R Lagedal^1^, M Jonsson^2^, L Elfwén^3^, D Smekal^4^, P Nordberg^2^, S James^5^, S Rubertsson^4^

#### ^1^Department of Surgical Sciences, Anaesthesia and Intensive Care, Uppsala University, Uppsala, Sweden; ^2^Department of Surgical Sciences, Anaesthesia and Intensive Care, Department of Medicine, Center for Resuscitation Science, Karolinska Institute, Solna, Sweden; ^3^Department of Surgical Sciences, Anaesthesia and Intensive Care, Department of Clinical Science and Education, Södersjukhuset, Karolinska Institute, Sweden, Uppsala, Sweden; ^4^Department of Surgical Sciences, Anaesthesia and Intensive Care, Department of Surgical Sciences/Anesthesiology and Intensive Care Medicine, Uppsala University, Uppsala, Sweden; ^5^Department of Surgical Sciences, Anaesthesia and Intensive Care, Department of Medical Sciences, Cardiology, and Uppsala Clinical Research Center Uppsala University, Uppsala, Sweden

**Introduction:**

Low socioeconomic status is associated with worse outcome after cardiac arrest. This study aims to investigate if patients´ socioeconomic status impacts the chance to receive early coronary angiography after cardiac arrest.

**Methods:**

In this nationwide retrospective cohort study, 4011 patients admitted alive after out-of-hospital cardiac arrest (OHCA) and registered in the Swedish Registry for Cardiopulmonary Resuscitation were included. Individual data on income and educational level, prehospital parameters, coronary angiography results and comorbidity were linked from other national registers.

**Results:**

In the unadjusted model there was a strong correlation between income level and rate of early coronary angiography where 35% of patients in the highest income quartile received early angiography compared to 15% in the lowest income quartile. When adjusting for confounders (educational level, sex, age, comorbidity and hospital type) there were still higher chance of receiving early coronary angiography with increasing income, OR 1.31 (CI 1.01-1.68) and 1.67 (CI 1.29-2.16) for the two highest income quartiles respectively compared to the lowest income quartile. When adding potential mediators to the model (initial rhythm, location, response time, bystander cardiopulmonary resuscitation and if the arrest was witnessed) no difference in early angiography related to income level where found. The main mediator was initial rhythm (Figure 1).

**Conclusions:**

Higher income is strongly related to the rate of early coronary angiography after OHCA. This finding is consistent when adjusting for known confounders. However, the association between income and early angiography seems to be mediated by initial rhythm. Patients with low income more often presents with non-shockable rhythms which lowers the likelihood to undergo early coronary angiography.

**Fig. 1 (abstract P046).**
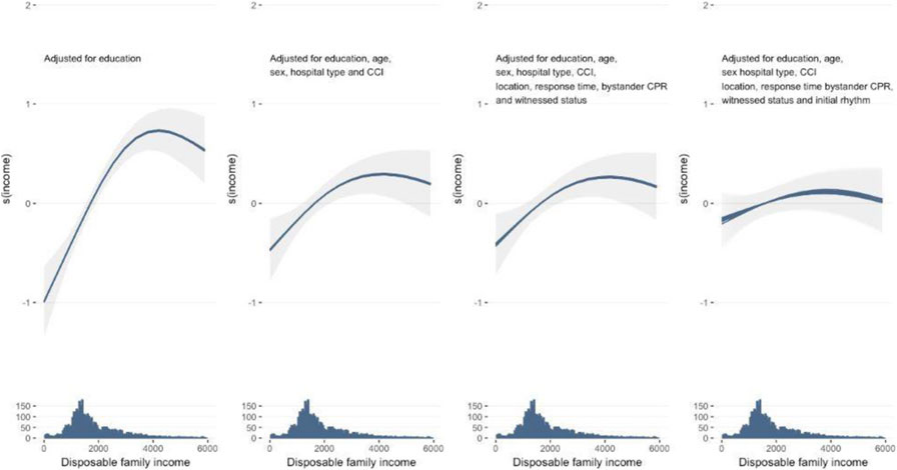
Correlation between disposable income and rate of early coronary angiography. Confidence interval in gray. Values above 0 on y-axis indicate increased rate of early coronary angiography. Second figure adjusted for confounders. Third and fourth figure adjusted for increasing numbers of potential mediators as stated in Figure

## P047 Effect of simulation teaching of cardiopulmonary resuscitation for nursing

### V Spatenkova^1^, I Danilova^2^, Z Jindrisek^2^, M Fronkova^2^, I Veverkova^2^

#### ^1^Faculty of Health Studies, Technical Faculty of Liberec, Neurocenter, NICU, Liberec, Czech Republic; ^2^Faculty of Health Studies, Technical Faculty of Liberec, Liberec, Czech Republic

**Introduction:**

Simulation teaching is a modern type of critical care (CC) education. The aim of this study was to assess the effect of simulation teaching of CC on a comparison of final examination in different model levels of cardiopulmonary resuscitation (CPR) after the first (CC1) and third, final CC3.

**Methods:**

The success rate of CPR was tested in prospective study (2017-2018) on two groups with a total of 66 students in CC1 and CC3 at the Faculty of Health Studies. Three semester of undergraduate nursing simulation education (lectures and training) used the Laerdal SimMan 3G. Quality of CPR was evaluated according to 4 parameters: compression depth, compression rate, chest release and time of correct frequency. We tested if CPR quality differed between the two groups. For the compression depth and compression rate parameters, first the conformity of variance was verified and then two-sample t-test. As the chest release and time of correct frequency are recorded as percentages, the Wilcoxon rank-sum test was conducted for these parameters. To ensure good resuscitation, all recorded parameters must be properly performed during resuscitation. Thus, pivot tables were used to generate statistics and test if the number of correctly performed resuscitation parameters for CC1 and CC3 differ.

**Results:**

The compression depth parameter was statistically significantly higher for the CC3 than for the CC1 (p=0.016). There were no differences in compression rate (p=0.210), chest release (p=0.514) and time of correct frequency (p=0.586). It was also tested how many of the parameters were performed correctly by students at CPR. The chi-square test shows the relative frequency of CPR success is higher for the CC3 group than for the CC1 group. At least 3 out of 4 parameters were correctly performed by 13% of CC1 students compared to 28% of CC3 students.

**Conclusions:**

The study showed a significant improvement of CPR in the final CC3 and supported the three semester simulation education.

## P048

**Withdrawn**

## P049 Changes in blood gases during intraoperative cardiac arrest

### JJ Wang, R Borgstedt, S Rehberg, G Jansen

#### Protestant Hospital of the Bethel Foundation, Anaesthesiology, Intensive Care and Emergency medicine, Transfusion medicine and Pain therapy, Bielefeld, Germany

**Introduction:**

Blood gas analysis (BGA) is a common approach for monitoring the homeostasis during surgery. While it is well known that cardiac arrest (CA) leads to circulatory collapse and disturbances in homeostasis, little is known about changes of blood gas during peri-operative CA.

**Methods:**

We retrospectively analysed patients ≥18 years who suffered from peri-operative CA during non-cardiac surgery from 01/2014 to 12/2018. Peri-operative CA was defined as need for cardiac compression during anaesthesia care. Collected data included pH, PaCO2, PaO2, return of spontaneous circulation (ROSC) and 30-day mortality after CA.

**Results:**

Within the study period, we observed 56 peri-operative CA (m=35, f=21; age 69±16) during 62742 anaesthesia procedures (ROSC occurred in 38 patients (68%). 30 days after CA, the mortality was 45% (n=17), 23% (n=13) were discharged, and 14% (n=8) still in hospital. 87% (n=49) of CA patients had an invasive blood pressure monitoring, 52% (n=29) had BGA before and 66% (n=37) during peri-operative CA. Prior to CA, the average values were: pH 7.3±0.1, PaCO2 39±8 and PaO2 225±107. During CA, the average values were pH 7.2±0.2, PaCO2 50±17 and PaO2 215±138. Table 1 shows the distributions of blood gas before and during CA. There were no statistical differences between the groups (pH: p=0.4; PaCO2: p=0.19; PaO2: p=0.21).

**Conclusions:**

Hypercapnia and respiratory acidosis is common in peri-operative CA. These data suggests inadequate ventilation during peri-operative resuscitation. Further studies should focus on its impact on the outcome.

**Table 1 (abstract P049).** Changes in blood gases during intraoperative cardiac arrest

Blood gases	Before CA (n[%])	During CA (n[%])	During CA ROSC (n[%])	During CA (n[%])
pH <7.35	18 [62]	29 [78]	19 [73]	10 [91]
pH >7.45	4 [14]	2 [5]	1 [4]	1 [1]
PaCO2 <35mmHg	9 [31]	7 [19]	6 [23]	1 [1]
PaCO2 >45mmHg	6 [21]	21 [57]	15 [58]	6 [55]
PaO2 <100mmHg	5 [17]	12 [32]	9 [34]	3 [27]
PaO2 100-200mmHg	28 [28]	8 [22]	6 [23]	2 [18]
PaO2 >200mmHg	16 [55]	17 [46]	11 [42]	6 [55]

## P050 Diagnostic strategies in intensive care unit cardiac arrest

### FO Holland^1^, S Entz^2^, S Lamprinaki^2^, M Abu-Tair^1^, R Borgstedt^3^, S Rehberg^3^, G Jansen^3^

#### ^1^Protestant Hospital of the Bethel Foundation, Department of Internal Medicine, Cardiology, Nephrology and Diabetology, Bielefeld, Germany; ^2^Protestant Hospital of the Bethel Foundation, Department of Internal Medicine and Gastroenterology, Bielefeld, Germany; ^3^Protestant Hospital of the Bethel Foundation, Department of Anaesthesiology, Intensive Care, Emergency Medicine, Transfusion Medicine, and Pain Therapy, Bielefeld, Germany

**Introduction:**

Cardiac arrest on ICU (ICU-CA) is usually monitored. Beside-diagnostics and trained healthcare professionals are immediately available. This offers exceptional opportunities and poses the question which diagnostic strategies are carried out in ICU-CA.

**Methods:**

Retrospective analysis of adult ICU-CA from 2016-2018 at a maximum care hospital in Germany with five ICU (two perioperative, two medical, one interdisciplinary) summarizing 71 ICU beds. ICU-CA was defined as requirement for chest compressions and/or defibrillation according to Utstein criteria. Diagnostics during ICU-CA were collected and analysed. Subgroup analyses included patients with and without ROSC. Primary endpoint was the performance rate of additional diagnostics during ICU-CA. Secondary endpoints were the type of diagnostics (i.e. blood gas analysis [BGA], echocardiography [EC] or chest-x-ray [XR]) and its therapeutic consequences.

**Results:**

There were 114 ICU-CA out of 14.264 ICU patients in total (incidence 79.9 per 10.000 ICU patients [CI95 65.3-94.5). All patients were monitored (electrocardiogram, pulsoxymetry, invasive blood pressure). During ICU-CA 50 (44%) patients received at least one additional diagnostic [i.e. BGA 41 (36%), EC 22 (19%) or XR 7 (6%)]. In 27 (54%) of these cases a therapeutic consequence was documented. These were application of HCO3- [n=12 (11%)], thoracic drainage [n=5 (4%)], transfusion or pericardiocentesis [each n=3 (3%)], lysis and bronchoscopy [each n=2 (2%)]. Comparing cases with and without ROSC, there were significant more diagnostics done in the group without ROSC but more therapeutic consequences seen in the ROSC-group (Table 1).

**Conclusions:**

ICU-CA is frequent. Diagnostics to detect reversible causes of CA were used rarely in ICU-CA (44%), even in patients without ROSC. Notably, diagnostics often had therapeutic consequences particularly in ROSC. Further studies are required to define standardized diagnostic algorithms during ICU-CA.

**Table 1 (abstract P050).** ICU-CA characteristics

	Overall	No-ROSC	ROSC	p-value
ICU-CA	114	37	77	-
Age [years]	72 ± 12	74 ± 9	72 ± 13	0.2
Male [n(%)]	78 (68)	25 (68)	53 (69)	0.89
Diagnostic done [n(%)]	50 (44)	25 (68)	25 (32)	0.0004
Therapeutic consequence [n(%)]	27 (54)	8 (32)	19 (76)	0.001

## P051 Continuous monitoring of cardiac patients on general ward were improved short term survival of in-hospital cardiac arrest

### UJ Go^1^, YJ Shin^1^, JM Lee^1^, HY Oh^1^, SB Hong^2^

#### ^1^Medical Alert Team, Asan Medical Center, Seoul, South Korea; ^2^Asan Medical Center, Department of Pulmonary and Critical care medicine, Seoul, South Korea

**Introduction:**

The importance of early detection in the in-hospital cardiac arrest (IHCA) is emphasized. Previous studies have reported that clinical outcomes are improved if IHCA is witnessed, or if a patient admitted to a monitored location [1, 2]. This study aimed to evaluate the association between continuous monitoring and survival of IHCA on general ward.

**Methods:**

A retrospective cohort study of IHCA in patients admitted to ward at an academic tertiary care hospital between January 2009 and December 2018 was performed. The primary outcome was return of spontaneous circulation (ROSC). The secondary outcomes were 72-hour survival and survival to hospital discharge.

**Results:**

Of the 996 enrolled patients, 54.7% patients had monitored. Among monitored group, 63.5% patients were pulse oximetry and electrocardiogram monitored. There were many witnessed events in the monitored group (93.8% vs. 57.4%, p<0.0001). By univariate analysis, there was difference of ROSC in the monitored group (73.8% vs. 66.7%, odds ratio [95% confidence interval] = 1.40 [1.07-1.84]; p=0.016). There were no significant difference 72-hour survival (1.00 [0.68-1.47]; p=0.993) and survival to hospital discharge (1.03 [0.77-1.36]; p=0.864). In subgroup multivariate analyses, the groups were divided according to illness category. In patients with cardiac illness, ROSC (83.0% vs, 63.5%, 2.86 [1.02-8.09]; p=0.047) and 72-hour survival (67.0% vs. 49.4%, 2.40 [1.04-5.51]; p =0.039) were higher in the monitored group (Table 1).

**Conclusions:**

Cardiac patients with continuous monitoring on general ward showed improving ROSC and 72-hour survival but not survival to hospital discharge in IHCA.

**References:**

1. Brady WJ et al. Resuscitation 82:845-852, 2011

2. Perman SM et al. J Am Heart Assoc 5:e003638, 2016

**Grant Acknowledgment:** This research was supported by a grant of the Korea Health Technology R&D Project through the Korea Health Industry Development Institute (KHIDI), funded by the Ministry of Health & Welfare, Republic of Korea (HI18C0599).

**Table 1 (abstract P051).** Association between monitored cardiac arrest and patient survival

Survival	Illness category	Monitored n(%)	Unadjusted OR (95% CI)	Adjusted OR (95% CI)
ROSC	Cardiac	73 (83.0)	2.79 (1.37-5.68)	2.86 (1.02-8.08)
ROSC	Noncardiac	329 (72.0)	1.24 (0.92-1.67)	0.68 (0.44-1.05)
72-hour survival	Cardiac	59 (67.0)	2.08 (1.13-3.85)	2.40 (1.04-5.51)
72-hour survival	Noncardiac	215 (47.0)	1.13 (0.86-1.49)	0.80 (0.52-1.23)
Survival to hospital discharge	Cardiac	35 (39.8)	1.50 (0.80-2.81)	1.36 (0.58-3.20)
Survival to hospital discharge	Noncardiac	109 (23.9)	0.95 (0.69-1.30)	0.69 (0.45-1.06)

## P052 Physiologic effects of steroids in in-hospital cardiac arrest (CORTICA study group1,2)

### E Pappa^1^, E Ischaki^1^, S Malachias^1^, A Giannopoulos^1^, K Vrettou^1^, G Karlis^1^, I Pantazopoulos^1^, D Makris^2^, S Zakynthinos^1^, S Mentzelopoulos^1^

#### ^1^Evaggelismos General Hospital, First Department of Intensive Care Medicine University of Athens Medical School, Athens, Greece; ^2^Larisa University Hospital, Department of Intensive Care Medicine, University of Thessaly Medical School, Larissa, Greece

**Introduction:**

In-hospital cardiac arrest is associated with poor outcomes. Although steroids are frequently used in patients with septic shock, it is unclear whether they are beneficial during cardiac arrest and after return of spontaneous circulation (ROSC).

**Methods:**

Of 369 cardiac arrest patients evaluated, 100 were enrolled. Advanced life support was conducted according to the2015 resuscitation guidelines. Forty-six patients were randomly assigned to receive methylprednisolone 40 mg during resuscitation, and 54 to receive saline (placebo). After resuscitation, steroid-treated patients received hydrocortisone 240 mg daily for up to 7 days, followed by tapering over the next 2 days. Primary outcomes were mean arterial blood pressure and central venous oxygen saturation (ScvO_2_) at 20 minutes, 4, 24, 48 and 72 hours after ROSC. Secondary end-points included left ventricular ejection fraction and eccentricity index at 12 and 72 hours, cardiac output and serum cytokine levels at 4, 24, 48 and 72 hours, cerebral blood flow index at 4 and 72 hours after ROSC, organ failure–free days from follow-up day 1 through 60,neurological status at dischargeand steroid-associated complications.

**Results:**

Post-resuscitation MAP did not differ significantly between steroid- and placebo-group at any time point (20 min: 85.2±21.3 vs. 84.7±21.4; 4h: 83.9±18.1 vs. 78.9±15.9; 24h: 79.9±16.0 vs. 81.9±15.2; 48h: 80.2±10.0 vs. 84.2±13.6; 72h: 85.2±11.6 vs. 84.7±14.5 (p>0.05 for all comparisons). There was no significant difference between the two groups in ScvO_2_ andall the secondary outcomes (p>0.05 for all comparisons).

**Conclusions:**

The present study found no significant physiologic benefit of corticosteroid administration during and after resuscitation in hospitalized patients with cardiac arrest.

## P053 The experiences of EMS providers taking part in a large randomized trial of airway management during out of hospital cardiac arrest, and the impact on their views and practice. Results of a survey and telephone interviews

### M Thomas^1^, M Robinson^2^, K Kirby^3^, J Brandling^3^, S Voss^3^, J Benger^3^

#### ^1^Bristol Royal Infirmary, Intensive Care Unit, Bristol, United Kingdom; ^2^South West Ambulance Service, Exeter, United Kingdom; ^3^University of West of England, Bristol, United Kingdom

**Introduction:**

The aim is to explore EMS experiences of participating in a large trial of airway management during out-of-hospital cardiac arrest (AIRWAYS-2), specifically to explore:

1. Any changes in views and practice as a result of trial participation.
2. Experiences of trial training.
3. Experiences of enrolling critically unwell patients without consent.
4. Barriers and facilitators for out-of-hospital trial participation.

**Methods:**

An online questionnaire was distributed to 1523 EMS providers who participated in the trial. In-depth telephone interviews explored the responses to the online questionnaire. Quantitative data were collated and presented using simple descriptive statistics. Qualitative data collected during the online survey were analysed using content analysis. An Interpretive Phenomenological Analysis approach was used for analysis of qualitative interview data

**Results:**

Responses to the online questionnaire were received from 33% of AIRWAYS-2 study paramedics and 19 study paramedics were interviewed. Paramedics described barriers and facilitators to trial participation and changes in their views and practice. The results are presented in five distinct themes: research process; changes in views and practice regardingairway management; engagement with research; professional identity; professional competence.

**Conclusions:**

Participation in the AIRWAYS-2 trial was enjoyable and EMS providers valued the training and study support. There was enhanced confidence in airway management as a result of taking part in the trial. Study paramedics expressed preference for the method of airway management to which they had been randomized. There was support for the stepwise approach to airway management, but also concern regarding the potential to lose tracheal intubation from ‘standard’ paramedic practice.

## P054 Causes of medical care-associated cardiac arrest on the intensive care unit

### S Entz^1^, FO Holland^2^, S Lamprinaki^1^, M Abu Tair^2^, M Krüger^1^, R Borgstedt^3^, S Rehberg^3^, G Jansen^3^

#### ^1^Protestant Hospital of the Bethel Foundation (EvKB), Department of Internal Medicine and Gastroenterology, Bielefeld, Germany; ^2^Protestant Hospital of the Bethel Foundation (EvKB), Department of Internal Medicine, Cardiology and Nephrology, Bielefeld, Germany; ^3^Protestant Hospital of the Bethel Foundation (EvKB), Department of Anaesthesiology, Intensive Care, Emergency Medicine, Transfusion Medicine and PainTherapy, Bielefeld, Germany

**Introduction:**

Cardiac arrest on intensive care unit (ICUCA) following therapeutic interventions is of imminent importance, because the interventions are comparatively predictable and precautions can potentially be taken. This study investigates medical care associated complications that led to ICUCA.

**Methods:**

Intensive care database was screened for patients ≥18 years who experienced ICUCA in a tertiary hospital with five ICU (two medical, two surgical, one interdisciplinary, with a sum of 71 ICU beds) in Germany from 2016-2018. ICUCA was defined as receiving chest compression and/or defibrillation *after* admission on ICU and classified as “medical care associated” if it was preceded by a therapeutic intervention (i.e. induced by medication, bedding procedures, iatrogenic injuries, procedure associated). Subgroups included patients with recurrence of spontaneous circulation (ROSC) vs. no-ROSC and patients with vs. without vasopressor therapy before intervention.

**Results:**

There were 125 ICUCA in 114 patients of totally 14,264 ICU patients. Medical care associated complications leading to ICUCA were detected in 28 cases (22%) [Incidence 19.6/10,000(*CI95 12.3 -26.9)].* ICUCA following therapeutic interventions occurred because of circulatory insufficiency [n=20(70%)], respiratory failure [n=5(17%)] and airway associated problems [n=3(10%)]. Nine of the 28 patients (32%) with care-associated ICUCA died. Table 1 demonstrates therapeutic interventions followed by ICUCA.

**Conclusions:**

Care-associated complications were common reasons for ICUCA. Most of events were induced by circulatory insufficiency due to induction of anaesthesia and bedding procedures. Further investigations should focus on preventive strategies, such as vasopressor infusion before therapeutic interventions.

**Table 1 (abstract P054).** Medical care associated causes of ICUCA

	Total [n(%)]	ROSC [n(%)]	no ROSC [n(%)]	No vasopressors before ICUCA [n(%)]
induction of anaesthesia	11(39)	8(73)	3(27)	8(72)
bedding procedures	7(25)	4(57)	3(43)	3(43)
other medication	4(14)	3(75)	1(25)	1(25)
injuries (pneumo-/hematothorax, tracheotomy injury)	3(11)	2(67)	1(3)	3(100)
switching syringes for continuous infusions	2(7)	2(100)	0(0)	0(0)
no pacemaker in AV-block III	1(4)	0(0)	1(100)	1(100)
	28(100)	19(68)	9(32)	16(57)

## P055

**Withdrawn**

## P056 Favorable effects of reducing low-flow duration in ECPR patients

### A Higashi^1^, TA Nakada^1^, T Imaeda^1^, R Abe^1^, K Shinozaki^2^, S Oda^1^

#### ^1^Chiba University Graduate School of Medicine, Department of Emergency and Critical Care Medicine, Chuo, Chiba, Japan; ^2^The Feinstein Institute for Medical Research, The Feinstein Institute for Medical Research, Manhasset, United States

**Introduction:**

The low-flow duration (LFD) in extracorporeal cardiopulmonary resuscitation (ECPR) is considered to be related to the neurological outcome. Nevertheless, there are a few reports on how the LFD and the prognosis had changed over time with continuous efforts. In this investigation, we aimed to clarify the temporal change in the LFD and outcomes of patients with ECPR at a single center between January 2003 and December 2017.

**Methods:**

This retrospective observational study was performed at Chiba University Hospital, a tertiary-care hospital in Japan. We reviewed medical records of all patients who received ECPR from January 2003 to December 2017. Over time changes in LFD were investigated, and then, the association of LFD with a 90-day outcome was analyzed.

**Results:**

Records from 175 patients were utilized in this study. Of them, 117 patients were in-hospital cardiac arrest (IHCA), and 58 patients were out-of-hospital cardiac arrest (OHCA). We found the significant shortening of LFD during the study period (IHCA, slope -5.39 [min/3-year], P<0.0001; OHCA, slope -5.49 [min/3-year], P=0.0084). In the multivariable regression, the shorter LFD was significantly associated with better 90-day survival and a favorable neurological outcome only in the IHCA group (LFD per minute, 90-day favorable neurological outcome: odds ratio=1.03, 95% confidence interval=1.00-1.06, P=0.046).

**Conclusions:**

The LFD was significantly shortened over time in both groups of IHCA and OHCA. The shorter LFD was associated with a better 90-day outcome.

## P057 Clinical features and outcomes of in-hospital cardiac arrest in Code Blue events: a retrospective observational study

### M Akatsuka, H Tatsumi, H Kuroda, S Kazuma, W Aisaka, S Suzuki, K Kikuchi, Y Goto, M Kodama, Y Masuda

#### Sapporo Medical University School of Medicine, Department of Intensive Care Medicine, Sapporo, Japan

**Introduction:**

In-hospital cardiac arrest (IHCA) is a lethal event. However, IHCA has received less attention than out-of-hospital cardiac arrest (OHCA). There have been some studies on IHCA; however, there is a lack of information on the evidence and clinical features of IHCA compared with information for OHCA. We therefore conducted this study to clarify important aspects of the epidemiology and prognosis of IHCA in patients with code blue activation.

**Methods:**

We carried out a retrospective observational study of patients with code blue events in our hospital during the period from January 2010 to October 2019. We obtained information on the characteristics of patients including age and gender, IHCA characteristics including the time of cardiac arrest, event being witnessed, presence of bystander cardiopulmonary resuscitation (CPR), initial shockable rhythm, vital signs 1 h or 6 h before cardiac arrest, survival to hospital discharge (SHD), and the cardiac arrest survival postresuscitation in-hospital (CASPRI) score. The primary endpoint was SHD. We performed univariate and multivariate logistic regression analyses.

**Results:**

A total of 293 code blue events were activated during the study period. Finally, 81 patients were included in this study. Overall, the SHD rate was 28.4%. The median time of CPR was 14 min (interquartile range, 6-28 min). The rate of initial shockable rhythm was 19.8%. There were significant differences in CPR duration, shockable rhythm, and CASPRI score between the SHD group and non-SHD group by univariate-logistic regression analysis. CASPRI score was found to be the most effective predictive factor for SHD (OR=0.98, p=0.006) by multivariate-logistic regression analysis.

**Conclusions:**

Our results demonstrated that CASPRI score is associated with SHD in CPA patients with in-hospital code blue events. CASPRI score in IHCA patients would be a simple and useful adjunctive tool for management of post-cardiac arrest syndrome (PCAS).

## P058 Peri-operative cardiac arrest in prematurity – incidence and causes at a tertiary care hospital between 2008-2018

### G Jansen, J Popp, E Lang, R Borgstedt, B Schmidt, S Rehberg

#### Protestand Hospital of the Bethel Foundation, Anaesthesiology, Intensive Care and Emergency Medicine, Bielefeld, Germany

**Introduction:**

The peri-operative care of premature pediatric patients requires special expertise and is therefore reserved for specialized centers. Although premature birth is described as a risk factor for peri-operative complications and cardiac arrest (POCA) there are no data on its incidence and causality in this particular population [1]. The present study investigates the incidence and causality of pediatric POCA at a tertiary care hospital and level I perinatal center in Germany.

**Methods:**

In the anesthesia database of the study center, all anaesthesiological procedures in patients <16 years of age were examined for POCA in preterm infants (gestational age <40th week of gestational age) between 2008 and 2018. The peri-operative period was defined between the beginning of anesthesiological care up to 60 minutes after anesthesia and/or sedation. We defined cardiac arrest as the necessity of chest compressions. The perioperative phase and the cause of the POCA, gestational age and birth weight were recorded.

**Results:**

Between 2008 and 2018, 308 (1.3%) of the 22,650 pediatric anesthesiological procedures were performed on 301 premature infants. In total, 10 POCA occurred in 9 of these patients (f=6, M=3; average gestional age 208±27 days; average birth weight 1510±747g (incidence 3.2%, CI 96 1.3-5.2%). The time of occurrence and the causes of POCA are shown in Table 1.

**Conclusions:**

POCA in premature babies is rare and has an incidence of 1.3%, which is significantly higher than the non-premature babies. The main causes are problems or complications associated with the respiratory tract and its management, as well as massive hemorrhage.

**References:**

1. Habre W et al. Lancet Respir Med 5:412-25, 2017

**Table 1 (abstract P058).** Perioperative phase and causes of peri-operative cardiac arrest

Peri-operative Phase	n	Cause	n
Induction	1	Difficult airway	1
Maintenance	8	Hemorrhagic shock	4
		Mucus obstructed endotracheal tube	2
		Dislocated endotracheal tube	1
		Septic Shock	1
Awakening	1	Hypoxaemic attack in case of fallot-pentalogy	1

## P059 Peri-operative pediatric cardiac arrest – incidence and mortality in 22,650 pediatric anesthetics at a tertiary care hospital between 2008-2018

### G Jansen^1^, J Popp^2^, E Lang^1^, R Borgstedt^1^, B Schmidt^1^, S Rehberg^1^

#### ^1^Protestand Hospital of the Bethel Foundation, Anaesthesiology, Intensive Care and Emergency Medicine, Bielefeld, Germany; ^2^Protestand Hospital of the Bethel Foundation, Bielefeld, Germany

**Introduction:**

Peri-operative cardiac arrest (POCA) in children's anesthesia care is a dreaded event. Depending on the country and population, studies describe incidences between 2.9-20.6 per 10,000 children's anesthetics. There are no data on the current incidence of pediatric POCA in Germany. The present study investigates the incidence of POCA at a tertiary hospital and level I perinatal center in Germany.

**Methods:**

In the anesthesia database of the study center, all anaesthesiological procedures in patients <16 years were examined for POCA. The peri-operative period was defined between the beginning of anesthesia care up to 60 minutes after anesthesia or sedation. Cardiac arrest was defined as the necessity of chest compressions. Age, weight, ASA status, cause of death and survival after 30 days were recorded.

**Results:**

18 POCA (median weight was 2525g [Q1 7151;Q75(14748)]) were observed in 22,650 anaesthesiological procedures (incidence 7.9±4.2 per 10,000 [CI95 4.3-11.6]). Table 1 shows the distribution of the individual age groups, incidences and mortalities of POCA. Peri-operative 30-day mortality was 3 per 10,000 [CI 95 1-5]. Three children died intraoperatively as a result of hemorrhagic shock, one on the PICU as a result of malignant hyperthermia. 30 days after POCA, 4 more children had died on the ICU due to their underlying disease.

**Conclusions:**

POCA is a rare event. Risk factors are an age <28 days and an ASA status ≥ III. The main cause of peri-operative death in patients <16 years of age is massive hemorrhage, the 30-day mortality is determined by the underlying disease.

**Table 1 (abstract P059).** Distribution of age groups

Age	n	Anesthesias	Incidence (per 10.000)	Mortality (per 10.000)
< 28 days	10	642	155.8	62.3
28 days-<1 year	5	2922	17.1	6.8
1- ≤5 years	2	8096	2.5	1.3
6- ≤12 years	1	7882	1.3	1.2
13-≤15 years	´0	3108	-	-

## P060 In-hospital cardiac arrest - predicting adverse outcomes

### T Partington, J Borkowski, J Gross

#### Northwick Park Hospital, Anaesthesia/Critical Care, London, United Kingdom

**Introduction:**

Cardiac arrest occurs in 1.2 per 1000 hospital admissions in the UK. Return of spontaneous circulation (ROSC) is achieved in approximately half of resuscitation attempts, but rate of survival to hospital discharge is substantially lower [1]. In our centre, post-arrest care accounts for 6.4% of ICU admissions. Premorbid social function is purported to affect outcomes, but comorbidity scores are more often used for risk stratification. Using a novel Social Function Score alongside an existing comorbidity scale, we aimed to identify trends to inform management of patients at risk of deterioration.

**Methods:**

A six-month prospective observational study was conducted in a major UK hospital from October 2017 to April 2018. For all adult inpatient cardiac arrests, medical notes were reviewed and data collected on the following domains:

Patient demographics

- Comorbidities and functional status
- Admission details
- Post-arrest events

Statistical analysis was performed using Student’s unpaired t-test.

**Results:**

54 cardiac arrests occurred. 85% were in medical patients, with the majority male (63%) and aged over 75 (63%). 89% were emergency admissions, with mean duration of hospital stay pre-arrest 9 days. In 17 cases (31%) sustained ROSC was achieved. However, seven of these (41%) were not subsequently admitted to the ICU. Only six patients (11%) survived to hospital discharge. Pre-admission function and comorbidity were worse in patients who did not survive to discharge (Fig. 1), but these were not statistically significant in view of small survivor group size.

**Conclusions:**

In an increasingly frail inpatient population, a substantial proportion of patients in whom circulation is restored after cardiac arrest are subsequently considered unsuitable for ICU admission. Given our understanding of inferior outcomes in patients with poor physiological reserve, we encourage early discussion regarding the appropriateness of CPR in selected patients, guided by social function and comorbidity.

**References:**

1. National Cardiac Arrest Audit 2017/18

**Fig. 1 (abstract P060).**
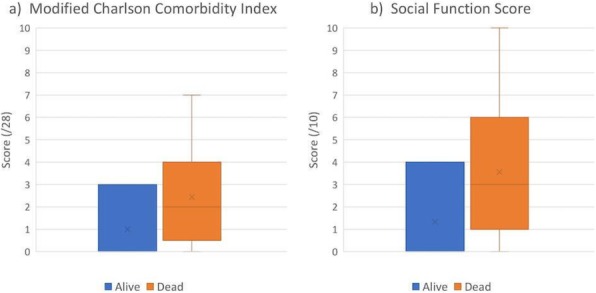
Box and whisker plots displaying a) Modified Charlson Comorbidity Index and b) Social Function Score at time of admission, in patients alive at hospital discharge (blue) vs. dead (orange)

## P061 Investigation of factors determining mortality in out-of-hospital cardiac arrest (OCHA)

### C Balci^1^, O Kucuk^2^, E Haftaci^3^, E Karaca^4^, S Ozbay^4^, H Karaca^4^

#### ^1^Kutahya Healty Science Un, Anaesthesiology and Reanimation, Kutahya, Turkey; ^2^Balıkesir City Hospital, ICU, Balıkesir, Turkey; ^3^Karaman Goverment Hospital, Karaman, Turkey; ^4^Derince Traning Hospital, Anaesthesiology and Reanimation, Kocaeli, Turkey

**Introduction:**

There are studies that determine events related to poor outcome in cardiac arrest [1].  In our study, following parametres were determined OHCA patients; age median years, Asian/Europe/Syrian, Bystander CPR, Bystander AED, EMS defibrillation, initial cardiac rhythm, prehospital ROSC, corneal and pupillary light reflex and day survival. We determineted poor prognostic sign with post-cardiac arrest patients. In this study, we identified the causes of poor outcome in patients with OHCA.

**Methods:**

This was a single-centre, retrospective study.  We determined incidence and epidemiological factors including: demographics, initial cardiac rhythm. Our study population were non-traumatic OHCA. Our ICU, All OHCA patient were evaluated wtih ECHO, and fluid, inotrope and vazopressor were added according to cardiac performance.

**Results:**

During our study, 5970 patients who were admitted to intensive care unit between 2012-2019 were screened. 133 of these patients were out-of-hospital arrest and 41 of them were in-hospital arrest. Development of cerebral oedema during treatment in hospital remains a poor prognostic sign. The evaluation of initial cardiac ritm is useful to predict neurological outcome in post-cardiac arrest patients.

**Conclusions:**

Survival after OHCA remains low. The evaluation of initial cardiac ritm is useful to predict mortality and neurological outcome in post-cardiac arrest patients.

**References:**

1. Ørbo M et al. Resuscitation 85 :1462-1468, 2014

## P062 Time factors, basic life support performance and outcomes of out-of-hospital cardiac arrests detected by schoolchildren

### H Inaba^1^, K Takada^2^, H Kurosaki^2^, Y Wato^3^, A Yamashita^2^

#### ^1^ Kanazawa University Graduate School of Medicine, Department of Circulatory Emergency and Emergency Medical Science, Kanazawa, Japan; ^2^Kanazawa University Graduate School of Medicine, Kanazawa, Japan; ^3^Kanazawa Medical University, Uchinada, Japan

**Introduction:**

Basic life support (BLS) education and training for school children is active in Japan. However, the BLS action by schoolchildren may be limited by school rules. This study aimed to analyse the time factors for basic life support performance and outcome in classmate-witnessed out-of-hospital cardiac arrest (OHCA) and to investigate how schoolchildren act when they detect OHCA.

**Methods:**

Nation-wide database for 1,068 school children cases with OHCA and local extended database for 5,478 EMS-unwitnessed OHCA, both of which were prospectively collected during the period of 2011–2016, were retrospectively analysed.

**Results:**

Proportion of schoolchildren-detected OHCA was low in classmate cases (16.8%, 179/889) in nationwide database and extremely low in all EMS-unwitnessed OHCAs (1.6%, 88/5,478) in local database. Nationwide database analyses revealed that both emergency call and bystander CPR were delayed when a classmate witnessed the OHCA case: median, 1 vs. 0 min and 3 vs. 2 min, respectively. Classmate-witnessed cases were associated with higher incidences of shockable initial rhythm, AED use and traumatic causes. The rate of neurologically favourable outcome was 19.6% and 12.3%, respectively in classmate-witnessed and other cases: adjusted OR; 99% CI, 1.24; 0.63–2.47. Of 88 cases detected by schoolchildren in our prefecture, 8 (34%) cases had presumed cardiac aertiology and 12(13.8%) cases were caused by suicide attempts (hanging and fall). School children placed emergency 119 calls as the first action only in 32 (36.4%) cases. Emergency calls were largely delayed when school children dialled other numbers or left the scene to seek adult help. School children were rarely involved in bystander CPR (21%) and AED placement (1%).

**Conclusions:**

School children are rarely involved in entire BLS. Emergency calls and bystander CPR are delayed when schoolchildren act to seek help. Because schoolchildren detect suicide-related OHCAs, psychological care to schoolchildren involved in BLS may be necessary.

## P063 Prognostic value of neutrophil/lymphocyte and platelet/lymphocyte predicting cardiopulmonary resuscitation with spontaneous circulation recovery

### C Li

#### the Affiliated Suzhou Hospital of Nanjing Medical University, Suzhou, China

**Introduction:**

To investigate the predictive value of peripheral blood neutrophil-to-lymphocyte ratio (NLR) and platelet-to-lymphocyte ratio (PLR) on in-hospital mortality in patients with spontaneous circulation recovery after cardiac arrest.

**Methods:**

A retrospective analysis was made of 30 patients who recovered from cardiac arrest in our hospital from April 2012 to November 2018 and were admitted to the intensive care unit for more than 24 hours. They were divided into survival group and death group according to the outcome of discharge.The dynamic changes and differences of NLR and PLR in 24 hours and 48-72 hours after admission to ICU between the two groups were analyzed and compared. Multivariate analysis and ROC curve were used to explore the predictive value of NLR and PLR for in-patient mortality.

**Results:**

Compared with the survival group, PLR in the dead group was significantly lower within 24 hours of admission to the intensive care department (P < 0.05), while NLR in 48-72 hours was significantly higher (P < 0.05). The NLR of surviving group was significantly lower than that of 24 hours (P < 0.05), while the NLR and PLR of death group were not significantly different (P < 0.05) from that of 24 hours (P < 0.05). Multivariate logistic regression analysis and ROC curve showed that NLR of 48-72 h in ICU was an independent risk factor for predicting in-patient mortality, and had high sensitivity and specificity in predicting death outcomes.

**Conclusions:**

Neutrophil to lymphocyte ratio, platelet to lymphocyte ratio can help to judge the outcome of patients with cardiac arrest and recovery of autonomic circulation after cardiopulmonary resuscitation.

## P064 Neurofilament light chain as an outcome predictor after cardiac arrest

### L Wihersaari^1^, N Ashton^2^, M Reinikainen^1^, P Jakkula^3^, V Pettilä^3^, J Hästbacka^3^, M Tiainen^3^, K Blennow^2^, H Zetterberg^2^, MB Skrifvars^3^

#### ^1^University of Eastern Finland and Kuopio University Hospital, Department of Anaesthesiology and Intensive Care, Kuopio, Finland; ^2^University of Gothenburg and Sahlgrenska University Hospital, Gothenburg, Sweden; ^3^University of Helsinki and Helsinki University Hospital, Helsinki, Finland

**Introduction:**

High concentrations of neurofilament light chain (NFL) in serum predicted poor outcome in patients resuscitated from cardiac arrest [1]. We aimed to evaluate the prognostic value of NFL in COMACARE trial [2,3] patients who were resuscitated from cardiogenic out-of-hospital cardiac arrest (OHCA) with a shockable initial rhythm.

**Methods:**

Using the commercially available Single Molecule Array (Simoa) NF-light immunoassay (Quanterix, Lexington, MA), we measured NFL levels from plasma taken 48 h after OHCA. We assessed neurological outcome six months after OHCA and defined good outcome as Cerebral Performance Category (CPC) 1-2 and poor outcome as CPC 3-5.

**Results:**

Overall, six-month outcome was good for 73 of 112 (65.2%) patients. The median (interquartile range) NFL concentration at 48 h was 19 pg/ml (11-31) in the patients with good outcome and 2343 pg/ml (587-5829) in those with poor outcome, p<0.001. NFL at 48 h predicted poor outcome with an area under the receiver operating characteristic curve (ROC) of 0.98 (95% CI, 0.97-1.00). With the cutoff 263 pg/ml, the sensitivity was 0.83 (95% CI, 0.71-0.96), specificity was 0.99 (95% CI, 0.96-1.00), and the positive likelihood ratio was 60.8 (95% CI, 8.6-428.4)

**Conclusions:**

In patients resuscitated from OHCA with a shockable rhythm, the plasma NFL concentration at 48 h after cardiac arrest had an excellent ability to predict 6-month outcome.

**References:**

1. Moseby-Knappe M et al. JAMA Neurol 76:64-71, 2019.

2. Jakkula P et al. Intensive Care Med 44:2091-2101, 2018.

3. Jakkula P et al. Intensive Care Med 44:2112-2121, 2018.

## P065 Changes in iron metabolism and prognosis of patients with out-of-hospital cardiac arrest

### P Aries^1^, O Huet^1^, O Tocquer^1^, M Consigny^2^, T Lefebvre^3^, M Padelli^4^, E Lher^5^, JM Tonnelier^5^, C Aubron^5^

#### ^1^Brest Teaching Hospital, Departement of Anaesthesia and Surgical Intensive Care, Brest, France; ^2^Brest Teaching Hospital, Centre d’Investigation Clinique CIC INSERM 1412, CHRU Brest, Brest, France; ^3^Hôpital Louis Mourier HUPNVS, Biochimie/Centre Français des Porphyries, Colombes, France; ^4^Brest Teaching Hospital, Département de Biochimie et Pharmaco-toxicologie, Brest, France, ^5^Brest Teaching Hospital, Department of Medical Intensive Care Unit, Brest, France

**Introduction:**

Out-of-hospital cardiac arrest (OHCA) remains associated with a very high mortality and poor long-term prognosis.  Ischemia reperfusion through lesions due to oxidative stress is responsible for cell damages and free iron may play a key role in these phenomenons. Little is known about magnitude of changes in iron metabolism parameters after cardiac arrest and its association to patient’s prognosis.

**Methods:**

This is a single centre cohort study.  Adults admitted for OHCA and receiving targeted temperature management were eligible. Free iron, transferrin saturation coefficient, ferritin, hepcidin and soluble transferrin receptor were measured in plasma at ICU admission (H0), at 24 hours (H24) and 48 hours (H48). SOFA score changes were calculated between H0, H24 and H48. Demographic data and neurological status at 6 months with Cerebral Performance Category score (CPC score) were also assessed. (NCT number:NCT03491670)

**Results:**

Between May 2018 and March 2019,31 patients were hospitalized for OHCA and 25 patients have been included. The reasons for cardiac arrest were asystole in 13 patients (52%) and shockable rhythm in 10 patients (40%). Hospital mortality was 68% (17 patients) and 19 patients had a poor neurological outcome (CPC score 3-5 or death) at 6 months. Mean SOFA score at H0 was 11.40 ± 3.14. Mean free iron level at ICU admission was 22.46± 12.41 μmol/l and significantly decreased between H0 and H48 (mean: 22.46 ± 12.41 μmol/l vs 5.27±6.10μmol/l; p = 0.0003). At H48, patients with SOFA score > 12 (vs SOFA score ≤ 12) had a higher free iron level (35.5 μmol/l vs 16 μmol/l, p = 0.0333) (Figure 1). We found a positive correlation between free iron level at H0 and changes of SOFA score between H0 and H48 (r= 0.56 IC95[0.08;0.76]).

**Conclusions:**

Out-of-hospital cardiac arrest is associated with a significant change of plasma free iron level. Free iron level at admission is associated with short term outcome. Further research is warranted to better determine the significance of such changes.

**Fig. 1 (abstract P065).**
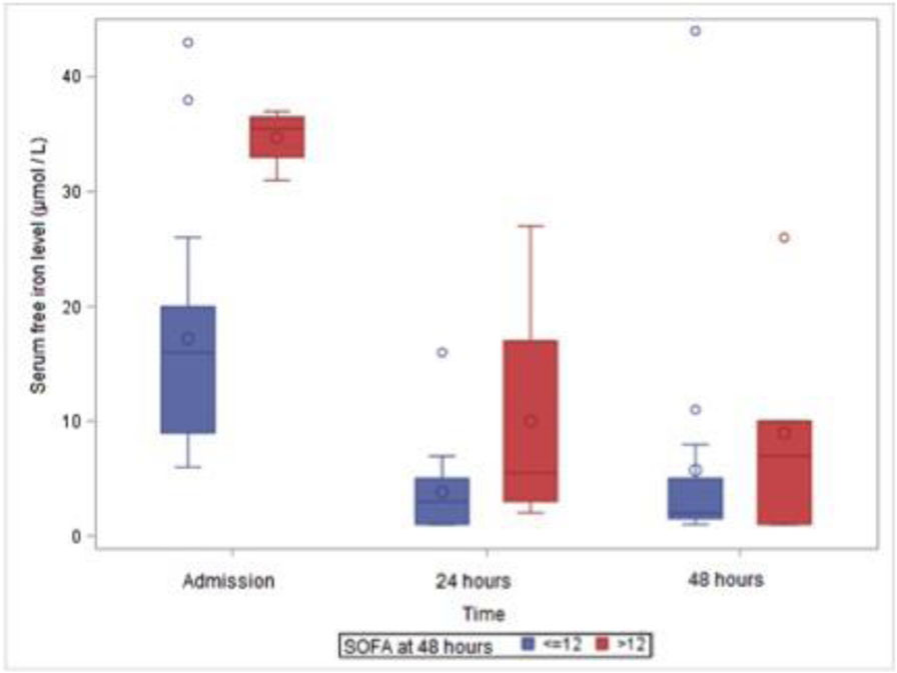
Comparison of serum free iron level at admission, 24 hours and 48 hours according to SOFA score at 48 hours

## P066 Association between early hyperoxia after out of hospital cardiac arrest with return of spontaneous circulation and 30-day survival

### A Awad^1^, J Olsson^2^, P Nordberg^1^, M Jonsson^3^, M Ringh^1^, J Hollenberg^3^, E Joelsson-Alm^2^

#### ^1^Karolinska Institute, Solna, Sweden, Department of Medicine, Center for Resuscitation Science, Solna, Stockholm, Sweden; ^2^Karolinska Institute, Solna, Sweden, Department of Clinical Science and Education, Södersjukhuset, Karolinska Institutet, Stockholm, Sweden, Solna, Stockholm, Sweden; ^3^Karolinska Institute, Solna, Sweden, Solna, Stockholm, Sweden

**Introduction:**

The optimal level of arterial oxygen in the post-resuscitation period is unknown. Recent studies show conflicting results in regard to hyperoxia and its association with survival after out-of-hospital cardiac arrest (OHCA) [1]. The aim of this trial is to study the association between early hyperoxia after OHCA with return of spontaneous circulation (ROSC) and 30-day survival.

**Methods:**

Observational study using data from three Swedish national registers (i.e. intensive care, cardiac arrest and national patient registries). Resuscitated OHCA patients, who are unconscious (Glascow Coma Scale ≤8) and admitted to an ICU between January 2010-March 2016 were included. Patients were divided into 4 groups based on lowest PaO_2_ within 1 hour from ICU arrival. *Hypoxia* was defined as PaO_2_<60 mmHg, *normoxia* as PaO_2_ 60-100 mmHg, *hyperoxia* as PaO_2_ 100-400 mmHg and *severe hyperoxia* as PaO_2_ > 400 mmHg. Multivariate logistic regression was performed. The primary endpoint was 30-day survival.

**Results:**

In total, 3496 patients were included in the analysis of which 284 patients had hypoxia, 1490 had normoxia, 1448 had hyperoxia and 274 had hyperoxia. The crude 30-day survival rates were 27.1% in the hypoxia group, 38.1% in the normoxia group, 34.9% in the hyperoxia group and 25.9% in the severe hyperoxia group (Figure 1). The hyperoxia group had significantly lower 30-day survival compared to the normoxia group, adjusted odds ratio 0.62 (95% confidence interval 0.44-0.89, p=0.01). The difference in survival between the other groups compared to the normoxia group did not meet statistical significance.

**Conclusions:**

In this Swedish observational cohort study of resuscitated OHCA patients, early severe hyperoxia, compared to normoxia, was associated with significantly lower 30-day survival. This is in accordance with several previous reports and warrant further studies.

**References:**

1. Wang CH et al. Resuscitation 85:1142-1148, 2014

**Fig. 1 (abstract P066).**
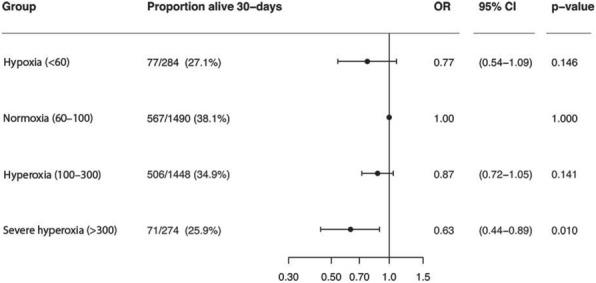
Comparison in 30-day survival between different groups according to lowest PaO2 (mmHg) within 1 hour from ICU arrival. Adjusted odds ratios (OR) are used and the normoxia group is used as a reference group

## P067 Correlation between increased inflammation and early-onset pneumonia in patients with out-of-hospital cardiac arrest treated with extracorporeal cardiopulmonary resuscitation

### D Shiba^1^, T Hifumi^2^, N Otani^2^, S Ishimatsu^2^

#### ^1^St. Luke´s International Hospital, Emergency Department, Tokyo, Japan; ^2^St. Luke´s International Hospital, Tokyo, Japan

**Introduction:**

After a successful resuscitation, a systemic inflammatory response occurs, and the C-reactive protein (CRP) level represents the degree of inflammation [1-3]. This study examined the association between increased inflammation and early-onset pneumonia (EOP) in patients treated with extracorporeal cardiopulmonary resuscitation (ECPR) after out-of-hospital cardiac arrest (OHCA).

**Methods:**

This retrospective study included data of patients with OHCA treated with ECPR admitted to St. Luke’s International Hospital between April 2006 and April 2019. The exclusion criteria were as follows: age < 18 years, therapeutic hypothermia withdrawal due to death or circulatory failure, or sepsis as a suspected cause of cardiac arrest. Patients were diagnosed with EOP according to clinical signs and symptoms acquired after a hospitalization period of >48 h and within 7 days of admission. The CRP levels were measured daily from admission to day 3.

**Results:**

We studied 55 patients with a median age of 55 years (interquartile range: 42-65 years). Furthermore, 52 (95%) patients were males, and the median time interval from collapse to adequate flow was 51 (42-63) min. All patients received prophylactic antibiotics, and 18 (33%) of them had favorable neurological outcomes (CPC, 1-2). EOP occurred in 32 (58%) patients, with a significantly higher CRP level on day 3 than that in those without EOP (13.9 [11.2-19.0] mg/dL vs. 10.9 [7.3-13.6] mg/dL, p = 0.006). Multivariable analysis revealed that the CRP level on day 3 (odds ratio, 1.28; 95% confidence interval, 1.06-1.56; p = 0.002) was significantly associated with EOP development.

**Conclusions:**

Increased inflammation correlated with EOP development in patients with OHCA treated with ECPR.

**References:**

1. Francois B et al. N Engl J Med 381:1831-1842, 2019

2. Dell'anna AM et al. Resuscitation 85:932-938, 2014

3. Shiba D et al. Circ Rep 1: 575–581, 2019

## P068 Mode of death after cannulation for venoarterial extracorporeal membrane oxygenation, insights from a single center registry

### V Zotzmann^1^, J Rilinger^2^, CN Lang^2^, M Jäckel^2^, T Wengenmayer^2^, C Bode ^2^, D Staudacher^3^

#### ^1^University of Freiburg, Heart Center, Freiburg, Germany; ^2^University of Freiburg, Freiburg, Germany; ^3^University of Freiburg, University of Freiburg, Freiburg, Germany

**Introduction:**

Categorizing reasons for death after eCPR is important for comparing outcomes to other studies, assessing benefits of interventions, and better define this heterogeneous patient collective. A categorizing for death after cardiac arrest in both in-hospital (IHCA) and out-of-hospital (OHCA) arrests has been proposed in non-eCPR patients by Witten et al. Here, we adopt this categorization to eCPR patients.

**Methods:**

Single-center, retrospective, cohort study of patients without ROSC after IHCA or OHCA and eCPR between 2010 and 2017. Patients with survival below 24 hours were excluded. Patients were allocated to one of five predefined reasons for death.

**Results:**

231 va-ECMO patients were included (age 58.6±14.3, 29.9% female, 58% eCPR, 30 day survival 42.9%). Reasons for death for patients with va-ECMO for shock (survival 53%) and eCPR (36%) were: neurological withdrawal of care (10% vs 25%), comorbid withdrawal of care (18% vs 4%), refractory hemodynamic shock (16% vs 33%), respiratory failure (3% vs 2%), and withdrawal due to presumed patient will (0% vs 1%) (Figure 1). The differences in reasons for death among the two groups were significant (p <0.001), driven by withdrawal due to neuroprognostication, comorbidity and hemodynamic instability.

**Conclusions:**

Categorizing death after va-ECMO into five categories is feasible. There are significant difference between patients with va-ECMO for shock and eCPR. Interestingly, only a quarter of patients after eCPR died due to brain damage.

**Fig. 1:**
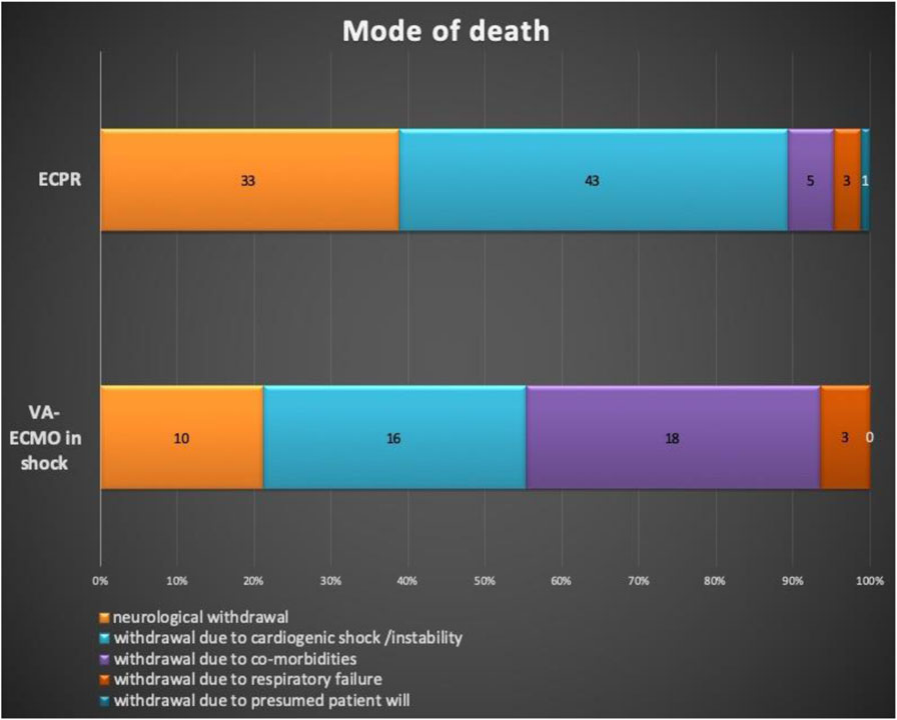
**(abstract P068).** Mode of death in VA-ECMO patients and patients after ECPR

## P069 ECMO mobile team for donor cardiac death. a pilot ECMO-TT study

### R Badenes^1^, C Álvarez^2^, F Hornero^3^, J Guijarro^3^, JM Segura^3^, R Zaragoza^2^

#### ^1^Hospital Clinic Universitari de Valencia, Department of Anesthesiology and Surgical-Trauma Intensive Care, Valencia, Spain; ^2^Hospital Dr. Peset, Valencia, Spain; ^3^Hospital Clinic Universitari de Valencia, Valencia, Spain

**Introduction:**

Scarcity of potential dead brain donors and the persistent mismatch between supply and demand of organs for transplantation has led the transplant community to reconsider donation after circulatory death (DCD) as a strategy to increase the donor pool. Normothermic regional perfusion (nRP) by extracorporeal membrane oxygenation (ECMO) may be the most effective method for preserving abdominal organs in DCD, especially in liver transplantation [1, 2]. A pitfall of this method is its complexity and the unavailability of this resource in some hospitals, especially in regional hospitals, where potential DCD donors may exist. Aim of this study is to report the use of Mobile ECMO team in controlled DCD.

**Methods:**

From June 2018 to November 2019 our group has worked as a mobile ECMO team for cDCD outside our center. Portable equipment included cannulation material and the ECMO device. The transplant team consisted of 1 transplant coordinator (anesthesiologist-intensivist, ECMO operator and organ extraction supervisor), 1 cardiac surgeon (cannulation), 1 interventional radiologist (cannulation) and one cardiovascular perfusionist (ECMO operator).

**Results:**

Twenty-five cDCD donations were performed. Characteristics of donors and organs retrieved are summarized in Figure 1. From 25 cDCD, 17 livers, 4 lungs, 45 kidneys were obtained. The evolution of grafts and receptors was favorable at day 30 post-transplant.

**Conclusions:**

Mobile ECMO teams may enable cDCD in hospitals without these resources, thereby increasing the pool of donors and optimizing graft outcomes.

**References:**

1. Oniscu GC et al. Am J Transplant 14:2846 2854, 2014.

2. Eren EA et al. Exp Clin Transplant 5:463 470, 2016.

**Fig. 1 (abstract P069).**
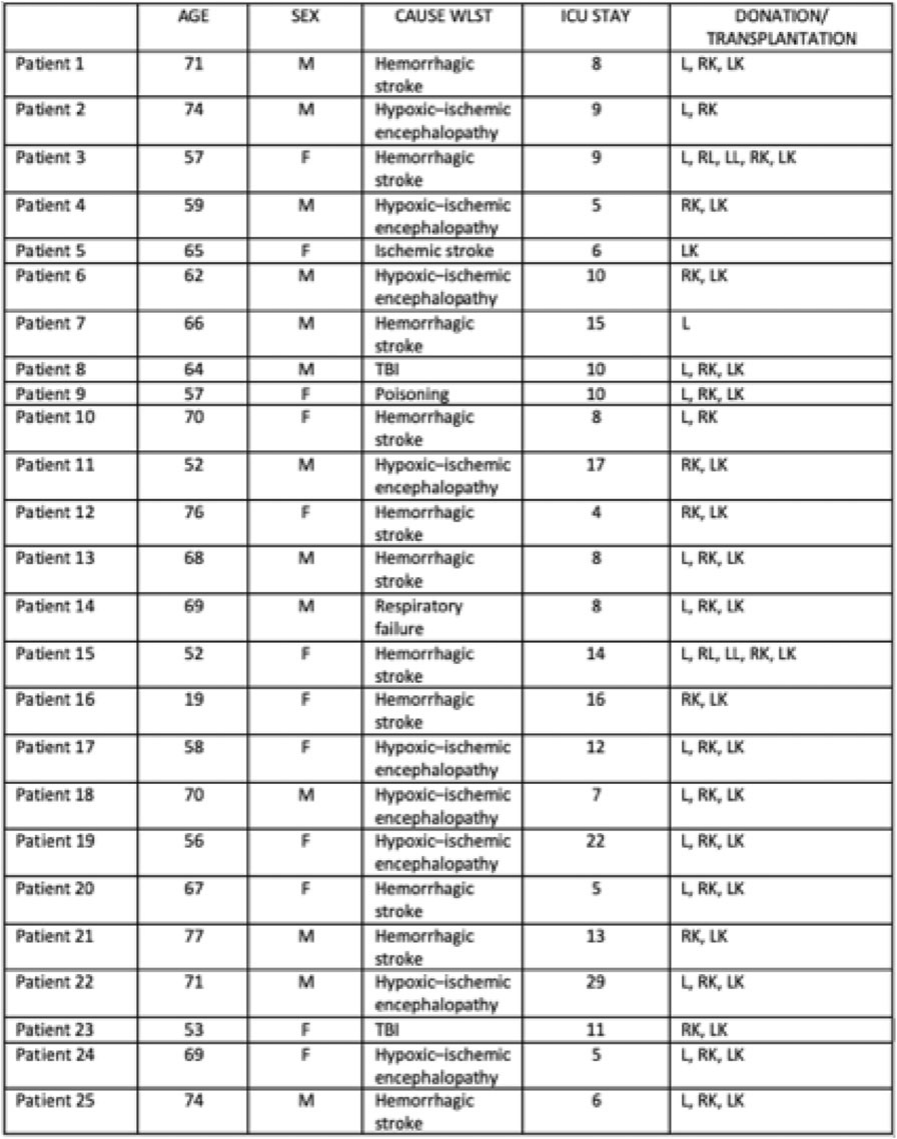
WLST, withdrawal of life sustaining therapy; ICU, intensive care unit; M, male; F, female; TBI, traumatic brain injury; L, liver; RL, right lung; LL, left lung; RK, right kidney; LK, left kidney

## P070 Veno-venous extracorporeal membrane oxygenation (ECMO) as a bridge to lung transplant

### C Palfreeman, P Vila, J Sokhi, A Hurtado-Doce

#### Harefield Hospital, Critical Care, Harefield, United Kingdom

**Introduction:**

The demand for donor lungs is still considerably greater than supply. 17% of patients listed for a lung transplant die within one year [1]. Veno-venous (VV) ECMO is an effective bridging strategy for patients listed for transplant, with end stage respiratory failure, who acutely deteriorate.

**Methods:**

A retrospective observational study of patients supported by VV ECMO whilst awaiting lung transplantation, over a 67-month period (March 2014- October 2019) at Harefield Hospital, in the UK.

**Results:**

32 patients with an acute deterioration of their end-stage lung disease required VV ECMO support as a bridge to transplantation (BTT). The mean age was 34.8 years (range 19-57), 62% were male patients. Aetiology included; end stage cystic fibrosis (n=24, 75%), pulmonary fibrosis (n=4, 12.5%), previous lung transplant failure (n=2, 6.3%), bronchiectasis (n=1, 3.1%) and pulmonary hypertension (n=1, 3.1%). Average duration of ECMO support pre-lung transplant was 11.1 days (range 1-39 days). Cannulation was femoral-femoral (n= 20), Avalon Elite® (n = 6) and femoral – jugular (n= 6). 19 patients (59.4%) underwent lung transplant on VV ECMO support, the others required conversion to veno-arterial (VA) ECMO or cardiopulmonary bypass, most commonly due to hemodynamic compromise. Average duration of invasive ventilation post-transplant was 19.8 days, with 18 patients (56.3%) requiring a tracheostomy. The mean ICU stay was 25.6 days, with an ICU mortality of 21.9% (n =7); in keeping with the literature [2]. However, patients requiring intubation despite VV ECMO (n=7), pre-transplant had a far higher mortality (n=5, 71.4%).

**Conclusions:**

VV-ECMO is a recognized support therapy for patients awaiting lung transplant. Our overall survival to discharge of 78.1%, suggests favourable outcomes are achievable.

**References:**

1. NHS Blood and transplant, 2017. Annual report on cardiothoracic organ transplantation 2016/17.

2. Chiumello D et al. Crit Care 19:19, 2015.

## P071 What is the useful coagulation and fibrinolysis marker for predicting extracorporeal membrane oxygenation circuit exchange due to intra-circuit thrombus?

### Y Izutani, K Hoshino, S Morimoto, K Muranishi, J Maruyama, Y Irie, Y Kawano, H Ishikura

#### Fukuoka University Hospital, Emergency and Critical Care Center, Fukuoka-shi, Japan

**Introduction:**

A thrombus formation is one of the most frequent and adverse complications during extracorporeal membrane oxygenation (ECMO) support. Previous studies have reported that increased D-dimer is a useful predictor of thrombus formation within the ECMO circuit. The purpose of this study was to identify coagulation/fibrinolysis markers for predicting the replacement of ECMO circuit due to intra-circuit thrombus during ECMO support.

**Methods:**

Fourteen patients who underwent veno-venous ECMO for acute respiratory failure between January 2014 and December 2018 were enrolled. These patients received a total of 125 days of ECMO support. Of these, 9 days (times) on which the ECMO circuits were replaced was regarded as the replacement group, while the remaining 116 days were considered as the non-replacement group. The several coagulation/fibrinolysis markers were routinely measured every day during ECMO support. we compared with the levels of these markers between two group to identify the most relevant marker for ECMO circuit replacement due to thrombus.

**Results:**

The mean duration of ECMO support was 9±11 days, and the mean number of ECMO circuit replacement was 0.6±1.0 times per patient. D-dimer, Thrombin-antithrombin complex (TAT), Plasmin-α2 plasmin inhibitor complex (PIC), and soluble fibrin (SF) were significantly higher in the replacement group rather than in the non-replacement group (P < 0.01, respectively). According to a multivariate analysis, SF was the only independent predictor of ECMO circuit replacement due to thrombus. The odds ratio (95% confidence intervals) for SF (10 μg/mL) was 1.2 (1.1-1.3). The area under the curve and optimal cut-off value were 0.94 and 85 ng/mL for SF, respectively (sensitivity, 100%; specificity, 85%).

**Conclusions:**

From these results, we concluded that SF may be the useful marker rather than D-dimer for predicting the replacement of ECMO circuit due to intra-circuit thrombosis.

## P072 Inhomogeneity of lung elastance in patients who underwent veno-venous extra corporeal membrane oxygenation (V-V ECMO)- a computed tomography scan study

### RD Di Mussi^1^, RI Iannuzziello^2^, FM Murgolo^2^, FD De Carlo^2^, E Caricola^2^, NA Barrett^3^, LC Camporota^3^, SG Grasso^2^

#### ^1^Università degli studi di Bari "Aldo Moro", Department of emergencies and organ transplant, Bari, Italy; ^2^Università degli studi di Bari "Aldo Moro", Bari, Italy; ^3^Department of Adult Critical Care, Guy´s and St Thomas´ NHS Foundation Trust, King´s Health Partners, London, UK

**Introduction:**

In patients with acute respiratory distress syndrome (ARDS), nonaerated, poorly aerated, and normally aerated regions coexist to variable degrees in lung parenchyma. The recruitment maneuvers aim to re-open collapsed lung tissue. In a theoretical point view, this strategy may also prevent the normal aerated lung tissue hyperinflation [1]. The objective of our study was to evaluate lung characteristics in terms of Hounsfield Units (HU), volume and elastance before and after a recruitment maneuver.

**Methods:**

In 37 patients with severe ARDS who underwent V-V ECMO, computed tomography scans (CT-scans) at 5 cmH_2_O of continuous positive airway pressure (CPAP) and 45 cmH_2_O were performed. The same CT image was selected at the two different levels of pressure. The distribution of lung opacities, in terms of HU, was classified using the “UCLA” colour coding table (OsiriX image processing software, Geneva, Switzerland). Correspondent lung regions of about 1020 voxels were selected. The quantitative analysis, in terms of Volume air (Vair) was performed with Maluna software (Version 3.17; Maluna, Goettingen, Germany). Elastance was calculated as the pressure(cmH_2_O)/ Vair (ml) ratio.

**Results:**

See Figure 1.

**Conclusions:**

Lung inhomogeneity occurs also after recruiting maneuvers. Our data confirm that the elastance of recruited lung regions is higher than the elastance of the normal aerated lung regions at low positive end-expiratory pressure (PEEP) (baby lung). On the contrary the “baby lung” frequently develops hyperinflation. The unpredictable pattern of distribution of volume after recruitment maneuverers may explain the controversial role of PEEP during the ARDS treatment.

**References:**

1. Camporota L et al. Crit Care Med 47:1177-1183, 2019

**Fig. 1 (abstract P072).**
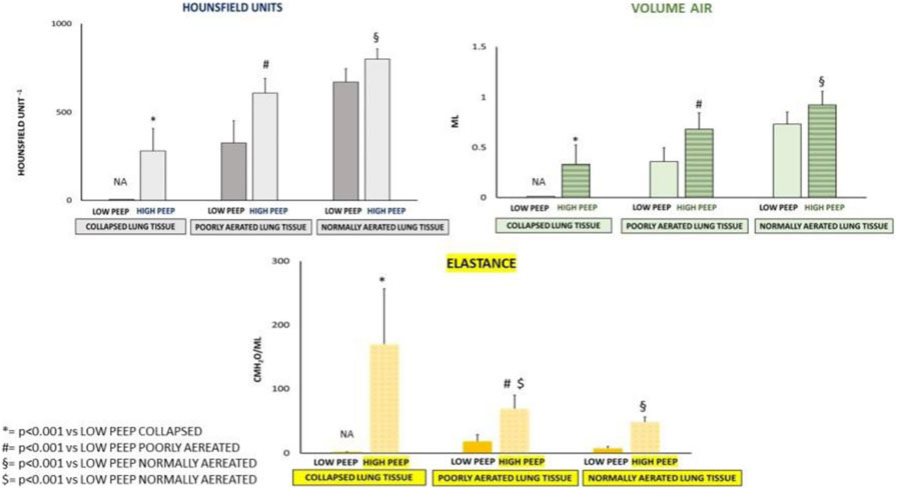
Variations in terms of HU, Vair and elastance in the correspondent lung regions at the two different levels of CPAP

## P073 Phase 3, pilot, prospective, randomized, single blinded, multicenter, controlled, two-arm trial on antithrombin supplementation during extracorporeal membrane oxygenation. the GATRA (Grifols Antithrombin Research Awards) study

### M Panigada^1^, A Cucino^1^, E Cipriani^1^, S De Falco^1^, E Spinelli^1^, G Occhipinti^2^, G Panarello^2^, A Arcadipane^2^, A Pesenti^1^, G Grasselli^1^

#### ^1^Fondazione IRCCS Ca´ Granda Ospedale Maggiore Policlinico, Intensive Care Unit, Milano, Italy; ^2^Istituto Mediterraneo per i Trapianti e Terapie ad Alta Specializzazione (ISMETT), Intensive Care Unit, Palermo, Italy

**Introduction:**

Supplementation of antithrombin (AT) might decrease heparin requirement to achieve an adequate level of anticoagulation during extracorporeal membrane oxygenation (ECMO). Formal recommendations on target, timing, and rate of AT supplementation are lacking. We conceived this study to evaluate the effect of prolonged AT supplementation in adult patients requiring veno-venous ECMO for respiratory failure on heparin dose, adequacy of anticoagulation and safety

**Methods:**

Before ECMO start patients were randomized to either receive AT supplementation to maintain a functional AT level between 80 and 120% (AT supplementation group) or not (control group) for the entire ECMO course. Anticoagulation was provided with unfractionated heparin following a standardized protocol [1]. The primary outcome was the dose of heparin required to maintain the ratio of activated partial thromboplastin time between 1.5 and 2. Secondary outcomes were the adequacy of anticoagulation measured with anti-Factor Xa and the incidence of hemorrhagic and thrombotic complications and amount of blood products transfused

**Results:**

From August 2017 to March 2019, 72 patients were screened and 49 enrolled in the study. Forty-eight patients underwent final analysis (1 patient was excluded for erroneous randomization). Patients did not differ with regards to baseline characteristics. AT (%) was 82.6±23.3 % in the control group and 106.2±22.3 %, p=0.001 in the treatment group. Supplementation of AT did not decrease heparin dose (14.9±6.2 vs 13.8±6.6 IU/Kg/h in the control and treatment group respectively, mean difference: -1.2 (95% CI: -3.7; - 1.2), p=0.33) and anti-Factor Xa levels. Bleeding, blood product transfusions and thrombosis were comparable in the two groups

**Conclusions:**

AT supplementation did not decrease heparin requirement in patients on veno-venous ECMO for respiratory failure

**References:**

1. Panigada et al. Ann Intensive Care 8:1–9, 2018

## P074 Prone positioning while extracorporeal membrane oxygenation therapy in severe ARDS

### J Rilinger, V Zotzmann, X Bemtgen, PM Biever, D Duerschmied, C Bode, DL Staudacher, T Wengenmayer

#### Heart Center Freiburg University, Department of Cardiology and Angiology I, Freiburg, Germany

**Introduction:**

Prone positioning (PP) improves survival in patients with severe ARDS. However, there is little evidence if PP is able to improve outcome in veno-venous extracorporeal membrane oxygenation (ECMO) patients. Therefore, we performed a retrospective analysis of patients treated with PP while ECMO support.

**Methods:**

We report retrospective data of a single-center registry of all patients treated with veno-venous ECMO for severe ARDS between October 2010 and Mai 2018 at the Interdisciplinary Medical Intensive Care Unit at the Medical Center, University of Freiburg, Germany. ECMO weaning success and survival were analysed before and after propensity score matching. Moreover, predictive factors for survival in patients with PP while ECMO were analysed.

**Results:**

A total of 158 patients with complete medical data could be analysed (age 52.2 years, 67% male). 38 patients (24.1%) received PP while ECMO therapy. There was no difference in ECMO weaning rate (47.4% vs. 46.7%, p=0.940) or ICU and hospital survival (36.8% vs. 36.7%, p=0.984, Fig. 1 A) between patients with and without PP while ECMO therapy. Propensity score matching confirmed this finding (survival: 36.8% vs. 42.1%, p=0.639). However, early initiation of PP while ECMO support (cut off via Youden-Index <0.71 days) was associated with superior survival in univariate and multivariate analysis (Odds ratio 20.6 [95%-CI 1.4-312.9], p=0.029, Fig. 1 B).

**Conclusions:**

This retrospective analysis was not able to show a survival benefit for additive PP to ECMO support in general. Early initiation of PP could be an important factor for improving survival in this setting and should be considered in a randomized controlled trial for further evaluation.

**Fig. 1 (abstract P074).**
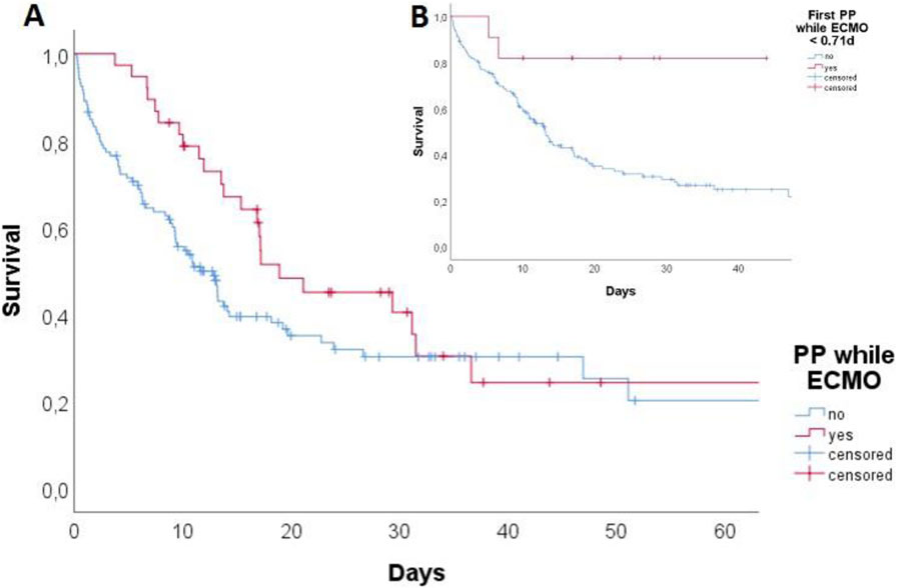
A) Survival of ECMO patients with vs. without prone positioning while ECMO. Initial survival differences equalized in Kaplan-Meier survival curve after 30 days (Log rank=0.109). B) Survival depending on early prone positioning after initiation of ECMO support. Patients treated with early PP while ECMO showed a superior survival to patients treated with late PP or without PP while ECMO. Optimal cut off value for duration of ECMO initiation to first PP was calculated using ROC-analysis (AUC = 0.789) and the Youden-Index. Highest sensitivity and specificity for beneficial survival were achieved for a beginning of PP in <0.71 days. (Log rank=0.018). PP: Prone positioning

## P075 Cause-specific mortality during extracorporeal membrane oxygenation, a single center review of medical records

### M Panigada, D Tubiolo, P Properzi, G Grasselli, A Pesenti

#### Fondazione IRCCS Ca´ Granda Ospedale Maggiore Policlinico, Intensive Care Unit, Milano, Italy

**Introduction:**

Mortality during Extracorporeal Membrane Oxygenation (ECMO) settles around 35% and the occurrence of bleeding during ECMO is associated with a high mortality rate. However, cause-specific mortality is rarely reported, probably due to the difficulty of its classification. The purpose of the study was to evaluate the agreement between two expert ICU physician in the classification of the cause of death of patients supported with ECMO for either respiratory or cardiac support.

**Methods:**

Two Intensive Care Unit (ICU) expert staff physicians independently reviewed the entire medical records of all ECMO patients who died before ICU discharge from January 2011 to September 2019 at Fondazione IRCCS Ca’ Granda, Milan. They were asked to choose the cause of patient’s death among six categories. In case of disagreement, a third expert adjudicated the case. The two reviewers were also asked whether, in their opinion, bleeding during the last 24 hours contributed to death. ELSO definition of major bleeding [1] during the last 24 hours was also recorded for each patient.

**Results:**

Two-hundred and two patients were supported with ECMO of whom 70 (34.6%) died. Most of these patients (N=53, 75.7%) died during ECMO. Interrater agreement for cause-specific mortality between the two expert physicians was substantial (k 0.71, SE 0.07, p<0.01) Of the 14 discordant cases 6 were categorized as refractory respiratory failure and 4 as multiorgan failure and septic shock respectively. The distribution of cause-specific mortality is shown in Figure 1. Major bleeding (ELSO) was present in 27 (38.6%) patients, only in 9 (33.3%) of them bleeding contributed to death according to the reviewers.

**Conclusions:**

The most common cause of death during ECMO in our cohort was multiorgan failure with substantial agreement between the two reviewers. Apparently, major bleeding contributed to death only in a small proportion of patients.

**References:**

1. https://www.elso.org/portals/0/files/elsoanticoagulationguideline8-2014-table-contents.pdf

**Fig. 1 (abstract P075).**
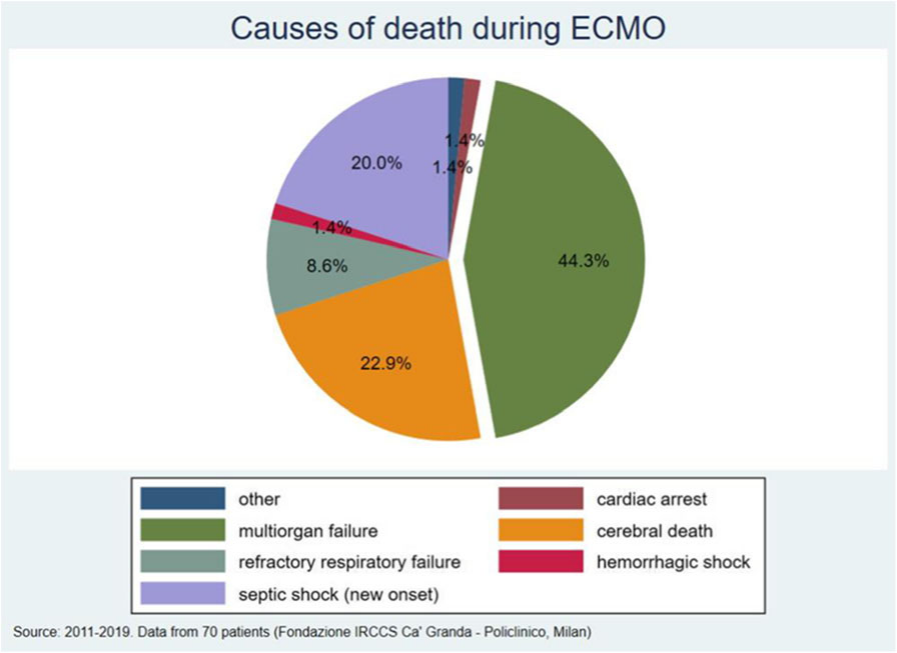
Causes of death during ECMO

## P076 Non-invasive mechanical ventilation in veno-venous extracorporeal membrane oxygenation

### J Rilinger, V Zotzmann, X Bemtgen, PM Biever, D Duerschmied, C Bode, DL Staudacher, T Wengenmayer

#### Heart Center Freiburg University, Department of Cardiology and Angiology I, Freiburg, Germany

**Introduction:**

Veno-venous extracorporeal membrane oxygenation (ECMO) support can be combined with a variety of different non-invasive ways to deliver oxygen to the patient’s lung. Several positive effects might be linked to this so called “awake ECMO”. So far there is little evidence about indications and outcome of this approach.

**Methods:**

We report retrospective registry data on all ARDS patients treated with ECMO support at a university hospital between 10/2010 and 04/2019. In a systematic review of medical records, we distinguished between patients with invasive mechanical ventilation (IMV) from the initiation of ECMO therapy (IMV group) and patients that received any kind of non-invasive oxygen supply (non-IMV group).

**Results:**

A total of 276 patients could be analysed. 16 (5.8%) patients received non-IMV ECMO support. Patients receiving non-IMV ECMO therapy showed severe underlying pulmonary disease and immunosuppression (Fig. 1). These patients had higher rates of lung fibrosis, long-term oxygen therapy, pulmonary hypertension, renal insufficiency and immunosuppression (p<0.05). 12 of 16 patients (75%) required IMV during the hospital stay in average 5.3±5.0 [0.8-17.1] days after ECMO initiation. Reasons were hypoxia despite of ECMO, insufficient ECMO-flow, insufficient protective reflexes or patient agitation. Patients with initially non-IMV ECMO support showed a numerical but not significant lower ICU and hospital survival (25.0% vs. 45.4%, p=0.111).

**Conclusions:**

Non-IMV ECMO support was applied in patients with severe underlying pulmonary disease and/or immunosuppression. In a high proportion of patients the ventilation regime had to be switched from non-invasive to invasive. Survival in this very selected cohort was low. In this retrospective analysis no evident benefit for a non-invasive ventilation strategy could be found. The high proportion of patients who switched from non-IMV to IMV therapy underlines the need for rigorous patient selection.

**Fig. 1 (abstract P076).**
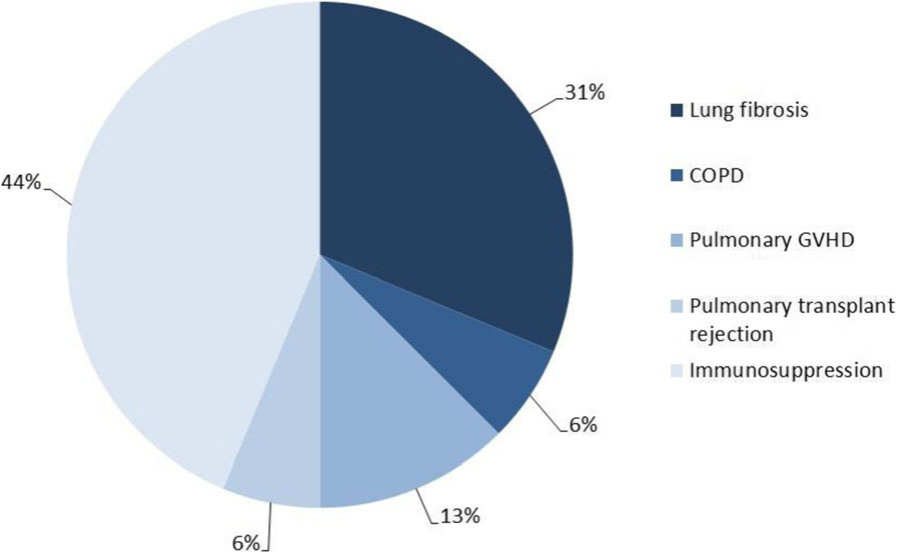
Underlying pulmonary disease or status of immunosuppression in ECMO patients without invasive mechanical ventilation

## P077 Intra-hospital transportation on extracorporeal membrane oxygenation (ECMO) - a single centre experience in Ireland.

### Z Siddique, S O´Brien, E Carton, I Conrick-Martin

#### Mater Misericordiae University Hospital, Department of Critical Care Medicine, Dublin, Ireland

**Introduction:**

The objective of this study is to evaluate intra-hospital transportation of patients on extracorporeal membrane oxygenation (ECMO). It is a retrospective analysis of prospectively collected database, performed as part of ongoing quality improvement initiatives.

**Methods:**

The setting of this study is an 18-bed, combined surgical and medical adult Intensive Care Unit (ICU) located in a 570-bed hospital that serves as the national referral centre for Cardiothoracic Surgery, Heart & Lung transplantation and ECMO in Ireland. We reviewed 33 months of data (from 2017 to 2019) regarding patients admitted to our critical care unit who required intra-hospital transfer for diagnostic and/or therapeutic interventions. We also compared the data to available local guidelines.

**Results:**

23 patients were transported on ECMO on a total of 28 occasions; the most common indication being CT brain (Table 1). ECMO cannulation sites were peripheral in 20 patients, 3 patients were centrally cannulated. Median time from start of the transfer until the patient was returned to ICU was 50 minutes (range: 35-195). The ECMO console was placed on a dedicated ECMO trolley apart from two occasions where it was placed on the patient's bed. Number of staff required for transport was between 4 to 10; with an ICU Consultant as team leader. ECMO specialist nurses were always present on the transport team. 27 transfers were during normal working hours with 1 happening on a weekend. A total of 12 complications occurred during the transports, of which 1 was significant and 11 were not. The significant complication encountered was Ventricular Tachycardia in a V-A ECMO patient which required electrical defibrillation. No adverse events related to transport were seen following return to ICU.

**Conclusions:**

In this single-centre study, we have demonstrated safe intra-hospital transport of ECMO patients. The use of local guidelines, appropriate personnel and performance during normal working hours is recommended.

**Table 1 (abstract P077).** Summary of results

Total number of transfers	28
Most common indication for transfer	CT brain
Sites of cannulation	Peripheral > central
Staff members involved in transport	4 to 10
Time of transportation	35 to 195 minutes
Complications arising during transport	12

## P078 A novel approach for flow simulation in ECMO rotary blood pumps

### A Supady^1^, C Benk^2^, J Cornelis^3^, C Bode^1^, D Duerschmied^1^

#### ^1^Heart Center Freiburg University, Cardiology and Angiogiology I, Freiburg, Germany; ^2^Heart Center Freiburg University, Department of Cardiovascular Surgery, Freiburg, Germany; ^3^FIFTY2 Technology GmbH, 79108 Freiburg, Germany

**Introduction:**

Extracorporeal membrane oxygenation (ECMO) is used increasingly in critically ill patients suffering from acute respiratory failure, cardiogenic shock or cardiac arrest. However, this therapy can have deleterious side effects such as bleeding or clotting complications and hemolysis. These complications are particularly caused by physical stress acting upon the blood components while passing through the ECMO system, especially within the rotary pump. We here present a novel approach to simulate blood flows through rotary blood pumps used in current ECMO systems in order to better understand the genesis of these complications.

**Methods:**

Geometries of the Xenios DP3 (Xenios AG, Heilbronn, Germany) rotary pump were reconstructed by CT-scans and manual measurements using computer-aided design (CAD). The Computational Fluid Dynamics (CFD) simulation was performed using the software PreonLab (FIFTY2 Technology GmbH, Freiburg, Germany), which implements a mesh-free Lagrangian method requiring minimal preprocessing of the CAD data. The geometries are introduced to the simulation model as tessellated surfaces. Five operating points have been specified by the rotation of the centrifugal fan and the corresponding inflow and outflow of blood. The blood is approximatively modelled as a Newtonian fluid with a density of 1040 kg/m^3^.

**Results:**

PreonLab allows detailed assessment of the blood flow while passing through the rotary pump including analysis of local flow rates, pressure gradients and shear stress acting upon the blood. Dead zones in the fluid flow can be detected which gives reference points for optimizations of the pump design.

**Conclusions:**

For the first time, we demonstrate a novel approach for flow simulation in an ECMO rotary pump (Figure 1). This approach may help better understand hemodynamics within the extracorporeal system to define optimal operating points or re-design components aiming to limit hemolysis, coagulation disorders and bleeding in seriously ill patients.

**Fig. 1 (abstract P078).**
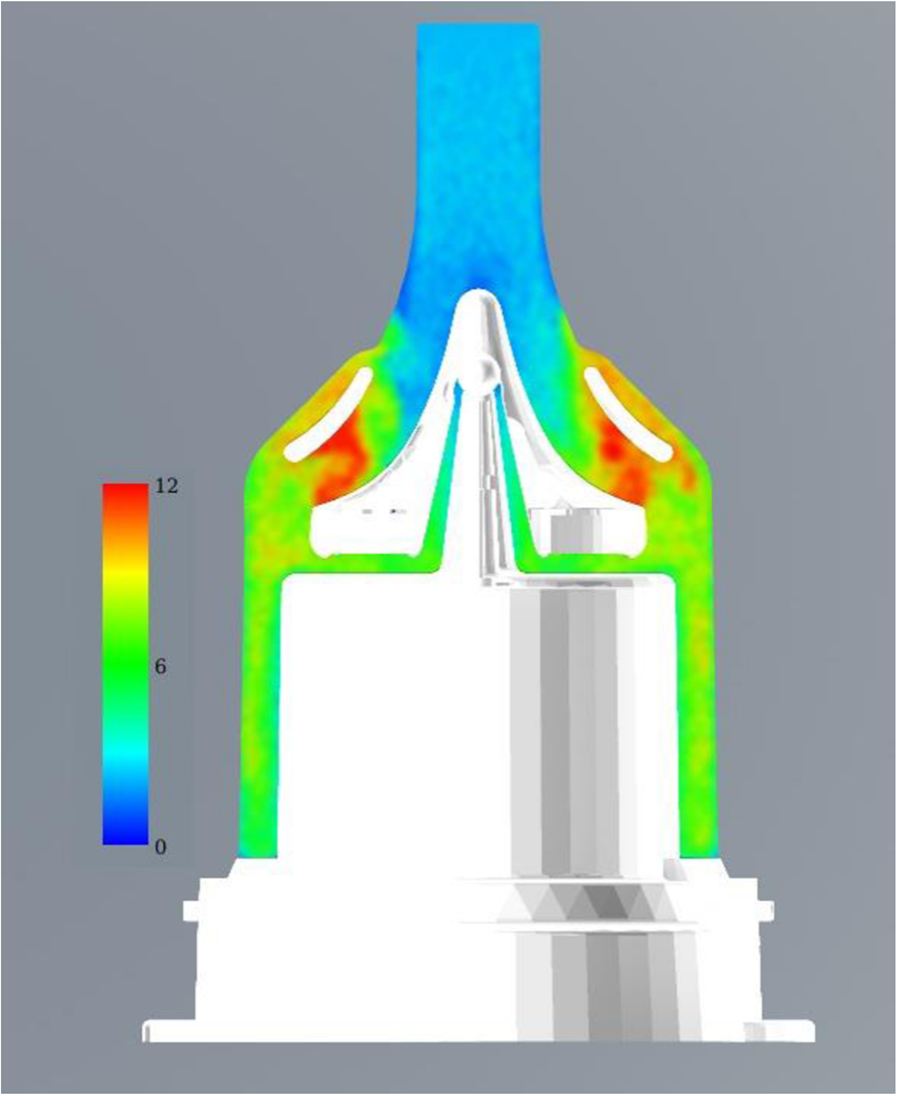
Visualization of fluid velocity (in m/s) within the rotary pump (cross-section)

## P079 One-year experience of bedside percutaneous VA-ECMO decannulation in a territory ECMO center in Hong Kong

### KM Fong, SY Au, PW Leung, KC Shek, HJ Yuen, SK Yung, HL Wu, SO So, WY Ng, KH Leung

#### Queen Elizabeth Hospital, Intensive Care Unit, Hong Kong

**Introduction:**

When veno-arterial extra-corporeal membrane oxygenation (VA-ECMO) support can be terminated, arteriotomy wounds of the patients of are traditionally closed by open repair in the operation theaters. Lots of manpower are involved and timeslots in operating theaters are scarce. Transport of the critically-ill is risky. Successful VA-ECMO decannulation using percutaneous device called ProGlide has been reported and our group had adopted and modified this approach [1].

**Methods:**

This is a retrospective study analyzing the one-year experience of bedside VA-ECMO decannulation. Our institution is a 23-bed tertiary ECMO referral center in Hong Kong. Our first bedside decannulation was performed in November 2018, and since then, this practice had replaced the traditional open repair, unless contraindicated. Data from November 2018 to October 2019 were analyzed.

**Results:**

In the study period, 39 patients received VA-ECMO. 28 survived to decannulation and 25 received bedside percutaneous decannulation. Their median age was 59 (52-67). The default arterial catheter size was 17Fr, with 15 Fr in 3 cases and 19Fr in one. Five (20%) failed percutaneous closure and they were subsequently surgically repaired without additional complications. Among the 20 successful cases, 2 ProGlides were used in 15 cases and 3 used in 5 cases. The procedure time was 27 minutes (15-45), which was faster than 2.5 hours by open repair. Median blood loss was 300mL (250-400). Post procedural clinical and ultrasound Doppler examination revealed minor complications in 4 patients, including 2 arterial clots and one pseudoaneurysm who were managed conservatively and one wound infection requiring exploration. There were no other major vascular complications.

**Conclusions:**

Bedside percutaneous VA-ECMO decannulation is safe and effective.

**References:**

1. Au SY et al. J Emerg Crit Care Med. 4:4, 2020.

## P080 CD52 positive NKT cells predict survival during veno-arterial extracorporeal membrane oxygenation

### EJ Kort^1^, MW Weiland^1^, EG Eugster^1^, EG Grins^2^, SF Fitch^3^, GM Marco^4^, T Timek^4^, ML Leacche^4^, TJ Boeve^4^, SJ Jovinge^5^

#### ^1^Van Andel Institute/Spectrumhealth, Grand Rapids, United States; ^2^Scania Univ Hosp Lund/Lund Univ, Dep of Clinical Medicine, Scania Univ Hosp Lund/Lund Univ Lund Sweden, Lund, Sweden; ^3^DeVos CV Res/Fredrik Meijer Heart and Vascular Inst Spectrumhealth, Spectrumhealth, Grand Rapids, MI United States, Grand Rapids, MI, United States; ^4^DeVos CV Res/Fredrik Meijer Heart and Vascular Inst Spectrumhealth, Grand Rapids, MI, United States; ^5^Van Andel Institute/Spectrumhealth, DeVos Cardiovascular Research Program, Grand Rapids, United States

**Introduction:**

Extra corporeal life support (ECLS) continues to be associated with high mortality rates. Our ability to predict outcome prior to initiation ECLS remains limited. Here we take a single cell RNASeq approach in an effort to identify novel immune cell types that are associated with—and may contribute to—survival on ECLS.

**Methods:**

Whole genome transcriptomic profiles were generated from ~40,000 peripheral blood monocytes obtained from 38 patients at the time of cannulation for veno-arterial ECLS (VA-ECLS). Within each subpopulation, differential gene expression analysis was performed to identify new markers associated with survival. Findings were validated in a additional cohorts by flow cytometry.

**Results:**

Surviving patients had significantly higher proportions of CD8^+^ NKT cells (CD3^+^/CD8^+^/CD19^-^/CD56^+^) that were CD52^+^ (p = 0.001, FDR < 0.05) (Figure 1). To validate this observation, we performed FC analysis of a second cohort of 20 patients. For each patient, we quantified the proportion of CD8^+^ NKT cells that were CD52^+^. Using the median proportion as the cutoff, we again found that a high proportion of CD52^+^ cells among CD8^+^ NKT cells was predictive of 48 hour survival (p=0.024). We noted that while high levels of CD52+ cells among the CD8+ NKT cells was protective in this cohort of VA-ECLS patients, this relationship did not hold for patients with sepsis. As only a few the VA-ECLS patients were septic, we analyzed a third cohort of septic ECLS patients. We observed that high levels of CD52+ cells among the CD8+ NKT populations was not protective in this population.

**Conclusions:**

The proportion of CD8+ NKT cells that are positive for CD52 is predictive of survival among patients undergoing VA-ECLS for non-infection related indications.

**Fig. 1 (abstract P080).**
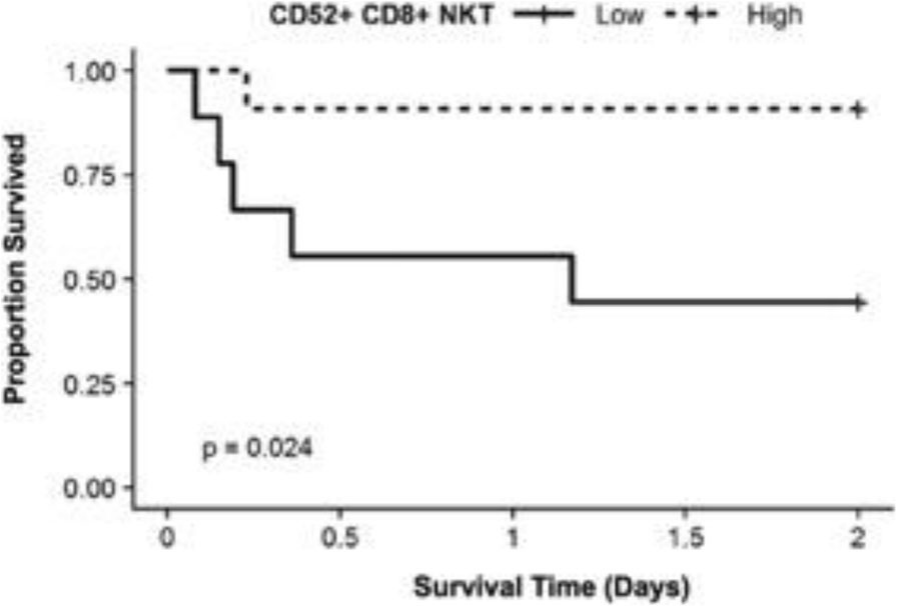
72 h survival

## P081 Neurologic complications in adult postcardiotomy cardiogenic shock patients receiving venoarterial extracorporeal membrane oxygenation: a cohort study

### H Wang, D Hou

#### Beijing Anzhen Hospital, Capital Medical University, Center for Cardiac Intensive Care, Beijing, China

**Introduction:**

This study aims to describe the prevalence of neurologic complications and hospital outcome in adult post-cardiotomy cardiogenic shock (PCS) patients receiving veno-arterial extracorporeal membrane oxygenation (V-A ECMO) support and factors associated with such adverse events.

**Methods:**

415 adult patients underwent cardiac surgery and received V-A ECMO for more than 24h because of PCS. Patients were divided into two groups: those who developed a neurological complication and those who did not (the control group). Multivariable logistic regression was performed to identify factors independently associated with neurologic complications.

**Results:**

Neurologic complications occurred in 87 patients (21.0%), including cerebral infarction in 33 patients (8.0%), brain death in 30 patients (7.2%), seizures in 14 patients (3.4%), and intracranial hemorrhage in 11 (2.7%) patients. In-hospital mortality in patients with neurologic complications was 90.8%, compared to 52.1% in control patients (*p* <0.001). In a multivariable model, the lowest systolic blood pressure (SBP) level pre-ECMO (OR, 1.12; 95% CI: 1.08–1.17) and aortic surgery combined with coronary artery bypass grafting (OR, 9.22; 95% CI: 2.10–40.55) were associated with overall neurologic complications (Figur 1). Lowest SBP (OR, 1.22; 95% CI: 1.15–1.31) was the only risk factor of brain death as well. Coagulation disorders (OR, 9.75; 95% CI: 1.83–51.89) and atrial fibrillation (OR, 12.19; 95% CI: 1.22–121.61) were shown to be associated independently with intracranial hemorrhage, whereas atrial fibrillation (OR, 8.15; 95% CI: 1.31–50.62) was also associated with cerebral infarction.

**Conclusions:**

Neurologic complications in adult PCS patients undergoing VA ECMO support are frequent and associated with higher in-hospital mortality. Identified risk factors of neurologic complications might help to improve ECMO management and might reduce their occurrence.

**Fig. 1 (abstract P081).**
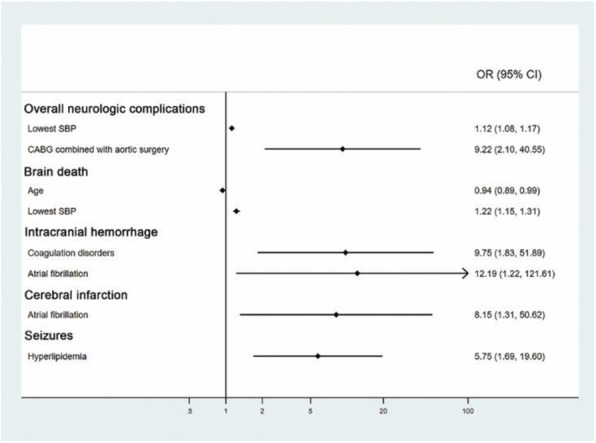
Risk factors of neurologic complications during extracorporeal membrane oxygenation. SBP, systolic blood pressure; CABG, coronary artery bypass grafting

## P082 Use of levosimendan during weaning of mechanical circulatory support

### S Hendrickx, J Verbeke, N De Neve, K De Decker

#### OLV Aalst, Intensive Care, Aalst, Belgium

**Introduction:**

The use of calcium sensitizers has grown enormously in the last decade, probably due to their interesting pharmacodynamic properties. Levosimendan (LS) is frequently administered in patients under mechanical circulatory support. We performed a retrospective evaluation of patients treated with LS prior to weaning from mechanical support. This evaluation was combined with a review of the literature.

**Methods:**

A query of our ICU patient data management system revealed 22 patients receiving LS prior to or during VAD/ECLS support. Outcome data were obtained from the patients medical records.

**Results:**

Of our 22 patients, 78% was successfully weaned off ECLS. Fourteen patients (63 %) died before being discharged of whom 5 while on ECLS support. Of the weaned patients, 9 died afterwards. 4 of the converted patients needed subsequent veno-venous ECLS support for right ventricular support after the implantation. Survival to discharge ratio for the whole group was 31 %. More detailed demographic results can be found in Table 1.

*Literature review*

A pubmed search using the terms “(ECMO OR ECLS) AND LS AND weaning” resulted in 7 publications which dealt specifically with weaning of ECLS support. Several weaning approaches are available, however poor outcome has remains a problem. Some recent studies show a possible beneficial effect of LS infusion prior to weaning from ECLS. However most of these studies are retrospective or observational at best. Because LS is primarily reserved for the most severe cases, outcome interpretation is difficult. Overall weaning success ranges from 82%-92% and variation is very dependant of inclusion criteria.

**Conclusions:**

The calcium sensitizer LS can be used when weaning off patients from ECLS, certainly given its low incidence of complications. Future, large randomized trials are however needed in order to confirm this strategy.

**Table 1 (abstract P082).** Patient demographics and mortality

	Discharge Home	Deceased		Total
		On ECLS or within 24h	After weaning	
All	7 (31,8%)	5 (22.7%)	10 (45.4%)	22
Male	7 (41.2%)	4 (23.5%)	5 (29.4%)	17
Female	0 (0%)	1 (20%)	4 (80%)	5
<60y	5 (45,5%)	2 (18.2%)	4 (36.4%)	11
>60y	3 (27.2%)	3 (27.2%)	5 (45.5%)	11

## P083 A series of two hemorrhage-associated pheochromocytoma crises precipitating cardiogenic shock requiring extracorporeal life support.

### P O´Connor, C Costello, S Egan, M Afrasenei, S O´Brien, E Carton, I Conrick-Martin

#### Mater Misericordiae University Hospital, Critical Care Medicine, Dublin 1, Ireland

**Introduction:**

Cardiogenic shock induced by catecholamine excess in pheochromocytoma affects 2.9-7.1% of new presentations, in whom 1.5-5.7% require mechanical circulatory support [1, 2]. Of patients in crises requiring veno-arterial extracorporeal membrane oxygenation (V-A ECMO) mortality is 7% [3]. Hemorrhagic pheochromocytoma is a known but rare precipitant of crisis [3].

**Methods:**

We included patients presenting to MMUH Jan 2016 – Dec 2019 in cardiogenic shock requiring V-A ECMO with new diagnosis of pheochromocytoma.

**Results:**

Case 1 – 30 year old female presented with abdominal pain & flash pulmonary oedema. Transthoracic echocardiography (TTE) demonstrated inverse Takotsubo’s cardiomyopathy. CT abdomen revealed a hemorrhagic left adrenal mass. Phentolamine precipitated cardiac arrest and following successful resuscitation peripheral V-A ECMO commenced for worsening cardiogenic shock. Decannulated from ECMO after four days, she subsequently underwent uneventful robotic adrenalectomy.

Case 2 – 58 year old female with chest pain and ischemic ECG. TTE revealed Takotsubo’s cardiomyopathy. CT revealed hemorrhage into right adrenal mass. Peripheral V-A ECMO commenced for cardiogenic shock. She awaits surgery following 6 day uncomplicated ECMO course with successful decannulation, extubation, and uptitration of alpha blockade.

**Conclusions:**

Cardiogenic shock is well described in newly diagnosed pheochromocytoma, and crisis may be precipitated by hemorrhage into tumour. V-A ECMO represents a rescue therapy in a subset of these patients refractory to medical management, facilitating cardiac recovery and subsequent definitive surgery.

Consent to publish: written informed consent for publication was obtained from the 2 patients.

**References:**

1. Riester A et al Eur J Endocrinol 173:757–764, 2015

2. Giavarini A et al. Heart 99:1438–1444, 2013

3. Hekimian et al. Ann Intensive Care 6:117, 2016

## P084 Cardiac tamponade complicating extracorporeal membrane oxygenation: a single center experience

### A Fontoura^1^, R Roncon-Albuquerque Jr.^2^, JA Paiva^2^, C Basílio^2^

#### ^1^Unidade Local de Saúde da Guarda, Intensive Care Medicine Department, Guarda, Portugal; ^2^Centro Hospitalar Universitário de São João, Intensive Care Medicine Department, Porto, Portugal

**Introduction:**

Although cardiac tamponade (CT) complicating extracorporeal membrane oxygenation (ECMO) support is seldom reported, risk factors such as vascular cannulation, systemic anticoagulation and recent heart surgery are frequently present. The goal of this study is to assess the prevalence, diagnostic challenges, therapeutic and outcome implications of CT complicating ECMO.

**Methods:**

Retrospective analysis of the Centro Hospitalar Universitário São João ECMO Referral Center (Porto, Portugal) database (10 year period). Of the 466 ECMO cases, CT complicating ECMO support occurred in 5 patients (1.07%): 3 veno-venous (VV) and 2 veno-arterial (VA).

**Results:**

Of the 5 patients, 2 were women and 3 men, with a mean age of 56,4 years. ECMO support was performed during 26,6±22,3 days: 3 for refractory respiratory failure (1 community-acquired pneumonia, 1 pneumocystosis and 1 status asthmaticus), and in 2 for severe cardiogenic shock (1 arrhythmic storm and 1 acute myocardial infarction). CT occurred after 19,6±24,6 days of ECMO support. CT was suspected during VA-ECMO due to sudden hemodynamic deterioration with decreased pulse pressure. In VV-ECMO, CT was suspected due to circulatory collapse, with cardiopulmonary arrest in 2 patients. A large pericardial effusion with collapsed right chambers by transthoracic echocardiography established the diagnosis, in all cases. Patients with VV-ECMO and cardiopulmonary arrest required ECMO-assisted cardiopulmonary resuscitation (E-CPR). Emergent surgical drainage of hemopericardium was required in all cases, with cardiac perforation repair in 2 cases. All patients survived to ICU (42,6±25,6 days) and hospital (55,6±28,9 days) discharge without significant complications.

**Conclusions:**

CT is a rare but life-threatening complication during both VV- and VA-ECMO. A high index of suspicion is needed and bedside transthoracic echocardiography plays a major role for timely diagnosis and treatment. Excellent prognosis is possible with E-CPR and emergent surgical drainage and repair.

## P085 Extracorporeal CO2 removal reduces inspiratory muscle effort during renal replacement therapy in a difficult to wean patient after orthotopic liver transplantation: a case report

### I Cavalli

#### Alma Mater Studiorum - Università di Bologna, Dipartimento di Scienze Mediche e Chirurgiche Anesthesia and Intensive Care Medicine, Policlinico di Sant´Orsola, Bologna, Italy, Bologna, Italy

**Introduction:**

During a spontaneous breathing trial respiratory mechanics can worsen, and respiratory muscle effort can increase, leading to respiratory muscle fatigue, pump failure, hypercapnia and an unsuccessful weaning from mechanical ventilation. This case report discusses the possibility of applying extracorporeal CO_2_ removal (ECCO_2_R) to reduce respiratory muscle effort in a liver transplant recipient who already failed three weaning attempts from mechanical ventilation.

**Methods:**

The ECCO_2_R membrane lung was integrated into a conventional renal replacement therapy circuit and blood flow was increased from 150 to 300 ml/min. Measurements of respiratory mechanics (including esophageal pressure, as shown in Fig. 1) were used to assess the reduction of respiratory effort before and during the application of ECCO_2_R.

**Results:**

After 5 minutes of spontaneous breathing (SB), respiratory rate increased from 12 to 24 breaths/min, PaCO_2_ increased from 39 to 54 mmHg, pressure time product per minute (PTP/b) and pressure time product per liter (PTP/L) increased to 142.9±26.9 cmH_2_O^**.**^s/min (from 78.9±15.3 cmH_2_O^**.**^s/min in PSV), and 13.4±2.5 cmH_2_O^**.**^s/L (from 7.1±1.1 cmH_2_O^**.**^s/L in PSV), respectively. ECCO_2_R was subsequently started with a sweep gas flow of 10 L/min. After 1 hour of ECCO_2_R, respiratory rate decreased to 18 breath/min, PaCO_2_ decreased to 44 mmHg; and PTP/b and PTP/L decreased to 83.9±19.3 cmH_2_O^**.**^s/min and 9.5±1.8 cmH_2_O^**.**^s/L, respectively. The patient was successfully extubated and ECCO_2_R was discontinued after 36 hours.

**Conclusions:**

In a difficult-to-wean patient recovering from orthotopic liver transplantation (OLT), ECCO_2_R can be added to a renal replacement circuit and may reduce the ventilatory demand and prevent severe hypercapnia and respiratory distress (Figure 1), thus facilitating the recovery of adequate spontaneous ventilation and allowing effective weaning from mechanical ventilation.

Consent to publish: written informed consent for publication was obtained from the patient.

**Fig. 1 (abstract P085).**
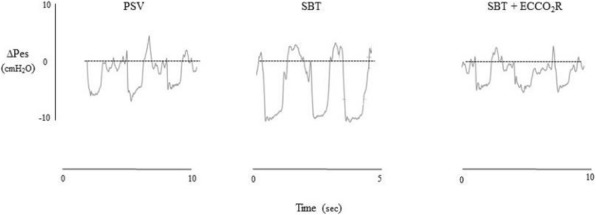
From the left to the right: esophageal pressure (i) during pressure support ventilation (PSV); (ii) during spontaneous breathing trial (SBT); (iii) during SBT + ECCO2R

## P086 AKI during aeCOPD: the potential role of combined renal-respiratory extracorporeal support

### L Zamidei^1^, R Marco^2^, D Atzeni^3^, G Boscolo^4^, F Turani^5^, G Consales^1^

#### ^1^Prato Hospital, emergency and Intensive Care, Prato, Italy; ^2^IRCCS Policlinico San Donato, Anesthesia and Intensive Care Department, San Donato, Italy; ^3^Marino Hospital, Intensive Care Department, Cagliari, Italy; ^4^Ospedale dell´Angelo, Anaesthesia and Intensive Care Department, Mestre, Italy, ^5^Aurelia and European Hospital, Anesthesia and Intensive Care Department, Rome, Italy

**Introduction:**

We describe the use of a low-flow platform (PrismaLung®-Prismaflex®) for combined extracorporeal CO2 removal-renal support (ECCO2R-CRRT) in patients affected by acute exacerbation of chronic obstructive pulmonary disease (aeCOPD) associated to acute renal failure (AKI).

**Methods:**

We evaluated aeCOPD patients submitted to PrismaLung-Prismaflex due to AKI (AKIN >2) associated to hypercapnic acidosis (pH<7.25, pCO2>60 mmHg) during the period May 2016-July 2019 at Prato Hospital ICU. Physiologic data were collected (T0, 24h, 48h, 72h).  Complications and outcome were recorded. Data were presented as mean±DS; Anova Test was used to compare changes over time (significance P<0.05).

**Results:**

14 aeCOPD patients with AKI and hypercapnic acidosis were identified (75±9yr SOFA 13±2). Rationale for ECCO2R-CRRT was to treat AKI and to facilitate extubation in invasively ventilated patients (IMV 9patients) or to avoid intubation (4patients at risk of non-invasive ventilation-NIV- failure). Hemo-diafiltration (effluent-flow 35 ml/kg/h) was delivered through a 13fr-double-lumen-cannula; 350 ml/min blood-flow with 10lt oxygen sweep-gas-flow and aPTT 1.5-2 baseline were maintained (iv-heparin). In all cases respiratory and metabolic parameters improved without complications (Figure 1). ECCO2R-CRRT facilitated extubation (4 out 9 IMV pts). In 4 out of 5 pts at risk of NIV failure, it avoided IMV. Treatment mean duration was 73±31 hours, mean lenght of ICU stay was 6±4 days. All patients survived to the treatment, nevertheless 2 patients died due to irreversible multiple MOF.

**Conclusions:**

In our aeCOPD series PrismaLung®-Prismaflex® facilitated weaning from IMV and avoided intubation in patients at risk of NIV failure without complications. These positive results may be related to minimal invasiveness of the low–flow device used and may constitute the rationale for a larger randomized controlled trial.

Consent: Written informed consent for data publication has been obtained.

**Fig. 1 (abstract P086).**
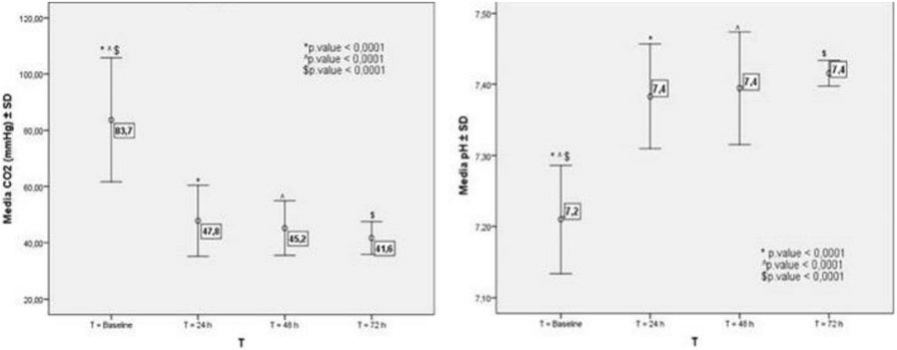
Mean CO2 and pH during ECCO2R-CRRT treatment

## P087 Extracorporeal carbon dioxide removal rates during the SUPERNOVA trial: estimates from a mathematical model

### JK Leypoldt^1^, J Kurz^2^, J Goldstein^3^, D Pouchoulin^4^, JG Laffey^5^, K Harenski^2^

#### ^1^Nalecz Institute of Biocybernetics and Biomedical Engineering, Polish Academy of Sciences, Modeling and Supporting of Internal Functions, Warsaw, Poland; ^2^Baxter Deutschland GmbH, Medical Affairs, Unterscheissheim, Germany; ^3^Baxter World Trade SPRL, Acute Therapies Global, Braine-l´Alleud, Belgium; ^4^Baxter, Gambro Industries, Research and Development, Meyzieu, France; ^5^School of Medicine, NUI Galway, Anesthesia and Intensive Care Medicine, Galway, Ireland

**Introduction:**

The primary outcome findings from the SUPERNOVA trial [1] demonstrated that the use of extracorporeal carbon dioxide reamoval (ECCO_2_R) allows a reduction in tidal volume (TV) to ultraprotective levels (≈4 mL/kg predicted body weight or PBW) during mechanical ventilation in ARDS patients without significant increases in the arterial partial pressure of carbon dioxide (PaCO_2_). Unfortunately, it was not feasible to directly measure ECCO_2_R rates during the trial.

**Methods:**

We used a mathematical model of whole-body oxygen (O_2_) and carbon dioxide (CO_2_) transport and biochemistry [2] to calculate ECCO_2_R rates that permit a fit to the data reported for Hemolung (ALung Technologies) and iLA (Novalung)/Cardiohelp (Getinge) devices in the SUPERNOVA trial [3]. The mathematical model was calibrated under baseline conditions where patients were mechanically ventilated at a TV of 6 mL/kg PBW in the absence of an ECCO_2_R device; the O_2_ consumption rate, CO_2_ production rate and pulmonary shunt fraction were adjusted to match the measured baseline arterial partial pressure of O_2_ and PaCO_2_. Assuming all baseline parameters were fixed, TV was then reduced to 4.1 mL/kg PBW and the mathematical model predicted the ECCO_2_R rate to the change in the PaCO_2_ level.

**Results:**

Model predictions for the devices are shown in Table 1.

**Conclusions:**

These predictions suggest that ECCO_2_R rates for iLA/Cardiohelp devices were approximately twice those for Hemolung devices during the SUPERNOVA trial. These results may be useful to evaluate the expected performance of novel ECCO_2_R devices.

**References:**

1. Combes et al. Intensive Care Med 45:592-600, 2019

2. Leypoldt et al. Artif Organs doi: 10.1111/aor.13601, 2019

3. Combes et al. Thorax 74:1179-1181, 2019

**Table 1 (abstract P087).** Model predicted extracorporeal carbon dioxide removal rates

	Hemolung	Hemolung	iLA/Cardiohelp	iLA/Cardiohelp
Time After TV = 4.1 mL/kg PBW	8 hours	24 hours	8 hours	24 hours
ECCO2R Rate (mL/min)	44	71	131	141

## P088 Efficiency and safety of a system CRRT plus ECCO2R to allow ultra-protective ventilation protocol in patients with acute renal failure

### F Maldarelli^1^, FA Alessandri^1^, RD D´Albo^1^, FP Pugliese^1^, MV Ranieri^2^

#### ^1^Sapienza University of Rome, Department of Anesthesia and Critical Care Medicine, Policlinico Umberto I, Rome, Italy; ^2^Alma Mater Studiorum Università di Bologna, Policlinico Sant´Orsola, Scienze mediche e chirurgiche, Bologna, Italy

**Introduction:**

Despite renal function replacement techniques (CRRT), a patient who develops acute renal failure(AKI) in intensive care unit (ICU) has a mortality rate of 5-80%. This risk is partly due to the adverse effect of AKI on other organs than the kidney. Respiratory complications are frequently associated with the development of AKI. New machines combining CRRT with a carbon dioxide removal membrane (ECCO2R) allows the setting up of an ultra-protective ventilation (4 ml/kg of predicted boby weight (PBW)) to reduce any lung damage from mechanical ventilation (MV). The reduction in tidal volume (Vt) is associated with a decrease in lung damage partly triggered by AKI. We evaluated the efficacy of a combined system CRRT+ECCO_2_R to reduce the Vt to ultraprotective values in patients with acute respiratory failure and AKI.

**Methods:**

Five patients with acute respiratory failure invasively mechanically ventilated for at least 48 that develop AKI needing CRRT were recruited in our ICU. CRRT + ECCO2R was performed with OMNI system® (B.Braun Avitum, Melsungen, Germany). The Vt was progressively reduced to 4 ml/kg/PBW according to a predefined ventilation protocol and therapy was set as follows: CVVHDF, blood flow up to 400 ml/min, dialysis dose 25-35 ml/kg/h, sweep gas:10 L/min. Hemodynamic, respiratory and biochemical clinical parameters were recorded: before the start of treatment (T0) and at the end of each 12-hour interval. Adverse events during treatment have been reported.

**Results:**

Within an hour, patients treated with CRRT+ECCO2R have achieved and maintained an ultra-protective ventilation protocol. The pH and PaCO2 values did not show statistically significant differences from T0 throughout the treatment. In treated patients no complications were recorded.

**Conclusions:**

The combined system CRRT+ECCO2R was safe and effective in allowing a reduction of the Vt up to 4 ml/kg of PBW. Further studies are needed to extend the result of our pilot study.

## P089

**Withdrawn**

## P090 Calculating the optimal ventilator strategy to reduce mechanical power in patients with extracorporeal carbon dioxide removal (ECCO2R)

### KF Bachmann, M Haenggi, D Berger

#### Inselspital Bern, University Hospital, Bern, Switzerland, Department of Intensive Care Medicine, Bern, Switzerland

**Introduction:**

ARDS is a syndrome with high morbidity and mortality. An emerging treatment option is ECCO2R, but the benefit its remains unclear. We assess different degrees of ECCO2R and varying dead space (DS) on ventilator settings in order to minimize mechanical power.

**Methods:**

We calculated mechanical power as

(1) Power=RR*{∆〖Vt〗^2*[1/2*EL+RR*(1+I:E)/(60*I:E)*R]+ ∆Vt*PEEP}

(EL: system elastance, R: airway resistance, PEEP: positive end expiratory pressure, I:E: Inspiratory to expiratory ratio).

We calculated the combination of respiratory rate (RR) and tidal volume (VT) (“optimal RR” and *optimal VT*) leading to minimal applied power for a stable carbon dioxide elimination of 300 ml/min (VCO2) for two scenarios: 1) variation of physiological DS from 10 to 40 % of VT at a fixed rate of ECCOR2. 2) variation of ECCO2R of either 80, 120, 160 or 200 ml/min at a fixed physiological DS of 20%. The alveolar ventilation (VA) necessary to eliminate the VCO2 was calculated as (2) VA= (-VCO2*σ_CO2*R*T*(1+K_c ))/(VCO2/Q-P_vCO2*σ_CO2*R*T*((1+K_c ))/760)

σCO2: CO2 solubility in blood, R: gas constant, T: temperature. PvCO2: venous partial pressure, Kc: function of pH (12.5 for a pH of 7.2), Q: blood flow [5 l/min]).

**Results:**

Increasing DS from 10 to 40% increases the minimal mechanical power from 5.9 to 10.8 J/min, primarily caused by an increase of optimal Vt (495 – 672 ml). Optimal RR was only slightly increased (6.4 – 7.5 /min, Figure 1 Panel A). For varying ECCO2R removal, necessary ventilation ranges from 1.6 to 3.6 L/min. This predicts a minimal power between 5.6 and 10.4 J/min with an unchanged optimal Vt (540 - 543 ml) and an increasing optimal RR (5.4 to 12.3 /min (Figure 1 Panel B)).

**Conclusions:**

In order to minimize mechanical power, increasing shunt or CO2 production should be met with increases in RR while increases in DS should be met with increases in VT. Our results indicate that during ECCO2R, mechanical power and thus risk for lung injury can be minimized with higher VT compared to conservative ventilation strategies.

**Fig. 1 (abstract P090).**
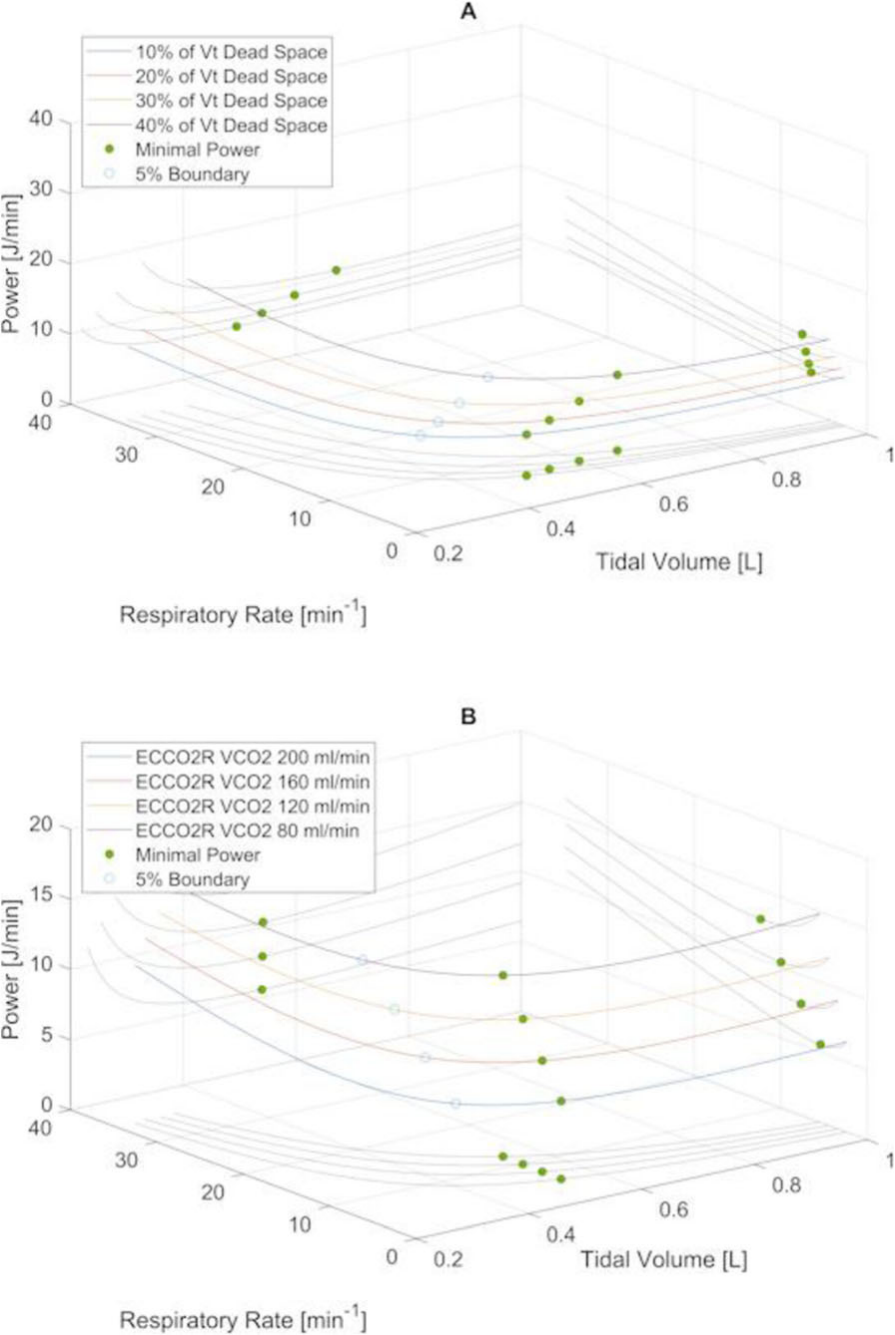
Mechanical power as a function of RR and VT for varying ECCO2R and deadspace

## P091 Validity of empirical estimates of physiological dead space in acute respiratory distress syndrome

### JD Dianti, EG Goligher, AS Slutsky

#### University of Toronto, Interdepartmental Division of Critical Care Medicine, Toronto, Canada

**Introduction:**

Increased physiological dead space fraction (V_D_/V_T_) is a hallmark of the acute respiratory distress syndrome (ARDS) and has been shown to predict ARDS mortality. V_D_/V_T_ is also important in estimating the reduction in tidal volume (V_T_) and driving pressure (∆P) with extracorporeal CO_2_ removal (ECCO_2_R). V_D_/V_T_ can be measured with volumetric capnography but empirical formulae using the patient’s age, weight, height, gender and PaCO_2_ have been proposed to estimate V_D_/V_T_ based on estimates of CO_2_ production (V_CO2_). The accuracy of this approach in critically ill patients, however, is not clear.

**Methods:**

Secondary analysis of a previously published trial [1] in which V_D_/V_T_ and V_CO2_ were measured in ARDS patients. Estimated dead space fraction (V_D,est_/V_T_) was calculated using standard formulae. Agreement between methods was evaluated by Bland-Altman analysis. The predicted change in ∆P with ECCO_2_R was evaluated using both measured and estimated alveolar dead space fraction (V_Dalv_/V_T_).

**Results:**

VD,est/VT was higher than measured VD/VT, with a low correlation between the 2 (R2= 0.21). VCO2 was underestimated by the predicted approach (Table 1), accounting for 57% of the error in estimating VD/VT. The expected reduction in ∆P with ECCO2R using VDalv/VT was in reasonable agreement with the expected reduction using VDalv,est /VT (Bias= -0.7, Level of agreement (LOA) = -1.87 to 0.47). Measured VD/VT was the only variable associated with increased risk of mortality in univariate and multivariate logistic regressions, after adjusting for, age, SOFA score, respiratory system compliance and PaO2/FiO2 (OR 1.9, [95%CI 1.08-3.3], p= 0.02).

**Conclusions:**

V_D_/V_T_ and V_D,est_/V_T_ showed low LOA and should not be used interchangeably. The predicted decrease in ∆P from ECCO_2_R was similar when using both approaches.

**References:**

1. Matthay MA et al. Am J Respir Crit Care 184:561–568, 2011

**Table 1 (abstract P091).** Correlation and agreement between measured and estimated dead space values

	Measured	Estimated	R2	Bias and limits of agreement
VD/VT	0.56 (±0.11)	0.61 (±0.14)	0.21	Bias= 0.05 (LOA= 0.21 – 0.31)
VDalv/VT	0.14 (±0.08)	0.3 (±0.12)	0.11	Bias= 0.17 ( LOA= -0.07 – 0.41)
Anatomical dead space fraction (VDanat/VT)	0.45 (±0.11)	0.34 (±0.06)	0.12	Bias= -0.11 (LOA= -0.33 – 0.11)
VCO2	208 (±66)	178 (±49)	0.39	Bias= -29.7 (LOA= -132 – 73)

## P092 Efficacy of PEEP on cyclic recruitment/derecruitment (R/D) in ARDS patients with different percentage of potentially recruitable lung

### Y Tang, Q Sun, S Liu, X Liu, J Zhang, S Fan, C Pan, L Liu, H Qiu

#### Southeast University, School of Medicine, Zhongda hospital, Department of Critical Care Medicine, Nanjing, China

**Introduction:**

Individualized mechanical ventilation (MV) in patients with ARDS may potentially reduce ventilator induced lung injury (VILI). We explore bedside approach of Electronic Impedance tomography (EIT) to evaluate the percentage of potentially recruitable lung in ARDS patients, and assess the efficacy of PEEPon cyclic R/D in ARDS patients with different recruitability.

**Methods:**

This is a prospective observational study. Patients with moderate and severe ARDS (PaO_2_/FiO_2_<200 mmHg) are enrolled. PEEP detrimental titration method (from 24 to 6 cmH2O) is used to estimate percentage of potentially recruitable lung, calculated by electrical impedance algorithm.ARDS patients are divided to non-recruitable and recruitable group with the median. According to ARDS net FiO_2_/PEEP table, PEEP is set to routine and high PEEP(PEEP_R_/PEEP_H_). Electrical impedance is recorded by EIT. Ventilation distribution is evaluated by ΔZ%, GI, alveolar collapse and hyperinflation percentage, RVDI. Respiratory mechanics parameters and gas exchange parameters are recorded.

**Results:**

20 patients are enrolled and 10 patients are divided to non-recruitable and recruitable group separately. When PEEP is set at PEEP_R_ or PEEP_H_, there is no significant difference in ventilation distribution in dependent lung and independent lung, recruit-able alveolar collapse and hyperinflation percentage between non-recruitable (NR) and recruitable(R) group (P>0.05). Compared with PEEP_R_, ventilation distribution (ΔZ%) increase in dependent lung and decrease in independent lung, recruitable alveolar collapse decrease significantly and hyperinflation significantly increase in NR and R group at PEEP_H_. At PEEP_H_, cyclic R/D in ROI4 is significantly higher in NR group than R group. Compared with PEEP_R_, cyclic R/D doesn’t change globally and in ROI1-4 in NR and R group at PEEP_H_.

**Conclusions:**

High PEEP strategy can improve ventilation distribution, but it can only decrease cyclic recruitment/ derecruitment in the dorsal part of ARDS patients in recruitable group.

## P093 Early neuromuscular blockers in acute respiratory distress syndrome: a systematic review and meta-analysis

### PY Gonzalez noris^1^, I Rivera^2^

#### ^1^ISSSTE General Hospital, Intensive Care Unit, La Paz, Mexico; ^2^ISSSTE General Hospital, Anesthesiology, La Paz, Mexico

**Introduction:**

Acute Respiratory Distress Syndrome (ARDS) is a common condition in critically ill patient. However neuromuscular blockers (NMB) result controvertial in early treatment of ARDS [1]. We ought to search systematically and realize a meta-analysis on the matter.

**Methods:**

An electronic search of randomized clinical trials in adult patient treated with early neuromuscular blockers compared without neuromuscular blockers in ARDS. The primary objective of the analysis was the mortality at 21 to 28 days. Secondary endpoints included mechanical ventilation free days, ICU acquired weakness and barotrauma.

**Results:**

The search obtained 6 studies for the analysis [1-6]. A total of 1557 patients, 785 patients in the NMB group and   772 patients in the control group were included. Comparing those treated with NMB vs. control, the analysis showed relative risk (RR) 0.87 for mortality at 21 to 28 days (95% CI [0.75-0.99], p=0.04), RR. 0.55 for Barotrauma (95% CI[0.35-0.85], p=0.008), RR.1.19 for ICU  acquired weakness (95% CI[0.99-1.42],p=0.07) and mean difference 0.56 for Mechanical ventilation free days (95% CI[-0.46-1.59],p=0.28) (Figure 1).

**Conclusions:**

The early use of neuromuscular blockers in ARDS showed no increase in mortality, but the results should be taken with caution. There was no differences in mechanical ventilation free days. Barotrauma is less with the use of NMB.

**References:**

1. PETAL, N Engl J Med 380:1997-2008, 2019.

2. Gainnier et al. Crit Care Med 32:113-9, 2004.

3. Forel JM et al. Crit Care Med 2006 34:2749-57, 2006

4. Papazian L et al N Engl J Med 2010 363:1107-16., 2010

5. Lyu G. Chin Crit Care Med 263:25-9, 2014

6. Gervillu C et al. Intensive Care Med 43:408-418, 2017.

**Fig. 1 (abstract P093).**
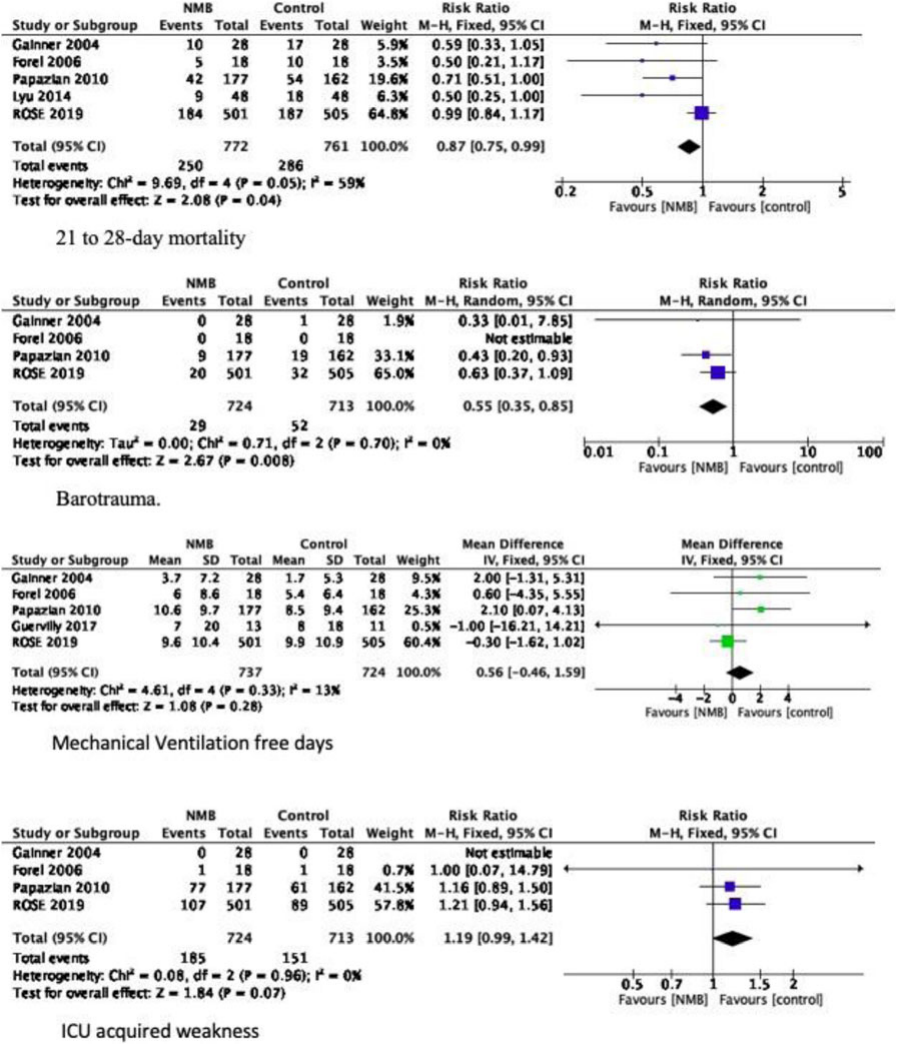
Forest plots

## P094

**Withdrawn**

## P095 The role of ultrasound in the detection of pulmonary infiltrates in critically ill patients with hematologic malignancies and acute respiratory failure

### MM Grgic Medic, NG Gubarev Vrdoljak^,^ OZ Zlopasa, RR Radonic

#### University Hospital Centre Zagreb, Internal Medicine, Zagreb, Croatia

**Introduction:**

Acute respiratory failure (ARF) is one of the major reason for admission of the patients with hematologic malignancies to the intensice care unit (ICU) and pneumonia is among the leading causes of lung infiltrates in these patients. Lung CT is the golden standard in the detection and characterization of pulmonary infiltrates, but it requires the transportation to the radiology units and radiation exposure. Ultrasound can be performed at the bedside without the need for transportation and without irradiation [1, 2]. The aim of this study was to compare the diagnostic accuracy of ultraound in the detection of lung infiltrates, compared to CT scan.

**Methods:**

Patients admitted to the ICU for ARF and suspected pulmonary infection, who required CT for detection and characterization of lung infiltrates, were examind by ultrasound. The assessment of six regions per hemithorax was used; each region was classified for the presence of normal aeration, B lines, indicating interstitial fluid and consolidation +/-air bronchogram. Diagnostic variables were assessed for lung ultrasound, using CT as a reference point.

**Results:**

PUlmonary infiltrates conssitent with pneumonia were confirmed by CT in 24/25 patinets. Ultrasound detected either inhomogenous B lines or onsolidation in 23/24 patients (total sensitivity 96%, specificity 100%, 95% CI 79.65% to 99.90% and 2.50% to 100.00%, PPV. Accuracy of ultrasound was 96.1% (80.36% to 99.90%).

**Conclusions:**

Ultrasound is fairly sensitive in the detection of lung infiltrates in patients with hematologic malignancies.

**References:**

1. Lichtenstein D. Minerva Anestesiol 75:313-7, 2009

2. Chiumello D et al. Crit Care Med 47:1599–1606, 2019

## P096 Right ventricular dysfunction is associated with mortality in patients with pneumonia admitted to intensive care

### M Chotalia^1^, M Bangash^1^, T Matthews^1^, M Kalla^2^, D Parekh^1^, J Patel^1^

#### ^1^University Hospitals Birmingham, NHS Foundation Trust, Critical Care and Anaesthesia, Birmingham, United Kingdom; ^2^University Hospitals Birmingham, NHS Foundation Trust, Birmingham, United Kingdom

**Introduction:**

In patients with pneumonia requiring intensive care (ICU) admission, we hypothesise that abnormal right ventricular (RV) function is associated with an increased 90-day mortality. RV dysfunction in critically ill patients has a well-known association with adverse outcomes [1]. However, its impact on mortality in patients with pneumonia has not been directly studied.

**Methods:**

Patients admitted to the Queen Elizabeth Hospital Birmingham ICU between April 2016 and July 2019 with a diagnosis of pneumonia who had a formal cardiologist TTE were included. Abnormal RV function was defined by either depressed function, dilated size or moderate to severe risk of pulmonary hypertension (pHTN). Abnormal LV function was defined by an LV ejection fraction £ 45% or grade II or more diastolic dysfunction. Patients with a clinical suspicion of pulmonary embolism were excluded. The primary outcome was 90-day mortality. Continuous data is presented as median (IQR). Categorical data is presented as % and analysed using a chi-squared test.

**Results:**

942 patients were admitted to ICU with pneumonia, of which 347 (37%) had a TTE. Patients were 59% male, had a median age of 67 (46-88) and 90-day mortality of 31%. Abnormal RV function was present in 30% (n=103), with 15% depressed, 15% dilated and 14% with moderate to severe risk of pHTN.  RV dysfunction was associated with an increased 90-day mortality compared to normal RV patients (62% vs. 18%, p<0.0001). LV function was abnormal in 25% (n=88) and was not associated with a higher 90-day mortality compared to normal LV patients (38% vs 29%, p = 0.20). RV dysfunction was associated with a higher 90-day mortality than LV dysfunction (62% vs 38%, p = 0.001).

**Conclusions:**

This is one of the first studies to demonstrate that abnormal RV function is associated with an increased mortality in ICU patients with pneumonia. Interestingly, abnormal LV function was not associated with an increased mortality.

**References:**

1. Biteker FS et al. Clin Microbiol Infect 22:1006-e1, 2016.

## P097 Validation of a new method for estimation of the change in pleural pressure using the change in central venous pressure in various clinical scenarios: a pig model study

### M Kyogoku^1^, S Mizuguchi^2^, T Miyasho^3^, Y Endo^4^, Y Inata^1^, K Tachibana^5^, K Yamashita^6^, M Takeuchi^1^

#### ^1^Osaka Women´s and Children´s Hospital, Intensive Care, Osaka, Japan; ^2^Kyushu University, Emergency and Critical Care Center, Fukuoka, Japan; ^3^Rakuno Gakuen University, Laboratory of Animal Biological Responses Department of Veterinary Science School of Veterinary Medicine, Hokkaido, Japan; ^4^Sapporo Night Animal Hospital, Hokkaido, Japan; ^5^Osaka Women´s and Children´s Hospital, Anesthesiology, Osaka, Japan; ^6^Rakuno Gakuen University, Anesthesiology, Hokkaido, Japan

**Introduction:**

We previously reported a simple correction method of estimating pleural pressure (Ppl) by using central venous pressure (CVP) and that it can be used to estimate Ppl and transpulmonary pressure in pediatric patients with respiratory failure. However, it remains unknown that this method can be applied to patients with various levels of chest wall elastance and/or intravascular volume. The objective of this study is to investigate whether our method is accurate in various conditions of chest wall elastance and intravascular volume.

**Methods:**

The study was approved by the Animal Care and Use Committee of Rakuno Gakuen University. Ten anesthetized and paralyzed pigs (43.2 ± 1.8kg) were mechanically ventilated and subjected to lung injury by saline lung lavage. Each pig was subjected to 3 different intravascular volume and 2 different intraabdominal pressures; in each condition, the accuracy of our method was tested. Specifically, airway flow, airway pressure (Paw), esophageal pressure (Pes), and CVP were recorded in each condition, then changes in Pes (ΔPes) and ΔPpl calculated using a corrected ΔCVP (cΔCVP-derived ΔPpl) were compared. cΔCVP-derived ΔPpl was calculated as κ × ΔCVP, where κ was the ratio of the ΔPaw to ΔCVP during the occlusion test.

**Results:**

Means and standard deviations of the two variables that reflect ΔPpl (ΔPes and cΔCVP-derived ΔPpl) in all pigs with all conditions were 6.1 ± 4.1 and 6.4 ± 5.3 cmH_2_O. The Bland-Altman analysis for the agreement between ΔPes and ΔCVP showed a bias of -0.3 cmH_2_O and a precision of 2.9 cmH_2_O in all pigs with all conditions; a bias of 0.1, -0.7, -0.2 cmH_2_O and a precision of 2.2, 3.6, 2.8 cmH_2_O in low, normal and high intravascular volume, respectively; a bias of -0.3, -0.2 cmH_2_O and a precision of 2.8, 3.1 cmH_2_O in low and high intraabdominal pressure, respectively.

**Conclusions:**

Our method can estimate pleural pressure from CVP with a reasonable accuracy regardless of intravascular volume and intraabdominal pressure (Fig. 1).

**Fig. 1 (abstract P097).**
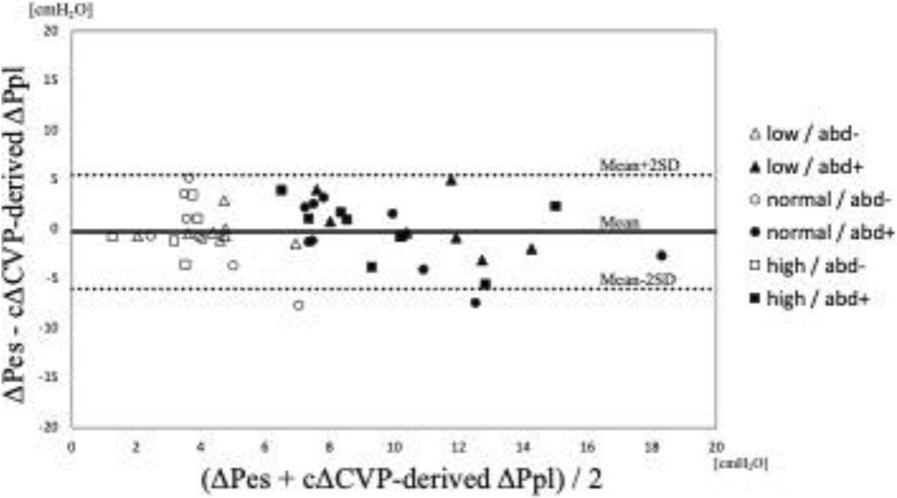
The Bland–Altman analysis for the agreement between ΔPes and cΔCVP-derived ΔPpl in various conditions. low: low intravascular volume, normal: normal intravascular volume, high: high intravascular volume, abd-: without an abdominal compression band, abd+: with an abdominal compression band

## P098 The role of ultrasound in monitoring diaphragm activity in critically ill patients

### F Righetti^1^, E Colombaroli^2^

#### ^1^Intensive Care Unit, Fracastoro Hospital, Emergency Department, San Bonifacio, Verona, Italy; ^2^Intensive Care Unit, Fracastoro Hospital, San Bonifacio, Verona, Italy

**Introduction:**

The activity and functionality of the diaphragm are difficult to measure in patients ventilated in intensive care. Ultrasound can be a useful tool for monitoring diaphragm muscle activity during different ventilation modes. Few data currently exist on diaphragm muscle activity in critically ventilated patients [1]. Our goal is to evaluate the respiratory muscular work of the diaphragm with different settings of the respirator by means of an ultrasound scan.

**Methods:**

The ultrasound assessments of the diaphragm were performed with a 10MHz linear probe at the apposition zone. We measured the thickening of the diaphragm with the respiratory acts, through the thickening fraction (Thickening Fraction, TF), defined as:TF = (Tdimax - Tdimin / Tdi min)% Tdimax: Diaphragm thickness at the end of inspiration (maximum thickness) Tdimin: Diaphragm thickness at the end of expiration (minimum thickness). Ventilatory support was divided into 4 classes: 1 - spontaneous breathing (SB) or Continous Positive Airway Pressure (CPAP); 2 - Pressure Support Ventilation (PSV) with low pressure support (5-12cmH2O); 3- PSV with high pressure support (> 12 cmH2O); 4 - Controlled Mechanical Ventilation (CMV).

**Results:**

A total of 223 assessments were performed in 70 patients. The evaluations were all possible at the right hemidiaphragm, while on the left they were not possible in 7% of the cases. The median TF (IQ range) of the 4 ventilation classes was respectively: 42% (25-62%) in SB / CPAP; 26% (17-31%) in low-PSV; 17% (9-22%) in high PSV; and 5% (2-13%) in CMV. The Kruskal-Wallis test confirms a significant difference between the groups (p <0.0001).

**Conclusions:**

The ultrasound of the diaphragm can be a valid tool for monitoring respiratory muscle activity during mechanical ventilation.

**References:**

1. Shahshahani A et al. Sensors (Basel). 18 :E2617, 2018

## P099 Does high flow nasal oxygen therapy reduce extubation failure in critically ill patients?

### A Mcguire, R Sundaram

#### Intensive Care Unit, Royal Alexandra Hospital, Royal Alexandra Hospital, Paisley, United Kingdom

**Introduction:**

Extubation failure is defined as reintubation after 48 hours of extubation in mechanically ventilated critically ill patients. It is associated with morbidity and mortality. The aim of our study was to assess reintubation rates in a busy district general hospital and evaluate the impact of high flow nasal oxygen therapy (HFNO) on reintubation rates.

**Methods:**

We performed a retrospective observational study looking at patients admitted to our 7 bedded Level 3 critical care unit (370 patients a year) for a period of 5 years between 1^st^November 2014 and 31^st^October 2019. We included patients over 16 years of age who were mechanically ventilated and length of stay was greater than 48 hours. Exclusions were age < 16 years, tracheostomy and patients requiring ventilation for < 48 hours. Data was collected from Ward Watcher, a SICSAG database and electronic patient records.

**Results:**

There was 1735 patients and 1413 patients were included (802 male 611 female). 1076 (76.2%) of patients required ventilation for over 48 hours, 66.7% (718/1065) of those were extubated (Table 1). 25.6% (184/718) of patients required reintubation, despite 54.3% (100/184) receiving HFNO.  Of the patients successfully extubated 41.4% (221/534) received HFNO.

**Conclusions:**

Our study failed to show any impact of HFNO on reducing extubation failure. Further work is needed to develop a standardized approach to weaning and to consider routine application of non-invasive ventilation to reduce reintubation rates [1].

**References:**

1. Hernández G, et al. JAMA 316:1565-74, 2016.

**Table 1 (abstract P099).** Extubation/reintubation rates plus use of nasal high flow in patients ventilated for >48 hours

	Number	Percentage
Ventilated >48 hours	1076	76.2%
Extubated	718	66.7%
Reintubated within 24 hours	126	17.6%
Reintubated overall	184	25.6%
Reintubated HFNO used	100	54.3%
Remained extubated	534	74.4%
Remained extubated HFNO used	221	41.4%

## P100 Introduction new practice of endotracheal tube holder to intubated patient to improve oral care in intensive care unit

### YT Ho^1^, WT Yeung^2^, OK Lee^2^, PO Lei^2^, MT Lai^2^, SC Ho^2^, EH Chan^2^, LY Law^2^, KY Ho^2^, HC Yuen^2^

#### ^1^Ruttonjee & Tang Shiu Kee Hospitals, Cardiac & Intensive Care Unit, Hong Kong; ^2^Ruttonjee & Tang Shiu Kee Hospitals, Hong Kong

**Introduction:**

Oral endotracheal intubation is common to critically ill patients in intensive care unit. Oral care for an intubated patient is important to maintain the moisture of oral mucosa. Also, the securement method of oral endotracheal tube developed from cloth tape to commercial tube holder.

**Methods:**

Training PowerPoint and video for microteaching was prepared to train up 30 ICU nurses to perform the new practice. Demonstration and re-demonstration was arranged to assess skills of every nurse. Afterwards, each nurse answered a quiz to evaluate the understanding of OETTH and its special techniques in application. Questionnaire was designed to collect the feedback from all nurses too.

**Results:**

The result showed there was 21 nurses (72%) out of 30 nurses achieved full marks in the post-quiz which demonstrated their full understanding of the use of oral ETT holder and its nursing care. About the feedback from nurse, 72% of nurses claimed that they were confident in using the new OETTH in clinical setting after training. 96% of nurses agreed in time-saving of nursing care routine with the use of an OETTH. However, only 56% of nurses agreed that the OETTH is effective in prevention of oral mucosa injuries and another 24% of nursing staff disagreed on its function in improving the patient’s oral care.

**Conclusions:**

In conclusion, some of the nurses did not agree the prevention of oral mucosa injuries by the new securement method with OETTH while some nurses welcomed the new OETTH as more easy and effective in oral care to intubated patients.

## P101 Execution of percutaneous dilatational tracheostomy using the standard laryngeal mask airway for ventilation: a prospective survey study

### G Gagliardi^1^, V Gagliardi^2^, C Chiani^3^, G Laccania^4^, F Michielan^3^

#### ^1^AULSS 5 - Veneto, Anesthesia and Intensive Care, Adria, Italy; ^2^AULSS 5 - Veneto, University of Padua, Adria, Italy; ^3^AULSS 5 - Veneto, Anaesthesia and Intensive Care, Adria, Italy; ^4^AULSS 6 - Veneto, Anaesthesia and Intensive Care, Padua, Italy

**Introduction:**

We fulfilled a survey study dealing with bronchoscope-guided percutaneous dilatational tracheostomies (PDT), using the classic laryngeal mask airway (LMA) for the airway management [1]. The aim was to verify the safety and the effectiveness of the aforementioned procedure

**Methods:**

We performed an observational prospective survey study enrolling 150 patients hospitalized in the Intensive Care Unit. Before performing the tracheostomy, the endotracheal tube has been replaced by the laryngeal mask airway. Arterial blood gases, ventilation pressures and tidal volumes have been monitored, registered and compared.

**Results:**

The median peak inspiratory pressure has been detected stable in all patients. Furthermore, during the ventilation with the laryngeal mask, the tidal inspiratory and expiratory volume difference observed between before and after the bronchoscope positioning, has shown a statistically significant variation. Finally, in all cases ETCO_2_, SpO_2._, PaO_2,_ and blood pH values persisted within the normal range.

**Conclusions:**

The standard LMA provides for a reliable airway management and allows an effective ventilation while performing the PDT. Once positioned in the supraglottic zone, the LMA does not need to be moved throughout all the PDT performance, avoiding risks of displacement, glottic harm and airway device damage, and permitting an easy handling of the bronchoscope, which gives an appropriated visualization of the trachea and a more efficient aspiration. In consequence to the large internal diameter of the LMA tube, Ppeak has continued to be stable in all patients, providing for minor resistance and inspiratory work. Eventually, no late complications, such as tracheal stenosis and infections, have occurred.

**References:**

1. Strametz R et al. Cochrane Database of Systematic Reviews 11: CD009901, 2018

## P102 Dilatative percutaneous tracheostomy vs surgical tracheostomy: what late complications?

### F Righetti^1^, E Colombaroli^2^

#### ^1^Intensive Care Unit, Fracastoro Hospital, Emergency Department, San Bonifacio, Verona, Italy; ^2^Intensive Care Unit, Fracastoro Hospital, San Bonifacio, Verona, Italy

**Introduction:**

There are many possible post-tracheostomy late complications with both percutaneous and surgical techniques (peristomal bleeding, granulomas, tracheomalacia, tracheal stenosis) [1]. The purpose of our retrospective study is to define the prevalence of these complications by comparing the two techniques.

**Methods:**

60 tracheostomized adult patients (pcs) were divided into 2 groups: group A (percutaneous dilatation technique according to Ciaglia) group B (surgical technique), followed for 4 years (2016-2019), cuffed cannulae, cannula change every 60 days at home, tracheofibroscopic control in Intensive Care Unit (ICU). The results were expressed as a percentage of prevalence and subsequently comparing the two groups.

**Results:**

30 pcs in group A and 30 pcs in group B. 1440 home tracheostomy cannula replacements, 240 tracheofibroscopy performed in ICU. In group A: 56% had peristomal bleeding, 36% external peristomal granulomas, 16% intra tracheal granulomas, 6% tracheomalacia. In group B: 23% had peristomal bleeding, 16% external peristomal granulomas, 33% intra tracheal granulomas, 13% tracheomalacia, 3% tracheal stenosis.

**Conclusions:**

Peristomal bleeding resolved independently. The external granulomas were treated with silver nitrate for a month. Intra-tracheal granulomas were treated with laser therapy. Tracheomalacia and tracheal stenosis resolved by placing a reinforced cannula with variable flange under bronchoscopic guidance. Comparing the two groups it emerged that the percutaneous dilatative technique has fewer serious late complications (intra tracheal granulomas, tracheomalacia, tracheal stenosis) than the surgical technique.

**References:**

1. Cipriano A et al. Int J Crit Illn Inj Sci 5:179–188, 2015

## P103 Is there benefit to delaying tracheostomy?

### FS Zimmerman, PD Levin

#### Shaare Zedek Medical Center, Intensive Care Unit, Jerusalem, Israel

**Introduction:**

Tracheostomies are the most common surgical procedure performed on critically ill patients. Randomized control trials comparing tracheostomy timing in intensive care patients have been equivocal. In order to perform non-urgent tracheostomy in our ICU, consent is required from the patient or a formal guardian appointed ad hoc by the courts. Since tracheostomies are practically the only elective surgery performed in the critically ill, ICU requested guardianship almost always indicates a clinical decision to perform tracheostomy. As appointing a guardian and arranging a tracheostomy takes about a week, the decision to appoint a guardian offers a unique "intention to treat" opportunity to evaluate outcomes in patients for whom tracheostomy is planned.

**Methods:**

We performed a retrospective analysis over 3 years on patients for whom guardianship was sought excluding those requiring urgent tracheostomy and those with a do-not-resuscitate order. Patients were divided according to outcome (tracheostomy, extubation or death prior to tracheostomy) and compared.

**Results:**

Guardianship was sought for 233 ventilated patients. A decision to withhold tracheostomy was made for 13 patients, who were excluded, leaving 220 patients for analysis. Tracheostomy was performed for 131/220 (60%) patients, 62/220 (28%) were extubated and 27/220 (12%) died while waiting for tracheostomy (from non-airway related reasons). Tracheostomy was performed on mean ventilation day 16±1. Comparing extubated patients to those who had tracheostomy (Table) shows similar demographics, but significantly lower mortality and hospital length of stay.

**Conclusions:**

A significant proportion of patients initially planned for tracheostomy were successfully extubated. Despite demographic similarities, mortality in this group was significantly lower than for patients undergoing tracheostomy. For a selected subgroup of possibly difficult to characterize patients, delaying tracheostomy may be beneficial.

**Table 1 (abstract P103).** Tracheostomy demographics and outcomes

	Variable	extubated n=62 (%)	tracheostomy n=131 (%)	p-value
Demographics	Male	45 (72.6)	78 (59.5)	0.108
	Age - years ± CI	63±8	63±6	0.915
	Surgical patient	28 (45.2)	50 (38.2)	0.432
	APACHE II score ± CI	22±1	21±1	0.425
Outcomes	In-hospital mortality	9 (14.5)	37 (28.2)	0.046
	ICU LOS, h, median (IQR)	422 (288-518)	383 (204-601)	0.152
	hospital LOS, h, median (IQR)	857 (628-1180)	1236 (857-1633)	0.001

## P104

**Withdrawn**

## P105 Retrospective audit of tracheostomy insertion in a training intensive care unit in Singapore

### D Bruce-Hickman^1^, G Aquino Vergel De Dios^2^, P A Dela Cruz^2^, F Khan^3^

#### ^1^Ng Teng Fong General Hospital, Singapore; ^2^Ng Teng Fong General Hospital, Respiratory Therapy, Singapore; ^3^Ng Teng Fong General Hospital, Intensive Care Medicine, Singapore

**Introduction:**

Percutaneous tracheostomy insertion (PTI) is a core competency for ICU trainees. Standards for insertion are available from Intensive Care Societies and eminence-based groups [1]. Standard of PTI has not previously been analyzed in Singaporean public ICUs.

**Methods:**

Retrospective analysis was performed of PTIs from September 2015 to 2018. Audit proforma was based on best practice guidelines. PTIs for acute airway scenarios were excluded. Ethical approval was granted via local processes for audit and quality improvement work.

**Results:**

151 tracheostomized patients were admitted in the study period. After exclusion, 109 patients were audited. 91 patients had a PTI as a bedside procedure by the ICU team, 17 patients had a tracheostomy performed in theatre; data was missing for 1 case. PTI was performed at a mean of 11 days from admission. Trial of extubation occurred in 44% of cases.  Ventilator settings were mostly appropriate (Figure 1). PTIs were analysed by speciality and by outcome. Complications occurred in 6 cases (incidence 6.5%). There were 3 cases of subcutaenous emphysema, 1 pneumothorax (occuring D6 post procedure) and 1 case each of stoma and suture site infection. There was 1 unplanned cannula change within 7 days of insertion. 24% of cases had cuff inflated on discharge from ICU. Handover of care was suboptimal; follow up care plans were documented in 18% of cases. A supervising consultant was present for all PTIs. There was a trend of increased insertion by consultant and increased reliance on theatre, with corresponding decrease in the number inserted by trainees.

**Conclusions:**

PTI in our training ICU appears safe with low incidence of complications and good senior support for tracheostomy insertion. Emphasis must continue on training junior intensivists in PTI. Transition of care beyond ICU requires further work where currently there is suboptimal handover of care and safety netting for non-ICU colleagues.

**References:**

1. McGrath B. The National Tracheostomy Safety Project Manual. London. BMJ Books; 2014

**Fig. 1 (abstract P105).**
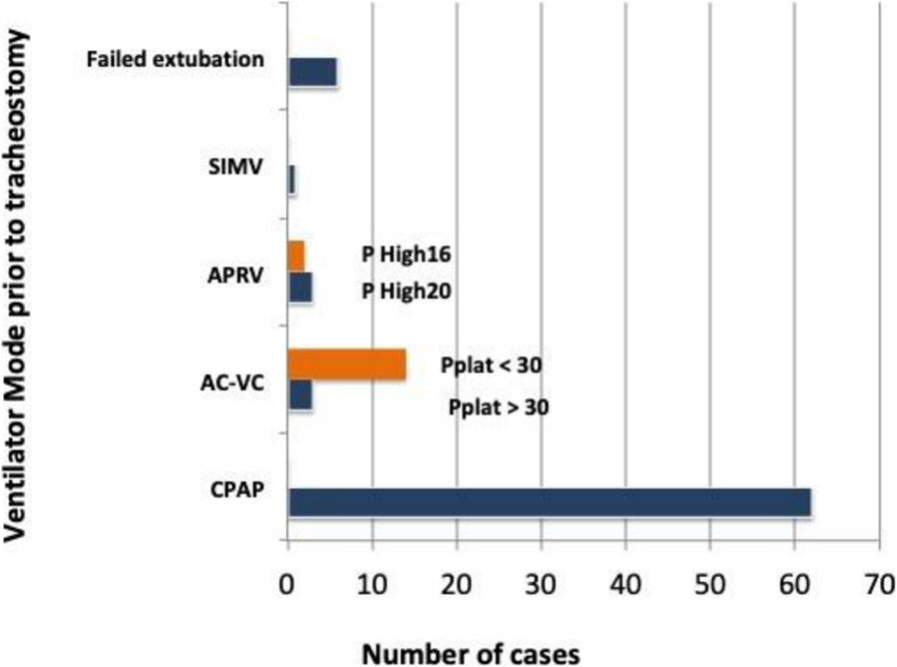
Ventilator Settings prior to PTI

## P106 Impact of bronchoscopically graded inhalation injury on outcomes in critically ill burns patients: 12-year experience at a regional burns centre

### WN Charles^1^, K Matwala^1^, A Dutt^1^, S Mandalia^2^, D Collins^3^, S Singh^4^

#### ^1^Imperial College London, Department of Surgery and Cancer, London, United Kingdom; ^2^Chelsea and Westminster Hospital NHS Foundation Trust, Department of Research and Development, London, United Kingdom; ^3^Chelsea and Westminster Hospital NHS Foundation Trust, Department of Plastic and Reconstructive Surgery, London, United Kingdom; ^4^Chelsea and Westminster Hospital NHS Foundation Trust and Royal Brompton and Harefield NHS Foundation Trust, Magill Department of Anaesthesia, Intensive Care and Pain Management, London, United Kingdom

**Introduction:**

Burns inhalation injury (BI) is suggested to significantly impact burns mortality. Yet, there is inconsistent evidence of how this is influenced by its bronchoscopically graded severity. This study evaluated the effect of different bronchoscopic BI grades on mortality.

**Methods:**

A retrospective, single-centre cohort study of all patients admitted to the London Burns centre intensive care unit (BICU) over 12 years. Demographic data, burn and BI characteristics, and ICU-related parameters were collected. A bronchoscopy expert retrospectively graded BI using bronchoscopy images and reports. The primary outcome was mortality. Secondary outcomes were hospital and ICU length of stay measures. The impact of pneumonia after 48 hours was determined. Univariate and multivariable Cox’s proportional hazards regression analyses informed factors predicting mortality.

**Results:**

BI was diagnosed in 84 of 231 (36.4%) critically ill burns patients. Median (IQR) total body surface area burned (TBSA) was 20% (10 to 40). Overall, mortality risk was significantly increased by BI (45.2% vs 23.8%, p<0.001) and pneumonia (27.2% vs 11.3%, p<0.001). Median survival time with BI was significantly lower than without BI (94 vs 131 days, Log-rank test p=0.002). More severe grades of BI (2/3) had significantly increased risk of mortality (adjusted HR=2.14, 95%CI: 1.12-4.09, p=0.022) compared with lower grades (1) (adjusted HR=0.58, 95%CI: 0.18-1.86, p=0.363) (Fig. 1). Facial burns (adjusted HR=3.13, 95%CI: 1.69-5.79, p<0.001), higher TBSA (adjusted HR=1.05, 95%CI: 1.04-1.06, p<0.001) and older age (adjusted HR=1.04, 95%CI: 1.02-1.07, p<0.001) also independently predicted mortality; though, pneumonia did not.

**Conclusions:**

Moderate to severe bronchoscopically graded BI significantly increased mortality in critically ill burns patients. Facial burns, higher TBSA and age also independently predicted mortality. Further studies on the diagnostic accuracy, reliability and impact of bronchoscopically grading BI are recommended.

**Fig. 1 (abstract P106).**
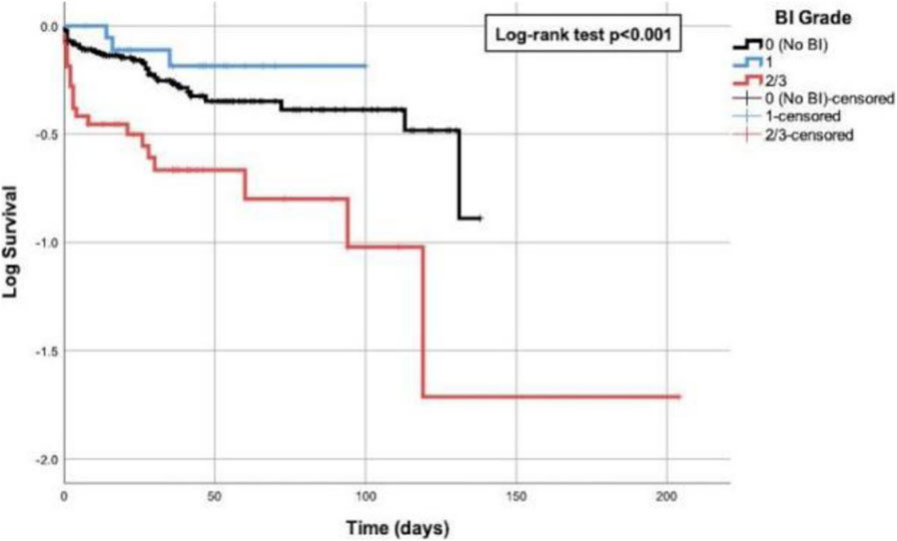
Kaplan-Meier log survival plots of the different grades of Burns Inhalation injury (BI)

## P107 A quality improvement project to prevent hyperoxia in a developing world intensive care unit

### J Harrington, D Hall, TY Wang, M Murali, S Mwansa

#### University Teaching Hospital, Anaesthesia, Lusaka, Zambia

**Introduction:**

Supplemental oxygen administration is ubiquitous in the critical care environment, yet evidence is mounting for the deleterious effects of hyperoxia [1]. Concerns over the adverse effects from hypoxaemia often exceed those of hyperoxaemia in developing world settings, and inconsistent availability of blood gas monitoring may limit judicious oxygen titration. The aim of this project was to audit oxygen delivery practice and introduce QI measures to avoid excess oxygen delivery in a tertiary ICU in Lusaka, Zambia.

**Methods:**

A prospective snapshot of ventilatory parameters were recorded for critically ill patients over a 5-week period, including positive end expiratory pressure (PEEP), FiO_2_, and time-course SpO_2_. Systematic education was provided through group and one to one tutorials to empower nursing and medical staff to titrate oxygen safely and appropriately. Repeat data collection was then performed over 4 weeks.

**Results:**

Initially 18/30 patients (60%) were over-oxygenated, as defined by FiO2 >0.5 and SpO_2_ consistently >95%. 12/18 patients with an FiO_2_ of >0.5 had PEEP ≤ 5cm (67%). No patient had a PaO_2_ recorded in the past 24 hours. Education was provided as well as implementation of unit protocols above all patient beds documenting a stepwise approach to titration PEEP and FiO_2_. Post intervention fewer patients were over-oxygenated: 7/21 (33%) had FiO2 >0.5 and SpO_2_ consistently >95%, and 7/18 with an FiO_2_ >0.5 (39%) had a PEEP ≤ 5cm. In addition, 7/21 (33.3%) had a PaO_2_ recorded within 24 hours.

**Conclusions:**

This QI project has shown that nurse engagement and systematic education to titrate FiO2 and PEEP can be achieved in a resource poor setting and may decrease the incidence of hyperoxia in critically ill patients. Availability of blood gas monitoring and knowledge of interpretation was a major barrier to oxygen titration

**References:**

1. Sinclair SE et al. Crit Care Med 32:2496–501, 2004

## P108 Epidemiology and factors associated with tracheal intubation in pediatric burned population

### S Lebrun, N Akrout, N Louvet, I Constant

#### Hopital Armand Trousseau (APHP), Anesthesie-Réanimation, Paris, France

**Introduction:**

Tracheal intubation (TI) in adult burn patients might be unnecessary in 30 to 40% of cases [1, 2]. In pediatric burn patients, there is little data on both the rate of TI and the rate of early extubation [3]. It has been common practice for a child with a facial burn and/or a suspected airway injury to be intubated early due to the risk of losing airway patency. However this risk should be mitigated against the potential risks of TI and mechanical ventilation in children. Therefore the aim of this study was to describe the airway status of child burn victims taken in charge of in our pediatric burn intensive care unit. Focused on patients arriving with TI, we investigated the rate of early extubation. In addition we compared non intubated patients with those with prolonged TI.

**Methods:**

This retrospective study described a cohort of 1520 patients hospitalized between 2010 and 2018. Data was retrospectively recorded from the patient’s paper clinical chart.

**Results:**

The mean age of our patients was 2.8 ±3.1 years [mean±sd] with an average burn area of 14±11%. 86% had scald burns and 45% had facial burns. 4% of the children were admitted in the burn ICU with TI. For 36% of them, tracheal tube was removed within the first 48 hours after admission. The probability of prolonged TI increased independently with the burned skin area (BSA) (p <0.0001), the presence of facial burns (p = 0.001), and in case of flame burns (p = 0.007) (Figure 1). Among patients with more than 70% BSA, 85% were intubated more than 48h. Among patients with less than 20% BSA, 0.5% were intubated more than 48h.

**Conclusions:**

According to our retrospective data, it seems appropriate to intubate children with 70% and more BSA, while for patient with less than 70% BSA, it might be relevant to seek guidance from physician of the nearest Burn Center. Under 20% BSA, TI seems rarely required.

**References:**

1. Romanowski KS et al. J Burn Care Res 37:e409-14, 2016

2. Cai AR et al. J Burn Care Res 38:e23-e29, 2017

3. Mosier MJ et al. J Burn Care Res 37:e1-6, 2016

**Fig. 1 (abstract P108).**
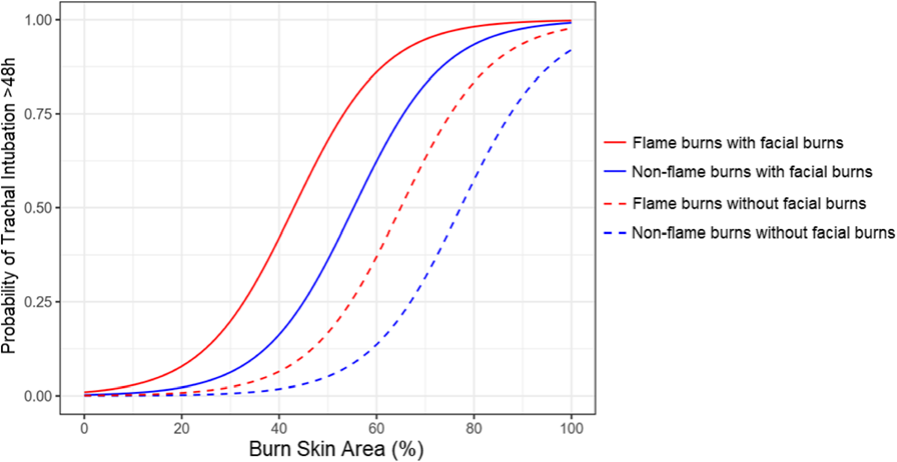
Probability of tracheal intubation depending on burn skin area, facial burns and flame burns (multivariate analysis)

## P109 An analysis of the predictive applicability of initial blood gas parameters for the need for intubation and the presence of inhalation injury in patients with suspected inhalation injury

### C Pirrone^1^, M Chotalia^2^, T Mangham^1^, R Mullhi^1^, K England^1^, T Torlinski^1^

#### ^1^University Hospitals Birmingham, NHS Foundation Trust, Birmingham, United Kingdom; ^2^University Hospitals Birmingham, NHS Foundation Trust, Critical Care and Anaesthesia, Birmingham, United Kingdom

**Introduction:**

We hypothesise that initial blood gas parameters have a good predictive applicability in detecting the need for intubation and the presence of inhalation injury in patients with suspected inhalation injury. To the best of our knowledge, this has not been directly studied in the literature.

**Methods:**

Patients with suspected inhalation injury admitted to the ICU at Queen Elizabeth Hospital, Birmingham between April 2016 and May 2019 were included. The initial blood gas parameters analysed were PaO_2_ (kPa), PaCO_2_ (kPa), pH, carbon monoxide level (COHb; %) and PaO_2_/FiO_2_ (PF) ratio. Receiver operator characteristics (ROC) for these parameters were plotted against the need for intubation for more than 48 hours and the presence of inhalation injury as detected by bronchoscopy and laryngoscopy. Area under the curve (AUC) for each parameter was calculated.

**Results:**

85 patients were admitted with suspected inhalation injury to the ICU. 68% were intubated for more than 48 hours. Of patients who were intubated, 69% had inhalation injury as indicated by bronchoscopy or laryngoscopy. Table 1 outlines the AUC for initial blood gas parameters in detecting the need for intubation for more than 48 hours and the presence of inhalation injury. pH was the parameter with the most prominent AUC, with reverse correlation indicating fair accuracy.  No clear inflection point was identified, although all patients with pH < 7.25 required intubation and had inhalation injury. PaCO_2_ had a fair predictive applicability in detecting the need for intubation. PF ratio, PaO_2_ and COHb had poor accuracy.

**Conclusions:**

Initial blood gas parameters had a broadly poor predictive applicability for the need for intubation and the presence of inhalation injury in patients with suspected inhalation injury. Severe acidosis (pH <7.25) was the most useful blood gas parameter. Clinicians should be cautious in using blood gas parameters alone to inform intubation decisions.

**Table 1 (abstract P109).** AUC for initial blood gas parameters in predicting the need for intubation for more than 48 hours and the presence of inhalation injury

	Need for intubation for more than 48 hours	Presence of inhalation injury
pH	0.203	0.241
PaCO2	0.715	0.691
PaO2	0.458	0.408
PF ratio	0.437	0.467
COHb	0.536	0.604

## P110 Lung protection during lung cancer surgery by non-intubated spontaneous breathing - a case series

### P Friederich^1^, F Fuchsgruber^2^, A Hiebinger^3^, H Angerer^2^, J Bodner^3^

#### ^1^München Klinik Bogenhausen, Technische Universität München, Anesthesiology, Critical Care, München, Germany; ^2^München Klinik Bogenhausen, Technische Universität München, Anaesthesiology, Critical Care, München, Germany; ^3^München Klinik Bogenhausen, Technische Universität München, Thoracic Surgery, München, Germany

**Introduction:**

Lung cancer surgery is associated with a high rate of pulmonary complications including ARDS and mandates lung protective ventilation strategies [1,2]. Such strategies include non-intubated video assisted thoracic surgery (NiVATS) with spontaneous breathing [3]. Currently neither data on respirator settings nor on gas exchange have been reported for applying the latter. This data constitutes a prerequisite for meaningful evaluating the respiratory consequences of non-intubated spontaneous breathing during lung cancer surgery. The aim of this case series was for the first time providing such data from lung cancer surgery including pneumonectomy.

**Methods:**

During a 12 month period 32 patients without contraindications [3] scheduled for video assisted thoracic surgery (VATS) for non-anatomical and anatomical lung resection including one pneumonectomy (Px) were offered non-intubated spontaneous breathing. All patients gave informed written consent to the procedure as well as for analysis and publication of data. Anaesthetic management included target controlled infusion of propofol and remifentanil, laryngeal mask airway, and pressure support ventilation. Data from 2 patients were excluded due to change of procedure for reasons of surgical access. Data are given as median and range (Figure 1).

**Results:**

All patients breathed spontaneously during the operation. It allowed for low driving pressures, lung protective tidal volumes, as well as adequate oxygenation also in pneumonectomy. It may result in hypercapnia. Cardiorespiratory complications were not observed.

**Conclusions:**

Spontaneous breathing during lung cancer surgery is feasible and it may be applied during pneumonectomy. Whether it decreases the rate of pulmonary complications in patients undergoing lung cancer surgery deserves further study.

**References:**

1. Park et al. Anesthesiology 130: 385-393, 2019

2. Marret et al. Eur J Anaesthesiol 35: 272-735, 2018

3. Moon et al. J Thorac Dis 10: 3490-3489, 2018

**Fig. 1 (abstract P110).**
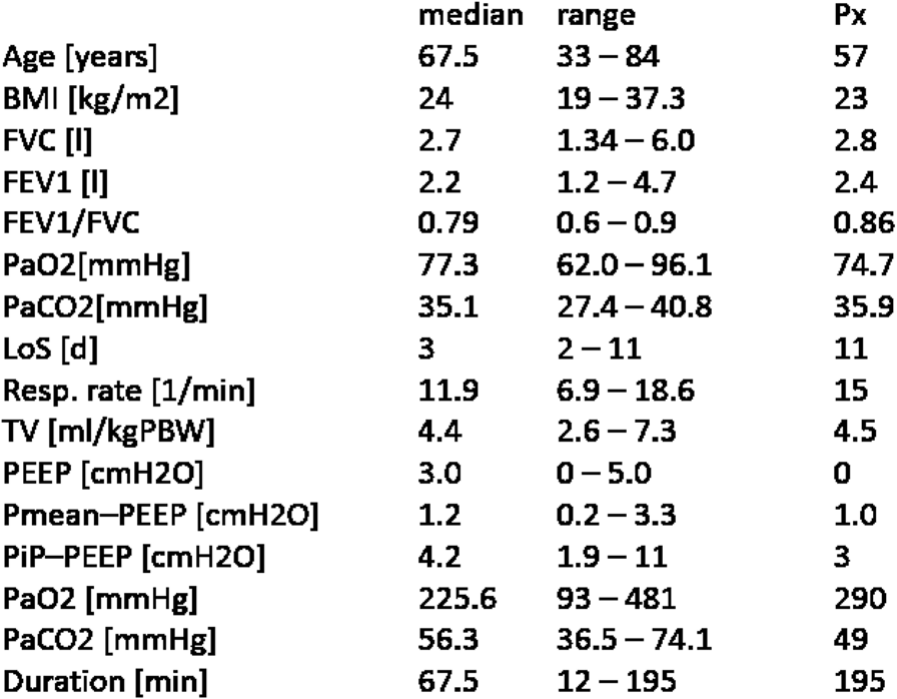
Demographics, results of preoperative lung testing, length of hospital stay (LoS), respirator settings, results of intraoperative blood gas analysis, and duration of surgery. Px = Pneumonectomy

## P111 The effect of early cuff deflation on weaning in tracheostomized patients requiring ventilatory support on the intensive care unit

### C Carroll^1^, L Poole^2^, C Lamont^2^, I Welters^2^

#### ^1^University of Liverpool, Liverpool, United Kingdom; ^2^Liverpool University Hospitals NHS Foundation Trust, Intensive Care Unit, Liverpool, United Kingdom

**Introduction:**

Prolonged cuff inflation in tracheostomized patients inhibits phonation, contributes to dysphagia, causes psycho-emotional distress and results in diaphragmatic weakness. It also increases the risk of ventilation acquired pneumonia and ventilator induced lung injury. The aims of this study were to determine whether cuff deflation trials had an effect on the length of the weaning period, length of ITU stay and total tracheostomy days.

**Methods:**

A retrospective audit of all tracheostomized patients admitted to ITU at Liverpool University Hospitals NHS Foundation Trust from 8 January 2017 – 30 August 2019 were reviewed. The exclusion criteria included long term tracheostomies, patients who had a tracheostomy at the point of admission, patients who died or were transferred prior to decannulation and patients with insufficient clinical documentation. Trials of early cuff deflation were introduced to ITU on 26 April 2018. Patients were divided into two groups: the ‘pre-trial group’, consisting of patients who received a tracheostomy prior to 26 April 2018, and the ‘post-trial group’ who received a tracheostomy after 26 April 2018.

**Results:**

183 tracheostomized patients were identified with 70 patients meeting the inclusion criteria. There were 27 patients in the pre-trial group and 43 in the post-trial group. The mean length of ITU stay (43.33 pre-trial vs 39.21 post-trial, p=0.242), the number of total tracheostomy days (31.99 vs 27, p=0.313) and the mean number of days between first cuff deflation and decannulation (21.77 vs 19.11, p=0.385) was not significantly different between groups. The protocol allowed significantly earlier cuff deflation (12.1 vs 4.8 days).

**Conclusions:**

We present early data that early trials of cuff deflation within 48 hours of tracheostomy insertion can be achieved using a standardized protocol. Its impact on length of stay, duration of ventilation and patient-centered outcomes needs to be investigated in larger multi-centre trials.

## P112 Preventing underinflation of the endotracheal tube cuff with a portable elastomeric device. A randomized controlled study

### JE Dauvergne^1^, AL Geffray^2^, K Asehnoune^2^, B Rozec^1^, K Lakhal^1^

#### ^1^Hopital Laënnec - CHU de Nantes, Service d´anesthésie-réanimation, Nantes, France; ^2^Hotel-Dieu - CHU de Nantes, Service d´anesthésie-réanimation, Nantes, France

**Introduction:**

The management of the endotracheal tube cuff pressure (P_cuff_) is routine practice for critical care nursing staff. Underinflation could lead to ventilator-associated pneumonia [1] whereas overinflation exposes to tracheal damage [2]. Multi-daily check and adjustment is recommended to ensure that P_cuff_ lies between 20 and 30 cmH_2_O [3]. To automate this task some devices exist but may be inconvenient, bulky and/or ineffective. Their use is not supported by guidelines. A portable elastomeric device could be appealing for P_cuff_ automated regulation. This prospective randomized controlled study tested whether the Tracoe Smart Cuff Manager^TM^ reduced the rate of patients undergoing ≥1 episode of underinflation (P_cuff_<20 cmH_2_O), as compared with routine manual P_cuff_ adjustment.

**Methods:**

Monocentric, randomized controlled study. Patients with acute brain injury and receiving mechanical ventilation were prospectively allocated to one of the two arms: manual reading and adjustment of P_cuff_ at least every 8h (routine care) or adjunction of the Smart Cuff Manager^TM^ (intervention). This study was approuved by an institutional review board.

**Results:**

Among 60 randomized patients (routine care in 32, Smart Cuff Manager^TM^ in 28), 506 measurements were performed in 48h. With routine care, a higher rate of patients experienced at least one episode of underinflation (62.5 vs. 17.8%;p<0.001). Episodes of underinflation episodes (15% vs. 2%;p<0.001) and manual adjustments (77% vs. 56%;p<0.001) were more frequent with routine care. For overinflation, there was no between-arms difference (p>0.99).

**Conclusions:**

The adjunction of continuous P_cuff_ control with the Tracoe Smart Cuff Manager^TM^ reduced the incidence of P_cuff_ underinflation as compared with manual intermittent adjustments. Overinflation was not promoted by this device.

**References:**

1. Rello J. et al. Am J Respir Crit Care Med 154:111-5, 1996

2. Nseir S et al. Crit Care 11:R109, 2007

3. Leone et al. Anaesth Crit Care Pain Med 37:83-98, 2018

## P113 Percutaneous tracheotomy in ICU advantage and complications

### V Pajtic^1^, S Zjalic^2^, B Josipovic^2^, N Ilic^2^, B Radanovic^2^, D Barac^2^, L Gvozdenovic^3^, S Lovrencic^2^

#### ^1^University of Novi Sad, Faculty of Medicine, Clinic for Anesthesia Intensive and Pain Therapy Clinical Center of Vojvodina, Novi Sad, Serbia; ^2^Clinic for Anesthesia Intensive and Pain Therapy Clinical Center of Vojvodina, Novi Sad, Serbia; ^3^University of Novi Sad, Faculty of Medicine, Novi Sad, Serbia

**Introduction:**

Percutaneous tracheotomy (PT) in patients at the ICU is an invasive method of airway providing. It is requiring prolonged mechanical ventilation. PT as a method has proven to be simpler than surgical tracheosomy (ST), because it is performed in hospital bed without transporting the patient to the operating room, which is important in cases of hemodynamically unstable patients. Original kits, quick training and ability to perform in patient's bed, have imposed PT as the method of choice for ICU patients.

**Methods:**

In a one-year retrospective study, we have followed 161 patients in 2 groups, at ICU II, Clinical for Anesthesia and ICU and Pain Therapy, Clinical Centre of Vojvodina. Patients in ICU underwent PT with assistance of fiberoptic bronchosce, or ST in the operating room, due to the need for prolonged mechanical ventilation. Ultrasound Doppler examination of the blood vessels in the neek and their relationship with trachea determined the indication for PT or ST. In PT group were 110 patients, in ST 51. We have observed early and late complications and lenght of mechanical ventilation after the tracheostomy was performed.

**Results:**

On most patients (110) we have made PT assisted by fiberoptic bronchoscope. 51 patients underwent ST. Early complications in PT in terms of minor bleeding (<100ml) from the smaller vein blood vessels of the skin and subcutaneous tissue of the neek were 3,6% during the performance. Serious complication, false route, subcutaneous cannula placement, with subcutaneous emphysema was 0,9%. The late complications of PT in the form of tracheal stenosis are still being monitored, and no reports have been reported so far. Early complications in ST( bleeding, twisting cannula as well as malposition) were 20%.

**Conclusions:**

PT with assistance of fiberoptic bronchoscop has proven to be a safe and rapid invasive method, with no more complication than ST, in the ICU performed by ICU doctors on patients, in bed without transport to the operating room and as a safe alternative to ST.

## P114 A comparison of the intersurgical i-View and Macintosh laryngoscope when used by novice personnel: a manikin study

### MO O´Sullivan^1^, EO Imhoff-O´Mahony^2^

#### ^1^Mercy University Hospital, Anaesthesiology and Intensive Care, Cork, Ireland; ^2^Sligo University Hospital, Anaesthesiology and Intensive Care, Sligo, Ireland

**Introduction:**

Direct laryngoscopy as a technique for tracheal intubation is a potentially lifesaving procedure that healthcare professionals in a variety of fields are taught. However, this skill is challenging to acquire and difficult to maintain. Poorly performed intubation technique can lead to potentially serious complications [1]. The Intersurgical iView video laryngoscope is a new intubation tool which may have advantages over direct laryngoscopes, such as the Macintosh, in the hands of novice personnel.

**Methods:**

A prospective randomized counterbalanced trial of 30 medical students, who did not have previous airway management experience, was conducted. Each student received brief didactic teaching,following this, participants were directly supervised performing laryngoscopy and intubation using the Macintosh and iView devices in an alternating pattern. Students were permitted up to three attempts to successfully intubate under four conditions, three laryngoscopy conditions using aLaerdal Intubation Trainer and one using a Laerdal SimMan Manikin.

**Results:**

There was no significant difference in the success rate of intubation or time to intubation between the two devices. The iView outperformed the Macintosh in time to intubation in the normal airway in the final scenario, once students gained experience with both devices. No significant difference was found in the number of optimisation manoeuvres, or intubation attempts between groups. Areas where the iView outperformed the Macintosh included severity of dental trauma and participants’ perception regarding ease of use ofthe device.

**Conclusions:**

The iView may prove to be a useful teaching tool for novice personnel who are acquiring the skills of tracheal intubation.

**References:**

1. Mulcaster JT et al. Anesthesiology 98:23-27, 2003

## P115 Assessing predictors of success in airway pressure release ventilation (APRV)

### SF Villegas^1^, M McHendry^1^, S Gillon^2^, M Al-Haddad^1^

#### ^1^Queen Elizabeth University Hospital, Department of Critical Care, Glasgow, United Kingdom; ^2^Royal Infirmary of Edinburgh, Department of Critical Care, Edinburgh, United Kingdom

**Introduction:**

Airway pressure release ventilation (APRV) is a mechanical ventilatory mode where elevated continuous positive airway pressure (CPAP) is applied with periodic releases to facilitate CO_2_ clearance [1]. To date, there are no clear parameters that predict a positive response to APRV. The purpose of our study was to identify these.

**Methods:**

This was a retrospective cohort study in a single, large tertiary adult ICU from 2015-2018. Demographic, pathological and physiological data were extracted from the clinical information system CareVue ©. APRV responders were defined as patients remaining on APRV at four hours post initiation with a positive change in PF ratio. The cohort was stratified by responder status; univariate and multivariate analyses were used to identify factors associated with APRV response. Figures are mean (SD). Anonymized data was stored in a password-protected, securely stored computer file.

**Results:**

103 patients mean age 53.4 (15.5) were ventilated using APRV and 96 fulfilled inclusion criteria. 55% were male, 61% had a primary pulmonary pathology. Total ventilated days were 12.1 (9.6). Prior to APRV, 6% had evidence of left ventricular systolic dysfunction, PaO2 was 9.7 (3.0), PF ratio was 15.1 (9.3), PaCO2 was 7.1 (2.2), H+ was 52 (19), PPeak was 25.9 (5.4), PEEP was 10.5 (3.2), Vt/kg was 7.4 (2.6), CRP was 186 (123). APRV settings were: Phigh 27.5 (4.2), Plow 0 (0.4), Thigh 4.5 (1.1), Tlow 0.5 (0.3). 1% were ventilated prone, 4% had a neuromuscular blocker infused and 11% had a neuroprotective strategy employed at time of APRV. 48 patients responded to APRV (table 1). Hospital mortality was 31.2% in responders vs 54.2% in non-responders (p=0.037).

**Conclusions:**

Patients with a primary pulmonary pathology were more likely to respond to APRV. This association has not been described before and warrants further multi-centre exploration in a larger patient group.

**References:**

1. Jain SV et al. Intensive Care Med Exp 4:11, 2016.

**Table 1 (abstract P115).** Comparison of APRV responder variables (physiology pre-APRV)

Variable	Responder (n=48)	Non-responder (n=48)	P value
APACHE II score	21.5 (8.4)	23.6 (10.5)	0.311
Fluid balance (ml)	+3258 (4107)	+4172 (5508)	0.382
Hours ventilated prior to APRV	53.3 (65)	87 (213)	0.3
PaCO2	6.7 (1.8)	7.6 (2.5)	0.047
PEEP	11.0 (3.2)	10.0 (3.1)	0.12
PF Ratio	13.9 (6.8)	16.7	0.189
Primary pulmonary pathology	70.8%	50%	0.037

## P116 Preventing atelectasis during closed endotracheal suctioning

### C Lima^1^, G Alcala^1^, S Gomes^1^, R Santiago^2^, M Amato^1^

#### ^1^University of Sao Paulo Medical School, Pulmonary Division, Heart Institute (InCor), São Paulo, Brazil; ^2^Massachusetts General Hospital, Department of Anesthesia, Critical Care and Pain Medicine, Boston, United States

**Introduction:**

Airway suctioning is common during mechanical ventilation, using either an open endotraqueal suctioning or closed endotracheal suctioning (CES). Closed circuits were developed to prevent arterial desaturation and atelectasis associated to ventilator disconnection. However, CES may cause substantial loss of lung volume. The purpose of this study was to investigate the effects of a compensation method to prevent the loss in aeration during CES.

**Methods:**

The suctioning technique was performed for 15 seconds, negative pressures limited at 150 mmHg. Closed suction catheters with 14Fr (Halyard Health, Georgia, EUA) were used. Electrical impedance tomography (EIT) monitoring and arterial blood gas were collected. A NihonKoden Mechanical Ventilator (NKV550, California, EUA) was applied, having a newly developed algorithm for suctioning which overcomes any pressure loss during suctioning (InlineSuction-APP). When activated, the APP delivers PCV ventilation, adding 2 cmH_2_O of end-expiratory pressure above PEEP, and delivering driving pressures of 15 cmH_2_O.

**Results:**

Pigs (30±5.4kg) with injured lungs and mechanically ventilated. We tested the aspiration procedures using low PEEP=5cmH_2_O, or high PEEP=±12.3cmH_2_O with V_T2_O), whereas maintenance of compliance was observed when the APP was ON (from12.2±1.4 ml/cmH_2_O to 12.5±4.5 ml/cmH_2_O. Blood gas in a representative animal showed a drop in PaO_2_ when APP was off (from 247, to 149 mmHg after 2 min, and to 176 mmHg after 10 min) (Figure 1). With APP ON the PaO_2_ changed from 259 (pre-suction), to 223 (2 min), to 253 mmHg (10 min).

**Conclusions:**

The new NKsoftware, delivering PCV ventilation during suctioning, could prevent atelectasis and functional loss associated to the procedure.

**Fig. 1 (abstract P116).**
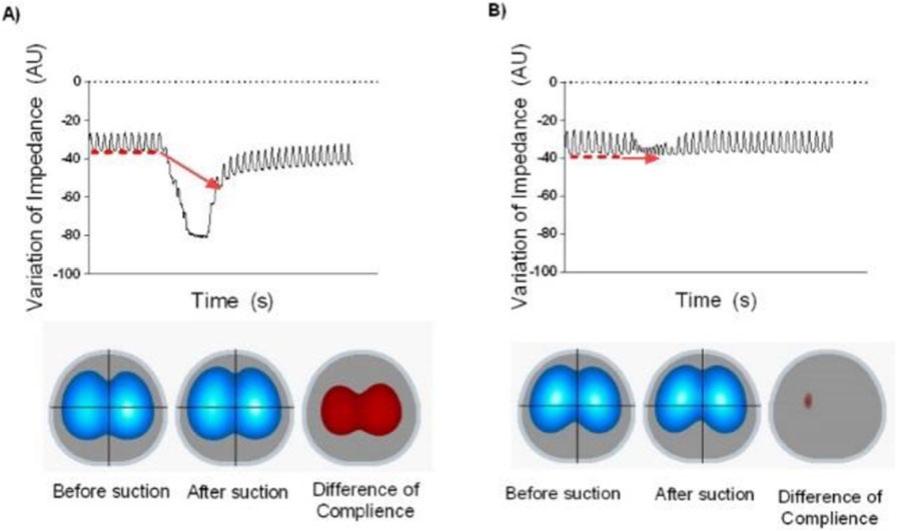
Representative image showing the ventilation maps before and after closed endotracheal suctioning (CES). A) The drop in the EIT plethysmogram during suctioning, when no Inline Suction App was used – associated with a significant drop of lung compliance. B) A minor change in EIT ventilation map and in the plethysmogram when the Inline Suction APP was ON

## P117 Tyrosine kinase inhibitor: an effective tool against lung cancer involvement responsible for acute respiratory failure in ICU

### Y Tandjaoui-lambiotte^1^, Y Akrour^1^, F Gonzalez^2^, A Stoclin^3^, F El Kouari^4^, A Gibelin^5^, F Wallet^6^, A Oppenheimer^7^, B Duchemann^8^, S Gaudry^1^

#### ^1^Avicenne Hospital, Intensive Care Unit, Bobigny, France; ^2^Institut Paoli Calmettes, Intensive Care Unit, Marseille, France; ^3^Institut Gustave Roussy, Intensive Care Unit, Villejuif, France; ^4^Pitié Salpetrière Hospital, Pharmacy, Paris, France; ^5^Tenon Hospital, Intensive Care Unit, Paris, France; ^6^Lyon Sud Hospital, Intensive Care Unit, Lyon, France; ^7^Versailles-Saint-Quentin University (UVSQ), EA 7285 Research Unit ´Risk and Safety in Clinical Medicine for Women and Perinatal Health´, Montigny-le-Bretonneux, France; ^8^Avicenne Hospital, Oncology, Bobigny, France

**Introduction:**

Patients with advanced-stage non-small-cell lung cancer have high mortality rates in the intensive care unit (ICU). In the last two decades, targeted therapies have changed the prognostic of patients with lung cancer outside the ICU. The fast efficacy of targeted therapies led some intensivists to use them as rescue therapy for ICU patients.

**Methods:**

We performed a national multicentric retrospective study with the participation of the GRRROH (Groupe de Recherche en Réanimation Respiratoire en Onco-Hématologie). All patients with non-small-cell lung cancer admitted to the ICU for acute respiratory failure between 2009 and 2019 were included in the study if a Tyrosine Kinase Inhibitor was initiated during ICU stay. Cases were identified using hospital-pharmacies records. The primary outcome was overall survival 90 days after ICU admission.

**Results:**

Thirty patients (age: 60+/-14 years old) admitted to a total of 14 ICUs throughout France were included. Seventeen patients (59%) were nonsmoker. Adenocarcinoma was the most frequent histological type (n=21, 70%). Most patients had metastatic cancer (n=21, 70%). Epithelial Growth Factor Receptor mutation was the most common oncologic driver identified (n=16, 53%). During the ICU stay, 17 (57%) patients required invasive mechanical ventilation, 13 (43%) catecholamine infusion, 3 (10%) renal replacement therapy and one (3%) extracorporeal membrane oxygenation. Eighteen patients (60%) were discharged alive from ICU and 11 (37%) were still alive after 90 days (see Figure). Moreover, 6 patients (20%) were alive one year after ICU discharge.

**Conclusions:**

Despite a small sample size this study showed that, in the context of lung cancer involvement responsible for acute respiratory failure, the use of Tyrosine Kinase Inhibitor should not be refrained in patients with severe condition in ICU.

**Fig. 1 (abstract P117).**
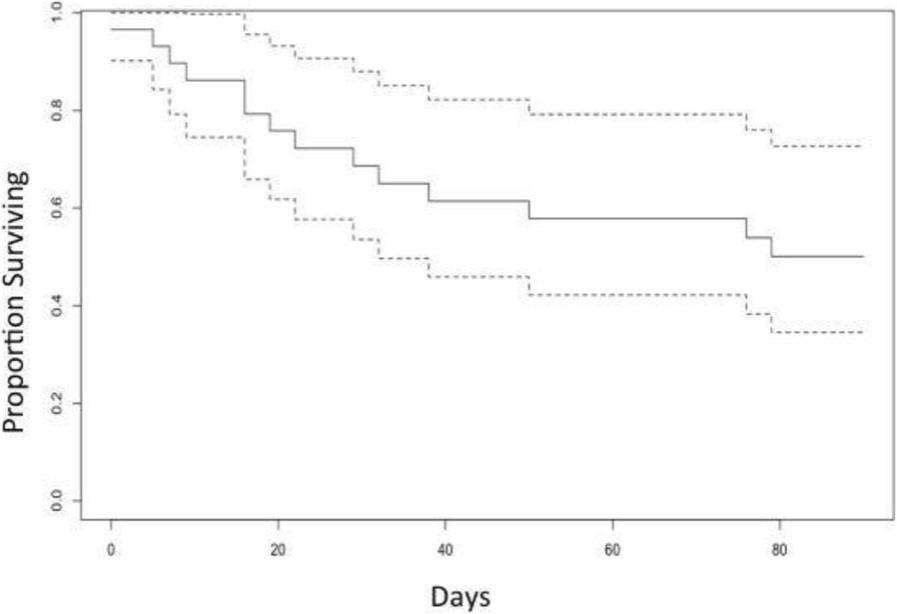
Probability of survival after ICU admission

## P118 Community-acquired pneumonia: factors predicting poor outcome

### C Bozikovich^1^, A Ramos^2^, A Dogliotti^3^, B Espinosa^1^, N Rodriguez^1^, C Lovesio^2^

#### ^1^Sanatorio Parque, Internal Medicine, Rosario, Argentina; ^2^Sanatorio Parque, Critical Care, Rosario, Argentina; ^3^Grupo Oroño, Epidemiology and statistics, Rosario, Argentina

**Introduction:**

Community-acquired pneumonia (CAP) is the most common infectious disease requiring admission to intensive care units (ICUs). The aim of this study was to investigate factors predicting poor outcome: the composite of in-hospital mortality and/or mechanical ventilation, in patients admitted to hospital because CAP.

**Methods:**

Between june 1, 2014 and september 30, 2018; 312 patients with CAP were prospectively registered. On admission data including patient characteristics, clinical findings, radiologic features and need for mechanical ventilation were assessed and further analyzed. Mechanical ventilation was defined as the requirement of invasive and/or non-invasive ventilation at any time during hospitalization. A multivariate analysis was performed to identify predictors of poor outcome.

**Results:**

Three hundred and twelve patients were included in the study and 26% of them had a poor outcome. In multivariate analysis, patients with a history of chronic obstructive pulmonary disease (COPD) (p=0.002), and those with elevated values of heart (p=0.001) and respiratory rates (p=0.01), and increased urea levels (p=0.01) had a worse outcome. (AUC ROC 0.83; 95% IC: 0.78-0.87).

**Conclusions:**

A history of COPD, elevated heart and respiratory rates and increased urea levels at admission were independent variables of poor outcome in patients with CAP.

## P119 Mortality study of burn patients with inhalation injury in a critical burn unit

### L Cachafeiro^1^, A Agrifoglio^1^, M Sánchez^1^, E Herrero^1^, A García de Lorenzo^2^

#### ^1^Hospital Universitario La Paz, Intensive Care Medicine Service, Madrid, Spain; ^2^Hospital Universitario La Paz, Madrid, Spain

**Introduction:**

The burned patient is one of the most complex patients whith a very high mortality. Those patients with inhalation injury have a worst prognosis, typically associated with respiratory complications. The aim of our study is to evaluate the mortality of burn patientes with inalation injury in a critical burn unit.

**Methods:**

A prospective, observational and descriptive study was conducted over a period of 3 years. Inhalation injury was defined with these criteria (≥ 2): history of injury in an enclosed space, facial burns with singed nasal hair, carbonaceus sputum and stridor. If they were intubated it was diagnosed by bronchoscopy. Demographic data, TBSA, ABSI, Baux score, APACHE II, SOFA, mechanical ventilation (MV), complications, length of stay, hospital course and mortality data were collected.

**Results:**

362 burns patients were admitted. 24% (84 patients) had inhalation injury. Mortality among patients with inhalation injury was 28,6% (24 patients). Most patients were men and those who died were older and with higher severity scores (Fig. 1). We found no significant differences between groups in the need for MV (95% vs. 85%) or in the percentage of tracheostomy performed (33.3 vs. 28.3). However, patients who died had more respiratory complications like ARDS, and also shock, renal failure and need of renal replancement therapies although infectious complications were similar in both groups. There was no statistically significant difference in volume used during initial resuscitation in the different groups.

**Conclusions:**

Patients with inhalation injury who died had higher severity scores at the begining. Although there were no differences in the need for MV patients who died had more respiratory complications as well as shock, renal failure and need of RRT, but no infectious complications.The volume used during inicial resuscitation, that was always related to the prognosis, was similar in both groups. Further studies are needed to see if this greater initial severity corresponds to the degree of inhalation.

**Fig. 1 (abstract P119).**
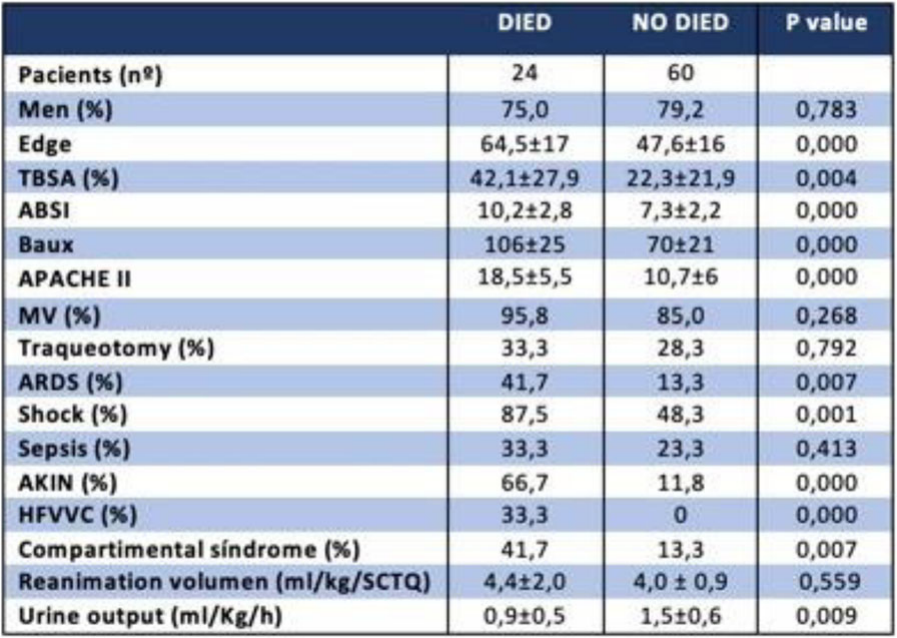
Demographic characteristics and severity scores

## P120 Consistency of aerosol dosing during the progression of respiratory therapy in the hospital setting

### E Fernandez fernandez^1^, G Bennett^1^, A Tatham^1^, A O´ Sullivan^2^, P McKiernan^1^, M Mac Giolla Eain^2^, M Joyce^2^, R MacLoughlin^2^

#### ^1^Aerogen, Medical Affairs, Galway, Ireland; ^2^Aerogen, Science, Galway, Ireland

**Introduction:**

Patients with acute exacerbations such as asthma are prescribed aerosol therapy from presentation in the Emergency Department to progression through to the Intensive Care Unit. However, the variability in dose delivery to the lung across the possible patient interventions is not well characterized. Here, we assess the predicted lung dose of a bronchodilator in a simulated spontaneously breathing adult patient via both facemask and nasal cannula, and via tracheostomy during mechanical ventilation.

**Methods:**

A standard dose of 2.5 mg in 2.5 mL salbutamol was aerosolized using the Aerogen Solo nebulizer (Aerogen, Ireland). For facemask testing, the nebulizer was used in combination with the Aerogen Ultra with 2LPM supplemental oxygen flow. For nasal cannula testing, the nebulizer was used in combination with the Airvo 2 system (Fisher and Paykel, NZ) system at both 10 and 50LPM gas flow rate. Tracheostomy-mediated ventilation was assessed in combination with a HME, with the nebulizer placed between the HME and the tracheostomy tube. International Standard ISO27427 adult breath settings (Vt 500mL, BPM 15, I:E 1:1) were used across all tests, and generated using a breathing simulator (ASL5000, Ingmar Medical, USA) or mechanical ventilator (Servo-U, Maquet, Sweden). The dose delivered to the lung was assessed using a capture filter at the level of the trachea, with drug mass determined using UV Spectrophotometry at 276nm and interpolation on a standard curve.

**Results:**

The results of testing are illustrated in Figure 1.

**Conclusions:**

The bronchodilator dose delivered to the simulated patient was seen to be relatively consistent between progressive interventions, except during high flow therapy, with the more clinically relevant 50LPM gas flow rate having a profound effect on the dose. These results may go some way towards explaining how different patient interventions can affect aerosol dose.

**Fig. 1 (abstract P120).**
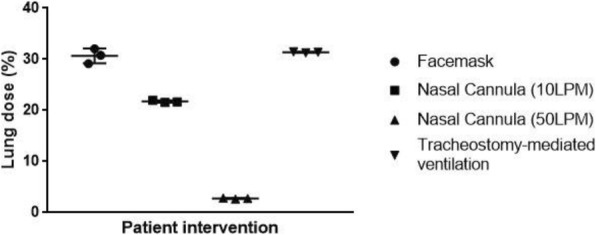
Illustration of lung dose for each patient intervention

## P121 The underlying inflammatory effect of stable COPD (chronic obstructive pulmonary disease) patient on thrombogenicity and clot microstructure

### S Pillai^1^, M Lawrence^2^, J Whitley^3^, K Harrison^3^, E Evans^4^, A Mughal^4^, S Hilldrup^4^, PR Williams^5^, K Morris^6^, PA Evans^3^

#### ^1^Welsh Centre for Emergency Medicine Research, Emergency Department, Morriston Hospital, Swansea, United Kingdom; ^2^Welsh Centre for Emergency Medicine Research, Emergency Department, Morriston Hospital, Swansea, United Kingdom; ^3^Welsh Centre for Emergency Medicine Research, Emergency Department, Morriston Hospital, Swansea, United Kingdom; ^4^Respiratory Medicine, Morriston Hospital, Swansea, United Kingdom; ^5^Swansea University, Swansea, United Kingdom; ^6^Cardiff Metropolitan University, Cardiff, United Kingdom

**Introduction:**

COPD patients are known to have marked underlying lung parenchymal damage and associated microvascular changes [1]. They have two to four times increased mortality from ischemic heart disease and one fourth of patients develop pulmonary embolism particularly during acute exacerbations. The aim of the study was to demonstrate whether these patients’ underlying inflammatory response promotes thrombotic risk using the functional biomarker (fractal dimension-d_f_). The stable COPD patients were divided into four groups based on their Forced Expiratory Volume at one second (FEV1); mild (>80%), moderate (50-80%), severe (30-50%) and very severe (<30%).

**Methods:**

30 stable ambulatory COPD patients with no evidence of infection or any underlying disease process that affect their coagulation system where recruited from the chest clinic of a tertiary teaching hospital. Blood samples were taken to perform fractal dimension (d_f_), full blood count, platelet aggregometry, PT, aPTT, fibrinogen, d-dimer, CRP and Factor XIII.

**Results:**

The mean d_f_ in stable COPD patients was 1.690 (normal d_f_ 1.73 ± 0.04). The very severe group had highest d_f_ (1.712 ± 0.023, p=0.103), had significantly low FEV1 (24.20 ± 3.70, p<0.001) and significantly low BMI (21.57 ± 1.49, p<0.001). There were no significant rise in inflammatory markers, standard markers of coagulation and d-dimer.

**Conclusions:**

Ambulatory COPD patients who has a well-controlled disease have a normal coagulation profile despite having known parenchymal and vasculo-inflammatory disease. The clot microstructure (d_f_) in these patients are within normal range. The study demonstrates that ambulatory COPD patients are less prone to develop thromboembolism and an increased risk occurs due to an exacerbation, sepsis or increased immobilisation.

**References:**

1. Davies et al. Intensive Care Med 42:1990-1998, 2016.

## P122 Respiratory support for lung cancer patients with acute respiratory failure

### HK Atalan^1^, ME Kavlak^1^, B Gucyetmez^2^, ZT Sarikaya^2^

#### ^1^Memorial Atasehir Hospital, Intensive Care Unit, Istanbul, Turkey; ^2^Mehmet Ali Aydinlar Acibadem University, Intensive Care Unit, İstanbul, Turkey

**Introduction:**

The mechanical ventilation (MV) have been identified as an independent factor indicating a worse prognosis for lung cancer patients [1]. This study was conducted in order to assess the results of non-invasive mechanical ventilation (NIV) and/or invasive mechanical ventilation (IMV) modalities in lung cancer patients admitted to the ICU with acute respiratory failure (ARF).

**Methods:**

In this study, lung cancer patients with respiratory failure who were admitted to the ICU between January 2017 and December 2018 were evaluated retrospectively.

**Results:**

93 patients were included in the study. The mortality rate was 18.3%. 83 patients had NIV. IMV was applied to 10 patients. In the first 24 hours, 39 of the 83 patients who were initially treated with NIV were administered IMV. The duration of hospital stay, diagnosis of pneumonia and mortality rate were found to be significantly lower in patients treated with NIV alone (p≤0.001, p=0.004, p=0.025), but Glaskow Coma Score (GCS) was significantly higher in this group (p≤0.001). The mortality rate was similar between the patients who were initially treated with IMV and those who were treated with IMV in the first 24 hours. Charlson Comorbidity Index (CCI) and MV duration were significantly higher in patients who died (p=0.01, p=0.021), but GCS was significantly lower in this group (p=0.032). In the linear regression model for the likelihood of mortality, CCl≥9 and unsuccessful NIV increased the mortality rate by 3.4 (1.1-10.5) and 5.2 times (12-23.6) respectively (p=0.036, p=0.032).

**Conclusions:**

NIV has been an effective modality for respiratory support in most lung cancer patients presenting with ARF. However, failed NIV seems to be a factor for increased mortality. Therefore, the choice of respiratory support modality to be applied in this patient group should be decided by considering the GCS, CCI and etiology of ARF.

**References:**

1. Roques S et al. Intensive Care Med 35:2044-50, 2009.

## P123 Association between PEEP level and renal blood flow in mechanically ventilated patients

### A Fogagnolo^1^, C Calandra^2^, M Dres^3^, E Morelli^2^, G Benetto^2^, F Franchi^4^, S Scolletta^5^, CA Volta^2^, S Spadaro^2^

#### ^1^Morphology, Surgery and Experimental Medicine, University Anaesthesia and Intensive Care, Ferrara, Italy; ^2^Morphology, Surgery and Experimental Medicine, Ferrara, Italy; ^3^Sorbonne Universités, Neurophysiologie Respiratoire Expérimentale et Clinique, Paris, France; ^4^University Hospital of Siena, Department of Medicine, Surgery and Neurosciences, Anesthesia and Intensive Care Unit, Siena, Italy; ^5^University Hospital of Siena, Siena, Italy

**Introduction:**

The interaction between ventilator settings and the occurrence of acute kidney injury is not fully elucidated. This study aimed at investigating the effect of stepwise increase in PEEP level on the risk of acute kidney injury as evaluated with the renal resistivity index (RRI).The primary outcome is to investigate whether increased levels of PEEP could lead to increase RRI and whether RRI could predict the occurrence of AKI.

**Methods:**

Patients mechanically ventilated for at least 48 hours and without AKI at admission were included in the study. RRI was calculated at ICU admission. Posterolateral approach was used for kidney ultrasound. The peak systolic velocity (V_max_) and the minimal diastolic velocity (V_min_) were determined by pulse wave Doppler, and the RRI was calculated as (V_max_ -V_min_)/V_max_. The exam was performed modifying the PEEP levels: 5, 10 and 15 cm H_2_O in random order for 15 minutes. Occurrence of AKI was defined within 7 days according to KDIGO criteria.

**Results:**

Sixty-four patients were enrolled in the study and incidence of AKI was 14/64 (22%). Demographical and clinical characteristics are reported in Table 1. Increase in PEEP showed a significant increase in RRI from PEEP 5 to PEEP 10 (p<0.001) and from PEEP 10 to PEEP 15 (p=0.001) (Figure 1). The area under the ROC curve of RRI to predict AKI was 0.845 at PEEP 5, 0.898 at PEEP 10 and 0.894 at PEEP 15 (all p<0.001). The Youden index analysis showed an RRI>0.70 as the best cut off for AKI with a sensibility of 65% and a specificity of 96%. Patients with RRI>0.70 were 11/64 (17%), 13/64 (20%) and 22/64 (34%) at PEEP 5,PEEP 10 and PEEP 15 respectively. Patients ventilated with a PEEP value associated with RRI>0.70 had higher incidence of AKI (11/14 vs 6/50, p<0.001).

**Conclusions:**

The application of PEEP can increase intrarenal vascular resistance,which is associated occurrence of AKI; PEEP level should therefore be balanced taking into account the RRI. The RRI seems able to predict occurrence of AKI in mechanically ventilated patients.

**Fig. 1 (abstract P123).**
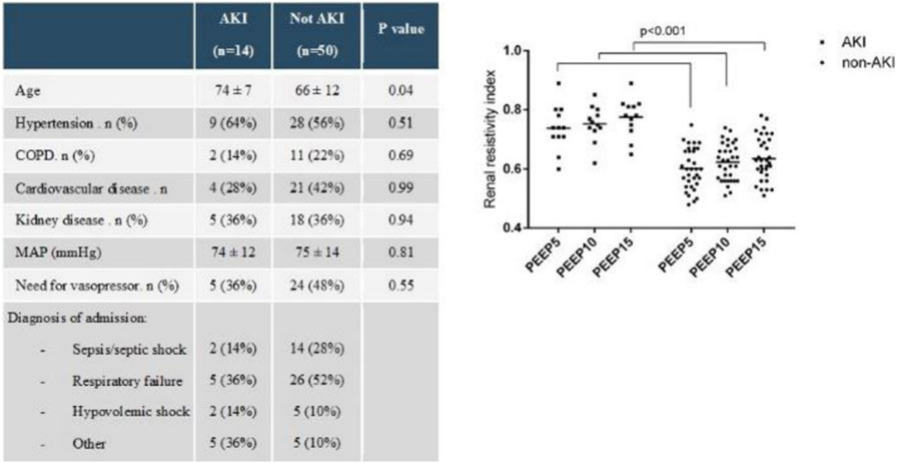
Demographic and clinical characteristics of the patients (left panel); RRI values in AKI and not-AKI patients at each level of PEEP (right panel)

## P124 Alveolar and respiratory mechanics modifications produced by different concentrations of oxygen in healthy rats subjected to mechanical ventilation with protective ventilatory strategy

### D Dominguez Garcia^1^, R Hernandez Bisshopp^1^, JL Martin Barrasa^2^, D Viera Camacho^1^, A Rodriguez Gil^1^, J Arias Marzan^1^, S Garcia Hernandez^3^, L Perez Mendez^4^, F Valladares Parrilla^3^, J Villar^2^

#### ^1^Hospital Universitario Ntra. Sra. de Candelaria, Anesthesiology and Surgical Critical Care, Santa Cruz de Tenerife, Spain; ^2^Hospital Universitario de Gran Canaria Dr. Negrín, Research Unit, Las Palmas de Gran Canaria, Spain; ^3^University of La Laguna, Anatomy, Pathology and Histology, La Laguna, Spain; ^4^Hospital Universitario Ntra. Sra. de Candelaria, Research Unit, Santa Cruz de Tenerife, Spain

**Introduction:**

High oxygen can damage tissues [1]. In this study, we analyze the histological and pulmonary mechanics modifications that can occur when identifying different inspiratory oxygen fractions (FiO_2_) in lungs of healthy rats during protective mechanical ventilation.

**Methods:**

We use Sprague-Dawley rat. 4 groups were designed, each with 6 animals, the tidal volume (6 ml/kg), PEEP (3 cmH_2_O) and respiratory rate (90 rpm) were kept constant, changing the FiO_2_ between the groups. Four groups were established: FiO_2_ 0.21, 0.4, 0.6 and 1. After 4 hours, the lungs were removed for histological study and obtaining the wet/dry index. The histological modifications studied were: alveolar septa (AS), alveolar hemorrhages (AH), intraalvelolar fibrin (IF) and inflammatory infiltrates (II). Each parameter was rated from 0 to 3 [2]. Peak pressure (Pp) and pulmonary compliance were monitored every 60 minutes. Different statistical tests will be used to analyze the data.

**Results:** References to the damage produced in the AS, AH, IF, II and the global histological pattern were identified in the groups with the highest FiO_2_ and there was more damage (p <0.00001) (Figure 1). The wet/dry index rose significantly as the oxygen concentration increased (p = 0.001). In the groups to which a FiO_2_ of 0.6 and 1 was administered, the Pp selected specific values with respect to the baseline intake from the first 60 minutes, an aspect that was not appreciated in the other groups (p <0.0001). Regarding pulmonary compliance, it will be seen that, in the FiO_2_ 0.6 and 1 groups, it decreased from the first 60 minutes, finding differences with respect to the other groups (p <0.0001).

**Conclusions:** Mechanical ventilation applied for 4 hours in healthy animals produces disorders that are more pronounced as oxygen concentration increase. FiO_2_ greater than or equal to 0.6 should be avoided without clinical justification.

**References:**

1. Hedenstierna G et al. Intensive Care Med Oct:1-4, 2019

2. Matute-Bello G et al. Am J Respir Cell Mol Biol. 44: 725-38, 2011

**Fig. 1 (abstract P124).**
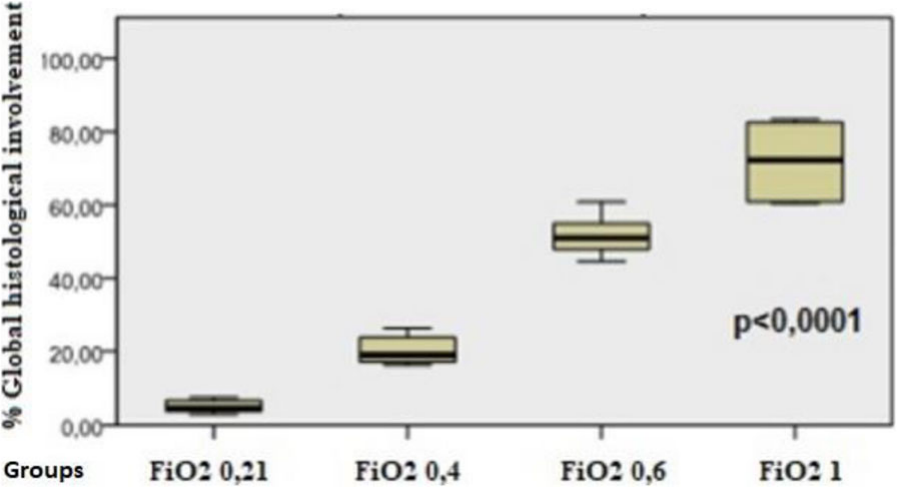
Distribution of the percentage of global histological involvement among the groups (median and P25-75)

## P125 Clinical outcomes among patients requiring prolonged acute vs. short-term mechanical ventilation in the US, 2014-2018: results from a multicenter cohort study

### M Zilberberg^1^, B Nathanson^2^, J Ways^3^, A Shorr^4^

#### ^1^EviMed Research Group, LLC, Goshen, United States; ^2^OptiStatim, LLC, Longmeadow, United States; ^3^Lungpacer Medical Inc., Exton, United States; ^4^Washington Hospital Center, Washington, United States

**Introduction:**

Patients requiring prolonged acute mechanical ventilation (PAMV, defined as 4+ days on MV) are sicker and incur disproportionate morbidity and costs relative to patients on short-term MV (STMV, <4 days of MV). We quantified specific clinical outcomes among patients requiring PAMV vs. STMV in a contemporary database.

**Methods:**

We conducted a multicenter retrospective cohort study within ~700 hospitals in the Premier database, 2014-2018. Using ICD-9-CM and ICD-10 codes we identified PAMV and STMV patients, and compared their baseline characteristics and hospital events. Because of the large sample size, we omitted hypothesis testing.

**Results:**

A total of 691,961 patients met the enrollment criteria, of whom 266,374 (38.5%) received PAMV. At baseline, patients on PAMV were similar to STMV with regard to age (years: 62.0 ± 15.8 PAMV vs. 61.7 ± 17.2 STMV), gender (males: 55.6% PAMV vs. 53.9% STMV), and race (white: 69.1% PAMV vs. 72.4% STMV). PAMV group had a higher comorbidity burden than STMV (mean Charlson score 3.5 + 2.7 vs. 3.1 + 2.7). The prevalence of each of the indicators of acute illness severity – vasopressors (50.3% vs. 36.9%), dialysis (19.4% vs. 10.3%), severe sepsis (20.3% vs. 10.3%), and septic shock (33.5% vs. 15.9%) – was higher in PAMV than STMV, as were hospital mortality and combined mortality or discharge to hospice (Figure 1), extubation failure (12.3% vs. 6.1%), tracheostomy (21.6% vs. 4.5%), development of *C. difficile* (4.5% vs. 1.7%), and incidence density of ventilator-associated pneumonia (2.4/1,000 patient-days vs. 0.6/1,000 patient-days).

**Conclusions:**

Over 1/3 of all hospitalized patients on MV require it for 4 days or longer. PAMV patients exhibit a higher burden of both chronic and acute illness than those on STMV. Commensurately, all clinical outcomes examined are substantially worse in association with PAMV than STMV.

**Fig. 1 (abstract P125).**
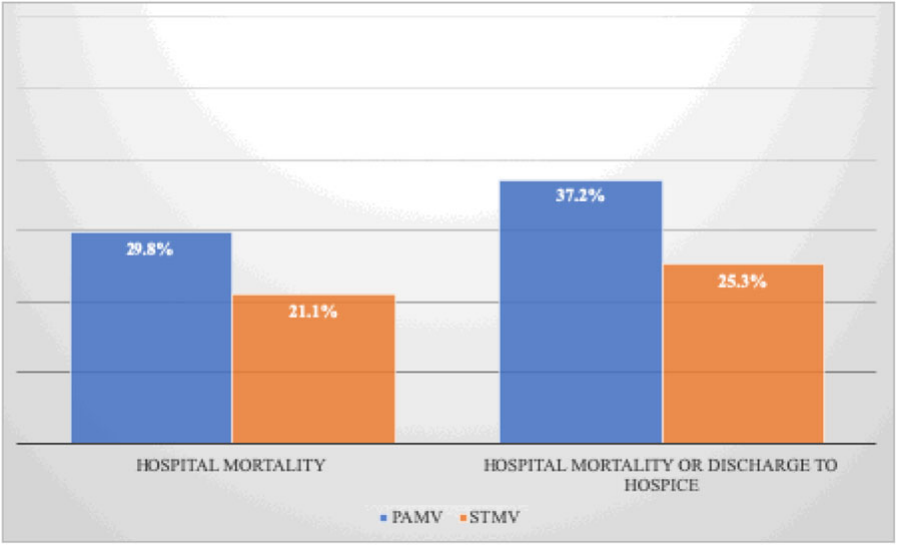
PAMV = prolonged acute mechanical ventilation; STMV = short-term mechanical ventilation

## P126 Use of spontaneous breathing trials and extubation data in Scottish ICUs - 2018 Scottish Intensive Care Society trainee audit

### A Short^1^, K Puxty^2^, D Hall^3^, J Gardner^1^, S Chapman^4^, N Lone^3^, N Stewart^5^

#### ^1^QEUH, Intensive Care Unit, Glasgow, United Kingdom; ^2^Glasgow Royal Infirmary, Intensive Care Unit, Glasgow, United Kingdom; ^3^Edinburgh Royal Infirmary, Intensive Care Unit, Edinburgh, United Kingdom; ^4^Ninewell Hospital, Intensive Care Unit, Dundee, United Kingdom, ^5^Forth Valley Royal Hospital, Intensive Care Unit, Larbert, United Kingdom

**Introduction:**

Identifying the readiness of patients recovering from critical illness for liberation from invasive mechanical ventilation (IMV) is not always straightforward [1]. The Scottish Intensive Care Society (SICS) trainee audit 2018 conducted a Scotland-wide study to understand current practices relating to liberation from IMV.

**Methods:**

Data were prospectively collected on patient demographics, indication for intubation, spontaneous breathing trial (SBT) practices, physiological markers, ICU outcome and ICU LOS. All patients >18 years ventilated with IMV for > 24hrs from the 1^st^Nov. 2018 – 30^th^Nov. 2018 were eligible for inclusion. Exclusion criteria included extubation for end-of-life, death whilst intubated and presence of tracheostomy. Logistic regression was performed to detect factors associated with extubation failure (EF). Results were analysed via Excel 2010 and Stata v.14.1. Patient Benefit and Privacy Panel approval was granted.

**Results:**

Total population of 172 patients were included: 108 (63%) male and median APACHE2 score 19 (IQR 13-23). EF at first attempt occurred on 27 occasions (15.7%), median ICU LOS of 10 days (IQR 7–12), mortality rate 22.2%. The cohort successfully extubated first time had a median ICU length of stay of 5 days (IQR 3–9) and mortality rate of 1.4%. Methods of SBT and extubation outcomes detailed in Table 1. No SBT prior to extubation had higher odds of EF (OR 2.52, CI 1.09-5.84, p=0.03); patient ventilation for < 3 days had a three times higher odds of EF (OR 3.31, CI 1.09-10.1, p=0.03). These were independently associated with EF on multivariate analysis

**Conclusions:**

We found a reintubation rate of 15.7% in Scottish ICUs. Type of SBT most commonly used is divergent from the methods advocated in the literature. The lack of SBT and early extubation attempt was associated with failure, which in turn was associated with longer ICU LOS and higher mortality.

**References:**

1. MacIntyre N. Respir Care 58:1074 – 1082, 2013

**Table 1 (abstract P126).** Extubation outcomes associated to ventilation days and SBT performed

	Total population (n=172)	Successful first extubation attempt (n=145)	Failed first extubation attempt (n=27)	Univariate analysis (p-value)
Time from intubation to first extubation <3days –	115 (66.9)	92 (63.4)	23 (85.2)	0.02
SBT not performed – no. (%)	69 (40.1)	53 (36.6)	16 (59.3)	0.03
	Total SBT population (n=103)	Successful first extubation (n=92)	Failed first extubation (n=11)	
T-piece - no. (%)	9 (8.7)	8 (8.7)	1 (9.1)	-
CPAP only - no. (%)	64 (62.1)	58 (63.0)	6 (54.6)	-
PS 5-8 ≥ PEEP - no. (%)	28 (27.2)	24 (26.1)	4 (36.4)	-

## P127 Both right and left ventricular dysfunction are associated with an increased mortality in patients undergoing prolonged invasive ventilation

### M Chotalia^1^, M Bangash^2^, T Matthews^2^, M Kalla^2^, D Parekh^2^, J Patel^2^

#### ^1^University Hospitals Birmingham, NHS Foundation Trust, Critical Care and Anaesthesia, Birmingham, United Kingdom; ^2^University Hospitals Birmingham, NHS Foundation Trust, Birmingham, United Kingdom

**Introduction:**

In patients undergoing prolonged invasive ventilation we hypothesise that abnormal right ventricular (RV) and left ventricular (LV) function are associated with increased 90-day mortality. Whether changes in LV or RV function could aid in the prognostication of these patients has not been directly studied.

**Methods:**

Patients admitted to the Queen Elizabeth Hospital Birmingham ICU between April 2016 and July 2019 who were intubated and ventilated for more than 7 days and had a formal transthoracic echocardiogram (TTE) whilst in ICU were included. Abnormal RV function was defined by the presence of depressed function, dilated size or moderate to severe risk of pulmonary hypertension. Abnormal LV function was defined by the presence of LV depression (LV ejection fraction £ 45% or grade II or more diastolic dysfunction) or a hyperdynamic LV (formally mentioned in TTE report). Patients who had a neurological cause for prolonged ventilation were excluded. The primary outcome was 90-day mortality. Categorical data is presented as % and analysed using a chi-squared test. Continuous data is presented as median (IQR).

**Results:**

871 patients required prolonged ventilation, of which 350 (40%) had a TTE. Patients were aged 62 (49-75), were 61% male and had a 36% 90-day mortality. The median ventilator days were 13 (6-20) and 77% required a tracheostomy. Abnormal RV function was present in 26% (n=90) and was associated with an increased 90-day mortality compared to normal RV function (68% vs. 25%, RR 2.71 [2.10-3.50], p<0.0001). LV function was abnormal in 27% (n=95) and was associated with an increased 90-day mortality compared to normal LV function (54% vs 28%, RR 1.91 [1.47 – 2.49], p < 0.0001). Abnormal RV function had a trend towards an increased mortality compared to abnormal LV function (68% vs 54%, RR 1.26 [1.00 – 1.60], p = 0.07).

**Conclusions:**

In this study, abnormal RV and LV function were present in a quarter of patients undergoing prolonged ventilation and were associated with an increased mortality.

## P128 Temporary transvenous diaphragm neurostimulation improves volume distribution during mechanical ventilation

### E Rohrs, M Ornowska, T Bassi, K Fernandez, M Nicholas, S Reynolds

#### Simon Fraser University, BPK, Burnaby, Canada

**Introduction:**

Tidal volume delivered by mechanical ventilation (MV) in sedated patients is distributed preferentially to ventral alveoli, causing overdistention and associated collapse in dorsal alveoli, driving volutrauma, atelectrauma and ventilator-induced lung injury [1]. Temporary transvenous diaphragm neurostimulation (TTDN) stimulates diaphragm contraction [2]. When used in synchrony with MV, TTDN encourages increased dorsal ventilation due to the change in pressure gradients with diaphragm contraction, mimicking a more normal physiological pattern. This may improve gas exchange and reduce injury.

**Methods:**

A pilot study was conducted using 50 kg pigs undergoing MV in a mock ICU. Deeply sedated subjects were provided lung-protective volume-control ventilation at 8 ml/kg. TTDN diaphragm contractions were delivered in synchrony with inspiration on every second breath, reducing the ventilator pressure-time-product by 15-20% during MV+TTDN breaths. Tidal volume distribution was recorded in each condition using electrical impedance tomography, and compared to never-ventilated, spontaneously breathing subjects (NV).

**Results:**

Dorsal ventilation changed from 49% during MV breaths to 54% during MV+TTDN breaths, compared to 60% in the NV group (p=0.035). Ventral ventilation changed from 51% during MV breaths to 46% during MV+TTDN breaths, compared to 40% in the NV group (p=0.042, Figure 1).

**Conclusions:**

TTDN diaphragm contraction used as an adjunct to MV yields a more physiological pattern of volume distribution. This translates into less overdistension in the ventral areas and less atelectrauma in the dorsal areas and reduces ventilator-induced lung injury. This technology has the potential to provide a novel method of lung-protective ventilation.

**References:**

1. Schiller HJ et al. Crit Care Med 29:1049-1055, 2001

2. Reynolds SC et al. Am J Respir Crit Care Med 195:339-348, 2017

**Fig. 1 (abstract P128).**
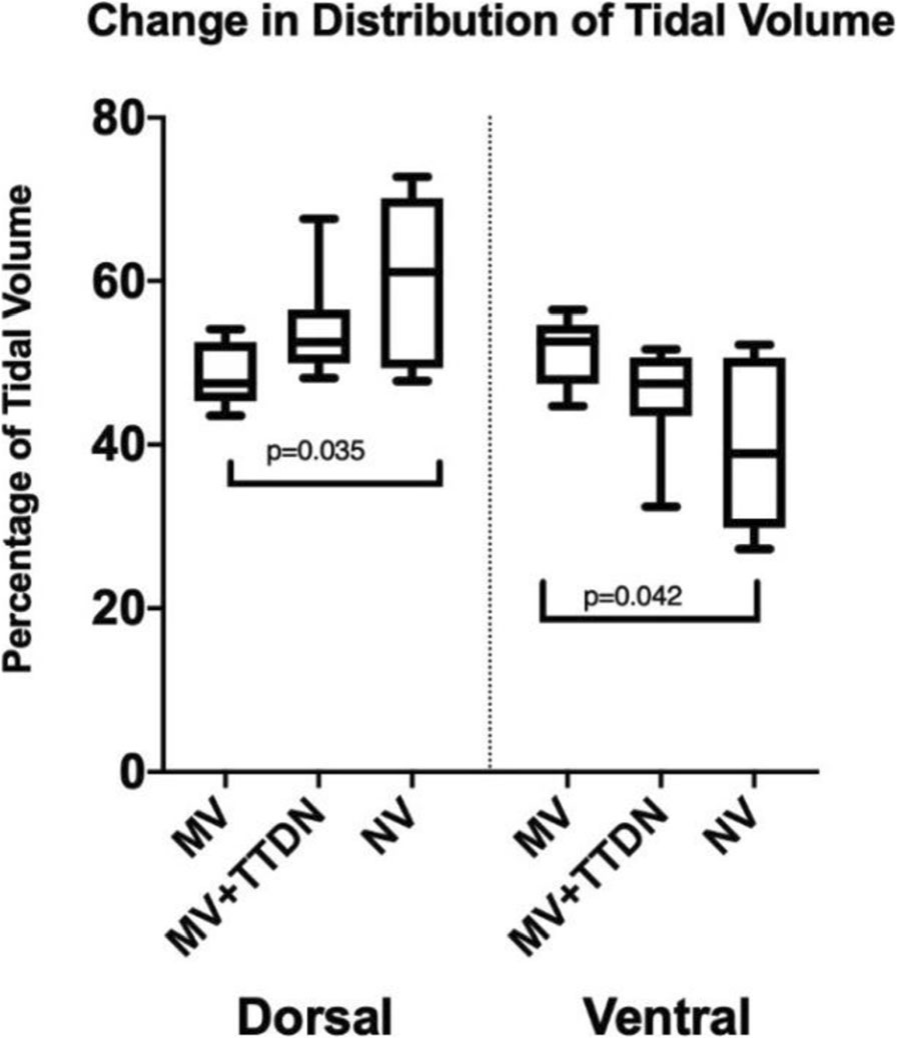
TTDN redistributes tidal volume during mechanical ventilation toward a more physiological pattern

## P129 Assessment of the relation Pswing/tidal volume during spontaneous ventilation test, as a predictor of successful weaning

### F Galiotti^1^, G Pagella^2^, M Carmona^1^, R Paffumi^1^, D Minzer^1^, G Zakalik^1^, P Riera^1^, A Chena^1^

#### ^1^Hospital Luis Carlos Lagomaggiore, UCI, mendoza, Argentina; ^2^Hospital Luis Carlos Lagomaggiore, Terapia intensiva, Mendoza, Argentina

**Introduction:**

By measuring the Pes and its derivatives, we can measure the relationship that exist between the diaphragmatic excursion and the oscillation of the esophageal pressure curve: Pswing (PS) so we infer that, just as with the Pes, the variations of it might be related to a weaning failure [1, 2]. However, no nominal value exists in the bibliography to predict the test result.

**Methods:**

Patients who meet with the inclusion criteria start the weaning process through a test of 30 minutes of spontaneous ventilation, T-Tube (TT). And also the respiratory rate (RR) and the tidal volume (TV). From this analysis, an average PS (APS) is determined for each moment of the test (APS1, initial and APS2, final.).A quotient was obtained in relation to these variables using the value previously obtained (quotient DTV/DPS x100.

**Results:**

A total of 13 patients were included (n=13).Regarding the evolution during TT, 9 (n=9) (69%) were successful, while 4 (n=4) (30.76%) failed When analyzing a rate that relates the variables TV and PS, a quotient was obtained in relation to these variables using the value previously obtained (quotient DTV/DPS) for patients who were successful and who failed, (DTV/DPS)/100 Successful patients presented a value of 18.75 while those of the failure group presented a value of 45.83, (OR 1,2 – 3 p=0.082) (Table 1).

**Conclusions:**

When presenting the relationship between TV and PS through the quotient (DVT/DPS)/100, it is observed a tendency to have a higher quotient among patients who failed versus those who did not fail.

**References:**

1 Akoumianaki E et al. Am J Respir Crit Care Med 189:520-31, 2014.

2 Tobin MJ et al. Weaning from mechanical ventilation. In: Tobin MJ, editor. Principles and practice of mechanical ventilation, 3rd ed. New York: McGraw-Hill; 2012, pp. 1307–1351.

**Table 1 (abstract P129).** Quotient DTV/DPS for patients who were successful and who failed, (DTV/DPS)/100

	DVT	DPS	DVT/DPS * 100
SUCCESS	4.56	0.24	18.75
FAILURE	112.3	2.45	45.8
P value	0.036	0.063	0.082 / Or = 1.2

## P130 Predicting weaning failure in CABG patients: role of echocardiographic evaluation of LVOT-VTI

### E Favilli^1^, G Brizzi^2^, P Bertini^2^, C Vullo^2^, R Baldassarri^2^, L Doroni^2^, D Amitrano^2^, F Guarracino^2^

#### ^1^Scuola di specializzazione in Malattie dell´Apparato Cardiovascolare, Università di Pisa, Pisa, Italy; ^2^Department of Cardiothoracic Anesthesia and Intensive Care Medicine, Azienda Ospedaliero Universitaria Pisana, Pisa, Italy

**Introduction:**

The process of weaning from mechanical ventilation imposes an additional workload on the cardiovascular system, which may result in impaired myocardial function, increase in left ventricular filling pressure and respiratory distress. Among surgical patients, those undergoing heart surgery are particularly susceptible to cardiac dysfunction induced by weaning because of inadequate cardiovascular reserve. The aim of our study was to depict the pathophysiological changes assessed by echocardiography during the steps of weaning and to identify possible predictors of weaning failure (WF).

**Methods:**

We enrolled 34 consecutive patients undergoing isolated coronary artery bypass grafting in our institution. Data were obtained by intraoperative transesophageal echocardiography before sternotomy (T0) and by transthoracic echocardiography at the beginning of weaning (T1) and at the time of extubation (T2). WF was defined as deferral of planned extubation or respiratory failure needing reintubation or non-invasive mechanical ventilation within 48 hours.

**Results:**

WF occurred in 7 patients (20.6%) and involved manifestations of respiratory distress in 5 (14.7%). We found a significant association between left ventricle outflow tract-velocity time integral (LVOT-VTI) and ventricular-arterial coupling measured at T1 and WF, with LVOT-VTI emerging as the best predictor of WF with an area under ROC curve of 0.8669 (Figure 1); an optimal cutoff value of 15 cm provided 100% sensitivity and 71% specificity. Significant increase in E/e’ measured at T2 (13.44 vs 9.96, P 0.02) suggested a cardiac etiology of respiratory distress in patients who failed the weaning trial.

**Conclusions:**

Our study showed that serial assessment of hemodynamic parameters by means of echocardiography is feasible in cardiac surgical patients and can provide insight into pathophysiological changes during weaning. Although these preliminary data need to be confirmed in a larger population sample, LVOT-VTI emerged as a promising predictor of subsequent WF.

**Fig. 1 (abstract P130).**
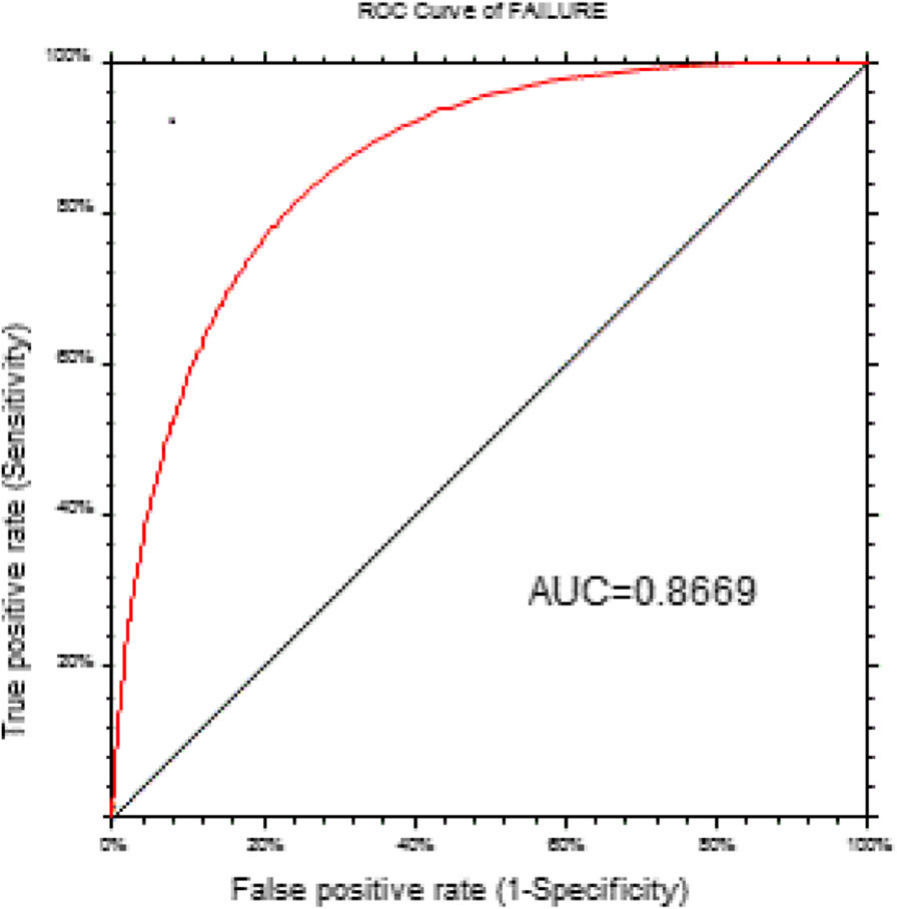
ROC curve of LVOT-VTI measured at the beginning of spontaneous breathing trial for weaning failure

## P131 Compliance with guidelines for respiratory therapy in preclinical emergency medicine

### G Jansen, N Kappelhoff, S Rehberg

#### Protestand Hospital of the Bethel Foundation, Anaesthesiology, Intensive Care and Emergency Medicine, Bielefeld, Germany

**Introduction:**

Current guidelines on pre-hospital emergency ventilation are based on the guidelines for lung protective ventilation in the intensive care unit. The present survey was designed to determine the accordance of actual pre-hospital emergency ventilation by German emergency physicians (GEP) with these recommendations.

**Methods:**

Recommendations include a respiratory rate (RR) between 10-16/min, a tidal volume (vt) between 6-8 ml/kg, a maximum pressure (Pmax) <30 mbar and a positive end-expiratory pressure (PEEP) of 5 mbar. An anonymous web-based questionnaire encompassing 7 questions was sent to GEP from September to December of 2018. GEP were asked to specify their level of education, their preferred ventilation settings and the usually chosen parameters employed to guide mechanical ventilation. Statistical analysis was performed using the Ch²-test with a significance level ≤0.05.

**Results:**

60% of the questionnaires were completed (159/261). 25% of the participants were trainees (Tr), 75% consultants (Co). As target parameters for guidance of ventilation, 87% of the Tr and 91% of the Co use capnometry. The Vt controlled 62% of the Tr and 54% of the Co on the basis of body weight. 81% of the Tr and 81% of the Co reported to control oxygenation using SpO2. Table 1 shows our analysis of the given answers. There were no statistically significant differences between the groups.

**Conclusions:**

Deviations from the guidelines of pre-hospital emergency ventilation settings are common and mainly concern the use of a guideline-compliant PEEP. In addition, recommended target parameters for guidance of ventilation were not applied in a significant proportion of GEP.

**Table 1 (abstract P131).** Guideline-compliance of ventilation parameters

	Trainee	Consultants
	Compliant with guidelines [n(%)]	Compliant with guidelines [n(%)]
RR	39(97)	116(97)
Vt	35(87)	103(86)
Pmax	37(92)	117(98)
PEEP	24(60)	56(47)

RR=respiratory rate; Vt = tidal volume; Pmax=maximum pressure; PEEP = Positive end-expiratory pressure

## P132 Analysis of inflammatory hippocampal cells after 50 hours of lung-protective mechanical ventilation in a preclinical pig model

### T Bassi, E Rohrs, K Fernandez, M Ornoswka, M Nicholas, S Reynolds

#### Simon Fraser University, Physiology, Burnaby, Canada

**Introduction:**

We hypothesize that hippocampal inflammation occurs as a result of mechanical ventilation (MV). Preclinical studies evaluating neuroinflammation in normal-lung MV models have not been reported. Microglia and reactive astrocytes take part in neuroinflammation and are important targets of investigation. They are responsible for modulating neuronal connections, assisting in establishing stronger synaptic connections and maintaining tissue homeostasis.

**Methods:**

Our study investigated the percentage of iba-1-positive cells (microglia), and the percentage of GFAP-positive cells (astrocytes) in the total number of cells counted (positive and negative cells per marker) in the hippocampal tissue of human-size pigs with non-injured lungs after 50 hours of lung-protective MV (MV group), compared with never-ventilated (NV) pigs. The cells were counted by machine learning software IMAGEJ. Lung-protective MV was defined as: driving pressure less than 15 cmH_2_O, tidal volume of 6ml/kg and peak pressure less than 30 cmH_2_O.

**Results:**

Six NV and six MV pigs were used in this study. The percentages of iba-1-positive cells in the hippocampus were 7% (42705 iba-1-positive cells/589888 total cells) and 32% (65655 iba-1-positive cells/205006 total cells) respectively in the NV and MV groups (p<0.01). The percentages of GFAP-positive cells were 9% (35941 GFAP-positive cells/406235 total cells) and 18% (91156 GFAP-positive cells/502195 total cells) respectively in the NV and MV groups (p<0.01). When comparing the percentage of iba-1-positive cells to that of GFAP-positive cells we calculated the ratios of approximately 0.78 (7/9) and 1.78 (32/18) in the NV and MV groups respectively (p<0.01).

**Conclusions:**

Our study demonstrated neuroinflammation in pigs after lung-protective MV. A higher presence of microglia and astrocytes in the MV group indicate an inflammatory hippocampal process as a consequence of lung-protective MV.

## P133 Predicting weaning from mechanical ventilation after lung transplantation

### L Chiscano^1^, M García-de-Acilu^1,2^, J Sacanell^1^, C Laborda^1^, L Sánchez^3^, M Ribas^4^, C Berastegui^5^, R Ferrer^1,6^, O Roca^1,6^

#### ^1^Vall d’Hebron University Hospital, Critical Care Department, Vall d’Hebron Research Institute, Barcelona, Spain; ^2^Departament de Medicina, Universitat Autònoma de Barcelona, Bellaterra, Spain; ^3^Vall d’Hebron University Hospital, Department of Respiratory Medicine, Vall d’Hebron Research Institute, Barcelona, Spain; ^4^Vall d’Hebron University Hospital, Department of Thoracic Surgery, Vall d’Hebron Research Institute, Barcelona, Spain; ^5^Vall d’Hebron University Hospital, Department of Anesthesiology, Vall d’Hebron Research Institute, Barcelona, Spain; ^6^Ciber Enfermedades Respiratorias (CibeRes), Instituto de Salud Carlos III, Madrid, Spain

**Introduction:**

Weaning from mechanical ventilation (MV) is an important issue in the immediate postoperative period following lung transplantation. No predictor of weaning success has been validated in this group of patients. The aim of the study was to establish whether clinical variables and/or the presence of DD may predict extubation failure in LTx patients.

**Methods:**

Prospective observational study including LTx recipients admitted to our ICU from February2017 to January2019, who underwent a spontaneous breathing trial (SBT) using a T-piece for 30 minutes. Clinical variables and arterial blood gas samples were recorded before starting SBT and after 20 minutes on the T-piece. Diaphragmatic excursion (DE) and thickening fraction (DTF) were also assessed using ultrasound(US) after 20 minutes on the Tpiece. US-DD was defined as DE<10 mm or DTF<0.3 of at least one hemidiaphragm. Patients who successfully completed a SBT, defined according to clinical criteria,were extubated. Extubation failure was defined as the need for reintubation within 48h. Results are expressed as medians (IQR) or frequencies (%).

**Results:**

193 LTx recipients were admitted to the ICU, 79 of whom underwent an SBT. 51 were male, and the median age was 58y. Main indications for LTx were interstitial lung disease (43.0%), COPD and cystic fibrosis. 59 were bilateral LTx, and 13 and 7 were left and right unilateral LTx respectively. 69 patients were extubated after SBT and 6 required reintubation within 48h. 53 presented US-DD, though there were no differences between patients who succeeded and those needing reintubation. In contrast, patients who succeeded showed higher PaO2/FIO2 after 20 minutes on the T-piece (Table 1). Similarly, higher reductions in deltaPaO2/FIO2 after 20 minutes on the T-piece were observed in patients who failed.

**Conclusions:**

Oxygenation after SBT performed using a T-piece may predict extubation failure in LTx recipients with successful SBT. US-DD was not associated with the need of reintubation.

**Table 1 (abstract P133).** Clinical and ultrasound variables

		Extubation success (N=63)	Extubation failure (N=6)	P-value
Pre-SBT	RR	17 (15 - 20)	16 (15 - 16)	0.27
	PaO2/FIO2	235 (218-278)	218 (218-220)	0.06
	PaCO2 (mmHg)	40 (38 - 41)	38 (37 - 39)	0.56
20 min T-piece	RR	18 (15 - 23)	23 (16 - 23)	0.43
	PaO2/FIO2	233 (219-265)	188 (188-188)	<0.001
	PaCO2 (mmHg)	40 (38 - 43)	42 (40 - 43)	0.33
	DE<10mm	10 (15.87%)	3 (50%)	0.08
	DTF<0.3	48 (76.2%)	5 (83.3%)	1

SBT: spontaneous breathing trial; RR: respiratory rate; PaO_2_/F_I_O_2_: ratio of partial pressure of arterial oxygen and fraction of inspired oxygen; P_a_CO_2_: partial pressure of arterial carbon dioxide; DE: diaphragmatic excursion; DTF: diaphragmatic thickening fraction

## P134 Descriptive study about the relationship between self-extubation episodes and patient-ventilator interaction

### S Nogales^1^, C De Haro ^1^, M Batlle^2^, L Sarlabous^3^, J Aquino Esperanza^3^, G Gomà^1^, J López Aguilar^3^, L Blanch^3^

#### ^1^Parc Tauli Hospital Universitari, Intensive care unit, Sabadell, Spain; ^2^Fundació Althaia. Xarxa Assistencial Universitària de Manresa., Intensive care unit, Manresa, Spain; ^3^Institut d´investigació i Innovació I3PT, Institut d´investigació i Innovació, Sabadell, Spain

**Introduction:**

To evaluate the relationship between self-extubation and patient-ventilator interaction, among other physiological variables, in order to predict and to prevent these events. Self-extubation (SE) are quality indicators in patients under invasive mechanical ventilations (IMV) and are related with mortality [1].

**Methods:**

Planned secondary analysis of a prospective data base of clinical and physiologic signals of patients receiving IMV. We included SE episodes (2012-2018) with continuous record of ventilator and monitor signals (BCLink BetterCare®). We analysed demographic data, physiological parameters (peripheral oxygen saturation SpO2, heart rate HR, respiratory rate RR and media arterial pressure MAP) and patient-ventilator interaction (asynchrony index AI, ineffective efforts during expiration IEE and double cycling DC). We studied a period of 12 hours prior to the SE episode. We used the Wilcoxon non-parametric test and for a proper analysis a Linear Mixed Effects Model.

**Results:**

We included 21 episodes of SE, mean age 62±13years, 76%men, APACHE II at admission 17±10, 4,6±3,8days under IMV until the episode, reintubation rate 47.6%, ICU stay 20,9±17,6days, ICU mortality 14%. At the time of the SE, 65% were under sedation, 65% with physical restraint. The 67% were in weaning. We observed a trend to increase in SpO2, RR, HR, MAP and asynchronies in the 2-hour period prior to SE episode. We compared these variables from this period with a 2-hour period before and we observed a statistically significant difference in RR, HR, MAP and AI. Clusters of IEE and DC increased its power in the 2-hour period before SE.

**Conclusions:**

In this preliminary study, patient-ventilator interaction, as well other physiological variables, are related with self-extubation episodes. Further analysis will be needed to analyze the value of these variables as a predictive model.

**References:**

1. Danielis M et al. Intensive Crit Care Nurs 47:69-77, 2018

## P135 Postoperative complications and mortality after transcatheter aortic valve implantation: a retrospective, single Belgian center analysis

### E Buts^1^, P Vermeersch^2^, S Verheye^2^, C Convens^2^, B Scott^2^, P Dewulf^1^, P Rogiers^1^

#### ^1^ZNA Middelheim, Intensive Care, Antwerp, Belgium; ^2^ZNA Middelheim, Cardiology, Antwerp, Belgium

**Introduction:**

The purpose of this study was to review the mortality and early postoperative complication rates after TAVI (transcatheter aortic valve implantation) for severe aortic stenosis in an interventional cardiology center in Antwerp, Belgium with more than 10 years of experience. All patients were postoperatively admitted to the ICU for judicious monitoring. Results were compared to large international registries.

**Methods:**

A retrospective data analysis by medical record review of 242 patients who underwent a TAVI procedure between april 2008 - april 2019 was carried out. A literature search was executed to compare the results with current published data. Endpoints were mortality (72 hours, 1 week, 30 days, 1 year), bleeding, vascular complications, definitive pacemaker implantation, stroke, conversion and cardiac tamponade. Standardized criteria according to the Valve Academic Research Consortium II recommendations for studies evaluating TAVI were used [1].

**Results:**

Vascular access was predominantly transfemoral (90.1%), compared to subclavian (7%), carotic (1.7%) and direct aortic (1.2%) approaches. Mean age was 82.6 (range 61-98) years old, with 51.2% female patients. Mortality rates were 1.2, 2, 4.8 and 15.9% at 72 hours, 1 week, 30 days and 1 year respectively. Life-threatening bleeding occurred in 2.9%, major in 10.7% and minor bleeding in 3.3%. The major and minor vascular complication rates were 12.4% and 5.8%. 97.7% was access site related. There were no patients with aortic dissection. The need for definitive pacemaker implantation was 18%. Incidence of stroke and cardiac tamponade was 1.7 and 3.3% respectively. Conversion to open sternotomy was necessary in 0.8%.

**Conclusions:**

The data presented in this study show that our results are in accordance with the literature with favorable mortality and early postoperative complication rates and support that this procedure is an excellent alternative for surgery in the elderly patients.

**References:**

1. Kappetein AP et al. Eur J Cardiothorac Surg 42:S45-60, 2012

## P136 Deterioration of pulmonary hypertension after TAVI predicts mid- to long-term poor prognosis

### H Yamamoto^1^, S Kayama^1^, Y Watanabe^2^, M Sugi^3^, T Ikeda^4^, S Sawamura^1^

#### ^1^Teikyo University Hospital, Anesthesia, Tokyo, Japan; ^2^Teikyo University Hospital, Cardiology, Tokyo, Japan; ^3^Tokyo Medical University Hachioji Medical Center, Anesthesia, Tokyo, Japan, ^4^Tokyo Medical University Hachioji Medical Center, Specific intensive care, Tokyo, Japan

**Introduction:**

It is reported that patients with pulmonary hypertension (PH; systolic pulmonary arterial pressure (sPAP)≥35 mmHg)) have frequent cardiac complications after transcatheter aortic valve implantation (TAVI). PH often gets worse in some patients despite the normal cardiac function after TAVI. No studies have ever examined prognosis after TAVI in patients with or without worsening of PH. Therefore, we retrospectively examined the frequency of mid- to long-term heart failure and cardiac death in patients with and without deterioration of PH after TAVI.

**Methods:**

Among 113 patients who underwent TAVI at our hospital between February 2014 and March 2016, we analysed 27 patients with PH (sPAP≥35 mmHg) before surgery. sPAP was measured in transthoracic echocardiography before and within 1 week after TAVI. Patients were divided into two groups according to whether sPAP worsened/did not change or improved after TAVI. We examined the frequency of admission due to heart failure or cardiac death (death caused by heart failure, angina, or myocardial infarction) during the period of 3 years after TAVI.

**Results:**

PH worsened or did not change after TAVI in 9 patients, while it improved in 18 patients. The left ventricular ejection fraction measured within 1 week after TAVI showed no difference between the two groups (56.6±11.9% vs 58.4±10.0%, p=0.71). The worsened/ no change group was higher in frequency of admission due to heart failure (logrank; p<0.05) and cardiac death (logrank; p<0.04).

**Conclusions:**

Despite successful treatment for AS by TAVI, the frequency of heart failure and cardiac death was higher in patients who did not show improvement of PH after TAVI, even in the absence of cardiac function decrease. Vigorous intervention for PH worsening after TAVI may be helpful to improve prognosis.

## P137 The anti-thrombotic effects of different P2Y12 inhibitors in the management of acute ST elevation myocardial infarction

### R Quarry^1^, M Lawrence^2^, A Sabra^2^, D Obaid^2^, A Chase^3^, D Smith^3^, J Whitley^2^, S Pillai^3^, K Morris^4^, PA Evans^2^

#### ^1^Morriston Hospital, Emergency Department, Swansea, United Kingdom; ^2^Swansea University, Welsh Centre for Emergency Medicine Research, Swansea, United Kingdom; ^3^Morriston Hospital, Welsh Centre for Emergency Medicine Research, Swansea, United Kingdom; ^4^Cardiff Metropolitan University, Welsh Centre for Emergency Medicine Research, Cardiff, United Kingdom

**Introduction:**

There are several different anti platelet drugs that can be used to treat acute cardiac events. Currently there are no effective markers that can assess how these drugs modify coagulation profile and quality. A new functional biomarker that measures Fractal dimension (df ) and clot formation time (TGP) has been developed [1]. df quantifies clot microstructure whereas TGP is a real-time measure of clotting time. We aimed to validate df and TGP in ST elevation myocardial infarction (STEMI) and assess the effect of two P2Y12 inhibitors which have different pharmacological mechanisms: clopidogrel and ticagrelor.

**Methods:**

We prospectively recruited 72 STEMI patients in the emergency setting. Venous blood samples were collected 12 hours after admission, following treatment with either ticagrelor or clopidogrel, in accordance with the local guidelines at the time. The blood samples were tested using the df and TGP biomarker, platelet aggregometry, clot contraction and standard markers of coagulation.

**Results:**

36 patients received clopidogrel and 36 received ticagrelor. The df for clopidogrel was higher than ticagrelor (1.75±0.05 vs 1.73±0.06, p=0.18 which corresponds to a decrease in clot mass of 20% Figure 1) and the TGP was reduced (205±91sec vs 257±89 sec, p=0.06 a 20% reduction in time).

**Conclusions:**

The results of the study suggest that clopidogrel is less powerful in its effects on clotting characteristics compared to ticagrelor. Blood from patients receiving clopidogrel formed quicker and denser clots. This would suggest the risk of secondary events or stent occlusion is lower in those patients on ticagrelor, highlighting that df and TGP may be important in identifying patients at risk of future thrombotic events, the study is ongoing and will investigate the long term outcome in these patients.

**References:**

1. Knowles RB et al. Platelets 29:162-170, 2018

**Fig. 1 (abstract P137).**
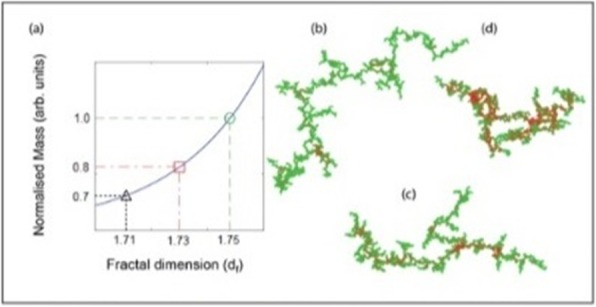
Visual representation of the relationship between clot microstructure and clot mass

## P138 Management of new onset atrial fibrillation in a district general hospital intensive care unit

### A Munro, M Yousif, W Du, R Sarkar, N Divekar

#### Medway Maritime Hospital, Intensive Care Unit, Gillingham, United Kingdom

**Introduction:**

New onset atrial fibrillation (NOAF) during critical illness frequently resolves prior to discharge. However long-term risks of NOAF (i.e. heart failure, ischemic stroke and death)remains high [1]. Previous studies noted that nearly half of NOAF cases did not have diagnosis recorded [2]. Addressing this may reduce post critical illness mortality by increasing AF surveillance post intensive care (ICU) discharge.

**Methods:**

Retrospective data was collected from an electronic health record for ICU admissions over a 10 month period from 1^st^ of January to 31^st^ of October 2019 at Medway Maritime Hospital. Data collected included; demographics, onset, rate, possible precipitants, attempted electrical or chemical cardioversion, rate control, anticoagulation, rhythm on discharge from ICU and hospital discharge medication. It was recorded if NOAF was documented in ICU and Hospital discharge summaries.

**Results:**

Over 10 months 429 patients were admitted to ICU. 37 patients developed new onset AF with an average age of 69.2 (±10.3) and 59.4% male. Patients with NOAF had an in-hospital mortality of 43.2% compared to 29.8% . Beta blockers were used in 38% and amiodarone in 59%. 23 patient’s with NOAF survived ICU admission (62%). 13.5% remained in AF on discharge of which 1 patient was discharged from ICU on therapeutic anticoagulation (2 total, 11%). Of those not anticoagulated 19 had a Cha2ds2-VASc of >= 2. On ICU discharge, 23 patients had NOAF. 35% episodes documented on ICU discharge summaries. Thirty three per cent patient had documentation about the reason for choosing rate control approach. On hospital discharge only 28% had documentation of NOAF episode on their hospital discharge summaries.

**Conclusions:**

Need to implement an automated referral to cardiology to assess long term anticoagulation for patients with NOAF with CHA2DS2-VASc score >=2. For clear documentation of NOAF episode in ICU discharge summaries.

**References:**

1. Walkey AJ et al. Chest 146: 1187-1195, 2014

2. Moss et al. Critical Care Medicine 45: 790-797,2017

## P139 Assessing the role of cardiac biomarkers in the emergency department in a Moroccan hospital

### S Abouradi^1^, R Benmalek^1^, R Habbal^1^, M Mouhaoui^2^

#### ^1^Cardiology Department, CHU Ibn Rochd, Casablanca, Cardiology, Casablanca, Morocco; ^2^Emergency Department, CHU Ibn Rochd, Emergency Department, Casablanca, Morocco

**Introduction:**

A biomarker is defined as a measurable indicator of some biological state or condition. Combined with a good clinical evaluation, they can enable an early and safe diagnostic, thus a faster management for the patient. Cardiac biomarker testing is not indicated in routine in the emergency department (ED) because of low utility and high possibility of false-positive results. However, current rates of testing are unknown. The aim of our study was to evaluate the importance of measuring cardiac biomarkers especially Troponins, D-dimer, and B-type natriuretic peptide in our daily practice, and to identify the latest recommendations for a better use of these biomarkers in the diagnostic and therapeutic approaches.

**Methods:**

We conducted a prospective observational study, over a 13 months periods performed in the ED of the university hospital center Ibn Rochd, Casablanca, Morocco, including all patients admitted during our study period and having a blood test for at least one biological marker. The dataset was analyzed by SPSS statistics 21.0.

**Results:**

A total of 182 patients was enrolled. Troponins were tested in 85.3% patients (High sensitive in 49.5% and troponin I TnI in 35.8%), D-dimer in 30.9%, BNP 19% and NT pro BNP in 9.5% of cases. The diagnostic impact was significant in 94.4% of cases for troponins, 84.6% of cases for D-dimer and 87.5% for BNP. The therapeutic impact was considered important in 80.6% cases for troponins, 69.2% for D-dimer and 87.5% for BNP.

**Conclusions:**

Cardiac biomarkers have an important role in the ED, not only do they confirm the diagnosis (including the role of troponins in ACS) but also eliminate others (with a strong negative predictive value of D-dimer for thromboembolic disease) and prove the cardiopulmonary origin of acute dyspnea (the significant place of BNP in confirming the diagnosis of acute heart failure).

## P140 A multicenter study on the comparison of inter-rater reliability of a new and the original HEART score among emergency physicians from three Italian emergency departments

### N Parenti^1^, ML Bacchi Reggiani^2^, F Numeroso^3^, L Bonfanti^3^, G Farina^4^, V Pezzilli^5^, C Campanella^6^, G Cervellin^3^, M Cavazza^5^, V Stanghellini^5^

#### ^1^Largo Nigrisoli, Internal Medicine, Bologna, Italy; ^2^University of Bologna Alma Mater, Biostatistics, Bologna, Italy; ^3^University Hospital of Parma, Emergency Dept, Parma, Italy; ^4^University of Bologna Alma Mater, Emergency Dept, Bologna, Italy; ^5^University of Bologna Alma Mater, Internal Medicine, Bologna, Italy; ^6^Nova Southeastern University USA, Internal Medicine, Davie, Florida, United States

**Introduction:**

The HEART (based on History,ECG,Age,Risk Factors,Troponin) score is a valid tool to stratify the ACS in chest pain. But some reports suggest that its reliability could be low for heterogeneity in the assignment due to the subjective interpretation of the History. We used the Chest Pain Score for the “History”. In this study we compare the reliability of the new HEARTCPS and original HEART.

**Methods:**

This is a multicenter retrospective study conducted in 3 Italian ED between July and October 2019 using clinical scenarios. Ten physicians were included after a course on HEART and HEARTCPS score. We used 53 scenarios which included clinical and demographic data. Each participant independently assigned scores to the scenarios using the HEART and HEARTCPS. We tested the interrater agreement using the kappa-statistic (k), the confidence intervals are bias corrected ; we used Stata/SE 14.2 statistical software . A p-value of <0.05 defines statistical significance.

**Results:**

The overall inter-rater reliability was good for HEART and HEARTCPS: Kappa =0.63 (CI 95%;0.57–0.72)and 0,65(CI95%;0.63 - 0.67); with good agreement among all the class of risk for HEARTCPS but moderate in the medium class for HEART .

We found significant differences of inter-rater reliability among the senior and junior physicians who used the HEARTCPS:K=0.56(CI95%;0.52–0.57)and 0.75(CI95%;0.70-0.79). HEARTCPS score increased its History inter-rater reliability specially among the junior physicians from K=0.35 (CI 95%; 0.27-0.43) to k=0.69(CI 95%;0.62-0.71).The Junior physicians seem to be more reliable than senior with the HEARTCPS:k=0.75 (0.71-0.79) vs K=0.56 (CI95%;0.52-0.57).

**Conclusions:**

The HEARTCPS showed inter-rater reliability better than original HEART among the medium class of risk and the junior group. It could be proposed to young doctors to stratify the ACS risk of chest pain. Limit: we used scenarios rather than real patients.

## P141 A hybrid approach as treatment for coronary artery disease: endo-CABG or PCI first, does it matter?

### V Dekoninck^1^, A Yilmaz^2^, J Dubois^3^, JP Ory^3^, J Vandenbrande^3^, B Stessel^3^

#### ^1^Jessa, Intensive Care & Anesthesiology, Hasselt, Belgium; ^2^Jessa, Cardiothoracic Surgery, Hasselt, Belgium; ^3^Jessa, Anesthesiology & Intensive Care, Hasselt, Belgium

**Introduction:**

The aim of this study is to discuss the short-term results of a hybrid approach combining minimally invasive endoscopic CABG (EndoCABG) with a percutaneous coronary intervention (PCI). To bypass the disadvantages and potential complications of conventional CABG via median sternotomy, we developed the EndoCABG technique to treat patients with single- and multi-vessel coronary artery disease (CAD). This procedure is performed with three 5-mm thoracic ports and a mini-thoracotomy utility port (3 cm) through the intercostal space. This technique can be combined with PCI: the hybrid approach. The sequence of the 2 procedures (EndoCABG followed by PCI or vice versa) may result in different outcomes.

**Methods:**

From 02/2016 to 12/2017 data from 81 consecutive patients scheduled for a hybrid technique at Jessa, Belgium, were prospectively entered into a customized database. This database was retrospectively reviewed. Subgroup analysis was performed to compare outcomes of patients who first received EndoCABG with patients who first received PCI. A p-value < 0.05 is considered significant, a p-value < 0.1 is considered as a trend toward significance.

**Results:**

Four patients underwent revision surgery and 2 patients died within the first 30 days. In 79 patients the left anterior descendens artery (LAD) was grafted with the left internal mammary artery (LIMA), the right coronary artery (RCA) was the most stented vessel using PCI. Patients first treated with PCI received more units of fresh frozen plasma after EndoCABG compared to those who were first treated with EndoCABG (p=0.03). There was also a trend toward significant more transfusion of packed cells in this small subgroup (p=0.07).

**Conclusions:**

The hybrid approach is a feasible technique as a treatment option for patients with multi-vessel CAD. If CABG follows the PCI, patients are more likely to receive transfusion. A possible explanation could be the need for dual antiplatelet therapy prior to surgery in this group, but this needs further investigation.

## P142 Prognostic difference between troponin elevation meeting the MI criteria and troponin elevation due to myocardial injury in septic patients

### A Shilova^1^, D Shchekochikhin^2^, M Gilyarov^3^, A Nesterov^4^, A Svet^5^

#### ^1^Moscow City Univercity hospital #1 n.a. Pirogov, ICCU, Moscow, Russia; ^2^Moscow City Univercity hospital #1 n.a. Pirogov, Cardiology, Moscow, Russia; ^3^Moscow City Univercity hospital #1 n.a. Pirogov, Cardiology, Moscow, Russia; ^4^Moscow City Univercity hospital #1 n.a. Pirogov, Cardiology, Moscow, Russia; ^5^Moscow City Univercity hospital #1 n.a. Pirogov, Management, Moscow, Russia

**Introduction:**

Troponin T (cTnT) elevation in critically ill patients is common and is associated with poor outcome. Using common assays, 40-50% of patients in the ICU will have elevated troponin level. Our aim was to determine whether there is any prognostic difference between troponin elevation meeting the MI criteria (rise and fall more than 20% together with Echo and ECG new abnormalities) and troponin elevation due to myocardial injury in septic patients.

**Methods:**

We enrolled 101 patients with sepsis and mean SOFA score 5,2 respectively in which cTnT level was measured more than once and analyzed there ECG and Echo findings. Patients were classified into three groups:definite MI (rise and fall cTnT ≥ 20% and contemporaneous changes on ECG and/or Echo),possible MI (rise and fall cTnT ≥ 20% and no other findings),myocardial injury (cTnT rise less than 20%)

**Results:**

Data from 101 patients were analyzed (49% female; mean age 61.9 (SD 16.9)). A total of 101 patients had at least one elevated cTnT more than 0.03 mkg/l. In 71 (70%) of patients cTnT level rised more than 20% from the first elevated measurement.64 (63%) of patients met MI criteria considering new ECG and Echo findings. The overall mortality rate in all patients was 53.9%.The mortality rate didn’t differ significantly in three groups: in the definite MI group 62.4%, in the suspected MI group 52%, in the non MI cTnT elevation group 56,4%, p=0,6. Coronary angiography was performed in 46 (73%) of patients from the definite MI group,PCI was performed in 18 (39%) of patients. The mortality rate in the invasive group was not significantly lower comparing to the nonivasive group 29% vs 37,8%, p=0,06. Bleeding complications were significantly more frequent in the definite MI group 13% vs 7% and 8% respectively

**Conclusions:**

cTnT level elevation is associated with poor outcome regardless coronary or non coronary injury. Myocardial revascularization may be beneficial in patients with sepsis and definite MI, but it is also associated with increased bleeding risk.

## P143 Diagnostic interest of "Marburg Heart Score" in patient consulting the emergencies department for acute chest pain

### NE Nouira^1^, M Lehyeni^1^, A Lahouegue^1^, K Hamzaoui^1^, D Hamdi^1^, M Boussen^2^

#### ^1^Mongi Slim Academic Hospital, Emergency Department, Tunis, Tunisia; ^2^Mongi Slim Academic Hospital, Tunis, Tunisia

**Introduction:**

Chest pain is a common reason for emergency department visits, although this primarily refers to Acute Coronary Syndrome (ACS), this symptom may be frequently related to other non-ischemic etiologies. The aim was to validate the Marburg Heart Score as a tool to exclude coronary artery disease in emergency department patients with non-traumatic acute chest pain.

**Methods:**

a prospective, observational, descriptive and analytic cohort study conducted in the emergency department, from February 1st to March 31st, 2019, collecting patients consulting for nontraumatic acute chest pain, the "Marburg Heart" score was calculated for all these patients. Telephone contact was made after 6 weeks to look for an ischemic cardiovascular event.

**Results:**

We included 171 patients. The mean age was 57 +/- 13 years, the sex ratio was 0.86. The majority of the patients (78.9%) consulted directly to the emergency department, 21.1% were referred by a primary care physician. The median time to consultation after the onset of chest pain was 24 hours. High blood pressure was the most common risk factor (43.9%), followed by smoking (31%), diabetes (24.8%) and dyslipidemia (23.4%). Thirty-five patients (20.5%) had already coronary heart disease, ECG was pathological in 19.3% of patients, 8 patients had an ACS with ST segment elevation. At six weeks, 20.6% of the patients had an acute coronary event. According to the patients' answers on the 5 questions of the Marburg Heart score. The area under the ROC curve of this score was 0.78 with a negative predictive value of 87.2%;

**Conclusions:**

The "Marburg Heart Score" is a simple, valid and reproducible clinical score with a discriminatory power to rule out the diagnosis of coronary artery disease from the first contact with the patient presenting for chest pain in emergencies.

## P144 Fluid management in abdominal aortic aneurysm surgery in elderly patients

### D Lončar Stojiljković^1^, MP Stojiljkovic^2^

#### ^1^Institut Dedinje, Anesthesia and Intensive Care, Belgrade, Serbia; ^2^Institut Dedinje, Belgrade, Serbia

**Introduction:**

The abdominal aortic aneurysm (AAA) surgery is a complex procedure in elderly patients with high cardiovascular risk. Anesthesiological techniques should play special attention to the volume status during cross-clamping as well as to the blood loss. Goal directed fluid therapies (GDT) in AAA surgery in elderly patients decrease the perioperative morbidity and mortality [1]. Aim of this study is to investigate administration of fluid-based on either a GDT approach or a control method (fluid administered based on static preload parameters and traditional hemodynamic) in all phases of AAA surgery and especially in the phase of clamping and de-clamping.

**Methods:**

A total of 30 patients ASA III, randomly scheduled for elective, open AAA surgery were included in this clinical trial. They were randomly assigned to two groups I – GDT with targeting stroke volume variation (SVV) and II - Control group where fluids were administered at the discretion of the attending anaesthesiologist. In both these groups hemodynamic parameters, central venous pressure (CVP), temperature, blood loss and diuresis were registered during the operation and 48 hours postoperatively. Each group was assessed for postoperative complications.

**Results:**

GDT group received less fluids and had a higher cardiac index (CI) (3.9± 0.6 vs. 2.9± 0.8 l/minute per m^2^, p < 0.01) and stroke volume index (55.1 ± 5.4 vs. 35.1 ± 5.8 ml/m^2^, p < 0.01) than the control group. There were significantly fewer complications in the intervention than control group (3 vs. 9, p = 0.02).

**Conclusions:**

GDT fluid administration enables less use of fluids, improved hemodynamic and fewer postoperative complications in elderly patients undergoing AAA surgery.

**References:**

1. Funk DJ et al. Crit Care 19:247, 2015

## P145 Should preoperative antihypertensive agents be hold in high risk hypertensive patients undergoing major noncardiac surgery with neuraxial block?

### S Kongsayreepong^1^, W Chaisi^2^

#### ^1^Siriraj Hospital, Anesthesiology & Critical Care, Bangkok, Thailand; ^2^Siriraj Hospital, Bangkok, Thailand

**Introduction:**

With the benefit of neuraxial block in major noncardiac surgery, but serious cardiac events’re reported in high risk pts receiving preop beta-adrenergic blocking agent. The aim of this study was to study predictors of intraoperative cardiac complications in pts underwent major noncardiac surgery with neuraxial block.

**Methods:**

This prospective observational study was done in 360 high risk for cardiac complication pts, underwent elective major noncardiac surgery with neuraxial block [spinal, thoracic (TE) or lumbar epidural (LE) anesthesia] alone or combined with GA & admitted to the SICU during Jan 2017-Sep 2019. Pts with ESRD or underwent arterial vas surgery were excluded. Study data included: pts demographic,comorbidities,ASA class, preop hypertensive med (beta adrenergic blocking agent, ACEI/ARB, vasodilator), intraoperative cardiac complications [significant hypotension (SBP<90 mmHg or MAP<65 mmHg>10 mins) requiring fluid resuscitation or inotropic agent, serious cardiac arrhythmias needed medication or intervention, PMI, cardiac failure, cardiac arrest, cardiac death), within 72 hours postoperative organ dysfunction (AKI, stroke) & 30days mortality.

**Results:**

Among the studied pts (age 65+16yrs), 72% was ASA III, 54% had hx of HTN. 64% underwent major abdominal surgery, 21%, 8% & 72% received spinal, spinal plus GA and TE or LE plus GA subsequently. 79% had serious intraop cardiac complications (80%, 15% & 4% had significant hypotension, PMI & cardiac arrest subsequently), 39%, 3% & 11% had AKI, ischemia stroke&delirium subsequently. 30 days mortality was 13%. From multivariate variate analysis showed age>75 yrs, hx of HTN, preop beta-adrenergic blocking agent, vasodilator, combined beta-adrenergic blocking agent & vasodilator or ACEI/ARB, high dose local anesthetic agent were significant predictors of intraop serious cardiac complications.

**Conclusions:**

Further study about decrease or withhold preop antihypertensive med in high risk patient undergoing major noncardiac surgery with central neuraxial block is warranted.

## P146 Clinical importance of ultrasonographic assessment of skeletal muscle mass in patients after cardiothoracic surgery

### V Raidou^1^, S Dimopoulos^2^, F Chatzivasiloglou^1^, D Elaiopoulos^2^, A Robola^2^, D Markantonaki^2^, E Lyberopoulou^2^, A Marathias^2^, S Nanas^1^, A Karabinis^2^

#### ^1^National and Kapodestrian University of Athens, Clinical Ergospirometry, Exercise and Rehabilitation Laboratory, Athens, Greece; ^2^Onassis Cardiac Surgery Center, Cardiac Surgery ICU, Athens, Greece

**Introduction:**

Ultrasonography is a valid diagnostic tool, used to measure changes of muscle mass. The aim of this study was to investigate the clinical value of ultrasound-assessed muscle mass, in patients undergoing cardiothoracic surgery that present muscle weakness postoperatively.

**Methods:**

For this study, 221 consecutive patients were enrolled, following their admission in the Cardiac Surgery Intensive Care Unit (ICU) within 24 hours of cardiac surgery. Ultrasound scans, for the assessment of quadriceps muscle thickness, were performed every 48 hours for 7 days. Muscle strength was also evaluated in parallel, using the Medical Research Council (MRC) scale.

**Results:**

Of the 221 patients enrolled, ultrasound scans and muscle strength assessment were performed in 165 patients. The muscle thickness of rectus femoris (RF), was slightly decreased by 2.18% ([95%CI: -0.21; 0.15], n=9; p=0.729) and the combined muscle thickness of the vastus intermedius (VI) and RF decreased by 3.5% ([95% CI: -0.4; 0.22], n=9; p=0.530). Patients whose combined VI and RF muscle thickness was below the recorded median values (2.5cm) on day 1 (n=78), stayed longer in the ICU (47 ± 74 vs 28 ± 45 hours, p = 0.015). Patients with MRC score ≤ 48 on day 3 (n=7), required prolonged mechanical ventilation support compared to patients with MRC score ≥ 49 (n=33), (44 ± 14 vs 19 ± 9 hours, p = 0.006).

**Conclusions:**

The use of muscle ultrasound seems to be a valuable tool in assessing skeletal muscle mass in critically ill patients after cardiothoracic surgery. Moreover, the results of this pilot study showed that muscle wasting of patients after cardiothoracic surgery is of clinical importance, affecting their stay in ICU.

## P147 Prediction of cardiac risk after major abdominal surgery

### S Musaeva, I Tarovatov, A Vorona, I Zabolotskikh, N Doinov

#### Kuban State Medical University, Anesthesiology and Intensive Care, Krasnodar, Russia

**Introduction:**

The aim is to assess the incidence of cardiovascular incidents in major abdominal surgery [1] using the revised Lee index.

**Methods:**

A study was conducted of 144 elderly patients who underwent major abdominal surgery in the Krasnodar Regional Clinical Hospital No. 2 under combined anesthesia. In the preoperative period, the risk of cardiovascular incidents was assessed using the revised Lee index and the functional status was assessed by MET. Depending on the Lee index, 3 groups were identified: group 1 (n = 69) - low risk (index value - 1), group 2 (n = 52) - intermediate risk (index value -2); group 3 (n = 23) - high risk (index value> 3). We estimated the incidence of critical incidents in groups: hypo-, hypertension, arrhythmias, and bradycardia.

**Results:**

In the general population, cardiac risk was 2.2 ± 0.7 points; functional status - 7.7 ± 1 MET. The greatest number of critical incidents was recorded in patients with high risk (58.4%), the smallest - in patients with low risk (9.1%), in patients with intermediate risk - 26.5% (n <0, 05 between groups according to Chi-square criterion). In the structure of critical incidents, hypotension was most often encountered - in 62 (43%) patients, while some patients revealed several incidents from the circulatory system (n = 116). Overall, the Lee scale showed good prognostic ability (AUROC = 0.81) in predicting hemodynamic incidents.

**Conclusions:**

The revised Lee index is a useful tool to help assess the risk of cardiovascular incidents and determine patient management tactics in the perioperative period.

**References:**

1. Veyler RV et al. Anesteziol Reanimatol 61:352-356, 2016

## P148 Cognitive dysfunction in rats after minor and major surgery

### V Sharipova, A Valihanov, A Alimov

#### Republican Research Centre of Emergency Medicine, Anesthesiology, Tashkent, Uzbekistan

**Introduction:**

Postoperative cognitive dysfunction (POCD) remains an unresolved problem due to lack of consensus on its etiology and pathogenesis. Some believe that POCD is the result of the direct toxic effect of general anesthetics on the nervous system. Others claim that surgical trauma activates proinflammatory factors that induce neuroinflammation.

**Methods:**

Wistar rats were allocated into 2 groups: 1-minor surgery (n=20), 2-major surgery group (n=20). After 5 days of handling and habituation rats undergone surgery under isoflurane general anesthesia (2 vol.%). Group 1 rats underwent laparotomy with gentle gut massage followed by wound closure. Rats in group 2 undergone left side nephrectomy. Starting from the 4th postoperative day spatial memory in rats was studied in Morris Water Maze which is a cylinder metal pool with a diameter of 1.5 and a height of 0.5 m filled with water (temp.26±1^o^C) up to half. It has a platform with a diameter of 12 cm and a height of 1 cm below the water level. Testing was preceded by a training stage, which included 8 sessions daily for 4 days. Thus, rats developed spatial memory to the location of the platform. On the 5th day of the study test stage was conducted to assess spatial memory: rats were launched from 3 points into maze without platform and data were recorded for 60 seconds at each session. Time spent on the target quadrant (TTQ) and the number of target area crossings (TAC) were registered. A second test was conducted 14 days after the first test to evaluate long-term spatial memory.

**Results:**

The duration of surgery and anesthesia did not differ significantly between groups. There was a significant difference between groups in average TTQ and TAC in test 1 (Table 1). In test 2 minor surgery group showed better results but they were less significant.

**Conclusions:**

Major surgery is associated with a more pronounced deterioration of spatial memory in rats in early postoperative period compared to minor surgery.

**Table 1 (abstract P148).** Data are shown as Mean±SD, TTQ-Time spent on the Target Quadrant, TAC- Target Area Crossings

	Minor surgery (n=20)	Major surgery (n=20)	p
Surgery duration, min	22.6 ± 6.3	20.1 ± 3.7	0.134
Anesthesia duration, min	41.2 ± 8.5	36.3 ± 8.2	0.071
TTQ-1, sec	59.5 ± 12.1	51.2 ± 13.7	0.049
TTQ-2, sec	57.8 ± 9.7	52.4 ± 12.5	0.135
TAC-1	4.9 ± 2.7	3.3 ± 1.9	0.036
TAC-2	4.6 ± 2.1	3.4 ± 2.3	0.093

## P149 Cardiac inflammatory markers in ICU patients with myocardiac ischemia after non cardiac surgery (a pilot study)

### P Manthou^1^, G Lioliousis^2^, P Vasileiou^3^, G Fildissis^1^

#### ^1^National Kapodistrian University of Athens, Athens, Greece; ^2^National Kapodistrian University of Athens, General Thoracic Hospital ´´Sotiria´´, Athens, Greece; ^3^National Kapodistrian University of Athens, University of Athens, Athens, Greece

**Introduction:**

Patients with known coronary artery disease have higher perioperative risk for myocardial ischemia [1, 2]. Mortality is frequent following cardiac ischemia in the intensive care unit (ICU) after non-cardiac surgery.

**Methods:**

The first group includes patients admitted to the Intensive Care Unit for post-operative follow-up without myocardiac ischemia in the first 24 hours. The second group includes patients with myocardiac ischemia postoperatively and needs intensive care monitoring. Cardiac risk assessment was made with the Lee Index,hemorrhagic risk assessment with the HAS-BLED bleeding score and thrombotic risk assessment with CHA2DS2-VASc score. Postoperatively, pathological test values such as BNP, troponin, CRP, calcitonin were estimated. The Sequential Organ Failure Assessment (SOFA) systeme was used to assess sepsis. The Nursing Activity Score (NAS) scale was used to measure the workload of various nursing activities in the ICU.

**Results:**

According to the pilot study, the sample consists of 35 patients. 31.4% had myocardial ischemia. The Lee index was significantly higher in patients with myocardial ischemia. The duration of hospitalization, the high dose of vasoconstrictive drugs, the length of stay in the ICU, the duration of mechanical stay and the nursing workload were higher in patients with myocardial ischemia. CK-MB and troponin levels differed significantly between the two groups. Creatinine, bilirubin and BNP during the 24 hours were significantly higher. Patients with myocardial ischemia had significantly higher mortality.

**Conclusions:**

Cardiac risk assessment, HAS- BLED score and CHA2DS2-VASc score in combination with cardiac enzymes such as troponin could predict myocardiac ischemia in severely ill ICU patients.

**References:**

1 2014 ESC/ESA. Eur Heart J 35: 2383–2431, 2014.

2 Spyropoulos JD et al. Blood 120: 2954-2962, 2012

## P150 Airway complications after thyroid surgery

### V Artemenko^1^, A Budnyuk^2^

#### ^1^MC MEDICAP, Anesthesia&ICU, Odessa, Ukraine; ^2^Odessa Regional Hospital, ICU, Odessa, Ukraine

**Introduction:**

According to the literature an airway complication followed thyroid gland surgery are: difficult trachea intubation, tracheomalacia, postextubation stridor and bleeding [1,2]. Most common cause of death was problem with respiration and airway obstruction [3]. Subsequent hypoxia could require emergency airway and even tracheostomy [3]. Aim of our study was to determine the most common of airway complications and their association with type of surgery in our region.

**Methods:**

The retrospective cohort study included 400 pts., (369 women, 31 men) was performed in Odessa regional Hospital, Oncology Centre Odessa. There were three types of patients: with euthyroid goiter - 170 (43%), polynodos goiter - 125(31%) and thyroid cancer - 105 (26%) (Table 1). Airway complications were diagnosed after trachea extubation based on indirect laryngoscope, presence of stridor, desaturation. The Pearson’s criteria was calculated.

**Results:**

The ratio of airway complications after thyroid surgery was 9.7% (39 pts). The main reasons of airway complications in thyroid surgery included:  laryngeal edema - 22 pts (5.5%); recurrent laryngeal nerve injury - 12 pts (3.0%) and postoperative bleeding 5 pts (1.2%).

**Conclusions:**

Thyroid gland cancer and polynodosal goiter associated with laryngeal edema and recurrent laryngeal nerve injury (Pearsen criteria were 0.271 -moderate and 0.203 consequentially). It's may require more attention from the anesthetists after extubation and readiness for an urgent airway.

**References:**

1. Ribakov S et al. Ternopil: TDMU, 2008. p 424.

2. Rosato N et al. World J Surg 28:271-6, 2004

3. Ignjatoviæ M et al. Acta Chir Iugosl 50:155-75, 2003

**Table 1 (abstract P150).** Association between type of surgery and airway complication

	Laryngeal edema	Pearson´s criteria	Recurrent nerve injury	Pearson´s criteria
Euthyroid goiter (170 pts)	4	0.116 weak	2	0.082 NaN
Polynodos goiter (125 pts)	7	0.194 weak	4	0.147 weak
Thyroid gland cancer (105 pts)	11	0.271 moderate	6	0.203 weak

## P151 Serum iron level and development of multiple organ dysfunction syndrome in patients in the perioperative period

### S Tachyla

#### Mogilev Regional Hospital, Department of Anesthesiology and Intensive Care, Mogilev, Belarus

**Introduction:**

Recently there has been attention of researchers to the problem of perioperative anemia. It was found that it increases the risk of death and postoperative complications. Threatening complication is multiple organ dysfunction syndrome (MODS). The objective was to determine the level of serum iron in the perioperative period in patients with endoprosthetics of large joints, and with the presence of MODS in abdominal surgery.

**Methods:**

A prospective cohort study was conducted in 77 patients, including 18 men and 59 women, age 61.9 ± 15.1 years. Two groups were identified: 1st (control) - patients after endoprosthetics of large joints (n = 40), 2nd (main) - patients in abdominal surgery with the presence of MODS (n = 37). The presence of MODS was established based on the criteria for the 2016 SCCM / ACCP Conference. Serum iron was monitored using an AU 680 analyzer (USA). The study identified several stages: 1st - before surgery, 2nd - 1st day after surgery, 3rd - 3rd day, 4th - 7th day, 5th - 10th day.

**Results:**

When studying the indicators of serum iron, its significant decrease (p <0.05) in the postoperative period was established. In the 1st group: 1st stage - 15.2 (10-19.4) mmol / L, 2nd stage - 5.2 (3.9-7.6) mmol / L, 3rd stage - 6.6 (5-8.7) μmol / L, stage 4 - 9.7 (8.6-12.1) μmol / L, stage 5 - 9.4 (7.8-11 9) μmol / L. In the 2nd group: 1st stage - 11.9 (10-15) mmol / L, 2nd stage - 3.7 (1.7-4.1) mmol / L, 3rd stage - 3, 6 (2.4-4.5) μmol / L, stage 4 - 6.5 (4.4-8.2) μmol / L, stage 5 - 7.6 (6.5-9 4) μmol / L. Moreover, in both groups, iron increased at the 4th stage against the 2nd stage (p <0.05). When comparing the level of iron between the groups, significant differences were found (p <0.05) at the 2nd, 3rd and 4th stages.

**Conclusions:**

In patients in the postoperative period, a decrease in serum iron is observed, the level of which rises by the 7th day, but does not reach the initial values. This decrease is more pronounced in patients with the presence of MODS after abdominal surgery.

## P152 Infection in simultaneous pancreas-kidney transplantation

### T Isidoro Duarte^1^, J Marques^2^, J Silva^3^, J Estevão^1^, N Germano^1^

#### ^1^Curry Cabral Hospital, Central Lisbon University Hospital Center, Intensive Care Medicine Department, Lisboa, Portugal; ^2^Curry Cabral Hospital, Central Lisbon University Hospital Center, Nephrology Department, Lisboa, Portugal; ^3^Covilhã Hospital, Cova da Beira Hospital Center, Internal Medicine Department, Covilhã, Portugal

**Introduction:**

Outcomes of simultaneous pancreas-kidney (SPK) transplantation have been improved over the decades. The increasing success is a result of improved surgical technique, better organ preservation, potent immunosuppression therapy and effective use of antibiotics. Nevertheless, morbidity and mortality following SPK transplantation remain high, mainly owing to infection [1].

**Methods:**

Retrospective analysis of patients admitted for SPK transplantation from 2013-2018 in an Intensive Care Unit (ICU). Immunosuppression was done with thymoglobulin, tacrolimus, mycophenolic acid and corticosteroids. Ceftazidime and fluconazole prophylaxis were given for 7 days as well as valganciclovir and trimethoprim/sulfamethoxazole. Infections were diagnosed by the presence of fever with clinical findings that could not be attributed to other cause. Microbiological samples were collected at admission and when suspicion of infection was present.

**Results:**

Total of 90 patients were admitted to ICU after SPK transplantation. Mean age 37.4±6.4 years. Donor’s mean age 34.6±11.6 years. Kidney and pancreatic graft thrombosis happened in 5.6% and 18.9%, respectively, and bleeding in 21.1%. Forty-one (45.6%) developed at least one infection during hospital stay. Infection during ICU was found in 13.3% and main pathogens were gram negative bacilli sensible to beta-lactam. After ICU, the incidence of multi-drug resistant pathogen was 13.5%, predominantly gram negative bacilli. Fungal infection was lower 4%. All-cause hospital mortality rate was 5.6%.

**Conclusions:**

Infectious complications are the main cause of morbidity and mortality following SPK transplantation. The administration of broad-spectrum prophylactic antibiotics are leading to the appearance of multi-drug resistant pathogens. Knowing local microbiological flora may be helpful, allowing more adequate antibiotic prophylaxis.

**References:**

1. Michalak G et al. Transplant Proceed 37,3560-3563, 2005

## P153 Thrombotic complications predictors in children underwent cardiac surgery with CPB

### V Lastovka^1^, O Gordeeva^2^, A Bidzhiev^2^

#### ^1^National Medical Research Center for Children´s Health, pediatric ICU, Moscow, Russia; ^2^National Medical Research Center for Children´s Health, Moscow, Russia

**Introduction:**

Cardiopulmonary bypass (CPB) is associated with thrombotic complications. Occurrence of thrombosis after CPB is 12% which takes the third place between CPB-associated complications. Our study determined preoperative predictors of thrombosis in children with congenital heart defects.

**Methods:**

138 patients with congenital heart diseases in age up to 11 months 29 days (median age - 4,7 months, youngest age – 2 days after birth, oldest – 11 months 29 days), underwent cardiac surgery with CPB, were enrolled in this study. All patients were divided into two groups: 1^st^-without thrombosis, 2^nd^-with thrombosis. Protein C, D-dimer, von Willebrand factor and Plasminogen plasma levels were assessed directly before surgery. Thrombotic cases were proven by performing doppler ultrasound or MRI.

**Results:**

Thrombotic complications were diagnosed in 30 children (21%). Between all thrombotic complications ischemic strokes were diagnosed in 73% (22 cases), arterial thrombosis in 17 % (5 cases), intracardiac thrombus in 7% (2 cases) and mechanical mitral prosthetic valve thrombosis 3%(1). Receiver operating characteristic (ROC) curves are created for the listed indicators. Area under the Curve (AUC) for Protein C 0,64 (Sensitivity(Sn)- 65%, Specificity(Sp) - 50%), D-dimer is 0,65 (Sn – 65%, Sp 50%), for Plasminogen activity - 0,62 (Sn 60%, Sp 40%) and for von Willebrand factor level - 0,64 (Sn 80%, Sp 55%). An ROC curve was created for all three indicators, the AUC was 0.7 (Sn – 80%, Sp – 40%). These parameters can be recommended as predictors of thrombosis in children after cardiac surgery.

**Conclusions:**

CPB is related with a large number of life-threatening complications. In our work, preoperative predictors of thrombosis were identified. Based on this data, it is possible to create thrombosis risk scale change the tactics of the anaesthetic approach, the prevention of thrombosis in the postoperative period. Further studies are needed to identify other possible predictors of thrombosis.

## P154 C-reactive protein level variation as a predictor of ischemic complications after vascular surgery

### SM Fernandes^1^, D Conduto^2^, C Candeias^1^, JM Ribeiro^3^

#### ^1^Centro Hospitalar Universitário Lisboa Norte, Serviço de Medicina Intensiva, Lisboa, Portugal; ^2^Faculdade de Medicina da Universidade de Lisboa, Lisboa, Portugal, ^3^Centro Hospitalar Universitário Lisboa Norte, Lisboa, Portugal

**Introduction:**

Abdominal ischemia occurs in 9% of patients submitted to aortic aneurysm repair. Its early diagnosis requires an elevated index of suspiction, particularly in more severe patients. We hypothesized that earlier increase and higher levels of C-reactive protein (CRP) may help to predict intra-abdominal ischemia.

**Methods:**

We performed a retrospective study of patients admitted to the intensive care department (ICD) after abdominal aorta aneurism surgery. We included all patients admitted during a two-year period, that survived for more than 48 hours. Primary outcome was splanchnic ischemia assessed by abdominal CT-scan. We also evaluated the presence of bacteremia, abdominal compartment syndrome and ICD mortality. Association between inflammatory parameters and ischemia was evaluated by multivariate logistic regression.

**Results:**

We included 88 patients (47 elective and 41 emergent) admitted to the ICD after major abdominal vascular surgery. Mean age was 71.0 ± 8.6 and 90% were male. Mean SAPS II value was 44.4 ± 20.6 and admission SOFA 6.7 ± 4.5. CRP levels increased after surgery with peak level at day 3 (26.01 ± 11.3 mg/dL). Suspicion of systemic infection occurred in 19 patients, but none of them had positive blood cultures. Mesenteric ischemia was confirmed in 20 patients (23%); at day 1, there was a higher CRP value in patients that developed ischemia (13.5±8.8mg/dL in ischemia group vs 8.1 ±5.1 mg/dL in non ischemia; p=0.004), as well as in day 3 (p=0.003). These differences remained after adjustment for patient severity, with an OR=1.1 (1.0-1.1; p=0.04). Of note, there were no association of CRP levels with ICD mortality.

**Conclusions:**

In this retrospective study, patients submitted to abdominal aortic surgery who developed mesenteric ischemia had an earlier and a higher increase in CRP levels. Defining an algorithm for systemic inflammatory biomarkers levels could be useful for early identification of ischemic complications, allowing for earlier diagnosis and treatment.

## P155 Postoperative C-reactive protein concentrations to predict infective complications following gastrectomy for cancer

### M Van Winsen^1^, ST McSorley^2^, R McLeod^1^, A MacDonald^3^, M Forshaw^3^, K Puxty^1^

#### ^1^Dept of Anaesthetics and Critical Care, Glasgow Royal Infirmary and University of Glasgow, Glasgow, United Kingdom; ^2^Academic Unit of Surgery, University of Glasgow, Glasgow, United Kingdom; ^3^Dept of Upper GI Surgery, Glasgow Royal Infirmary, Glasgow, United Kingdom

**Introduction:**

CRP (c-reactive protein) has been shown to be a useful biomarker in identifying complications after major abdominal surgery. Gastrectomy is a high-risk surgical procedure that requires post-operative critical care support to monitor for complications which are predominantly infective in nature. The aims of this study were to determine whether there is a relationship between post-operative CRP levels and patients who developed post-operative infective complications.

**Methods:**

A retrospective analysis was performed on patients undergoing elective gastrectomy for gastric cancer at a single centre between September 2011 and July 2016. Post-operative CRP levels for each day following resection were analysed for all patients. ROC curve analysis was used to determine which post-operative day (POD) gave the optimal cut-off.

**Results:**

Of 144 patients included, the majority were male (61.8%), mean age was 68.5 years and 53.5% had node-negative disease. A total of 84 patients (58.3%) had an infective complication, which includes those who experienced an anastomotic leak. CRP levels on post-operative day 3 gave the greatest AUC for the gastrectomy group (0.765). CRP cut-off of 220mg/L was significantly associated with infective complications (OR7.29, 95% CI 3.42-15.58, p= <0.001) and gave a sensitivity of 70% and specificity 76% (PPV 67%, NPV 78%). More patients with a CRP >220 on post-operative day 3 experienced an infective complication (67% vs 21%, p = <0.001) or a leak in particular (17% vs 6%, p = 0.059).

**Conclusions:**

A CRP level of less than 220 mg/L on POD3 may be useful to predict the development or exclude the likelihood of such infective complications in this group of patients prior to clinical signs (PPV 67%, NPV 78%). This may prompt and facilitate decision-making regarding early investigation and intervention or prevent inappropriate early discharge from critical care, whilst providing more assurance in identifying those who could be stepped down to ward level care.

## P156 Renin in cardiopulmonary bypass-associated vasoplegic syndrome

### MM Juarez^1^, SJ Pearson^1^, BM Morrissey^1^, NW Flemming^2^, P Devarajan^3^, LS Chawla^4^, TE Albertson^1^

#### ^1^University of California, Davis School of Medicine, Department of Internal Medicine, Pulmonary and Critical Care and Sleep Medicine, Sacramento, United States; ^2^University of California, Davis School of Medicine, Department of Anesthesiology and Pain Medicine, Sacramento, United States; ^3^Cincinnati Children´s Hospital Medical Center, University of Cincinnati College of Medicine, Division of Nephrology and Hypertension, Cincinnati, United States; ^4^Veterans Affairs Medical Center, San Diego, USA and La Jolla Pharmaceutical Company, San Diego, United States

**Introduction:**

Vasoplegia is commonly observed after cardiopulmonary bypass surgery (CPB) and associated with high mortality. Chronic use of renin-angiotensin aldosterone system inhibitors (RAASi) is associated with its incidence and ensuing need for vasopressor support after CPB. Renin serves as marker of tissue perfusion [1]. We examined the role of renin in the setting of RAASi exposure and vasopressor needs in the peri-CPB period.

**Methods:**

Prospective observational study of 31 adult patients undergoing CPB, aged 66.0±10.5 years (22 men, 9 women). Blood was collected 1) post induction, pre-CPB; 2) 30 min post cardioplegia, and 3) immediately post bypass. Vital signs and perioperative medications were recorded. As control, blood was collected from 5 men and 4 women aged 53.5 ±10.7, not diagnosed with lung disease and not prescribed any RAASi.

**Results:**

Baseline plasma renin in CPB patients tended to be higher than in control subjects (mean=38.5pg/ml±9.2 vs. 17.1 pg/ml ± 3.5, respectively, p=0.670). 30 minutes into CPB, mean renin was increased from baseline (77.6 pg/ml±17.5, p=0.211), and remained elevated immediately post CPB (74.6 pg/ml±19.1). Patients using RAASi prior to CPB tended to have a larger increase in renin post CPB (delta=55.3 pg/ml±30.9) vs. those not previously on RAASi (26.9 pg/ml±9.5, p=0.092). Renin was elevated in patients requiring vasopressor support in the 24 hours post CPB vs. those not requiring pressors (41.4 pg/ml±15.7 vs. 25.1 pg/ml±16.1 p=0.0246). In those prescribed RAASi and requiring pressors post CPB, there was a tendency toward greater renin increase than those not requiring pressors postoperatively (49.9 pg/ml±49.7 vs. 8.5 pg/ml±3.9, p=0.036).

**Conclusions:**

This study suggests a trend toward higher renin levels, particularly during CPB, in patients prescribed RAASi, and a positive association between renin and postoperative vasopressor needs. We speculate that increased renin levels may predict postoperative vasoplegia.

**References:**

1. Gleeson PJ et al. Crit Care Med 47;2:152-158, 2019

## P157 Blood loss and transfusion in minimal invasive coronary artery bypass surgery

### K Buyck^1^, J Vandenbrande^1^, JP Ory^1^, J Dubois^1^, A Yilmaz^2^, B Stessel^1^

#### ^1^Jessa Hospital, Critical Care, Hasselt, Belgium; ^2^Jessa Hospital, Cardiac Surgery, Hasselt, Belgium

**Introduction:**

Cardiac surgery is associated with perioperative blood loss and a high risk of allogenic blood transfusion. It has been recognized that high blood product transfusion requirement is associated with adverse clinical outcomes.^1^ Guidelines on patient blood management therefor aim at reducing blood loss and blood transfusion requirements in cardiac surgery.^2^ As there remains controversy about the advantage of minimal invasive techniques on blood loss an transfusion requirements,^2^ we wanted to investigate if the average blood loss and transfusion requirement in minimal invasive endoscopic coronary artery bypass graft surgery (endo-CABG) differ from conventional technique. We assessed the influence of pre-operative anticoagulant medication for blood loss. Estimated average blood loss after conventional CABG^3^ is 400ml (+/-200) and transfusion requirement 3,4 units packed red blood cells^4^.

**Methods:**

We performed a retrospective cohort study of our cardiac surgical database. From 01/01/2016 to 31/12/2017, we collected data from 423 patients undergoing endo-CABG. We analyzed blood loss, transfusion as well as pre-operative use of anti-coagulants as a risk factor for blood loss.

**Results:**

We found that mean total blood loss in endo-CABG does not differ from conventional CABG, nonetheless mean transfusion requirement was lower in our cohort. Use of direct oral anticoagulant is aossciated with increased blood loss and transfusion requirements (Table 1).

**Conclusions:**

Total blood loss is not influenced by minimal invasive technique for CABG (endo-CABG). An explanation for the lower transfusion requirements is the use of a minimal extracorporeal circulation, which is known to reduce the risk of transfusion. Another important factor is the implementation of a standardized transfusion-protocol based on available evidence. Reducing transfusion requirements is an important component in improving patient outcome after cardiac surgery and is related to multiple factors in perioperative care of our patients.

**Table 1 (abstract P157).** Use of pre-operative anti-coagulant medication

	Total transfusion (blood) (units)	Total blood loss (ml)	
Total group (n=423)	0.7 ± 3.07 0 (0, 0)	428.2 ± 343.3 314.5 (247.8, 499.3)	
Use of pre-operative medication	Number of patients (%)	Blood loss (ml)	Total transfusion blood (units)
No pre-operative treatment	46 (10.8%)	480.8 ± 449.9 354 (257.5, 540)	1.62 ± 7.8 0 (0, 0)
NOAC	24 (5.6%)	542 ±435.6 343.5 (250, 671.5)	2.62 ± 5.64 0 (0, 3)
ASA	346 (81.7%)	424.8 ±336.8 306.5 (247, 492.8)	0.60 ± 1.85 0 (0, 0)
API	107 (25.9%)	411.6 ± 309.6 295 (244, 523)	0.89 ± 1.58 0 (0, 1.25)
ASA + API	89 (21 %)	426.2 ± 334.1 300 (244.5, 530.5)	0.97 ± 1.56 0 (0, 2)

## P158 Retinal microvascular damage associated with mean arterial pressure during cardiopulmonary bypass surgery

### V Shipulin^1^, M Dyakova^1^, Y Podoksenov^2^, E Shishneva^2^, Y Svirko^3^, M Denisko^4^, O Krivosheina^4^, I Mandel^5^

#### ^1^Cardiology Research Institute,, Cardiosurgery, Tomsk, Russia; ^2^Cardiology Research Institute,, ICU, Tomsk, Russia; ^3^Cardiology Research Institute,, Laboratory, Tomsk, Russia; ^4^Siberian State Medical University, Ophthalmology, Tomsk, Russia; ^5^Sechenov University, Anesthesiology and Intensive Care, Moscow, Russia

**Introduction:**

Retinal perfusion corresponds to cerebral perfusion and it is very sensitive to hemodynamic disturbances [1, 2]. We investigated the association between retinal microvascular damage and hemodynamic characteristics in patients undergoing coronary artery bypass grafting surgery (CABG) with cardiopulmonary bypass (CPB).

**Methods:**

10 patients with coronary artery disease and systemic hypertension were examined. Ophthalmoscopy and optical coherence tomography were performed before and 10-14 days after CABG. The hemodynamic parameters during CPB were analyzed.

**Results:**

4 (40%) patients had changes in the retinal vessels and in the ganglionic fiber structure on 10-14 day after surgery: in 30% of patients the foci of ischemic retinal oedema appeared, in 10% the decrease of the thickness of ganglionic fiber were observed. These changes may be associated with intraoperative ischemia of the central retinal artery. In 6 (60%) patients the mean arterial pressure (MAP) during CPB was increased up to 90 mmHg. In 4 (67%) of them the association between MAP and foci of ischemic retinal oedema were revealed. The ischemic retinal changes were observed significantly more often if the delta of MAP during CPB was over then 20 mm Hg compared with the patients where the delta of MAP was less than 20 mm Hg (p=0.035). This is probably due to an intraoperative disorders of the myogenic mechanism of blood flow autoregulation in the retinal microvasculature in patients with coronary artery disease [3].

**Conclusions:**

The level of MAP up to 90 mm Hg during CPB is associated with retinal blood flow impairment and the foci of ischemic retinal oedema. Delta of MAP more than 20 mmHg was associated with the foci of ischemic retinal oedema and decreased ganglionic fiber thickness in 67% of cases.

**References:**

1. Ascione R et al. Circulation 112:3833-3838, 2005.

2. Murphy GS et al. Anesth Analg 108 :1394-1417, 2009

3. Joshi B. Anesth Analg 114:503-10, 2012

## P159 Atrial fibrillation after cardiac surgery: implementation of a prevention care bundle on intensive care unit improves adherence to current perioperative guidelines and reduces incidence

### M Buerge^1^, R Magboo^2^, D Wills^3^, I Karpouzis^4^, P Cooper^5^, D Balmforth^6^, N Roberts^6^, B O´Brien^3^

#### ^1^Royal Papworth Hospital NHS Trust, Anaesthesia and Intensive Care, Cambridge, United Kingdom; ^2^St Bartholomew´s Hospital, Barts Heart Centre, Adult Critical Care Unit, London, United Kingdom; ^3^St Bartholomew´s Hospital, Barts Heart Centre, Perioperative Medicine, London, United Kingdom; ^4^Gennimatas General Hospital, Cardiology, Athens, Greece; ^5^St Bartholomew´s Hospital, Barts Heart Centre, Pharmacy, London, United Kingdom; ^6^St Bartholomew´s Hospital, Barts Heart Centre, Cardiothoracic Surgery, London, United Kingdom

**Introduction:**

Atrial fibrillation after cardiac surgery (AFACS) is a very frequent complication affecting 30-50% of all patients. It is associated with an increase in morbidity, mortality and hospital and intensive care unit (ICU) length of stay. We aimed to implement an AFACS prevention care bundle based on a recently published practice advisory [1], focusing on early postoperative (re)introduction of β-blockers.

**Methods:**

Baseline AFACS incidence and β-blocker administration practices in our centre were audited for all patients undergoing valve surgery or coronary artery bypass graft (CABG) during a 6 weeks period. The AFACS prevention care bundle – an easy to follow graphical tool – was subsequently introduced to the cardiac ICU by a multidisciplinary team and audited following a model of improvement approach. After exclusion of patients with preoperative AF, differences between pre- and post-implementation groups were compared with Chi-square and Fisher’s exact tests for categorical, and One-way ANOVA for continuous variables, using SPSS.

**Results:**

A total of 384 patients were analysed. Patient and surgery characteristics did not differ between groups. Significantly more patients received postoperative β-blockers after bundle implementation (82.7% pre- vs 91.3% post-bundle, p=0.019) with a higher proportion on day 1 (36.7% pre- vs 67% post-bundle, p<0.001, Figure 1). The incidence of AFACS was significantly reduced from 35.4% to 23.3% (p=0.009), with a particularly marked reduction in the age group 65-75 years and for isolated aortic valve and CABG surgery. There was no significant reduction in hospital length of stay for this cohort.

**Conclusions:**

Introduction of an AFACS prevention care bundle using a graphical tool improved adherence to current guidelines with regards to early β-blocker administration and significantly reduced AFACS incidence. Future care bundles should include preoperative interventions and might reduce hospital length of stay.

**References:**

1. O'Brien B et al. J Cardiothorac Vasc Anesth 33:12-26, 2019

**Fig. 1 (abstract P159).**
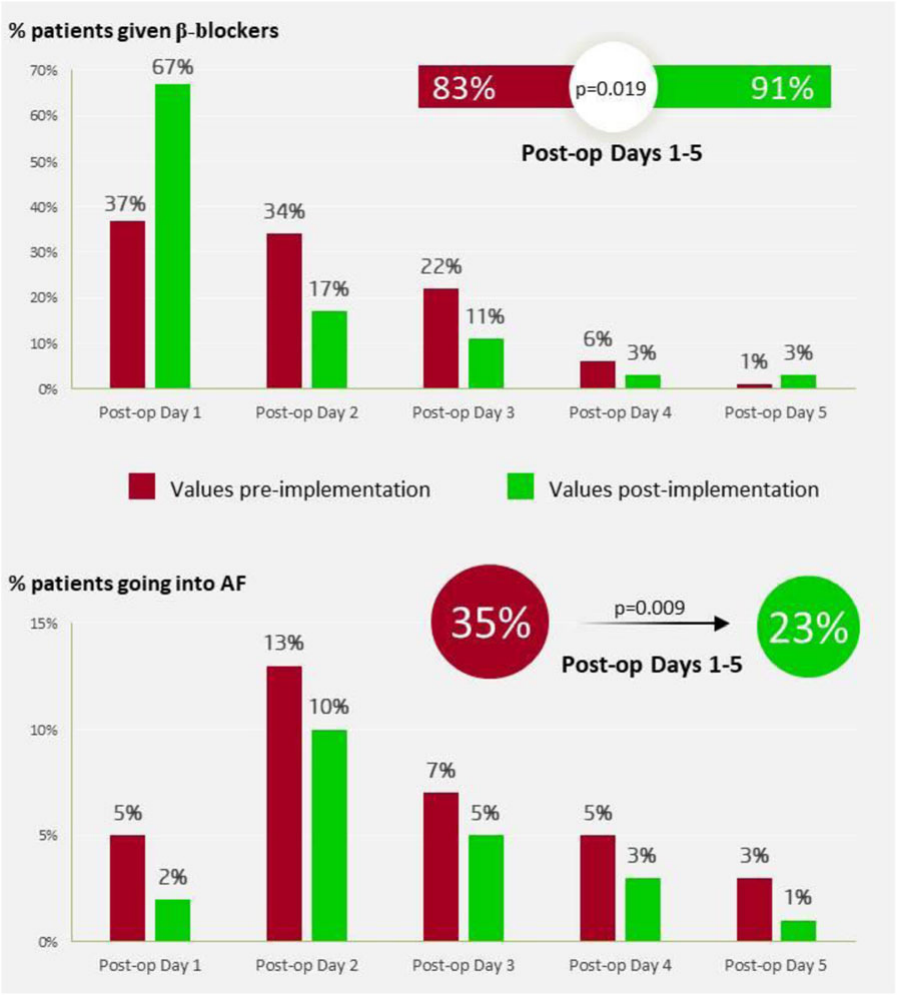
Timing of beta-blocker (re)initiation versus incidence of AFACS before and after prevention care bundle implementation, per post-operative day and for postoperative days 1-5 (insets)

## P160 Use of hypoxic gas mixture in neonates with single ventricle physiology prior to the Norwood procedure

### V Sheward, E Al Mahmoud, K Ramakrishnan

#### Sheikh Khalifa Medical City Hospital, PCICU, Abu Dhabi, United Arab Emirates

**Introduction:**

In neonates with univentricular physiology, there is a delicate balance between pulmonary and systemic circulations, with a tendency towards generous pulmonary blood flow, and a risk of systemic underperfusion. Preoperatively, the use of hypoxic gas mixture (HM) has been advocated as a therapy to increase PVR, with the aim of improving systemic oxygen delivery. It is a therapy which has been routinely initiated in our institution in the setting of signs of pulmonary overcirculation.

**Methods:**

We performed a retrospective analysis of all patients in our institution who underwent a Norwood procedure and who received HM preoperatively. We compared peripheral saturations, arterial blood gas analysis, serum lactate, regional cerebral and renal saturations and invasive blood pressure, prior to, and then 4,8 and 24 hours after HM was commenced.

**Results:**

Between 2014 and 2018 (inclusive), 49 patients underwent the Norwood procedure. 18 patients received preoperative HM. Average FiO2 was 17% during administration of HM. Average peripheral saturations were 96.1% prior to HM, and dropped to 87.4% at 4 hours, and 88% at 8 and 24 hours after initiation (p < 0.05). There was no change in any of the measured markers of systemic oxygen delivery, including regional cerebral and renal saturations, lactate, urine output or blood pressure. There was an association between an extended period of HM (> 48 hours) and the need for pulmonary vasodilator therapy post Norwood procedure.

**Conclusions:**

Hypoxic gas mixture in patients with parallel systemic and pulmonary cicrculations causes desaturation and hypoxia. It does not lead to an increase in systemic perfusion and thus an improvement in systemic oxygen delivery. Its ongoing use in this fragile population should be considered.

## P161 Multimodal analgesia in neurocritical care patients: complementing therapies to reduce the use of endovenous opioids

### M Canitrot^1^, J Ramirez^2^, J Serra^3^, Y Santos^3^, V Munoz^3^, F Paravic^4^, S Ugarte^3^

#### ^1^INDISA CLINIC, Critical Care, Santiago, Chile; ^2^Andres Bello University, NeuroICU, Santiago, Chile; ^3^INDISA CLINIC, NeuroICU, Santiago, Chile; ^4^INDISA CLINIC, Neurorehabilitation, Santiago, Chile

**Introduction:**

Analgesia in the critical patient, and especially in the neurocritical patient, is a basic goal in all therapeutic practices. Patients in the ICU are frequently administered prolonged and/or high doses of opioids. Multiple serious complications due to the use of infusion of opioids at large doses has been described. To reduce high doses of intravenous opioids, multimodal forms of analgesia can be used.

**Methods:**

Prospective observational study of the use of tapentadol enteral and buprenorphine in transdermal patches, at low doses, for the control of pain and its effect on reducing the use of fentanyl infusion in high doses on 84 patients admitted to Neuro ICU of INDISA Clinic during 2 consecutive years (2018-19). Enteral tapentadol (through NG tube) 50 mg/6 hours, was considered in patients who required intravenous fentanyl in continuous administration. Buprenorphine was also added at low doses (5 ug/hr) in a weekly transdermal patch, in cases of neurosurgical spine patients, fractures and long-term neuropathic pain. Pain was controlled on Behavioral Pain Scale (BPS) and Visual Analogical Scale (VAS) scores, according to the conditions of each patient. Their hemodynamic, gastrointestinal complications and the appearance of delirium episodes according to CAM-ICU scale were recorded.

**Results:**

84 patients received tapentadol. 32 of them also received transdermal buprenorphine. All managed to maintain adequate level of analgesia, not requiring fentanyl at doses greater than 0.5 ug / kg / hr. Distribution by diagnoses: neurotrauma 21 patients, Guillain Barre 12, spine surgery 15, HSA 18, HICE 10, malignant ischemic ACV 8. Complications: gastric retention 12 patients (7%), hypotension 1 (1%), acute hypoactive delirium 3 (3.5%), acute hyperactive delirium 8 (9%). No drug interactions were found.

**Conclusions:**

The introduction of enteral tapentadol and buprenorphine patches in neurocritical patients was safe and resulted in a decrease in the use of endovenous opioids and its adverse effects.

## P162 Assessment-driven, protocol-based pain management post cardiac surgery significantly improves measured pain scores

### I Charles, A Johnson, M Govender, M Stevens, M Gohobur, CY Kim, L Campbell, EE Quaynor, H Hideg, G Zilahi

#### St George´s University Hospital NHS Foundation Trust, CTICU, London, United Kingdom

**Introduction:**

We hypothesized that changing the pain management for our post cardiac surgical patients to an assessment-driven, protocol-based approach using fast acting and easily titratable agents will significantly improve patient satisfaction by reducing pain intensity in the first 24h after surgery as suggested by Society of Critical Care [1] guideline.

**Methods:**

We prospectively assessed 101 and 99 (05.2018 vs 06.2019) consecutive patients before and after introducing our pain management protocol. The nursing and medical team received rigorous training on the guideline as well as the correct assessment using appropriate pain scores measured at least hourly (Numeric Pain Score, ≥ 2 is moderate to severe or Critical Care Observation Tool, > 2 is moderate to severe). We introduced a multimodal approach with a combination of fast acting iv, long acting oral opiates, regular paracetamol and rescue iv boluses for difficult to control situations and we created a prescription bundle on our electronic prescribing record. Among other variables we assessed hours spent in moderate to severe pain in the first 24h after surgery and compared to the data collected before the guideline was introduced.

**Results:**

We analysed 101 patients from 2018 and 99 from 2019. Baseline characteristics were similar between the two groups. In 2018 only 41.6% of the patients spent less than 5 hours and 29.4% spend more than 10 hours in moderate to severe pain. The 2019 data showed significant improvement in that 79.5% of patients spent less than 5 hours and only 5% patients who spent more than 10 hours in moderate or severe pain. (p <0.0001, Chi Square) (Figure 1). Only 9% of the patient needed rescue medications. 3% of time was the protocol inadequate necessitating other approach.

**Conclusions:**

Introducing an assessment driven, stepwise, protocolized pain management significantly improved patient satisfaction by reducing pain intensity in the first 24h on our Cardiothoracic Intensive Care Unit.

**References:**

1. Devlin JW et al. Crit Care Med 46:e825–e873, 2018

**Fig. 1 (abstract P162).**
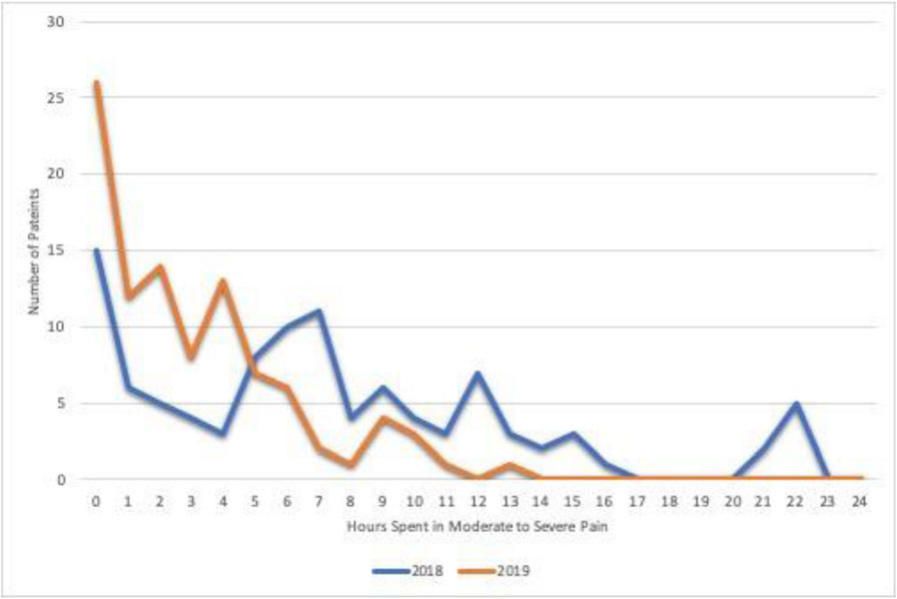
Hours spent in moderate to severe pain post cardiac surgery in the first 24h

## P163 Compartment psoas block efficacy for perioperative analgesia in the elderly with proximal femur fractures

### K Bielka, I Kuchyn, I Tokar

#### Bogomolets National medical university, Postgraduate surgery, anesthesiology and intensive care, Kyiv, Ukraine

**Introduction:**

Proximal femur fractures are most common fractures in the elderly and associated with significant mortality and morbidity, with high economic and social impact. Perioperative pain management influence outcomes and mortality after surgery with early mobilization being possible [1, 2]. The goal of the study was to compare the efficacy and safety of the compartment psoas block for perioperative analgesia in elderly patients with proximal femur fractures.

**Methods:**

The randomized controlled study was held in medical center "Into-Sana" (Odesa, Ukraine) from January 2018 till July 2019. Patients with proximal femur fractures and older than 60 years were included in the study. They were randomly allocated to 2 groups – compartment psoas block group (bupivacaine analgesia was started as soon as possible before surgery and prolonged during and after surgery with additional ischiadicus block before surgery) and general (inhalational) anesthesia with systemic analgesia perioperatively.

**Results:**

60 patients were included in this study. Perioperative compartment psoas block was associated better pain control, decreased opioid consumption, better sleep quality, earlier mobilization after surgery, decreased incidence of opioid-associated vomiting/nausea and myocardial injury. There were no difference in the incidence of hospital acquired pneumonia and delirium.

**Conclusions:**

Perioperative compartment psoas block is effective and safe for perioperative analgesia in elderly patients with proximal femur fractures, and is associated with better pain control and decreased complications incidence.

**References:**

1. Dixon J et al. Geriatr Orthop Surg Rehabil 9:2151459318806443, 2018

2. Zhang X et al. Ther Clin Risk Manag 9:299–302, 2013

## P164 Evaluation of safety and efficacy of concomitant parenteral olanzapine and benzodiazepine for agitation

### HH Huynh, TS Lam, JT Jancik, G Betterman

#### Hennepin County Medical Center, Clinical Pharmacy, Minneapolis, United States

**Introduction:**

Parenteral olanzapine is frequently used in combination with parenteral benzodiazepines for hospitalized patients with severe agitation. The FDA issued a warning for increased risk of excessive sedation and cardiorespiratory depression with this combination based on post-marketing case reports with overall limited quality of evidence [1]. The purpose of this study is to evaluate the safety and efficacy of concomitant parenteral olanzapine and benzodiazepine for agitation.

**Methods:**

This retrospective chart review evaluated agitated patients who received concomitant parenteral olanzapine and benzodiazepine within 60 minutes from 1/31/2016 to 9/1/2019 . The primary end points were rate of respiratory depression requiring mechanical ventilation and hypotension requiring vasopressors. The secondary end points were percentage of patients requiring additional sedatives for agitation during the same time frame, cumulative dose of olanzapine and benzodiazepine (midazolam equivalent) received, and rate of cardiac arrest and death.

**Results:**

A total of 208 patients were included with notable baseline characteristics: median age of 35 years old, 59% with a history of substance abuse, and 40% with a history of psychiatric illness. For the primary outcomes, 2.9% of patients required mechanical ventilation and 0% required vasopressors. Additionally, 27.9% patients received additional sedating agents to control agitation. Refer to Table 1 for more details. No cardiac arrests or deaths were observed.

**Conclusions:**

Concomitant use of parenteral olanzapine and benzodiazepine within 60 minutes for the treatment of agitation appears to have a small risk of respiratory depression without significant hypotension.

**References:**

1. Marder SR et al. J Clin Psychiatry 71:433-41, 2015

**Table 1 (abstract P164).** Results

Primary Outcomes	Initial	Post	Difference
SBP, median (IQR), mm Hg	130 (119-145)	125 (109-136)	-5
Vasopressor support	--	0/208 (0%)	--
Mechanical ventilation for respiratory depression	--	6/208 (2.9%)	--
Secondary Outcomes			
Olanzapine, mean, mg	--	11.8	--
Midazolam equivalent, median (IQR), mg	--	5 (4-5)	--

## P165 Could preoperative US guided fascia iliaca compartment block (FICB) decrease opioid consumption and perioperative morbidity of elderly patients with hip surgery for femoral fracture?

### D Pavelescu^1^, I Grintescu^2^

#### ^1^Emergency Hospital Floreasca, Anesthesiology and Intensive Care, Bucharest, Romania; ^2^Emergency Hospital Floreasca, Intensive Care and Anesthesiology, Bucharest, Romania

**Introduction:**

Hip fracture is very common in the elderly,it causes moderate to severe pain often undertreated. FICB is a simple safe method, easy to learn and use. The aim of our study is to assess the efficacy and safety of preoperative FICB compared with intravenous analgesia for elderly patients with femoral fracture and hip surgery in terms of opioid consumption and perioperative morbidity

**Methods:**

After informed consent obtained,54 patients 50-98 yo ASA I-III with hip fracture were randomized to receive either an US guided FICB(40 ml of ropivacaine 0,35%) or a sham injection with normal saline 30' before surgery. Both groups were operated under general anesthesia. Postoperative analgesia was done according to VAS: VAS 0-30 mm, paracetamol 1g iv at 8 h, VAS 30-60 mm, ketoprofen 100 mg iv at 8 h, VAS>60, morphine 0,1mg/kgBW iv. The primary outcome was the comparison of VAS score at rest over the first 30'following the procedure, at the end of the surgery and at 6h intervals for 24h. The secondary outcome were the incidence of the cardiovascular events, of the PONV and of the confusion episodes, the amount of morphine consumption for 24h

**Results:**

At baseline, FICB group (A) had a lower mean pain score than the sham injection group (B). The same difference was observed over 24 h of follow-up (p<0.05). There was a significant difference between the two groups in total cumulative iv morphine consumption at 24 h and in the incidence of PONV and confusion episodes (Figure 1).

**Conclusions:**

FICB provides effective analgesia for elderly patients suffering from hip fractures, with lower morbidity and lower opioid consumption compared with intravenous analgesia.

**Fig. 1 (abstract P165).**
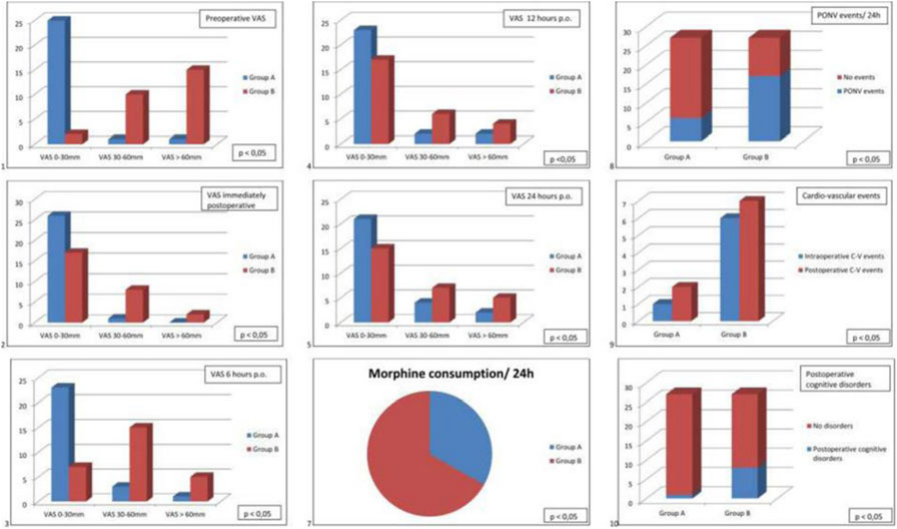
Results

## P166 Pain assessment in chronic disorders of consciousness patients with ANI monitoring

### E Kondratyeva, M Aybazova, N Dryagina

#### Almazov National Medical Reseach Centre, Minimally conscious research group, St Petersburg, Russia

**Introduction:**

Pain and suffering controversies in DOC to be debated by the scientific, legal and medical ethics communities.

**Methods:**

ANI (anti nociception index) monitor was used to assess pain in patients with chronic disordersof consciousness (DOC) age range 22 to 56 years - 9 in vegetative state/ unresponsive wakefulness syndrome (VS/UWS) and 20  minimal consciousness state (MCS). Average age: in MCS group 31,8±11,29 and 42,1±8,46 in VS/UWS group. Neurological status was assessed using  CRS-R scale. The average score on the CRS-R scale was 5±1.4 in VS/UWS and 10.45±4.5 in MCS. Pressure on the nail phalanx was used as a pain impulse. ANI and Nociception coma scale was evaluated before the application of pain stimulus, immediately after and  past 30 minutes. Prolactin level was measured before the pain stimulus application and 10 minutes after. ANI less than 50 indicates pain, 70-100 hypoalgesia, 30 severe pain.

**Results:**

The mean value of the ANI in MCS patients: before the pain stimulus 66.25±14.11, after the pain stimulus application 45±16.12 and 30 minutes later 66.55±18.13. Prolactin level in MCS patients before pain 13.01±9.06 ng/ml; after pain 13.75±8.73 ng/ml (p>0.05). Prolactin in VS/UWS patients before pain 10.79±7.2 ng /ml, after pain 14.5 ±8.88 ng / ml (p> 0.05).

**Conclusions:**

ANI monitor revealed that VS/UWS and MCS patients react equally to the pain impulse. Prolactin dynamics showed poor statistical mean and can not be consider as a marker of nociception in this group of patients. It is possible that the level of pain impulse was insufficient neuroendocrine response  activation or the increase of prolactin level occurs in the long term (more than 10 minutes). In all patients the basal level of the ANI index was above 66.25, which indicates absence of pronounced pain sensations at rest, but medical manipulations, physical therapy, can deliver pain.

Acknowledgement: The reported study was funded by RFBR project number 19-29-01066/2019

## P167 Hemodynamic effects of dexmedetomidine in patients with septic shock: a cohort study of the sedation practice in intensive care evaluation [SPICE III] trial

### L Cioccari^1^, N Luethi^2^, M Bailey^2^, A Messmer^1^, L Peck^3^, B Howe^2^, Y Shehabi^4^, J Takala^1^, S Jakob^1^, R Bellomo^3^

#### ^1^University Hospital and University of Bern, Department of Intensive Care Medicine, Bern, Switzerland; ^2^School of Public Health and Preventive Medicine, Monash University, Australian and New Zealand Intensive Care Research Centre, Melbourne, Australia; ^3^Austin Hospital, Department of Intensive Care Medicine, Heidelberg, Australia, ^4^School of Clinical Sciences, Monash University, Monash Health, Critical Care and Perioperative Services, Melbourne, Australia

**Introduction:**

Septic shock can result in catecholamine hyposensitivity, leading to hemodynamic instability and death. Experimental data suggest that central alpha2-agonists like dexmedetomidine (DEX) increase vasopressor responsiveness and reduce catecholamine requirements in septic shock [1]. However, because it can cause hypotension and bradycardia, clinicians may be reluctant to use DEX in such patients.

**Methods:**

In this cohort study of the Sedation Practices in Intensive Care Evaluation (SPICE III) trial, an international randomized trial comparing sedation with DEX to usual care, we studied critically ill patients with septic shock admitted to the Austin Hospital, Melbourne, Australia, and the University Hospital of Bern, Switzerland. The primary outcome was mean noradrenaline requirements in the first 48 hours.

**Results:**

Between November 2013 and February 2018, 417 patients were recruited into the SPICE III trial at both sites. Eighty-three patients with septic shock were included in this sub-study, of whom 44 (53%) received DEX and 39 (47%) usual care. Mean noradrenaline dose during the first 48 hours was 0.03 [0.01, 0.07] μg/kg/min in the DEX group and 0.05 [0.01, 0.15] μg/kg/min in the usual care group (p=0.08). Over the first 48 hours, patients in the DEX group had higher mean arterial pressures (MAP) (Figure 1) and lower vasopressor requirements to maintain the target MAP (expressed by the noradrenaline equivalents to MAP ratio: NEq/MAP) compared to the usual care group (ratio of adjusted difference in geometric means 1.4 [1.1, 1.9], p=0.02).

**Conclusions:**

In critically ill patients with septic shock, sedation with DEX does not increase noradrenaline requirements in the first 48 hours as compared to usual care. In contrast, DEX appears to be associated with lower vasopressor requirements to maintain the target MAP.

**References:**

1. Geloen A et al. Crit Care Med 41: e431-438, 2013

**Fig. 1 (abstract P167).**
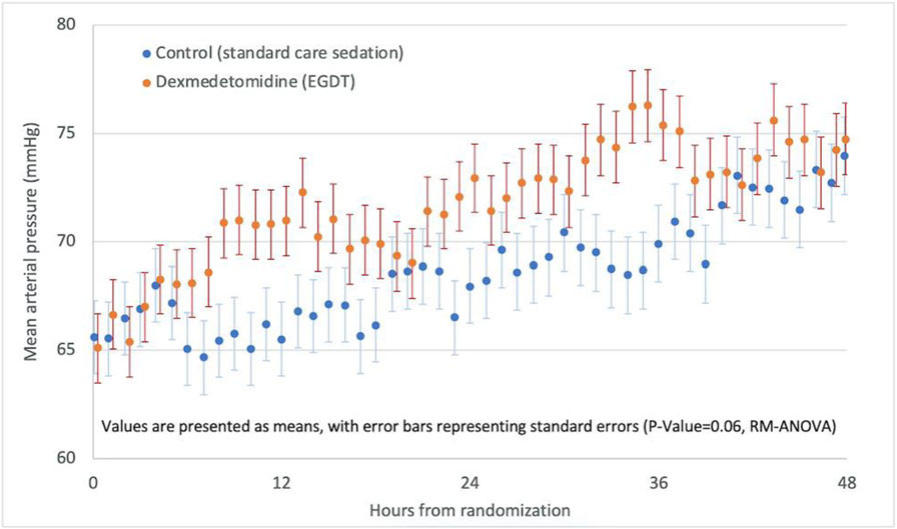
Mean arterial pressure in the first 48 hours after randomization

## P168 Multimodal analgesia with low dose of ketamine and dexamethasone, an interesting option for patients with total hip arthroplasty?

### D Pavelescu^1^, I Grintescu^2^

#### ^1^Emergency Hospital Floreasca, Anesthesiology and Intensive Care, Bucharest, Romania; ^2^Emergency Hospital Floreasca, Intensive Care and Anesthesiology, Bucharest, Romania

**Introduction:**

Total hip arthroplasty THA is one of the most common major surgical procedures associated with significant postoperative pain that can adversely affect patient recovery and could increase morbidity. Effective perioperative pain management allows an accelerated rehabilitation and improve the functional status of these patients. Multimodal analgesia MMA combines analgesics with different mechanism of action which by synergistic and additive effects enhance postoperative pain management and reduce complications. The aim of our study is to assess if perioperative association of very low dose of ketamine, a potent NMDA antagonist and dexamethasone, by antiemetic and antiinflammatory properties could decrease opioid consumption and postoperative morbidity of patients with THA.

**Methods:**

After informed consent, 58 patients scheduled for primary hip joint replacement surgery aged 55-91 yo ASA I-III were prospective randomized in two groups. Both groups were operated under general anesthesia fentanyl/sevoflurane. Supplementary, patients in group A received 12 mg iv dexamethasone and 8mg at 12 h and ketamine 10 mg iv bolus at induction and 10mg/h iv during surgery. Postoperative analgesia was done according to VAS, 0-30 mm paracetamol 1 g iv at 8 h, 30-60 mm ketoprofen 100 mg iv at 12h, VAS>60 mm morhine 0,1 mg/kgBW iv. We recorded perioperative opioid consumption, the number of intraoperative cardiac events, VAS score at the end of surgery and at 24 h, the incidence of PONV and persistance of chronic pain at 3 months.

**Results:**

We obtain a significant less pain score at the end of surgery p<0.05 in group A, no significant difference at 24 h, a significant less chronic pain at 3 months, a fewer NPVO and cardiovascular events in group A, p<0.05 (Figure 1).

**Conclusions:**

A multimodal approach with very low doses of ketamine and dexamethasone could be efficent in the treatment of pain for elderly patients with hip arthroplasty, decreasing postoperative side-effects and reducing chronic pain persistance.

**Fig. 1 (abstract P168).**
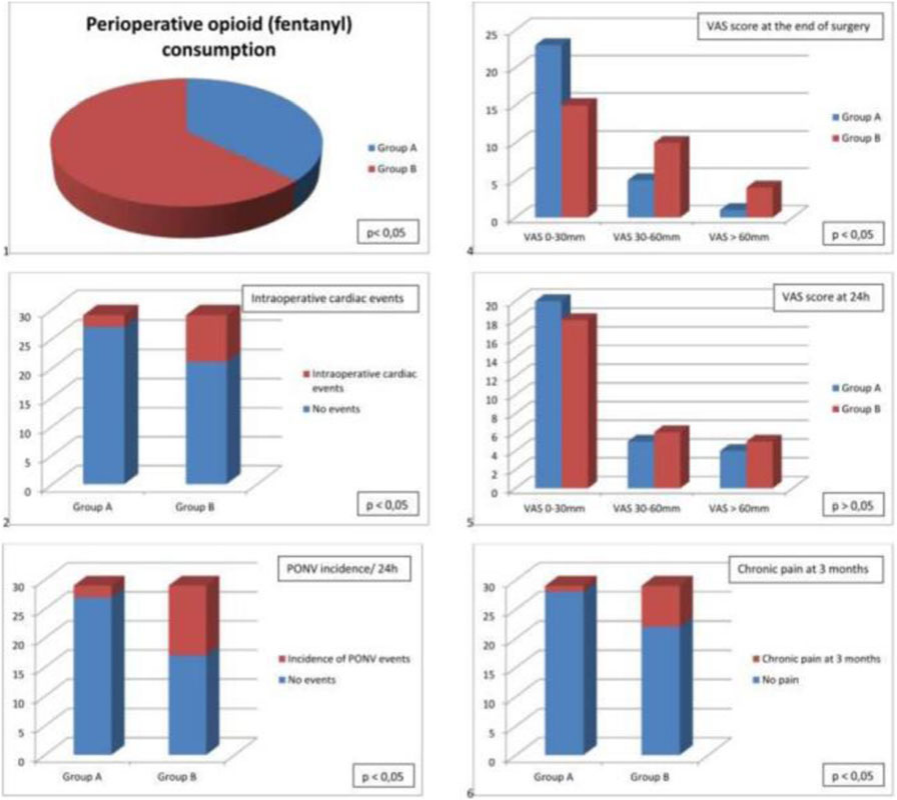
Results

## P169 Analgesia usage in ICU survivors

### P Mactavish^1^, C Pudrie^2^, M Shaw^3^, P Henderson^2^, H Devine^4^, T Quasim^5^, J Iwashyna^6^, J McPeake^4^

#### ^1^Glasgow Royal Infirmary, Pharmacy, Glasgow, United Kingdom; ^2^University of Glasgow, School of Medicine, Dentistry and Nursing, Glasgow, United Kingdom; ^3^NHS Greater Glasgow and Clyde, Clinicial Physics, Glasgow, United Kingdom; ^4^Glasgow Royal Infirmary, Intensive Care Unit, Glasgow, United Kingdom; ^5^University of Glasgow, School of Dentistry and Nursing, University of Glasgow, Glasgow, United Kingdom; ^6^VA Ann Arbor Health System, Centre for Clinical Management Research, Ann Arbor Michigan, United States

**Introduction:**

Treatment in an Intensive Care Unit (ICU) often necessitates uncomfortable and painful procedures for patients. Chronic pain is becoming increasingly recognized as a long term problem for patients following an ICU admission [1]. Throughout their admission patients are often exposed to high levels of opioids, however there is limited information available regarding analgesic prescribing in the post-ICU period. This study sought to examine the analgesic usage of ICU survivors pre and post ICU admission.

**Methods:**

183 patients enrolled in a post-intensive care programme between September 2016 and June 2018. Intensive Care Syndrome: Promoting Independence and Return to Employment (InS:PIRE), is a 5-week multicentre, multidisciplinary rehabilitation programme for ICU survivors and their caregivers. Patients’ level of analgesia was recorded pre-admission and upon attending InS:PIRE, their level of prescribed analgesia was categorized using the Word Health Organisation (WHO) analgesic ladder [2].

**Results:**

33.3% of patients (n=61) were prescribed regular analgesia pre-admission; this increased to 60.7% (n=111) post-admission, representing a significant absolute increase of 27.4% (95% CI: 20.2% - 34.4%, *p*<0.001) in the proportion of patients who were prescribed regular analgesia pre and post ICU. In addition, pre-admission, 22.4% (n=41) of patients were prescribed a regular opioid (step 2 and 3 of the WHO ladder) compared to 38.7% (n=71) post-admission, representing an absolute increase of 16.3% (95% CI: 9.8% -22.8%, *p*<0.001).

**Conclusions:**

This study found a significant increase in analgesic usage including opioids in ICU survivors. Follow-up of this patient group is essential to review analgesic prescribing and to ensure a long term plan for pain management is in place.

**References:**

1. Battle CE et al. Crit Care 17 :R101, 2013

2. World Health Organization. Cancer pain relief, with a guide to opioid availability. 2nd edition. Geneva: WHO, 1996

## P170 Impact of a multifaceted and multidisciplinary intervention on pain, agitation, and delirium management in a Canadian community ICU: a quality improvement study

### Z Ma^1^, M Carmargo Penuela^2^, M Law^2^, HO Chung^3^, J Tsang^3^

#### ^1^McMaster University, NIagara Regional Campus, Michael G. DeGroote School of Medicine, St. Catharines, Canada; ^2^Brock University, Department of Health Science, St. Catharines, Canada; ^3^McMaster University, Division of Critical Care, Hamilton, Canada

**Introduction:**

Pain, agitation, and delirium (PAD) are commonly encountered b patients in the Intensive Care Unit (ICU). Delirium is associated with adverse outcomes, including increased mortality and morbidity. Clinical guidelines suggest that routine assessment, treatment and prevention of PAD is essential to improving patient outcomes. Despite the well-established improvements on patient outcomes, adherence to clinical guidelines is poor in community hospitals. The aim of this quality improvement project is to evaluate the impact of a multifaceted and multidisciplinary intervention on PAD management in a Canadian community ICU.

**Methods:**

A PAD advisory committee was formed and involved in the development and implementation of the intervention. The 4-week intervention targeted nurses (educational modules, visual reminders), family members (interviews, educational pamphlet, educational video), physicians (multidisciplinary round script), and the multidisciplinary team (poster). An uncontrolled, before-and-after study methodology was used. Adherence to PAD guidelines in the assessment of PAD by nurses was measured 6 weeks pre-intervention and 6 weeks post-intervention.

**Results:**

Data on 430 patient-days (PD) and 406 PD were available for analysis during the pre- and post-intervention, respectively. The intervention significantly improved the proportion of PD with assessment of pain and agitation at least 4 times per 12-hour shift from 68.0% to 87.5% and from 69.7% to 82.2%, respectively (Figure 1). Proportion of PD with delirium assessment at least once per 12-hour shift did not significantly improve.

**Conclusions:**

A multifaceted and multidisciplinary PAD intervention is feasible and can improve adherence to PAD assessment guidelines in community ICUs. Quality improvement methods that involve front-line staff can be an effective way to engage staff with PAD.

**Fig. 1 (abstract P170).**
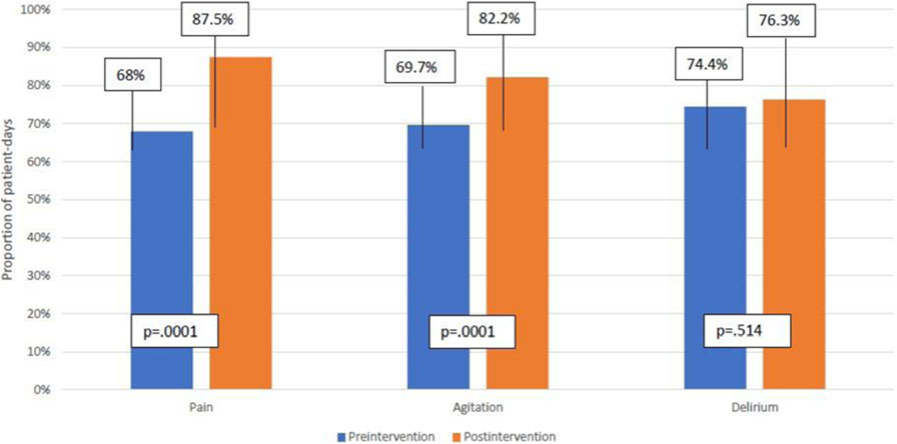
Pre-intervention and post-intervention PAD assessment rates

## P171 Oversedation and ICU-acquired delirium: an observational study

### M Marchesi^1^, N Latronico^1^, F Terranova^1^, S Sala^1^, M Brognoli^2^, GP Nocivelli^2^, R Bertuetti^2^, E De Peri^2^, F Rasulo^1^

#### ^1^Università degli studi di Brescia, Anestesia e Rianimazione, Brescia, Italy; ^2^Spedali Civili di Brescia, Anestesia e Rianimazione, Brescia, Italy

**Introduction:**

Sedation is a significant part of medical treatment in ICU patients. A too deep sedation is associated with a longer time of mechanical ventilation, lung injury, infections, neuromuscular disease and delirium, which can lead to a longer duration of ICU hospitalization, as well as an increase of morbility and mortality. Many patients spend a considerable amount of time in a non-optimal sedation level. A continuous monitoring system of the sedation level is therefore necessary to improve clinical evaluation.

**Methods:**

Our goal was to evaluate the incidence of non-optimal sedation (under and over sedation) comparing the parameters expressed from NGSedLine with clinical evaluations and to correlate oversedation and the incidence of delirium. We have studied a cohort of patients admitted to the ICU of Spedali Civili of Brescia University Hospital requiring continuous sedation for more than 12 hours. In addition to standard monitoring, the patients have been studied using Next Generation Sedline (Masimo). Sedation depth was evaluated through RASS scale and the presence of delirium was evaluated with CAM-ICU scale.

**Results:**

We collected data from 50 adult patients. Our data showed high incidence of oversedation. 45 of our 50 patients had a SR>2 and 48 had a PSI level<30. A logistic regression analysis was performed and it showed statistically significant association between incidence of delirium and the age of the patients (p 0.009). The association between delirium incidence and Suppression Rate time was at the limits of statistics significance (p 0.059) and was statistically significant for non neurocritical patients (p 0.049). Our study didn’t show an association between delirium and the total time of sedation.

**Conclusions:**

Non-optimal sedation is an unsolved problem in ICU, affecting lot of patients, with a major incidence of over-sedation compared to under-sedation. Our study shows an association between SR levels and the incidence of delirium.

## P172 Predictors of delirium after myocardial infarction, insights from a retrospective registry

### M Jäckel, V Zotzmann, T Wengenmayer, D Dürschmied, C Von zur Mühlen, P Stachon, C Bode, DL Staudacher

#### Heart Center Freiburg University, Department of Cardiology and Angiology I, Freiburg, Germany

**Introduction:**

Delirium is a common complication on intensive care units. Data on incidence and especially on predictors of delirium in patients after acute myocardial infarction (MI) are rare. By analyzing all patients after acute MI, we aim to identify incidence and potential risk factors for delirium.

**Methods:**

In this retrospective study, all patients hospitalized for acute MI treated with coronary angiography in an university hospital in 2018 were included and analyzed. Incidence of Delirium within the first 5 days of care attributed to the MI and was defined by a NuDesc Score ≥2, which is taken as part of daily care three times a day by especially trained nurses. This research is authorized by ethics committee file number 387/19.

**Results:**

626 patients with acute MI (age 68.5±13.3 years, 260 STEMI, mortality 3.4%) were analyzed. Delirium occurred in 70 (11.2%) patients and was associated with a longer hospital stay (12±15.9d vs 5.5±4.3d, p<0.001). Patients with delirium were significantly older than patients without (76.3±11.14 vs. 67.5±13.10 years, p<0.001) and had more often preexisting neurological diseases (24.3% vs. 10.8%, p<0.001) and dementia (11.4% vs. 1.4%, p<0,001). Multivariate logistic regression analysis suggested that odds ratio for delirium was higher in patients after resuscitation OR 7.5 (95% CI 2.1-26.7), preexisting dementia OR 28.9 (CI 3.1-268) and in patients with alcohol abuse OR 18 (CI 2.7-120). While maximum lactate was also connected to delirium OR 1.4 (CI 1.1-1.9), infarct size or type had no effect on the incidence of delirium.

**Conclusions:**

In patients with MI, delirium is frequent. Incidence is associated with clinical instability and preexisting neurological diseases rather than infarct size.

## P173 Incidence and risk factors of delirium in surgical intensive care unit

### MA Ali, B Saleem

#### Aga Khan University, Anaesthesia, Karachi, Pakistan

**Introduction:**

Delirium in the critically ill patients is common and distressing. The incidence of delirium in the ICU ranges from 45% to 87%. Although delirium is highly common among intensive care patients, it is mostly underreported. To date, there have been limited data available related to prevalence of delirium in surgical patients. In a study published in 2008, the risk was observed 73% in surgical and trauma patients [1]. The purpose of this study was to find out the incidence and associated risk factors of delirium in surgical ICU (SICU) of a tertiary care hospital.

**Methods:**

We conducted prospective observational study in patients with age more than 18 years and who were admitted to the surgical ICU for more than 24 hours in Aga Khan University Hospital from January 2016 to December 2016. Patients who had preexisting cognitive dysfunction or admitted to ICU for less than 24 hours were excluded. Delirium was assessed by Intensive Care Delirium Screening Checklist ICDSC. Incidence of delirium was computed and univariate and multivariable analyses were performed to observe the relationship between outcome and associated factors.

**Results:**

Delirium was observed in 19 of 87 patients with an incidence rate of 21.8%. Multivariable analysis showed that COPD, pain >4 and hypernatremia were strong predictors of delirium. Midazolam [aOR=7.37; 95%CI: 2.04-26.61] and Propofol exposure [aOR=7.02; 95%CI: 1.92-25.76] were also the strongest independent predictors of delirium while analgesics exposures was not statistically significant to predict delirium in multivariable analysis.

**Conclusions:**

Delirium is significant risk factor of poor outcome in surgical intensive care unit. . There was an independent association between pain, sedation, COPD, hypernatremia and fever in developing delirium

**References:**

1. Pandharipande P et al. J Trauma 65:34–41, 2008

## P174 Evaluation of delirium risk factors in intensive care patients

### S Erel^1^, E Macit Aydın^1^, B Nazlıel^2^, L Karabıyık^1^

#### ^1^Gazi University School of Medicine, Anesthesiology and Intensive Care, Ankara, Turkey; ^2^Gazi University School of Medicine, Neurology and Intensive Care, Ankara, Turkey

**Introduction:**

Delirium is an acute mental syndrome which may cause negative consequences if it is misdiagnosed [1,2]. The aim of this study was to determine the incidence of delirium in different intensive care units and reveal the risk factors.

**Methods:**

The study was performed with 212 patients hospitalized in intensive care units of anesthesia, neurology and general surgery departments. Written informed consent was obstained from patients or relatives. Delirium screening test was performed twice daily with CAMICU (Confusion Assessment Method for the ICU). Patients who met the study criterias, were evaluated for the possible risk factors of delirium and the data was recorded daily. Patients were reevaluated after the treatment.

**Results:**

The incidence of delirium was 32.5%. Delirium was found to increase with the length of stay (p <0.001). The mean age of the patients with delirium was 67.46. this was higher than the patients without delirium (52.48) (p<0.001). Visual impairment (p<0.001), hearing impairment (p=0.001), educational status (p=0.024), hypertension (p=0.002), mechanical ventilation (p = 0.001), oxygen demand (p=0.002), midazolam infusion (p=0.025), propofol infusion (p=0.042), infection (p <0.001), SOFA (p = 0.001), APACHE II (p <0.001), nasogastric catheter (p=0.012), aspiration (p <0.001), number of aspirations (p<0.001), enteral nutrition (p<0.025), albumin (p=0.025), steroid (p=0.024), hypercarbia (p=0.039) hypoxia (p=0.039), sleep disturbance (p<0.001) were found risk factors for delirium. Oral nutrition (p<0.001) and mobilization (p=0.003) were found to prevent delirium development.

**Conclusions:**

Various factors are important in the development of delirium. These risk factors should be considered in reducing the incidence of delirium in intensive care units.

**References:**

1. Salluh JI et al. BMJ 350:2538, 2015

2. Kotfis K et al. Anaesthesiol Intensive Ther 50:160-67, 2018

## P175

**Withdrawn**

## P176 Delirium tremens in ICU: a descriptive study of clinical practices and patients complications

### M Geslain, J Le Roy, P Aries, X Chapalain, T Gargadennec, O Huet, E L´Her, P Bailly

#### CHRU Brest, Brest, France

**Introduction:**

Alcohol use disorders (AUDs) is very common in ICU patients (up to 20%). An unplanned and brutal stop of alcohol consumption, as it can occur during ICU admission, may lead to an alcohol withdrawal syndrome (AWS). The most severe clinical manifestation of AWS is described as delirium tremens (DT). There are no current guidelines available for AWS treatment in ICU. The study’s aim was to describe the clinician’s practices for DT treatment and the outcome of DT in ICU patients.

**Methods:**

Observational retrospective cohort study in two ICUs of a university-affiliated, community hospital in France. Patient diagnosed for DT during their ICU stay, as defined by DSM-V classification, were enrolled in the study.

**Results:**

61 patients with DT were included between 2014 and 2017. Benzodiazepines was administered to 23% of the patients in order to prevent an AWS. As associated measures, vitamin therapy was administered to 83% of the patients and 59% had an increased fluid intake (mean 2.5L+/-0.7). Concerning the curative approach of AWS, the treatment’s heterogeneity was notable. There was a high frequency of treatment’s association (66% of the patients), every patient had benzodiazepines and the use of second line treatments such as neuroleptic, alpha-2 agonist, propofol was variable (Figure 1).

Complications of DT were the following:

- Need for mechanical ventilation due to unmanageable agitation or acute respiratory distress (33% of the patients)
- Self inflicted injuries such as pulling out of central lines, tubes, surgical drain (46%)
- Falls (7%).
- Seizures (33%).

**Conclusions:**

Delirium tremens is a severe complication of an untreated AWS, which can lead to serious adverse events in ICU. The current lack of evidence concerning the management of AWS in ICU probably explains the heterogeneity of treatments. Given the potential severity of AWS in ICU, further evidences are required to optimize care of AWS in ICU patients.

**Fig. 1 (abstract P176).**
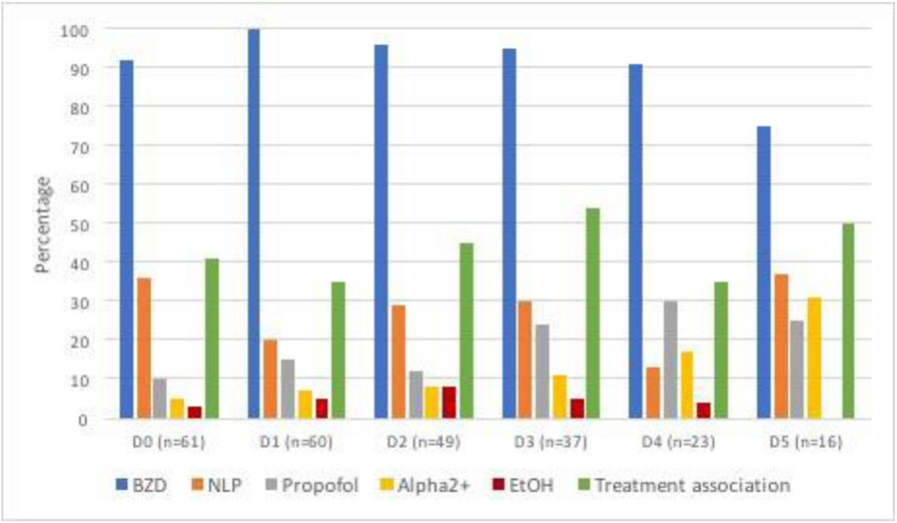
DT treatment in ICU from D0 to D5. Most of the patients presenting a DT during their ICU stay were treated with benzodiazepine. A significant number of them received association of different molecules regardless the absence of evidence in the literature

## P177 The incidence and related risk factor of delirium in surgical stepdown unit

### S Yoon^1^, S Yang^2^, G Cho^2^, H Park^2^, K Park^2^, J Ok^2^, Y Jung^2^

#### ^1^Asan medical center, Nursing department, Seoul, South Korea; ^2^Asan Medical Center, Seoul, South Korea

**Introduction:**

Step Down Units (SDUs) provide an intermediate level of care between the ICU and the general medical-surgical wards. The Critically ill patients who are in recovery after long-term intensive care or who require monitoring after acute abdominal surgery are admitted to SDUs. Delirium in critically ill patient is common and leads to poor clinical outcomes. It is, however, preventable if its risk factors are identified and modified accordingly.

**Methods:**

To determine risk factors associated with delirium in critically ill patients to admitted surgical SDU at Asan Medical Center. This is retrospective study conducted on critically ill patients who were admitted to the SDU from September 2018 to April 2019 and able to express themselves verbally. Delirium status was determined using the Short-CAM tool. Data were analyzed by SPSS 13.0 software, using t-test, Fisher's exact test and logistic regression.

**Results:**

The incidence of delirium was 32.1%(25 of 78 patients) and hypoactive delirium(12case, 48.0%) was the most commonly assessed, followed by hyperactive delirium(9case, 36.0%), mixed type(4case, 5.1%). Risk factors associated with developing delirium identified from univariate analysis were age(P=0.048), admission via ICU (P=0.041), tracheostomy (P=0.037), chronic heart failure (CHF) (P=0.017), invasive hemodynamic monitoring (P=0.041), heart rate (P=0.037). After adjusted in multivariate analysis; factors those remained statistically significant were old age (RR 1.16, 95% Confidence Interval [CI]=1.07-1.25, P=0.041), having a tracheostomy (RR 2.99, 95% CI=1.48-2.08, P=0.028), CHF (RR 4.01, 95% CI=1.98-5.23, P<0.001), and chronic renal failure (RR 4.45, 95% CI=1.12-3.23, P<0.001). Those were significant risk factors of delirium in SDU.

**Conclusions:**

We identified risk factors consistently associated with incidence of delirium following admitted to surgical SDU. These factors help to focus on patients at risk of developing delirium, and to develop preventive interventions that are suitable for those patients.

## P178 Validation of the prediction model for delirium in ICU patients (pre-deliric model) in mechanically ventilated Japanese patients with sepsis

### K Miyamoto^1^, T Nakashima^1^, N Shima^1^, S Kato^1^, Y Kawazoe^2^, Y Ohta^3^, T Morimoto^4^, H Yamamura^5^

#### ^1^Wakayama Medical University, Department of Emergency and Critical Care Medicine, Wakayama, Japan; ^2^Tohoku University Graduate School of Medicine, Division of Emergency and Critical Care Medicine, Sendai, Japan; ^3^Hyogo College of Medicine, Division of General Medicine, Department of Internal Medicine, Nishinomiya, Japan; ^4^Hyogo College of Medicine, Department of Clinical Epidemiology, Nishinomiya, Japan; ^5^Osaka Prefectural Nakakawachi Emergency and Critical Care Center, Higashiosaka, Japan

**Introduction:**

Patients with sepsis frequently develop delirium during their intensive care unit (ICU) stay, which is associated with increased morbidity and mortality. The prediction model for delirium in ICU patients (PRE-DELIRIC model) was developed to facilitate the effective preventive strategy of delirium [1]. However, the PRE-DELIRIC model has not yet been validated enough outside Europe and Australia. The aim of this study is to examine the external validity of the PRE-DELIRIC model to predict delirium using Japanese cohort.

**Methods:**

This study is a post hoc subanalysis using the dataset from previous study in nine Japanese ICUs, which have evaluated the sedative strategy with and without dexmedetomidine in adult mechanically ventilated patients with sepsis [2]. These patients were assessed daily throughout ICU stay using Confusion Assessment Method-ICU. We excluded patients who were delirious at the first day of ICU, were under sustained coma throughout ICU stay and stayed ICU less than 24 h. We evaluated the predictive ability of the PRE-DELIRIC model to measure the area under the operating characteristic curve. Calibration was assessed graphically.

**Results:**

Of the 201 patients enrolled in the original study, we analyzed 158 patients in this study. The mean age was 69.4 ± 14.0 years and 99 patients (63%) were male. Delirium occurred at least once during their ICU stay in 63 patients (40%). To predict delirium, the area under the receiver operating characteristics curve of the PRE-DELIRIC model was 0.60 (0.50 to 0.69). Graphically, the prediction model was not well-calibrated (Figure 1).

**Conclusions:**

To predict delirium in Japanese ICUs, we could not show the well discrimination and calibration of the PRE-DELIRIC model in mechanically ventilated patients with sepsis.

**References:**

1. van den Boogaard M et al. Intensive Care Med 40:361-9, 2014

2. Kawazoe Y et al. JAMA 317:1321-8, 2017

**Fig. 1 (abstract P178).**
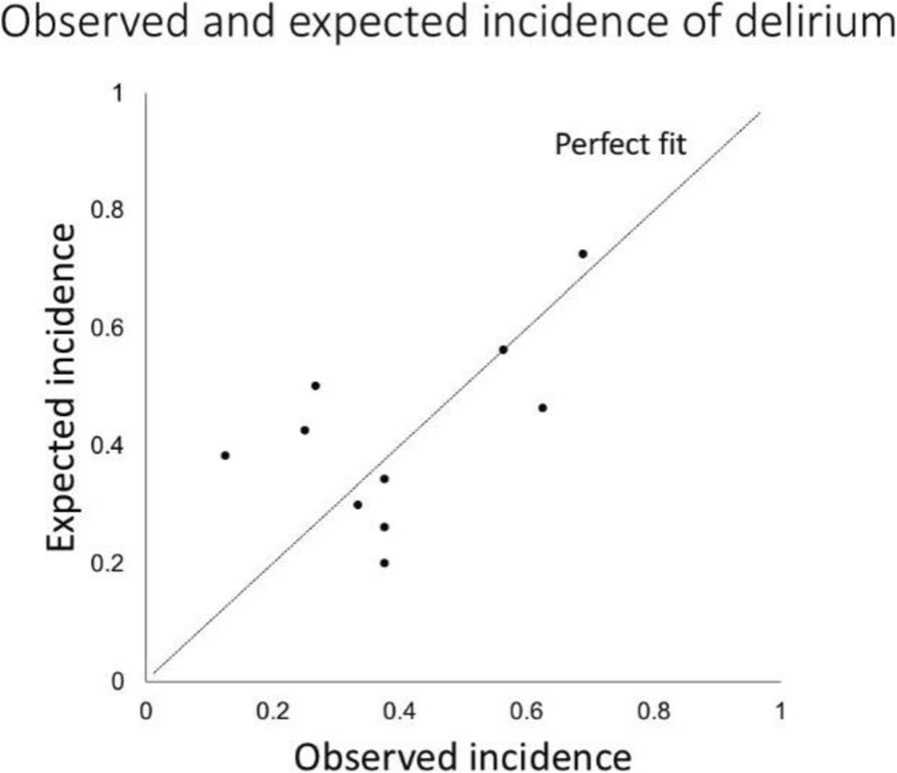
Observed and expected incidence of delirium

## P179 The effect of dexmedetomidine on the duration of delirium and the use of narcotic analgesics in elderly patients with femur fracture

### A Konkayev^1^, N Bekamagambetova^2^, M Konkayeva^3^

#### ^1^Astana Medical University, Anaesthesiology and Intensive Care, Nur-Sultan, Kazakhstan; ^2^Trauma institution, Nur-Sultan, Kazakhstan; ^3^Astana Medical University, Nur-Sultan, Kazakhstan

**Introduction:**

Delirium is a serious and common complication and in some cases it treatment is difficult. Aim of the study was an evaluation of the prevalence, structure of delirium and efficacy of dexmedetomidine and haloperidol sedation in geriatric patients after femur fracture.

**Methods:**

After local ethic committee approval 207 case-records of geriatric patients with femur fracture in the period from 2017 to 2018 in the Institute of traumatology and orthopedics in Astana were analyzed. Patients was divided for 2 groups: in D – patients with delirium treated by i/v dexmedetomidine (0.2-1.4 mkg/kg per hour), in G group patients with delirium treated by i/v galoperidol (0.10-0.15 mkg/kg). Delirium was assessed by RASS at day of permission and every day at 8 a.m. The prevalence, structure of delirium and efficacy of sedation were analysed.

**Results:**

By anthropometric and gender characteristics of the group did not differ. The average age in the D-group with delirium was 81.8±0.9 years old, which was comparable to the G-group — 79.7±0.7 years old (p = 0.06). All study participants had similar comorbidities. Delirium in all patients debuted at 2.0±1.4 days, with an average duration of 2.2±1.2 days. The effect of dexmedetomidine was better and expressed in 52% decrease in the duration of delirium in compare to haloperidol (p <0.05). Dexmedetomidine provided a more controlled and safe sedation compared with haloperidol. The average consumption of narcotic analgesics in the subgroup with dexmedetomidine was two times less than in the subgroup with haloperidol. Thus, the average consumption of trimeperidine hydrochloride in patients of group D was 6.9 mg versus 14.1 mg in group G (p = 0.004).

**Conclusions:**

In gerontological patients with femur fracture treatment delirium by dexmedetomidine was more effective in compare with haloperidol. When using dexmedetomidine, the consumption of narcotic analgesics in postoperative period was 50% less than with haloperidol.

## P180 Live music therapy in intensive care unit

### MC Soccorsi^1^, C Tiberi^1^, G Melegari^1^, J Maccieri^2^, F Pellegrini^2^, E Guerra^1^, A Marudi^1^, E Bertellini^1^

#### ^1^Policlinico Teaching Hospital, Modena, Department of Anaesthesia and Intensive Care, Modena, Italy; ^2^Policlinico Teaching Hospital, Modena, School of Anaesthesia and Intensive Care, Modena, Italy

**Introduction:**

Intensive care units (ICU) are not comfortable for patients, relatives or next of kin. In the last years many news approaches were described to implement the humanization of medical treatments. The positive effect of music therapy in ICU is well described, especially reducing delirium risk [1]. The aim of this paper is describing the effect in patients and their family of a music live performance in ICU.

**Methods:**

After Ethical Committee approval (Procedure AOU 0018356/18, Italy) for three months (November 2018-January 2019) patients in ICU were treated twice a week with live music therapy performed by Coral Vecchi-Tonelli of Modena, Italy (Fig. 1). Data were collected all awake and conscious patients. Vitals parameters, GCS, RAAS and CAM ICU were collected before, during and after the treatment, at every performance. After the treatment a feedback questionnaire were given to patients and to next of kin.

**Results:**

18 subjects were enrolled in the research with mean age of 66.66 years old, delirium rate before the treatment was 16.38% later 15.38%, RAAS does not show any difference. Over 85% of patients were satisfied, and relatives felt less anxiety. We recorded also a satisfaction also in relatives not enrolled.

**Conclusions:**

The study does not demonstrate a delirium risk reduction for the small sample and the length treatment, anyway it was recorded a low delirium rate. The safety and the potential effect of music therapy are well known, surely the research underlines the feeling of patients and their next of kin: ICU is the most stressful setting for admitted patients and its humanization is a current topic for medical literature. Live performances could be an entertainment moment and probably create a moment of an interaction among patients, their family and medical and nurse: ICU become more Human. The high level of satisfaction push us to continue this experience.

**References:**

1. Mofredj A et al. J Crit Care 35:195–199, 2016

**Fig. 1 (abstract P180).**
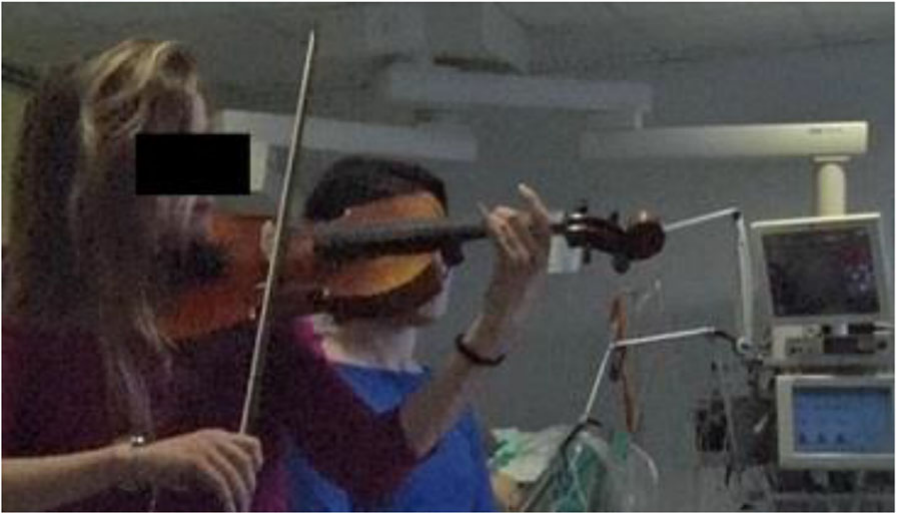
Live performance of music therapy in ICU

## P181 Medical hypnosis and virtual reality glasses are safe and effective tools to alleviate pain and anxiety in patients undergoing medical procedures

### EK Okur kavak^1^, G Van Berlaer^2^, M Diltoer^1^, M Malbrain^1^

#### ^1^Universitair Ziekenhuis Brussel, Intensive Care, Brussels, Belgium; ^2^Universitair Ziekenhuis Brussel, Emergency and Disaster Medicine, Brussels, Belgium

**Introduction:**

Patients undergoing medical procedures benefit from distraction techniques to reduce the need for drugs alleviating pain and anxiety. This study investigates if medical hypnosis or virtual reality glasses (VRglasses) as adjuvant method reduces the need for additional drugs.

**Methods:**

In a prospective, randomized, interventional trial, patients undergoing procedures were stratified in four age groups, and randomly assigned into three arms by means of a closed envelope system. All patients received standard care for pain before the procedure; the control group received further drugs for pain and stress as indicated by the Visual Analog Scale (VAS; threshold 3/10) and ComfortScore (threshold 14/30), two index groups received either medical hypnosis or VR glasses as a plus before and during the procedure. VAS and Comfort were scored continuously and analysed with the Kruskal-Wallis Test. Patients, parents and healthcare providers scored their satisfaction at the end.

**Results:**

Of 104 included patients 6 to 86 years old, 47% were female. Regardless of age, pain and comfort scores were similar before and at the start of the procedure (VAS 3.7-4.2; Comfort 16-16.7), but as of one minute after starting the procedure, both VAS and Comfort reduced significantly more in both index groups compared to the control (p<0.001), remaining far below the threshold for both pain and stress (Figure 1). There was no advantage of one index group over the other (p=0.43). There were no adverse effects. Patients in the VR group were more satisfied than in the standard group (p=0.02) or in the hypnosis group (p=0.04). There was no significant difference in satisfaction of parents or healthcare providers.

**Conclusions:**

From the very start of the intervention, the application of either medical hypnosis or VR glasses significantly reduces pain and anxiety in patients undergoing medical procedures. More studies are needed but both are promising safe adjuvant tools to standard pharmacological treatment.

**Fig. 1 (abstract P181).**
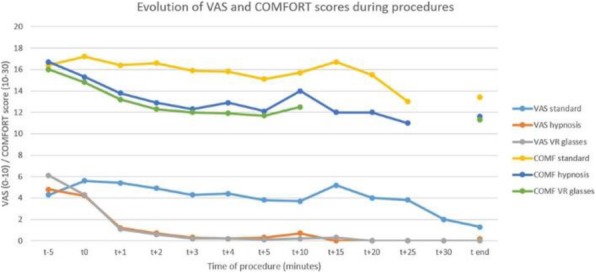
Evolution of VAS and COMFORT scores during procedures

## P182 Music to reduce pain and distress due to emergency care: a randomized clinical trial

### NE Nouira, I Boussaid, D Chtourou, S Sfaxi, W Bahria, D Hamdi, M Boussen, M Ben Cheikh

#### Mongi Slim Academic Hospital, Emergency Department, Tunis, Tunisia

**Introduction:**

Recent clinical studies have confirmed the benefits of music therapy in managing pain and improving quality of care in the emergency department. The aim wasTo evaluate the impact of receptive music therapy on pain and anxiety induced by emergency care

**Methods:**

A randomized controlled study in patients consulting the emergency department. Two groups: the music therapy group; Patients needed venous sampling, peripheral venous catheter or arterial catheter. Will bless ten minutes music therapy by headphones and a second control group of patients with the same care without music therapy. Consent was requested from all participants. The level of pain caused by the act of care was assessed by visual analogic scale. Heart rate, blood pressure and the mood of the patient were assessed before and after emergency care. We assessed patient satisfaction, adverse events. Patients admitted to the emergency room, patients with communication difficulties and non-consenting patients were not included

**Results:**

Two hundred and forty patients were included randomized in both groups, 123 with music therapy and 117 without music therapy, the results showed comparable characteristics between the two groups: demographic data, pathological history, and initial clinical presentation. After the session of music therapy a difference was noted in the evaluation of the mean VAS who was in the group with Music of 2.87 ± 1.82 versus 3.60 ± 2.06 in the control group p< 0.004 CI 95% [-1.23; -0.23], and the mean of diastolic blood pressure which was 71, 07 mmHg in the first group against 74.27 mmHg for the control group p = 0.048 CI 95% [-6.37; -0.25]. As for the mood, the patients were more smiling after the act of care in the group music therapy. All patients were satisfied with their experience and 93% recommend this therapy to their relatives .

**Conclusions:**

Music therapy may reduce pain and anxiety in patients during emergency care.

## P183 Impact of music therapy as non-pharmacological intervention in patients in weaning from mechanical ventilation

### M Carmona^1^, G Pagella^2^, G Federico^3^, R Paffumi^1^, D Minzer^3^, G Zakalik^1^

#### ^1^Hospital Luis Carlos Lagomaggiore, UCI, mendoza, Argentina; ^2^Hospital Luis Carlos Lagomaggiore, Terapia intensiva, Mendoza, Argentina; ^3^Hospital Luis Carlos Lagomaggiore, mendoza, Argentina

**Introduction:**

The music therapy is the intervention of music and/or its elements to achieve individual goals within a therapeutic.the music has proved to have positive physiological and psychological effects on patients [1]. Patients admitted to the Intensive Care Unit (ICU) experience anxiety and stress even when sedated, negatively influencing recovery [2].

**Methods:**

Two groups are established, a music therapy group (MG) and a control group (CG). The first one undergoes music therapy interventions, it consists of 10-minutes sessions of live music. Patients of the GC will receive the usual treatment established by the service protocol for weaning management and the data are collected during the same time interval. Data collection includes Mean arterial pressure (MAP), heart rate (HR), respiratory rate (RR), oxygen saturation (SaO2) and temperature (T).

**Results:**

A total of 28 patients were recruited, of which 6 patients had to be excluded for meeting any of the exclusion criteria (n=22). Of which 13 (n=13) were randomized in the GM and the rest to the GC (n=9) IC95%. Regarding delirium in GM 9 (40.9%) presented a POSITIVE CAM-ICU, while in the CG were 13 (59.9%) (P=0.09). When analyzing the variables in the CG and GM, it was observed that there were no differences with respect to HR, RR and MAP variable (Figure 1).

**Conclusions:**

According to the results, we can say that music therapy as a non-pharmacological strategy for management of anxiety and delirium in patients of critical care units, might be an useful tool for the management of patients in weaning of mechanical ventilation

**References:**

1. Sliwka A et al. Complement Ther Med 22:756–766, 2014

2. Dijkstra BM et al. J Clin Nurs 19:1030-9, 2010

**Fig. 1 (abstract P183).**
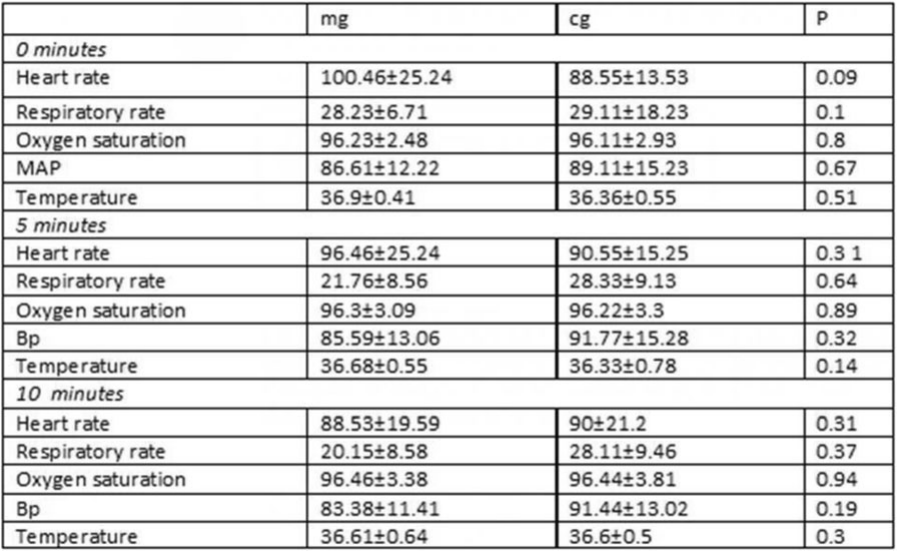
Comparison between MG and CG

## P184 Association between severity and coagulopathy in patients with anaphylaxis

### S Yamaga^1^, Y Yanase^2^, K Ishii^2^, K Hosokawa^1^, S Ohshimo^1^, N Shime^1^, M Hide^2^

#### ^1^Hiroshima University, Department of Emergency and Critical Care Medicine, Hiroshima, Japan, ^2^Hiroshima University, Department of Dermatology, Hiroshima, Japan

**Introduction:**

Coagulopathy and basopenia are common features of anaphylaxis, but the role of coagulopathy in anaphylaxis remains uncertain. The aim of this study is to evaluate the association between coagulopathy and clinical severity or basopenia in patients with anaphylaxis.

**Methods:**

We conducted a single-center, retrospective study of patients with anaphylaxis about their coagulopathy. Levels of fibrin degradation products (FDP) and D-dimer were analyzed with the cause of anaphylaxis, clinical symptoms, medications and outcomes. We also studied the levels of intracellular histamine as a biomarker of basophil degranulation in the peripheral blood in relation to FDP and D-dimer.

**Results:**

In total, sixty-nine patients were enrolled to the study, and the levels of intracellular histamine were analyzed in 14 patients. The symptoms included respiratory failure (n=47), shock (n=56), abdominal impairment (n=20), and consciousness disturbance (n=37). Thirty-two patients needed continuous intravenous vasopressors for refractory shock. The increase of FDP was significantly associated with consciousness disturbance (p=0.029) and refractory shock (p<0.0001). The increase of D-dimer was also significantly associated with refractory shock (p=0.0066). There was no correlation between the levels of intracellular histamine and either of FDP or D-dimer (p=0.13 and p=0.16, respectively).

**Conclusions:**

The increase of FDP and D-dimer were associated with severe symptoms of anaphylaxis, while they were not correlated with intracellular histamine. These results suggest that anaphylaxis is closely associated with coagulopathy in a mechanism which is different from basophile degranulation in anaphylaxis.

## P185 Cardiac manifestations of H1N1 infection in a Greek ICU population

### E Nanou^1^, P Vasiliou^1^, E Tsigou^2^, V Psallida^1^, E Boutzouka^2^, V Zidianakis^1^, G Fildissis^1^

#### ^1^Agioi Anargiroi Hospital, Attiki, Greece; ^2^Agioi Anargiroi Hospital, ICU, Attiki, Greece

**Introduction:**

Cardiovascular involvement in influenza infection occurs through direct effects on the myocardium or through exacerbation of pre-existing cardiovascular disease [1]. The aim was to study cardiac manifestations in all pts admitted to the ICU with severe influenza’s attack.

**Methods:**

Clinical, laboratory, electrocardiographic, echocardiographic and hemodynamic data were retrospectively recorded in all pts admitted to the ICU due to influenza infection (winter 2018-spring 2019). Diagnosis was established by PCR on bronchial aspirates the next 7 days after admission. Myocardial injury was defined by troponin levels >116 pg/ml (10 fold ULN). Left ventricular systolic dysfunction was defined as EF <50% and was characterized as either global or regional. Hemodynamic monitoring by transpulmonary thermodilution method (PICCO) was recorded in pts with shock (norepinephrine >0.1 μg/kg/min). Values are expressed as mean±SD or as median (IR).

**Results:**

Nine pts (5 males) with a mean age 49.78±17.01 years, APACHE II 19±5.29 and SOFA score 10.50±2.93 were assessed. ICU admission was due to ARDS (7) and COPD exacerbation (2). ICU LOS was 24.44±14.19 days and mortality rate was 18%. No history of vaccination or coronary heart disease was referred. Results are shown in table 1. Levosimendan was administered in 2 pts with severe cardiogenic shock. In all survivors, shock and indices of myocardial dysfunction subsided till discharge. Coronary angiography was performed in 1 pt showing no abnormalities. Mortality was attributed to septic shock and multi-organ failure.

**Conclusions:**

Myocardial involvement, though common in influenza pts admitted to the ICU, didn’t contribute to a dismal prognosis.

**References:**

1. Fagnoul D et al. J Crit Care 28: 321–327, 2013

**Table 1 (abstract P185).** Hemodynamic indices in H1N1 patients

Elevated troponin	N=7 (77.78%)
Systolic dysfunction	N=4 (36%)
ECG abnormalities	N=7 (77.78%)
CI (l/min/m2)	2.86±0.13
GEF (%)	20.50±4.95
GEDI (ml/m2)	824±64.1
SVRI (dyn/sec/cm/m2)	2179±339
EVLWI (ml/kg)	11.33±5.13

## P186 Levosimendan improves oxidative balance in low cardiac output patients

### E Grossini^1^, S Farruggio^1^, V Bolzani^2^, L Rossi^2^, E Monaco^3^, P Pollesello^4^

#### ^1^Università Del Pienmonte Orientale, Department of Translational Medicine, Novara, Italy; ^2^Azienda Ospedaliero Universitaria, Cardiology Division, Novara, Italy; ^3^Azienda Ospedaliero Universitaria, Intensive Care Unit, Novara, Italy; ^4^Orion Corporation Orion Pharma, Critical Care Propretary Products, Espoo, Finland

**Introduction:**

The cardioprotective effects of levosimendan could be related to the modulation of oxidative balance. We aimed to examine the effects of levosimendan in patients with cardiogenic shock or with ejection fraction (EF) lower than 30% on cardiac systo-diastolic function and plasma oxidants/antioxidants (glutathione, GSH; thiobarbituric acid reactive substances, TBARS).

**Methods:**

In 4 patients undergone coronary artery bypass grafting or angioplasty, cardiovascular parameters were measured at T0 (before the beginning of levosimendan, 0.1mcg/Kg/min), T1(1 h after the achievement of the therapeutic dosage of levosimendan), T2 (at the end of levosimendan infusion), T3 (at 72 h after the end of levosimendan infusion), T4 (at the end of cardiogenic shock). The same time-course was followed for plasma GSH and TBARS measurements.

**Results:**

We found an improvement in cardiac output, cardiac index and systolic arterial blood pressure. EF increased from mean 25% to 45%. A reduction of central venous pressure and wedge pressure was also observed. Moreover, indices of diastolic function were improved by levosimendan administration (E/E’ from 14 to 6; E/A from >1 to <1) at early T2. It is to note that an improvement of GSH and TBARS was observed early after levosimendan administration (T1), as well (Figure 1).

**Conclusions:**

The results obtained have shown that levosimendan administration can regulate oxidant/antioxidant balance as an early effect in low cardiac output patients. The modulation of oxidative condition could be speculated to play a role in exerting the cardio-protection exerted by levosimendan in those patients.

**Fig. 1 (abstract P186).**
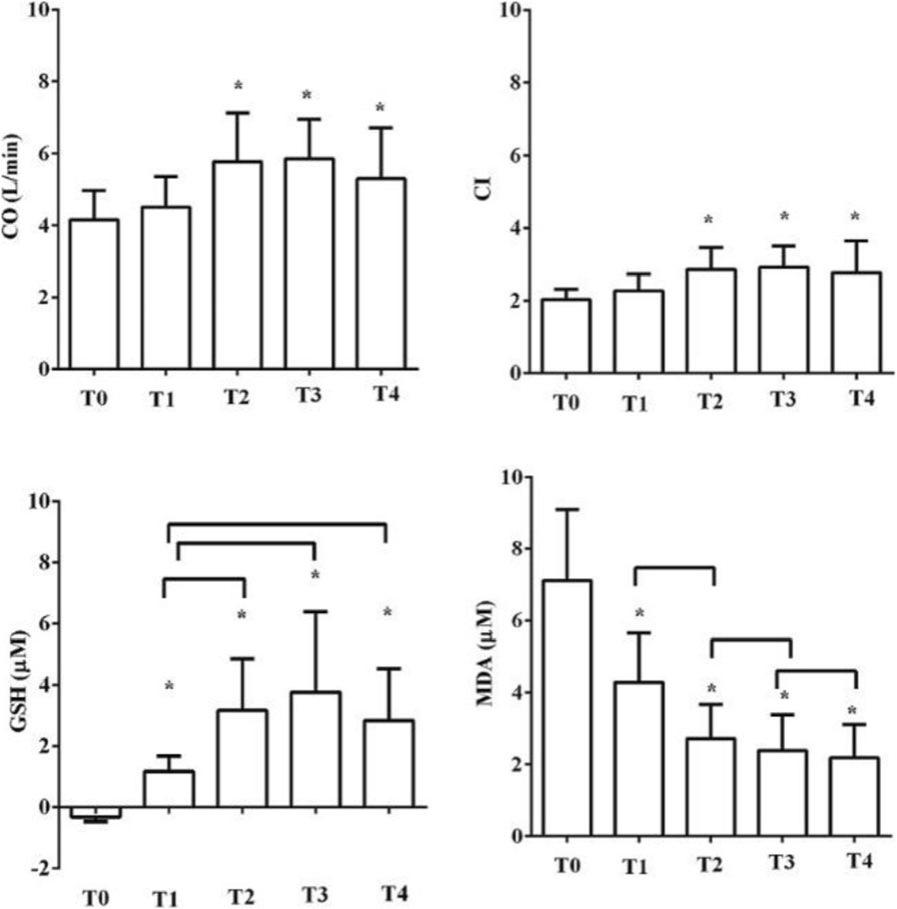
Effects of levosimendan on cardiac output (CO), cardiac index (CI), glutathione (GSH) and malonyldialdeide (MDA, which is representative of thiobarbituric acid reactive substances (TBARS). * p<0.05 vs T0. Square brackets indicate significance between groups (p<0.05)

## P187 An analysis of the prognostic ability of noradrenaline dose in patients with septic shock

### M Chotalia^1^, M Bangash^2^, T Matthews^2^, D Parekh^2^, J Patel^2^

#### ^1^University Hospitals Birmingham, NHS Foundation Trust, Critical Care and Anaesthesia, Birmingham, United Kingdom; ^2^University Hospitals Birmingham, NHS Foundation Trust, Birmingham, United Kingdom

**Introduction:**

This study analyses the prognostic ability of noradrenaline dose in the first four days following the onset of septic shock.

**Methods:**

Patients with a diagnosis of septic shock (Sepsis 3.0) admitted to the ICU at Queen Elizbeth Hospital, Birmingham between April 2016 and July 2019 were included. The primary outcome was 90-day mortality. Continuous data is presented as mean (SEM). Categorical data is presented as % and analysed using a chi-squared test. A p value of <0.05 was used to determined significance. To allow for multiple comparison between data, a Bonferroni calculation was used to determine significance at a p value of <0.01. Receiver operator characteristic (ROC) curves were generated for noradrenaline dose and area under the curve (AUC) was calculated.

**Results:**

847 patients were admitted with septic shock. The majority (61%) were male, with a mean age of 62 (±0.6) and a 90-day mortality of 43%. Median noradrenaline dose had a fair prognostic ability on day 1 (AUC = 0.754 [0.714 – 0.794]) and good prognostic ability on day 2 (AUC = 0.844 [0.810 – 0.878]) (Figure 1). A noradrenaline dose of 0.25μg/kg/min on day 1 and 0.15 μg/kg/min on day 2 had a 70% sensitivity and 70% specificity for mortality. Median noradrenaline dose was an excellent prognostic indicator on day 3 (AUC =0.911 [0.878 – 0.943]) and day 4 (AUC = 0.908 [0.879 – 0.948]). A noradrenaline dose of 0.1μg/kg/min on day 3 and 0.05 μg/kg/min on day 4 had a 90% sensitivity and 90% specificity for mortality. In patients who survived, vasopressors were weaned off within 4 days in 90% of cases. In those who died, death occurred within 4 days 88% of the time.

**Conclusions:**

Noradrenaline dose has a good prognostic ability in septic shock. In this septic shock cohort, the majority of patients that died did so within 4 days. Furthermore, the majority of survivors were weaned off vasopressors within 4 days. Therefore, by day 3 and 4, small doses of noradrenaline had an excellent prognostic ability for septic shock.

**Fig. 1 (abstract P187).**
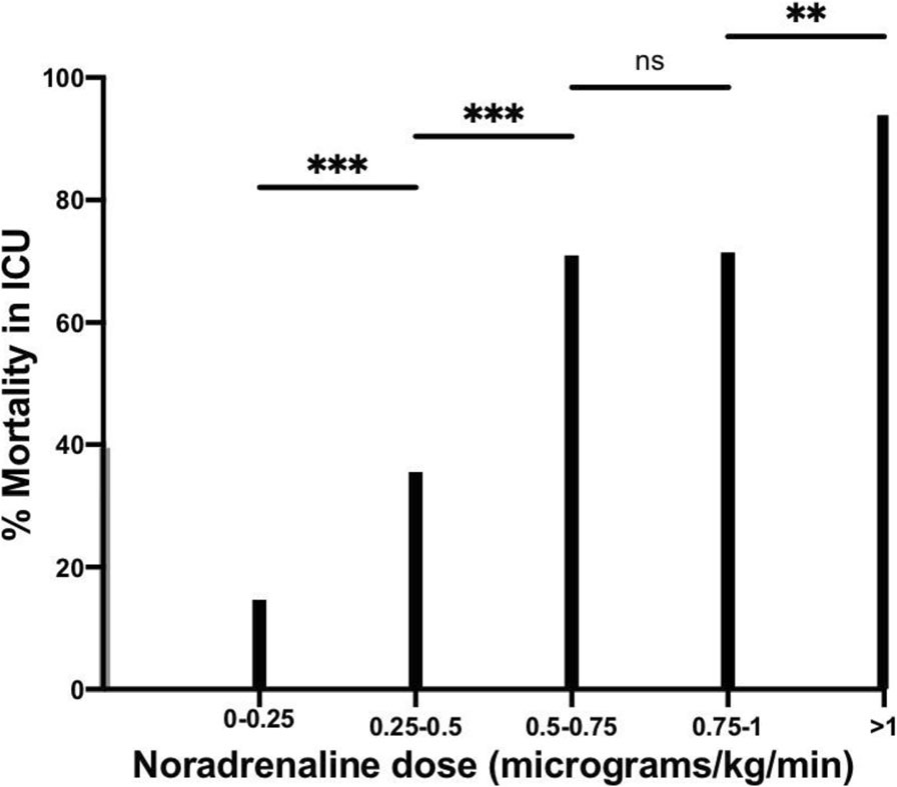
Maximum noradrenaline dose (micrograms/kg/minute) from onset to day 4 of septic shock and % 90-day mortality. (**: p = 0.005; ***: p <0.0001; ns = non-significant)

## P188 Early vasopressor use in the emergency department is associated with survival in patients with septic shock

### M Chotalia^1^, M Bangash^2^, T Matthews^2^, D Parekh^2^, J Patel^2^

#### ^1^University Hospitals Birmingham, NHS Foundation Trust, Critical Care and Anaesthesia, Birmingham, United Kingdom; ^2^University Hospitals Birmingham, NHS Foundation Trust, Birmingham, United Kingdom

**Introduction:**

This study evaluates whether vasopressor use in the emergency department (ED) and vasopressor use within four hours of arrival to ED is associated with survival in patients with septic shock.  The optimal timing of vasopressor administration in septic shock is unknown. Emerging evidence suggests early administration in non-intensive care settings may improve outcomes [1].

**Methods:**

Patients with a diagnosis of septic shock (Sepsis 3) who were admitted to intensive care directly from the emergency department (ED) between April 2016 and July 2019 at Queen Elizabeth Hospital, Birmingham were included.  The primary outcome was 90-day mortality. Early vasopressor use was defined as the administration of metaraminol or noradrenaline within 4 hours from arrival in ED. Continuous data is presented as mean (SEM) and analysed using an unpaired t-test. Categorical data was analysed using Fishers exact test.

**Results:**

87 patients had septic shock and were admitted to intensive care directly from ED. Patients were 61% male, aged 64.7±1.9 years, and had a 44% 90-day mortality. Seventy-nine percent of patients had vasopressors in ED; 56% within 4 hours. The first vasopressor was metaraminol in 60% of cases. Early vasopressor use was associated with a lower mortality than late vasopressor use (33% vs. 58%; RR 0.56 [0.35-0.92]; p = 0.029). Vasopressor use in the ED was associated with a lower mortality than no vasopressor use in the ED (38% vs 67%; RR 0.56 [0.36-0.88]; p = 0.035). There was no difference in the median MAP in ED between early or late vasopressor use (64.3 [1.2] mmHg vs 63.8 [2.7] mmHg; p = 0.56). A comparison between patients who survived and died is outlined in Table 1.

**Conclusions:**

Early administration of vasopressors and their use in the emergency department was associated with survival in septic shock. This seemed to be independent of median MAP recorded in the ED.

**References:**

1. Permpikul C et al. Am J Respir Crit Care Med 199:1097-105, 2019.

**Table 1 (abstract P188).** A comparison of early vasopressor use in septic shock patients who survived and died

	Survived (n = 49)	Died (n=38)	p value
Age (years)	63.7 (2.6)	66.0 (2.8)	0.55
Median MAP in ED (mmHg)	64.5 (1.1)	63.9 (1.9)	0.77
Fluid given (mls)	3372 (179)	3394 (158)	0.93
Vasopressor use in ED (%)	88.9% (43/49)	68.4% (26/38)	0.035
Early vasopressor use (%)	67.3% (33/49)	42.1% (16/38)	0.029
Time spent in ED (mins)	401 (27.5)	427 (38.3)	0.57
Time to ITU review (mins)	201 (19.4)	199 (24.1)	0.94

## P189 Prevalence and management of tuberculous cardiac tamponade : experience of Casablanca cardiac care unit

### R Benmalek^1^, H Zahidi^2^, J Hassari^3^, S Arous^3^, E Benouna^3^, R Habbal^3^

#### ^1^CHU Ibn Rochd, Cardiology Department, Casablanca, Morocco; ^2^Cardiology department, CHU Ibn Rochd, Casablanca, zahidi, Casablanca, Morocco; ^3^Cardiology department, CHU Ibn Rochd, Casablanca, Cardiology, Casablanca, Morocco

**Introduction:**

Cardiac tamponade is a life-threatening clinical syndrome caused by an increase in intrapericardial pressure that requires early diagnosis and management. In our Moroccan endemic context, tuberculosis (TB) still remains a real public health issue and its pericardial localization is a serious form of extrapulmonary TB associated with substantial morbidity. However, pericardial effusions causing pericardial tamponade are rare in patients with TB. The aim of our study was to evaluate the prevalence of tuberculous cardiac tamponade, its clinical presentation and evolutive particularities in our cardiac care unit

**Methods:**

We conducted a cross-sectional prospective study from May 2016 to April 2019 including all the patients admitted in our unit for cardiac tamponade. We excluded all the traumatic or post-myocardial infarction forms.

**Results:**

Out of 83 patients, the tuberculous etiology was identified in 15 cases (18,1%), mean age was 34 years, 57,8% were men. 9 patients reported a TB contact in their environment, 5 had a medical history of pulmonary TB. After pericardiocentesis, the liquid was citrine yellow in 6 cases and hematic in 5 patients, no patient underwent surgical drainage in our serie. Mycobacterium tuberculosis was found in the expectorations in 4 cases and ADA was positive in 4 patients. HIV serology was negative in all our patients. A 6 months anti bacillary therapy with isoniazid, rifampin, pyrazinamide, and ethambutol was initiated in all our patients with a good evolution in 7 cases, 2 deaths, 1 chronic constrictive pericarditis, 2 small pericardial effusion and 3 lost to follow-up.

**Conclusions:**

Althought cardiac tamponade is rarely caused by tuberculosis, this condition remains common in endemic countries such as Morocco and affect younger population, hence the importance of a better knowledge of its prevalence and and multidisciplinary management and more importantly the treatment of the underlying cause using Combined antibacillary medication that has shown satisfying results.

## P190 Point-of-care ultrasound teaching during anaesthesia and critical care residency: an Italian national survey

### F Bonomi, S Mongodi, A Colombo, A Stella, S Pregnolato, G Salve, S Bonaiti, A Orlando, F Mojoli

#### Fondazione IRCCS Policlinico San Matteo - Università di Pavia, Anaesthesia and Intensive Care, Pavia, Italy

**Introduction:**

Point-of-care ultrasound (PoCUS) became an invaluable tool in anaesthesia and critical care (ACC) [1]; appropriate training should integrate ACC residency programs [2].

**Methods:**

On-line surveys sent to 3 groups (ACC schools directors, residents and consultants), as approved by CPAR (Collegio Professori Anestesia Rianimazione), on teaching in vascular accesses-VA, lung ultrasound-LUS, transthoracic echocardiography-TTE, focused-assessment sonography for trauma-FAST, transcranial Doppler- TCD, regional anaesthesia-RA, diaphragm ultrasound-DUS.

**Results:**

In 270 survey days the form was filled by 568 residents (30/40 universities) 22 directors, 216 consultants (24/40 universities). For all the groups bedside teaching, followed by frontal lectures, is the most frequent tool for all techniques. LUS and DUS more frequently include research activities; AR e VA usually include simulations. Overall, the most neglected technique is FAST. According to directors and consultants, residents are mentored by consultants (96.2%/ 95.4%) and older residents (46.2%/ 26.9%), while according to residents, consultants/older residents mentor in 72%/ 39.6% of cases, while 48,6% of the training is performed with no mentoring. Ultrasound competences evaluation is performed mostly in everyday bedside activity (>55%) for all groups; however, residents report no evaluation in a higher percentage (37.5 vs.7.7%). Perception on adequacy of the training is displayed in Fig 1. The main perceived limiting factor is the absence of a standardized didactic program, followed by mentor’s availability in residents’ perception and by mentor’s experience in consultants’ one.

**Conclusions:**

PoCUS teaching is present although not optimal and not homogenous in Italian ACC residency schools. Standardisation of residents’ ultrasound curriculum is suggested to improve ultrasound teaching.

**References:**

1. Mojoli F. Am J Respir Crit Care Med. 199:701-714, 2019

2. Stolz LA. Acad Emerg Med 24:353-361, 2017

**Fig. 1 (abstract P190).**
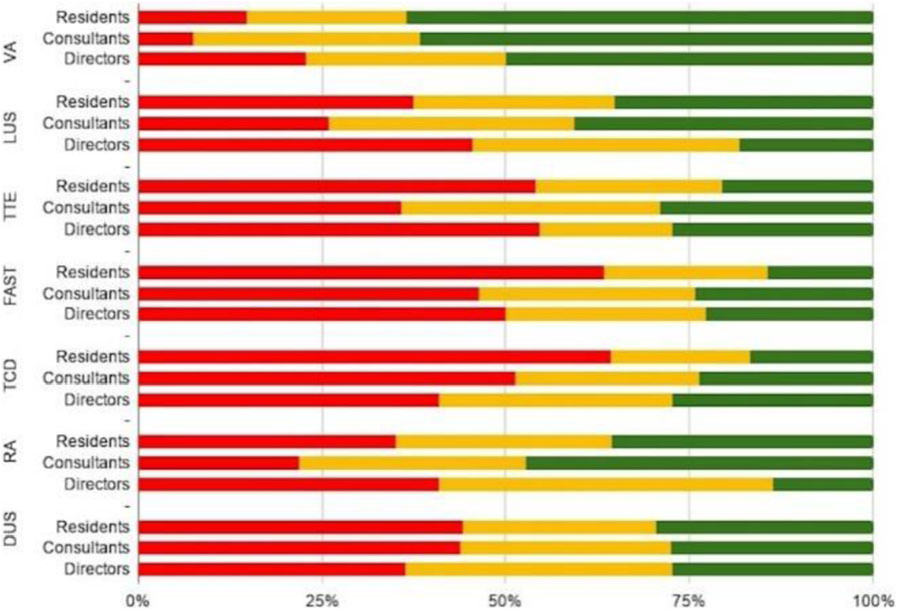
Perception of adequacy of training. Red: to be improved, orange: sufficient, green: adequate

## P191 Confirming central line position through bedside ultrasound

### JD Dillemans, AD Dumoulin, WS Stockman, PL Lormans

#### AZ Delta, Intensive Care, Roeselare, Belgium

**Introduction:**

Ultrasound (US) guidance during Central Venous Catheter (CVC) placement is recommended by guidelines and radiographic confirmation of the position is considered as the golden standard. Nevertheless, there is little literature regarding the feasibility of ultrasonic evaluation of the CVC tip position. The main objective was to examine the accuracy of bedside ultrasound for confirmation of the CVC position and exclusion of pneumothorax compared with chest radiography (CXR).

**Methods:**

The study included a convenience sample of critically ill patients with supradiaphragmatic CVCs and a CXR for confirmation. US is used for direct confirmation of the guidewire in the Internal Jugular (IJV) or Subclavian (SCV) vein and visualizing the guidewire in the right atrium. To evaluate for pneumothorax, “sliding sign” of the pleura was noted on US of the anterior chest.

**Results:**

34 patients have been included, 35% of the catheters have been placed in the SCV and 65% in the IJV. It was possible to confirm the position of the CVC tip for 70.6% (23 correct, 1 incorrect CXR) of 34 cases by transthoracic echocardiography (TTE) (Figure 1). Overall, it was not possible to identify the guide in the right atrium 11 cases (10 false negatives, 2 of them due to the presence of defibrillator leads). Regarding the 1 case where an incorrect position was seen on CXR it was also detected on ultrasound: US of the inserted vein and a negative TTE confirmation. In all cases it was possible to exclude a pneumothorax by US.

**Conclusions:**

These results show that bedside ultrasound might be a feasible technique to confirm the CVC positioning. It is important to note that the level of the operator’s expertise is significant when assessing the feasibility of this method. We only had a limited sample size and the occurrence of only one misplaced catheter. These preliminary results need to be confirmed on a larger scale.

**Fig. 1 (abstract P191).**
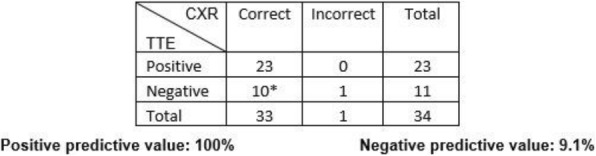
TTE confirmation

## P192 A systematic review on the comparison of the role of internal jugular vein, inferior vena cava and carotid ultrasound measurements in assessment of patients with heart failure

### N Parenti^1^, F Musto^1^, V Pezzilli^1^, G Borelli^1^, T Rada^1^, F Agrusta^2^, S Menetti^2^, M Nuzzetti^2^, E Romboli^1^, M Silingardi^1^

#### ^1^Hospital Maggiore, Internal Medicine, Bologna, Italy; ^2^Policlinico Modena, Internal Medicine, Modena, Italy

**Introduction:**

Recent reports suggested to use internal jugular vein (IJV), inferior vena cava (IVC) and carotid ultrasound measures to confirm congestion and to predict prognosis in heart failure (HF) patients. Our aims were to check the validity of  the previous US measures in predicting HF diagnosis and prognosis.

**Methods:**

This review was based on the PRISMA guideline . The systematic search of the literature published from 1941 through 30 October 2019 explored the PubMed and Cochrane Library databases. Inclusion criteria: studies who investigated the reliability, the accuracy in predicting HF diagnosis and death or re-hospitalization of the IJV, IVC diameter, IVC collapsibility index (IVC-c = IVC max – IVC min / IVC max X 100) and Common Carotid US measures in adult (>18 yrs)  with HF. Five researchers selected studies using inclusion criteria and then assessed their quality using the QUADAS-2 guidelines. The key words for literature search were: common carotid, internal jugular veins, inferior vena cava, ultrasonography and heart failure.

**Results:**

We collected 744 studies: 727 excluded with reasons, 18 studies were included for the final analysis: 4 on IJV, 13 on IVC, 1 on Carotid US. A IJV ratio < 4 predicts death and readmission : HR=2.7-10. A IVC ≥2 cm and IVC-c ≤15% showed an high accuracy in HF diagnosis and a moderate validity in predicting death and re-admission : AUC=0.63-0.78; HR=1.1-5.8 for IVC; AUC=0.63-0.74, HR=0.7-6.8 for IVC-c. The LVET ( time interval between end diastole and the dicrotic notch of Common carotid artery Doppler waveform showed a good validity in predicting HF diagnosis: AUC=0.81 (95% CI, 0.72–0.87).The studies collected showed a moderate quality according to QUADAS-2 guidelines.

**Conclusions:**

Because few reports have been published on this topic the conclusions of this review should be confirmed. The IJV and IVC  ultrasound measures seem to have a moderate accuracy in predicting diagnosis, death and hospitalization in patients with Heart Failure.

## P193 Epidemiology of baseline echocardiography among critically ill patients with sepsis

### MH Senussi^1^, JN Kennedy^2^, M Schmidhofer^1^, O Marroquin^3^, DC Angus^2^, CW Seymour^2^

#### ^1^University of Pittsburgh, Heart & Vascular Institute, Pittsburgh, United States; ^2^University of Pittsburgh, Department of Critical Care Medicine, Pittsburgh, United States; ^3^University of Pittsburgh, UPMC Department of Clinical Analytics, Pittsburgh, United States

**Introduction:**

More than 7-million transthoracic echocardiograms are performed annually in the US. These studies may reveal structural and functional cardiac abnormalities that inform clinical care in the intensive care unit. And yet, little is known about the epidemiology of baseline echocardiography, specifically among patients admitted to intensive care with sepsis.

**Methods:**

We studied electronic health records of 1,076,925 adult patients from 12 hospitals at UPMC from 2013 to 2014. Eligible patients were those with Sepsis-3 at presentation who required intensive care during hospitalization. Demographics, comorbidities, illness acuity, and baseline echo parameters (within 1-year prior to incident sepsis hospitalization) were abstracted. An abnormal echocardiogram was defined as LVEF<50%, LV diastolic dimension >6 cm, moderate/severe RV dysfunction, or moderate/severe valvular stenosis/regurgitation. We compared characteristics of patients who did and did not receive an echo, those with normal and abnormal studies, and patients stratified by in-hospital mortality.

**Results:**

Among 43,086 adults with sepsis, 8,077 (18%) had baseline echocardiographic data within 1-year of admission, on average 131 (SD 103) days prior to presentation (Figure 1). Most septic patients had abnormal echo studies (N=5782, 72%). Of those who died (2,666 (6.2%)), 1,821(68.3%) had no echo data,192(7.2%) had normal and 653 (24.5%) had abnormal studies, compared to 40,420 (93.8%) survivors of which 7,232 (17.9%) had baseline echo, 2,298 (5.7%) had normal, and 5,779(14.3%) had abnormal echocardiograms. Comparing deaths to survivors, reduced EF<39% (15.7% vs 12.6%, p=0.01), severe RV dysfunction (11.1% vs 8.4%, p=0.52), RV dilation (21% vs 17.2%, p<0.01) and severe tricuspid regurgitation (19.4% vs 13.1%, p<0.001) were more common among deaths.

**Conclusions:**

Most septic patients admitted to intensive care have abnormal baseline echocardiography. A reduced LV ejection fraction and severe right sided heart disease were more common among deaths than survivors.

**Fig. 1 (abstract P193).**
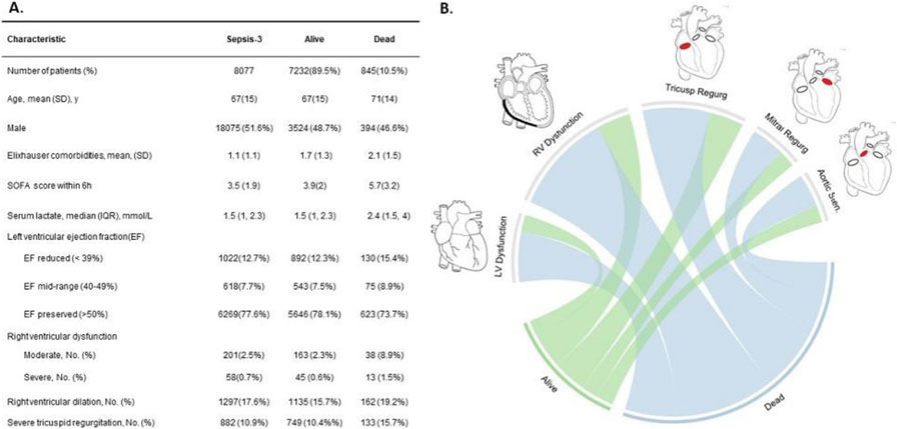
Panel A. Patient baseline characteristics. Panel B. Chord diagram showing abnormal echocardiographic parameters by mortality. The ribbons connect from mortality status to individual echocardiographic abnormalities if the group mean is greater or lesser than the overall mean for the entire cohort. For example, patients who died (light blue) are more likely to have RV dysfunction, LV dysfunction, and tricuspid regurgitation but not aortic stenosis or mitral regurgitation

## P194 Most guidewires used for central venous catheterization of the right subclavian vein in adults are too short; a CT-based observational study

### M Adrian^1^, P Bengtsson^2^, O Borgquist^1^, G Bozovic^2^, T Kander^1^

#### ^1^Skåne University Hospital, Anaesthesia and Intensive Care, Lund, Sweden; ^2^Skåne University Hospital, Diagnostic Radiology, Lund, Sweden

**Introduction:**

Central venous catheter (CVC) misplacement occurs more frequently after cannulation of the right subclavian vein compared to the other sites for central venous access. Misplacement can be avoided with ultrasound guidance by using the right supraclavicular fossa view to confirm correct guidewire J-tip position in the lower part of the superior vena cava. However, retraction of the guidewire prior to the CVC insertion may dislocate the J-tip from its desired position, thereby increasing the risk of CVC misplacement. The aim of this study was to determine the minimal guidewire length needed to maintain correct guidewire J-tip position throughout an US-guided infraclavicular CVC placement in the right subclavian vein.

**Methods:**

100 adult intensive care patients with a computed tomography scan of the chest were retrospectively and consecutively included in the study. The distance from the most plausible distal puncture site of the right subclavian/axillary vein to the junction of the right and left brachiocephalic veins (= vessel length) was measured using multiplanar reconstructions. In addition, measurements of the equipment provided in commonly used 15-16 cm CVC kits were performed. The minimal guidewire length was calculated for each CVC kit.

**Results:**

The guidewires were up to 90 mm too short to maintain correct J-tip position throughout the CVC insertion procedure in seven of nine commercial CVC kits. Four of these are shown in Table 1.

**Conclusions:**

When US guidance is used to confirm a correct guidewire J-tip position, retraction of the guidewire prior to the CVC insertion must be avoided to ensure correct CVC-tip positioning. This study shows that most of the commonly used 15-16 cm CVC kits contain guidewires that are too short for CVC placement in the right subclavian vein.

**Table 1 (abstract P194).** Minimal guidewire length = 105 mm (95th percentile of the vessel length) + 30 mm (to ensure that the guidewire J-tip passes the junction of the right and left brachiocephalic veins and the azygos arch into the lower part of the superior vena cava) + steel cannula length (the maximum length that can be inserted into the patient) + CVC full length + 10 mm (the part of the guidewire that must extend from the CVC before CVC-insertion)

Manufacturer	Model	Actual guide-wire length (mm)b	Calculated minimal guidewire length (mm)c	Discrepancy (mm)
Arrow, 2-lumen	Blue FlexTip 16 cm	590	506	84
Bactiguard, 3-lumen	BIP CVC 16 cm	440	513	-73
B Braun, 3-lumen	Certofix 15 cm	495	528	-33
Meritmedical, 3-lumen	Careflow 15 cm	445	535	-90

## P195 The reliability of lung B-lines to assess fluid status in patients with long period of supine position

### K Mnif^1^, O Doukali^2^, E Unluer^3^, R Ammar^2^, C Ben hamida^2^, M Bouaziz^2^

#### ^1^University Hospital Habib Bouguiba Sfax, Intensive Care Unit, Sfax, Tunisia; ^2^university hospital habib bouguiba sfax, intensive care unit, sfax, Tunisia; ^3^S.B.U Bozyaka Egitim ve Arastirma hastanesi, emergency department, izmir, Turkey

**Introduction:**

In critically ill patients, due to long period of supine position, lung water accumulation in posterior areas,outside of lung or heart diseases, can be induced and B-lines can be observed. The aim of our study is to find, by transthoracic ultrasound, the correlation between B_lines and fluid status in patients with long period of supine position.

**Methods:**

This prospective study included 924 assessments performed in 154 patients with length of stay of 10 days or more . Each patient’s fluid status was evaluated in admission and each 48 hours,using B-lines number assessment by lung ultrasound,measurement of inferior vena cava (IVC) diameters, and central venous pressure (CVP) analysis .Patients were stratified into three subgroups according to their B-lines number : mild ≤14, moderate (14–30) and severe (>30).

**Results:**

There were 530 separate assessments with mild,294 with moderate and 100 with severe B-lines occurrence. There was a significant positive correlation between the log B-lines number and both IVC diameter and CVP values.The correlation between log B-lines number and IVC diameters decreases but not significantly during hopitalization ( r in admission= 0.55, p=0.001 ; r in day 10 =0.43,p=0.035). Correlations between the log B-lines number and CVP during hospitalization yielded similar results (r in admission= 0.75, p=0.001 ; r in day10 = 0.63, p=0.0026).

**Conclusions:**

B-lines number assessment keeps a significant reliability to reflect hydration status in patients with long period of supine position.

## P196 Oblique approach for ultrasound-guided cannulation of femoral vein overlapped by artery

### A Kothekar, J George, A Kulkarni, V Patil

#### Tata memorial center, Homi Bhabha National Institute, Mumbai, India

**Introduction:**

Ultrasound-guided cannulation is usually done using either longitudinal or transverse approach. The oblique approach utilizes advantages of both these approaches allowing visualization of the entire course of needle including tip and lateral discrimination of artery from vein [1]. The reported incidence of the complete overlap of femoral vein by the femoral artery is 8-10 percent [2,3]. We describe the use of the oblique approach for successful cannulation of such a femoral vein which is not possible by usual approaches (Figure 1).

**Methods:**

Case 1: A 36-year male, a diagnosed case of carcinoma right buccal mucosa was operated for wide local excision, right-sided neck dissection, and reconstruction. In immediate perioperative period, he sustained cardiac arrest due to exsanguinating bleeding from maxillary artery and pterygoid plexus needing external carotid artery ligation. Later in the course, hemodialysis was planed for acute kidney injury. Case 2: A 25-year male was under evaluation for multiple cervical lymphadenopathies and superior vena cava obstruction (SVCO). He presented to ICU with tumour lysis syndrome and life-threatening hyperkalemia requiring urgent hemodialysis.

**Results:**

In both the patients, neck vein cannulation was risky and screening ultrasound revealed a complete overlap of the femoral vein by femoral artery precluding its cannulation by traditional longitudinal or transverse approach. However, due to unavailability of safer alternatives, femoral vein cannulation was done under ultrasound guidance using oblique approach technique by the operator experienced in the oblique approach

**Conclusions:**

Oblique approach allowed successful cannulation of the femoral vein completely overlapped by the femoral artery as it offers both the visualization of the entire course of the needle and lateral discrimination of artery from the vein.

(Written informed consent was obtained)

**References:**

1. Phelan et al. J Emerg Med 37:403–8, 2009

2. Hughes et al. Anaesthesia 55:1198-202, 2000

3. Warkentine et al. Acad Emerg Med 15:426–30, 2008

**Fig. 1 (abstract P196).**
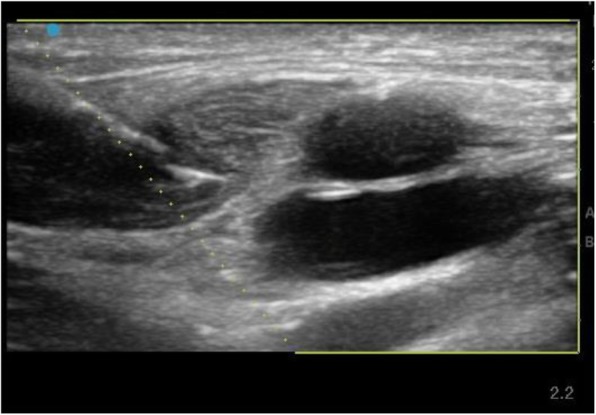
Ultrasound image showing the needle path and vessels in the oblique approach

## P197 P wave indices in critically unwell patients with new-onset atrial fibrillation (NOAF)

### JT Lay^1^, B Johnston^2^, K Williams^3^, I Welters^2^

#### ^1^University of Liverpool, School of Medicine, Liverpool, United Kingdom; ^2^Royal Liverpool Hospital, Liverpool, United Kingdom, ^3^University of Liverpool, Institute of Ageing and Chronic Disease, Liverpool, United Kingdom

**Introduction:**

Previous studies have demonstrated the potential of P wave characteristics to predict atrial fibrillation (AF). However, data on p-wave measurements in critically unwell patients are lacking. This study aimed to determine whether P wave indices in our sample diverged from previously established reference values in healthy subjects.

**Methods:**

Digital ECG recordings of 49 critically ill patients admitted to the Royal Liverpool University Hospital were collected from the Clinical Information System. 19 patients were excluded due to absent or inadequate data. ECGs of 30 patients were converted to images and analysed with a new technique in ImageJ to allow measurement of P wave indices. P wave indices included P wave axis, PR interval, mean, maximum and minimum durations, dispersion, and P wave terminal force in V1 (PTFV1). Results were compared to reference values described in previous studies [1 - 3].

**Results:**

Maximum P wave duration (137.4ms ± 19.6) and dispersion (79.5ms ± 16.5) were increased compared to reported values in the literature (103ms and 34ms, respectively) [1]. Mean PR interval (155.4ms ± 26.4) was also decreased [1]. Mean P wave duration (100.6ms ± 17.3) was greatly lower than values recorded in previous studies (66 (59, 72), 123 ± 12, 111.4 ± 14.7) [1,2,3]. Measurements in 6 patients demonstrated abnormal PTFV1 values of ≥4000μVms. Abnormal P wave axis was found in 5 patients. 1 patient displayed both abnormal PTFV1 and P wave axis.

**Conclusions:**

Our preliminary results demonstrate that mean maximum P wave duration, dispersion, and PR interval were altered in patients with NOAF compared to healthy subjects. 37% of patients had abnormalities in PTFV1 or P wave axis. Further research is warranted to investigate differences of p-wave characteristics in critically ill patients who do and those who do not develop NOAF.

**References:**

1. Magnani JW et al. Ann Noninv Electrocard 15:344-52, 2010

2. Havmoller R et al. BMC Cardiovasc Dis 7:22, 2007

3. Militaru C et al. Ann Noninv Electrocard 16: 351–6, 2011

## P198 Measuring peripheral arterial tone and biomarkers to identify endothelial dysfunction

### V Schechten^1^, A Larena-Avellaneda^1^, B Saugel^2^, R Schnabel^3^, L Plümer^2^, ES Debus^1^, C Zöllner^2^, E Schwedhelm^4^, G Daum^1^, MS Winkler^5^

#### ^1^University Heart Center, Clinic and Policlinic for Vascular Medicine, Hamburg, Germany; ^2^University Medical Center Hamburg-Eppendorf, Center for Anesthesiology and Intensive Care Medicine, Hamburg, Germany; ^3^University Heart Center, Clinic and Policlinic for Cardiology, Hamburg, Germany; ^4^University Medical Center Hamburg-Eppendorf, Institute of Clinical Pharmacology and Toxicology, Hamburg, Germany; ^5^University Medicine Göttingen, Department of Anesthesiology and Intensive Care Medicine, Göttingen, Germany

**Introduction:**

Endothelial cells play a pivotal role in the atherogenic process. Endothelial cell dysfunction (ED) is the main risk factor for cardiovascular diseases such as hypertension, coronary heart disease (CHD) and peripheral occlusive disease (POD). These diseases significantly increase the risk for perioperative complications. Therefore, identifying patients with ED is important and should influence our prospective perioperative strategy. However, sensitive tools to diagnose ED are still missing and do not belong to our standard of care. Aim of this study was the validation of a new non-invasive method to detect ED and a correlation with a set of established an new endothelial biomarkers.

**Methods:**

The cohort includes 20 preoperative patients without anamnestic relevant cardiovascular disease and 20 patients with known peripheral occlusive disease (POD). We used non-invasive EndoPAT® Technology from ITAMAR-Medical to measure ED by changes in vascular tone before and after occlusion of the brachial artery and calculate a reactive hyperemia index (RHI). In addition, we measured established markers and alternative biomarkers potentially indicate vascular diseases such as substrates and products from the NO-metabolism L-Arginin, asymmetric/symmetric dimethylarginine (ADMA/SDMA), von-Willebrand factor (vWF) and Sphingosine-1-phosphate (S1P).

**Results:**

RHI was able to identify patients with POD. RHI was significant lower in patients with clinical signs and symptoms of POD (P<0.05). Among other markers ADMA was significant higher in POD patients compared to controls and correlates with RHI.

**Conclusions:**

The PAD technology is a helpful non-invasive functional test to measure ED and seems able in identify patients with vascular disease. In future, a combination of anamnesis, new diagnostic tools and biomarkers may further increase our sensitivity in identifying risk-patients.

## P199 Single-lumen 5Fr and triple-lumen 6Fr peripherally inserted central catheters (PICCs) for cardiac output assessment by transpulmonary thermodilution

### S D´Arrigo^1^, C Sandroni^1^, S Cacciola^1^, AM Dell´Anna^1^, M Pittiruti^2^, MG Annetta^1^, G Chiuri^1^, M Antonelli^1^

#### ^1^Fondazione Policlinico Universitario A. Gemelli IRCCS, Department of Anesthesiology, Intensive Care and Emergency Medicine, Rome, Italy; ^2^Fondazione Policlinico Universitario A. Gemelli IRCCS, Department od Surgery, Rome, Italy

**Introduction:**

Trans-pulmonary thermodilution (TPTD) using single-lumen 4Fr and double-lumen 5Fr Peripherally Inserted Central Catheters (PICCs) significantly overestimate cardiac index and the other hemodynamic parameters if compared to Centrally Inserted Central Catheters (CICC) [1]. However, the reliability of PICCs of larger size is still unknown.

**Methods:**

Prospective method-comparison study conducted in a medical-surgical ICU of a teaching hospital. We compared TPTD measurements via single-lumen 5Fr or triple-lumen 6Fr polyurethane power injectable PICCs vs. triple-lumen 7Fr CICC using a TPTD-calibrated Pulse Contour hemodynamic monitoring system (VolumeView/EV1000^TM^ Edwards ©).

**Results:**

Out of 160 manual measurements in 15 patients, we did not found any difference in CI between single-lumen 5Fr PICC compared to CICC (3.2±1.04 vs. 3.2±1.06 L/min/m^2^, p=0.824; percentage of error 14.7%); we also found no differences GEDVI, EVLWI, SVI and CVP. CI was slightly higher when using triple-lumen 6Fr PICC than when using CICC (3.3±0.8 vs. 3.0±0.7 L/min/m^2^, p=0.107, percentage of error 19.0%), although this difference did not reach the statistical significance. We found instead a difference in GEDVI (685±133 vs. 632±102 mL/m^2^, p=0.05; percentage of error 19.5%).

**Conclusions:**

Single-lumen 5Fr PICCs are surely accurate for TPTD use, whereas triple-lumen 6Fr PICCs lead to a slight overestimation (ClinicalTrial.gov NCT03834675).

**References:**

1. D'Arrigo S et al. Crit Care Med 47:1356-1361, 2019

## P200 Pilot study: Advanced hemodynamic monitoring after acute spinal cord injury

### N Drotleff^1^, O Jansen^1^, M Aach^2^, TA Schildhauer^1^, C Waydhas^1^, U Hamsen^1^

#### ^1^BG University Hospital Bergmannsheil, Department of General and Trauma Surgery, Bochum, Germany; ^2^BG University Hospital Bergmannsheil, Department of Spinal Cord Injury, Bochum, Germany

**Introduction:**

Spinal cord injury causes vasoplegia and bradycardia, therefore severely affecting the cardiovascular system. To date, no publication describes the applicability of advanced hemodynamic monitoring in these patients.

**Methods:**

From March 2017 onwards we conducted a prospective, single center pilot study including all patients with an acute spinal cord injury. We excluded patients that had a preexisting cardiac condition as well as patients suffering from sepsis. Measurements were performed using the PiCCO system (Pulsion, Munich, Germany) at least 3 times a day.

**Results:**

Until December 2019 25 Patients (mean age 56 ± 20 years, range 18-82) with a total of 337 measurements were included. In 19 Patients the levels of C2 to C7 were affected. Three patients had a lesion of the thoracic level and 3 subjects suffered from lumbar paraplegia. We observed a mean Cardiac Index (CI) of 4,2 ± 1,2 l/min/m^2^, a mean Stroke Volume Index (SI) of 59 ± 15 ml/m^2^ and a mean Systemic Vascular Resistance Index (SVRI) of 1384 ± 443 dyn*s*cm^-5^*m^2^. There were 14 Patients requiring norepinephrine on at least one day of measurements. With vasopressors (n=46) we observed a mean CI of 4,1 ± 1,2 l/min/m^2^, a mean SI of 59 ± 16 ml/m^2^ and a mean SVRI of 1317 ± 572 dyn*s*cm^-5^*m^2^. Without catecholamines (n=292) we noted a mean CI of 4,2 ± 1,1 l/min/m^2^, a mean SI of 59 ± 15 ml/m^2^ and a mean SVRI of 1394 ± 418 dyn*s*cm^-5^*m^2^.

**Conclusions:**

Our preliminary results do show a difference of the mean SVRI in patients suffering from acute spinal cord injury when compared to the reference range of healthy individuals. Furthermore, the SVRI is reduced in both subjects under vasopressor therapy and patients that did not receive catecholamines. We assume a complex adaptation of the cardiovascular system that can compensate for the loss of vascular resistance in the absence of vasopressors without affecting the cardiac index.

## P201 Establishing a focused critical care echocardiography programme in a developing world intensive care unit

### D Hall^1^, J Harrington^1^, M Murali^2^, TY Wang^2^, N Shamambo^2^

#### ^1^Royal Infirmary of Edinburgh, Edinburgh, United Kingdom; ^2^University Teaching Hospital, Lusaka, Zambia

**Introduction:**

Achieving effective critical care in low- and middle-income countries is a global health goal [1], which includes the provision of effective point of care ultrasound [2]. We sought to establish Zambia’s first focused critical care echocardiography training programme in a 16-bedded ICU at University Teaching Hospital, Lusaka.

**Methods:**

The programme was accredited by the UK Intensive Care Society FICE programme, with teaching adapted for local disease patterns such as tuberculous pericardial effusions. Parasternal, apical and subcostal windows were used to assess ventricular dysfunction, hypovolaemia, pleural effusion, alveolar interstitial syndrome and pneumothorax. Zambian doctors working with critically ill patients received an intensive one-day course, followed by mentored scanning at the bedside. Teaching was delivered by visiting fellows from the UK who are accredited in echocardiography and experienced ultrasound educators.

**Results:**

26 Zambian doctors who work with critically ill patients (16 anaesthetists, 8 paediatricians, 2 physicians) were enrolled. Two handheld ultrasounds (GE VScan and Phillips Lumify) were donated to the programme. Feedback from participants indicated high satisfaction with the course: 100% agreed that the course improved their understanding of critical care echocardiography and that it was pitched at the right level. Participants believed the course was essential for developing skills in critical care, and that it was relevant to their clinical practice. Demand for further training is high. Challenges include maintaining sustainability, clinical governance, local accreditation, and servicing of equipment.

**Conclusions:**

Establishing a training programme in focused critical care echocardiography in a resource poor setting is both feasible and well received. Clinician motivation to gain competence at critical care echocardiography is high in an environment with limited availability of diagnostic services.

**References:**

1. Turner HC et al. BMJ Global Health 4:e001675, 2019

2. Becker BM et al. Trop Med Int Health 21:294-311, 2016

## P202 Association of thermodilution-derived cardiac index, stroke volume index and heart rate with hospital mortality in ICU patients: an observational study

### LR Rapp, TL Lahmer, MH Heilmaier, GB Batres-Baires, RS Schmid, WH Huber

#### Klinikum Rechts der Isar der technischen Universität München, Medizinische Klinik und Poliklinik II, München, Germany

**Introduction:**

An increasing number of less or non-invasive devices provide estimates of cardiac index CI and stroke volume index SVI with acceptable accuracy (low bias), but low precision (high percentage error).

Nevertheless, the usefulness of more precise measurements by indicator dilution techniques and strict normal ranges are questioned.

Therefore, we investigated the association of CI and SVI derived from transpulmonary thermodilution (TPTD) as well as heart rate HR and their distribution within and without normal ranges (3≤CI≤5L/min; 40≤SVI≤60mL/min; 60≤HR≤90/min) with hospital mortality.

**Methods:**

We analyzed data of a prospectively maintained database including 927 TPTD-measurements (PiCCO; Pulsion; Germany) of 55 ICU patients. Primary endpoint: hospital mortality of patients with mean values of CI, SVI and HR within and without normal range. Statistics: chi-square test. (SPSS 26).

**Results:**

Patients: 15 female, 40 male; APACHE II 23±6. Mortality was 61.5%, 30.3%, 55.6% for patients with mean CI below, within and above normal range. Mortality was higher for patients with mean CI outside compared to mean CI inside normal range. (59.1% vs. 30.3%, p=0.034). Mortality was 54.6%, 41,7% and 11.1% for patients with mean SVI below, within and above normal range. Mortality was not different for patients with abnormal mean SVI (41.9% vs. 41.7%, p=0.984). Mortality was 0%, 22.7%, 56.3% for patients with mean HR below, within and above normal range. Mortality was higher for patients with abnormal mean HR (54.6% vs. 22.7%, p=0.019).

**Conclusions:**

Patients with abnormal mean CI or HR suffer from increased hospital mortality. Abnormality of mean SVI was not associated with mortality. These data support accurate measurement of CI as a hemodynamic target and the normal range defined for CI. Since CI also carries the HR information, CI seems to be the more important target than SVI. Our data cannot necessarily be interpolated to less invasive and less precise measurements of CI.

## P203 An evaluative study of the novelty device with the function of auto-aspirating and pressure indicator for safety central venous catheterization

### LY Lin, WF Luo, CY Tsao

#### National Taiwan University Hospital, Taipei, Taiwan

**Introduction:**

Previous studies have shown that 0.8% of CVC attempts resulted in arterial punctures that were not recognized by blood color. To overcome the problem, our team has developed a concept of pressure detecting syringe that can indicate the artery puncture [1]. Based on previous research, different springs, the actuator of the design, have been evaluated to optimize the proposed device and reduce the risk of CVC procedure.

**Methods:**

Tested devices - The inner-spring is set between the pressure indicator and plunger (Fig. 1A). Three springs are tested.

Test condition **-** Blood samples were simulated by glucose solution with absolute viscosities of 2 and 6 mPa-s. Different blood pressures were applied to simulate the artery and vein (Fig. 1B). The response time (RT) is defined as the time required to show the indicating signal (IS) which is the movement of the piston from the position in Fig. 1B: A2-1 to A2-2.

**Results:**

The RT is strongly influenced by spring (Fig. 1B) but every design can show the IS when pressure is higher than 50 mmHg, the assumed minimum artery pressure. The RT of S1, the strongest spring design, is about 10s in the 50mmHg-pressure and high viscosity condition. During our tests we found the user can realize the IS before the position be fully changed from Fig. IB: A2-1 to A2-2. Thus, we believe the 10s RT, the worst case, is still acceptable. We also found the weak spring force may lead to difficulty to empty the syringe because the spring must to overcome the blood pressure and the friction between the piston and barrel. As a result, it was difficult for S3 to absolutely empty the syringe even if the blood pressure is only 30 mmHg. The spring will be compressed as Fig. 1B: A2-2 and fail to push the piston when pushing the plunger forwardly, which is not acceptable in clinical use.

**Conclusions:**

The results indicate the feasibility of using the device to facilitate CVC and we believe the S1 or S2 are more suitable for the future application.

**References:**

1. Lin LY et al. Eur J Anaesthesiol 36, e-Supplement 57:222, 2019

**Fig. 1 (abstract P203).**
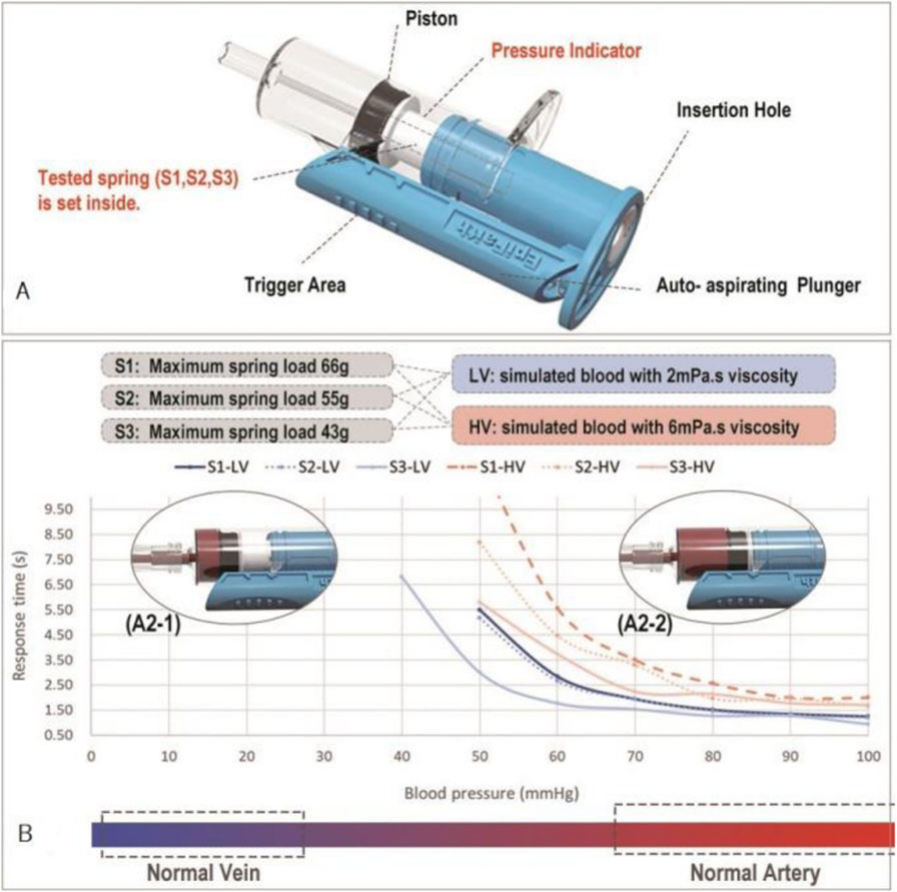
A The illustration of the proposed device; B. the relationship between response time and blood pressure

## P204 Cross-correlation features of vital signs enable robust detection of hemorrhage

### V Jeanselme^1^, A Wertz^1^, G Clermont^2^, MR Pinsky^2^, AW Dubrawski^1^

#### ^1^Carnegie Mellon University, Auton Lab, School of Computer Science, Pittsburgh, United States; ^2^University of Pittsburgh, School of Medicine, Pittsburgh, United States

**Introduction:**

Models using standard statistical features of hemodynamic vital sign waveforms (VS) enable rapid detection of covert hemorrhage at a predetermined bleed rate [1]. By featurizing interactions between VS we can train powerful hemorrhage detectors robust to unknown bleed rates.

**Methods:**

Waveforms (arterial, central venous, pulmonary arterial pressures; peripheral and mixed venous oxygen saturation; photoplethysmograph; ECG) of healthy pigs were monitored 20 min prior and during a controlled hemorrhage at 20 mL/min (N=38) and 5 mL/min (N=13). Two sets of VS features were extracted: statistical features [1] and maximal pairwise cross correlations between pairs of VS within a 5s lag over various time window sizes (30s, 60s, 180s, 300s); and normalized with pre-bleed data of each given animal. For each feature set, a tree-based (ERT) model [2] was trained and tested in a one-animal-out setting to mitigate overfitting on the 20 mL/min cohort, and another trained on the 5 mL/min and tested on the 20 mL/min cohort. We evaluated models with Activity Monitoring Operating Characteristics curves [3] that measure false alert rate as a function of time to detect bleeding.

**Results:**

Models using cross-correlations show no significant deterioration of performance when applied to detect bleeding at different rates than trained for, while standard models require 64s longer on average to detect hemorrhage at 1% false alert rate in the previously unknown setting (Figure 1).

**Conclusions:**

Correlations between VS data encode physiologic responses to hemorrhage in a way independent of the actual bleed rates. This enables training effective hemorrhage detectors using only limited experimental data, and using them in practice to detect bleeding that occurs at rates other than used in training.

Acknowledgement: Work was partially supported by DARPA FA8750-17-2-013 and NIH NIGMS 1R01GM117622

**References:**

1. Wertz et al. Critical Care Explorations 1: e0058, 2019.

2. Geurts et al. Machine Learning 63:3-42, 2006.

3. Jiang et al. AMIA Annu Symp Proc 2009:281–285, 2009

**Fig. 1 (abstract P204).**
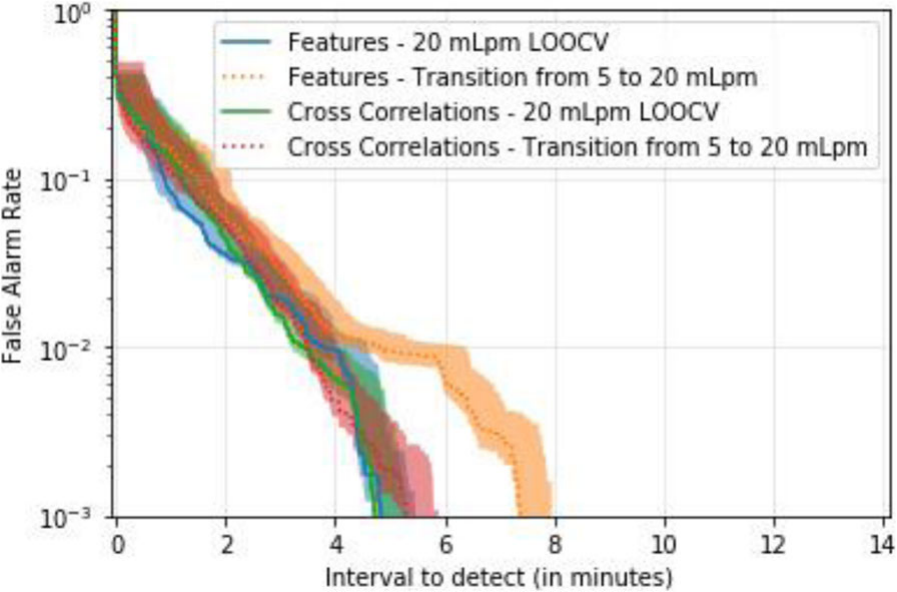
AMOC curves displaying false alarm rate as a function of time to bleeding detection ; Features - 20 mL/min LOOCV: model trained and tested on the 20mL/min cohort using standard statistical features; Features - Transition from 5 to 20 mL/min model trained on standard features on 5 mL/min cohort and tested on 20 mL/min cohort; Cross Correlations: Corresponding models trained on cross-correlation features only

## P205 Hemoguide: a new tool for decision-making support in critically ill patients

### A Minini^1^, SG Sakka^2^, FJ Belda^3^, M Kirov^4^, JL Teboul^5^, W Huber^6^, A Perel^7^, E Fernández-Mondéjar^8^, Z Molnar^9^, ML Malbrain^1^

#### ^1^University Hospital Brussels (UZB), Intensive Care Medicine, Brussels, Belgium; ^2^Universität Witten/Herdecke, Anaesthesiology and Intensive Care Medicine, Witten, Germany; ^3^Hospital Clinico Universitario, Anaesthesiology and Critical Care, Valencia, Spain; ^4^Northern State Medical University, Anaesthesiology and Intensive Care Medicine, Arkhangelsk, Russia; ^5^Hôpitaux Universitaires Paris-Sud, Médicine Intensive-Réanimation, Le Kremlin-Bicêtre, France; ^6^Klinikum rechts der Isar der Technischen Universität, Intensive Care Medicine, München, Germany; ^7^Sheba Medical Center, Anesthesiology and Critical Care, Tel Aviv, Israel; ^8^Hospital Universitario Virgen de las Nieves, Servicio de Medicina Intensiva, Granada, Spain; ^9^University of Szeged, Anaesthesiology and Intensive Therapy, Szeged, Hungary

**Introduction:**

Artificial intelligence (AI) is receiving increasing interest in modern ICU. The purpose of this project was to develop an application software for guiding hemodynamic decision support in critically ill patients.

**Methods:**

We validated a dataset of 634 data lines containing hemodynamic variables and treatment options. We selected nine hemodynamic variables as inputs. Furthermore, data were collected regarding underlying conditions: heart failure, septic shock, renal failure or respiratory failure or a combination. We applied DataStories regression on the dataset (Turnhout, Belgium, www.datastories.com). Six different interventions were analyzed as KPI: administration or removal of fluids, increasing or decreasing inotropes and increasing or decreasing vasopressors. Finally, we elaborated and challenged 63537 predictive models to generate a decision algorithm to predict each KPI. We first looked at how each hemodynamic parameter impacts the prediction of each KPI individually and performed a standard correlation analysis as well as a more involved analysis of the mutual information content between each KPI and all other hemodynamic parameters individually. Confusion matrix and variable importance was obtained for each KPI.

**Results:**

The baseline hemodynamic parameters were: GEDVi 738±218 ml/m2, EVWLi 11.2±5.5 ml/kg PBW, SVV 14.8±8 %, MBP 77.8±16.5 mmHg, HR 94.2±23.5 bpm, CI 3.3±1.2 L/min.m2. The results of the regression analysis identified the different variables of importance for each of the different interventions (Fig 1A). Based on these results the hemodynamic variables (HR, MBP, GEDVI, ELWI, CI, SVV) were used to develop the final HemoGuide prediction model (Fig 1B).

**Conclusions:**

The HemoGuide app can be used to advise physicians with respect to basic therapeutic decisions at the bedside or as an educational tool for students. With the collection of new data, the accuracy of the system may grow over time. The next step of the project is to develop a more-sophisticated suite: the ICU cockpit.

**Fig. 1 (abstract P205).**
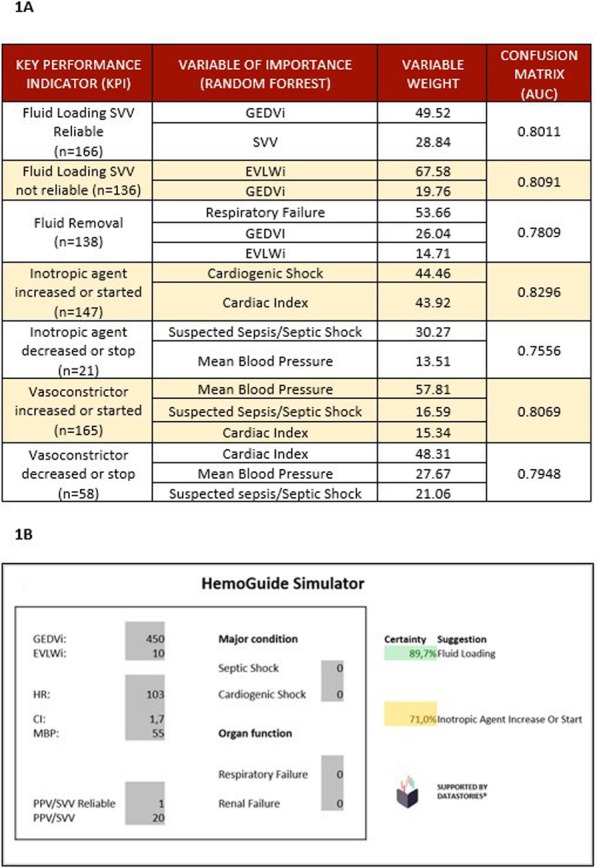
A: Key results of the regression analysis; B: HemoGuide Simulator Interface

## P206 Feedback function contributes to accurate measurement of capillary refill time

### R Kawaguchi^1^, TA Nakada^2^, M Shinozaki^2^, T Nakaguchi^2^, H Haneishi^2^, S Oda^2^

#### ^1^Chiba University, Department of Emergency and Critical Care Medicine, Chiba, Japan; ^2^Chiba University, Chiba, Japan

**Introduction:**

Capillary Refill time (CRT) is well known as an indicator of peripheral perfusion. However, it has been reported to have an intra-observer variance, partly because of manual compression and naked-eye measurement of the nailbed color change. We hypothesized that a feedback function on a CRT measurement device would lead to an accurate compression.

**Methods:**

We developed a novel portable CRT measurement device with an OLED display that feedbacks weather the strength of the nailbed compression is enough and counts the time. We settled the target strength and time as 5N and 5seconds according to the study we reported before [1]. 20 examiners measured CRT with and without the feedback function. The pressing strength and time during the measurement were evaluated.

**Results:**

There was a significant difference among the pressing strength and time between the CRT measurement using the device with and without the feedback function (strength: P<0.001; time: P<0.001). Furthermore, intra-examiner variance was significantly reduced with the feedback function (strength: P<0.001; time: P<0.001). In all measurements without the feedback function, 41% was outside the optimal strength while the measurements with the feedback function 100% achieved the targeted range. Without the feedback function, 12% could not reach the optimal time, while 100% with the feedback function did. In total, 49% of the measurements could not achieve the optimal pressing strength and time.

**Conclusions:**

The feedback function for CRT measurements, guiding examiners to an optimal pressing strength and time, fulfilled the required measurement conditions and reduced intra-examiner variance. Our novel portable device would assist an accurate CRT measurement regardless of personal work experience.

**References:**

1. Kawaguchi R et al. Crit Care 23:4, 2019

## P207 Course of conjunctival microcirculatory changes in patients with sepsis

### J Simkiene^1^, Z Pranskuniene^2^, V Pilvinis^3^, A Pranskunas^4^

#### ^1^Department of Intensive Care Medicine, Lithuanian University of Health Sciences, Intensive Care, Kaunas, Lithuania; ^2^Department of Drug Technology and Social Pharmacy, Lithuanian University of Health Sciences, Kaunas, Lithuania; ^3^Department of Intensive Care Medicine, Lithuanian University of Health Sciences, Kaunas, Lithuania; ^4^Department of Intensive Care Medicine, Lithuanian University of Health Sciences, Intensive Care, Kaunas, Lithuania

**Introduction:**

The aim of the study was to detect the difference of conjunctival microcirculation between septic patients and healthy subjects and evaluate the course of conjunctival microcirculatory changes in survivors and non-survivors over a 24 hours period of time.

**Methods:**

This single-centre prospective observational study was performed in mixed ICU in a tertiary teaching hospital. We included patients with sepsis or septic shock within the first 24 hours after ICU admission. Conjunctival imaging using IDF videomicroscope as well as systemic hemodynamic measurements were performed at three time points: at baseline, 6 hours and 24 hours later. Baseline conjunctival microcirculatory parameters were compared with healthy control.

**Results:**

A total of 48 patients were included in the final assessment and analysis. Median APACHE II and SOFA scores were 16 (12-21) and 10 (7–12) respectively. 44 (92%) were in septic shock, 48 (100%) required mechanical ventilation. 19 patients were discharged alive from the intensive care unit. We found significant reductions in all microcirculatory parameters in the conjunctiva when comparing septic and healthy subjects. We found a significant lower proportion of perfused vessels and microvascular flow index (MFI) of small vessels during all three time points in non-survivors compared with survivors. In non-survivors we observed no significant changes in conjunctival microcirculatory parameters over time. However, survivors had significantly improved MFI of small vessels at second and third time points compared to first time point.

**Conclusions:**

Microcirculatory perfusion in conjunctiva was altered in septic patients. Over 24 hours evaluation survivors in comparison with non-survivors had better microcirculatory flow with incremental improvement of microvascular flow index.

## P208 Assessment of right heart function during extracorporeal therapy by modified thermodilution

### KF Bachmann^1^, L Zwicker^1^, K Nettelbeck^1^, D Casoni^2^, PP Heinisch^3^, H Jenni^3^, M Haenggi^1^, D Berger^1^

#### ^1^Inselspital Bern, University Hospital, Bern, Switzerland, Department of Intensive Care Medicine, Bern, Switzerland; ^2^University of Bern, Experimental Surgery Facility (ESF), Department for BioMedical Research Faculty of Medicine, Bern, Switzerland; ^3^Inselspital Bern, University Hospital, Bern, Switzerland, Department of Cardiaovascular Surgery, Bern, Switzerland

**Introduction:**

Extracorporeal membrane oxygenation (ECMO) therapy is an emerging treatment modality for acute respiratory and/or cardiac failure. Cardiac output monitoring during ECMO therapy remains challenging. This study aims to validate a new method of thermodilution technique during ECMO therapy.

**Methods:**

16 healthy pigs were centrally cannulated for veno-arterial ECMO and precision flow probes were placed on the pulmonary artery main trunk for reference. 10ml boluses of iced 0.9% saline chloride solution were injected into the ECMO circuit and right atrium at different ECMO flow settings (4, 3, 2, 1 L/min). Rapid response thermistors of standard PA-catheters in the ECMO circuit and pulmonary artery recorded the temperature change. After calibration of the catheter constants for different injection volumes in the ECMO circuit, the distribution of injection volumes passing each circuit was assessed and enabled calculation of pulmonary blood flow. Analysis of the exponential decay of the signals allowed assessment of right ventricular function.

**Results:**

Calculated blood flow correlated well with true blood flow (r^2^ = 0.74, p < 0.001, Figure 1 Panel A, individual measurements). The calculated changes in blood flow tracked the true changes with 100% concordance (Panel B, average of 5 measurements). Bias was -6 [95% CI -53 – 48] ml/min with clinically acceptable limits of agreement (668 [95% CI 502 – 834]) ml/min, Panel C). Right ventricular ejection fraction (RVEF) was 17 [14 – 20] %. ECMO flow reductions increased end-diastolic (EDV) and end-systolic (ESV) volumes (Panel D) with reductions in pulmonary vascular resistance, but without changing central venous pressure (CVP) and only little changes in RVEF. EDV correlated highly with ESV (r^2^ = 0.98, p < 0.001, Panel D).

**Conclusions:**

Adapted thermodilution allows reliable measurements of cardiac output and assessment of right ventricular behaviour. During ECMO weaning, the right ventricle dilates even with stable function, possibly due to increased venous return.

**Fig. 1 (abstract P208).**
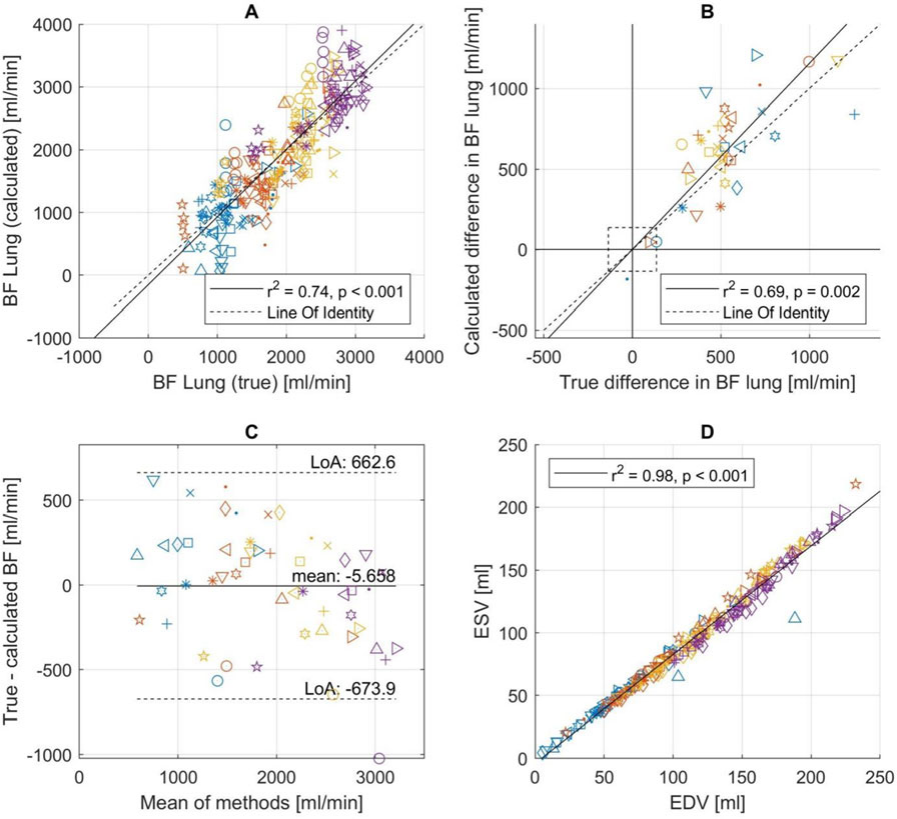
Cardiac output monitoring (Panel A, B, C) and right heart function (Panel D) during VA ECMO weaning, assessed by modified thermodilution

## P209 Quantifying systemic congestion with point-of-care ultrasound: development of the Venous Excess Ultrasound Score

### W Beaubien-Souligny^1^, P Rola^2^, K Haycock^3^, J Bouchard^4^, Y Lamarche^5^, R Spiegel^6^, A Denault^7^

#### ^1^Centre Hospitalier de l´Université de Montréal (CHUM), Nephrology, Montréal, Canada; ^2^Washington Hospital Center, Division of Intensive Care, Washington DC, United States; ^3^Washington Hospital Center, Department of Emergency Medicine, Loma Linda University School of Medicine, Washington DC, United States; ^4^Washington Hospital Center, Division of Nephrology, Washington DC, United States; ^5^Washington Hospital Center, Department of Surgery and Critical Care, Washington DC, United States; ^6^Washington Hospital Center, Departments of Critical Care and Emergency Medicine, Washington DC, United States; ^7^Washington Hospital Center, Department of Anesthesiology and Intensive Care, Washington DC, United States

**Introduction:**

Organ congestion is susceptible to be a mediator of adverse outcomes in critically ill patients. Point-Of-Care ultrasound (POCUS) is widely available and could enable clinicians to detect signs of venous congestion at the bedside. The aim of this study was to develop prototypes of congestion scores and to determine their respective ability to predict acute kidney injury (AKI) after cardiac surgery.

**Methods:**

This is a post-hoc analysis of a prospective study in 145 patients for which repeated daily measurements of hepatic, portal, intra-renal vein Doppler and inferior vena cava (IVC) ultrasound were performed before surgery and during the first 72 hours after cardiac surgery [1]. Five prototypes of venous excess ultrasound (VExUS) scores combining multiple ultrasound markers were developed (Figure 1). The association between each score and AKI was assessed using time-dependant Cox models as well as conventional performance measures of diagnostic testing.

**Results:**

A total of 706 ultrasound assessments were analyzed. We found that defining severe congestion as the presence of severe flow abnormalities in multiple Doppler patterns with a dilated IVC (>2cm), corresponding to grade 3 of the VExuS C score, showed the strongest association with the development of subsequent AKI compared with other combinations of ultrasonographic features (HR: 3.69 CI: 1.65-8.24 p=0.001). The association remained after adjustment for baseline risk and vasopressor/inotropic support (HR: 2.82 CI: 1.21-6.55 p=0.016). Furthermore, this VExUS score offered a useful positive likelihood ratio (+LR: 6.37 CI: 2.19-18.50) when detected at ICU admission, which outperformed central venous pressure measurements.

**Conclusions:**

The VExUS C score combines multiple POCUS measurements and may identify clinically significant venous congestion. Further studies should aim to validate this score in other patient populations.

**References:**

1. Beaubien-Souligny W et al. J Am Heart Assoc 7:e009961, 2018

**Fig. 1 (abstract P209).**
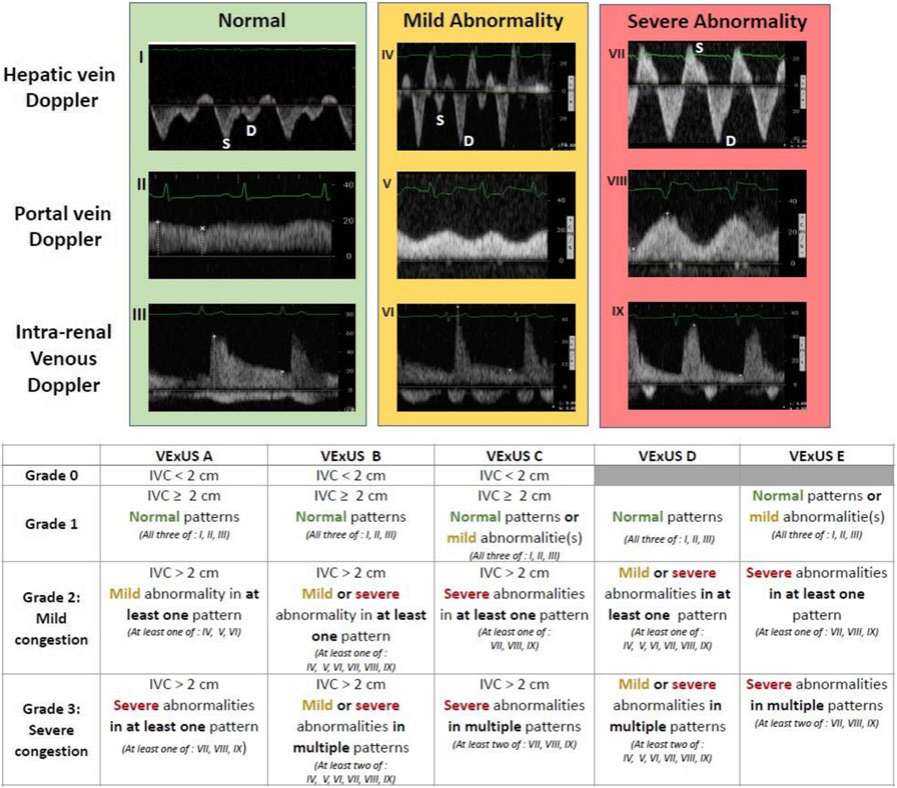
Venous Excess Ultrasound Score prototypes combining inferior vena cava (IVC) measurements to pulse-wave Doppler patterns in the hepatic vein, portal vein and intra-renal veins. Portal Doppler is considered mildly abnormal when a variation in the velocities during the cardiac cycle of 30 to <50% are observed while is considered severely abnormal when a variation of more than 50% is seen. Hepatic Doppler is considered mildly abnormal when the systolic component is lower in magnitude than the diastolic component but still toward the liver while it is considered severely abnormal when the S component is reversed (toward the heart). Intra-renal venous Doppler is considered mildly abnormal when it is discontinuous with a systolic and diastolic phase while is it considered severely abnormal when it is discontinuous with only a diastolic phase seen during the cardiac cycle. S: systole, D: diastole

## P210 Comparison of superior vena cava & inferior vena cava diameter changes by echocardiography in predicting fluid responsiveness in mechanically ventilated patients

### V Upadhyay^1^, SS Nath^1^, M Tripathi^1^, D Malviya^1^, A Jha^2^

#### ^1^Dr Ram Manohar Lohia Institute Of Medical Sciences, Department Of Anesthesia And Critical Care, Lucknow, India; ^2^Dr Ram Manohar Lohia Institute Of Medical Sciences, Department Of Cardiology, Lucknow, India

**Introduction:**

To assess and compare the efficacy of Superior Vena Cava (SVC) & Inferior Vena Cava (IVC) diameter changes in response to passive leg raise by Echocardiography in predicting fluid responsiveness in mechanically ventilated hemodynamically unstable critically ill patients.

**Methods:**

30 patients with hypovolemia or septic shock, mechanically ventilated and critically ill were prospectively enrolled over a one-year period in our ICU. Heart rate, systolic blood pressure, diastolic blood pressure, mean arterial blood pressure, respiratory variation in SVC diameter measured by transesophageal echocardiography(TEE), IVC diameter by transthoracic echocardiography(TTE) and change in cardiac index measured by maximal doppler velocity in left ventricular outflow tract were recorded. With formulas, predictive indices like Collapsibility Index of SVC (cSVC) and Distensibility Index of IVC(dIVC) were calculated and measurements were performed at baseline and 1 minute after PLR. Patients were separated into responders(R)(increase in cardiac index ≥10%) and non-responders(NR) (increase in cardiac index <10% or no increase).

**Results:**

Among 30 included patients, 24 (80%) patients were responders. cSVC was significantly more accurate than dIVC in predicting fluid responsiveness.The areas under the receiver operating characteristic curves for cSVC and dIVC regarding assessment of fluid responsiveness were 1.00 (95% confidence interval(CI):1.00 to1.00) and .66 (95% CI: 0.44 to 0.89) respectively. No significant correlation between cSVC and dIVC was found among R (r=.02, P=0.92) and NR (r=0.46, P=0.35)at baseline.The best threshold values for discriminating R from NR was 35% for cSVC with sensitivity and specificity of being 100% and 25% for dIVC with 54% sensitivity and 86.7% specificity.

**Conclusions:**

cSVC had the better sensitivity and specificity than dIVC in predicting fluid responsiveness. cSVC had a greater diagnostic accuracy than dIVC in our study.

## P211 Changes in the microvascular bed availability as an index of fluid loading

### LR Ramahi^1^, CM Maimone^2^, VG Giammatteo^2^, RD De Blasi^2^

#### ^1^Sant’Andrea University Hospital- Sapienza University of Rome, Italy, Intensive Care, Rome, Italy; ^2^Sant’Andrea University Hospital- Sapienza University of Rome, Italy, Rome, Italy

**Introduction:**

There is an increasing awareness on the consequences of fluid administration in patients leading to the development of methods that evaluate the effects of fluids loading on the cardiocirculatory system. However, most of methods used in the clinical practice investigate the effects of fluids on the cardiac function, instead of investigating those on the determinants of venous return. Besides volume of fluids, the determinants of fluid loading are the blood volume distribution and the availability of vascular bed. In this study we aimed to test non-invasively the effects of fluids administration on the venular compartment in the skeletal muscle. In addition to the mean systemic filling pressure (msfp), we calculated changes in the stressed and unstressed volumes (Vs, Vu) and the venular bed availability.

**Methods:**

We enrolled 10 critically ill patients in our Intensive Care Unit. We assessed volumes and pressures by the Near Infra-Red Spectroscopy on the forearm using graded venous occlusions in steps of 5mmHg from 50 to 0 mmHg. The msfp, Vu and Vs were measured as previously reported (Microcirculation 2014; 21:606–614). The vascular bed availability was measured by changes in the volume recruited from the occlusion maneuvers. All the measures were done at baseline and after a fluid load ranging from 250 to 500 ml. Values were expressed as median and interquartile range. Wilcoxon test was used to compare data and a p< 0.05 was considered as significant.

**Results:**

Vascular bed recruited was 33.6% (22.8–42.2) at baseline and 23.9% (19.0–33.4) after a fluid load (p=0.002). The Vs was 0.5mL (0.3 – 0.7) at baseline and 0.6 mL (0.5–0.9) after fluids (p=0.620). The Vu was 1.9mL (1.7-2.5) at baseline and 2.3mL (2.0-2.4) after fluids (p=0.193).

**Conclusions:**

Following fluid administration the only variable that decreased in all subjects was the vascular bed recruited. This was possibly related to changes in the interstitial pressure due to fluid leakage.

## P212 Prediction of post-induction hypotension associated with general anesthesia using point-of-care cardiac ultrasound: the value of two dynamic markers of fluid responsiveness

### Y Aissaoui

#### Avicenna Military Hospital - Caddi Ayyad University, Department of Anesthesiology and Intensive Care, Marrakech, Morocco

**Introduction:**

Hypotension is a common side effect of general anesthesia (GA) and is associated with organ hypoperfusion and poor perioperative outcome [1]. Post-induction hypotension (PIH) is caused by the depressant cardiovascular effect of anesthetic drugs and could be amplified by hypovolemia. The aim of this study was to assess the ability of two echocardiographic fluid responsiveness markers to predict PIH: the inferior vena cava collapsibility index (IVC-CI) and the velocity time integral change (ΔVTI) after passive leg raising.

**Methods:**

Sixty patients > 50 years of age and scheduled for elective surgery were included. IVC-CI and ΔVTI were measured before GA induction. Anesthesia protocol, fluid infusion and vasopressor administration were standardized in all patients. PIH was defined as a mean arterial pressure (MAP) <65 mmHg or a relative decline from pre-induction value of at least 30% within 12 minutes of GA induction. Receiver operating characteristic (ROC) curve analysis was used. The optimal cut-off was selected to maximize the Youden index (sensitivity + specificity − 1).

**Results:**

The measurement of IVC-CI and/or ΔVTI were unsuccessful in seven patients (11.6%). PIH occurred in 32 patients (incidence 53 %). The areas under the ROC curves (Figure 1) were 0.84 [95% CI: 0.72 – 0.96; p<0.0001] for ΔVTI and 0.67 [95% CI: 0.52 – 0.83; p=0.043] for IVC-CI. The optimal cut-off values were 18% for ΔVTI (sensitivity 75%, specificity 91%) and 25% for IVC-CI (sensitivity 75 %, specificity 45%).

**Conclusions:**

The ΔVTI after passive leg raising was able to predict PIH and performed better than IVC-CI. The use of this marker could help individualize strategies to prevent PIH (pre-induction fluid loading, vasopressors, anesthetic technique and close hemodynamic monitoring).

**References:**

1. Bijker JB et al. Anesthesiology 107:213–20, 2007

**Fig. 1 (abstract P212).**
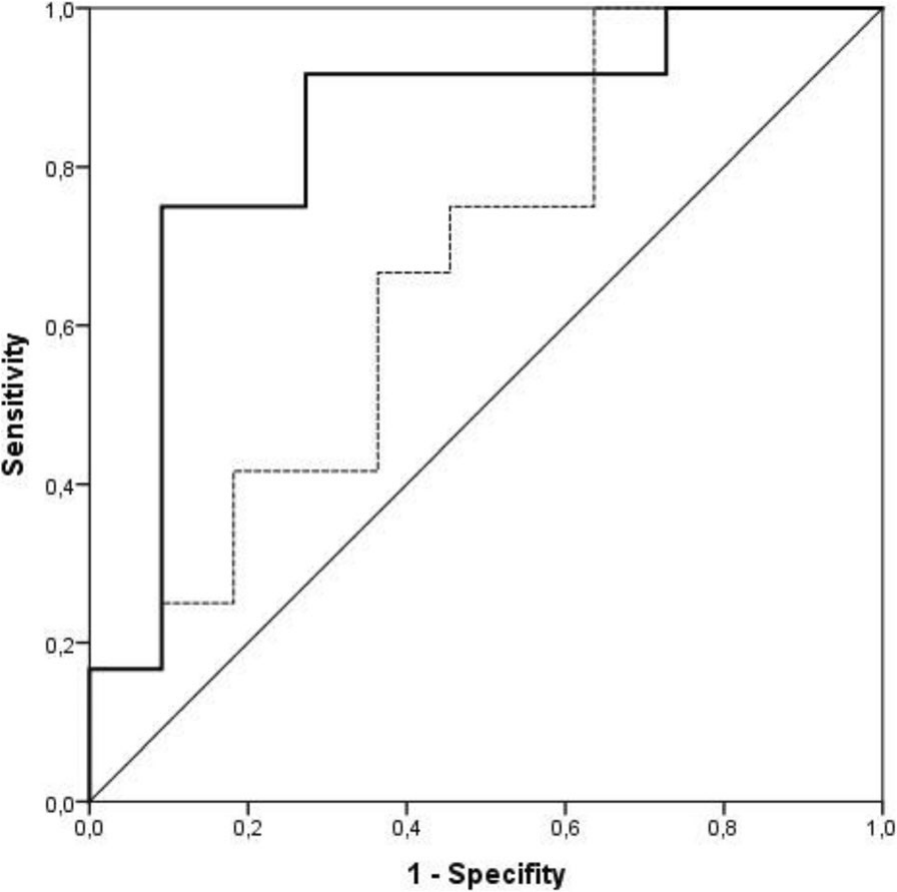
Receiver operating characteristics (ROC) curves of ΔVTI (continuous line) and IVC-CI (Dashed line) for prediction of post-induction hypotension

## P213 Pleth varisbility index (PVI) vs. pulse pressure variation (PPV) and stroke volume variation (SVV): an observational study on agreement with PVI including a derivation of comparable thresholds. The Climate-III study

### L Offman, U Mayr, T Lahmer, R Schmid, W Huber

#### Klinikum rechts der Isar, Klinik und Poliklinik für Innere Medizin II, München, Germany

**Introduction:**

Pulse pressure variation (PPV) and stroke volume variation (SVV) predict fluid responsiveness. In addition, numerous non-invasive devices provide other dynamic indices such as „Pleth varisbility index (PVI) which is derived from the pulse oximetry plethysmography wave-form. Although several studies showed prediction of fluid responsiveness by PVI, the data are conflicting, and the cut-offs of PVI range from 7 to 20%.

**Methods:**

We compared 240 measurements of PVI (Massimo; USA) with PPV and SVV simultaneously derived with the PiCCO-device (Pulsion, Germany) in 30 ICU-patients. Primary endpoint: Concordance of measurements within the categories ‘<9%’, ‘9-13%’ and ‘>13%’. Secondary endpoint: Derivation of corrected thresholds for PVI. Statistics: SPSS 26.

**Results:**

n=30; 15 female, 15 male; mechanical ventilation 240/240 (100%); vasopressors 156/240 (65%). PVI could not be derived in 1 of 240 measurements (0.4%). PVI (14.8±9.3%) was higher (p<0.001) than PPV (11.1±9.0%) and SVV (12.6±7.3%). Classifications within the categories ‘<9%’, ‘9-13%’ and ‘>13%’ agreed in 117 of 239 (49%) measurements for PVI vs. PPV (Kendall-tau r=0.435; p<0.001) and in 131 of 239 (55%) cases for PVI vs. SVV (r=0.431; p <0.001). PVI-values of 14% and 17% had the highest sum of sensitivity and specificity (ROC-analysis; Youden-Index) to predict the established PPV-cut-offs of 9% and 13%. PVI in the corrected categories ‘<14%’, ‘14-17%’ and ‘>17%’ substantially better agreed (166/239; 65%; r=0.500) with the PPV-categories ‘<9%’, ‘9-13%’ and ‘>13%’ compared to the uncorrected PVI categories ‘<9%’, ‘9-13%’ and ‘>13%’ (agreement 117 of 239 measurements (49%); p<0.001 vs. 166 of 239; chi-square-test).

**Conclusions:**

PVI was significantly higher than PPV and SVV. Only 49% and 55% of PPV- and SVV-values are classified in the same categories ‘<9%’, ‘9-13%’ and ‘>13%’ as PVI. Adjusting the PVI thresholds to 14% and 17% instead of 9 and 13% significantly improves the agreement PVI- with PPV-categories.

## P214 Preload responsiveness can be detected by mini and micro fluid challenges monitored with pulse contour analysis

### N De Vita, F Gavelli, JL Teboul, A Beurton, A Pavot, R Shi, X Monnet

#### Hôpital de Bicêtre, Service de médecine intensive-réanimation, Le Kremlin-Bicêtre, France

**Introduction:**

Preload responsiveness might be detected by the changes of cardiac index (ΔCImini) induced by a “mini-fluid challenge” (mini-FC) of 100 mL or even by the changes (ΔCImicro) in response to a “micro-fluid challenge” (micro-FC) of 50 mL. However, the smaller the fluid challenge, the larger the “grey zone” of diagnostic uncertainty. We tested whether (1) micro- and mini-FC monitored by calibrated pulse contour analysis detect preload responsiveness and (2) adding 50 mL when the result of a micro-FC is within the grey zone improves diagnostic accuracy.

**Methods:**

In 30 patients with circulatory failure, we infused 50 mL saline over 30s followed by 50 mL over 60s. We measured ΔCImicro and ΔCImini by the pulse contour analysis (PiCCO2). Preload responsiveness was defined by an increase in CI (ΔCIPLR) during a passive leg raising test ≥10%. Diagnostic uncertainty was described by calculating the grey zone after bootstrapping.

**Results:**

ΔCImicro were larger in responders than in non-responders (5.1[2.5-9.9]% *vs*. 0[0-0.7]%, respectively; p<0.0001). It was also the case for ΔCImini (9.7[7.0-12.6]% *vs*. 0.8[0-2.9]%, respectively; p<0.0001). We found a correlation between ΔCImicro and ΔCImini on the one side and ΔCIPLR on the other side (r=0.71 and r=0.82, respectively; p<0.0001 for both). For the micro-FC, the area under the receiver operating characteristic curve was 0.975±0.03 (threshold 1%), while it was 0.955±0.03 for the mini-FC (threshold 4%). For the micro-FC, the grey zone ranged from 0.82% to 3.47% and included 9 (30%) patients. For the mini-FC, it ranged from 2.8% to 6.8% and included 9 (33)% patients, among which 6 were already in the grey zone of the micro-FC.

**Conclusions:**

When evaluated by pulse contour analysis, micro- and mini-FC reliably detect preload responsiveness but with a large diagnostic uncertainty. It seems that adding 50mL more fluid to a micro-FC when its result is within the grey zone does not improve the diagnostic accuracy. The study is ongoing.

## P215 The applicability of fluid responsiveness indices in circulatory failure (AFRIC study)

### R Shi, N De Vita, F Gavelli, JL Teboul, A Pavot, S Carelli, X Monnet

#### Hôpital de Bicêtre, Hôpitaux universitaires Paris-Saclay, Le Kremlin-Bicêtre, Service de médecine intensive-réanimation, Paris, France

**Introduction:**

Passive leg raising (PLR), pulse pressure variation (PPV), and the end-expiratory occlusion (EEXPO) test are dynamic indices of fluid responsiveness, widely validated in critically ill patients. Our study aims to investigate the prevalence of conditions in which their use is limited.

**Methods:**

From January to November 2019, patients with acute circulatory failure, defined by the need of either norepinephrine support or fluid administration in the previous 24h, were included. The validity criteria of PLR, PPV and EEXPO were evaluated.

**Results:**

100 patients were enrolled. Septic shock was present in 79% of cases, cardiogenic and hypovolemic in 8% and 7% respectively, and 6% of shocks were vasoplegic (non-septic). At the time of enrolment, 94% of patients had norepinephrine infusion, 66% were mechanically ventilated (MV) and 28% had acute respiratory distress syndrome. PLR results were not reliable in 34% of cases, due to either compression stockings (14%) or intra-abdominal hypertension (IAH) (11%). In 7% of cases, no cardiac output (CO) monitoring could be obtained. Among MV patients, PPV was not interpretable in 83% of cases mainly due to spontaneous breathing activity (24%) and low tidal volume ventilation (21%). The remaining non-interpretable cases (38%) had multiple concurrent limitations. EEXPO could not be interpreted in 20% of MV patients, because either a 15-s respiratory hold could not be maintained (62%), or CO could not be monitored (38%). PLR and EEXPO were both valid in 33% of patients, while all the 3 tests were valid in 6% of patients (50% and 9% of MV patients, respectively).

**Conclusions:**

In conclusion, PLR interpretation is not reliable in 34% of shock patients. In MV patients, PPV and EEXPO test cannot be evaluated in 83% and 20% of cases, respectively.

## P216 A modified version of the bioreactance device reliably detects preload responsiveness through the end-expiratory occlusion test

### F Gavelli, A Beurton, JL Teboul, N De Vita, R Shi, A Pavot, X Monnet

#### Hôpital de Bicêtre, Service de Médecine Intensive-Réanimation, Le Kremlin-Bicêtre, France

**Introduction:**

The Starling-SV bioreactance device (Cheetah Medical) reliably detects passive leg raising (PLR)-induced changes in cardiac index (∆CI). We tested whether it can also track the small and short-time ∆CI induced by the end-expiratory occlusion (EEXPO) test, and whether shortening the time over which it averages cardiac output (24 s in the commercial version) improves the detection.

**Methods:**

In 42 mechanically ventilated patients, during a 15-sec EEXPO, we measured ∆CI (in absolute value and in percentage) through calibrated pulse contour analysis (CI_pulse_, PiCCO2 device) and Starling-SV. For the latter, we considered both CI_Starling-24_ provided by the commercial version and CI_Starling-8_ obtained by averaging the raw data over 8 s. We calculated the correlation between ∆CI_pulse_ and both ∆CI_Starling-24_ and ∆CI_Starling-8_, and the area under the receiver operating characteristic curve (AUROC) to detect preload responsiveness, defined by a PLR test.

**Results:**

When considering absolute values, the correlation coefficient *r* between ∆CI_pulse_ and ∆CI_Starling-24_ was 0.362 (p=0.02), which was lower than the one between ∆CI_pulse_ and ∆CI_Starling-8_ (*rr* comparison). When considering percentage changes, no correlation was observed between ∆CI_pulse_ and ∆CI_Starling-24_. Conversely, the correlation coefficient between ∆CI_pulse_ and ∆CI_Starling-8_ was 0.402 (p=0.01), but it was lower than the one obtained for absolute values (p=0.04 for *r* comparison). EEXPO-induced ∆CI_Starling-8_, both in absolute values and in percentage, detected preload responsiveness with AUROCs of 0.90 (sensitivity 83%, specificity 87%) and 0.89 (sensitivity 83%, specificity 79%), respectively.

**Conclusions:**

Shortening the averaging time of the bioreactance signal increases the reliability of the Starling-SV device to detect EEXPO-induced ∆CI. Moreover, the accuracy of the method is increased when absolute rather than percentage changes of CI are considered.

## P217 Detection of the effects of the end-expiratory occlusion test with the plethysmography perfusion index to detect preload responsiveness

### A Beurton^1^, F Gavelli^2^, JL Teboul^2^, N De Vita^2^, X Monnet^2^

#### ^1^Hôpital de Bicêtre, Service de réanimation médicale, Inserm UMR S_999, Université Paris-Sud, Assistance Publique Hôpitaux de Paris, Service de réanimation médicale, Inserm UMR S_999, Université Paris-Sud,, Le Kremlin Bicêtre, France; ^2^Hôpital de Bicêtre, Service de réanimation médicale, Inserm UMR S_999, Université Paris-Sud, Assistance Publique Hôpitaux de Paris, Le Kremlin Bicêtre, France

**Introduction:**

The end-expiratory occlusion (EEXPO) and end-inspiratory occlusion (EIXPO) tests consist in interrupting mechanical ventilation for 15-sec and observing its effects on cardiac output with direct measurements of CI. The perfusion index (PI) is the ratio between the pulsatile and the non-pulsatile portions of the plethysmography signal. It is in part determined by stroke volume and can detect a positive passive leg raising (PLR) test. We hypothesized that the changes in PI could detect a positive EEXPO test and thus preload responsiveness in a totally non-invasive way.

**Methods:**

In critically ill patients, we measured PI (Radical 7, Masimo) and CI (PiCCO2, Pulsion Medical Systems) before and during a PLR test, a 15-sec EEXPO and EIXPO tests and, if decided, before and after volume expansion (VE) (500-mL saline).

**Results:**

We included 31 patients. In 19 patients with a positive PLR test (increase in CI ≥ 10%), CI and PI increased during PLR by 17±7% and 49±23%, respectively, and during EEXPO test by 6±2% and 11±8%, respectively. During EIXPO, CI and PI decreased significantly by 12±4% and 11±9% respectively. In the 12 patients with a negative PLR test, PI did not significantly change during PLR, EEXPO and EIXPO tests. Only four patients received VE, causing an increase of CI and PI by 15±3% and 32±47% respectively. The correlation between the PI and CI PLR-induced changes was significant (r = 0.84, p<0.001). During the EEXPO test, if PI increased by >5%, a positive response of CI (≥5%) to the EEXPO test was diagnosed with a sensitivity of 87 (60-98)% and a specificity of 94 (70-100)% (AUROC curve: 0.92 (0.77-0.99), p<0.0001). During the combination of EEXPO and EIXPO tests, if PI increased by >9%, a positive response of CI to the EEXPO test was diagnosed with the same sensitivity and specificity.

**Conclusions:**

An increase in PI >5% during an EEXPO test accurately detects a positive response of CI to the EEXPO test.

## P218 Does the infusion speed impact the hemodyamic effect of a fluid bolus in septic shock patients? A pharmacodynamic study

### AP Pavot, F Gavelli, JL Teboul, S RUI, D Vimpere, I Adda, L Guerin, X Monnet

#### Bicetre Hospital, Medical Intensive Care, Le Kremlin-Bicêtre, France

**Introduction:**

Fluids are among the most prescribed drug in intensive care, particularly among patient with circulatory failure. Yet, very little is known about their pharmacodynamic properties and this topic has been left largely unexplored. There is a lack of strong scientific evidence in current guidelines for fluid administration in shock. Several factors may impact the hemodynamic efficacy of fluids among which the infusion rate. The aim of this study was to study the influence of fluids administration rate on their pharmacodynamics in particular by studying mean systemic pressure (P_ms_).

**Methods:**

We conducted a prospective observational study in 17 patients with circulatory failure to compare two volume expansion strategies. When a patient required a fluid bolus, 500 mL of normal saline were administered and several hemodynamic parameters were recorded continuously: cardiac output (CO), arterial pressure (AP), mean systemic pressure (P_ms_). Infusion rate was let to the discretion of the attending physician and a “slow” and a “fast” group were determined based on the median of the infusion time. Fluids effect was measured by the area under the curve (AUC), maximal effect (E_max_) and time to maximal effect (t_max_) for each hemodynamic variable.

**Results:**

P_ms_ AUC was higher in the “fast” group compared to the “slow” group (p=0.043). We observed a shorter t_max_ and a higher E_max_ for P_ms_ in the “fast” group compared to the “slow” group (p=0.039 and 0.02 respectively). Regarding CO, t_max_ was also shorter in the “fast” group (p=0.041). AUC and E_max_ were similar between the two groups. Fluid effect dissipated within 60 minutes following the end of fluid infusion for every patient in both groups. The decreasing slope from maximal effect was comparable in the groups, for P_ms_ and CO alike.

**Conclusions:**

The effect of a 500 mL fluid bolus in septic shock patients vanished within one hour. A faster infusion rate increased maximal effect and shortened the delay to reach it. Study is ongoing.

## P219 Fluid management in the control arm of sepsis trials

### AA Anparasan, AC Gordon, MK Komorowski

#### Imperial College London, Department of Surgery and Cancer, London, United Kingdom

**Introduction:**

In the past, high-volume intravenous fluid resuscitation in severe sepsis and septic shock was common. More recently, concerns over the harmful effects of this practice have led some clinicians to adopt less liberal fluid strategies. We sought to analyse temporal trends in fluid administration in the control arms of recent adult sepsis trials and assess any correlation with patient severity and mortality.

**Methods:**

A literature search was conducted to identify relevant randomized controlled trials that reported fluid administration published post 2000. We recorded 4 outcomes: total amount of IV fluid administered in the control arms of these trials between hospital admission and hour 6 and hour 72 following trial enrolment, mortality rates at the latest reported time point and APACHE-II score at admission. We computed the Pearson correlation coefficient and linear regression between study dates and the 4 outcomes.

**Results:**

We identified 9 relevant trials [1-9], which recruited a total of 2,444 patients in their control arms, from 1997 to 2018. The temporal analysis revealed no obvious trend in the in the total volume of IV fluid given by hour 6 following trial enrolment (Correlation p=0.94) (Figure 1). However, the total volume of fluid given by hour 72 decreased significantly over the period of interest (R=-0.78, p=0.02). In parallel, we observed a decrease in mortality (R=-0.6, p=0.08) but there was no evidence of decrease in illness severity over time (p=0.84).

**Conclusions:**

We found that in published RCTs over the last two decades, the amount of intravenous fluid given to patients with sepsis in the initial 6 hours did not appear to change, however less intravenous fluid was given over the first three days. Upcoming large RCTs will test the safety and efficacy of restrictive fluid administration approaches in sepsis.

**References:**

1. Rivers E et al. N Engl J Med 345:1368-77, 2001

2. Jansen TC et al. Am J Respir Crit Care Med 182:752-6, 2010

3. The ProCESS Investigators. N Engl J Med 370:1683-1693, 2014

4. The ARISE Investigators. N Engl J Med 371:1496-1506, 2014

5. Andrews B et al. Crit Care Med 42:2315-24, 2014

6. Mouncey et al. N Eengl J Med 372:1301-1311, 2015

7. Andrews B et al. JAMA 318:1233-1240, 2017

8. MacDonald SP et al. Intensive Care Med 44:2070-2078, 2018

9. Corl KA et al. Crit Care Med 47:951-959, 2019

**Fig. 1 (abstract P219).**
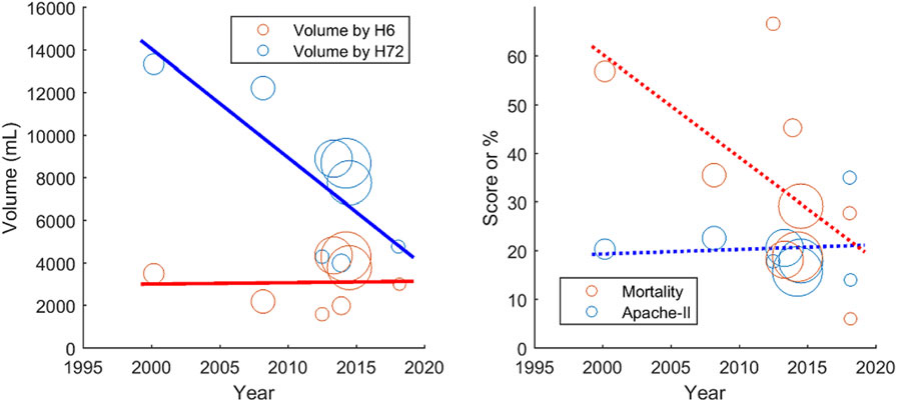
Temporal trends and regression lines of: A) volume of fluid given in control arms from hospital admission and hour 6 or hour 72 post trial enrolment B) Mortality and Apache-II. References 2, 5 and 6 report 28-day mortality; references 1, 3 and 9 report 60-day mortality; references 4, 7 and 8 report 90-day mortality. The size of the circles is proportional to the size of patient cohorts

## P220 Fluid bolus resuscitation in pediatric sepsis presenting to community hospitals

### IV Evans, J Kennedy, J Carcillo, DC Angus, CW Seymour

#### University of Pittsburgh, Clinical Research, Investigation, and Systems Modeling of Acute Illness (CRISMA) Center, Pittsburgh, United States

**Introduction:**

Clinical practice guidelines recommend prompt intravenous (IV) fluid resuscitation for pediatric sepsis, including an initial fluid bolus of 20 mL/kg [1]. However, recent evidence is conflicting as to the effectiveness, volume, and consequences of aggressive fluid resuscitation in septic children. Therefore, we sought to determine the epidemiology of early IV fluid resuscitation in an integrated health system, specifically at community hospital emergency departments (ED).

**Methods:**

We studied a retrospective cohort of pediatric patients (ages > 1 month to < 18 years) with sepsis identified in electronic health record data at 11 community EDs in southwestern Pennsylvania from 2010 to 2014. Sepsis was defined as 1) suspected infection (combination of fluid culture collection and administration of antibiotics and 2) organ dysfunction (pediatric SOFA score ≥ 1) within 24 hours of suspected infection. Fluid bolus therapy was defined as electronic documentation of administration of 0.9% normal saline IV bolus within 1 hour of the time of sepsis onset.

**Results:**

Among 1,247 patients with pediatric sepsis, 513 (41%) received IV fluid bolus therapy within 1 hour of time of sepsis onset. The volume of fluid administered ranged from 2 mL/kg to 67 mL/kg (Figure 1, Panel A), corresponding to a median volume of 20 mL/kg (IQR 17-22 mL/kg). Patients who received ≥ 20 mL/kg of fluids (n = 258, 50%) were younger (mean age 5 years, SD 5 vs. 9 years, SD 6; p<0.001), more often had blood cultures collected during evaluation (86% vs. 76%, p=0.003), and were more often transferred to another facility (48% vs. 33%, p<0.001) when compared to patients who received < 20 mL/kg of fluids (n = 255, 50%). Mean fluid bolus volume within 1 hour of time of sepsis onset by hospital ranged from 12 mL/kg to 24 mL/kg (Figure 1, Panel B).

**Conclusions:**

In a cohort of community emergency departments, 41% of septic children received intravenous fluid boluses within one hour, and of those, only one half received volumes concordant with guidelines.

**Fig. 1 (abstract P220).**
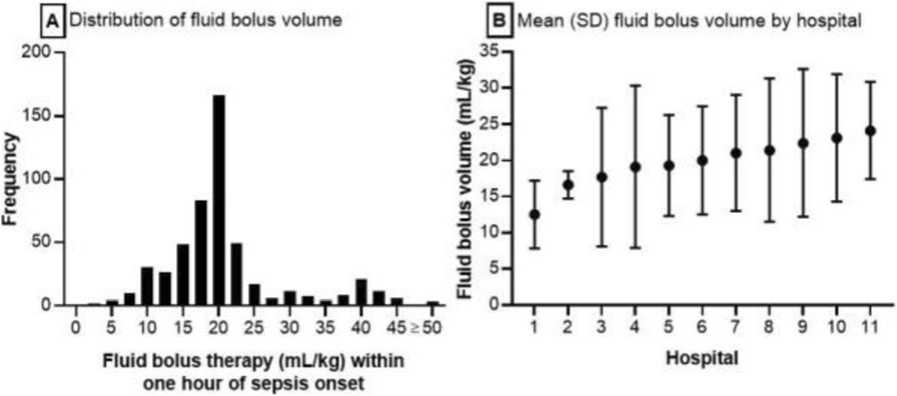
Overall distribution of fluid bolus volume and mean fluid bolus volume by hospital. A) Frequency of fluid bolus therapy volumes within one hour of sepsis onset. B) Mean fluid bolus therapy volume (SD) within one hour of sepsis onset by hospital

## P221 Fluid balance in standard of care treatment in patients with sepsis/septic shock

### I Douglas^1^, P Alapat^2^, K Corl^3^, L Forni^4^, M Exline^5^, A Holder^6^, A Khan^7^, J Sahatjian^8^, W Self^9^, D Hasell^10^

#### ^1^Denver Health Medical Center, Denver, United States; ^2^Ben Taub Hospital, Houston, United States; ^3^Rhode Island Hospital, Providence, United States; ^4^Royal Surrey Hospital, Guilford, United States; ^5^Ohio State University, Ohio, United States; ^6^Emory University, Atlanta, United States; ^7^Oregon Health and Sciences University, Oregon, United States; ^8^Cheetah Medical, Newton Center, United States; ^9^Vanderbilt University, Nashville, United States; ^10^Massachusetts General Hospital, Boston, United States

**Introduction:**

Septic shock patients are often at risk for volume overload. We explore a control patient population receiving standard of care (SOC) resuscitation to evaluate fluid balance in patients with sepsis or septic shock.

**Methods:**

FRESH is a prospective randomized controlled study, evaluating the incidence of fluid responsiveness (FR) and patient centered outcomes in critically ill septic patients (NCT02837731). Patients initially presented to the ER with hypotension and symptoms of septic shock and were admitted to the ICU. Patients randomized to treatment group had a dynamic assessment of fluid responsiveness (Starling SV, Cheetah Medical) to guide their resuscitation. Control patients received resuscitation per Institution standard of care. Patients were broken into sextets based on fluid balance.

**Results:**

48 patients received SOC treatment across 11 institutions globally. 38% were female, and the average age was 62 years. Patients exhibited 2.8 SIRS criteria with a 2.0 average qsofa score. Patients received a mean of 6.6 ± 3.4L of fluid from hospital arrival until ICU discharge or 72 hours, whichever occurred first. Patients exhibited an average fluid balance of 4.2 ± 3.4 L of fluid at ICU discharge/ 72 hours. Fluid balance ranged from 14.9L to -1.5L (Figure 1).

**Conclusions:**

A wide range of fluid balance exists in septic shock patients cared for in ICU.

**Fig. 1 (abstract P221).**
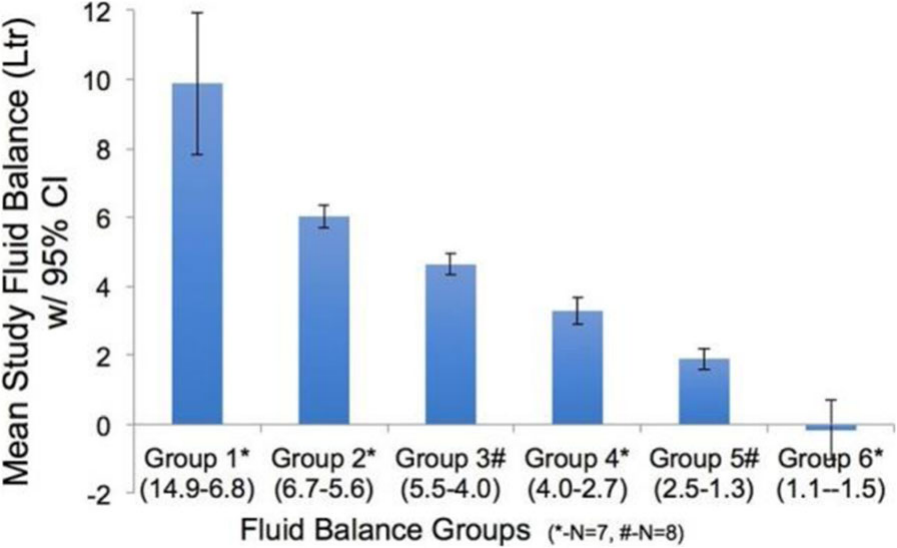
Subjects by fluid balance sextiles with range

## P222 Trends of serum albumin in septic and non-septic critically ill patients

### A Waite^1^, T Steele^2^, M Mogk^3^, I Welters^2^

#### ^1^Royal Liverpool University Hospital, Intensive Care, Liverpool, United Kingdom; ^2^Royal Liverpool University Hospital, Liverpool, United Kingdom; ^3^Moredata GmbH, Moredata GmbH, Giessen, Germany

**Introduction:**

The link between hypoalbuminaemia and poor outcomes in critical care is well established [1]. Limited data are available on serum albumin trends during critical illness [2]. In this study we assessed trends in serum albumin for up to 7 days in both septic and non-septic critically ill patients.

**Methods:**

We retrospectively examined the records of 1107 adult patients admitted to critical care at the Royal Liverpool University Hospital between 2008 and 2014. We then excluded patients who did not have albumin data available for the first 7 days, leaving us with 758 patients. 506 patients (66.8%) had sepsis, and of these patients 116 had died by day 28. Of the 252 non-septic patients (33.2%), 40 patients had died by day 28. Albumin levels were collected for 7 days from admission to critical care, in addition to other demographic and biochemical data. Statistical analysis was performed using repeated measures analysis.

**Results:**

Septic patients had lower serum albumin than non-septic patients throughout the 7 day period (p<0.001). We observed a decrease in albumin by day 2 in all groups, with levels increasing over the subsequent days. There was no difference in daily serum albumin between non-septic patients who survived or died.

**Conclusions:**

This is the first study, to our knowledge, to compare albumin trends in septic and non-septic critically ill patients over 7 days. Further research is needed to elucidate the optimal recipients and timing of albumin therapy.

**References:**

1. Vincent JL et al. Crit Care 18:231, 2014

2. Kendall H et al. Biol Res Nurs 21:237-244, 2019

## P223 Albumin recruits the microcirculation of burn patients with shock

### O Dilken^1^, A Dijkstra^2^, G Guven^1^, C Ince^1^, N Trommel^3^, M Van Baar^3^, K Van der Vlies^3^

#### ^1^Erasmus MC, Department of Intensive Care, Rotterdam, Netherlands; ^2^Maasstad Ziekenhuis, Intensive Care Burn Unit, Rotterdam, Netherlands; ^3^Maasstad Ziekenhuis, Department of Burn Unit, Rotterdam, Netherlands

**Introduction:**

Burn injury is characterized by marked inflammation, capillary leakage, and profound hemodynamic alterations. Early albumin resuscitation is avoided fearing a paradoxical fluid escape into the interstitium. On the other hand, administration of crystalloids in massive amounts causes tissue edema and fluid extravasation, which deteriorates tissue perfusion by increasing oxygen diffusion distance. Albumin administration could reduce the amount required to maintain hemodynamic stability in this population. We investigated whether albumin improves tissue perfusion and microcirculation by reducing tissue edema.

**Methods:**

This is an observational study conducted in the Burn Unit of Maasstad Hospital, Rotterdam. Patients with burns higher than 15% of Total Body Surface Area (TBSA) were included in the study. Sublingual microcirculation was measured at admission (T0), 4(T4), and 12(T12) hours after burn injury. Total Vessel Density (TVD) and Functional Capillary Density (FCD) were analyzed. Fluid Management was calculated according to the modified Parkland formula. Albumin (20%) infusion was started 12 hours after the burn insult.

**Results:**

A total of nine patients were recruited between January and December 2019. Patients were included in the study after 5.7±2.3 hours of the insult with a mean TBSA of 36±22%. The amount of crystalloid infusion was 2718±3348 ml and 8501±5230 ml at T0 and T12,respectively. Within the first 12h (T12) 502±386 ml albumin was given. TVD decreased from 23.6±2.2 at T0 to 20±1.3 at T4 (p<0.05) (Figure 1). It increased to 22.7±3.2 at T12 (ns vs T0). FCD decreased from 21.2±2.3 at T0 to 18.4±2.1 at T4(p<0.05). It increased to 21.5±3.2 at T12 (ns vs T0). Focus depth increased (117±32 to 143±28) until albumin administration (p=0.23). It decreased to 95±28 μm at T12(p<0.05). Hematocrit decreased after albumin administration (45 to 34%)(p<0.05)

**Conclusions:**

Resuscitation with crystalloids impaired tissue perfusion. Microcirculation was improved after albumin therapy.

Acknowledgement:

OD was funded by TUBITAK for this study.

**Fig. 1 (abstract P223).**
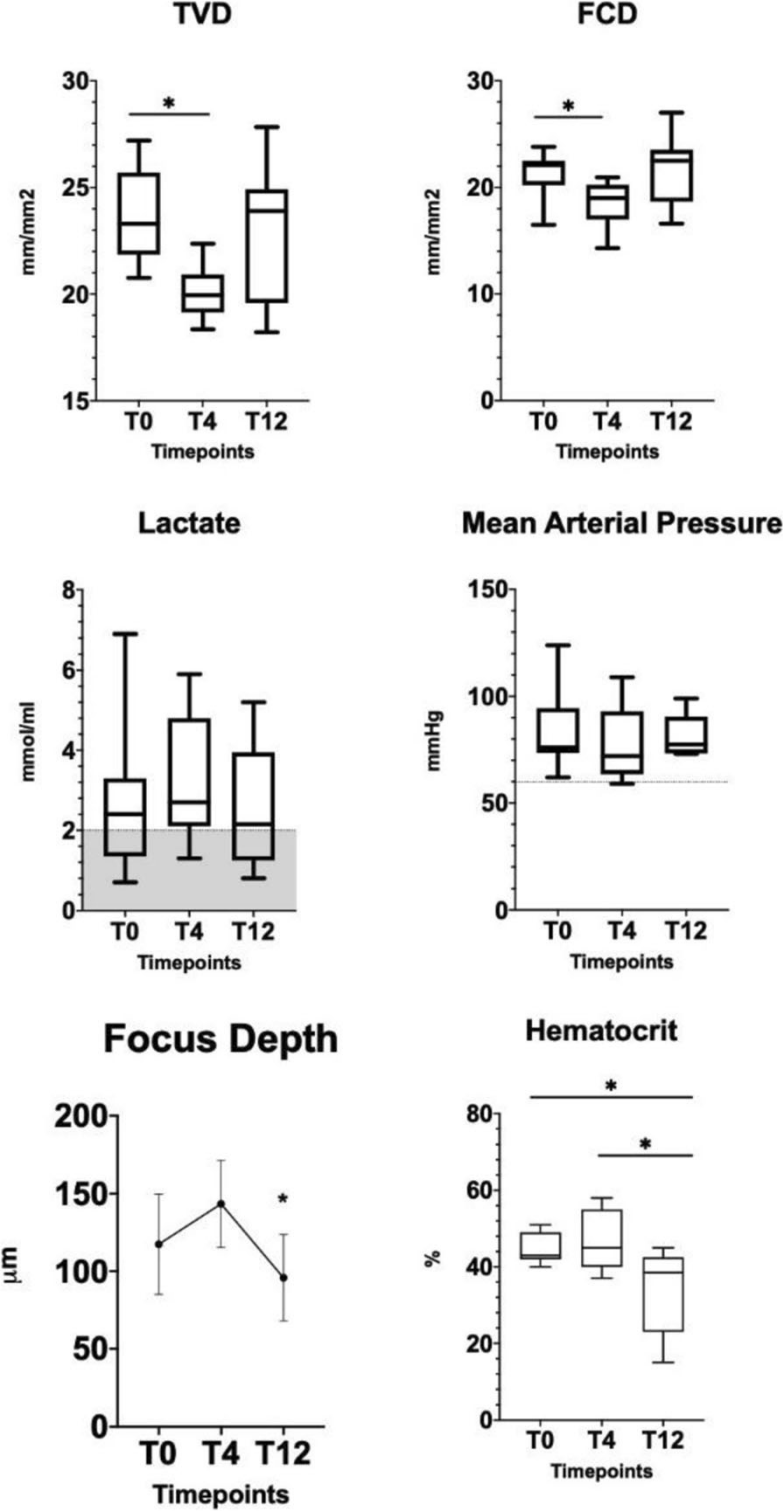
Macro- and microhemodynamic parameters

## P224 Early albumin infusion leads to a shorter hospital stay in cirrhotic patients hospitalized with spontaneous bacterial peritonitis: real-world evidence in the United States

### WR Kim^1^, K Raghunathan^2^, G Martin^3^, EA Davis^4^, N Sindhwani^4^, S Telang^5^, K Lodaya^5^

#### ^1^Stanford University School of Medicine, Stanford, United States; ^2^Duke University, Durham, United States; ^3^Emory University, Atlanta, United States; ^4^Grifols, Research Triangle Park, United States, ^5^Boston Strategic Partners, Inc., Boston, United States

**Introduction:**

Spontaneous bacterial peritonitis (SBP) accounts for ≥24% of the bacterial infections that occur in patients with cirrhosis, and SBP has a high mortality rate (20% to 50%). Albumin infusion has been shown to improve the outcome of SBP. The aim of this study is to examine the impact of albumin infusion on hospital length of stay (LOS) for cirrhotic patients with SBP.

**Methods:**

We utilized a nationwide Electronic Health Record data set (Cerner Health Facts®) to extract real-world data on adult patients (≥18 years old) with cirrhosis and SBP who received antibiotics and admitted between January 1, 2009, and April 30, 2018. International Classification of Diseases (ICD-9/10) codes were used to identify cirrhosis and SBP. We used laboratory data for calculation of the Model for End-stage Liver Disease Sodium (MELD-Na) score and vital signs data for calculation of the quick Sepsis Related Organ Failure Assessment (qSOFA) score at baseline for each encounter. A generalized linear model was used to assess the relationship between albumin infusion and hospital LOS.

**Results:**

There were 2,131 encounters that identified patients with SBP and cirrhosis, of which 1,661 survived hospitalization. Albumin was infused within 24 hours of admission ('early albumin') in 43% (n=718), after 24 hours in 31% ('late albumin', n=517), and not administered in 26% ('no albumin', n=426). MELD-Na was higher at presentation in early albumin cases versus late- or no-albumin cases (mean 24.0 and 19.5). Unadjusted LOS was lower in patients receiving early albumin (8.7 days versus 10.4 days). Risk-adjusted analysis demonstrated that early albumin led to a 17.5% reduction in LOS (95% CI 12.6%-22.2%, p = <0.0001).

**Conclusions:**

In these real-world data, albumin infusion within 24 hours of admission in patients with cirrhosis and SBP was associated with a shorter hospital stay despite more severe illness. Early albumin may not only improve clinical outcomes but may also reduce the costs of hospitalization in cirrhotic patients with SBP.

## P225 Early albumin use in patients with septic shock is associated with a shorter hospital stay: real-world evidence in the United States

### G Martin^1^, J Kempker^1^, K Raghunathan^2^, EA Davis^3^, N Sindhwani^3^, S Telang^4^, K Lodaya^4^

#### ^1^Emory University, Atlanta, United States; ^2^Duke University, Durham, United States; ^3^Grifols, Research Triangle Park, United States; ^4^Boston Strategic Partners, Inc., Boston, United States

**Introduction:**

Septic shock is among the most common critical care illnesses and incidence is rising, with mortality in excess of 35%. Septic shock predisposes patients to multiple organ failure. While albumin is effective in management of circulatory dysfunction in septic shock, its utilization in this population is understudied in the US. We evaluated the impact of albumin utilization on hospital length of stay (LOS) among septic shock patients.

**Methods:**

We used a nationwide Electronic Health Record data set (Cerner Health Facts®) to extract real-world data on adult patients (≥18 years old) with severe sepsis or septic shock, admitted between January 1, 2013, and April 30, 2018, identified by International Classification of Disease (ICD-9/10) codes, and receipt of antibiotics and vasopressors. We calculated the Charlson Comorbidity Index (CCI) and the Acute Physiology Score (APS) at baseline. A generalized linear model was used to examine the association between albumin and hospital LOS, especially accounting for the timing of albumin infusion.

**Results:**

We identified 3,156 unique visits for septic shock patients that survived to discharge. Albumin was infused within 24 hours of admission ('early albumin') in 15%, after 24 hours ('late albumin') in 20%, and not administered in 65%. Both CCI and APS were higher, at presentation, in early albumin cases than late- or no-albumin cases (mean: 7.49 and 7.17, and 51.50 and 43.23, respectively). Unadjusted LOS was slightly lower in patients receiving early albumin (11.81 days versus 11.84 days). A risk-adjusted analysis demonstrated that early albumin was associated with 4.92% shorter LOS (95% CI 0.43%-9.22%, p = 0.0322).

**Conclusions:**

Albumin infusion within 24 hours of admission was associated with a shorter length of hospital stay. Early albumin infusion may lead to better outcomes and reduced costs in patients with septic shock. Further research is being conducted to assess other potential benefits of early albumin administration in this patient population.

## P226 Minute-to-minute urine flow rate and urine flow rate variability fluctuations during septic event in critically ill patients

### E Brotfain

#### Ben Gurion University of the Negev, Department of Anesthesiology and Critical Care, Beer Sheva, Israel

**Introduction:**

Every new septic event follows by hemodynamic instability may lead sequentially to decreased organ perfusion, multiple organ failure. Acute renal failure is recognized clinical feature during sepsis (up to 40-50% in all cases). Furthermore, urine output close monitoring is a cornerstone diagnostic clinical tool in each septic critically ill patient. In present study, we analyzed the dynamic minute-to-minute changes in the urine flow rate (UFR) and also the changes in its minute-to-minute variability (UFRV) during new septic event in critically ill patients.

**Methods:**

Demographic and clinical data were extracted from the of 50 critically ill patients who were admitted to the ICU and developed new septic event (followed by fever and leukocytosis) and analyzed. A Foley catheter was inserted into the urinary bladder of each study patient. The catheter was then connected to electronic urinometer, a collecting and measurement system which employs an optical drop detector to measure urine flow. The urine flow rate variability (UFRV) is defined and calculated as the change in UFR from minute to minute.

**Results:**

UFR and UFRV both decreased significantly immediate after new septic episode until beginning fluid resuscitation (ppvalues <0.001) (Figure 1). Statistical analysis by the Pearson method demonstrated a strong direct correlation between the decrease in UFR, UFRV and the decrease in the MAP (R=0.03, p=0.003; R=0.03, p=0.004) (Figure 1), and heart rate (R=0.12,p=<0.001) since systemic pressure starts to drop. UFRV and UFR demonstrated good clinical response to fluid administration despite the fact that systemic blood pressure did not improve (Figure 1).

**Conclusions:**

We consider that dynamic changes in UFRV and UFR could potentially serve as a more sensitive signals ofclinicaldeterioration during the new septic event in critically ill patients.We also suggest that those parameters mightbeable to identify the optimal end-point of fluid resuscitative measures in septic critically ill patients.

**Fig. 1 (abstract P226).**
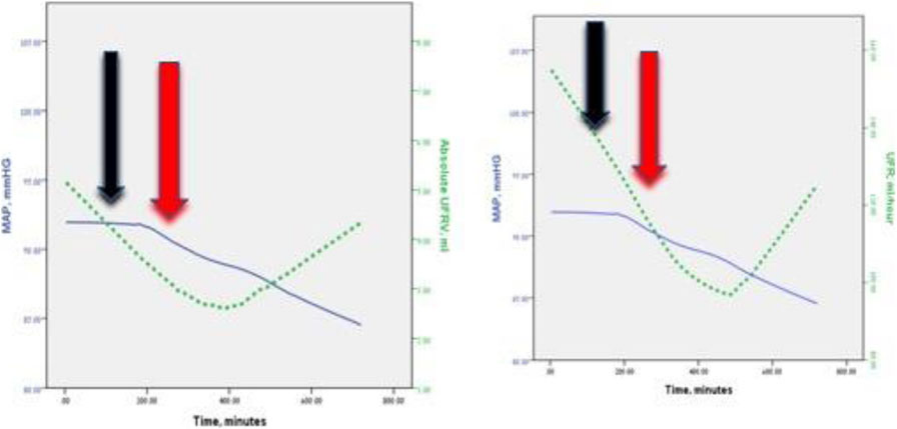
Clinical correlation between urine flow rate variability (UFRV) and UFR and mean arterial blood pressure over new septic event (black arrows) and and after initial fluid resuscitation (red arrows). Note: The UFRV and UFR decreased progressively in parallel with the falling mean arterial blood pressure and, than, rose again after the administration of fluids

## P227 Impact of urinary output on incidence and 30-day mortality of acute kidney injury during ICU admission

### J Montomoli^1^, G Guven^2^, F Termorshuizen^3^, F Raiez^3^, MP Hilty^4^, A Donati^5^, NF De Keizer^3^, C Ince^2^

#### ^1^Academic Medical Center, Department of Translational Physiology, Amsterdam, Netherlands; ^2^University Medical Center Rotterdam, Department of Intensive Care, Erasmus MC,, Rotterdam, Netherlands; ^3^Amsterdam Public Health Institute, AmsterdamUMC, Department of Medical Informatics, Amsterdam, Netherlands; ^4^University Hospital of Zurich, Institute of Intensive Care Medicine, Zurich, Switzerland; ^5^Università Politecnica delle Marche, Ancona, Italy, Anesthesia and Intensive Care, Department of Biomedical Sciences and Public Health, Ancona, Italy

**Introduction:**

Diminished urinary output (UO) is largely used as marker of acute kidney injury (AKI) in critically ill patients. We aimed to explore the role of urinary output on incidence and mortality of AKI developed during ICU admission.

**Methods:**

The study population consists of all patients admitted between 2007 and 2018 to one of the Dutch ICUs included in the NICE database with an ICU length of stay of at least 48 hours, having daily measurement of creatinine and UO. Only patients without renal replacement therapy that have a serum creatinine lower than 1.1 mg/dl (97.5 μmol/L) or a UO above 0.5 ml/kg/h on the day of the index ICU admission were considered at risk for AKI. Patients were followed during their ICU stay and classified according to the highest KDIGO criteria reached based on creatinine alone (model 1) and creatinine plus UO (model 2) using ICU admission serum creatinine as baseline. In both models, patients were classified as: no AKI, renal impairment at the first day of ICU admission, AKI stage 1, AKI stage 2, and AKI stage 3.

**Results:**

We identified 52,863 patients (60% male, mean age 63 years, median ICU-LOS 4 days). Of those, 51.2% of patients had renal impairment at the first day of ICU admission. Among the remaining patients, 44.4% in model 1 and 29.9% in model 2 were classified as having no AKI, 2.6% and 1.4% as AKI stage 1, 0.7% and 9.3% as AKI stage 2, and 1.1% and 8.2% as AKI stage 3, respectively. Survival at 30-day markedly differed according to the AKI classification model used (Figure). Similarly, adjusted HRs for 30-day mortality differed among patients with and without AKI compared to patients with renal impairment at the first day of ICU admission (Figure 1).

**Conclusions:**

Among patients admitted to the ICU 50% had renal impairment at the first day of ICU admission. Our findings suggested that UO plays an important role both on AKI incidence and mortality and should be carefully interpret in the clinical setting especially in AKI stage 2 classification.

**Fig. 1 (abstract P227).**
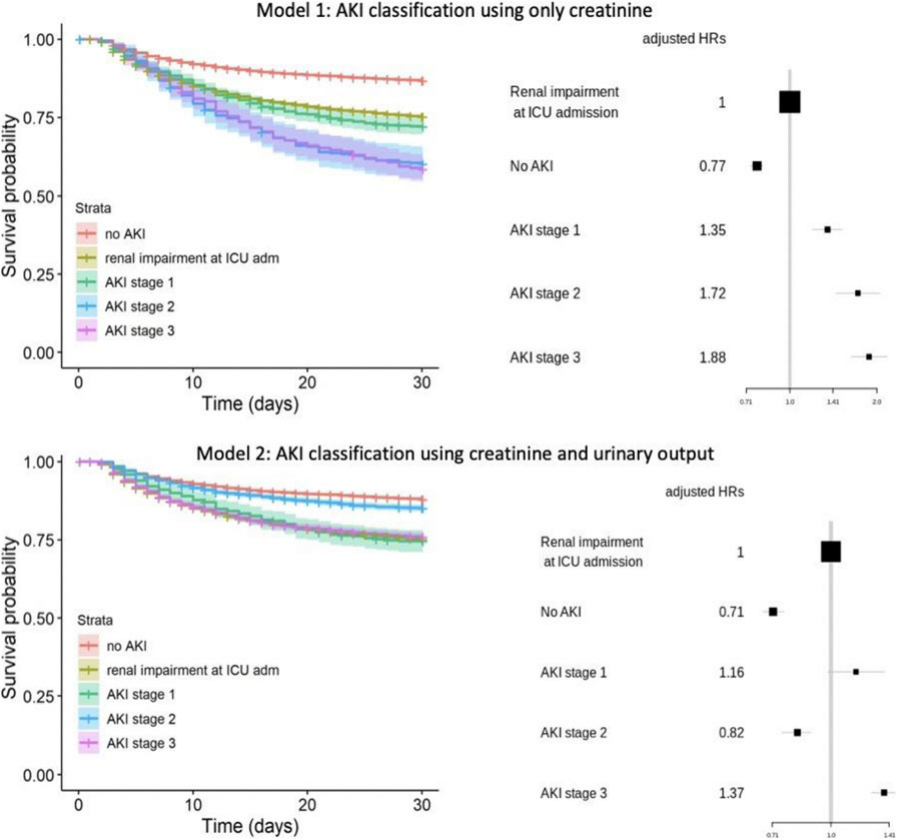
Thirty-day survival according to AKI classification model 1 and model 2. Hazard Ratios (HRs) for 30-day mortality adjusted by sex, age, type of admission, APACHE IV score, SOFA score at day of admission (excluded renal SOFA score) for patients with AKI classified with model 1 and model 2

## P228 Furosemide stress test predicts the progression of acute kidney injury

### W Farouk^1^, H Saber^1^, M Bayoumy^2^, H Khaled^1^

#### ^1^Faculty of Medicine Cairo university, Critical Care Medicine, Cairo, Egypt; ^2^Nasser institute, Critical Care Medicine, Cairo, Egypt

**Introduction:**

Acute kidney injury (AKI) mostly attributed to renal tubular damage, has a high morbidity and mortality outcome [1], so a sensitive tool to assess the degree of tubular affection is needed for early detection and management of this condition.

**Methods:**

We investigated the ability of furosemide stress test (FST) (one-time bolus dose of 1mg/kg or 1.5 mg/kg if on prior furosemide-intake) to predict progression to AKIN Stage-III in critically ill subjects with early AKI.

**Results:**

We studied 80 subjects; 40 consecutive patients in group I receiving FST and 40 consecutive patients in group II receiving standard medical management for AKI;15 patients (37.5%) and 20 patients (50%) met the primary endpoint of progression to AKIN-III in groups I and II respectively. Patients with progressive AKI had significantly lower urine output following FST in the first 6 hours (p<0.033). The area under the ROC curves for the total urine output over the first 2 hours following FST to predict progression to AKIN-III was 0.87 (p = 0.001). The ideal-cutoff for predicting AKI progression during the first 2 hours was a urine volume of less than 325 milliliters with a sensitivity of 87.1% and specificity 84.1% group receiving FST. On the other hand, statistically significant hypotension, hypo-(kalemia, phosphatemia and magnesemia) occurred in group I.

**Conclusions:**

The FST in patients with early AKI could predict liability for progression of AKI, however it should be performed under adequate monitoring.

**References:**

1. Bellomo R et al. Lancet 380: 756-766, 2012

## P229 Furosemide does not protect the kidney from ischemia reperfusion injury

### B Ergin, O Dilken, C Ince

#### Erasmus MC, Department of Intensive Care, Rotterdam, Netherlands

**Introduction:**

Ischemia-Reperfusion (IR) causes renal dysfunction and damage. IR induces renal tubular injury triggered by hypoxia and hyperoxia, mediated by oxidative stress and inflammation. Furosemide inhibits Na^+^-K^+^-2Cl^-^ cotransporter in the thick ascending limb of the renal medulla to decrease Na^+^ reabsorption, reducing oxygen consumption. We investigated if furosemide could improve renal oxygenation, function and damage by reducing O_2_ consumption and oxidative stress after IR.

**Methods:**

24 Wistar albino rats were divided into 4 groups, with 6 in each group; Sham-operated Control (C), Control + Furosemide (C+F), IR and IR+F. After anaesthesia (BL), 45 min supra-aortic occlusion was applied to IR and IR+F groups followed by 15 min (T1) and 2 hours of reperfusion (T2). Furosemide 50μg/kg/h infusion was simultaneously administered to C+F and IR+F after ischemia. Systemic hemodynamic, renal blood flow (RBF), renal vascular resistance (RVR), renal oxygen delivery (DO_2ren_), renal oxygen consumption (VO_2ren_), creatinine clearance (Ccr), sodium handling, urine output (UO), cortical (CμO2) and medullar (MμO2) microvascular oxygenation were measured.

**Results:**

RBF was reduced in IR (2.1±1) and IR+F (2.3±1) at T1 (p<0.05) but it was further reduced in IR+F (1.9±1) (p<0.05) at T2 compared to C and C+F. RVR was increased in IR (5338±2860) and IR+F (5123±2517) at T1 compared to C. RVR was normalized in IR (2198±879) but not in IR+F (4232±2636) at T2 compared to C (p<0.05). CμO_2_ and MμO_2_ did not differ between groups after IR insults (Figure 1). Tissue O_2_ was reduced at the medulla, but not at the cortex in IR+F group compared to IR. DO_2ren_ and VO_2ren_ were reduced in IR (56±17 and 26±12 ml/min) and IR+F (34±20 and 21±14) at T2 (p<0.05). PC was higher in IR+F (37.33±4.27) compared to IR 29.67±3.39 (p<0.05). VO_2_/TNa^+^ was increased in IR+F compared to IR. No change in Ccr and UO was observed.

**Conclusions:**

Furosemide after IR causes further impairment of renal perfusion, energy utilization and renal oxygenation resulting in renal damage.

**Fig. 1 (abstract P229).**
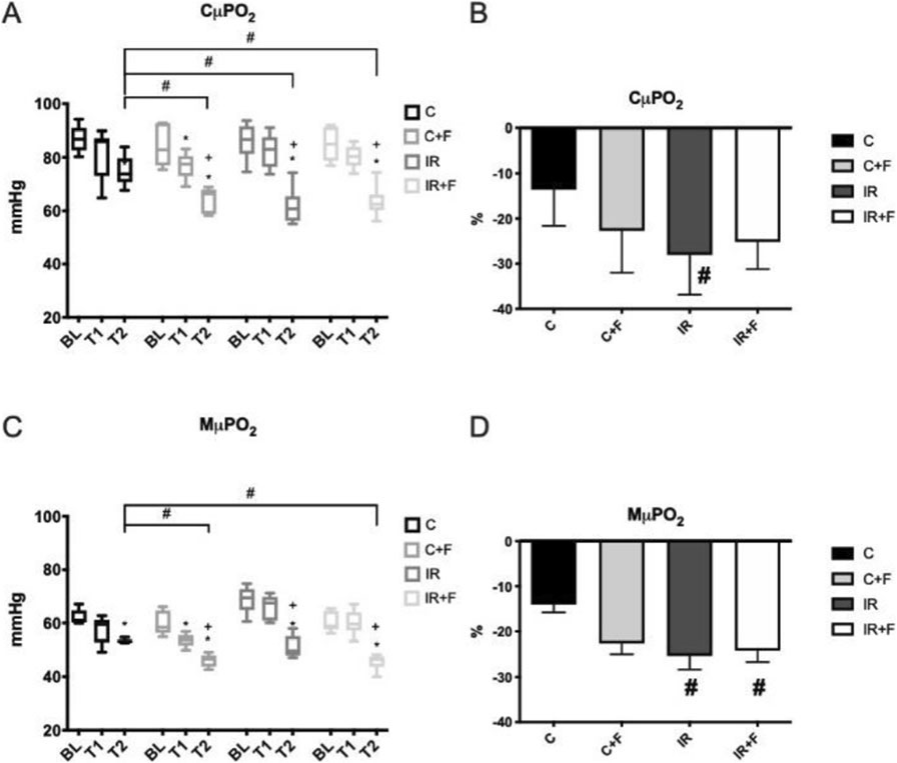
Cortical and medullar oxygenation. C: Control, C+F: C+Furosemide, IR: ischemia reperfusion, IR+F: IR + furosemide *: p<0.05 vs BL +: p<0.05 vs T1 #: p<0.05 vs C

## P230 Acute renal failure induced by hypoxemia: incidence and correlation study

### A Trifi^1^, H Fazzeni^2^, A Mehdi^2^, C Abdennebi^2^, F Daly^2^, Y Touil^2^, S Abdellatif^2^, S Ben Lakhal^2^

#### ^1^La Rabta hopital, Medical intensive care unit., Tunis, Tunisia; ^2^La Rabta hopital, Tunis, Tunisia

**Introduction:**

Acute renal failure (ARR) is a common complication in ICUs and usually caused by hypoperfusion. ARF induced by hypoxemia is a concept rarely reported in ICU. Its incidence and pathogenesis are not well understood. We aimed to study the relationship between hypoxemia and the occurrence of ARF.

**Methods:**

Retrospective cohort study including patients with hypoxemia whatever its etiology between January 2016 and August 2019. Patients with chronic renal failure were excluded. ARF was defined and ranked according to the KDIGO criteria 2012. Arterial blood gas, urea, creatinine and clearance were reordered on the first, third and seventh days of evolution.

**Results:**

50 patients were included and 2 groups were obtained: group of hypoxemic patients with ARF (ARF+, n=30): versus group of hypoxemic patients without ARF (ARF-, n= 20). The incidence of hypoxemie-induced ARF was therefore 60%. Clinical characteristics were comparable in both groups with a mean age of 47 ± 16 and a sex ratio of 1.77. The comparative study showed in ARF+ group: a lower pH (7.20 [7.8-7.33] vs.7.34 [7.27-7.41], p = 0.003) and a higher C reactive protein (CRP) in the same group (201 [129.75-325.75] vs. 117.5 [29.5-225.25], p = 0.023). The most significant correlation was showed with MDRD clearance at day 3 and P/F ratio at day 1 (Rho = 0.338, p = 0.038). Multivariate analysis found that septic shock and non invasive ventilation in hypoxemic patients were the factors related to ARF with respectively OR=11.08, 95% CI=1.56-83.84, p=0.016 and OR=6.18, 95% CI=1.16-34.07, p=0.033. Overall mortality was 68% (n=34) and ARF was an independent factor of mortality: OR=6, and 95% CI=1.35-26.64, p = 0.017.

**Conclusions:**

Hypoxemia-induced ARF is a common complication associated with excess mortality. Our study suggests that renal function is correlated with the degree of hypoxemia and that this correlation is rather distinct 48 hours from hypoxemia.

## P231 Metformin reduces mortality and development of severe AKI in diabetic patients with sepsis

### G Del Rio-Pertuz^1^, CL Manrique-Caballero^2^, P Priyanka^2^, CH Chang^2^, R Murugan^2^, BS Zuckerbraun^3^, DC Angus^4^, JA Kellum^2^, H Gomez^2^

#### ^1^Center for Critical Care Nephrology, The CRISMA Center, Department of Critical Care Medicine, University of Pittsburgh, Department of Critical Care Medicine, Pittsburgh, United States; ^2^Center for Critical Care Nephrology, The CRISMA Center, Department of Critical Care Medicine, University of Pittsburgh, Pittsburgh, United States; ^3^Department of Surgery, University of Pittsburgh, Pittsburgh, United States; ^4^Department Of Critical Care Medicine, University of Pittsburgh, Pittsburgh, United States

**Introduction:**

In preclinical models of sepsis, we have previously demonstrated that activation of AMP activated protein kinase (AMPK) using metformin, improves survival and organ function. Thus, AMPK activation is a potential therapeutic target in sepsis, and we hypothesize that exposure to metformin during sepsis is associated with decreased AKI and mortality

**Methods:**

Retrospective analysis of a 13-hospital cohort of adult ICU patients with type 2 diabetes mellitus (T2DM) who presented sepsis. We investigated if exposure to metformin during the hospitalization was associated with reduced 90-day mortality and AKI. We used 1:4 Propensity Score Matching (PSM), Propensity Score Stratification (PSS) and Propensity Score Weighting (PSW) based on the probability to be exposed to metformin using 55 covariates. For PSM an exact match for insulin, amputation, cardiovascular diseases, retinopathy, Charlson Index, eGFR, HbA1C, and APACHE III, were used. Sepsis was defined using sepsis 3 criteria, and AKI as KDIGO stage 2 or 3.

**Results:**

From 164,910 patients, we found 673 diabetic adults exposed to metformin during hospitalization and 14,174 who were not. PSM included 523 treated vs 1,680 no treated patients and resulted in an OR of 0.54 (95%CI 0.41-0.71, p<0.001) for 90-day mortality favoring metformin exposure (Figure 1). PSS: (OR: 0.49, 95%CI 0.34-0.63, p<0.001) and PSW (OR: 0.58, 95%CI 0.55-0.61, p<0.001) confirmed this protective effect. Metformin exposure also had a protective effect on stage 2-3 AKI (PSM: OR 0.82, 95%CI 0.67-0.99, p=0.04; PSS: OR 0.83, 95%CI: 0.71-0.98, p=0.02; PSW: OR 0.72, 95%CI 0.69-0.76 p<0.001).

**Conclusions:**

Metformin exposure during hospitalization is associated with decreased 90-day mortality and AKI in septic adult patients with T2DM. These findings suggest that metformin may constitute a potential therapeutic strategy in sepsis, and the potential role of AMPK activation as a protective mechanism. However, studies are needed to confirm this association and the specific mechanisms of action.

**Fig. 1 (abstract P231).**
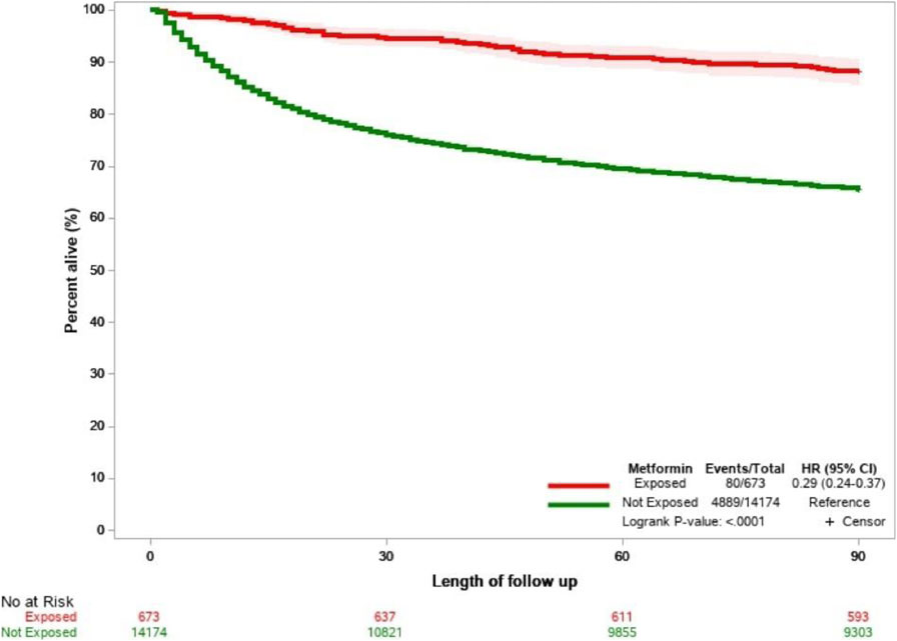
Survival estimates (90 days) by metformin exposure

## P232 Development and internal validation of a model to predict acute kidney injury recovery at hospital discharge

### CY Huang, F Güiza Grandas, M Schetz, J Gunst, M Casaer, G Van den Berghe, G Meyfroidt

#### KU Leuven, Laboratory of Intensive Care Medicine, Leuven, Belgium

**Introduction:**

Acute kidney injury (AKI) may occur up to 50% in the intensive care unit (ICU). Predicting AKI recovery may allow for risk stratification of patients, patient and family counseling, and early post-discharge renal care planning. However, predicting AKI recovery at an early stage remains a challenge.

**Methods:**

This is a retrospective study of the EPaNIC multicenter randomized controlled trial database [1], which was split into development (n=2194) and validation (n=2446) cohorts, and patients experiencing AKI stage 3 and/or renal replacement therapy (RRT) in the ICU were included [2]. AKI recovery was defined as being alive, without any stage of AKI, and without need of RRT at hospital discharge. A logistic regression model with backward feature elimination was developed. The model performance was assessed by discrimination, calibration, and net benefit analysis, and internally validated with ten-fold cross validation.

**Results:**

Only the results in the development cohort are reported. Of the 229 patients who developed AKI3, 86 patients (37.55%) recovered from AKI. The multivariable model selected age, bilirubin, heart rate, mean arterial blood pressure, surgical diagnostic group on ICU admission, mechanical hemodynamic support on ICU admission, suspected sepsis on ICU admission as AKI recovery predictors. The model had a mean area under the receiver operating characteristic curve (AUROC) of 0.75 (Standard deviation (SD) 0.01), mean calibration slope of 1.02 (SD 0.04), and mean calibration-in-the-large of <0.01 (SD 0.01) (Figure 1). At the classification threshold that maximized sensitivity and specificity, mean net benefit with respect to treat-none was 0.16 (SD 0.01) and mean net benefit with respect to treat-all was 0.11 (SD 0.01).

**Conclusions:**

By using the routinely collected clinical data, the developed prediction model can fairly identify patients with a higher chance of AKI recovery at hospital discharge.

**References:**

1. Casaer MP et al. N Engl J Med 365:506-17, 2011

2. Kellum JA et al. Kidney Int Suppl 2:1–138, 2012

**Fig. 1 (abstract P232).**
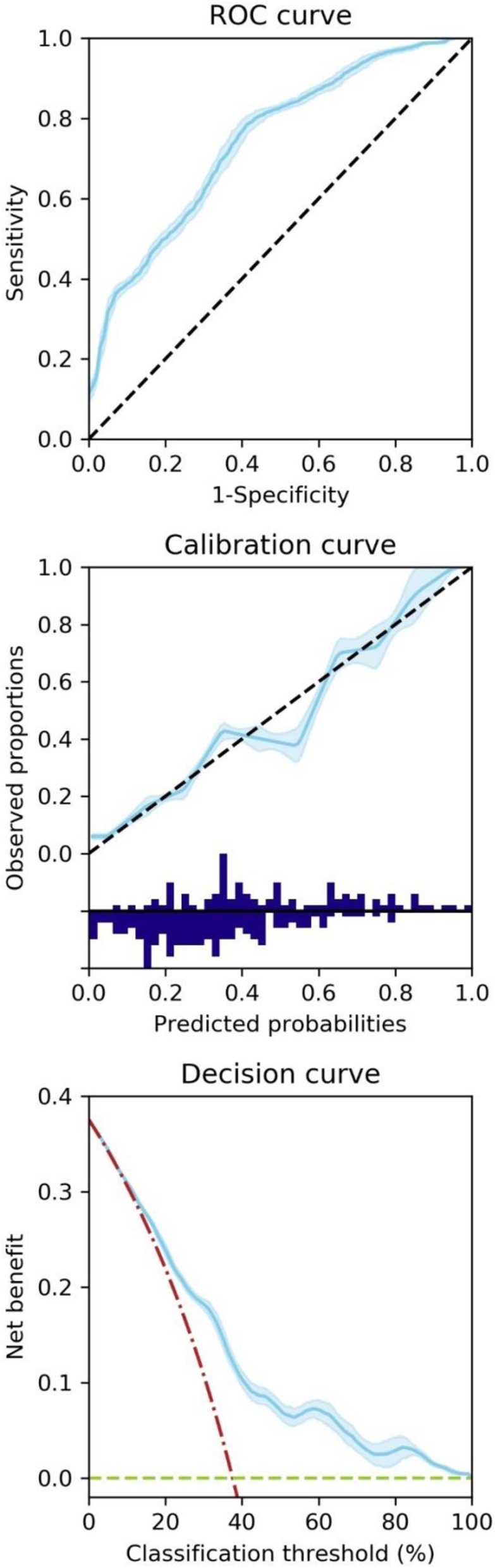
Internally validated model performance: (top row) ROC curve; (middle row) calibration curve; (bottom row) decision curve

## P233 Sepsis induced acute kidney injury: incidence, risk factors and prognostic impact in critically ill patients

### K Mnif^1^, R Ammar^2^, M Bahloul^2^, O Doukali^2^, C Ben Hamida^2^, M Bouaziz^2^

#### ^1^University Hospital Habib Bouguiba Sfax, Intensive Care Unit, Sfax, Tunisia; ^2^university hospital habib bouguiba sfax, intensive care unit, sfax, Tunisia

**Introduction:**

Acute kidney injury (AKI) is a frequent complication in critically ill patients and is associated with increased morbidity and mortality. Sepsis is one of the most Common cause of AKI.

**Methods:**

A prospective study was conducted over 6 months (January 01–June 30, 2018).We included patients with septic shock at admission or at any time during hospitalization.The AKI staging was based on KDIGO criteria.Patients were divided into two groups, a group with AKI (AKI+) and a group without AKI (AKI-).Then we compared the baseline characteristics, laboratory and physiologic data. Patients with AKI (AKI+) were subdivided according to their prognosis.

**Results:**

Were enrolled 75 patients. The mean (SD) age was 56.43(±18) years.Sex ratio was 1.91. Fifty-two (70%) patients developed AKI.SAPSII and SOFA score in admission were higher in patients with kidney injury [59 Vs 44 points (p= 0.002), 6.5 Vs 4 points ;(p=0.003)] respectively.The serum lactate level was significantly higher in (AKI +) group patients during the first day of septic shock [6.12± 1.38 mmol/l (AKI+)Vs 4.11± 0.79 mmol/l(AKI-);(p=0.002) ] and its clearance was lower [(32±10.99% (AKI +)Vs 61±13%(AKI-);(p=0.001)]. A significant difference was observed in C reactive protein level [224±114 mg/l (AKI +) Vs 124±77 mg/l (AKI-) ; (p=0.004)].Among (AKI+) patients, KADIGO III was observed in 59.6% of cases.Nineteen (36.5%) patients received hemodialysis.A normal kidney function was recovered in 40.4% of cases.AKI+ patients had a higher occurrence in Disseminated intravascular coagulation (32 Vs 3 patients, p=0.002),acute respiratory distress syndrome (18 Vs 2 patients; p=0.023) and cardiac dysfunction (20 Vs 1 patient, p=0.001).Mortality was higher in AKI group (67% Vs 9%; p=0.001).

**Conclusions:**

The development of septic AKI was associated with poor outcomes and prognosis.A better understanding of sepsis induced AKI pathway will enable us to develop targeted therapeutic protocols.Newer tools,permitting AKI early detection, may make these therapies more fruitful.

## P234

**Withdrawn**

## P235 Contrast induced acute kidney injury (CIAKI) – fact or fable?

### P Pekic^1^, M Percic^2^, D Ljubas^3^, M Mackovic^4^, N Maric^4^

#### ^1^Univ. Hospital “Sveti Duh”, Department for cardiovascular disease - Cardiac intensive care and arrhythmology unit, Zagreb, Croatia; ^2^Univ. Hospital “Sveti Duh”, Zagreb, Croatia; ^3^Institute for Emergency Medicine of Zagreb County, Zagreb, Croatia; ^4^Univ. Hospital “Sveti Duh”, Intensive Care Unit, Zagreb, Croatia

**Introduction:**

This study aims to show that contrast procedures do not significantly increase the risk of renal injury and should not be deferred. Traditionally CIAKI is the most important cause of in-hospital renal failure after nephrotoxic drugs and shock. Problem is also the non-uniform definition of CIAKI proposed by three different initiatives (AKIN, ESUR and KDIGO). AKIN, being the most rigorous, defines CIAKI as an increase in serum creatinine >0.3 mg/dL or >50% of baseline within 48hours.

**Methods:**

A retrospective observational single-centre cohort study analyzed 82 patients who underwent a contrast procedure with Iomeron 350. The first group underwent a CT pulmonary angiography (CTPA), and the second a coronary angiography with PCI. No patient was previously prepared (RAAS blockade removal, crystalloid administration etc). We studied demographics, history of CKD and comorbidities and their impact on the CIAKI by the AKIN criteria.

**Results:**

A total of 82 patients were divided into two groups (CTPA and PCI). CTPA group (20M, 21F) all had acute PE and the PCI group (28M, 13F) were treated for ACS. The mean age was 69 and 65 years respectively. CKD was more prevalent in the PCI group (8pt vs. 3pt) possibly explained by the more advanced atherosclerotic disease. Advanced CHD (NYHA III/IV) was found in 3pt (PCI) vs. 2pt (CTPA) while diabetes and shock were equally distributed (11pt and 5pt) in both groups. The mean amount of contrast was significantly higher in the PCI group (242.3mL vs. 60mL). The mean creatinine/eGFR measured before and after contrast in the CTPA group was 87.3/71.7 vs. 75.1/83 and in the PCI group 91.4/75.9 vs. 105.3/76.1. None of the patients, according to the AKIN definition, developed any significant rise in creatinine/eGFR despite existing comorbidities and risk factors.

**Conclusions:**

Contrast procedures with non-ionic contrast in patients with significant comorbidities and risk factors for secondary AKI pose minimal risk for CIAKI and therefore should not be deferred in clinical practice.

## P236 Estimating the GFR and predicting the AKI in cardiac surgery patients: role of bioelectrical impedance derived fat free mass

### V Vicka, E Januskeviciute, J Krauklyte, D Ringaitiene, J Sipylaite

#### Institute of Clinical Medicine, Faculty of Medicine, Vilnius University, Department of Anesthesiology and Intensive Care, Vilnius, Lithuania

**Introduction:**

The goal of this study was to determine whether changing the body mass (BM) with fat-free mass (FFM) in Cockcroft-Gault (CG) formula could provide a more accurate prediction of AKI in obese patients undergoing cardiac surgery.

**Methods:**

In this retrospective study, we reviewed institutional data of patients who underwent elective cardiac surgery in a tertiary referral university hospital. Baseline patient creatinine value was collected and GFR was estimated using the MDRD, CKD-EPI and CG formulas. CG formula was further modified by replacing the BM with FFM derived from the bioelectrical impedance analysis. Postoperative AKI was defined by KDIGO creatinine change definitions. Accuracy of the eGFR values to predict the AKI was calculated with ROC-AUC analysis. All the calculations were performed in different categories of BMI.

**Results:**

476 patients were included in the study, 67.2% of them were men, mean age was 64.5±10.8y. Median Euroscore II was 1.69 [IQR: 1.05-2.49]; 66.4% patients underwent CABG. Total of 138 patients were diagnosed with postoperative AKI (28.99%). The mean measured eGFR varied according to equation: MDRD 74.74±0.9, CKD-EPI 76.79±0.9, CG 86.42±1.4, mCG 61.19±1.0. When comparing AUC in different categories of BMI, the mCG appeared to be the only statistically accurate formula in patients with BMI 30–34.9 (Figure 1).

**Conclusions:**

The eGFR is a poor predictor of AKI in obese patients undergoing cardiac surgery. The FFM modified Cauckraft-Gault formula yield more accuracy in this specific group.

**Fig. 1 (abstract P236).**
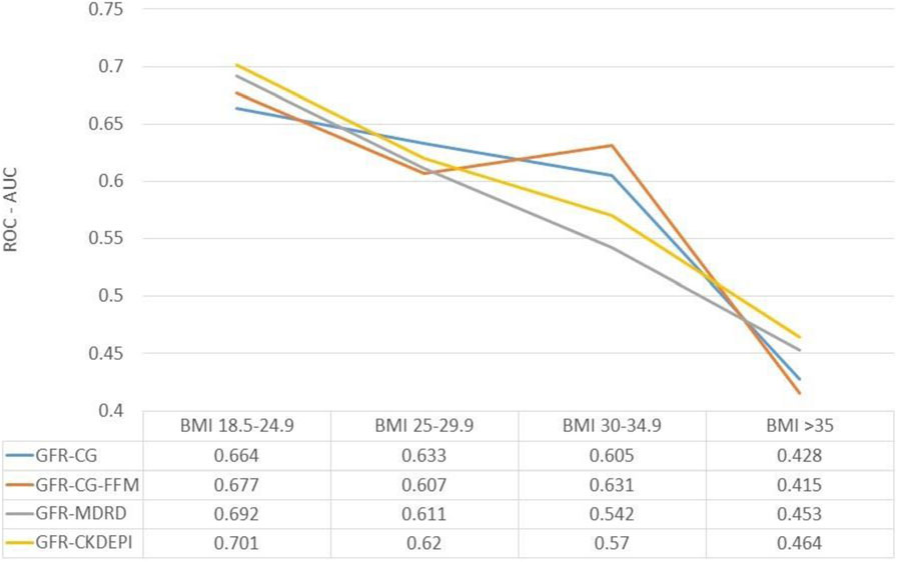
When comparing AUC in different categories of BMI, the mCG appeared to be the only statistically accurate formula in patients with BMI 30–34.9

## P237 RetroAKI: a ten-year retrospective study of acute kidney injury in intensive and progressive care units

### A Gardon, VZ Zorio, MB Bodinier, MD Dutour, CM Monard, JC Crozon, GM Marcotte, JT Textoris, TR Rimmelé

#### Edouard Herriot Hospital, Hospices Civiles de Lyon, Department of Intensive Care, 5 Place d´Arsonval, France

**Introduction:**

Acute kidney injury (AKI) is a frequent condition in intensive care units (ICU) and progressive care units (PCU), affecting 15% to 70% of the patients, depending on the studied population and AKI definition. AKI has been identified as an independent risk factor of ICU mortality and development of chronic kidney desease. The objective of this study was to describe the incidence of each AKI stages as defined by KDIGO definition (with evaluation of urine output, serum creatinine and initiation of renal replacement therapy (RRT)), in a mixed medical and surgical population of patients hospitalized in ICU and PCU over a 10-year period (2008-2018).

**Methods:**

We included all patients who stayed more than 12 hours in ICU or PCU of Edouard Herriot Hospital from May 2008 to January 2019. Data used to classify the patients were the urine output over a six-hour period, serum creatinine and the need for RRT, according to KDIGO classification

**Results:**

18,882 hospital stays were analyzed. Median ICU/PCU length of stay was 3 days [IQR: 1.5-6.6]. Among ICU patients, 74% had at least one AKI episode graded 1, 2 or 3 and 49% had at least one severe episode (stage 2 or 3). Among PCU patients, 44% had at least one episode of AKI and 20% a severe episode of AKI. Patients had an average of 1.9 episodes of AKI per stay. Table 1 represents the incidence of maximal AKI stage during one stay. We found that urine output was the more frequent criteria to make diagnosis of AKI stage 1 or 2 whereas RRT was more frequent for AKI stage 3.

**Conclusions:**

This retrospective study reports a more important AKI incidence in our ICU/PCU than in previous studies. The difference could be explained by the difficulty to collect urine output from conventional database. Serum creatinine and the use of RRT are often the only two criteria used to define and classify AKI. These results confirm the high incidence of AKI in ICU and PCU and the importance to make an early AKI screening of patients for whom preventive nephroprotective actions are needed.

**Table 1 (abstract P237).** Incidence of highest AKI stage reached during one ICU/PCU stay. AKI = AKI stage 1, 2 or 3 ; severe AKI = AKI stage 2 or 3

	ICU (n = 12356)	PCU (n = 6019)
No AKI (%)	25.4	56
AKI stage 1 (%)	25.4	24.4
AKI stage 2 (%)	23.6	15
AKI stage 3 (%]	25.5	4.6
AKI (%)	74.6	44
Severe AKI (%)	49.2	19.6

## P238 Epidemiology and outcomes of infection in AKI patients

### S Cattoir^1^, P Depuydt^1^, L De Bus^1^, E Hoste^2^

#### ^1^Ghent University Hospital, Dept. of Intensive Care Medicine, Ghent, Belgium; ^2^Ghent University Hospital, Dept. of Intensive Care Medicine/Research Foundation Flanders (FWO), Ghent, Belgium

**Introduction:**

ICU-patients with acute kidney injury (AKI) requiring renal replacement therapy (RRT) are at risk for infections [1,2]. In this study we evaluated the incidence of infection in ICU patients with and without less severe AKI. Finally, impact on outcomes was explored.

**Methods:**

This is a retrospective study on the PDMS (Protection Data Management System) of the 4 adult ICUs of a University Hospital. AKI was assessed on KDIGO criteria (creatinine (Scr) and urine output), during the first 7-d of ICU stay. Infection was validated in the PDMS by a team of ICU specialists.

**Results:**

During a 4-year period, a total of 7485 subjects were enrolled. AKI was diagnosed in 64.7% of patients during ICU stay. AKI patients were older (63 vs. 59 y, p=0.001), had higher SAPS 2 (57 vs. 41, p<0.001), and had more urgent ICU admission (64% vs. 48%, p<0.001). More AKI patients had mechanical ventilation (55% vs. 41%, p<0.001) and vasopressors on d-1 (47% vs. 23%, p<0.001). AKI stage 1, 2, and 3 was present in 25.5%, 28.0% and 11.1% of patients. More AKI patients had infection (57% vs. 28%, p<0.001) and increasing AKI stages were associated with higher infection rates (AKI-0: 28%; AKI-1: 55%, AKI-2: 55%, AKI-3: 69%, p<0.001) (Figure 1). We observed 2-3 times higher mortality in AKI patients with infection, and a stepwise increase of mortality with increasing AKI stages. After correction for infection and other confounders we found that all AKI stages were associated with in-hospital mortality (ORs AKI-1: 1.7, AKI-2: 2.0, AKI-3: 3.6, all p< 0.001).

**Conclusions:**

Over half of AKI patients experienced an episode of infection and increasing AKI severity was associated with higher infection rate. AKI patients with infection had marked higher mortality, suggesting that infection was an important driver of outcome. However, after adjustment, AKI stages had strong association with hospital mortality.

**References:**

1. Reynvoet E et al. Crit Care Med 37:2203–9, 2009.

2. Hoste EAJ et al. J Am Soc Nephrol 15:454–62, 2004

**Fig. 1 (abstract P238).**
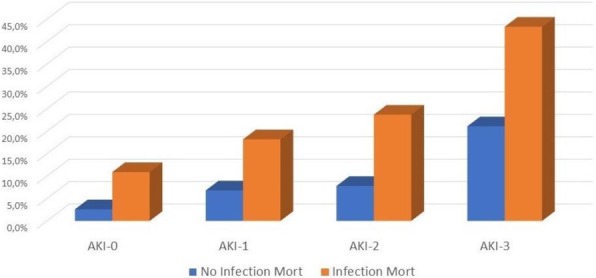
In-hospital mortality in patients with and without infection and different AKI stages. AKI, acute kidney injury; Mort, mortality

## P239 Prediction of outcome by routine measurement based of TIMP-2 (tissue inhibiting metalloproteinase 2) and IGFBP7 (insulin-like growth factor binding protein 7) by NephrocheckTM

### V Eising, A Beitz, B Henschel, M Messer, T Lahmer, RM Schmid, W Huber

#### Klinikum rechts der Isar der TU München, II Medizinische Klinik und Poliklinik, München, Germany

**Introduction:**

Several new biomarkers have been introduced to improve early diagnosis of acute kidney injury (AKI). “NephroCheck” (NC; Astute Medical, USA) is a bedside test calculating “AKIRisk” (product of urinary concentration of the cell cycle arrest-markers TIMP-2 and IGFBP7). Several studies suggest the usefulness of NC in selected populations. However, the value of early routine measurement of NC is unclear.

**Methods:**

Therefore, we compared the prediction of a combined endpoint (CEP: death <60 days and/or requirement of renal replacement therapy RRT) by NC within 12h of ICU admission (NC1) and 24h later (NC2) with admission values of serum-creatinine, BUN, cystatin C, urinary NGAL, APACHE II and SOFA (ROC-analysis). As a secondary endpoint we investigated the additional value of pathological measurements of NC1≥0.3 and/or NC2≥0.3 (NC+) in addition to AKI defined as KDIGO≥1 in a combined model (Kaplan-Meier-analysis). Statistics: SPSS 26.0.

**Results:**

106 patients of a general ICU (63±17 years; APACHE II 18±8; SOFA 6±4). ICU-mortality was 14/106 (13,2%), mortality <60d 33/106 (31,1%). De novo or acute on chronic (AoC) AKI according to KDIGO≥1: 65/106 (61,3%). Requirement of RRT 21/106 (19.8%). CEP 40/106 (37.3%). NC2 provided the largest ROC-AUC regarding the CEP (AUC=0.716; p<0.0001). Furthermore, cystatin C (AUC=0.715; p<0.001), BUN (AUC=0.700; p=0.001), creatinine (AUC=0.700; p=0.001), NC1 (AUC=0.659; p=0.008) and APACHE-II (AUC=0.651; p=0.012) significantly predicted the combined endpoint. Kaplan-Meier-analysis (p=0.024) demonstrated the lowest survival time for patients with both AKI and elevated NC1and/orNC2 (AKI+/NC+: 36±4d) compared to AKI+/NC- (42±8d), AKI-/NC+ (49±4d) and AKI-/NC- 54±4d).

**Conclusions:**

1) NC1 and NC2 were significant predictors of the combined endpoint CEP. However, their predictive capacity was not superior to cystatin C on admission. 2) By contrast, combination of NC with AKI according to KDIGO improves prediction of the combined endpoint vs. KDIGO alone.

## P240 The role of renal artero-venous coupling (RAVC) in the prediction of acute kidney injury in post-surgical critical care patients

### L Tecchi^1^, S Maiorano^1^, C Brusasco^2^, F Forfori^1^, F Corradi^3^

#### ^1^Azienda Ospedaliero Universitaria Pisana, Department of Surgical, Medical and Molecular Pathology and Critical Care Medicine, University of Pisa, Pisa, Italy., Pisa, Italy; ^2^Ospedali Galliera. Mura delle Cappuccine 14, 2. Anaesthesia and Intensive Care Unit, E.O. Ospedali Galliera, Genova, Italy., Genova, Italy; ^3^Azienda Ospedaliero Universitaria Pisana, Pisa, Italy

**Introduction:**

Objective: This study aimed to explore the diagnostic performance of a novel Doppler index expression of renal artero-to-venous coupling (RAVC) in the prediction of acute kidney injury (AKI-3) in post-surgical critically ill patients compared with systolic time intervals derived from electrocardiographic-gated intrarenal artery Doppler (STI), renal venous impedence index (VII) and renal Doppler resistive index (RDRI) [1, 2].

**Methods:**

Design: Prospective observational study including 45 critically ill patients. Doppler were measured within 6hours following admission to the intensive care unit (ICU). AKI was defined according to the Kidney Disease Improving Global Outcomes criteria (KDIGO). The new Doppler index RAVC was calculated as follows: (Cardiac Cycle Time – venous flow time)/Arterial Total Ejection time – Pre-Ejection Time).

**Results:**

Mean RAVC was 4.7±3.4 in patients with AKI 0–2 and 0.8±2.3 in patients with AKI 3 (P<0.001).  Mean STI was 0.82±0.60 in patients with AKI 0–2 and 0.50±0.27 in patients with AKI 3 (P<0.001). Median VII was 0.81±0.19 in patients with AKI 0–2 and 0.43±0.24 in patients with AKI 3 (P<0.001). Mean RDRI was 0.81±0.14 in patients with AKI 0–2 and 0.71±0.08 in patients with AKI 3 (P<0.001). As assessed by the area under the receiver operator characteristic curves (AUC), RAVC performed best in diagnosing AKI 3 [AUC=0.95 (95% CI: 0.83–0.99)] if compared with STI [AUC=0.90 (95% CI: 0.79–0.99)], VII [AUC=0.92 (95% CI: 0.83–0.99)] and RDRI [AUC=0.67 (95% CI: 0.43–0.92)] (Figure 1).

**Conclusions:**

In our study, artero-venous renal coupling has been shown to be the best predictor for AKI stage 3.

**References:**

1. Nijst P et al. JACC Heart Fail 5:672-681, 2017

2. Lee WH et al. Sci Rep 6:29293, 2016

**Fig. 1 (abstract 240).**
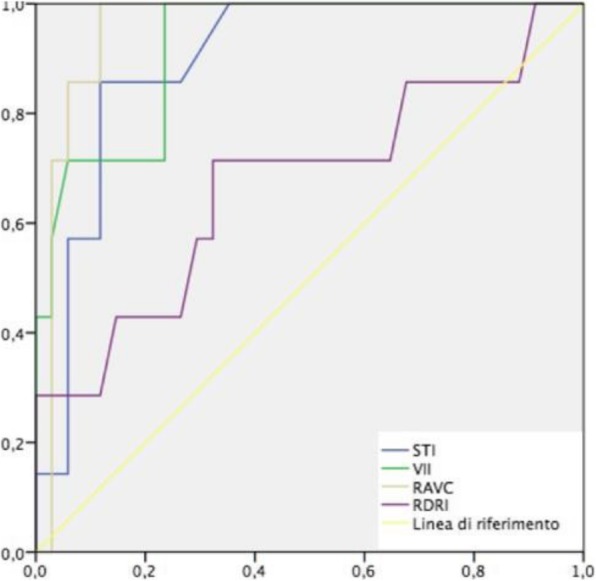
ROC

## P241 Influence of urinary creatinine excretion on augmented creatinine clearance in critically ill patients

### C Mendes Silva, JP Baptista, P Martins

#### Centro Hospitalar e Universitário de Coimbra, Serviço de Medicina Intensiva, Coimbra, Portugal

**Introduction:**

The first days of critical illness are associated with increased protein catabolism [1]. Urinary creatinine excretion (UCE) may be a predictor of endogenous protein catabolism [2]. This study aimed to evaluate the relation between UCE and creatinine clearance (CrCl), in particular, with augmented renal clearance (ARC), during intensive care unit (ICU) admission.

**Methods:**

Retrospective study in adult patients admitted to a multipurpose ICU of a tertiary center during 12 months. Urine was collected for an 8-h period and daily UCE was calculated by extrapolation to 24h. Relative UCE was calculated as a percentage of theoretical values obtained with equations proposed by Walser [3].

**Results:**

A total of 407 patients were included, with a mean age of 62.3±17.3 years, and 35.4% were female. Mean CrCl in the first 2 days of admission was <60, 60-129 and ≥130mL/min/1.73 m^2^ in 35.4%, 35.9% and 28.6% of patients, respectively. Considering only patients with a CrCl ≥60mL/min/1.73 m^2^, an analysis by age categories (20-39, 40-64 and 65-89 years), showed significant higher incidence of trauma on admission and higher CrCl in younger patients. Relative UCE in the first 2 days of admission was 119.4±37.5%, 114.5±39.5% and 115.0±42.6% for 20-39, 40-64 and 65-89 years groups (p=0.795) and decreased for all groups in the 3-7 days of admission period, with lower values for older patients (p=0.011). Relative UCE was positively associated with the presence of ARC in the different periods of time evaluated. In a multivariate logistic regression, 90-day mortality was not associated with relative UCE ≥ 100% or ARC.

**Conclusions:**

Critically ill patients showed increased relative UCE in the first days of ICU admission, which may be attributed to higher protein catabolism. Increased relative UCE was associated with ARC and both had no effect on 90-day mortality.

**References:**

1. Sharma K et al. Nutr Clin Pract 34: 12-22, 2019

2. Carlotti A et al. Q J Med 101: 197-205, 2008

3. Walser M. J Parenter Enteral Nutr 11:73S, 1987

## P242 Long-term outcomes of community- versus hospital-acquired acute kidney injury: a retrospective analysis of a large cohort in a German tertiary care center

### D Khadzhynov^1^, L Lehner^2^, D Schmidt^2^, K Eckardt^2^, K Schmidt-Ott^2^

#### ^1^Charité Universitätsmedizin Berlin, Department of Nephrology and Internal Intensive Care, Berlin, Germany; ^2^Charité Universitätsmedizin Berlin, Medical Department, Division of Nephrology and Internal Intensive Care Medicine, Berlin, Germany

**Introduction:**

This study compared epidemiology, short- and long-term outcomes for patients with community-acquired (CA) and hospital-acquired (HA) acute kidney injury (AKI).

**Methods:**

We retrospectively analyzed all episodes of AKI over a period of 3.5 years (2014–2017) on the basis of routinely obtained serum creatinine measurements in 103,161 patients whose creatinine had been measured at least twice and who had been in the hospital for at least two days. We used the “Kidney Disease: Improving Global Outcomes” (KDIGO) criteria for AKI and analyzed the first hospital admission. A total of 103161 were admitted in hospital and fulfilled the inclusion criteria. Average observation period per patient was 248 days.

**Results:**

The incidence of CA-AKI among included hospital admissions was 9.7% compared with an incidence of 8.6% of HA-AKI, giving an overall AKI incidence of 18.3%. Patients with CA-AKI were younger than patients with HA-AKI (64 vs 66.2y) and had significantly less comorbidities, including preexisting cardiac failure, ischemic heart disease, hypertension, diabetes. Patients with CA-AKI were more likely to have stage 1 AKI (69,3 vs 58,4%, p<0.001) and had significantly shorter lengths of hospital stay than patients with HA-AKI (14 vs 24d, p<0.001). Those with CA-AKI had better survival than patients with HA-AKI (Figure 1; p<0.001). Patients with CA-AKI were less likely dialysis dependent before discharge (1.9 vs 4.8% in HA-AKI; p<0.001). Patients with HA-AKI received more often administrative coding of AKI (29.3% vs 26.5% in CA-AKI patients; p<0.001)

**Conclusions:**

Patients with CA-AKI sustain less severe AKI than patients with HA-AKI, accordigly showing better short- and long-term outcomes. However, patiens with CA-AKI are at risk of inadequate clinical perception of AKI.

**Fig. 1 (abstract P242).**
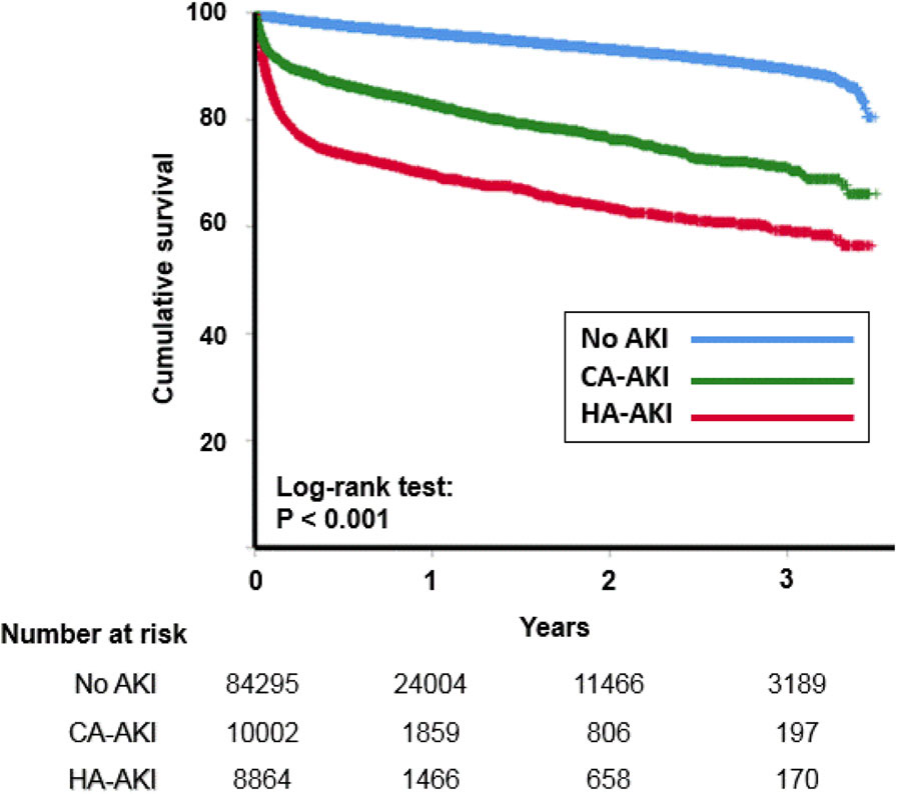
Long-term survival

## P243 Fluid removal practices during renal replacement therapy – a European survey of critical care practitioners

### N Seylanova^1^, L Tovey^2^, P McCready^2^, R Murugan^3^, M Ostermann^2^

#### ^1^Sechenov First Moscow State Medical University, Moscow, Russia; ^2^Guy’s and St Thomas’ NHS Foundation Trust, London, United Kingdom; ^3^University of Pittsburgh School of Medicine, Pittsburgh, United States

**Introduction:**

The evidence base for management of fluid removal during renal replacement therapy (RRT) is limited. A recent international survey revealed the extent of practice variation worldwide [1]. Our aim was to summarise the responses from Europe-based healthcare professionals who participated in the survey.

**Methods:**

The international self-administered, cross-sectional, internet-assisted, open survey was disseminated between January 2018 and January 2019 via website links and emails to members of different critical care societies.

**Results:**

485 participants from 31 European countries completed the survey of whom 365 (75%) were intensivists and 306 (63%) worked in university-based hospitals. Persistent oliguria / anuria was the most common indication for fluid removal (51% responders). The parameters which guided fluid removal included hemodynamic status (47% responders), cumulative fluid balance since admission (23% responders), and 24-hour fluid balance (17% responders). 90% of participants reported using CRRT with a median net ultrafiltration rate 98 mL/hr (IQR 51–108mL/hr) for hemodynamically unstable and a rate of 300 mL/hr (IQR, 201–352mL/hr) for hemodynamically stable patients. Only 26% of practitioners checked net fluid balance hourly (70% nurses, 16% physicians). New hemodynamic instability, defined as new onset or worsening tachycardia, hypotension, or need to start or increase the dose of vasopressors was reported to occur in 20% patients (IQR 10.0–30.0). Different strategies to re-gain hemodynamic stability were used. (Figure 1) Main barriers to fluid removal were patient intolerance (72% physicians, 85% nurses) and interruptions in fluid removal (43% physicians, 64% nurses). The majority of participants agreed that guidelines and protocols would be beneficial.

**Conclusions:**

The practice of fluid removal during RRT is very variable across European countries. Nurses and doctors identified a need for evidence-based protocols and clear guidelines.

**References:**

1. Murugan R et al. Crit Care Med 48:e87-e97, 2020

**Fig. 1 (abstract P243).**
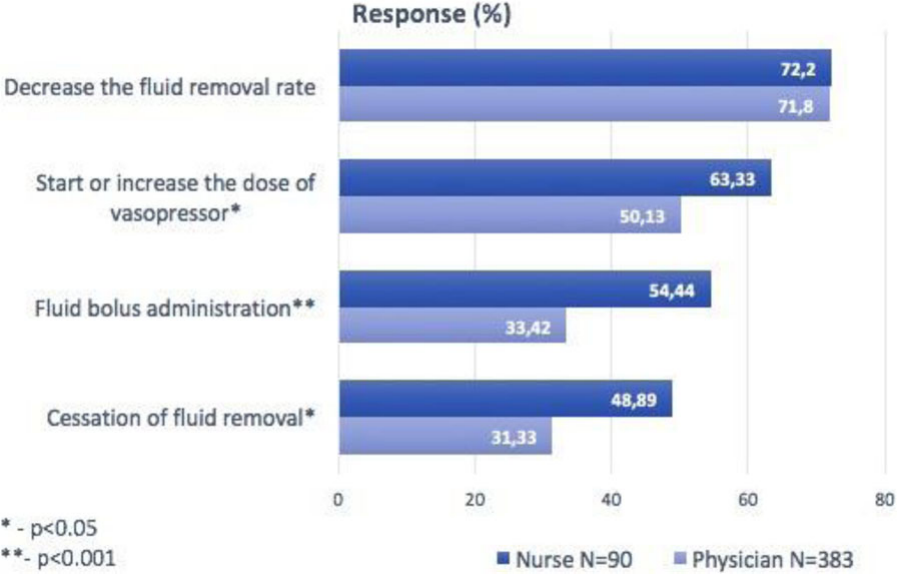
Common measures to correct hemodynamic instability during fluid removal

## P244 Comparing of regional citrate and systemic heparin anticoagulation methods in continuous renal replacement therapies on critically ill patients

### P Kucukdemirci kaya, FS Kahveci, P Rahimi, N Kelebek Girgin, R Iscimen

#### Bursa Uludag University, Anesthesiology and Intensive Care, Bursa, Turkey

**Introduction:**

Kidney Disease Improving Global Outcomes (KDIGO) guidelines suggest the use of anticoagulation in continuous renal replacement therapy (CRRT) [1]. The effectiveness of the anticoagulation is important because replacing the hemofilter and tube interrupts CRRT and increases total therapy time**.** Regional Citrate Anticoagulation (RCA) and Unfractionated Heparin (UFH) are most commonly using methods for CRRT anticoagulation [2]**.** The aim of this study was to investigate the efficacy, safety and metabolic differences of the patients in ICU who underwent CRRT and anticoagulation method changed from UFH to RCA for different reasons.

**Methods:**

After ethics committee approval (2019-14/9) 100 patients who underwent CRRT between 2018-2019 at Bursa Uludag University Hospital ICU have been investigated and 11 patients who underwent CRRT by both RCA and UFH included in the study. We divided patients in two groups (RCA, UFH), demographic data (sex, age), SOFA score, creatinine, urea, mean filter life time (FLT) and ultrafiltration flow (UF), platelets*,* electrolytes (Na, K, Ca, Mg), lactate, NaHCO3 and pH of groups at beginning and ending of first RCA and UFH hemodialysis collected. We used t-test and 1000 bootstraps statistic tests.

**Results:**

In agreement with other studies [3,4], FLT and UF was statistically significant lower in UFH group (Table 1). There was no statistically significant difference in efficiency (urea and creatinine decrease), pH, lactate, NaHCO3 level, platelets count and electrolytes between two groups. To our knowledge, there are no studies comparing these two anticoagulation methods in the same patients. Small number of patients and retrospective evaluation are limitations of the study.

**Conclusions:**

Our results suggest that the implementation of RCA method is safe and effective as UFH method with longer FLT and UF.

**References:**

1. Kellum JA et al. Kidney Int Suppl 2:1, 2012

2. Towlani AJ et al. Semin Dial 22:141, 2009

3. Hetzel GR et al. Nephrol Dial Transplant 26:232-9, 2011

4. Schilder L et al. Crit Care 18:472, 2014

**Table 1 (abstract P244).** Results

Gender (F/M)	4/7		
Age (year)	57,9±20,1		
SOFA score (during first CRRT)	11,3±4,9		
Sepsis (n)	10		
Cirrhosis (n)	2		
	UFH	RCA	p-value
Mean filter life time (hour)	40,5±38,9	67,8±29,8	<0,001
Mean ultrafiltration flow (ml/hour)	81,4±39,6	118,2±51,4	<0,001

## P245 Regional citrate anticoagulation during CRRT in liver failure

### MJ Jain, PK Kumar G, DG Govil, JK KN, SP Patel, MS Shafi, RH Harne, DP Pal, SM Monanga

#### Medanta the Medicity, Critical Care, Gurugram, India

**Introduction:**

Continuous renal replacement therapy (CRRT) with Regional citrate anti-coagulation (RCA) is increasingly being used as a treatment modality in critically ill patients. There is limited experience of use of citrate anticoagulation patients with acute liver failure and acute on chronic liver failure who pose a tough challenge of being at a higher risk for bleeding. An institutional protocol was formulated for use of commercially available citrate solutions and the same was studied to assess filter life and safety of citrate in liver disease. The primary objective was to assess safety of citrate anticoagulation in liver disease.

**Methods:**

This study was a single centre, prospective, non-randomized, single arm, observational study. All adult patients, with acute liver failure and acute on chronic liver failure requiring CRRT were included. Blood ionized calcium levels of 0.9 to 1.1mmol/l was targeted throughout the therapy and total to ionized calcium ratio of less than 2.4 was maintained. RCA was stopped if the ratio was more than 2.4 for 2 consecutive assessments. Incidence of citrate accumulation and toxicity were assessed. Average filter life was also assessed. Metabolic parameters, electrolytes and strong ion gap were followed till 24 hours after completion on CRRT.

**Results:**

A total of 25 patients were included in the study. Nineteen patients of acute on chronic liver failure and 6 patients of acute liver failure underwent CRRT with RCA. Baseline average serum bilirubin, lactate and INR were 11.8 mg/dL, 6.4 mmoL/L and 2.1 respectively. The average filter life was 50 hours 3 minutes. Citrate accumulation took place in (n=13) patients and RCA had to be stopped for ( n=6) patients due to the same. None of the patients had evidence of citrate toxicity.

**Conclusions:**

Citrate anticoagulation was well tolerated in patients with acute liver failure in patients with or without pre-existing chronic liver disease on CRRT.

## P246 Optimising regional citrate anticoagulation (RCA) in continuous renal replacement therapy (CRRT): a pre-study of complications and citrate loads

### N Hussain, I Carrasco

#### Barking, Havering and Redbridge University Hospitals NHS Trust, Critical Care Department, London, United Kingdom

**Introduction:**

The intention of this study is to highlight the levels of citrate load for the general population that increases the risk of citrate complications (insufficient trisodium citrate delivery; net citrate overload and citrate accumulation) [1].

**Methods:**

This was a prospective data collection between February and March 2019 in a fourteen bedded Critical Care Unit. Eleven consecutive episodes of CRRT were collected (a new episode characterized if CRRT was discontinued for 48 hours and above). One episode was excluded due to short duration (less than 4 hours). Patients undergoing RCA-CRRT received either a fixed 25 or 35 ml/kg/h effluent dose protocol.

**Results:**

Median patient age was 59, male 100%. Average time on CRRT was 4.1 days (2-9). 70% of the patients had complications, although 60% were minor (Figure 1). All of the patients with net citrate overload had citrate loads of 13.8mmol/h or above. The main risk factors were found to be shock and liver impairment which occurred in 60% of cases of which 40% developed complications.

**Conclusions:**

A fixed dose effluent protocol to standardise practice can potentially lead to a higher risk of minor complications. In our experience this is likely due to a lack of appropriate monitoring for RCA-CRRT complications. Despite this, our complication rate of citrate accumulation is in line with that reported in literature. Citrate loads in our 25 ml/kg/hr protocol were 22.6% higher than our 35 ml/kg/hr protocol and strongly related to higher complication rate that worsened in patients with risk factors for poor citrate metabolism.

**References:**

1. Morabito S et al. CJASN 9 :2173-2188, 2014

**Fig. 1 (abstract P246).**
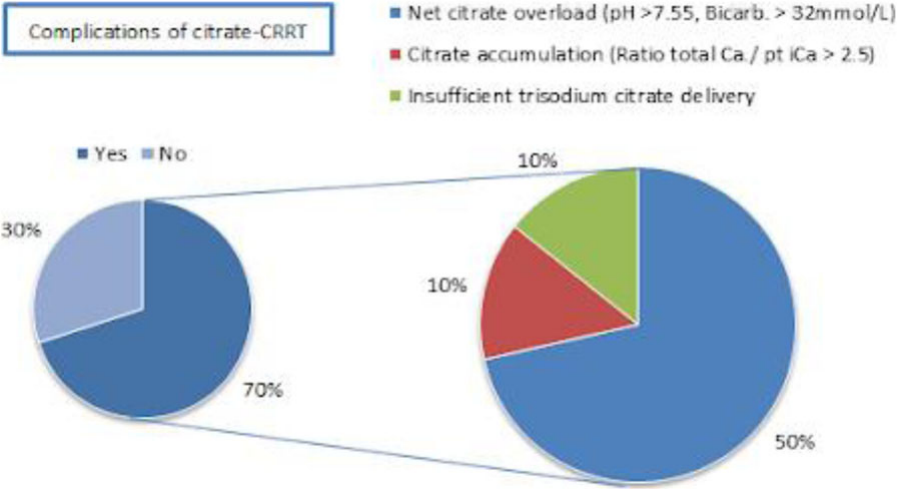
Incidence of complications

## P247 Delta-pH unmeasured anion: a predictor for starting continuous renal replacement therapy in acute kidney injury

### B Gucyetmez^1^, ZT Sarikaya^2^, F Tuzuner^3^, IO Akinci^4^, L Telci^5^, F Toraman^6^

#### ^1^Acıbadem Mehmet Ali Aydınlar University School of Medicine, Department of Anesthesiology and Reanimation, Istanbul, Turkey; ^2^Acıbadem Mehmet Ali Aydınlar University School of Medicine, Department of Anesthesiology and Reanimation, Istanbul, Turkey; ^3^Taksim Acıbadem Hospital, General Intensive Care Unit, Istanbul, Turkey; ^4^Altunizade Acıbadem Hospital, General Intensive Care Unit, Istanbul, Turkey; ^5^Acıbadem International Hospital, General Intensive Care Unit, Istanbul, Turkey; ^6^Acıbadem Mehmet Ali Aydınlar University School of Medicine, Department of Anesthesiology and Reanimation, Istanbul, Turkey

**Introduction:**

There is no optimal timing of continuous renal replacement therapy (CRRT) in acute kidney injury (AKI); however, it is based on volume overload, azotemia, hyperkalemia and severe metabolic acidosis [1]. An important reason for metabolic acidosis in AKI is increased unmeasured anions (UA) [2]. Delta-pH-UA (ΔpH_UA_) detects the degree of metabolic acidosis caused by UA and is calculated by using ‘The Partitioned pH Model’ [3]. In this study, we investigated whether ΔpH_UA_ was a predictor to start CRRT in patients with AKI.

**Methods:**

The study was designed as a multicentric, prospective, observational study in 2019. Patients who were ≥18 years old and diagnosed with AKI [1] were included. The moment AKI was diagnosed, arterial blood gas, albumin, magnesium, inorganic phosphorus, urea, creatinine and ΔpH_UA_ values were recorded. All patients were divided into two groups as CRRT(-) and CRRT(+) which consists of patients performed CRRT due to traditional criteria.

**Results:**

90 of 709 patients (12.7%) were diagnosed as AKI. CRRT rates in stage I, II and III AKI were 0.2% (1/41), 47.1% (16/34) and 100% (15/15) respectively. When patients with stage II were evaluated, the only parameter which was significantly different between CRRT(+) and CRRT(-) was ΔpH_UA_ (-0.124±0.076; -0.054±0.048) (p=0.001). Likewise, the only parameter which had significant UAC value to predict starting CRRT was also ΔpH_UA_ (for≤-0.083; 0.82 [0.67-0.97]) (p=0.002). In the logistic regression model, the likelihood of starting CRTT was increased 25-fold (3.5-169) by only ΔpH_UA_≤-0.083 (p=0.001).

**Conclusions:**

ΔpH_UA_ is prominently negative in patients with AKI performed CRRT and the only decisive parameter in stage II AKI in which clinicians have difficulty in deciding whether to start CRRT. Hence, we think that ΔpH_UA_ may be a promising parameter to detect the optimal timing of CRRT in AKI.

**References:**

1. KDIGO Guideline. 2012 Vol 2 Suppl 1

2. Kellum JA et al. Nephrol Dial Transplant 30:1104-11, 2015

3. Gucyetmez B et al. Int Care Med Exp 7(Suppl 3):55, 2019

## P248 Initiatives to enhance standard, workflow and nursing competence in delivery of safe continuous renal replacement therapy

### YC Wong, WT Yeung, PO Lei, PK Chan, YC Chiu, SY Chu, SC Chin, WS Shum

#### Ruttonjee & Tang Shiu Kin Hospitals, Cardiac & Intensive Care Unit, Hong Kong SAR, Hong Kong

**Introduction:**

Continuous renal replacement therapy (CRRT) is labor intensive and requires advanced nursing knowledge and skills. However, 40% of registered nurses (RN) are less than 2-year post-registration experiences in our unit. Also there is an increasing demand of CRRT from 185 CRRT days in 2017 to 248 CRRT days in 2018. The obstacles for CRRT in our department, includes variation of regimen, complicated workflow and insufficient training of nurses. A continuous quality improvement project is carried out to standardize the regimen, enhance workflow and provide structured training to nurses in the intensive care unit, to enhance nursing competence.

**Methods:**

1. Questionnaires was set and distributed to identify obstacles of safe CRRT care.
2. Workgroup was set up to carry out discussion and conduct literature review. New nurse led protocols were designed . A standardize CRRT doctor prescription form was designed to guide the new practice
3. Department journal club applied the evidence-based practice to introduce the new update practice
4. A single page CRRT nursing observation form was made to allow easy observation. The practice of safety check was introduced. Micro-teaching was conducted.
5. Lecture, demonstration and stimulation workshop was arranged.

**Results:**

1. Patient outcome showed 80% electrolyte imbalance (Potassium) was corrected within 8 hours of the commencement of the new CRRT regime.
2. CRRT circuit half-life is prolonged from 12 hours to 48 hours.
3. The new method of capping procedure . No catheter associated blood-stream infection was seen after the new method was launched.
4. Knowledge improvement from 70% in pretest to 98% passing rate after training.
5. 90% of experienced nurse expressed the theory enhancement session and simulation workshop were useful.
6. Majority of nurses can perform CRRT independently.

**Conclusions:**

By standardizing the CRRT regimen, improving workflow and providing structured training, patient safety and nursing staff competency are achieved.

## P249 Does hemoperfusion therapy affect survival in patients with sepsis?

### C Balci^1^, E Haftaci^2^, H Karaca^3^, E Karaca^3^

#### ^1^Kutahya Healty Science Un, Anaesthesiology and Reanimation, Kutahya, Turkey; ^2^Karaman Goverment Hospital, Intensive Care, Karaman, Turkey; ^3^Derince Traning Hospital, Anaesthesiology and Reanimation, Kocaeli, Turkey

**Introduction:**

Sepsis and septic shock is a leading cause of mortality in the intensive care unit. We tried to evaluate a novel hemoperfusion cartridge through a retrospective evaluation of patient’s data in our centre. We used it as an adjuvant therapy in our patients with Sepsis and septic shock due to varied causes. The aim of this study was to evaluate the efficacy of therapeutic hemoperfusion cartridge (HC-Foshan Biosun Medical ®^)^ in the management of patients with sepsis.

**Methods:**

We retrospectively analysed data of Group 1 (n=30 Sepsis) and Group 2 (n=30 sepsis+Hemoperfusison; sepsis treated with Hemoperfusion cartridge) admitted between 2015 to 2018. Group 2 had received Hemoperfusion cartridge as adjuvant therapy along with standard of care. Demographic data, procalcitonin [1] and leukocyte levels before and after therapeutic cytokine removal and duration of HC were recorded.

**Results:**

While the mean duration of CVVHDF was 96.4 hours, the duration of Hemoperfusion cartridge (application was 32.1±16.4 hours). Among 30 patients who survived 25 patients were administered hemoperfusion cartridge within 12 hours of ICU admission. There was a significant reduction in scores like APACHE and SOFA score post Hemoperfusion cartridge therapy procalcitonin and leucocyte levels after therapeutic Hemoperfusion cartridge were found significantly lower than the pretreatment values (respectively p=0.001, p=0.001). Retrospective analysis showed significant reduction of vasopressors, and improvement in MAP in Group2.

**Conclusions:**

Therapeutic Hemoperfusion cartridge with cytokine removal applied with CVVHDF in septic patients have positive contributions to provide survival advantage.

**References:**

1. Balci C et al. Crit Care 7 :85-90, 2003.

## P250 Immunomodulation in septic shock with an endotoxin removal device

### SA Shlyapnikov^1^, MI Gromov^2^, AV Fedorov^2^, LP Pivovarova^3^, ME Malyshev^3^, OB Ariskina^3^, IV Osipova^3^

#### ^1^Federal State Budgetary Institution “Saint Petersburg I.I. Dzhanelidze Research Institute of Emergency Medicine”, department of emergency gastroenterology, Saint-Petersburg, Russia; ^2^Federal State Budgetary Institution “Saint Petersburg I.I. Dzhanelidze Research Institute of Emergency Medicine”, department of efferent therapy, Saint-Petersburg, Russia; ^3^Federal State Budgetary Institution “Saint Petersburg I.I. Dzhanelidze Research Institute of Emergency Medicine”, department of laboratory diagnostics, Saint-Petersburg, Russia

**Introduction:**

Removal of activated leukocytes and endotoxin from the blood is a complex therapeutic effect of the device for removing endotoxin.

**Methods:**

In the main group (16 patients with abdominal septic shock) after surgery, the traditional treatment was supplemented with two sessions of endotoxin removal (2 hours each with an interval of 24 hours) using “Alteco LPS adsorber” (Sweden). The control group consisted of 8 patients with a similar diagnosis and only traditional treatment.

**Results:**

28% of white blood cells were adsorbed in LPS adsorber. Among them, granulocytes (35%) were maximally extracted, then CD14 ^+^ monocytes (CD14^+^ Mo) (33%), HLA-DR ^+^ mononuclear cells (6%), monocytes (2%). IL-6, IL-10, procalcitonin (PCT) were not adsorbed. The 28-day mortality rate in the main group was 50% and was lower compared to the control group - 75%. During monitoring, in the main group 24 hours after the first removal of endotoxin, a decrease in the initially increased amount of activated CD14^+^ Mo by 2.2 times, as well as functionally mature defensin^+^ granulocytes (def^+^ Gran) by 1.6 times was observed. IL-6, IL-10, and PCT decreased by 1.9; 17.8; and 1.2 times, respectively. During this period, the control group showed an increase in CD14^+^ Mo and def ^+^ Gran, while IL-6, IL-10 did not change, and PCT increased 1.9 times. A day after the second removal of endotoxin and then 5 days later, the main group of IL-6, IL-10, and PCT continued to decline. In the control group, only IL-10 decreased after 3 days, the rest continued to grow.

**Conclusions:**

The cellular adsorption of endotoxin-bound CD14 ^+^ Mo and mature def ^+^ Gran is an important part of the mechanism of action of the endotoxin removal device.

## P251 Does the endotoxin adsorption of PMX column saturate in 2 hours? Preliminary study

### C Yamashita^1^, K Moriyama^2^, D Hasegawa^1^, T Kawaji^1^, N Kuriyama^1^, T Nakamura^1^, Y Shimomura^1^, S Suzuki^1^, Y Kato^1^, O Nishida^1^

#### ^1^Fujita Health University School of Medicine, Department of Anesthesiology and Critical Care Medicine, Toyoake, Japan; ^2^Fujita Health University School of Medicine, Laboratory for Immune Response and Regulatory Medicine, Toyoake, Japan

**Introduction:**

In the EUPHRATES trial, the polymyxin B-immobilized fiber column (PMX) hemoperfusion (HP) had no significant effect on 28-day mortality. Endotoxin (LPS) burden by endotoxin activity assay >0.90 may exceed 50 μg [1], so the dose and duration of PMX-HP could be insufficient to lower the LPS burden. To confirm this issue, we experimented in a closed-circuit with 24 h continuous LPS addition, and PMX can adsorb > 50μg [2]. Further, LPS concentration became constant within 2 h in the single LPS spike test for determining PMX-HP duration [3]. To prove our hypothesis that the single LPS spike test reflects the adsorption equilibrium, and not saturation, we added LPS intermittently to reaction.

**Methods:**

LPS (10 ng/mL) was mixed with 125 mL deactivated fetal calf serum as a reflux solution, as previously described [2]; this concentration is much higher than that observed in septic patients. We created a closed circuit that incorporates PMX-01R at 1/14^th^ the amount of an adult PMX and performed PMX-HP at 10 mL/min for 5 h. LPS was added in two shots (post 2 h: 1250 ng, 10 ng/mL; post 4 h: 3750 ng, 30 ng/mL). LPS was measured using the Limulus Amebocyte Lysate test at 0, 0.5, 1, 2, 3, 4 and 5 hr.

**Results:**

After an initial decrease between 0 and 1 h, LPS concentration did not decrease between 1 and 2 h after PMX-HP initiation. Post LPS pulse addition at 2 h, it increased and then decreased till 3 h. Futher, it did not decrease between 3 and 4 h, but it increased and then decreased again after LPS pulse addition post 4 h (Figure 1). LPS adsorption rates were 76.2, 43.4, and 40.7% at 2, 4, and 5 h, respectively.

**Conclusions:**

LPS adsorption capacity of PMX-01R was maintained even after two additional shots of LPS, suggesting that the constant LPS concentration in the previously reported LPS spike test might be indicative of adsorption equilibrium rather than saturation.

**References:**

1. Dellinger RP, et al. JAMA 320:1455-1463, 2018

2. Sakai Y, et al. Ther Plasmapheresis 12:837-842, 1993

3. Yamashita C, et al. Blood Purif 46:269-273, 2018

**Fig. 1 (abstract 251).**
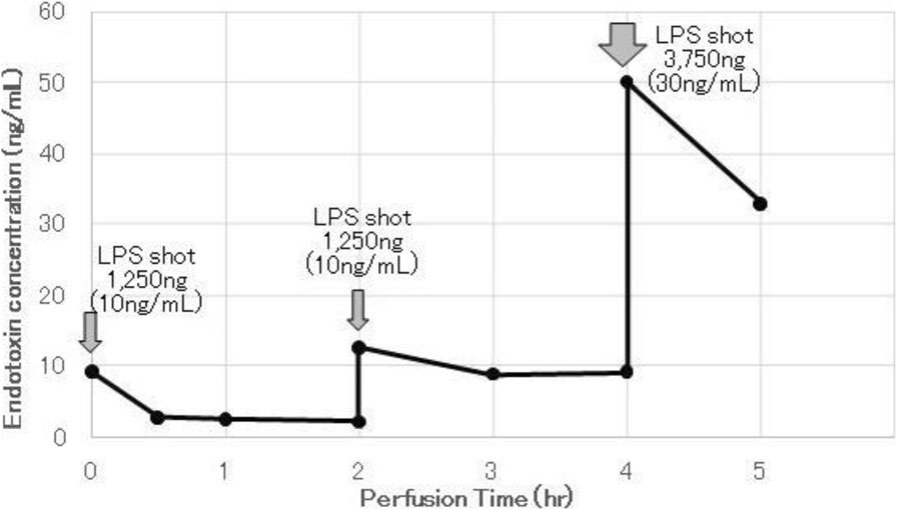
LPS concentration in LPS pulse addition test

## P252

**Withdrawn**

## P253 CRRT with the oXiris filter in septic patients with AKI: effect of the basal AKIN stage

### F Turani^1^, S Busatti^2^, S Martini^3^, F Gargano^4^, M Falco^4^, L Weltert^5^, M Dauri^6^, P Caravetta^7^

#### ^1^Aurelia Hospital, Anesthesia and Intensive Care, Rome, Italy; ^2^Aurelia Hospital, Rome, Italy; ^3^Aurelia Hospital, Anesthesia And Intensive Care, Rome, Italy; ^4^European Hospital, Anesthesia And Intensive Care, Rome, Italy; ^5^European Hospital, Cardiac Surgery, Rome, Italy; ^6^University Tor Vergata, Anesthesia And Intensive Care, Rome, Italy, ^7^Ospedale S Camillo, Anesthesia And Intensive Care, Rome, Italy

**Introduction:**

Exta-corporeal blood purification ( BF ) is used in patients with sepsis – septic shock . However the main Critical Care  Societies don’t suggest it’s use, because of the negative RCT. Probably lacking of precise clinical end points and stratification of the patients  may explain these results. The aim of this study is to evaluate whether 1- Patients submitted to CRRT with the oXiris stratified on the basal AKIN stage have different response. 2- This translates in a different clinical response.

**Methods:**

A coohort study included 65 patients admitted to three Intensive Care  with  sepsis / septic shock ( SEPSIS 3 Criteria ) and AKI ( AKIN score). All patients were submitted to CVVHDF with the oXiris filter (Baxter, USA) .   The main clinical data, Il 6, Procalcitonin, Endotoxin ( EAA ) and SOFA score were evaluated at basal time ( T0 ) and at the end of the treatment ( T1 ). All data are expressed as mean ± SD or median and IQR . ANOVA TEST was used to compare the changes in the time.

**Results:**

60 patients were submitted to RRT with the oXiris filter for 46 ± 12 hours . 21 patients had AKI 3 stage , 13 patients AKI 2 stage and  25 patients had AKI 1 stage. At T0 all groups had an high vasopressor support to maintain MAP ≥ 70 mmHg. IL6, Procalcitonin EAA and SOFA total were also elevated with no difference between the groups. At T1 creatinine improved better in AKI 2 ( p< 0.001 vs. T0 ) and in AKI 1 ( p< 0.0001 vs T0) then in AKI 3 group. MAP increased in AKI 2 ( p< 0.01 vs T0)  and AKI 1 ( p < 0.01 vs T0) , but not in AKI 3 group. IL6, procalcitonin decreased more in AKI 1 ( p < 0.0001 vs T0) then AKI 3 . At T 2 SOFA total was higher in AKI 3 then AKI 1 ( p< 0.001 ) and AKI 2 ( p< 0.01 ).

**Conclusions:**

AKI 2 and AKI 1 stage patients submitted to BP with the Filter oXiris respond better then AKI 3 stage patients . 2 – This transalte in a better clinical course. 3- CRRT with Oxiris filter is useful in septic patients with AKI, but AKI 3 stage septic patients represent an high risk group.

## P254 A non-interventional, multicenter, non-randomized patient registry for multiple organ dialysis with the ADVOS system: 2-year interim analysis in 118 patients

### V Fuhrmann^1^, A Perez^2^, A Faltlhauser^3^, B Tyczynski^4^, D Jarczak^1^, J Lutz^5^, J Weinmann-Menke^6^, W Huber^7^, A Kribben^4^, S Kluge^1^

#### ^1^Department of Intensive Care Medicine, University Medical Center Hamburg-Eppendorf, Hamburg, Germany; ^2^ADVITOS GmbH, München, Germany; ^3^First Department of Internal Medicine, Kliniken Nordoberpfalz AG, Klinikum Weiden, Weiden, Germany; ^4^Klinik für Nephrologie, Universität Duisburg-Essen and Universitätsklinikum Essen, Essen, Germany; ^5^Medical Clinic, Nephrology-Infectious Diseases, Gemeinschaftsklinikum Mittelrhein, Koblenz, Germany; ^6^Division of Nephrology, I. Department of Medicine, University Medical Center Mainz, Mainz, Germany; ^7^Medizinische Klinik und Poliklinik II, Klinikum rechts der Isar, Technische Universität München, Munich, Germany

**Introduction:**

Multiple organ failure is a challenging problem in the ICU. As an advanced dialysis system, the ADVOS procedure can eliminate water-soluble and protein-bound substances, regulate the acid-base balance as well as fluid and temperature. In 2017, a national registry was established to collect data under "real-life" conditions of patients treated with ADVOS without any trial-specific interventions (DRKS ID: DRKS00017068).

**Methods:**

Data from 01/2017 to 02/2019 from 4 German hospitals (university hospitals in Hamburg-Eppendorf, Mainz, Essen, and Klinikum Weiden) were analyzed. Clinical parameters, treatment settings and adverse events were documented. The 28- and 90-day mortality rates were compared with extrapolated rates based on the SOFA score.

**Results:**

118 patients with a median age of 60 years (IQR 45-69), of whom 70 (59%) were male, were evaluated. Patients had a median SOFA score of 14 (IQR: 11-17) before the 1st ADVOS treatment, which is associated with an expected mortality of 80%. The number of failing organs was 3 (IQR 2-4): cardiovascular (74%), lungs (57%), liver (47%), kidneys (74%), coagulation (69%) and CNS (29%). 429 treatments with a median duration of 16 (IQR: 10-20) hours were evaluated. 87 were discontinued, of which 25 (6%) were due to a device error. 79 adverse events were documented, 13 were related to the device (all due to clotting and recovered without sequelae). Significant removal of protein-bound (bilirubin: 11.2 vs 9.2 mg/dl) and water-soluble toxins (BUN 32 vs 20 and creatinine 1.9 vs 1.4 mg/dl). In addition, improvement in acid-base balance was observed: pH (7.33 vs. 7.40), bicarbonate (21.3 vs. 25.5 mmol/l) and base excess (-4.5 vs. 1.0 mmol/l) (Table 1). 28- and 90-day mortality rates were 60% and 65%, respectively.

**Conclusions:**

In a cohort of patients with multiple organ failure, we observed an improvement in the expected mortality rate, especially if the ADVOS procedure was applied early. Adverse events are comparable to other dialysis therapies in intensive care patients.

**Table 1 (abstract P254).** ADVOS treatment performance parameters

Parameter	Before 1st ADVOS	After 1st ADVOS	p-value
Bilirubin total [mg/dl]	11.2 ± 1.2	9.2 ± 0.8	<0.001
Creatinine [mg/dl]	1.9 ± 0.2	1.4 ± 0.1	<0.001
BUN [mg/dl]	32 ± 3	20 ± 2	<0.001
pH	7.33 ± 0.01	7.40 ± 0.01	<0.001
HCO3 [mmol/l]	21.3 ± 0.6	25.5 ± 0.7	<0.001
Base Excess [mmol/l]	-4.5 ± 0.8	1.0 ± 0.8	<0.001
Platelets [/nl]	129 ± 15	100 ± 13	<0.001

## P255 Cyclosporine does not affect cytokine release in response to extracorporeal circulation in coronary artery bypass grafting

### EG Grins^1^, SJ Jovinge^2^

#### ^1^Skåne University Hospital, Lund, Department of Cardiothoracic Surgery, Anesthesia and Intensive Care, Lund, Sweden; ^2^Fredrik Meijer Heart and Vascular Institute, Spectrum Health, Fredrik Meijer Heart and Vascular Institute, Grand Rapids, Michigan, United States

**Introduction:**

Acute kidney injury (AKI) due to ischemia-reperfusion affects one-third of the patients in cardiac surgery. We investigated the potential role of Cyclosporine (CsA) to prevent postoperative AKI and mitigate inflammatory response to extracorporeal circulation (ECC).

**Methods:**

Double-blind, randomized, placebo-controlled single-center study. Patients (n=67) scheduled for elective cardiac surgery were randomized to 2,5 mg/kg CsA or placebo before the surgery. The primary objective was to assess the role of CsA to reduce the incidence of postoperative AKI. The secondary objective was to study CsA induced changes in the inflammatory response to ECC.

**Results:**

All enrolled patients were analyzed. Postoperative AKI was more pronounced in the Cyclosporine group compared to placebo. OR=5.03 (1.76-15.74), 95% CI. The cytokine production in response to ECC was not affected by Cyclosporine (Figure 1).

**Conclusions:**

In patients undergoing cardiac surgery, a single preoperative dose of CsA does not prevent the postoperative decrease in renal function. CsA does not alter cytokine release in response to extracorporeal circulation. Elevated post-ECC levels of pro-inflammatory cytokine IL-6 are associated with kidney dysfunction and may be predictive.

**Fig. 1 (abstract P255).**
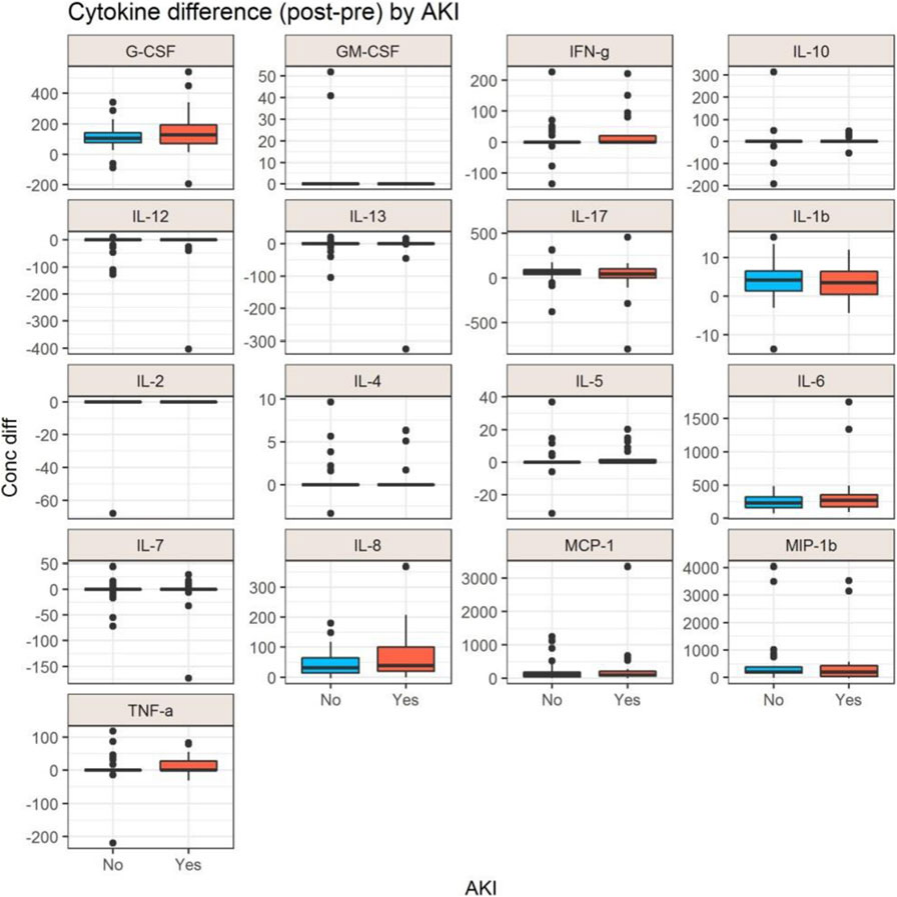
Differences in cytokine concentration before and after extracorporeal circulation and AKI. AKI defined as a 30% increase of preoperative Cystatine C

## P256 New generation adsorbent in hemofiltration helps reducing inflammatory response in septic shock patient

### M Cindryani^1^, V Irawany^2^, M Taufik^2^

#### ^1^Faculty of Medicine, Universitas Indonesia, Jakarta, Indonesia; ^2^Fatmawati Hospital, Jakarta, Indonesia

**Introduction:**

New generation adsorbent such as oXiris^R^ was introduced as novel technique in renal support for critically ill patients [1]. Septic shock patients require decatecholaminization strategies emphasizing blood purification to remove catecholamine-producing mediators and evacuate overload fluid in interstitials.

**Methods:**

Our 64-year-old female patient, admitted to ICU after surgery with history of ovarium cancer. Her septic shock was worsened with ARDS, hypercoagulable state and AKI. Vasopressors were set. Patient was controlled with mode SIMV16,PS12,TV350 ml,PEEP7,FiO270%. Renal support was implemented by diuretic and CVVH started on the second day. At first,regular adsorbent was used, post-filter mode was set, and periodic fluid removal target was 50 ml/h. But after 24hours, no significant changes observed. OXiris^R^ added and after 12 hours passed, requirements of vasopressors reduced, tidal volume increased, hemodynamic parameters stabilized, urine production increased. It was continued for 2 days and patient was recovered.

**Results:**

Our patient had fallen into inadequate CARS stage in which not able to counter septic effects on vital organs (Figure 1). Renal would be primary target for filtration and monitoring tool. Adsorbent consisted of AN69 and polyethyleneimine was useful to purify blood from endotoxins conjoined with slower filtration. Continuous yet cautious process in CVVH evacuate fluid and mediators while maintain steady hemodynamics. Biomarkers could not be evaluated due to limited resources, but improving parameters could be signs that showed recovery process had already took place.

**Conclusions:**

Advanced hemofiltration is a privilege. Implementing and enhancing it with new generation adsorbent would increase survivors by extracting unnecessary fluids and eliminating catastrophic endotoxins and mediators.

Consent to publish: written informed consent for publication was obtained from the patient.

**References:**

1. Schwindenhammer V et al. Blood Purif 47 (suppl 3) :29-35, 2019

**Fig. 1 (abstract P256).**
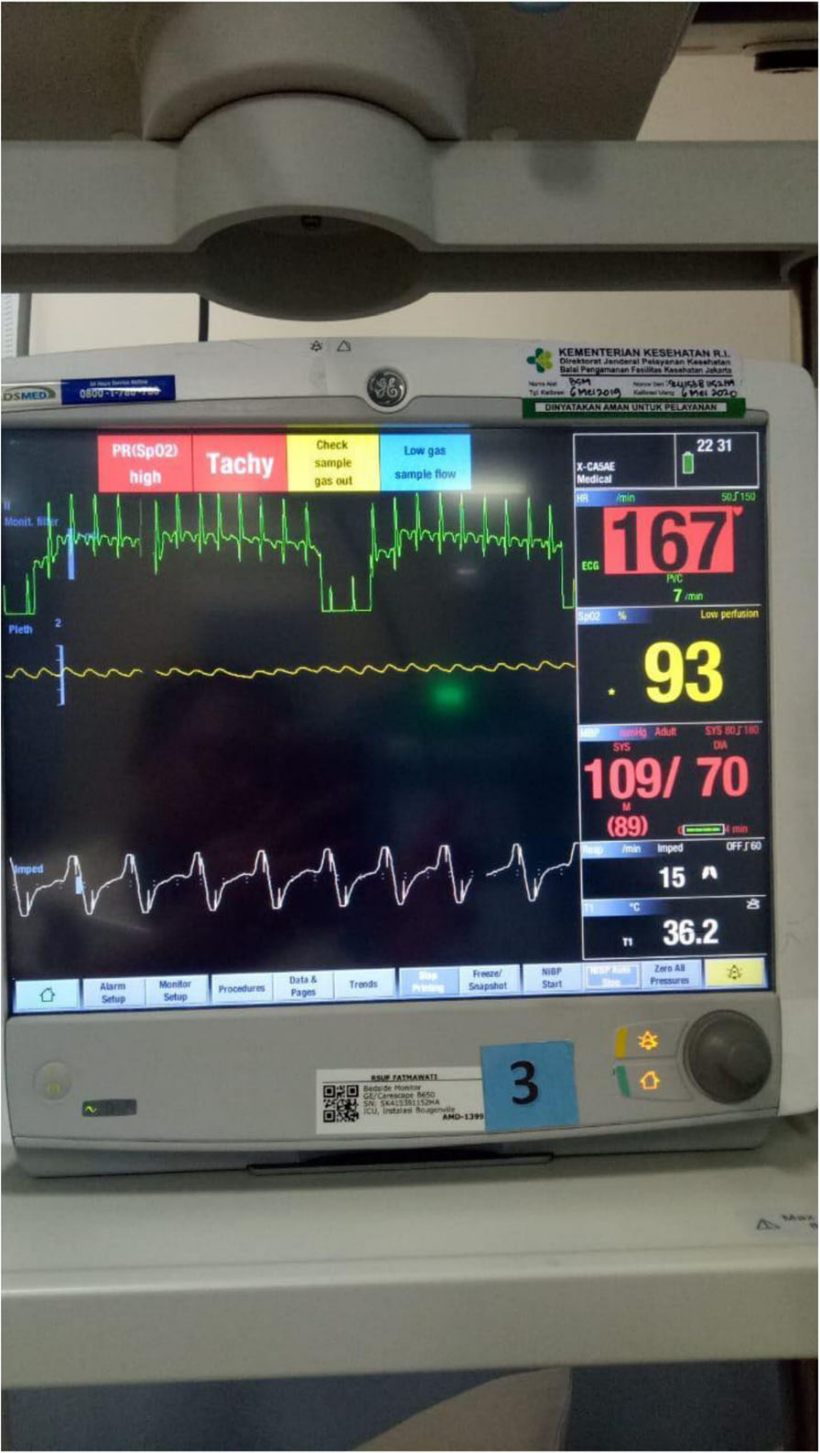
Clinical presentations before CVVH with oXiris

## P257 Influence of complications of diabetic ketoacidosis treatment on length of stay at intensive care unit

### D Adukauskiene, L Jazokaite, R Verkauskiene

#### Lithuanian University of Health Sciences, Medical Academy, Kaunas, Lithuania

**Introduction:**

Aim of study was to estimate the rate of hypokalemia and hypoglycemia as diabetic ketoacidosis (DKA) treatment complications, relate them also insulin interruption and use of sodium bicarbonate (NaH_2_CO_3_^-^) with length of stay (LOS) in ICU.

**Methods:**

Analysis of retrospective cohort study data of 120 patients (pt) treated for DKA at ICU of Kaunas Clinics during 2014 - 2019 has been carried out. Serum kalemia, glycemia; hypokalemia, hypoglycemia episodes; rate of insulin interruption for hypo- and normoglycemia during ketoacidosis; use of NaH_2_CO_3_^-^ for ketoacidosis, and LOS in ICU were analysed. SPSS 23.0 was used for statistic calculations. Traits evaluated as significant at p < 0.05.

**Results:**

At the beginning of DKA treatment in totally hypokalemia (3.1 ± 0.3 mmol/l) was recorded in 64/120 pt (53.3 %). Due to ignoring of blood pH (6.8 - 7.3 (7.0 ± 0.1) kalemia was falsely misinterpreted as “normo-“ or “hyper-“ 3.5 – 7.1 (5.1 ± 0.9 mmol/l) in 49/68 pt (72.1 %), thus disregarded so complicated by obvious hypokalemia additionally in 26/49 pt (53.1 %). In hypokalemia LOS in ICU was 52.9 ± 29.7 vs 32.8 ± 18.6 h, p < 0.05. Insulin use has caused hypoglycemia (1.2 – 3.3 (2.5 ± 0.7 mmol/l)) in 22/120 pt (18.3 %), LOS in ICU 63.2 ± 38.5 vs 38.9 ± 21.2 h, p < 0.05.Insulin use was interrupted in case of normo - and hypoglycemia with still persisting ketoacidosis in 39/120 pt (32.5 %), LOS in ICU was found to be 56.5 ± 30.7 vs 37.0 ± 22.5 hr, p < 0.05. NaH_2_CO_3_^-^ was given for symptomatic treatment of ketoacidosis during first 10 h of DKA in 33/120 pt (27.5 %) with stable hemodynamic: HCO^-^_3_ buffer has increased (4.8 ± 3.3 - 7.9 ± 3.1 mmol/l), p < 0.05, but it didn’t control ketoacidosis, and LOS in ICU was 55.2 ± 27.5.2 vs 39.1 ± 25.6 h, p < 0.05.

**Conclusions:**

Hypokalemia, hypoglycemia, precocious interruption of insulin use were recorded as complications of DKA treatment. All of them have prolonged LOS in ICU. Symptomatic treatment of ketoacidosis with NaH_2_CO_3_^-^ had no effect on it, and prolonged LOS in ICU as well.

## P258 Central venous-arterial PCO2 difference to arterio-venous oxygen content difference ratio, Pcv-aCO2/Ca-cvO2, in patients with metformin associated lactic acidosis (MALA): a marker of anaerobic metabolism?

### A Casazza^1^, E Bellazzi^2^, D Ciprandi^2^, R Preda^2^, R Vanzino^2^, L Carnevale^2^

#### ^1^ASST Pavia, Anaesthesia and Intensive Care Vigevano, Vigevano, Italy; ^2^ASST Pavia, Vigevano, Italy

**Introduction:**

A growing interest exists about CO_2_ derived parameters in shock management. Central venous-arterial PCO_2_ difference (P_cv-a_CO_2_) is strictly related to cardiac output; central venous-arterial PCO_2_ difference to arterial-central venous O_2_ content difference ratio, P_cv-a_CO_2_/C_a-cv_O_2_, has been proposed as anaerobic metabolism when it’s >1.4 mmHg/ml [1].

**Methods:**

To evaluate P_cv-a_CO_2_/C_a-cv_O_2_ reliability in detecting anaerobic metabolism, we analyzed it in 7 consecutive patients affected by MALA admitted to our ICU, considering these patients as a prevalent anaerobic metabolism model. We calculated, by Douglas formula, central venous-arterial CO_2_ content difference to arterial-central venous O_2_ content difference ratio, C_cv-Ca_CO_2_/C_a-Ccv_O_2_, as a Respiratory Quotient surrogate. We performed arterial and central venous blood gas analysis simultaneously at admission, we calculated P_cv-a_CO_2_, P_cv-a_CO_2_/C_a-cv_O_2_ and C_cv-a_CO_2_/C_a-cv_O_2_ and we recorded ScvO_2_. We verified relationship between P_cv-a_CO_2_/C_a-cv_O_2_ and ScvO_2_ and arterial pH, arterial lactates, SOFA score at admission and C_cv-a_CO_2_/C_a-cv_O_2_ by linear regression analysis.

**Results:**

Pcv-aCO2/Ca-cvO2 greatly increases in MALA (2.16 ± 0.84). Pcv-aCO2/Ca-cvO2 (Fig.1) shows significant co-variation with pH (R2=0.618; p=0.003) and SOFA score at admission (R2=0.628; p=0.003). Pcv-aCO2/Ca-cvO2 has poor agreement with Ccv-aCO2/Ca-cvO2 (R2=0.008) and disagrees with it in identifying anaerobic metabolism, in our series, in fact, Ccv-aCO2/Ca-cvO2 is, in 3 patients, < 1 like an aerobic RQ value. Pcv-aCO2/Ca-cvO2 shows better agreement with pH, SOFA score and lactate level than ScvO2.

**Conclusions:**

In our series, P_cv-a_CO_2_/C_a-cv_O_2_ is good illness and acidosis severity marker, but it seems to be affected by pH value in accord with Haldane effect [2]. P_cv-a_CO_2_/C_a-cv_O_2_, in our study, doesn’t seem to be a reliable anaerobic metabolism marker nor a RQ surrogate.

**References:**

1. Mekontso-Dessap A et al. Intensive Care Med 28:272-277, 2002.

2. Teboul JL et al. Intensive Care Med 43:91-93, 2016

**Fig. 1 (abstract P258).**
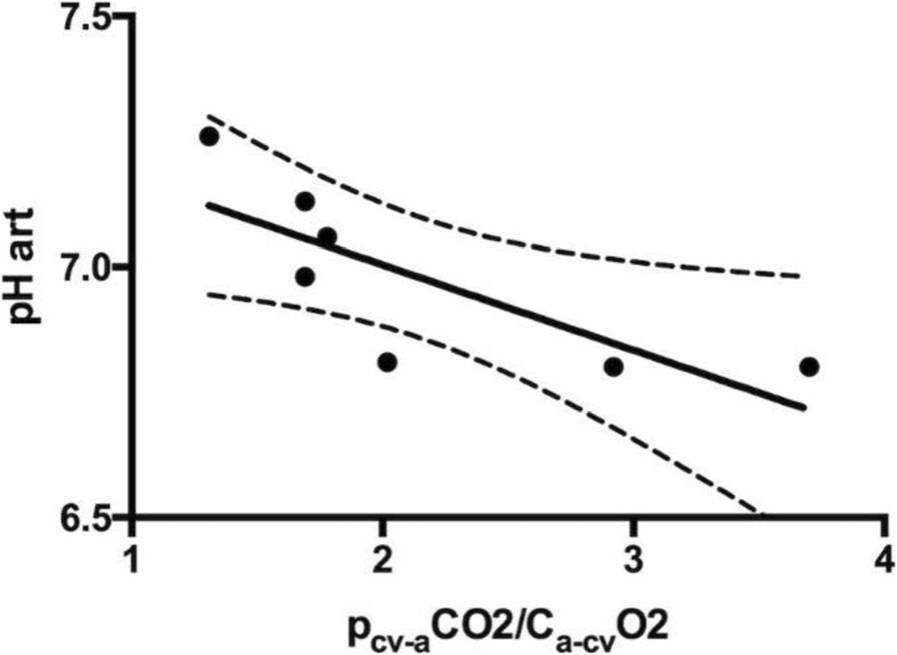
Pcv-aCO2/Ca-cvO2 variation with pH

## P259 Early versus delayed administration of basal insulin in diabetic ketoacidosis: a retrospective study

### M Dustrude, TS Lam, JT Jancik

#### Hennepin County Medical Center, Pharmacy, Minneapolis, United States

**Introduction:**

It is thought that early administration of basal insulin to patients with diabetic ketoacidosis (DKA) may improve outcomes. Small studies have shown trends towards decreases in time to closure of anion gap (TCAG), rates of rebound hyperglycemia following discontinuation of intravenous (IV) insulin, rates of hypoglycemia, intensive care unit (ICU) length of stay (LOS), and hospital LOS [1-4].

**Methods:**

This was a single-center, retrospective chart review of our institution’s DKA protocol between January 2010 and August 2019. Patients that received early basal insulin within 24 hours of initiation of IV insulin and before closure of the anion gap (AG) were compared to those that did not receive early basal insulin. The primary outcome was median TCAG. Secondary efficacy outcomes include: time on IV insulin infusion, time to de-escalation of level of care, hospital LOS, and re-elevation of AG. Secondary safety outcomes included incidences of hyperglycemia, hypoglycemia, and hypokalemia.

**Results:**

A total of 334 patients were identified meeting inclusion and exclusion criteria. Median TCAG was longer in the experimental group (9 vs. 6 hours, p <0.01). Incidence of re-elevation of AG and incidence of hyperglycemia were lower in the experimental group. Other outcomes were similar (Figure 1).

**Conclusions:**

Early administration of basal insulin to patients with DKA resulted in a longer TCAG with a lower incidence of re-elevation of AG and hyperglycemia. Early administration of basal insulin appears to be safe with respect to hypoglycemia and hypokalemia.

**References:**

1. Hsia E et al. J Clin Endocrinol Metab 97:3132-7, 2012

2. Doshi P et al. Acad Emerg Med 22:657-66, 2015

3. Houshyar J et al. J Clin Diag Res 9:1-5, 2015

4. Rappaport S et al. J Endocr Soc, 3:1079-86, 2019

**Fig. 1 (abstract P259).**
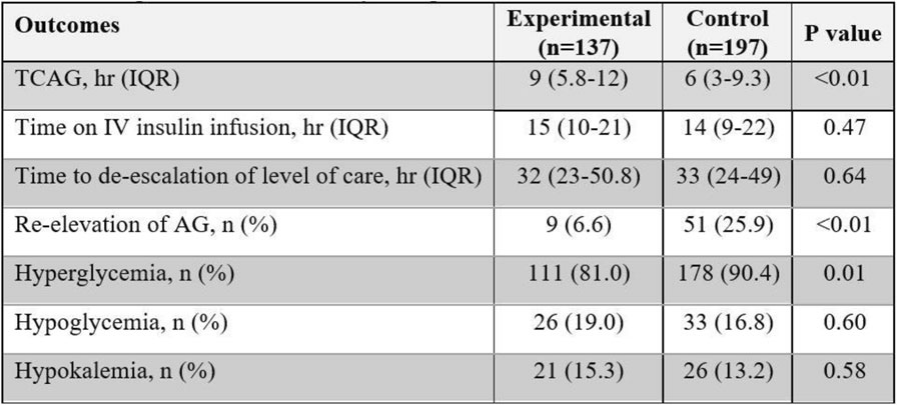
Results

## P260 Glycaemic control in critically ill patients – a work in progress

### M Andonovic, A Kinsey, H Browne, P O´Neil, R Sundaram

#### Royal Alexandra Hospital, Department of Intensive Care, Paisley, United Kingdom

**Introduction:**

Glycaemic control continues to be a challenge in critically ill patients. Stress induced hyperglycaemia has been associated with increased morbidity and mortality [1]. Conversely, patients receiving intensive glucose control have a higher risk of death [2]. A quality improvement project was designed to develop a comprehensive insulin protocol that recognized pre-existing diabetes and reduced hypoglycaemia.

**Methods:**

Data was collected prospectively in all adult patients admitted to the RAH intensive care unit (ICU) between October 2018 and August 2019 from the national ICU audit database and electronic patient records. Daily figures were collected for numbers of hypoglycaemic episodes (<4mmol/l), “in range” (4-10mmol/l) blood sugar measurements and patients with a pre-existing diagnosis of diabetes. Data was collected and analysed using Microsoft Excel.

**Results:**

307 patients were identified; 56 patients (18.2%) had pre-existing diabetes. A total of 6908 blood sugar measurements were reviewed; 5268 (76.3%) were “in range” and 126 hypoglycaemic episodes (1.8%) occurred. There was no significant correlation between number of diabetic patients and measurements within range. Of note, there was an increase in number of measurements per patient in the second half of the time period (11 vs 32).

**Conclusions:**

The development of this protocol has improved glycaemic control in our ICU. There are considerably fewer episodes of hypoglycaemia and a large proportion of blood sugar measurements are in range. We hope to continue data collection and interrogate the prevalence of pre-existing diabetes further to reduce glycaemic variability.

**References:**

1. Silva-Perez LJ et al. World Journal of Diabetes 8:89-96, 2017

2. The NICE-SUGAR Study Investigators. N Engl J Med 360:1823-1297, 2009

## P261 Time in blood glucose range 110 to 179 mg/dl > 80% is associated with survival rate in critically ill patients

### H Naraba^1^, H Nakano^2^, Y Takahashi^2^, T Sonoo^2^, H Hashimoto^2^, K Nakamura^2^

#### ^1^Hitachi General Hospital, Department of Emergency and Critical Care Medicine, Ibaraki, Japan; ^2^Hitachi General Hospital, Ibaraki, Japan

**Introduction:**

The optimal management of blood glucose levels for critically ill patients remains unclear. Hypoglycemia, hyperglycemia and glycemic variability are associated with mortality. The time in targeted blood glucose range (TIR) has been suggested to correlate with mortality depending on the status of antecedent glycemic control, but It has not been verified optimal TIR and whether there is an optimal disease-specific TIR.

**Methods:**

A retrospective observational study was performed at a single center. In the present study, we enrolled all critically ill patients admitted in intensive care unit from 1 January 2016 to 31 October. Patients with diabetic ketoacidosis or hyperosmolar hyperglycemic syndrome and patients who had < 10 blood glucose readings were excluded. Gathered information included, in part, demographics, comorbidities, severity of illness scores, diagnosis at admission, length of ICU stay and hospital discharge status. The primary outcome was 28-day mortality. We analyzed to find the optimal TIR for critically ill patients. Several TIRs were each tested for correlation with mortality.

**Results:**

A total of 1,523 patients, 51.8% of whom had diabetes, were studied. TIR 70 to 139 mg/dL (OR, 0.33; 95%CI, 0.18-0.58), TIR 70 to 179 mg/dL (OR, 0.33; 95%CI, 0.23-0.47) and TIR 110 to 179 mg/dL (OR, 0.28; 95%CI, 0.17-0.44) > 80 % was independently associated with mortality in critically ill patients respectively. The optimal TIR did not differ depending on diagnosis at admission.

**Conclusions:**

In this retrospective evaluation, TIR 110 to 179 mg/dL > 80 % was independently associated with mortality in critically ill patients, especially those with good antecedent glucose control. These findings have implications for the design of future trials of intensive insulin therapy.

## P262 Prevalence and impact of chronic dysglycemia in intensive care unit patients

### A Balintescu^1^, I Palmgren^2^, M Lipcsey^3^, A Oldner^4^, A Larsson^5^, M Cronhjort^6^, M Lind^7^, J Wernerman^8^, J Mårtensson^4^

#### ^1^Karolinska Institutet Södersjukhuset, Department of Clinical Science and Education, Stockholm, Sweden; ^2^Hudiksvall Hospital, Department of Anesthesia and Intensive Care, Hudiksvall, Sweden; ^3^Uppsala University, Hedenstierna Laboratory, Department of Surgical Sciences, Section of Anaesthesiology and Intensive care, Uppsala, Sweden; ^4^Karolinska Institutet, Department of Physiology and Pharmacology, Stockholm, Sweden; ^5^Uppsala University, Department of Medical Sciences, Clinical Chemistry, Uppsala, Sweden; ^6^Karolinska Institutet Södersjukhuset, Department of Anesthesia and Intensive Care Södersjukhuset, Stockholm, Sweden; ^7^Uddevalla University, Department of Medicine, NU Hospital Group, Uddevalla, Sweden; ^8^Karolinska Institutet, Department of Clinical Science Intervention and Technology, Division of Anaesthesia and Intensive Care, Stockholm, Sweden

**Introduction:**

The prevalence of chronic dysglycemia (diabetes and prediabetes) in patients admitted to Swedish intensive care units (ICUs) is unknown. We aimed to determine the prevalence of such chronic dysglycemia and asses its impact on blood glucose control and patient-centred outcomes in critically ill patients.

**Methods:**

In this retrospective, observational study, we obtained routine glycated hemoglobin A1c (HbA1c) measured in patients admitted to four tertiary ICUs in Sweden between March and August 2016. Based on previous diabetes history and HbA1c we determined the prevalence of chronic dysglycemia (prediabetes, undiagnosed diabetes and known diabetes). We compared indices of acute glycemic control in the ICU and explored the association between chronic dysglycemia and ICU-associated infections, mechanical ventilation, renal replacement therapy, vasopressor therapy, and mortality within 90 days.

**Results:**

Of 943 patients, 312 (33%) had chronic dysglycemia. Of these 312 patients, 127 (41%) had prediabetes or undiagnosed diabetes and 185 (59%) had a known diabetes diagnosis. During ICU stay, patients with chronic dysglycemia had higher average blood glucose, spent less time in target glucose range, had greater glucose variability, and were more likely to develop hypoglycemia than patients without chronic dysglycemia. Chronic dysglycemia was associated with greater need for renal replacement therapy (odds ratio 2.10, 95% CI 1.35-3.27) and increased 90-day mortality (hazard ratio 1.33, 95% CI 1.01-1.77) after adjustment for Simplified Acute Physiology Score 3. In contrast, chronic dysglycemia was not associated with mechanical ventilation, vasopressor therapy, or ICU-associated infections.

**Conclusions:**

In four tertiary Swedish ICUs, measurement of HbA1c showed that 1/3 of patients had chronic dysglycemia (prediabetes or diabetes). Chronic dysglycemia was associated with marked derangements in glycemic control during ICU stay, greater need for renal replacement therapy and with increased mortality at 90 days.

## P263 Case report: modern antidiabetic therapie causes ketoacidosis

### AM Heiden, M Emmerich

#### Krankenhaus Bad Oeynhausen, Institut für Anästhesie, Bad Oeynhausen, Germany

**Introduction:**

The modern antidiabetic class of SGLT2-inhibitors, that are known to reduce the risk for cardiac events [1], are increasingly used in the last few years. A 68-year old male patient with diabetes mellitus suffered 10 days after colectomy surgery from abdominal pain and nausea. The patient had an antidiabetic therapy with empaglifozin that was paused until day 5 after surgery (nutrition start on day 5, weaning on day 6).

**Methods:**

This is a case report of one male patient seen in the ICU setting. Daily blood values including arterial blood gases, vital parameters and clinical status of the patient were observed and evaluated.

**Results:**

The blood gases showed this metabolic acidosis: pH 7.38; PCO2 20.3 mmHg, bicarbonate 12 mmol/l, BE -11.63 mmol/l, lactate 1.6 mmol/l, glucose 7 mmol/l. A ketonuria despite normal blood glucose values was noticed, so that the diagnosis of ketoacidosis was clear. After analyzing the possible causes we found out, that empaglifozin in times of catabolism and fasting can cause this severe symptomatic. We terminated the therapie with empaglifozin and under the treatment with insulin the symptoms disappeared within 3 days and the patient could be discharged from the ICU on day 17 after surgery. After one episode of ketoacidosis the therapy with SGLT2-inhibitors should lifelong never be started again.

**Conclusions:**

We recommend that intensivists should be aware of the modern SGLT2-inhibitors because of the shown severe complications and the increased use of this medication.

Consent to publish: written informed consent for publication was obtained from the patient.

**References:**

[1: SGLT2-Inhibitor Empagliflozin: Jardiance auf einen Blick, DtschArztebl International-34-11620, 2019

## P264 Unraveling the obesity paradox in the intensive care unit: a causal inference approach

### A Decruyenaere^1^, J Steen^1^, K Colpaert^2^, D Benoit^2^, J Decruyenaere^2^, S Vansteelandt^3^

#### ^1^Ghent University Hospital, Department of Internal Medicine and Pediatrics, Gent, Belgium; ^2^Ghent University Hospital, Department of Intensive Care Medicine, Gent, Belgium; ^3^Ghent University, Department of Applied Mathematics, Computer Science and Statistics, Gent, Belgium

**Introduction:**

While obesity confers an increased risk of death in the general population, numerous studies have reported an association between obesity and improved survival among critically ill patients. This contrary finding has been referred to as the obesity paradox. This retrospective study uses two causal inference approaches to address whether the survival of non-obese critically ill patients would have been improved if they had been obese.

**Methods:**

The study cohort comprises 6,557 adult critically ill patients hospitalized at the Intensive Care Unit of the Ghent University Hospital between 2015 and 2017. Obesity is defined as a body mass index of ≥30 kg/m^2^. Two causal inference approaches are used to estimate the average treatment effect in the untreated (ATU): a naive approach that uses traditional regression adjustment for confounding and that assumes missingness completely at random, and a robust approach that uses super learning within the targeted maximum likelihood estimation framework and that uses multivariate imputation of missing values under the assumption of missingness at random.

**Results:**

Obesity is present in 18.9% of patients. The in-hospital mortality is 14.6% in non-obese patients and 13.5% in obese patients. The marginal associational risk difference for in-hospital mortality between obese and non-obese patients is -1.06% (95% confidence interval (CI) -3.23% to 1.11%, P=0.337). The naive approach results in an ATU of -2.48% (95% CI -4.80% to -0.15%, P=0.037), whereas the robust approach yields an ATU of -0.59% (95% CI -2.77% to 1.60%, P=0.599).

**Conclusions:**

A robust causal inference approach that may handle confounding bias due to model misspecification and selection bias due to missing data mitigates the obesity paradox, whereas a naive approach results in even more paradoxical findings. The robust approach does not provide evidence that the survival of non-obese critically ill patients would have been improved if they had been obese.

## P265 Patterns of bowel motions in ICU and the implementation of a stepwise management protocol to reduce decision fatigue

### C Cole, H McConnell

#### Royal Victoria Infirmary, Perioperative and Intensive care, Newcastle upon Tyne, United Kingdom

**Introduction:**

Bowel management within an ICU environment is often difficult. Recent data collection from an intensive care unit at the RVI identified either loose stool or constipation on > 50% of patient days. It was postulated this could be improved with a more tightly controlled bowel management regimen. To test this hypothesis a step-wise bowel protocol was created and introduced. Data was collected in the 4 month period following its implementation with the following aims:

1) Assess effectiveness of the protocol

2) Further observe the reasons for loose or constipated stool on an ICU

**Methods:**

Bowel data recorded once a day for each patient. A single recording was defined as ’one bowel day’. Definitions: Loose stool - type 5-7; Constipated - type 1-2 or BNO for > 3 days; ‘Normal bowel activity’ - type 3-4 or BNO < 3 days. In addition, the number and result of per rectal examinations performed was recorded.

**Results:**

1) 512 bowel days: Constipation 17%, ‘normal bowel’ 59%, loose stools 24%. (NOTE: Pre protocol 693 bowel days collected Constipation 30%, Normal bowel 47%, Loose stools 23%)

2) 86 PRs. 65% empty rectum, 35% stool present

3) Total number of days BO 157. Total number of days BNO 355

4) When BO type of stool as follows: Type 1-2 3 days; Type 3-4 30 days; Type 5-7 124 days

**Conclusions:**

Re. protocol

1) Did not cause harm

2) Decreased constipation. Increased ‘normal bowel days’

3) loose stool days unchanged - discussion required

Re. Observational

4) When BO predominant motion is loose stools (124 days out of 157 days - 79%) - also seen in previous studies

5*) PR results show 65% of constipation was the result of an empty rectum

6*) Days of non-defecation (355) was significantly larger than defecation (157) - 69% vs 31%.

7*) During the 512 days only 3 were recorded as hard stools

Conclusions from 5)6)7) Constipation in ICU more likely to be the result of non-defecation (reduced activity of the gut/empty gut) than loaded/hard stool. If unable to PR preference should be towards oral laxative as opposed to suppository/enema

## P266 Ogilvie syndrome in ICU

### P Delaney, S Delaney, A Fahy, M Donnelly

#### Tallaght University Hospital, Intensive Care Department, Dublin, Ireland

**Introduction:**

Acute colonic pseudo-obstruction (ACPO) or Ogilvie Syndrome is a disorder characterized by acute dilatation of the colon in the absence of mechanical obstruction. ACPO can result in colonic ischemia and perforation, leading to increased morbidity and mortality. Reported risk factors include trauma, major surgery, sepsis, shock, electrolyte imbalance, renal failure and medications such as opiates, sedatives and anti-cholinergics [1]. To date there are few reports of ACPO in the intensive care cohort. We investigated the incidence and risk factors associated with ACPO.

**Methods:**

The ICU admission database was interrogated to identify all patients diagnosed with ACPO from October 2018 to October 2019. In study subjects, a retrospective chart review was carried out recording patient demographics, admission diagnosis and severity of illness. Charts were examined for risk factors associated with ACPO and for details of the management of ACPO once diagnosed.

**Results:**

Of 400 admissions, 7 patients (2 female) developed ACPO, (mean age 52.2 years, mean APACHE 2 score 15.6 (range 8-22). Five patients were admitted with respiratory failure. Mean time to diagnosis of ACPO was 7.1 days after ICU admission. All patients were mechanically ventilated at the time of diagnosis. Six patients received a cephalosporin as per stewardship guidelines. All patients were on corticosteroid therapy. One patient responded to conservative management and one patient is receiving ongoing treatment. Five patients developed a colonic perforation, 3 of these underwent emergency surgical intervention. All patients who developed a colonic perforation died.

**Conclusions:**

ACPO is a condition with multiple associations in critically ill patients and substantially increases mortality risk. Further research is required into causality to better inform prevention of ACPO in ICU patients.

**References:**

1. Wells C et al, World J Gastroenterol 23:5634, 2017

## P267 Diarrhea in intensive care unit

### Z Binici, S Bozbay, O Demirkiran

#### Istanbul University Cerrahpasa, Anesthesiology and Intensive Care, Istanbul, Turkey

**Introduction:**

Diarrhea is an important problem in each critically ill pateints [1]. We aimed to investigate the frequency and management of diarrhea in our ICU.

**Methods:**

In this study 47 patient retrospectively reviewed, in our ICU between 01.01.2017-03.01.2018. Patients were divided into two group as diarrhea “positive” and “negative”. Patients with diarrhea had fluid or loose stools 3 or more times a day. Each diarrhea period of the patients with diarrhea was examined separately and compared with the group without diarrhea. Nutritional status, enteral product formulation, leukocyte, neutrophil, albumin values, gastric sparing, antibacterial and antimycotic use, LOS in hospital and in ICU were compared. In diarrhea positive group, on the day of hospitalization, laxative and/or enema administration, toxin A in stool, nitrogen balance before and after diarrhea, enteral product change in diarrhea, probiotic, metronidazole or oral vancomycin use were examined.

**Results:**

The incidence of diarrhea was 68.3%. The most common diagnosis of ICU admision was respiratory failure (60-85%) in both groups. Diarrhea occurred in two days after laxative and/or enema treatment. Enteral nutrition was higher in both groups (≥90%). Nasogastric tube feeding was significantly higher in the diarrhea group (p=0.041). There was no difference between nutritional product formulation and diarrhea development (p>0,1). Antibacterial use was high in both groups (75%); however, Teicoplanin use was significantly higher in the group diarrhea negative group (p=0.028). The LOS in ICU, and hospital was higher in diarrhea group (p<0.001). No difference in mortality rates (p>0.5).

**Conclusions:**

Many factors may cause diarrhea in ICU, and diarrhea may adversely affect patient treatment and increase morbidity. We think that preventive methods are as important as the treatment of diarrhea.

**References:**

1. Murali M. J Intensive Care Soc 1:1-7, 2019

## P268 Prognostic value of malnutrition in cardiac surgery. an eight year follow up data

### S Efremov^1^, V Lomivorotov^2^, P Vedernikov^2^, T Dzhumatov^3^, T Ovchinnikov^3^, A Rashidov^3^

#### ^1^Saint Petersburg University Hospital, Anesthesiology and Intensive care, Saint-Petersburg, Russia; ^2^E. Meshalkin National Medical Research Center, Anesthesiology and Intensive care, Novosibirsk, Russia; ^3^St. Petersburg State University, Medical faculty, Saint-Petersburg, Russia

**Introduction:**

Prognostic value of malnutrition on postoperative complications and prolonged hospitalization in cardiac population, operated on under cardiopulmonary bypass (CPB) has been previously demonstrated [1]. The impact of malnutrition on long term follow up was the aim of this study.

**Methods:**

A data of 1200 patients previously enrolled in prospective observational study (NCT01366807) was used. Five nutritional screening tools Malnutrition Universal Screening Tool (MUST), Mini Nutritional Assessment (MNA), Nutritional Risk Screening 2002 (NRS-2002) and Short Nutrition Assessment Questionnaire (SNAQ) were used for nutritional screening during initial hospitalization. Follow up data of 747 patients was successfully collected. Among them 8 year follow up was reported in 397 patients and 2 year follow up – in 350 patients. In order to analyze prognostic value of preoperative variables on survival, univariate and multivariate Cox regression analyses and Kaplan-Meyer curve analysis was performed

**Results:**

Nutritional screening tools did not significantly predict mortality. Among total patient cohort mortality was predicted by Euroscore II (HR 1.05; 1.03-1.07 CI; p<0.0001), CPB time (HR 1.003; 1.002-1.005 CI; p<0.0001) and albumin (HR 0.94; 0.92-0.96 CI; p<0.0001) by univariate and multivariate (Euroscore II (HR 1.05; 1.03-1.08 CI; p<0.0001), CPB time (HR 1.006; 1.003-1.009 CI; p=0.0001), albumin (HR 0.95; 0.92-0.98 CI; p=0.0004)) analyses. Despite lack of significance, trend for worse outcomes, associated with malnutrition were found among patients with heart valve diseases (Figure 1).

**Conclusions:**

Among cardiac patients operated on under cardiopulmonary bypass preoperative malnutrition does not significantly affected long-term survival.

**References:**

1. Lomivorotov VV et al. Nutrition 29:436–42, 2013

**Fig. 1 (abstract P268).**
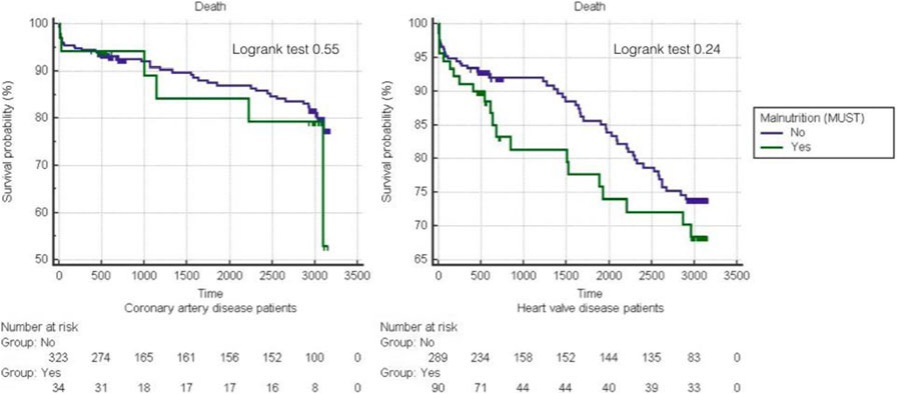
long term survival with accordance to primary cardiac pathology

## P269 The validity of a simple assessment tool for screening sarcopenia in surgical elderly cancer patients

### O Chaiwat^1^, A Siriussawakul^1^, P Pramyothin^2^, C Thanakiattiwibun^1^

#### ^1^Siriraj Hospital, Anesthesiology, Bangkok, Thailand; ^2^Siriraj Hospital, Medicine, Bangkok, Thailand

**Introduction:**

Sarcopenia is defined as the combination of decreased skeletal muscle mass and muscle function. However, muscle mass is assessed by a specific instrument which is costly and immobilization. A simple assessment tool for screening sarcopenia without muscle mass measurement might be practical. Objective: to design and validate the diagnostic performance of a simple assessment tool for screening sarcopenia in elderly cancer surgical patients who schedule to have elective surgery.

**Methods:**

Cancer surgical patients aged > 60 years were enrolled. Nutritional status was evaluated using Mini Nutrition Assessment-Short Form (MNA®-SF).Sarcopenia was assessed according to Asian Working Group for Sarcopenia criteria (AWGS). The validity of four combination formulas used for a diagnosis of sarcopenia compared to AWGS definition was presented.

**Results:**

251 surgical patients were enrolled. 84 patients were diagnosed sarcopenia according to AWGS. The combination of low muscle strength and risk of malnutrition & malnutrition and/or abnormal physical performance (C3) showed the highest sensitivity (81%), specificity (78%) and accuracy (79%) as compared to C1, C2 and C4 combination (table). The PPV and NPV of this combination were 65.4% and 89% respectively. C3 showed a higher diagnostic performance in male than female. A proposed practical algorithm for a diagnosis of sarcopenia started with the measurement of muscle strength, physical performance (gait speed) and assess nutrition status with MNA® -SF. If patients demonstrated low strength & risk of malnutrition and/or low physical performance, sarcopenia is probable. The confirmation process was then performed by muscle mass measurement. If an abnormal muscle mass is presented, sarcopenia is then diagnosed.

**Conclusions:**

The screening of sarcopenia can be performed without the muscle mass measurement. The combination of low muscle strength and risk of malnutrition & malnutrition and/or abnormal physical performance showed the highest diagnostic performance.

**Table 1 (abstract P269).** Combinations used to diagnose sarcopenia

	Muscle mass	Muscle strength	Physical performance	Risk of malnutrition & Malnutrition
AWGS	decrease	decrease	and/or decrease	-
C1	-	decrease	and decrease	
C2	-	decrease	and decrease	and decrease
C3	-	decrease	and/or decrease	and decrease
C4 (and decrease BMI)	-	decrease	and/or decrease	-

## P270

**Withdrawn**

## P271 Enteral glutamine supplementation in critically ill patients with burns

### V Bagin, M Astafyeva, I Korobko, E Elmak, A Pushniak, V Rudnov

#### City Clinical Hospital No 40, ICU, Yekaterinburg, Russia

**Introduction:**

The use of parenteral glutamine is studied in number of RCTs and systemic reviews (Heyland D 2013, Wischmeyer P 2014), while there is a lack of data about the use of enteral glutamine. The aim of our study was to determine the effect of enteral glutamine supplementation on the incidence of hospital infections and death.

**Methods:**

Design: retrospective cohort study. Inclusion criteria: males and females > 18 years of age, TBSA burned 20%-80%, nasogastric intubation.Patients were divided in two groups: glutamine group (n=25) and control group (n=17). In the study group enteral glutamine was administered to the patients for 5 days after admission to the ICU. Baseline characteristics were well balanced between groups. No significant difference was found between groups on patients’ age, sex, TBSA, need for mechanical ventilation and rate of inhalation injury. Primary outcome was all-cause mortality. Secondary outcome was rate of nosocomial infections (skin and skin structure infections (SSSI), lower respiratory tract infections, urinary tract infections, bacteremia, sepsis).

**Results:**

Mortality rate was 6 (24%) and 7 (41%) in the glutamine group and the control group, respectively, p=0.40. Rate of nosocomial infections was 14 (56%) in the glutamine group and 11 (65%) in the control group, respectively, р=0.81. Rates of SSSI, lower respiratory tract infections, urinary tract infections and sepsis did not differ significantly between the groups: 11 (44%) and 6 (35%), p=0.81; 6 (24%) and 7 (41%), р=0.40; 1 (4%) and 1 (6%), р=1.00; 6(24%) and 5 (29%), р=0.97, respectively. Rate of bacteremia was significantly different between the groups: 1 (4%) in the glutamine group and 5 (29%) in the control group, p=0.03. Retrospective design is a significant limitation of our study.

**Conclusions:**

Enteral glutamine supplementation may reduce the incidence of bacteremia in burn patients, but has no influence on the incidence of other nosocomial infections and mortality. Further large clinical trials are needed.

## P272 The effect on ketogenesis of withholding early parenteral nutrition in critically ill children, as a potential mediator of the improved intensive care outcomes

### A De bruyn^1^, J Gunst^2^, C Goossens^2^, G Garcia Guerra^3^, S Verbruggen^4^, K Joosten^4^, L Langouche^2^, G Van den Berghe^2^

#### ^1^KU Leuven, Laboratorium of intensive care medicine, Leuven, Belgium; ^2^KU Leuven, Laboratory of Intensive Care Medicine, Leuven, Belgium; ^3^University of Alberta, Department of Pediatrics, Edmonton, Alberta, Canada, ^4^MC Erasmus, Rotterdam, Netherlands

**Introduction:**

In adults and children, accepting a macronutrient deficit early during critical illness by withholding parenteral nutrition (PN) for 1 week (late PN) accelerates weaning from mechanical ventilation, reduces infections and shortens ICU stay as compared with early supplementing insufficient enteral nutrition with PN (early PN). We hypothesized that these outcome benefits are in part mediated by fasting-induced ketogenesis.

**Methods:**

This is a secondary analysis of the PEPaNIC RCT. First, to identify a potential time point of maximal effect of late vs. early PN, we quantified plasma 3-hydroxybutyrate (3HB) in a matched subset of 96 patients with a PICU stay of ≥5 days. Thereafter, we quantified plasma 3HB and insulin on that “maximal effect” day (or last day for shorter stayers; 1142 patients with available sample). Associations of 3HB with outcomes were assessed with multivariable logistic regression and Cox proportional hazard analyses, adjusted for baseline risk factors and randomization. In sensitivity analyses, models were further adjusted for key regulators of ketogenesis to assess whether any effect was direct or indirect.

**Results:**

Late PN increased plasma 3HB as compared with early PN, with maximal effect on day 2 (P<0.0001 for day 1 to 5 and for the “maximal effect” day in the 1142 patients). Adjusted for baseline risk and randomization, plasma 3HB associated with a higher likelihood of earlier live weaning from mechanical ventilation (P=0.0002) and of earlier live PICU discharge (P=0.004). As plasma 3HB replaced the effect of the randomization, the 3HB effect statistically explained these benefits of the randomization. Further adjustment for key regulators of ketogenesis did not alter these findings. Plasma 3HB did not independently associate with the risk of infections and mortality.

**Conclusions:**

Withholding early PN increased ketogenesis in critically ill children, an effect that statistically mediated part of its clinical benefits.

## P273 Interruptions of enteral nutrition and energy deficits in critically ill patients. Is still a headache for the ICU physicians? A single-center study

### A Kasti^1^, M Tsirindani^2^, V Lepouras^2^, K Katsas^2^, S Fotiou^2^, M Tsakoni^2^, M Lygnos^3^, MP Almyroudi^3^, A Armaganidis^3^, M Theodorakopoulou^4^

#### ^1^Department of Nutrition and Dietetics, Attikon University Hospital, Department of Nutrition and Dietetics, Attikon University Hospital, Athens, Greece; ^2^Department of Nutrition and Dietetics, Attikon University Hospital, Athens, Greece; ^3^2nd ICU Department, Attikon University Hospital, Athens, Greece; ^4^2nd ICU Department, Attikon University Hospital, Intensive Care Medicine, Athens, Greece

**Introduction:**

Critical care patients are prone to frequent feeding interruptions for various reasons including feeding intolerance. These interruptions can lead to adverse outcomes. The aim of the study was to determine the reasons for and the duration of interruptions of enteral nutrition (EN).

**Methods:**

Single-center observational, cross-sectional study in a 19-bed mixed ICU of a tertiary hospital. Duration: 6 months. 50 patients, aged 65.4 years old (±14.6), that stayed in the ICU > 48 hrs and were fed with EN were included. Anthropometric data, BMI, time of initiation of prescribed EN, type of EN formula, daily calories delivered were recorded. Energy intake was calculated according to ESPEN guidelines (25 Kcal/ kg BW/day). The causes for and duration of interruption were reviewed from the patient’s chart. APACHE II and mNutric score was calculated for all patients. mNutric score ≤5 was used to diagnose malnutrition.

**Results:**

All patients included in the study were endotracheally intubated. APACHE II was 22.4 ±5.6. 58% of patients had increased risk of malnutrition. ICU stay was 24.4 (8.0±32.0) days, and the in-hospital mortality was 24%. There were 318 episodes of EN interruptions over a median ICU stay of 24.4 days. Median 2.5 interruptions/patient. The most common reason for EN interruption was gastric residual volume monitoring followed by diagnostic and therapeutic procedures (Figure 1). Other reasons include surgery, intolerance and/or delayed feeding and extubation. The median lost feeding time was 5.4 hours/day (3.7-7.4) for all causes, while the mean loss of total energy intake was 790 kcal/day (±321)/day. Average body weight of the patients was 78 Kg (±12). Caloric deficit was calculated at 1950 Kcal/day or 40% of the prescribed caloric goal.

**Conclusions:**

The results of this study showed that interruptions can lead to substantial caloric deficit, malnutrition and adverse events. An interruption-minimizing protocol could be useful in order to reduce the missing hours and to improve the clinical outcomes.

**Fig. 1 (abstract P273).**
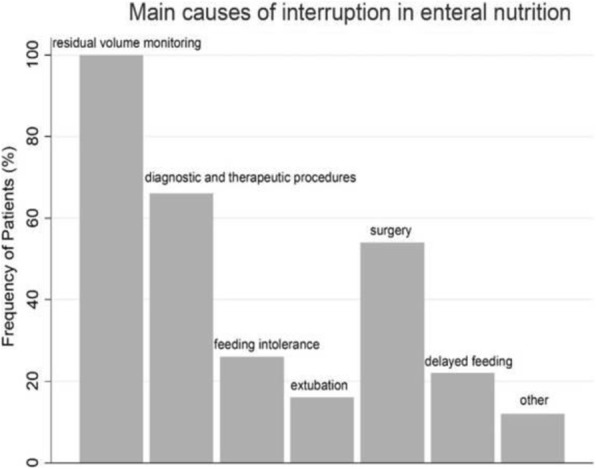
Main causes of interruption of enteral nutrition

## P274 Relationship of goal-directed nutritional adequacy with clinical outcomes in critically ill patients

### PC Tah^1^, ZY Lee^1^, BK Poh^2^, HA Abdul Majid^3^, VR Hakumat-Rai^4^, MB Mat Nor^5^, CC Kee^6^, M Kamarul Zaman^7^, MS Hasan^1^

#### ^1^University of Malaya, Department of Anesthesiology, Faculty of Medicine, Kuala Lumpur, Malaysia; ^2^Universiti Kebangsaan Malaysia, Nutritional Sciences Programme & Centre for Community Health, Faculty of Health Sciences, Kuala Lumpur, Malaysia; ^3^University of Malaya, Department of Social and Preventive Medicine, Faculty of Medicine, Kuala Lumpur, Malaysia; ^4^KPJ Tawakkal Specialist Hospital, Kuala Lumpur, Malaysia; ^5^International Islamic University Malaysia, Department of Anesthesiology, Pahang,, Malaysia; ^6^National Institutes of Health, Sector for Biostatistics & Data Repository, Research Policy & Planning Division, Selangor,, Malaysia; ^7^Universiti Teknologi MARA, Centre of Nutrition and Dietetics Studies, Faculty of Health Sciences, Selangor, Malaysia

**Introduction:**

There are controversies surrounding the effects of optimal nutritional intake on clinical outcomes in critically ill patients. This study aimed at investigating the relationship of goal-directed energy and protein adequacy on clinical outcomes which includes mortality, intensive care unit(ICU) and hospital length of stay (LOS), and length of mechanical ventilation (LOMV).

**Methods:**

This was a single centre prospective observational study. Nutritional requirements were guided by Indirect Calorimetry and 24-h urinary urea.Nutritional intake was recorded daily until death, discharge, or until day 14 of ICU stay. Clinical outcomes were collected from patient’s hospital record. The relationship between the two groups (<80% and ≥80% of overall nutritional requirement) with mortality outcomes was examined by using logistic regression with adjustment for potential confounders.

**Results:**

Data were collected from 314 critically ill patients (age, 53.6±18.9 years; 63.4% male; body mass index, 25.7±7.2 kg/m ; APACHE II score,23.5±6.2). Patients in the ≥80% of nutritional requirement group (n=207) had a higher mean energy adequacy (97.9±12.3 vs. 65.4±14.7 %,p<0.001) and protein adequacy (96.8±21.2 vs. 67.3±24.1 %, p<0.001). Overall, no significant difference between <80% or ≥80% of energy adequacy in mortality outcomes in univariate and multivariate analysis. Energy adequacy was not associated with 60-day mortality when adjusted for protein adequacy (Adj OR 0.668; 95% CI, 0.332 – 1.343; p=0.257). However, energy adequacy of ≥80% signicantly increased ICU LOS (p<0.001), hospital LOS (p=0.02) and LOMV (p<0.001).

**Conclusions:**

Goal-directed energy and protein adequacy of ≥80% associated with increased ICU LOS, hospital LOS and LOMV, but not associated with mortality outcomes. Based on this result, energy provision should be conservative during the acute phase of critical illness. The optimal protein dose requires further investigation.

## P275 Terlipressin-induced hyponatremia in acute gastrointestinal bleeding due to portal hypertension

### CC Castelo Branco, RA Alves, AP Pinto

#### Centro Hospitalar e Universitário do Porto, Anesthesiology, Emergency and Critical Care, Porto, Portugal

**Introduction:**

Terlipressin, despite being one of the main treatments for acute variceal bleeding, may lead to severe hyponatremia due to its antidiuretic activity.We aimed to identify risk factors for development of hyponatremia during terlipressin treatment.

**Methods:**

Retrospective study of patients admitted to Acute Intermediate Care Unit for hypertensive upper gastrointestinal bleeding due to chronic liver disease who received terlipressin(december2011-december2018).Hyponatremia was defined as a decrease in Na serum levels ≥5mEq and severe hyponatremia as >10mEq within 3 days of treatment.

**Results:**

We studied 191 patients, 84.3% male, mean age of 58.6 years (SD 10.8). Alcohol-related liver disease was the most frequent etiology. Hyponatremia occurred in 53 patients (27.7%). Serum Na ∆between -5 and -10mEq and serum Na ∆>-10mEq occurred in 20.4 and 7.3%, respectively (Table 1). Severe hyponatremia occurred in 11 patients (5.8%) and symptoms were reported in two cases (status epilepticus and altered mental status). Patients with higher baseline levels of Na were more susceptible to terlipressin-induced hyponatremia and a longer length of stay was observed in patients with serum Na∆>-10 mEq (6.3 vs 4.4 days, p<0.022).

**Conclusions:**

The prevalence of hyponatremia in our study was lower than previously reported.Higher serum Na at admission and AIH as etiology of cirrhosis were predictors of terlipressin-induced hyponatremia. Neither the cumulative dose of terlipressin nor the duration of treatment appear to be related to the development of hyponatremia

**Table 1 (abstract P275).** Results

	Na ∆≤-5 mEq	5 >Na ∆≤ -10 mEq	Na ∆>-10 mEq	p
Patients, n(%)	138(72.3)	39(20.4)	14(7.3)	
Length of stay (days), mean(SD)	4.4(2.3)	4.5(2.7)	6.3(2.9)	0.022
Autoimmune hepatitis (AIH), n(%)	6(4.3)	0	3(21.4)	0.013
Terlipressine cumulative dose (mg), mean(SD)	33.0(17.4)	37.8(16.6)	39.3(14.7)	0.169
Terlipressine duration (days), mean(SD)	3.3(1.6)	3.8(1.6)	3.6(1.0)	0.124
Serum Na admission (mmol/L), mean(SD)	136.9(4.5)	140.1(4.7)	141.8(3.4)	<0.001
Mortality, n(%)	4(2.9)	0	1(7.1)	0.237

## P276 An increase in serum sodium predicts mortality in ICU patients

### CC Grim^1^, F Termorshuizen^2^, HJ Helmerhorst^1^, NF De Keizer^2^, E De Jonge^1^

#### ^1^Leiden University Medical Center (LUMC), Intensive Care, Leiden, Netherlands; ^2^Amsterdam University Medical Centre, Medical Informatics, Amsterdam, Netherlands

**Introduction:**

It is unknown whether the established association of hyponatremia with mortality is causal and whether correction of mild hyponatremia would improve survival. Our objective was to assess the independent association of change in serum sodium in the first 48 hours after ICU admission with hospital mortality.

**Methods:**

Multicenter cohort study in ten Dutch ICUs between January 2011 and April 2017. Inclusion criteria: patients with at least one serum sodium measurement within 24 hours of ICU-admission [Na_1_] and at least one serum sodium measurement 24-48 hours after ICU admission [Na_24-48h_]. A Cox proportional hazard model adjusted for age, gender, and APACHE-IV score was used to assess the association between ∆48h-[Na] (mean-[Na_24-48h_]-[Na_1_]) and hospital mortality.

**Results:**

In total, 36,660 patients were included for analysis. Patients with severe hyponatremia (<125 mmol/L) and hypernatremia (>145 mmol/L) at the time of ICU admission had a higher risk of mortality. For mild hyponatremia, normonatremia, and hypernatremia at ICU admission, a ∆48h-[Na] >5 mmol/L was associated with larger hazards of mortality (Figure 1).

**Conclusions:**

An increase in serum sodium in the first 48 hours of ICU admission is independently associated with a higher mortality in patients admitted with mild hyponatremia, normonatremia, and hypernatremia. Based on our findings, it is possible that mild hyponatremia may be a protective mechanism in critical illness, which questions common practice of routinely correcting serum sodium when it is too low.

**Fig. 1 (abstract P276).**
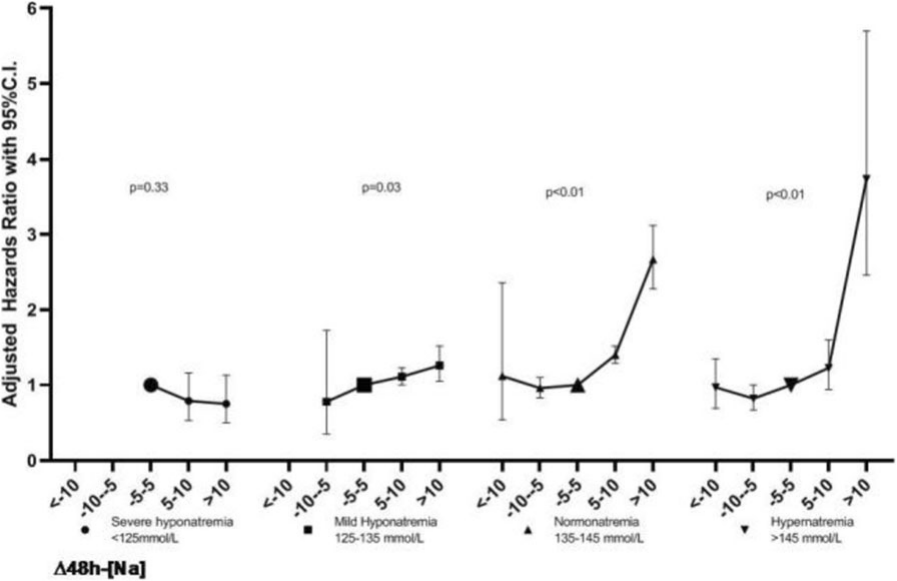
Association between in-hospital mortality and ∆48h-[Na] according to first serum sodium measurement at ICU admission

## P277 Variceal bleeding in cirrhotic patients: factors influencing poor outcome. a single-center serie

### A Ramos^1^, E Pizzo^1^, A Dogliotti^2^, D Latasa^1^, M Perezlindo^1^, S Ferretti^3^, F Acharta^1^, C Lovesio^1^

#### ^1^Sanatorio Parque, Critical Care, Rosario, Argentina; ^2^Grupo Oroño, Epidemiology and statistics, Rosario, Argentina; ^3^Sanatorio Parque, Hepatology, Rosario, Argentina

**Introduction:**

Variceal bleeding is one of the major causes of death in cirrhotic patients. We analyzed our database to identify factors determining poor outcome: the composite of in-hospital death from any cause and rebleeding after days 5 and 45.

**Methods:**

During the period 2007-2019, 87 patients with variceal bleeding were treated in our institution. On admission data including patient characteristics and laboratory findings were assessed and further analyzed. A multivariate analysis was performed to identify predictors of poor outcome.

**Results:**

Twelve patients died (10.4%) and 24 (20.8%) patients had a poor outcome. Integrated multivariate model: albumin (p=0.14), active alcoholism (p=0.09), portal thrombosis (p=0.01), mechanical ventilation assistance (p=0.24), creatinine (p=0.04), base excess (p=0.09), encephalopathy (p=0.07), heart rate (p=0.99), white blood cells (p=0.05), MELD (p=0.08), mean arterial pressure (p=0.04), platelets (p=0.48), prothrombin time (p=0.72) and gastric varices (p=0.01). In the multivariate model the presence of portal thrombosis, elevated creatinine, diminished mean arterial pressure and gastric varices were independent associated with poor outcome in cirrhotic patients admitted for variceal bleeding. (AUC ROC 0.90; 95% IC: 0.82-0.95).

**Conclusions:**

The presence of portal thrombosis, elevated creatinine, diminished mean arterial pressure and gastric varices were independent associated with poor outcome in cirrhotic patients admitted for variceal bleeding.

## P278 Impact of acute liver failure on bleeding risk in critical care settings; a multi-center retrospective analysis

### Y Yasuda^1^, D Kasugai^2^, T Yamamoto^2^, M Ozaki^2^, N Matsuda^2^

#### ^1^Nagoya University Graduate School of Medicine, Department of Emergency and Critical Care Medicine, Nagoya, Aichi, Japan; ^2^Nagoya University Graduate School of Medicine, Nagoya, Aichi, Japan

**Introduction:**

While acute liver failure (ALF) develop coagulopathy, the risk of bleeding in ALF is controversial [1, 2]. We aimed to evaluate the bleeding risk of ALF among critically ill patients.

**Methods:**

We analyzed 200,859 critically ill patients from multi-center database who admitted to 335 Intensive care units (ICUs) in the United States between 2014 and 2015, which is monitored by eICU Programs. Patients without diagnosis of trauma and bleeding on admission were included. Primary outcome was all hemorrhage occurred during ICU stay. Logistic regression analysis was performed to evaluate the risk of bleeding in ICU.

**Results:**

Of 153,691 patients included in this study, 428 were ALF. Bleeding events were occurred in 1,817 (1.18%) patients. Intracranial hemorrhage (ICH) were occurred in 488 (0.32%), and gastrointestinal (GI) bleeding were in 827 (0.54%) cases. On multivariable Logistic regression analysis, variables significantly associated with all hemorrhage were ALF (Odds ratio[OR] 4.13; 95% confidence interval [CI]:2.52-6.77; p<0.0001), older age, African American, Hispanic, and Native American ethnicity, cardiac arrest, stroke, sepsis, mechanical ventilation, and history of GI bleeding. ALF were also associated with intracranial hemorrhage (OR:8.54; 95%CI:3.09-23.6, p<0.0001), and GI bleeding (OR:5.25; 95%CI:3.03-9.08; p<0.0001 ). ALF significantly strengthen magnitude of effect on all hemorrhage in mechanically ventilated patients (p=0.003) and sepsis (p=0.08).

**Conclusions:**

ALF is associated with all type of hemorrhage, including ICH and GI bleeding in critical care settings. ALF showed synergy effect on bleeding in patient with mechanical ventilation and sepsis.

**References:**

1. Wendon J et al. J Hepatol 66:1047-1081, 2017

2. Stravitz RT et al. Hepatology 67:1931-1942, 2018

## P279 Potential benefits of hemoadsorption in patients with acute liver failure

### M Popescu^1^, A Vasile^2^, A Tanase^2^, A Dinca^2^, C David^2^, D Tomescu^2^

#### ^1^Fundeni Clinical Institute, Anaesthesia and Critical Care, Bucharest, Romania; ^2^Fundeni Clinical Institute, Bucharest, Romania

**Introduction:**

Acute liver failure (ALF) represents a life-threatening organ dysfunction associated with increased mortality and liver transplantation represents the only definitive treatment. The aim of this study was to assess the effects of renal replacement therapy in combination with hemoadsorption in ALF patients.

**Methods:**

Twenty-nine patients with ALF admitted to the intensive care unit (ICU) of Fundeni Clinical Institute were included in the study. After ICU admission, 3 consecutive session of hemoadsorption in combination with continuous veno-venous hemodiafiltration were applied. Number of organ dysfunctions and SIRS criteria were recorded at ICU admission. The following data were recorded before and after the 3 hemoadsorption therapies: Glasgow coma scale, PaO2/FiO2, creatinine, 24-hours urine output, bilirubin, leucocyte and platelet count, heart rate, mean arterial pressure and vasopressor support, C-reactive protein and procalcitonine. CLIF-SOFA score was calculated before and after the therapy. ICU length of stay and 28-days outcome were noted.

**Results:**

The mean age in the study group was 34±14 years. The median number of SIRS criteria was 3 [2,4] and the median number of organ dysfunctions was 3 [1,6]. The use of hemoadsorption was associated with a decrease in creatinine (from 1.9±1.4 to 1.2±0.8 mg/dL, p=0.02), bilirubin (from 14.2±12.6 to 9.2±9.1 mg/dL, p=0.05) and platelet count (96482±70913 / uL to 51275±24393 /uL, p=0.01). We also observed a decrease in Clif-SOFA score from 12.0±2.1 to 10.0±2.6 (p=0.05). Overall mortality was 37.9% (n=11). Six patients (20.7%) underwent liver transplantation with 100% 28-days survival.

**Conclusions:**

The use of hemoadsorption in patients with ALF is associated with improvement in liver and kidney functional tests and may represent a new therapy in bridging these patients to liver transplantation.

## P280 Preliminary study on the relationship between in vitro lactic acid bacteria, intestinal microecology and sepsis

### CX Wei, JY Li, XZ Wang

#### Fujian Medical University Union Hospital, Department of Gastroenterology, Fuzhou, China

**Introduction:**

Impairment of intestinal mucosal barrier function is the initiating factor of sepsis. In order to explore the effect of lactic acid bacteria on intestinal barrier function impaired by sepsis, it is necessary to establish sepsis and lactic acid bacteria ecological models. However, how to construct these models is still unclear.

**Methods:**

Co-cultures with a gradient of lactic acid bacteria and Caco-2 cells were constructed. The symbiotic state was observed under an inverted microscope and lactate dehydrogenase (LDH) toxicity tests, transepithelial electrical resistance(TEER) tests and Western blots were used to determine effective concentrations of lactic acid bacteria in monolayer cell models. Lipopolysaccharide (LPS) was used to treat cells, and Cell Counting Kit-8, quantitative reverse transcription PCR(RT-qPCR) and enzyme linked immunosorbent assays (ELISA) were used to determine the appropriate concentration for sepsis models.

**Results:**

The number of living cells decreased significantly when the MOI(number of lactic acid bacteria/cell number) reached 8 (Figure 1, panels 1a, b). The release of LDH indicated that damage to cells began to increase when the MOI exceeded 10 (panels 2a, b). At an MOI of 0.5, resistance values began to increase over time, whereas resistance values began to decrease when the MOI reached 10 (panel 3). As the number of lactobacilli increased, the expression of tight junction protein increased and then decreased (panel 4a, b, c). In sepsis model experiments, the cell survival rate began to decrease once the concentration of LPS exceeded 10^4 ng/ml (panel 5). RT-qPCR results showed that 10^2^ ng/ml LPS significantly increased inflammatory cytokines (panel 6), and ELISA results consistently showed that TNF-α and IL-6 increased significantly when LPS concentrations reached 10^2^ ng/ml (panel 7a, b).

**Conclusions:**

It is feasible to construct a cell monolayer model of lactic acid bacteria and LPS. The appropriate MOI of lactic acid bacteria is 0.5 and the optimal concentration of LPS is 10^2^ ng/ml.

**Fig. 1 (abstract P280).**
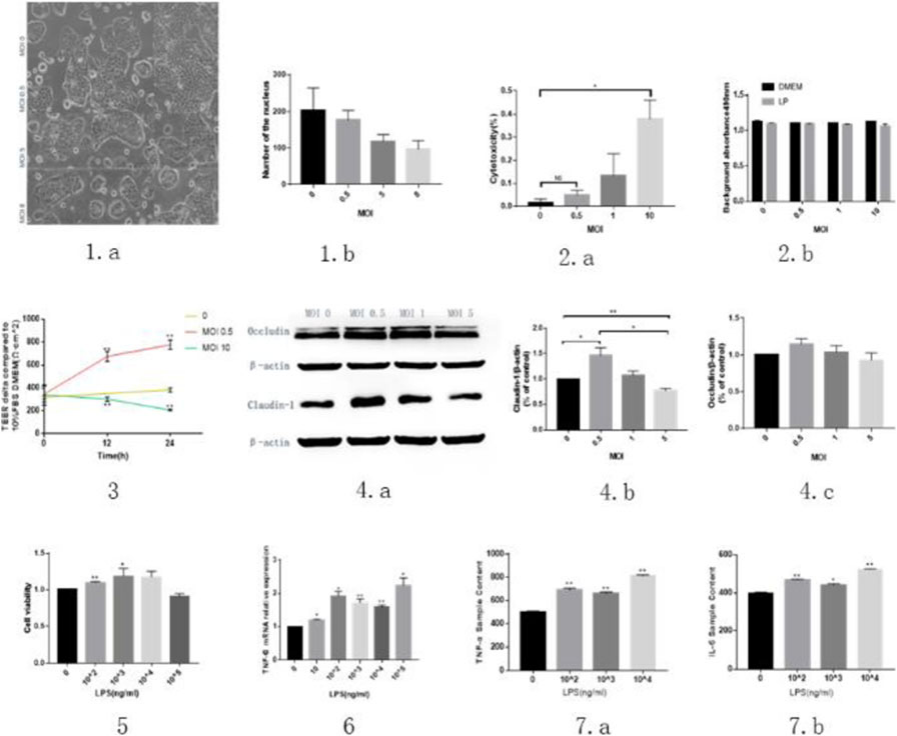
Results

## P281 The effects of vitamin D level on sepsis severity and risk of hospitalization in emergency patients

### M Romposra^1^, K Trongtrakul^2^, A Vaniichkulbodee^1^

#### ^1^Faculty of Medicine Vajira Hospital, Navamindradhiraj University, Emergency Medicien, Bangkok, Thailand; ^2^Faculty of Medicine Vajira Hospital, Navamindradhiraj University, Internal Medicine, Bangkok, Thailand

**Introduction:**

Vitamin D is not only important for calcium and phosphate homeostasis, but it also has a role in sepsis regulation. Many previous studies reported a high prevalence of vitamin D insufficiency in critically ill patients; however, data about vitamin D status are limited in adult patients with sepsis who visited an emergency department (ED). Therefore, we decided to evaluate the association of 25-hydroxyvitamin D (25-OH vit D) level on sepsis severity and risk of hospitalization in the adult septic patient who visited ED.

**Methods:**

Cross-sectional study was conducted in the ED of the Faculty of Medicine Vajira Hospital, Navamindradhiraj University, Bangkok, Thailand, from 1 March to 30 September 2015. Patients age ≥ 18 who diagnosed with sepsis were enrolled. 25-OH vit D level was analyzed correlated with illness severity of sepsis measured by APACHE-II and MEDS score using linear regression analysis. And the risk of hospitalization was analyzed using logistic regression analysis.

**Results:**

One hundred and one patients were enrolled. On average, there were 67.9±18.2 years old, 56% (n=57) were female, APACHE-II score = 14.2±6.3, MEDS score = 7.9±4.6, and 25-OH vit D level = 18.5±11.3 ng/mL. The admission rate in our cohort was 88% (n=89). Significant correlations between 25-OH vit D level and sepsis severity scores were found, which measured by APACHE-II and MEDS score (coef -0.12; 95%CI, -0.22 to -0.006, p -0.04 and – 0.10; 95%CI, -0.17 to -0.02, p = 0.02, respectively). However; 25-OH vit D level could not predict hospitalization in our septic patients (OR = 0.98; 95%CI, 0.93-1.03; p=0.33), while APACHE-II and MEDS score had an influence on patient hospitalization (OR = 1.51; 95%CI, 1.21-1.89; p<0.001 and OR = 1.56; 95%CI, 1.23-1.97, p<0.001; respectively).

**Conclusions:**

25-OH vit D level had a significant negative association with sepsis severity evaluated by APACHE-II and MEDS score. However, it cannot predict hospitalization as the same as APACHE-II and MEDS scoring system.

## P282 A new and accurate low-cost printable pH sensor tested in a preclinical model for detecting changes in metabolic acidosis

### Z Tehrani^1^, S Whelan^1^, M Lawrence^2^, S Pillai^2^, A Evans^2^, M Peacock^3^, D Gethin^4^, O Guy^5^

#### ^1^Centre for Nano Health, College of Engineering, Swansea University,, Swansea, United Kingdom; ^2^Welsh Centre for Emergency Medicine Research Morriston Hospital Swansea Bay University Health Board, Swansea, United Kingdom; ^3^Zimmer and Peacock Ltd - 1 Market Hill, Royston, SG8 9JL, United Kingdom; ^4^4Welsh Centre for Printing and coating, College of Engineering, Swansea University, Swansea SA18EN, United Kingdom; ^5^Department of Chemistry, College of Science, Swansea University, Swansea SA2 8PP, UK, United Kingdom

**Introduction:**

Sepsis is associated with high mortality and morbidity. As the severity increases, physiological parameters such as pH changes are one of the most notable features in metabolic acidosis secondary to high lactate. Currently there is no point of care test other than blood gas measurement that could detect these pH changes. This is challenging especially in prehospital environment. The aim of this study is to develop a novel rapid point of care testing using a sensor to detect pH change in blood.

**Methods:**

Sensors were produced by screen printing graphene and silver electrodes and functionalizing the graphene working electrode with an active layer of melanin. A preclinical sensor model was produced by adding lactic acid to a citrated plasma sample thus altering its pH over a clinically relevant range. The pH sensors were exposed to modified plasma, recording any changes in the voltage. The relationship between the voltage potential and plasma pH was established using weighted least squares regression.

**Results:**

A pH dependent change in the measured voltage, with respect to the pH of the solution, was observed with a sensitivity of -111.27 mV/pH +/- 15.95 over a physiologically relevant pH range between pH7.1 and pH7.6.

**Conclusions:**

In this first phase proof of concept study a low cost, pH sensor was fabricated and demonstrated to be effective in measuring the pH of the plasma. This is the first time that such a sensor has been demonstrated and validated to work in this preclinical model of acidosis. The technology demonstrated here is a promising candidate for a point of care test whereby abnormal blood pH levels can be detected and monitored outside of a laboratory environment in a rapid manner. Further studies are now underway to detect this change in whole blood.

## P283 Feasibility of new hand-held near-infrared spectroscopy (NIRS) monitor during prehospital anaesthesia

### J Nurmi^1^, P Laukkanen-Nevala^1^, H Kirves^2^, L Raatiniemi^3^, M Tommila^4^, H Piiroinen^5^, P Karhivuori^6^, S Tukia^7^, T Toivonen^1^, A Olkinuora^1^

#### ^1^FinnHEMS, Research and Development Unit, Vantaa, Finland; ^2^Helsinki University Hospital and Univeristy of Helsinki, Emergency Medicine and Services, FinnHEMS 10, Helsinki, Finland; ^3^Oulu University Hospital, Centre for Prehospital Emergency Care, Oulu, Finland; ^4^Turku University Hospital, Division of Perioperative Services, Intensive Care and Pain Medicine, Turku, Finland; ^5^Tampere University Hospital, Emergency Medical Services, Tampere, Finland; ^6^Kuopio University Hospital, Centre for Prehospital Emergency Care, Kuopio, Finland; ^7^Lapland hospital district, Lapland HEMS unit, Rovaniemi, Finland

**Introduction:**

Many patients anaesthetized in prehospital setting are at risk of inadequate cerebral oxygenation. New hand-held oximeter enables monitoring of near-infrared spectroscopy (NIRS) in prehospital setting. We aimed to assess the feasibility of the new device during prehospital anaesthesia.

**Methods:**

We performed a prospective, observational pilot study in two anaesthesiologist-staffed Helicopter Emergency Medical Services (HEMS) units. Adult patients who underwent rapid sequence intubation and prehospital anaesthesia because of any reason were included by the HEMS team. NIRS monitoring of left frontal cerebral regional oxygen saturation (rSO_2_) with Nonin H500 oximeter system was performed throughout the prehospital anaesthesia. The rSO_2_records were reviewed in terms to identify potentially artefact values caused e.g. moving or transporting the patient.

**Results:**

Total of 97 patients were enrolled in the study. Due to technical problems or user error adequate monitoring was not achieved in 13 (13%, 95% confidence interval 8 to 22%) patients. Software update of the monitor was installed during the study and no monitoring failures occurred afterwards. No difference was observed in the on-scene-time during the study period compared to eligible patients treated in four previous years. Proportion of likely artefact values was 5.5% in total (median 0.99%, Q1-Q3 0,25%-6.18%).

**Conclusions:**

Monitoring cerebral oxygenation with Nonin H500 hand-held oximeter system during prehospital anaesthesia is feasible. The value of this new modality available for prehospital critical care providers needs to be further evaluated.

## P284 Risk-adjusted management strategies for patients admitted with acute pulmonary embolism to a teaching hospital in the UK

### T Owen, N Bunker

#### Royal London Hospital, Critical Care Unit, London, United Kingdom

**Introduction:**

Risk stratification of patients with acute PE helps to determine the most appropriate treatment. The European Society of Cardiology proposes the use of PESI (Pulmonary Embolism Severity Index) class to determine when reperfusion therapies (full/partial dose thrombolysis, catheter directed local thrombolysis, ECMO, unfractionated heparin or LMWH) are most appropriate [1]. Our study aimed to determine the number of Patients eligible for reperfusion strategies and inform the likely impact on High Dependency services.

**Methods:**

We performed a retrospective analysis of all patients admitted to the Royal London Hospital from 1^st^ April 2018 through 31^st^ March 2019 with acute pulmonary embolism. Data was obtained on demographics, suspected cause and PESI score on day of admission.

**Results:**

Over 1 year 165 people were diagnosed with acute PE. The majority (85%, n=140) were diagnosed with CTPA. The mean age was 55.6 years old (median 55). Right ventricular (RV) strain pattern was present in 10% (n=16). The majority of PE’s were unprovoked (39%), other causes included immobility (33%), cancer (17%), deranged clotting (10%) and pregnancy (1%). 45 (27%) patients were PESI III/IV of these 7 (4%) had both echocardiographic evidence of right ventricular strain and elevated troponin values. 13 (8%) Patients had died at time of data collection with the majority of deaths (n=6) within the PESI V class (Figure 1).

**Conclusions:**

Over one year only a small proportion of patients (n=7, 4%) were classified as ‘Intermediate High’ risk and potential candidates for reperfusion therapies. The average age of PESI class 3 or 4 Patients was 74.8 years (median=78). Extremes of age skew the PESI score disproportionately and a number of ‘Intermediate High’ risk patients would likely not be clinically suitable for reperfusion therapies. Adoption of the ESC’s proposed treatment algorithm would therefore likely place minimal additional burden on HDU services.

**References:**

1. Konstantinides SV et al. Eur Heart J. 2019 Aug 31. doi: 10.1093/eurheartj/ehz405.

**Fig. 1 (abstract P284).**
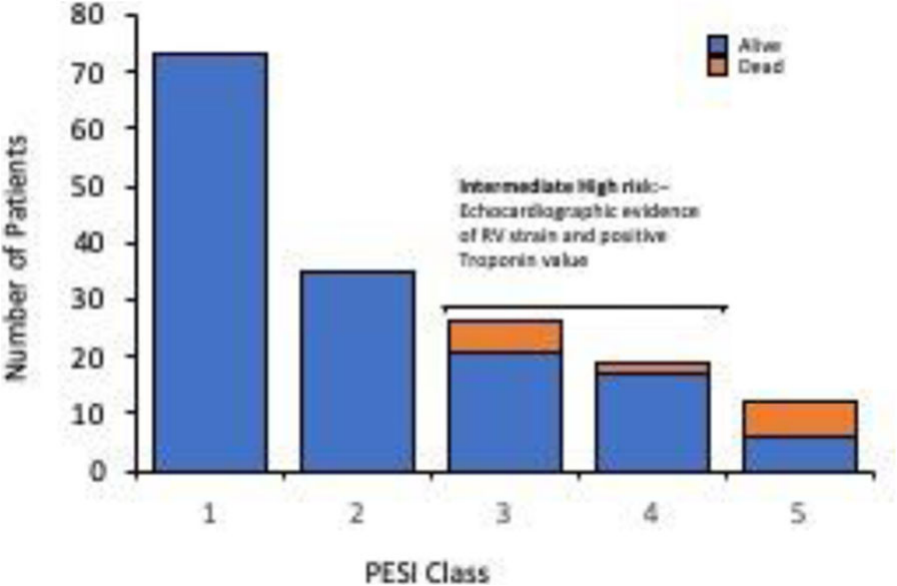
PESI scores for patients presenting to a central London teaching hospital with acute PE over the course of a year

## P285 Serum vitamin C levels were decreased from patients with severe emergency patients: an electron spin resonance study

### R Takenaka^1^, S Matsumoto^2^, S Fujimoto^1^, T Himeno^1^, S Nureki^3^, K Ishii^1^, T Sakamoto^4^, O Shigemitsu^1^

#### ^1^Oita University Hospital, Department of Emergency Medicine, Yufu city, Oita, Japan; ^2^Oita University Hospital, Department of Anaesthesiology and Intensive Care Medicine, Yufu city, Oita, Japan; ^3^Oita University Hospital, Department of Respiratory Medicine and Infectious Diseases, Yufu city, Oita, Japan; ^4^Oita University Hospital, Advanced Trauma, Emergency and Critical Care Center, Yufu city, Oita, Japan

**Introduction:**

Vitamin C (VC) plays an important role as anti-oxidant agent which could not be synthesized *in vivo*. The lack of VC results in excessive oxidant leading to multiple organ failure. Serum levels of VC radical/DMSO (VCR/DMSO) could be measured using electron spin resonance apparatus. Serum VCR/DMSO is previously reported to mean serum ascorbic acid concentrations [1]. We hypothesized that patients with severe damage may lack in VC. The aim of this study is to measure the serum VC concentrations from patient with severe damage who have admitted to emergency room.

**Methods:**

One hundred patients who have admitted to emergency and critical care center in Oita University Faculty of Medicine and fifteen healthy volunteers were enrolled in this study after obtaining written informed consent. Serum VCR/DMSO concentrations were measured using electron spin resonance apparatus (JES-FR30, JEOL Ltd, Japan) on and after admission. The results were expressed as average ± SEM. Intergroup comparisons were done using Student’s t-test or Mann-Whitney U Test. A p<0.05 was considered statistically significant.

**Results:**

Serum levels of VCR/DMSO were significantly lower in admitted patients (n=100, 0.9345±0.0524) compared with healthy volunteers (n=15, 0.2784±0.0143). The details of admitted patients were as follows; trauma (n=69, 0.2979±0.0159), post cardiac arrest syndrome (n=21; 0.2151±0.0272), sepsis (n=2; 0.1229±0.0131), acute respiratory distress syndrome(n=2; 0.3575±0.3089), stroke (n=3; 0.1833±0.0952), poisoning (n=2; 0.2312±0.0600), gastric hemorrhage (n=1; 0.12235). Serum VCR/DMSO levels decreased after admission (day 2; 0.2180±0.0151, day 3; 0.1777±0.0152).

**Conclusions:**

Serum VCR/DMSO measurements using the ESR spectrometer is clinically very useful to enable real-time monitoring of serum vitamin C concentrations. Our results showed that serum levels of VC were significantly decreased on admission and over time in severe emergency patients.

**References:**

1. Matsumoto S et al. J Trauma 68:796-801, 2010

## P286 The revised national early warning score (NEWS) with modified Glasgow PROGNOSTIC SCORE (mGPS) is superior to the NEWS for predicting in-hospital mortality in elderly emergency patients

### T Mitsunaga

#### Jikei university school of medicine, Emergency Medicine, Tokyo, Japan

**Introduction:**

The National Early Warning Score (NEWS) was developed in the UKto identify the risk of death. The previous study showed that the modified Glasgow Prognostic Score (mGPS) correlate with frailty in elderly patients [1]. The aim of this study is to evaluate the predict value of the revised NEWS with mGPS for in-hospital mortality (in 28 days) in elderly emergency patients.

**Methods:**

This study is secondary analysis and was carried out in Jikei University Kashiwa Hospital, in Japan, from 1 April 2017 to 31 March 2018. The acute medical patients aged 65 and older were included. The NEWS was derived from seven physiological vital signs. The mGPS was derived from C-reactive protein (CRP) and Albumin. Discrimination was assessed by plotting the receiver operating characteristics (ROC) curve and calculating the area under the ROC curve (AUC).

**Results:**

The AUCs for predicting in 28 days in-hospital mortality were 0.818 for revised NEWS with mGPS and 0.797 for the original NEWS. The AUC of the revised NEWS with mGPS was significantly higher than that of the original NEWS for predicting in-hospital mortality (p < 0.001) (Figure 1).

**Conclusions:**

Our single-centred study has demonstrated the utility of the revised NEWS with mGPS as a high predictor of acute phase in-hospital mortality in elderly emergency patients.

**References:**

1. Viviane L et al. J Geriatr Oncol 6:479-483, 2015.

**Fig. 1 (abstract P286).**
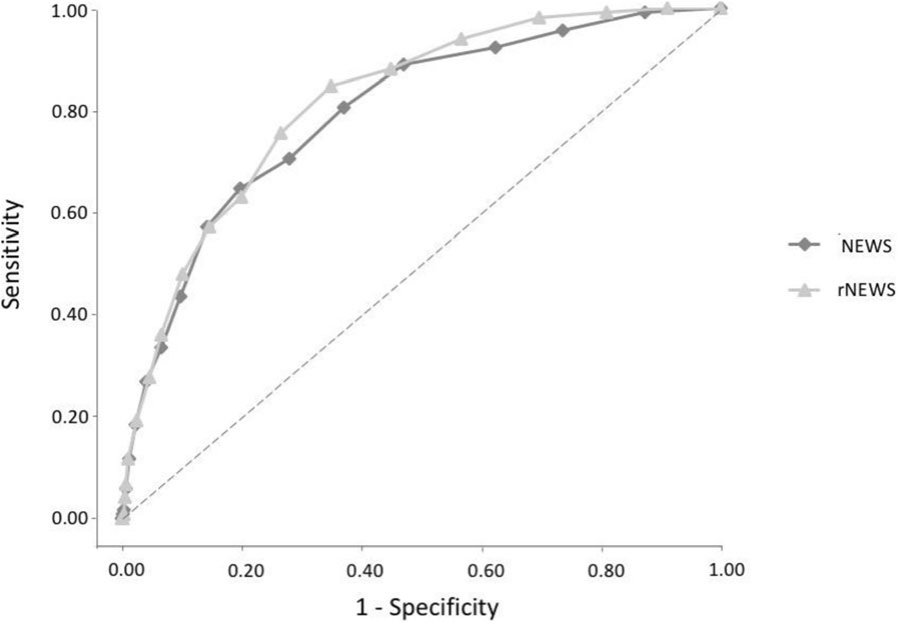
Receiver operator characteristic (ROC) curves for in-hospital mortality comparing the revised National Early Warning Score (rNEWS) with modified Glasgow Prognostic Score (mGPS) and the National Early Warning Score (NEWS) in the emergency department

## P287 Combination of ECG and cardiovascular risks factors to increase the diagnostic performance of chest pain triage at emergency department. CECIDOC study, preliminary results

### AS Pirot^1^, D Castanares Zapatero^2^, M Dechamps^3^

#### ^1^Cliniques Universitaires Saint-Luc, Université Catholique de Louvain, Emergency Department, Brussels, Belgium; ^2^Cliniques Universitaires Saint-Luc, Université Catholique de Louvain, Intensive Care Unit, Brussels, Belgium; ^3^Cliniques Universitaires Saint-Luc, Université Catholique de Louvain, Cardiovascular Intensive Care Unit, Brussels, Belgium

**Introduction:**

The diagnostic performance of the five main emergency department (ED) triage systems has been shown to be poor in distinguishing acute coronary syndromes (ACS) from mild severity diseases in chest pain patients. These ED triage systems are either clinically-based, being more sensitive or ECG-based, more specific [1]. The goal of the study was to evaluate if incorporation of cardiovascular risk factors (CVRF) into ECG-based triage could increase his diagnostic performance.

**Methods:**

CECIDOC is a prospective, observational, single-center study in an academic Hospital. All consecutive adult patients admitted for acute chest pain were included. We compared the ECG-based FRENCH triage system [2] to a modified system upgrading patients with a normal ECG but significant cardiovascular risk from a low acuity triage score (waiting period before medical assessment of max. 60 min.) to a high acuity triage score (waiting period before medical assessment of max. 20 min.). The final diagnosis was determined after a 30-day follow-up. We predefined as being adequate a high-acuity triage score (level 1 or 2) for ACS and a low-acuity score (level 3, 4 or 5) for mild severity diseases.

**Results:**

A total of 190 patients was enrolled over a 5-month period (age 56.8 ±16.4; M/F ratio 1.7). Triage scores of 35 patients (18.4%) with ACS were compared to 103 patients (54.2%) with mild severity diseases. Taking into account CVRF, the sensitivity of the triage system increased from 60 to 80% whereas the specificity decreased from 74 to 61%. Area Under the ROC Curve (AUC) went from 0.69 to 0.72 (Fig. 1).

**Conclusions:**

For chest pain triage at ED, addition of cardiovascular risk factors into ECG-based triage increases his diagnostic performance.

**References:**

1. Dechamps M et al. Intern Emerg Med 12:1245-1251, 2017

2. Taboulet P et al. Eur J Emerg Med 16:61-67, 2009

**Fig. 1 (abstract P287).**
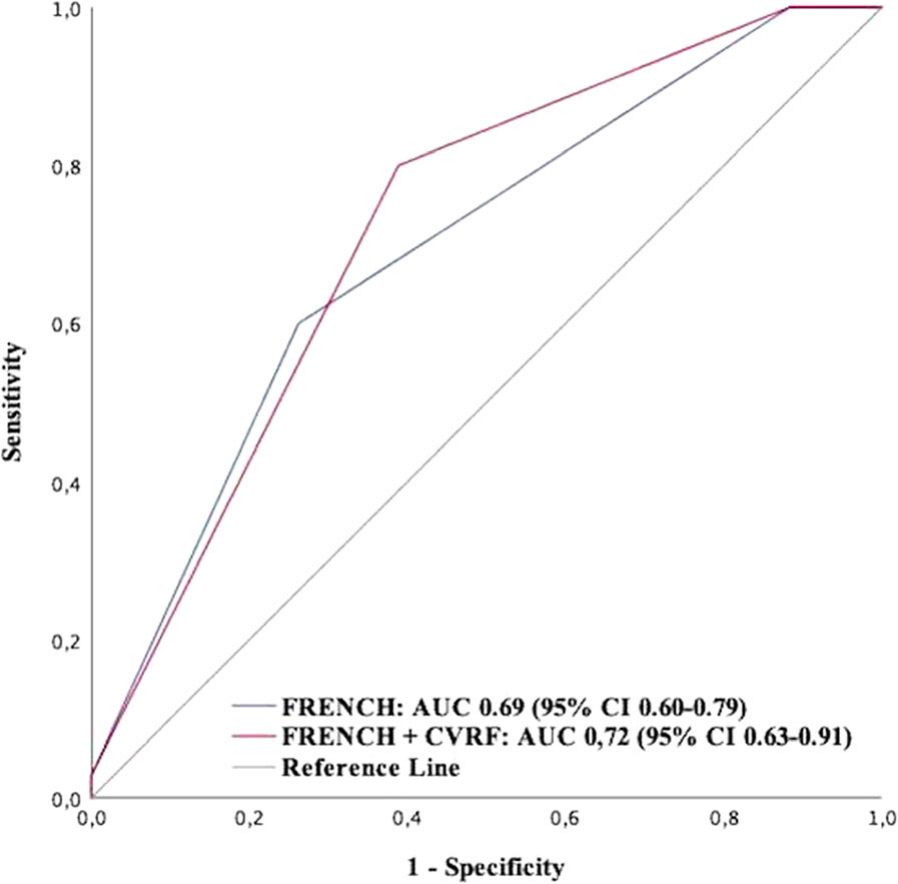
Diagnostic performances of the triage systems

## P288 To scan or not to scan? Should we be performing CT head scans on obtunded patients admitted to intensive care with suspected self-poisoning

### A Lee, R Hunt, P Margetts

#### University Hospital Plymouth Trust, Intensive Care Unit, Plymouth, United Kingdom

**Introduction:**

Approximately 20% of patients presenting to hospital with an intentional overdose require admission to an intensive care unit (ICU) [1]. There are currently no UK guidelines regarding the optimal use of CT head scans (CTH) in this patient cohort [2, 3]. This study aims to determine whether we should be performing CT head scans in obtunded patients with suspected overdose requiring admission to Intensive Care.

**Methods:**

We performed a retrospective search of the ICNARC database for Plymouth University Hospital Trust, looking for patients admitted to the ICU with overdose or self-poisoning as a primary diagnosis. 146 patients were identified and 86 of these patients required intubation due to obtundation(GCS<8).

**Results:**

There were 59 males and 27 females with an average age of 38 years old. The median length of stay on the unit was 1 day. 52 of the patients has a past medical history of mental illness, and 53 overdosed on prescribed medications. The average GCS recorded on admission was 5. 52 of the 86 (64%) patients had a CTH on admission, of which 5 were part of a trauma scan. 11 were known overdoses and 7 were suspected overdose as per the CTH request form. The main rationale behind those requests were to exclude additional intracranial injury. None of those CTH showed any signs of acute pathology (Figure 1).

**Conclusions:**

In this retrospective study, obtunded patients with suspected or known overdose with no history of apparent trauma or injury do not benefit from CTH. In the absence of a history of trauma or focal neurological signs our conclusions are that CTH provides limited value in the management of these patients.

**References:**

1. Clark D et al. JICS 12:2–7, 2011

2. Head injury: assessment and early management; NICE Clinical Guideline (January 2014, updated June 2017)

3. Henderson et al. Intensive Care Med Exp 3(Suppl 1):A498, 2015

**Fig. 1 (abstract P288).**
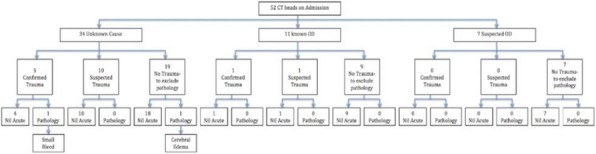
Thematic analysis of CT head scans performed on admission

## P289 Local audit of urinary catheterization practices in accordance with Northampton Healthcare NHS Foundation Trust guidelines 2015

### MH Sultan

#### Lahore General Hospital, Lahore, Pakistan, Department of Internal Medicine, Lahore, Pakistan

**Introduction:**

The audit was carried out to objectively investigate the problems associated with technique of folley catheterization in emergency department and 3 indoor units of internal medicine wards [1].

**Methods:**

A questionnaire was designed in accordance with Northampton Healthcare NHS foundation trust guidelines 2017 and data was collected from 102 House Officers and Post-Graduate residents after permission from Head of Departments of Internal Medicine. Personal help was available to people while they were filling questionnaire in case they have any questions.

**Results:**

45.1% participants claimed that they had no formal training before they started practicing urinary catheterization. Not being fully aware of protocols, 24.5% doctors did not have the habit of taking informed consent from patients or their attendants before catheterization. 74-94% do not wear disposable apron, keep disposable pad under patient’s thighs or clean perineum of patient before catheterization. Almost 60% doctors allow time for lubricant to take its effect before they insert catheter to patients and 58.8% let urine to drain before balloon inflation. Regarding post-catheterization, 25-40% doctors do not guide patients or attendants about when and how to drain catheter bag. For long term catheterization, only 24.5% doctors give written advice about catheter maintenance and 54.9% change catheter bag after 7 days. Similarly, 7.8% percentage didn't know about positioning catheter bag below level of patients and 10% about importance of decontaminating hands before manipulating a patient’s catheter.

**Conclusions:**

Regular teaching sessions should be held for both new and old doctors to improve their technique of catheterization.

**References:**

1. Bhardwai R et al. Br J Nurs 19:S26-30, 2010

## P290 Metformin-associated lactic acidosis in ICU

### M Mackovic, N Maric

#### Clinical hospital Sveti Duh, Zagreb, Croatia

**Introduction:**

Aim of this study is to present a single center experience with metformin-associated lactic acidosis (MALA) along with patient characteristics, type of therapies employed and outcomes. Metformin is the first-line treatement option and one of the most prescribed drugs for diabetes mellitus type 2. Main limitation for its use is chronic renal failure, eGFR <30 ml/min/1,73m^2^ being treshold for its discontinuitation. One of the serious side effects is developement of MALA, reported to be very rare. It is defined by arterial lactate>5 mmol/l and pH<7.35 with recent metformin exposure, blood metformin levels unnecessary for diagnosis.

**Methods:**

Single-centre retrospective study was performed in a medical ICU from Jan2017 to Nov2019. General data, pH, lactate and creatinine levels, need for vasoactive support, dose of bicarbonate administered, necessity and type of renal replacement therapy (RRT) and outcome were assessed.

**Results:**

MALA was diagnosed in 15 of 1710 ICU admissions (incidence of 0.009%). It occured more frequently in females (73%), mean age of 73.9y, mean APACHE II score 33.6. Symptoms on addmisson were: nausea and vomiting (73%), diarrhoea (47%), mental status change (40%), abdominal pain (27%). Predisposing factors for MALA were: infection (27%), CKD (20%), heart failure (13%). Mean pH was 6.96 with mean serum bicarbonates of 5.78 mmol/l, lactate of 16.3 mmol/l and serum creatinine of 648.6 μmol/l. 87% required vasoactive therapy, 33% needing triple vasoactive support. Mean dose of parenteral bicarbonates (8.4%) was 316.7 ml. 40% of patients underwent CPR on admission. All reported patients required RRT, and in 87% CVVHDF was employed. Mortality rate was 40%.

**Conclusions:**

Although reported rare, when occuring, MALA has a high mortality rate. It is associated with profound acidosis, severe renal insufficiency and in our experience high need for vasoactive support and RRT.

## P291 Epigenetic and morphological aspects of brain damage in acute exposure to clozapine combined with ethanol (experimental study)

### A Goloubev^1^, A Babkina^1^, M Khadzhieva^2^, I Rhyzhkov^2^, A Kuzovlev^2^, A Bashirova^3^, D Sundukov^3^

#### ^1^Federal Research and Clinical Center of Intensive Care Medicine and Rehabilitology; Dept. of Forensic Medicine, Peoples’ Friendship University of Russia, Moscow, Russia; ^2^Federal research and clinical center of intensive care medicine and rehabilitology, Moscow, Russia; ^3^Department of Forensic Medicine, Peoples’ Friendship University of Russia, Moscow, Russia

**Introduction:**

Cellular and molecular mechanisms, epigenetic aspects of acute clozapine poisoning are studied insufficiently. The aim of this study was to identify morphological and epigenetic alteratons in brain neurons during acute exposure to clozapine combined wit ethanol.

**Methods:**

The experiments were carried out on male Wistar rats weighting 200–250g (n=21). Group I (control) received 0.9% NaCl solution enterally; group II — clozapine 150 mg/kg in 0.9% NaCl solution; group III — clozapine 150 mg/kg in 40% ethyl alcohol. After 4 hours euthanasia was performed. Autopsy included withdrawal of brain samples for histological examination (n = 21) and for determination of global DNA methylation level (n = 21). The global DNA methylation level (5-mC%) was determinated by fluorimetric method. Inter-group comparisons were made by Kruskal-Wallis test. Histological examination of paraffin sections of brains stained with hematoxylin and eosin was performed by light microscopy.

**Results:**

Histological examination of the cerebral cortex of animals received clozapine and clozapine with alcohol established alterations in the contours of nerve cells, neuronal hyperchromatosis and nuclear deformation, eccentric positon location of the nucleoli, consolidation of Nissl bodies. Increased global DNA methylation level was found in the ‘clozapine+alcohol’ group compared to the control group and ‘clozapine’ group (2.56±0.3140 vs. 1.35±0.1069 and 1.70±0.3295, p <0.02).

**Conclusions:**

In acute сlozapine poisoning and its combination with ethanol morphological changes in neurons of the cerebral cortex were detected. In acute сlozapine with alcohol poisoning an increase of global DNA methylation level was observed. Probably the identified changes have a common pathogenesis which will be clarified in our further studies.

## P292 Prevalence, treatment and outcome of adder bites in South West Wales. Are current guidelines safe?

### S Devine^1^, S Pillai^2^, J Whitley^3^, M Lawrence^4^, K Morris^5^, PA Evans^3^

#### ^1^Welsh Centre for Emergency Medicine Research, Emergency Department, Swansea, United Kingdom; ^2^Welsh Centre for Emergency Medicine Research, Emergency Department/Intensive Care Unit, Swansea, United Kingdom; ^3^Welsh Centre for Emergency Medicine Research, Welsh Centre for Emergency Medicine Research, Swansea, United Kingdom; ^4^Swansea University, Swansea University, Swansea, United Kingdom; ^5^Cardiff Metropolitan University, Cardiff Metropolitan University, Cardiff, United Kingdom

**Introduction:**

The common European adder (*Viperia berus*) is the only venomous snake indigenous to the UK. Although non-aggressive if disturbed adders may bite, envenomation can be life threatening or even fatal. There is limited information available regarding the prevalence of adder bites and the complications of envenomation. NHS data suggests there are 100 adder bites annually in the UK with the last fatality in 1975 [1]. We performed an audit into adder bites in South West Wales to identify the number attending our Emergency Departments, their management and clinical course as well as any environmental factors that predict increased likelihood of being bitten or the severity of the bite.

**Methods:**

A retrospective study of adder bites attending Emergency Departments in South West Wales was undertaken (Jan 2014 to Aug 2019). Measurements included were patient demographics, clinical presentation, type of treatment (conservative vs anti-venom) and outcome.

**Results:**

31 patients were included, age range 2-72 years (Figure 1). The majority of bites occurred in sand dunes (41.9%) and all bites were on extremities. Anti-venom was administered to 45.2% (14/31) of patients. There was a significant positive association between the use of anti-venom and the length of hospital stay (r = 0.520; p=0.003) and a significant negative correlation between the anti-venom use and both diastolic and systolic blood pressure (p= 0.501 and 0.487 respectively p=0.01). All patients fully recovered.

**Conclusions:**

In this study, we demonstrated that with a full clinical assessment on presentation it is safe to decide whether anti-venom is required. The current guidelines are safe and effective in the treatment of adder bites.

**References:**

1. Snake Bites, NHS Conditions, 2016. Accessed at https://www.nhs.uk/conditions/snake-bites/ on 22/11/19

**Fig. 1 (abstract P292).**
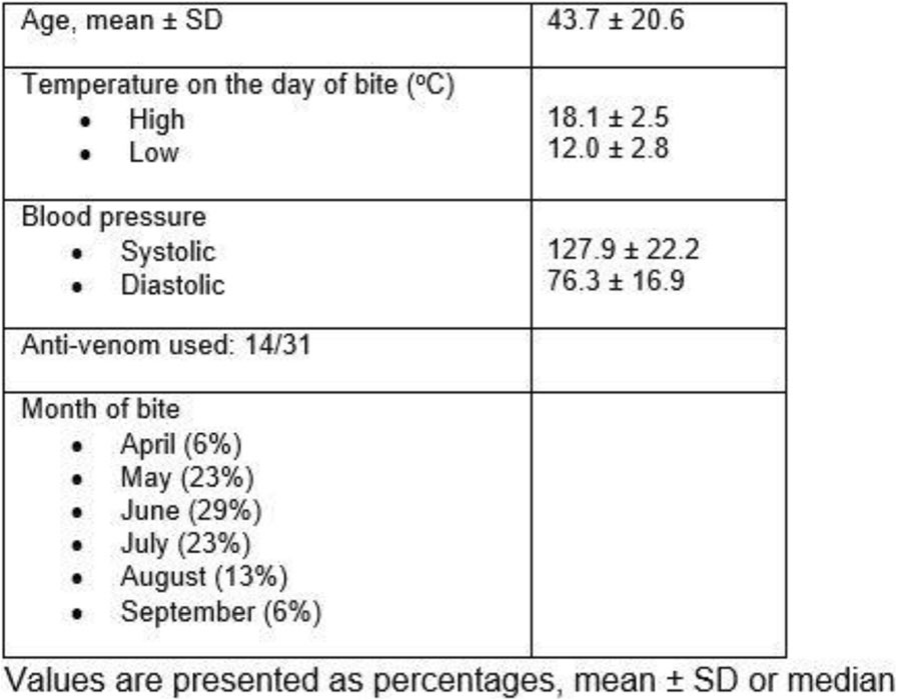
Patient demographics and climate data (n=31)

## P293 Cut-offs for increased risk of 7-day mortality, for vital- and laboratory values; have we got them right?

### PB Pedersen^1^, DP Henriksen^2^, M Brabrand^1^, AT Lassen^1^

#### ^1^Odense University Hospital, Department of Emergency Medicine, Odense C, Denmark; ^2^Odense University Hospital, Department of Clinical Biochemistry and Pharmacology, Odense C, Denmark

**Introduction:**

The aim was to validate or refute the known thresholds for increased risk of 7-day mortality among acute patients, according to vital and laboratory values. Vital- and laboratory values measured at arrival to an emergency department (ED), are part of different risk-stratification scores, like the Sequential Organ Failure Assessment score, that indicates whether a patient is critical ill or in risk of deterioration.

**Methods:**

A population-based cohort study at the ED at Odense University Hospital, covering all adult patients ≥18 years arriving from 1 April 2012 to 31 March 2015. Patients were included at first contact within the study period. Variables were first recorded standard vital values and laboratory-values included in risk-stratification scores; respiratory rate, blood pressure, heart rate, Glasgow Coma Scale, temperature, saturation, creatinine, PaO_2_, platelet, and bilirubin. The association between vital- and laboratory values and mortality were described using a restricted cubic spline. A predefined 7-day mortality rate of 2.5% was chosen as a relevant threshold of increased risk.

**Results:**

We included 40,423 patients, 52.5% female, median age 57 (IQR 38-74) years, and 7-day mortality 2.8%. Seven-day mortality of 2.5% had thresholds of; respiratory rate < 12/min and > 18/min, systolic blood pressure < 112mmHg and > 192mmHg, heart rate < 54beats/min and > 102beats/min, temperature < 36.0°C and > 39.8°C, saturation < 97%, Glasgow Coma Scale < 15, creatinine < 41μmol/L and > 98μmol/L, for PaO2 < 9.9kPa and > 12.3 kPa, platelets < 165*10^9/L and > 327*10^9/L, and bilirubin > 12μmol/L.

**Conclusions:**

In our population of adult ED patients, the thresholds of vital values associated with increased 7-day mortality were very close to routinely used values, and most of the thresholds were included in the lowest urgency level in triage and risk-stratification scoring systems.

## P294 The workload in the emergency room: direct assessment by the Therapeutic Intervention Scoring System-76 and indirect assessment by the NASA Task Load Index

### NE Nouira^1^, L Arbi^2^, D Chtourou^1^, A Chamsi^1^, W Bahria^1^, EM Ben Othmane^1^, M Bouraoui^1^, M Ben Cheikh^1^

#### ^1^Mongi Slim Academic Hospital, Emergency Department, Tunis, Tunisia; ^2^Ministry of Public Health, Directorate of Medical Inspection, 1029 Bab Saâdoun Tunis, Tunisia

**Introduction:**

The number of emergency room admissions continues to increase each year, which increases the care workload of the emergency department staff, who should to use its theoretical and practical knowledge in order to provide quality care in difficult working conditions. The aim of our study was to assess the emergency room staff workload its impact on health workers and patients and to suggest an improvement strategy to decrease this workload.

**Methods:**

A prospective, monocentric cohort study with descriptive and analytic approach over one month (December 2018) conducted at the Emergency Department of an academic hospital. The workload endured by the emergency room staff was evaluated by the NASA Task Load Index and on patients by the Therapeutic Intervention Scoring System-76.

**Results:**

There were 286 cumulative days of hospitalization in 67 consecutive patients admitted to the emergency room. The average age was 61 ±15 years. The average length of stay at the emergency room was about 103 ±48h. The average TISS-76 score was 31.7 ±14.9. Factors associated with important care workload were: age ≥ 65 years, diabetes, more than 3 comorbidities, the use of intravenous antibiotics; the use of vasoactive drugs and the use of mechanical ventilation; a high TISS score was predictive of emergency room mortality. In the indirect assessment of the care workload, 41 medical and paramedical staff were interviewed, 73% of them were under 40 years old with a sex ratio of 0.58. A high level of mental and physical workload was expressed by ED staff with considerable level of frustration; The ED staff suggested mainly to improve the working conditions, communication and to redefine tasks "who does what".

**Conclusions:**

Our study had shown a significant workload in the emergency room, a process to reduce this workload is being implemented

## P295 The role of simulation-based education in cardiovascular emergencies for medical students’ basic training

### R Benmalek^1^, S Abouradi^2^, R Habbal^2^, M Mouhaoui^3^

#### ^1^CHU Ibn Rochd, Cardiology Department, Casablanca, Morocco; ^2^Cardiology department, CHU Ibn Rochd, Casablanca, Cardiology, Casablanca, Morocco; ^3^SAMU Urgences 02, Samu urgences 02, Casablanca, Morocco

**Introduction:**

Medical simulation is a modern teaching tool increasingly used in specialties such as anesthesia, emergency medicine and obstetrics. However, it’s not widely used in specialties like cardiology, althought cardiovascular emergencies are very frequent. The purpose of our study was to assess the effectiveness of simulation-based medical education in the management of cardiovascular emergencies among moroccan graduate students.

**Methods:**

We conducted a prospective, observational, multi-centrer study including the students of three moroccan universities from the 5^th^ to the 7^th^ year of medicine who underwent 6 phases: First a pre-test, then a theoretical and practical training on cardiovascular emergencies after which the students were separated in two groups, one undergoing the medical simulation training (group 1) and one who didn’t (group 2), followed by a theoretical then a practical post-test on *Resusci Anne and* SimMan®. At last, the students were asked to answer a satisfaction survey.

**Results:**

57 students were enrolled in the study and divided into 2 groups (Group 1, N= 25 and Group 2, N=27). Both groups were comparables in terms of year of study, faculty and their previous internships as well as a former participation in medical simulation. The pre-test results were comparable between the two groups (6, 05±2,68 for group 1 versus 5,88±2,52 for group 2). However, both theoretical post-test results (18,83±1,27 versus 17,13±2,01 for group 2, p<0,001) and practical evaluation results (16,15 ± 1,72 versus 10,92 ± 1,35, p< 0,001), were in favor of group 1.

**Conclusions:**

This preliminary study showed the significant benefit of simulation-based medical teaching and learning as an innovating teaching tool in the cardiovascular emergencies in the medical students’ basic training and suggests its generalization to the cardiology and intensive care interns’ continuous training.

## P296 Great East Japan Earthquake and Tsunami supressed family- and friend-performance of cardiopulmonary resuscitation

### H Inaba^1^, K Takada^2^, H Kurosaki^2^, Y Wato^3^, A Yamashita^2^

#### ^1^Kanazawa University Graduate School of Medicine, Department of Circulatory Emergency and Emergency Medical Science, Kanazawa, Japan; ^2^Kanazawa University Graduate School of Medicine, Kanazawa, Japan; ^3^Kanazawa Medical University, Uchinada, Japan

**Introduction:**

Disaster may psychologically affect the social behaviour of citizens [1]. However, the impact of great disaster on basic life support activities of laypersons is unknown. The East Japan Earthquake swept the Pacific coast of Japanese mainland on Friday 11 March 2011, and caused nuclear accidents. This study aimed to investigate whether and how this disaster influenced bystander cardiopulmonary resuscitation (BCPR) in family- and friends-witnessed out-of-hospital cardiac arrests (OHCAs).

**Methods:**

From nationwide OHCA registry, we extracted 74,684 family and friend bystander-witnessed OHCA cases without prehospital physician-performed advance life support, which were recorded between 11 March, 2010 and 10 March, 2013. The alterations in BCPR rate after the disaster in Tsunami-affected prefectures and others were analysed by univariate and multivariable analyses.

**Results:**

The BCPR rate in Tsunami-affected prefecture decreased in a biphasic manner during 20-weeks period after the onset of disaster in disaster year of 2011, compared to pre-disaster 2010 and post-disaster 2012: 42.5% (375/882) in 2011 and 48.2% (754/1565) in 2010&2012. This decrease in BCPR rate in tsunami-affected prefectures was more prominent in the subgroup receiving dispatcher-assisted CPR instruction (Figure 1). A lower rate of dispatcher-assisted CPR instruction (49.1% vs 53.4%) and higher proportion of presumed cardiac aetiology (62.8% vs 56.0%) were also observed during the same period of 2011 in Tsunami-affected prefectures. One-month survival rate of the family- and friends-witnessed subgroup receiving dispatcher-assisted CPR was 8.2% in 2011 and 10.2% in 2010/2012. In Tsunami-unaffected prefectures, the changes in BCPR rate and outcome of family- and friends-witnessed OHCA were extremely small.

**Conclusions:**

A great disaster affects the family- and friend-performance of BCPR by diminishing the willingness of family and friend bystanders to follow the instruction provided by dispatchers.

**References:**

1. Bravo M et al. Am J Commun Psychol 18: 661-680, 1990

**Fig. 1 (abstract 296).**
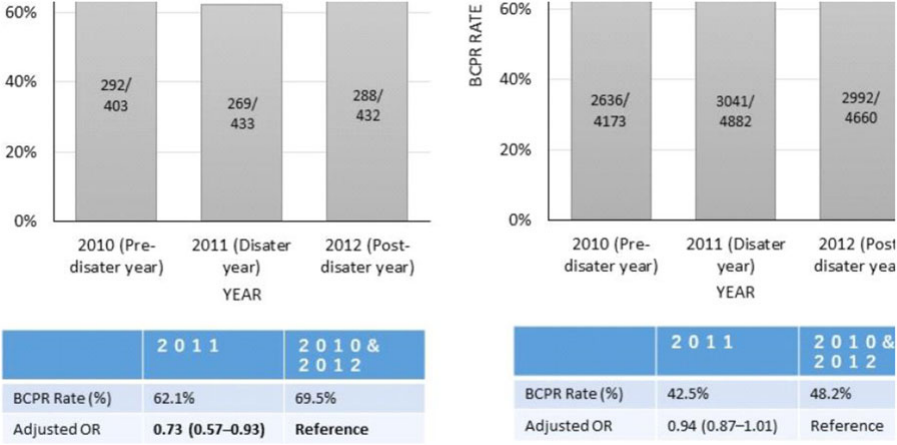
Changes in total BCPR rate in family- and friends-witnessed OHCA cases with dispatcher-assisted instruction during 20-week period after the day of disaster during three years

## P297 Prognostic value of ISS and TRISS scores in terrorism victims

### C Romdhani^1^, T Khoufi^2^, M Shimi^3^, W Sellami^4^, A Mrabet^2^, M Ferjani^2^

#### ^1^Military Hospital of Gabes, Anesthesiology & Intensive care, Gabes, Tunisia; ^2^Ministry of Defense, Directorate General of Military Health Tunisian Armed Forces, Tunis, Tunisia; ^3^Military Hospital of Tunis, Department of Forensic Medicine, Tunis, Tunisia, ^4^Military Hospital of Tunis, Departement of Anesthesiology & Intensive Care, Tunis, Tunisia

**Introduction:**

This study aimed to assess the prognostic value of the Injury Severity Score (ISS) and the Trauma-Related Injury Severity Score (TRISS) for predicting mortality in terrorism victims.

**Methods:**

We conducted a retrospective study from February 2012 to December 2017. We included all terrorism victims in Tunisia who were hospitalized or autopsied at the Tunis Military Hospital. The performance of the ISS and TRISS scores was studied using the area under the ROC curve (AUC). We determined the best threshold using the Youden index to predict 28-day survival.

**Results:**

We included 168 patients. We excluded 15 patients because the records were unusable. Forty-six victims (30%) died (43 at the scene and 3 in hospital). We found 79 blast injuries (51.6%), 65 bullet injuries (42.5%), 2 (1.3%) bullet and blast injuries, and 2 (1.3%) stab wounds ( sticking). The area under the ROC mortality prediction curve was 0.955 (95% confidence interval: 0.925-0.984) for the ISS and 0.948 (95% confidence interval: 0.916-0.980) for the TRISS. The best threshold values ​​for predicting mortality were 23.50 for the ISS score (Sensitivity: 100%, Specificity: 83%) and 22.38 for the TRISS score (Sensitivity: 93%, Specificity: 89%).

**Conclusions:**

The ISS and TRISS scores can be used for prognostic evaluation to predict mortality in terrorism victims.

## P298 Mega-disaster medicine, including NBC hazards and intensivist

### Y Haraguchi^1^, Y Tomoyasu^2^, T Tsubata^2^, M Hoshino^3^, M Sakai^2^, T Ishihara^2^, E Hoshino^2^

#### ^1^2 chome Matsue Edogawaku ward, Keiyo Hospital, Tokyo, Japan; ^2^2 chome Matsue Edogawaku ward, Disaster Medicine Compendium Group, Tokyo, Japan; ^3^2 chome Matsue Edogawaku ward, Disaster Medicine Compendium Group, Keiyo Hospital, Tokyo, Japan

**Introduction:**

The large number of casualties during mega-disasters are serious global problems. Intensivist's role is critically analyzed especially focusing on the special disasters and the vulnerable

**Methods:**

Our actual medical experience on disaster cites was basically researched. Tokyo Subway Sarin attack (1995), 9-11 attack (2001), Hurricane Katrina (2005), Indian Ocean earthquake/tsunami (2004), Chernobyl (1986) and Higashi Nihon Earthquake/Fukushima nuclear plant catastrophe (2011), Flu pandemic (2009), etc. are analyzed.

**Results:**

These mega-disasters had severe negative health and mental influence of various type. Linked catastrophe forms a malignant cycle. In these situations most of the intensivist seemed useless. However many essential roles of intensivist/medical staff existed which were reviewed: i.e., as reliable medical/surgical/intensive care team leader, and/or chief triage officers, in addition to operate in the field/shelter for safety/security as well as making up preventable life-saving systems. In order to support/help victims in shelter, who are often abused or ignored, diseased chronically and injured by trauma, and contaminated/damaged by NBC (nuclear, biological and chemical) or BCRNE (biological, chemical, radiological, nuclear and explosive) hazards. Especially regarding NBC hazard, intensivist should be accustomed more, which were often overlooked or ignored even in Japan DMAT( disaster medical assistant teams).

**Conclusions:**

We had been compiling Disaster Medicine Compendium, completed first version with 22 volumes, 2005, although this version had three-fourth in Japanese. Second version were four-fifth in English by adding several important parts. At present third version are nearly completed with mainly focusing on the vulnerable, including ding LGBD (lesbian, gay, bisexual and transgender), pets/animals/vegetables, religious problems and/or natural condition, etc.

## P299 Repair of the bodily injury to those injured by terrorism in Tunisia (2013-2019)

### M Gabouj

#### Ministère de la défense Nationale Tunisienne, Centre de réforme, Tunis, Tunisia

**Introduction:**

The reform procedure in the Tunisian army consists in repairing the physical damage and deciding on the applicant's ability to continue working. Terrorism increases the impact of the co-morbidity generated and the socio-economic consequences that result from it. The purpose of this work was to study the epidemiological, clinical and evolutionary profile of terrorist injuries, to specify the rates of consequent Partial Permanent Disability (PPI) and the possibilities of returning to work.

**Methods:**

Descriptive retrospective cross-sectional study of 177 reform files on military personnel injured during anti-terrorist operations from January 2013 to September 2019. The data collection was carried out on the basis of a collection form.

**Results:**

- Our 177 wounded were male, 96% of whom belonged to the army.
- The average age was 36 years and 3 months ± 8.869.
- Half of our wounded were troopers.
- Infantry and special forces were the most exposed military units.
- Half of the accidents were recorded in the Kasserine region (88 cases).
- Chronic post-traumatic stress disorder (CPTSS) was found in 130 injured, followed by amputations in 18 injured.
- The after-effects were psychological in 32%, physical in 26% and mixed in 39% of our injured.
- The PPI rate ranged from 36% to 75% in 23.7% of injuries..
- More than half of the injured had returned to their professional activity, 33% were put on reform for health reasons.

**Conclusions:**

Our results showed that the ESPTC was the most recorded sequel, and that the PPI rate was significant in a quarter of our injuries. In our series, a third of our wounded were put on reform for health reasons. To state the importance of initial care and adequate and rigorous follow-up to recover a greater number of war wounded.

## P300 Questionnaire survey on prevalence of rapid response systems in acute-care hospitals in western Japan

### J Ishii^1^, H Kamada^2^, K Hosokawa^1^, S Yamaga^1^, K Ota^1^, N Shime^1^

#### ^1^Hiroshima University Hospital, Department of Emergency and Critical Care Medicine, Graduate School of Biomedical and Health Sciences, Hiroshima, Japan; ^2^Hiroshima University Hospital, Medical student, Department of Medicine, Hiroshima, Japan

**Introduction:**

The rapid response system (RRS) has been shown to decrease hospital mortality [1]. The Japanese Coalition for Patient Safety has set a major goal for hospitals to more widely implement the RRS. However, prevalence and actual circumstances of use in acute care hospitals (including small scale hospitals) in Japan are as yet not well-known.

**Methods:**

Web-based questionnaires were sent to acute care hospitals (of scale 75 beds-or-larger) of 17 prefectures in western Japan. Each participant hospital selected a certain department which answered the questionnaire. The RRS included the medical emergency team (MET), the rapid response team (RRT), and the critical care outreach team (CCOT). We investigated the presence and circumstances of in-hospital emergency calls, RRS and other systems, and then illuminated issues to be solved.

**Results:**

Among the 971 hospitals which questionnaires were distributed to, a total of 149 hospitals (15.3%) replied. The in-hospital emergency call and RRS were operated in 117 (78.5%) and 26 (17.4%) hospitals, respectively. Among the middle-to-small scale hospitals with ≤ 200 beds, both in-hospital emergency calls and RRS were less frequently operated than larger hospitals with > 200 beds (69.6% vs. 83.9%, p=0.04; 5.4% vs. 24.7%, p=0.003, respectively). Several hospitals operated non-team services through a certified critical care nurse. Data of activities was registered to the In-Hospital Emergency Committee in Japan in 7 (4.6%) hospitals. Satisfaction rates with the effectiveness of their own RRS were 60%, and problems to be resolved were shortage of staff and knowledge, and a lack of flow during response.

**Conclusions:**

Compared to larger hospitals with > 200 beds, the prevalence of the RRS was significantly lower in middle-to-small scale hospitals. For them, we suggest that efficacious activities and education programs for RRS be sought.

**References:**

1. Lyons PG et al. Resuscitation 128: 191-197, 2018

## P301 Higher rapid response system call rate was associated with decreased short-term serious outcome: Japanese nationwide database retrospective study

### T Kurita^1^, TA Nakada^2^, R Kawaguchi^2^, S Fujitani^3^, K Atagi^4^, T Naito^3^, M Arai^5^, H Arimoto^6^, T Masuyama^7^, S Oda^1^

#### ^1^Chiba University Graduate School of Medicine, Department of Emergency and Critical Care Medicine, Chiba, Japan; ^2^Chiba University Graduate School of Medicine, Chiba University Graduate School of Medicine, Department of Emergency and Critical Care Medicine, Chiba, Japan; ^3^St. Marianna University School of Medicine, Department of Emergency and Critical Care Medicine, Kanagawa, Japan; ^4^Nara General Medical Center, Intensive Care Unit, Nara, Japan; ^5^Research and Development Center for New Medical Frontiers, Kitasato University School of Medicine, Division of Advanced Surgical Oncology, Kanagawa, Japan; ^6^Osaka City General Hospital, Department of Emergency and Critical Care Medical Center, Osaka, Japan; ^7^Jichi Medical University Saitama Medical Center, Department of Emergency and Critical Care Medicine, Saitama, Japan

**Introduction:**

Association of hospital volume or rapid response system (RRS) call rate with altered clinical outcomes of RRS patients was not fully investigated. We tested a hypothesisthat hospital bed number and RRS call rates are associated with altered clinical outcomes of patients with RRSs.

**Methods:**

We performed a retrospective analysis of a large RRS cohort, In-Hospital Emergency Registry in Japan (IHER-J), which is a data registry of RRSs conducted by the Japanese Society of Intensive Care Medicine and the Japanese Society for Emergency Medicine. This study enrolled 4,818 patients in 24 hospitals from April 2014 to March 2018. Primary outcome variable was short-term serious outcome defined as unplanned intensive care unit (ICU) admission after RRS activation, or death at the scene where RRS care was initiated.

**Results:**

There was no significant correlation between the number of hospital bed and RRS call rate (R^2^ =0.0047,P=0.75). In the primary analysis of the presentstudy using a multivariate analysis adjusting potential confounding factors,higher RRS call rate was significantly associated with decreased short-term serious outcome (P=0.0010, OR 0.94, 95% CI [0.91-0.98]), but there was no significant association of hospital volume (P=0.97).In the secondary analysis of the study, there was a non-significant trend of increased cardiac arrest on arrival at the location of the RRS provider at large-volume hospitals (P=0.084, OR 1.16, 95% CI 0.98-1.38) but there was no significant association of the RRS call rate with the incidence of cardiac arrest (P=0.60). Large-volume hospitals had a significantly higher 1-month mortality rate (P=0.0040, OR 1.10, 95% CI 1.03-1.18) but there was no significant association of RRS call rate with 1-month mortality rate (P=0.76).

**Conclusions:**

Hospitals with increased RRS call rates had significantly decreased adverse short-term outcomesin patients who had RRS activations. Patients who had RRS activations at large-volume hospitals had an increased 1-month mortality rate.

## P302 Time from rapid response team activation to ICU admission

### J Camões^1^, R Guedes^2^, S Gaião^3^, R Pimentel^2^, R Roncon-Albuquerque Jr^4^, JA Paiva^3^

#### ^1^ULS Matosinhos, Department of Emergency and Intensive Care Medicine, Matosinhos, Portugal; ^2^São João Hospital Center, Department of Emergency and Intensive Care Medicine, Porto, Portugal; ^3^São João Hospital Center | Faculty of Medicine - University of Porto, Department of Emergency and Intensive Care Medicine| Department of Medicine, Porto, Portugal; ^4^São João Hospital Center | Faculty of Medicine - University of Porto, Department of Emergency and Intensive Care Medicine | Department of Surgery and Physiology, Porto, Portugal

**Introduction:**

Early identification of patient’s deterioration on wards and appropriate Rapid Response Team (RRT) response have been implemented in many healthcare facilities. However, the impact of delays in patient transfer to the Intensive Care Unit (ICU) after an RRT assessment was poorly studied. Our aim was to assess time from RRT activation to ICU admission (RRT-ICU time) and study its impact on clinical evolution.

**Methods:**

We conducted a retrospective study between January and August 2019 including patients admitted from wards to ICU, after RRT activation, in a University Hospital. RRT-ICU time was measured, physiological/clinical parameters were collected and mortality was assessed. We considered 2 hours as the cutoff time between early and late admission.

**Results:**

We enrolled 119 patients, 57.1% male, median age 69 years, APACHE II 21, SAPS 43, RRT-ICU time 4 hours, 86.1% medical admissions, 37.8% sepsis/septic shock. ICU and hospital mortality were 22.6% and 34.0%. Comparing early (E,n=30) and late (L,n=76) admission groups, there were no statistically significant differences between mean SAPS II (44.53 vs 41.77, p=0.523), APACHE II (21.00 vs 21.49, p=0.564), sepsis/septic shock (46.7% vs 40.8%, p=0.581) or admission motive. There were no differences between ICU length of stay (5.5 vs 6.0, p=0.332), hospital length of stay (24.5 vs 24.5 days, p=0.997), ventilation days (0.0 vs 0.0, p=0.881), vasopressors days (0.5 vs 0.0, p=0.770), and ICU mortality (13.3% vs 26.3% p=0.150). There was a clear but not statistically significant difference in hospital mortality between E and L groups (20.0% vs 39.5%, p= 0.057). Regarding the assessment at admission and 72 hours, differences were found between E and L groups for bilirubin (-16.6% vs 6.6%, p=0.002) and lactate (-44.8% vs -21.1%, p=0.011) variation.

**Conclusions:**

Our study suggests that delays in patient transfer to the ICU after RRT activation in the wards were associated with slower physiological improvement.These findings support further and larger studies.

## P303

**Withdrawn**

## P304 Blood and blood products use in intensive care unit

### M Akcivan, S Bozbay, O Demirkiran

#### Istanbul University Cerrahpasa, Anesthesiology and Intensive Care, Istanbul, Turkey

**Introduction:**

Blood and blood product (BP) transfusions are frequently used in intensive care units (ICU) [1]. It is important to know transfusion epidemiology and the effect of adverse transfusion reactions and their effect on mortality and morbidity.We aimed to investigate the blood and BP transfusions in the ICU.

**Methods:**

Blood and BP transfusions in ICU, between 2013-2017 were reviewed retrospectively. We evaluated each transfusion as a data and examined the pre- and post-transfusion laboratory values, demographic data, cause of ICU admission and comorbidities.

**Results:**

284 patients who underwent transfusion in the ICU, and 2188 transfusion data from these patients were included. The most frequent cause of hospitalizations were respiratory failure and sepsis. The rate of patients transfused in the five-year period decreased from 73.9% to 36.67%. The hemoglobin threshold before transfusion decreased from 8.34 g / dl to 7.91 g / dl. A total of 148 transfusion reactions were observed and the most common transfusion reaction was febrile non-hemolytic reaction. The most commonly transfused product was red blood cell suspension. Transfusion reactions were found to be slightly higher in men than women in young age group(<65y) (p = 0.44 and p=0.021, respectively). Transfusion reactions were found to be more frequent in emergency transfusions (p <0.01). The number of transfusions was significantly lower in patients with APACHE II score <20 (p <0.01). The need for transfusion was found to be higher in patients with hematological malignancy (p <0.01). It was observed that as the mean number of transfusions increased the mortality is also increased (p <0.01).

**Conclusions:**

Transfusion therapies are the treatments that are vital but have a serious mortality and morbidity risk. In particular, intensive care patients should be considered in detail because of their specific features. Restrictive transfusion practices have positive results.

**References:**

1. Vincent JL. Crit Care Med 34:96-101, 2006

## P305 Association between anemia or red blood cell transfusion and outcome in oncologic surgical patients. A retrospective observational study

### X Chapalain^1^, Y Ozier^1^, C Le Niger^2^, O Huet^1^, C Aubron^3^

#### ^1^Brest University Hospital, Department of Anaesthesiology and Surgical Intensive Care., Brest, France; ^2^Brest University Hospital, Hemovigilance Unit, Brest, France; ^3^Brest University Hospital, Medical Intensive Care Unit, Brest, France

**Introduction:**

Up to 40% of surgical oncologic patients receive red blood cells (RBC) to treat anemia. We aim to determine the potential impact of anemia and red blood cells (RBC) transfusion on post-operative complications and mortality in oncologic surgical critically ill patients.

**Methods:**

Retrospective, single center study. Adults admitted to intensive care unit (ICU) after surgery for cancer with a high risk of bleeding were eligible. Multivariate analyses were preformed to determine whether anemia and/or RBC transfusion were associated to post-operative complications and/or in-hospital mortality.

**Results:**

From January 2017 to December 2018, 287 oncologic patients were admitted to ICU, 49.5% of the patients had anemia based on the World Health Organization (WHO) definition; 69 (24%) had moderate anemia (Hb < 10 g/dL and ≥ 8g/dL) and 32 patients (12.5%) had severe anemia (Hb < 8 g/dL). RBC were given to 19.6% of the patients. Patients exposed to moderate-to-severe anemia had more post-operative complications. Patients who received RBC transfusion had also more post-operative complications including renal failure (0.9% vs. 19.6% ; *p* < 0.001), thromboembolic events (2.2% vs. 8.9% ; *p* = 0.039), and infections (13.5% vs. 41.1% ; p < 0.001) compared to non-transfused patients. Multivariate analysis found an independent association between moderate (OR 15.03 [2.73 – 282.3] ; p < 0.001) and severe anemia (OR 16.65 [2.71 – 325.7] ; p = 0.011) and post-operative complications (Figure 1A). The association between RBC transfusion and adverse events also remained after adjustment (OR 4.3 [2.2 – 8.8] ; *p* < 0.001) (Figure 1B).

**Conclusions:**

In oncologic surgical critically ill patients, there was an independent association between anemia (even moderate anemia) or RBC transfusion and patient outcomes. Our findings highlight the need for further research to determine the optimal transfusion strategy in surgical oncologic patients.

**Fig. 1 (abstract 305).**
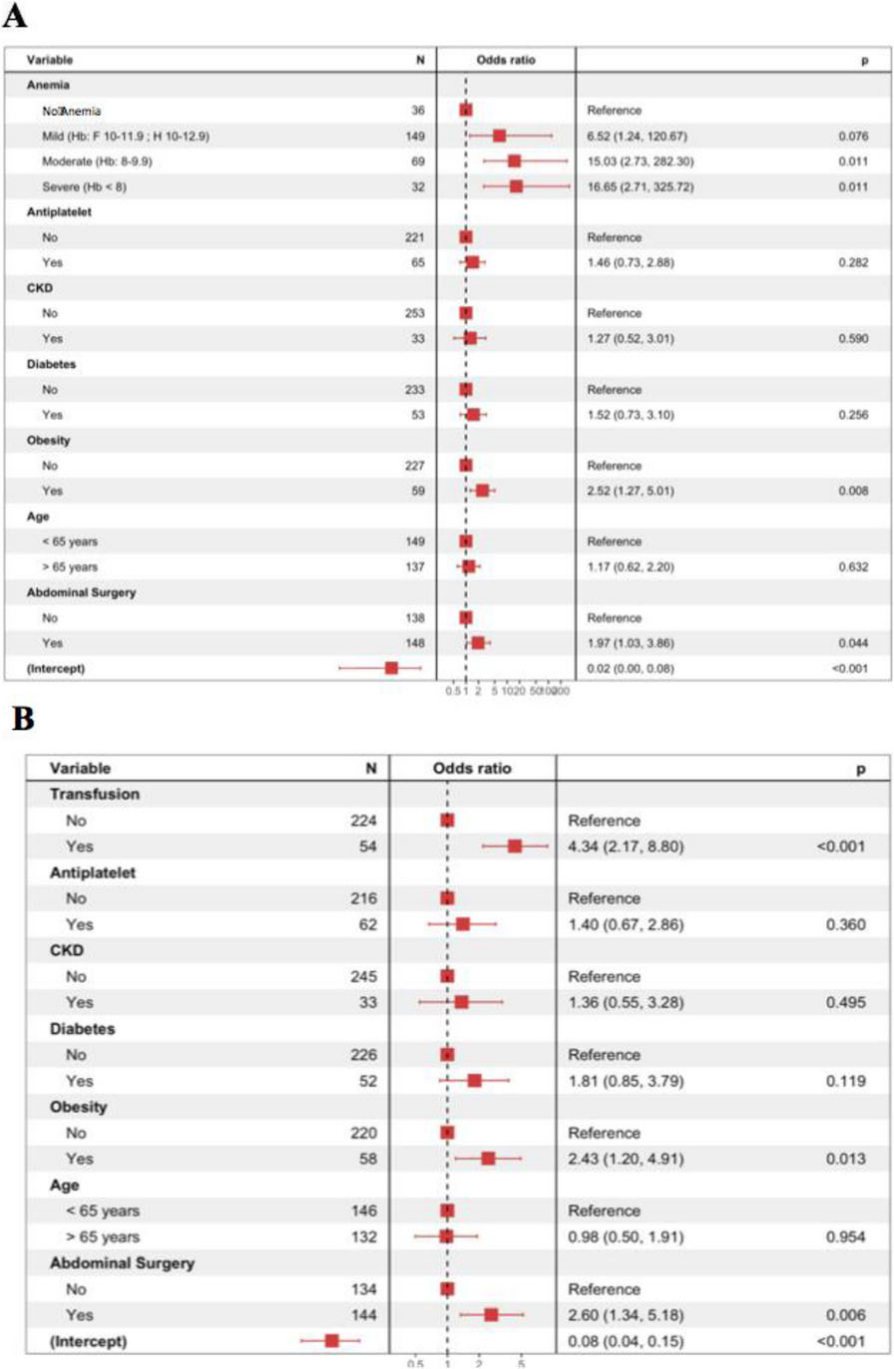
Multivariate analysis analyzing the risk factors for the primary outcome (severe post-operative complications and /or mortality) including anemia (A) or transfusion (B). (CKD: Chronic Kidney Disease)

## P306 Transfusion impaired skin blood flow when initially high

### E Cavalcante dos santos, W Mongkolpun, P Bakos, AL Alves da Cunha, C Woitexen Campos, JL Vincent, J Creteur, FS Taccone

#### Erasme Hospital, Intensive Care Department, Brussels, Belgium

**Introduction:**

Red blood cell transfusion (RBCT) increases global oxygen delivery (DO_2_) and may improve microcirculation. However, the effects on blood flow have been found to be conflicting.

**Methods:**

We studied ICU patients with stable hemodynamic status (mean arterial pressure (MAP) ≥ 65 mmHg for at least 6 hours) and without active bleeding, who received a RBCT. Skin blood flow (SBF) was determined (Periflux System 5000, Perimed, index finger; perfusion unit, PU) together with MAP, heart rate (HR), hemoglobin (Hb), lactate levels and ScvO_2_ before and after RBCT. SBF was measured before RBCT (T0) and after (T1) for each 3 min. According to previous data indicating the lowest SBF value found in non-infected ICU patients was 151 PU, all patients were analyzed according to the baseline SBF (i.e. <151 PU - low SBF vs. ≥151 PU – high SBF). The relative change of SBF (∆SBF) was calculated after RBCT and the responders were defined by the function of >10%.

**Results:**

63 ICU patients were studied. RBCT was associated with increases in MAP and ScvO_2_ but no change in SBF. At baseline, ScvO_2_ was lower in the responders than in the non-responders (p=0.04) and lower in patients with low SBF than in the high SBF (p=0.04). There was no difference in Hb, MAP, and lactate, between the patients with low and high SBF. After RBCT, MAP rose in the responders (p<0.01) and in the non-responders (p=0.03), SBF (p<0.01) rose in patients with low SBF, and SBF (p=0.02) decreased in patients with high SBF. There was a negative correlation between baseline ScvO_2_ (r= -0.363, p<0.01) or baseline SBF (r= -0.560, p<0.01) and the relative increase in SBF after RBCT.

**Conclusions:**

RBCT increases skin blood flow only when it is impaired at baseline.

## P307 Effects of erythrocyte transfusion on hemodynamic and oxygen metabolism: cross-sectional study of paired series

### A Trifi^1^, A Mehdi^2^, H Fazzeni^2^, F Daly^2^, Y Touil^2^, S Abdellatif^2^, S Ben Lakhal^2^

#### ^1^La Rabta hopital, Medical intensive care unit., Tunis, Tunisia; ^2^La Rabta hopital, Tunis, Tunisia

**Introduction:**

Erythrocyte transfusion (ET) is a common practice in ICUs. While it can save lives in hemorrhagic shock, its efficacy is more uncertain in moderate anemia of inflammatory origin. There is still considerable debate about the risks / benefits and indications of transfusion of ET in patients with non-hemorrhagic anemia. We aimed to study the effects of ET on hemodynamic and oxygen metabolism.

**Methods:**

Comparative cross-sectional study on matched series before / after ET. Patients receiving ET for non-hemorrhagic anemia (Hb <7 g / dL) were eligible. Ultrasound Cardiac output (CO), arterial and central venous blood gas were performed before and 2h after transfusion. Blood Formula Count (BFC) was collected 24 hours after ET. All hemodynamic and O2 metabolism parameter’s (using the following formulas: arterial O2 content (CO2, ml / dl) = 1.34 (ml / g) x Hb (g / dl) x SO2 + [(0.0031 x PO2 (mm Hg)] and Oxygen delivery (DO2, ml / min) = CO (l / min) x CO2 x 10) and hematologic were compared before/after ET by paired samples

**Results:**

20 patients were included of 43 years as median age and median SOFA = 6. The median Hb indicating ET was 6.6 [6.2-6.9]. After transfusion of 1 RBC pellet: CO2 (ml / dl) increased from 8.9 [8.5-9.3] to 10.4 [9.4-11.3] with p <10^-3^. CO (l / min), and DO2 (ml / min) decreased but did not reach significance: CO: 5.6 [4.4 -7.01] vs 4.6 [3.8-6.7] and p = 0.33, CI: 3.2 [2.4-4.1] vs 2.4 [2.3-3, 8] and p = 0.16, OD2: 522 [402-623] vs 503 [433-666] and p = 0.17. Hematological parameters increased (p <10^-3^ for all) with a change in Hb (g / dl) from 6.6 [6.2-6.9] to 7.9 [7.02 -8.4], hematocrit (%) of [19-22] to 24 [22-25] and GR count (106 / ml) of 2.47 [2.14-2.75] to 2, 9 [2.49 to 3.22].

**Conclusions:**

Our results tend to confirm that, despite the improvement of CO2 after ET, DO2 does not improve (ultimate goal of transfusion!). This effect seems to be due to the decrease of the CO consequent to the increase of blood viscosity.

## P308 Local audit of blood transfusion practices in comparison with NICE blood transfusion guidelines 2015

### MH Sultan

#### Lahore General Hospital, Lahore, Pakistan, Department of Internal Medicine, Lahore, Pakistan

**Introduction:**

This audit was carried out to objectively inspect flaws in technique of blood transfusion in both medical emergency and internal medicine wards.

**Methods:**

A questionnaire was designed with reference to NICE blood transfusion guidelines 2015 and data was collected from 102 House Officers and Post-graduate Residents after permission from Head of Departments of Internal Medicine. Personal help was available to them while they filled the questionnaire.

**Results:**

Information regarding reason for transfusion and risks and benefits of blood transfusion is conveyed by 80 (78.4%) of doctors. Transfusion process is explained by 56 (54.9%) of doctors and only 44 (43.1%) doctors talk about any transfusion needs that are specific to patients. 29 (28.4%) of doctors guide about any alternatives to blood transfusion if available and 20 (19.6%) tell patients that they are no longer eligible to donate blood. 21(20.6%) encouraged their patients to ask questions. Regarding documentation, 37 (36.3%) doctors do not document this discussion in patient’s notes. Also, 11(10.8%) of doctors do not monitor patient’s condition and vitals before, 16 (15.7%) during and 39 (38.2%) after transfusion. 32 (31.4%) doctors said that they had formal training for blood transfusion, 60 (58.8%) said that they do not have that, while 10 (9.8%) said that they don’t know if they had any formal training for this purpose or not (Figure 1).

**Conclusions:**

1) Documented consent, vitals before, during and after transfusion, fresh laboratory investigations after transfusion should be made an integral part of process.

2) Workshops, audits and brief one-to-one meetings with physicians and installation of algorithms and guidelines should be done.

**Fig. 1 (abstract P308).**
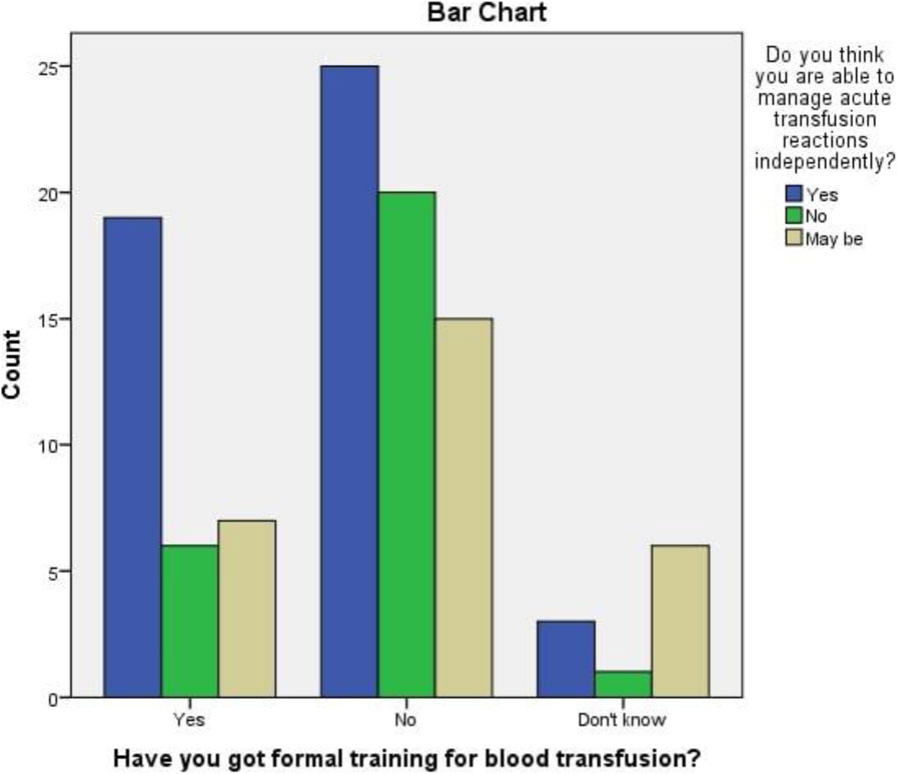
Compares the relationship of formal training of blood transfusion with the ability to manage acute transfusion reactions independently among junior doctors

## P309 A systematic review and meta-analysis exploring the safety and efficacy of erythropoiesis- stimulating agents in critically ill patients

### E Litton^1^, P Latham^2^, J Inman^3^, J Luo^4^, P Allan^5^

#### ^1^SJOG Hospital Subiaco, ICU, Perth, Australia; ^2^Noosa hospital, ICU, Sunshine Coast, Australia; ^3^Fiona Stanley Hospital, Anaesthesia, Murdoch, Australia; ^4^Rockingham General Hospital, Anaesthetics, Rockingham, Australia, ^5^Fiona Stanley Hospital, ICU, Murdoch, Australia

**Introduction:**

Severe immune dysregulation is associated with adverse outcomes and is common in intensive care unit (ICU) patients [1]. Erythropoietin-stimulating agents (ESAs) have both anti-apoptotic and immune-modulating properties [2]. Despite potential benefit, both the safety and efficacy of these agents remains unclear [3]. Here we evaluate the impact of ESAs on morality at hospital discharge in critically unwell adult patients admitted to the ICU.

**Methods:**

We conducted our search strategy in accordance with a predetermined protocol. We searched OVID MEDLINE, OVID EMBASE and The Cochrane Central Register of Controlled Trials, from inception until May 2019. Eligible publications were randomized controlled trials (RCTs) reporting mortality, including adult patients admitted to an ICU, with both a group receiving ESA therapy and a comparator group not receiving ESA therapy with both groups reporting mortality. No language restrictions applied.

**Results:**

16 of the 21 included studies reported in-hospital mortality, with evidence of a decrease in the group receiving ESA therapy (276 of 2187 patients, 12.6%) when compared with the comparator group (339 out of 2204 patients, 15.4%), [relative risk (RR) 0.82, 95% CI 0.71–0.94, *P* = 0.006*, I*2 = 0.0%]. Of these studies only one was categorized as being of low risk of bias.

**Conclusions:**

We have demonstrated that in heterogenous populations of critically ill adults, ESA therapy may decrease mortality. The evidence used in this trial was mainly of low or uncertain quality.

**References:**

1 Muszynski JA et al. Curr Opin Pediatr 28:267-273, 2016.

2 Nairz M et al. Microbes Infec 4:238-46, 2012

3 Corwin HL et al. N Engl J Med 357: 965–76, 2007

## P310 Safety and efficacy of prothrombin complex concentrate in PPH

### A Ronenson^1^, E Shifman^2^, A Kulikov^3^

#### ^1^Tver Regional Perinatal Center, Anesthesia and Intensive care, Tver, Russia; ^2^Moscow Regional Research Clinical Institute named M.F. Vladimirsky, Anesthesia and Intensive care, Moscow, Russia; ^3^Ural State Medical University, Anesthesia and Intensive care, Yekaterinburg, Russia

**Introduction:**

The use of FFP is associated with an increased incidence of complications such as acute respiratory distress and infections, and the rate of complications increased with the quantities of FFP transfused [1]. PCC contain several important coagulation factors and it has been suggested that they could replace FFP. This has been shown mainly in case reports or series in which coagulation factor deficit was detected by using POC viscoelastic tests in trauma [2] or traditional hemostatic tests in obstetric patients [3].

**Methods:**

Multicenter observational study of the safety and efficacy of the prothrombin complex concentrate. A survey of anesthetists was conducted in 19 maternity hospitals at various levels of care in the Russian Federation. Data has been collected and processed. As a result, 251 patients were analyzed. PPH was determined as a volume of blood loss more than 500 ml during vaginal delivery or CS.

**Results:**

The most significant risk factors for PPH were: preeclampsia or arterial hypertension and a history of postpartum hemorrhage. 32.3% had no risk factors for PPH. It was determined that the use of Prothromplex 600 IU decreased the number of patients with transfusion FFP 12-15 ml/kg by 27.8% and increased the number of patients without transfusion by 25.9%, compared with patients without use of Prothromplex 600 IU (Figure 1). No complications were detected.

**Conclusions:**

The use of PCC safety and efficacy reduce use of FFP during PPH.

**References:**

1. Watson GA et al. J Trauma 67:221–7, 2009

2. Schöchl H, et al. Crit Care 14:R55, 2010

3. Glynn JC et al. Anaesthesia 62:202–3, 2007

**Fig. 1 (abstract P310).**
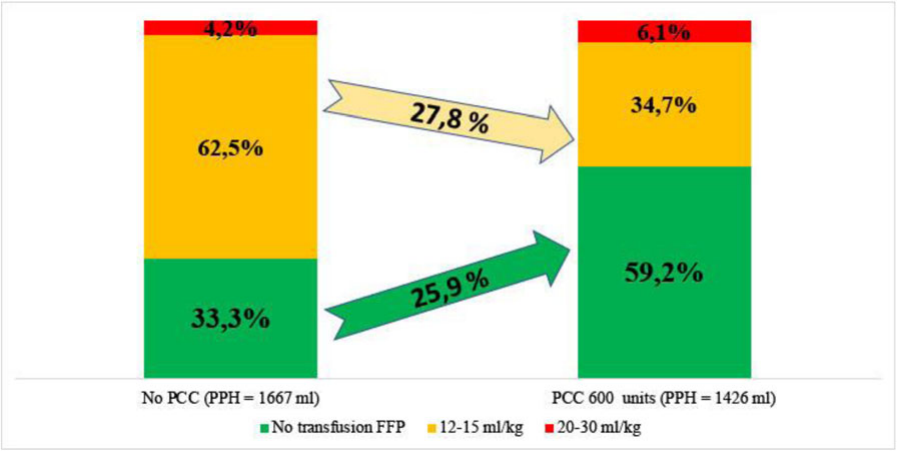
Use of Prothromplex and a decrease transfusion of FFP

## P311 The effect of intravenously given vitamin K1 to critically ill patients with spontaneously increased prothrombin time

### S Dahlberg^1^, U Schött^1^, T Kander^2^

#### ^1^Skåne University Hospital, Lund, Sweden; ^2^Skåne University Hospital, Anaesthesia and Intensive Care, Lund, Sweden

**Introduction:**

Previous studies indicate vitamin K-deficiency to be common in critically ill patients without overt bleeding. The aim of this study was to evaluate the effect of intravenously given fytomenadion on routine coagulation status, activities of vitamin K- dependent coagulation factors, thrombin generation and thromboelastography.

**Methods:**

Critically ill patients with prolonged prothrombin time (PT) – Owren (PT-INR) >1.2) were included during office hours. Routine coagulation status (Owren PT, Quick PT), activity of coagulation factor (F) II, FVII, FIX, FX, protein C, protein S, thrombin generation (TGA) and thromboelastography (ROTEM), were measured before and 24 hours after 10 mg fytomenadion had been given intravenously. The exclusion criteria were on-going treatment with warfarin- or NOACs, hepatocellular cancer, liver resection <6 months, known coagulopathy or treatment with fytomenadion <36 hours.

**Results:**

27 patients were included. A significant decrease of Owren PT and Quick PT was demonstrated 24 hours after given fytomenadion (p<0.001), Figure 1. The activity of FII, FVII, FIX and FX was increased (p<0.01, p<0.01, p<0.01, p<0.001). The activity of protein C, protein S and TGA were unchanged. No changes were demonstrated in ROTEM, except for maximal clot formation (MCF), indicating increased clot strength. The sensitivity analysis demonstrated that patients with higher Owren PT>1.3 at baseline showed a stronger response to fytomenadion, including increased TGA.

**Conclusions:**

PT decreases and the activity of FII, FVII, FIX and FX increases 24 hours after intravenously given fytomenadion in critically ill patients. In patients with more pronounced PT increase at baseline, these effects were even stronger and also included increased TGA.

**Fig. 1 (abstract P311).**
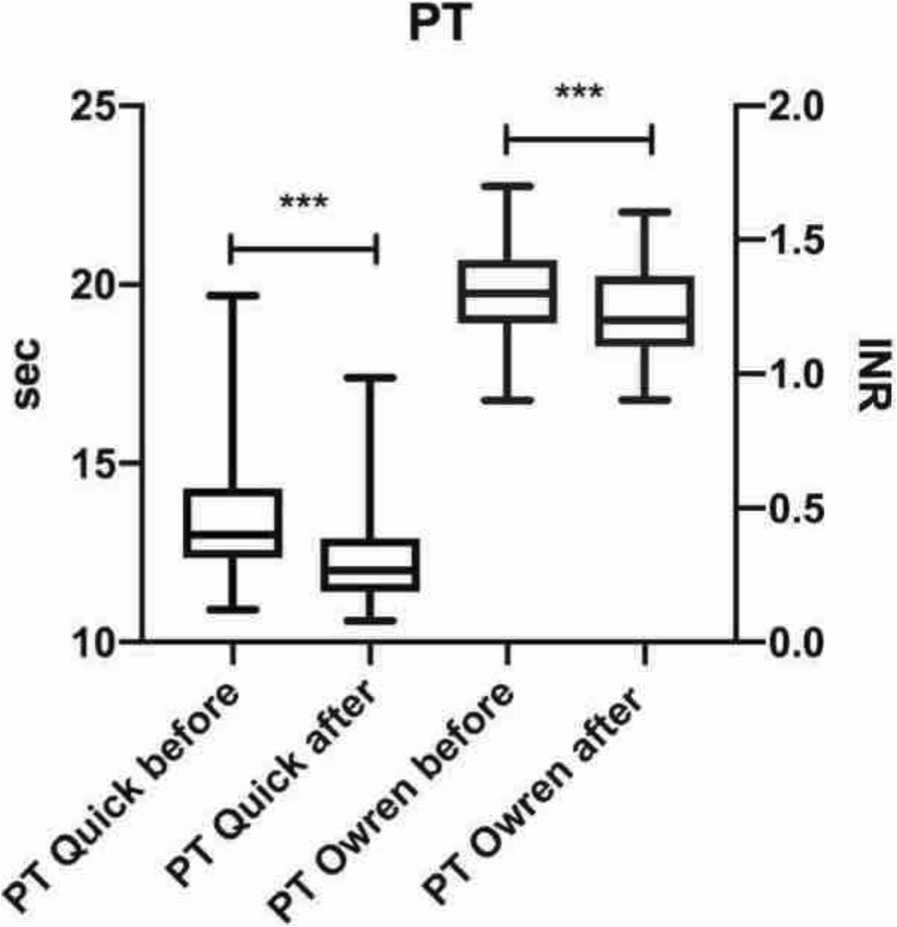
PT before and 24 hours after the administration of 10 mg fytomenadion given intravenously. Box-plots with min-max whiskers. PT= prothrombin time. ***p<0.001

## P312 Comparison of coagulation parameters associated with fibrinogen concentrate and cryoprecipitate in the treatment of bleeding in pseudomyxoma peritonei surgery: results from the prospective, randomized, controlled phase 2 FORMA-05 study

### A Roy^1^, N Sargant^1^, S Rangarajan^1^, S Alves^1^, J Bell^1^, S Stanford^1^, C Solomon^2^, I Kruzhkova^3^, S Knaub^2^, F Mohamed^1^

#### ^1^Basingstoke and North Hampshire Hospital, Hampshire, United Kingdom; ^2^Octapharma, Lachen, Switzerland; ^3^Octapharma, Clinical R&D, Lachen, Switzerland

**Introduction:**

The FORMA-05 study compared hemostatic efficacy and safety of human fibrinogen concentrate (HFC) vs cryoprecipitate for bleeding patients with acquired fibrinogen deficiency undergoing cytoreductive surgery (CRS) for pseudomyxoma peritonei (PMP). Maintaining adequate levels of coagulation proteins, including plasma fibrinogen concentration, during CRS are important to help control hemostasis.

**Methods:**

FORMA-05 was a single-center, prospective, randomized, controlled Phase 2 study. Patients undergoing PMP surgery with predicted intraoperative blood loss ≥2 L received HFC (4 g) or cryoprecipitate (2 pools of 5 units, approximately 4.0–4.6g fibrinogen), repeated as needed. Plasma fibrinogen concentration (measured using Clauss assay) and FIBTEM A20 (measured using thromboelastometry) were measured hourly intraoperatively, while Factor (F) XIII, FVIII, von Willebrand Factor (VWF) levels, standard laboratory tests and endogenous thrombin potential (ETP) were measured every two hours. Post-surgery, all parameters were measured at 6, 12, 24, and 28 hours, and 10 days.

**Results:**

The full analysis included 45 patients on either HFC (n=22) or cryoprecipitate (n=23). The intraoperative and postoperative changes in ETP and fibrinogen concentration are shown in Table 1. For FIBTEM A20 (intraoperatively) and fibrinogen concentration (intraoperatively and postoperatively), the mean numerical values appeared higher with HFC than cryoprecipitate. FXIII (HFC: 121.86%, 66.85%; cryoprecipitate: 115.55%, 68.68%, at baseline and 4hr after surgery start), FVIII and VWF were maintained throughout surgery in both treatment groups. This was also the case for laboratory tests activated partial thromboplastin time, prothrombin time and platelet count.

**Conclusions:**

The FORMA-05 coagulation parameters analyses showed broad overlaps between HFC and cryoprecipitate, with satisfactory maintenance of the clot quality parameters, FXIII concentrations and thrombin generation parameters.

**Table 1 (abstract P312).** The intraoperative and postoperative changes in ETP and fibrinogen concentration. HFC, human fibrinogen concentrate; SD, standard deviation

Parameter	Endogenous thrombin potential (nmol/L/min)	Endogenous thrombin potential (nmol/L/min)	Plasma fibrinogen concentration (g/L)	Plasma fibrinogen concentration (g/L)
Treatment group	HFC	Cryoprecipitate	HFC	Cryprecipitate
Baseline mean	1514.5 (430.6)	1639.1 (339.1)	4.79 (1.316)	4.50 (1.497)
Interoperative, 2hr after surgery start, mean (SD)	1673.5 (340.1)	1690.2 (320.2)	3.20 (0.722)	2.67 (0.984)
Interoperative, 6hr after surgery start, mean (SD)	1310.7 (171.2)	1260.7 (342.2)	2.34 (0.610)	1.91 (0.543)
Postoperative end-of-surgery, mean (SD)	1225.4 (220.7)	1346.4 (243.6)	2.21 (0.515)	2.08 (0.562)
2 days after surgery end, mean (SD)	1263.44 (176.9)	1385.6 (301.6)	5.47 (0.832)	5.24 (0.818)
10 days after surgery end, mean (SD)	1426.16 (260.4)	1357.0 (349.5)	6.62 (1.484)	6.48 (1.685)

## P313 Estimation of fibrinogen deficit based on base excess and lactate level at the patient with multiple trauma

### A Baetu, IC Grintescu, C Cobilinschi, M Tiglis, AM Cotae, M Buiuc, E Ciobanu, IM Grintescu

#### Emergency Clinical Hospital Bucharest, Anesthesia and Intensive Care Unit, Bucharest, Romania

**Introduction:**

Estimation of fibrinogen level by classical laboratory methods or by viscoelastic method-rotational thrombelastometry (ROTEM) is essential for the patient with multiple trauma as it plays a key role in hemostasis and is the first coagulation factor to be consumed. We investigated the predictive ability of lactate level and base excess to determine fibrinogen deficiency for the patient with multiple trauma from admission.

**Methods:**

This retrospective study includes 158 patients admitted to the Emergency Clinical Hospital of Bucharest which meet the Berlin criteria for the diagnosis of multiple trauma. There were taken into account: the level of lactate, base excess, and Maximum Clot Formation(MCF/FibTEM-ROTEM) from the admission of patients in hospital. For statistical analysis we used MedCalc14.1.

**Results:**

The study group includes 118 men and 40 women with a mean age of 43,008 vs. 40.32 years (p=0.639) admitted with the diagnosis of multiple trauma. We found a directly proportional and highly significant statistical correlation between base excess and fibrinogen level diagnosed using the MCF/FibTEM parameter(r=0.6382, p<0.0001)and an inverse proportional correlation between lactate level and fibrinogen level (r= -0.2164, p=0.0065). In the ROC analysis that uses as a variable the level of base excess and as a criterion of classification the fibrinogen deficit (MCF/FibTEM<12 mm) it can be observed that at a value of BE<-7 mmol/l, we can diagnose a fibrinogen deficit with a sensitivity of 88.2% and a specificity of 80.6% (AUC= 0.872,p<0.0001). Lactate appears to be inferior to the excess base (Figure 1), but still has a good diagnostic power, a value of 2.6 mmol/l has a sensitivity of 67.1% and a specificity of 75% (AUC= 0.754,p<0.0001). The difference between the two ROC curves (0.118) is statistically significant (p = 0.0028).

**Conclusions:**

Both base excess and serum lactate can be used to diagnose fibrinogen deficiency with the mention that base excess appears to have a higher sensibility and specificity ability**.**

**Fig. 1 (abstract P313).**
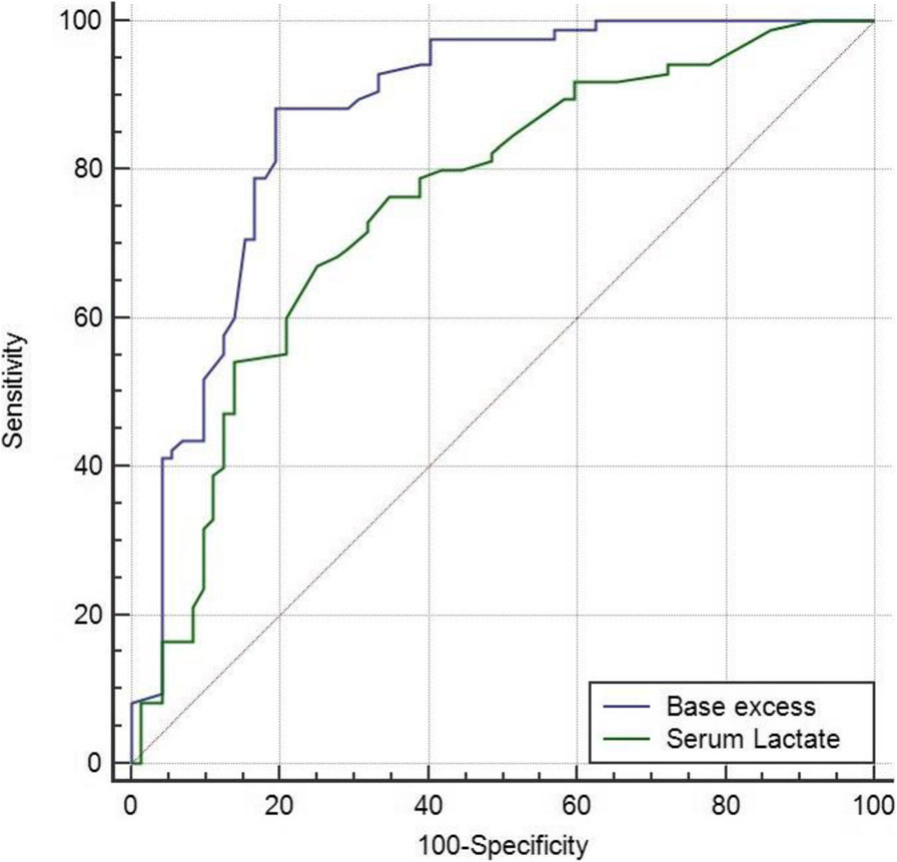
Comparison of ROC curves of Base excess (AUC=0.872) vs Lactate level (AUC=0.754) for diagnosis of fibrinogen deficiency

## P314 Fibrinogen-based vs plasma-based therapy for trauma-induced coagulopathy: implications on blood transfusion

### GN Jesus^1^, S Cunha^2^, L Carneiro^3^, J Galacho^3^, SM Fernandes^1^

#### ^1^Centro Hospitalar Universitário Lisboa Norte, Serviço de Medicina Intensiva, Lisboa, Portugal; ^2^Faculdade de Medicina da Universidade de Lisboa, Lisboa, Portugal; ^3^Centro Hospitalar Universitário Lisboa Norte, Lisboa, Portugal

**Introduction:**

Coagulopathy is the leading cause of preventable death in patients with severe trauma. A better understanding on the role of hypofibrinogenemia has lead to an increase in fibrinogen administration, leading to shift from fixed-ratio transfusion protocol to a viscoelastic-based goal-directed algorithm. This approach requires further clinical validation.

**Methods:**

We conducted a retrospective study comparing transfusion strategies in patients with major trauma between 2013 and 2018. We retrieved demographic data and blood products administered from patients with at least one red-blood cell (RBC) transfusion. Primary outcome was a reduction of RBC administration. Secondary outcomes were mortality, ICU length of stay and acute kidney injury.

**Results:**

We included 141 patients admitted in the ICU due to severe trauma (SAPSII:41.5 ±21.9), and mainly after emergent surgery (68.8%). They featured a mean age of 45.3±19.3y, were predominantly male (76.6%) and 73% were in shock. In the first 24 hours of hospital admission a mean of 3.6±4.5 RBC units were administered. Most patients received a fibrinogen-based protocol (FBP) (78%), with an average of 5±3g of fibrinogen and 1±3 fresh-frozen plasma (FFP) units, versus 3±4 g of fibrinogen and 6±4 FFP units in the FFP group. The FBP was associated with a decrease administration of RBCs in the first 24 hours (R = -2.6; p < 0.004), even after adjustment for severity (p=0.003) and for tranexamic acid use (p = 0.003). It was associated also with a decrease of platelet transfusion (p=0.004). Fibrinogen-based protocol was not associated with a decrease in mortality, acute kidney injury or noradrenaline dose.

**Conclusions:**

Treatment of TIC in past years has progressively changed to a goal-directed fibrinogen-based approach. In our population, the use of FBP lead to a reduction of RBC administration in severe trauma patients.

## P315 Prospective, multicenter, randomized study comparing administration of clotting factor concentrates with a standard massive hemorrhage protocol in severely bleeding trauma patients

### L Da Luz^1^, J Callum^2^, A Beckett^3^, H Peng^4^, P Engels^5^, N Parry^6^, H Tien^1^, A Nathens^7^, B Schwartz^8^, K Karkouti^9^

#### ^1^2075 Bayview Avenue, Dept of Surgery, Toronto, Canada; ^2^2075 Bayview Avenue, Transfusion Medicine, Toronto, Canada; ^3^Saint Mchael`s Hospital, Dept of Surgery, Toronto, Canada; ^4^Defence Research and Development Canada, Toronto Research Center, Defence Research and Development Canada, Toronto Research Center, Toronto, Canada; ^5^Hamilton General Hospital, Dept of Surgery, Hamilton, Canada; ^6^London Health Sciences Centre, Dept of Surgery, London, Canada; ^7^2075 Bayview Avenue, Toronto, Canada; ^8^Clinical Research & Development, Octapharma, Clinical Research & Development, Octapharma, Hoboken, USA, Hoboken, United States; ^9^University Health Network, Sinai Health System, and Women’s College Hospital, Dept of Anesthesia and Pain Management, Toronto, Canada

**Introduction:**

The FiiRST-2 study will investigate if fibrinogen concentrate (FC) + prothrombin complex concentrate (PCC) administered within the first hour of hospital arrival is superior to the current standard of care: blood component therapy via a massive hemorrhage protocol (MHP) in trauma patients. Hemorrhaging trauma patients may develop acute trauma coagulopathy, which has multifactorial etiology. However, acquired fibrinogen deficiency and impaired thrombin generation are becoming recognized as major drivers. Prompt and targeted coagulation factor replacement with FC and PCC may be superior to standard blood component therapy.

**Methods:**

FiiRST-2 is a multicenter (8 Canadian centers), pragmatic, randomized, parallel-control, superiority trial with an adaptive two-stage design. Trauma patients >16 years old at risk of massive hemorrhage will be randomized to receive FC + PCC or standard of care (1:1:1 red cell [RBC]:plasma:platelet transfusion) until after the second MHP pack has been administered, MHP is terminated, or 24 h from admission to the trauma bay (Figure 1). Exclusion criteria include receiving >2 U RBCs within 1 h post-admission, ≥3 h elapsed from injury, severe traumatic brain injury, or known congenital or acquired bleeding disorders. The primary endpoint is to demonstrate superiority with respect to the number of allogeneic blood product units (RBCs + plasma + platelets) transfused from admission to 24 h after admission. Secondary endpoints include RBC units transfused from admission to 24 h after as a surrogate for hemorrhage control. Safety endpoints include AEs and SAEs during 28 days after admission.

**Results:**

The study is expected to end Q1 2022.

**Conclusions:**

This pragmatic multicenter trial will determine if early hemostatic therapy with FC + PCC is superior to standard MHP packs in bleeding trauma patients. Results could have a major impact on clinical practice and improve the management and outcomes of this high-risk group of patients.

**Fig. 1 (abstract 315).**
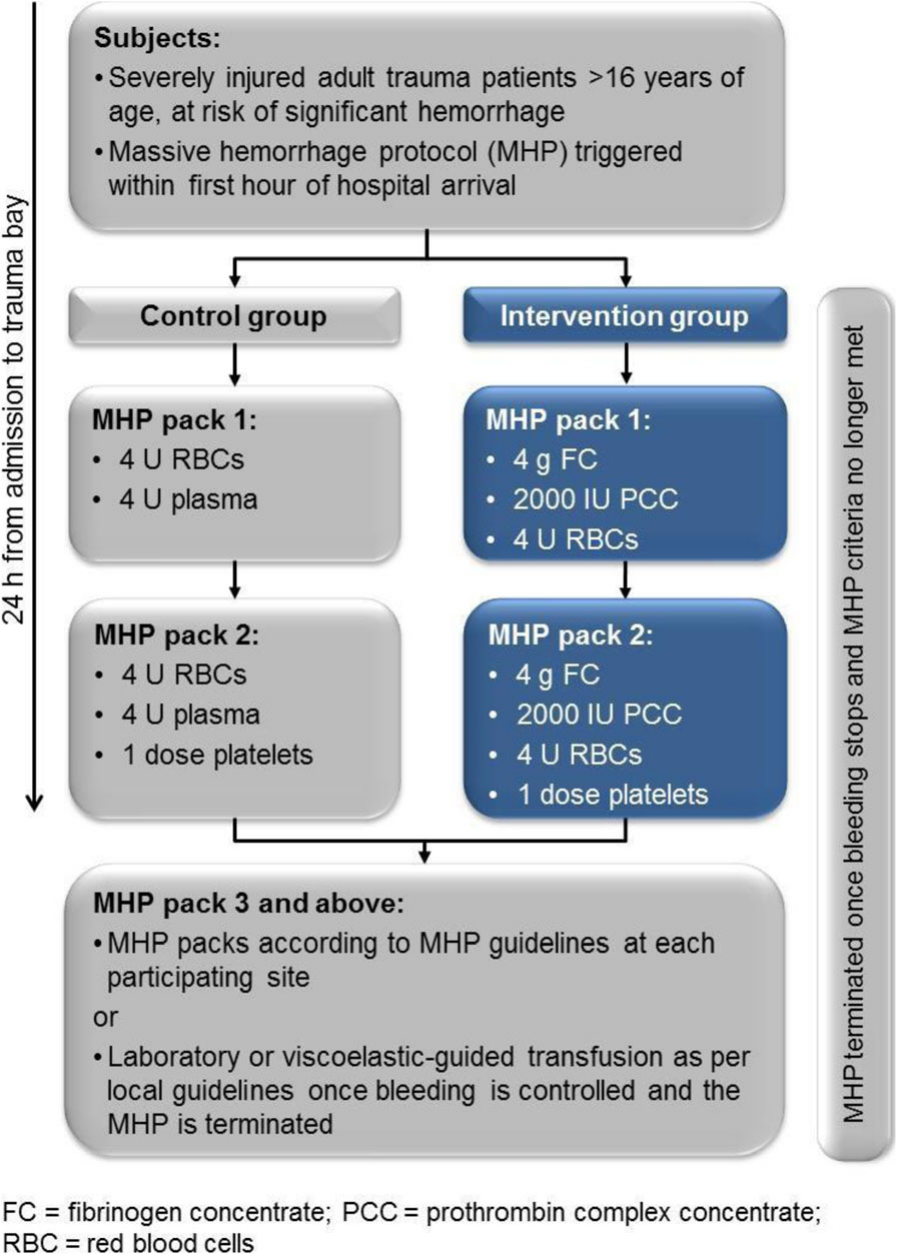
Study Treatment Plan

## P316 Trauma-induced coagulopathy detected by the Quantra® QStat® system

### E Michelson^1^, M Cripps^2^, B Ray^3^, D Winegar^4^, F Viola^5^

#### ^1^Texas Tech University Health Sciences Center El Paso, Department of Emergency Medicine, El Paso, United States; ^2^University of Texas Southwestern, Surgery, Dallas, United States; ^3^University Medical Center of El Paso, Patient Blood Management, El Paso, United States; ^4^HemoSonics, LLC, Clinical Affairs, Charlottesville, United States; ^5^HemoSonics, LLC, Chief Scientific Officer, Charlottesville, United States

**Introduction:**

The objective of this study was to assess the ability of the Quantra**®** QStat**®** System (HemoSonics) to detect coagulopathies in trauma patients. Many Level 1 trauma centers have adopted whole blood viscoelastic testing, such as rotational thromboelastometry (ROTEM®, Instrumentation Lab) for directing transfusion therapy in bleeding patients. The Quantra QStat System is a cartridge-based point-of-care (POC) device that uses ultrasound to measure viscoelastic properties of whole blood. and provides measures of clot time, clot stiffness and a test of fibrinolytic function.

**Methods:**

Adult subjects were enrolled at two Level 1 trauma centers which use a ROTEM based protocol to guide transfusion decisions. Study protocols were approved by the site’s Ethics Committee. For each subject, whole blood samples were drawn upon arrival to the emergency department and again, in some cases, after administration of blood products or antifibrinolytics. Samples were analyzed on the Quantra (at POC) in parallel to ROTEM delta (in lab).

**Results:**

A total of 54 patients were analyzed. Approximately 42% of samples had a low Clot Stiffness (CS) values suggestive of an hypocoagulable state. The low stiffness values could be attributed to either low platelet contribution (PCS), low fibrinogen contribution (FCS), or a combination (Figure 1). Additionally, 12% of samples showed evidence of hyperfibrinolysis based on the Quantra Clot Stability to Lysis parameter. Samples analyzed on standard ROTEM assays showed a lower prevalence of low clot stiffness and fibrinolysis based on EXTEM, FIBTEM results. The correlation of CS and FCS vs equivalent ROTEM parameters was strong with r-values of 0.83 and 0.78, respectively.

**Conclusions:**

This first clinical experience with the Quantra in trauma patients showed that the QStat Cartridge detected coagulopathies associated with critical bleeding and may be useful for directing blood product transfusions in these patients. Ability to perform testing at POC may provide additional clinical advantage.

**Fig. 1 (abstract P316).**
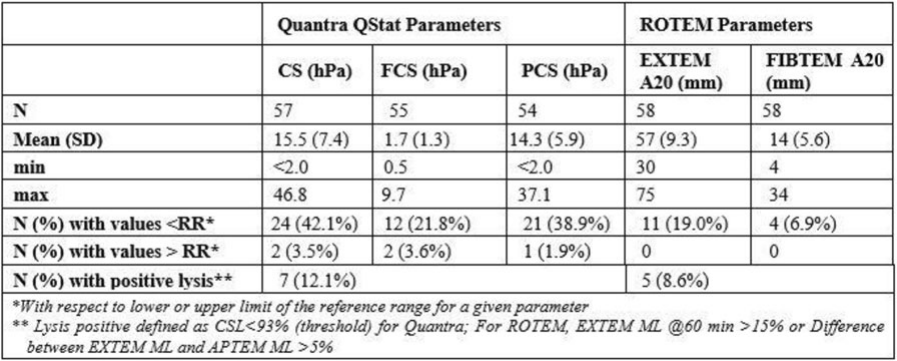
Summary of clot stiffness parameters

## P317 First real-world experiences with a new human fibrinogen (FIBRYGA®) in France based on temporary authorization

### F Stéphan^1^, L Gutermann^2^, M Pennetier^3^, S Bourget^4^, S Djabarouti^5^, J Berdugo^6^, Y Fardini^7^, P Clerson^7^, C Belmokhtar^8^, G Hébert^2^

#### ^1^Hôpital Marie Lannelongue, Unité de soin intensif, Le Plessis Robinson, France; ^2^Hôpital Marie Lannelongue, Service Pharmacie et Stérilisation, Le Plessis Robinson, France; ^3^Hôtel Dieu, Pharmacie Clinique, Nantes, France; ^4^Centre Hospitalier de Valence, Service Pharmacie, Valence, France; ^5^Groupe Hospitalier Sud, CHU de Bordeaux, Service Pharmacie, Pessac, France; ^6^Hôpital Saint-Joseph, Service Pharmacie, Marseille, France; ^7^Soladis Clinical Studies, Roubaix, France, ^8^Octapharma France, Boulogne-Billancourt, France

**Introduction:**

The objective of the study was to describe the conditions of use of FIBRYGA® 1g, a new, highly purified, human fibrinogen (HF) recently granted a temporary import authorization for use in congenital and acquired fibrinogen deficiencies in France.

**Methods:**

Observational, non-interventional, non-comparative, retrospective study conducted in 5 French hospital centres using FIBRYGA®. Data from patients with fibrinogen deficiency having received FIBRYGA® from December 2017 to July 2019 were retrieved from their medical files. Indications, modalities, efficacy and safety outcomes were recorded. Indications encompassed non-surgical bleeding (NSB) either spontaneous or traumatic, including post-partum hemorrhage (PPH), bleeding during surgery (SB) or administration to prevent bleeding during planned surgery. Treatment success was defined as control of the bleeding or hemoglobin loss <20% for bleeding treatment and as absence of major perioperative hemorrhage for pre-surgical prevention.

**Results:**

This analysis included 110 patients aged 56,7 ± 17.7 years and 60% were male. All presented an acquired fibrinogen deficiency requiring administration of HF. Indications were NSB (n=45, 40.9%) including 15 (13.6%) PPH, SB (n=31, 28.2%), and prevention of SB (n=34; 30,9%). Cardiac surgeries were the main procedures associated with treatment and prevention of SB. Mean total doses of FC were 2.95±1.66g, 2.00±1.37g and 2.21±1.23g for NSB, SB and prevention of SB. Success rates were 88.4% (95%CI 78.8-98.0%), 96.8% (95%CI 90.6-100%) and 91.2% (95%CI 81.6-100%) respectively. For PPH, mean dose of HF was 2.53±0.74g with a success rate of 86.7% (95%CI 69.5-100%). Overall, tolerance was good.

**Conclusions:**

Fibrinogen concentrate FIBRYGA® is mostly used for bleeding control. In one third of patients, HF was administered preventively to avoid bleeding during surgery. Use of FIBRYGA® was associated with favourable efficacy outcomes.

## P318 Functional testing for tranexamic acid effect duration using modified viscoelastometry

### T Kammerer^1^, P Groene^2^, S Sappel^2^, P Scheiermann^2^, ST Schaefer^2^

#### ^1^Ruhr-University Bochum, Institute of Anaesthesiology, Heart and Diabetes Center NRW, Bad Oeynhausen, Germany; ^2^Ludwig-Maximilans University, Department of Anaesthesiology, Munich, Germany

**Introduction:**

Tranexamic acid (TXA) is the gold standard to prevent or treat hyperfibrinolysis [1]. Effective plasma concentrations are still under discussion [2]. In this prospective, observational trial using modified viscoelastometry we evaluated the time-course of the antifibrinolytic activity of TXA in patients undergoing cardiac surgery.

**Methods:**

25 patients were included. Modified viscoelastometry (TPA-test) was performed and TXA-plasma-concentration, plasminogen-activator-inhibitor-1 (PAI-1) and PAI-antigen-plasma-concentrations were measured over 96h. Additionally, in vitro dose-effect-curves from blood of healthy volunteers were performed. Data presented as median with interquartile range (Q1/Q3).

**Results:**

TXA plasma-concentration was increased compared to baseline (T1:0 μg ml^-1^) at every time-point with a peak concentration 30min (T2) after application (p<0.0001; see Fig.1A). Lysis was inhibited from 30min (LysisTime_TPA-test_: p<0.01; LysisOnsetTime_TPA-test_:p<0.0001). MaximumLysis_TPA-test_ was decreased at T2 (T1:97% (96/97) vs. T2: 9% (6/11); p<0.0001). Of note, after 24h some patients (n=17) had normalized lysis whereas others (n=8) had strong lysis inhibition (ML<30%;p<0.05) up to 96h. High and low lysis groups differed regarding kidney function (cystatin C:1.64mg l^-1^(1.42/2.02) vs. 1.28mg l^-1^(1.01/1.52);p=0.002) and active PAI-1 (93.05ng ml^-1^ (33.15/9100.0) vs. 16.13ng ml^-1^ (6.62/79.98);p=0.047). In-vitro, TXA concentrations >10μg ml^-1^ were effective to inhibit fibrinolysis.

**Conclusions:**

In our trial, after 24h there was still completely blocked lysis in patients with moderate renal impairment. This could be critical with respect to postoperative thromboembolic events [3]. Here modified viscoelastometry could be helpful to detect the individual fibrinolytic capacity.

**References:**

1. Myles PS et al. NEJM 376:1893, 2017

2. Jerath A et al. Anesth Analg. 127:1323–32, 2018

3. Chornenki NLJ et al. Thromb Res. 179:81–6, 2019

**Fig. 1 (abstract P318).**
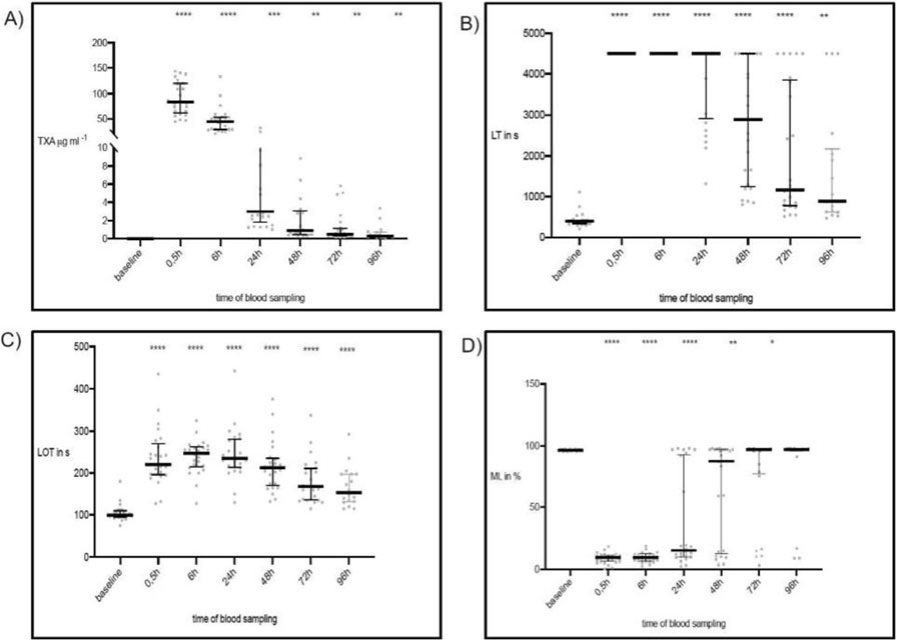
Viscoelastometric variables (TPA-test) (median + IQR; n=25) over time. A) Tranexamic acid plasma concentrations (TXA). B) Lysis Time = time span between CT and 50% lysis. C) Lysis Onset Time = time span from ClottingTime to 15% lysis. D) Maximum Lysis = difference between MCF and lowest amplitude in % of MCF;* p<0.05 vs. baseline (before tranexamic acid (TXA)); ** p<0.01 vs. baseline; *** p<0.001 vs. baseline; **** p<0.0001 vs. baseline

## P319 Fibrinogen concentrate and ROTEM-guided hemostasis management are associated with less bleeding and transfusion in surgery for thoracic aortic dissection

### M Stefan, I Marinica, A Paunescu, M Luchian, A Stegaru, A Dumitrescu, D Filipescu

#### "Prof Dr CC Iliescu" Emergency Institute for Cardiovascular Diseases, ICU II, Bucuresti, Romania

**Introduction:**

Peri-operative coagulopathy correction based on viscoelastic hemostatic assays (VHAs) and single-factor coagulation products has changed the paradigm of bleeding management in cardiac surgery [1].

**Methods:**

In a retrospective study, we analysed patients with emergency surgery for thoracic acute aortic dissection (TAAD), before and after the introduction of fibrinogen concentrate in clinical practice. Data were collected from paper and electronic records. The study was approved by the institutional ethical committee. 60 patients were included in the analysis, 19 operated in 2012, before fibrinogen concentrate was approved for human use, and 41 in 2018-2019. Therapy was guided by a rotational thrombo-elastometry (ROTEM) algorithm. Exclusion criteria were non-compliance with the institutional protocol and intra-operative death. We investigated allogeneic blood transfusion (ABT), fibrinogen use, peri-operative bleeding (POB), surgical re-exploration and post-operative complications (POC).

**Results:**

The groups were similar in gender, age, body weight, additive EuroScore and aortic cross-clamp time. Fresh frozen plasma, cryoprecipitate and red blood cell transfusion were lower in the fibrinogen group, but not platelet transfusion (table). 48,7% of patients in the study group received fibrinogen concentrate and median dose was 2 g (IQR 2-3). Day 1 postoperative chest tube drainage and surgical re-exploration were significantly lower. There were no differences in stroke, renal replacement therapy, mechanical ventilation time and ICU stay.

**Conclusions:**

In patients with TAAD surgery, ROTEM-guided algorithms which include fibrinogen concentrate are associated with less (POB), surgical re-exploration and ABT. Further research is needed to document the role of VHAs and concentrated factors in reducing (POC).

**References:**

1. Meco M et al. J Cardiothorac Vasc Anesth 34:119-12, 2020

**Table 1 (abstract P319).** Peri-operative transfusion and bleeding

	Fibrinogen group (n= 41)	Control group (n= 19)	p-value
PRBCs - patients transfused (n, %)	35 (85,4%)	18 (94,7%)	
PRBCs - units per patient (median, IQR)	4 (1-6)	5 (3-10)	0,07
FFP - patients transfused (n, %)	28 (68,3%)	19 (100%)	
FFP - units per patient (median, IQR)	4 (0-6)	9 (4-18)	0,001
Cryoprecipitate - units per patient (median, IQR)	0	6 (0-10)	<0,001
Chest tube drainage (ml/24h, median, IQR)	640 (450-1075)	1037 (800-1600)	0,009
Surgical re-exploration (n, %)	2 (4,9%)	6 (31,6%)	0,005

## P320 IFiTEM a novel modality of visco-elastic blood clotting test for ROTEM device

### J Benes^1^, M Peltanova^2^, J Zatloukal^2^

#### ^1^Faculty of Medicne in Plzen, Department of Anesthesiology and Intensive Care Medicine, Plzen, Czech Republic; ^2^Faculty of Medicne in Plzen, Plzen, Czech Republic

**Introduction:**

Viscoelastic methods are currently an indispensable method in the diagnosis of coagulopathy in critically ill. The analysis is carried out using defined reagents of the external (EXTEM), internal pathways (INTEM), fibrinogen (FIBTEM) or methods to diagnose pathological or iatrogenic disorders. Limited channels may decrease time availability under certain circumstances. The combination of reagents could allow for the same information from a smaller amount of examination. We have designed the IFiTEM test, which combines internal pathway activator with a platelet aggregation inhibitor (cytochalazin D).

**Methods:**

Prospective observational study approved by the local ethical board of the University hospital, Plzen in a cohort of 80 patients over 18 years in whom a ROTEM assessment was indicated during routine perioperative or intensive care. Conventional coagulation (serum fibrinogen and platelet count) and routinely performed methods (EXTEM, INTEM, FIBTEM) were examined. The experimental IFiTEM test was performed using a combination of liquid reagents by adding cytacholizin D to the INTEM test. The maximal clot firmness (MCF), the difference of the MCF with and without the added inactivator (dIN=MCF(INTEM)–MCF(IFiTEM); dEX=MCF(EXTEM)–MCF(FIBTEM)) and conventional tests were examined.

**Results:**

Results of the ROTEM and standard laboratory tests are displayed in the Figure 1. A close correlation was observed between the MCF of EXTEM vs. INTEM (r = 0.97) and FIBTEM vs. IFiTEM (r = 0.89). Correlation between MCF(IFiTEM) and fibrinogen serum level (r=0.82) was comparable to the MCF(FIBTEM) (r=0.88). The platelet count showed similar (though lower) correlation with the dIN (r=0.80) and dEX (r=0.80) parameters.

**Conclusions:**

The experimental method IFiTEM could be an alternative of FIBTEM in cases when internal coagulation pathways assessment is prioritized (i.e. heparinized patients on extracorporeal supports).

Acknowledgement

Supported by the program for the Development of Scientific Fields of Charles University (Progres Q39)

**Fig. 1 (abstract P320).**
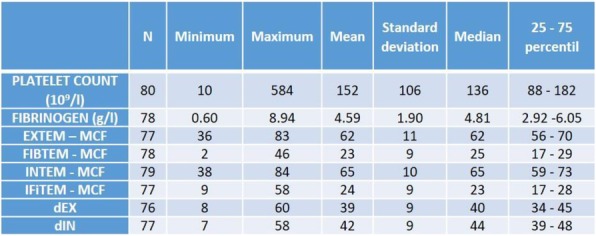
Standard and ROTEM variables

## P321 Factor Xa inhibitor antidote, andexanet alfa differentially reverses the effects of apixaban, betrixaban, edoxaban and rivaroxaban

### F Siddiqui^1^, D Hoppensteadt^1^, W Jeske^1^, A Tafur^2^, K Zorriasateyn^2^, E Ramacciotti^3^, E Bontekoe^1^, J Fareed^1^

#### ^1^Loyola University Medical Center, Pathology, Maywood, United States; ^2^North Shore University Health System, Cardiovascular Institute, Skokie, United States; ^3^Santa Casa School of Medicine, Cardiovascular Institute, São Paulo, Brazil

**Introduction:**

Andexanet alfa (AA, Portola Pharmaceuticals, San Francisco, CA) represents a modified factor Xa agent which is approved antidote for apixaban and rivaroxaban. Andexanet alfa may also neutralize the anti-Xa effects of betrixaban and edoxaban. This study aims to compare the relative neutralization of these four anti-Xa agents by andexanet alfa in different matrices.

**Methods:**

Andexanet alfa was diluted at 10 mg/ml. Apixaban (A), betrixaban (B), edoxaban (E) and rivaroxaban (R) were diluted in pH 8.4, 0.5 M tris buffer (TB), blood bank plasma (BBP) and in 5% albuminated buffer (AB) at 0.062 - 1.0 ug/ml. Anti-Xa activities of all four agents were measured in three systems and the reversibility indices of AA were profiled. The reversibility index (RI_50_) of anti-Xa effects by AA was determined at 25 - 100 ug/ml.

**Results:**

Each of the four agents produced varying degrees of inhibition of anti-Xa at 0.062 – 1.0 ug/ml, the IC_50_ ranged 0.61 - 1.53 ug/ml in BBP, 0.47 - 1.28 ug/ml in AB and 0.49 - 1.4 ug/ml in TB. Andexanet alfa produced a concentration dependent reversal of all four anti-Xa agents. In the BBP, the RI_50_ values for A (192 ug/ml), B (32 ug/ml), E (152 ug/ml) and R (85 ug/ml). In the AB, the RI_50_ values for A (140 ug/ml), B (46 ug/ml), E (176 ug/ml) and R (58 ug/ml). In the TB, the RI_50_ values for A (154 ug/ml), B (79 ug/ml), E (>400 ug/ml) and R (110 ug/ml).

**Conclusions:**

Each of the four anti-Xa agents exhibit varying degrees of matrix independent anti-Xa potencies in different systems, the collective order follows edoxaban > apixaban > betrixaban > rivaroxaban. Andexanet alfa produced matrix dependent differential neutralization of the anti-Xa effects of these agents. Individualized dosing of andexanet alfa may be required to obtain desirable clinical results.

## P322 Correlation between central laboratory and point of care measurements of INR in adult intensive care patients

### D Ernest^1^, D Bhatia^2^, A Pakavakis^1^

#### ^1^Monash Health & Monash University, Intensive Care Unit, Melbourne, Australia; ^2^Monash Health, Critical Care and Perioperative Medicine, Melbourne, Australia

**Introduction:**

Early identification of potential participants for enrolment into the SCARLET study [1] (evaluating ART-123 for sepsis associated coagulopathy) was contingent upon identifying a threshold level of coagulopathy (INR>1.40). Point of Care (PoC) devices measuring INR are commercially available but limited validation studies have been conducted for measuring INR values in intensive care patients. We therefore sought to determine whether INR measurements measured using a PoC device correlate with concurrently measured laboratory (LAB) INR values in adult intensive care patients.

**Methods:**

Using blood already collected for clotting profile measurements as part of clinical management in a convenience sample of adult ICU patients, we concurrently measured INR values using a PoC (Roche CoaguChek Pro II) and LAB (ACLTOP750 Instrumentation Laboratory) devices. The relationship between devices was assessed via correlation, Bland-Altman agreement and diagnostic predictive values.

**Results:**

In 72 independent paired INR measurements using PoC (range 1.0 - 2.4) and LAB (range 0.9 – 1.4) devices in 37 ICU patients (1 - 4 samples/patient) we determined a Pearson correlation coefficient r = 0.76 and Bland-Altman mean difference (bias) 0.01; 95% limits of agreement 0.33 (Figure 1). A PoC INR ≤ 1.2 had a 0.96 negative predictive value for a LAB INR ≥ 1.4 and a PoC INR ≥ 1.3 had a 1.0 positive predictive value for a LAB INR ≥1.4.

**Conclusions:**

PoC device INR measurements correlate relatively well with LAB measurements within this target INR range of interest in adult intensive care patients. The likely value of PoC device INR measurements for future sepsis associated coagulopathy studies requiring a threshold level of coagulopathy would be as a negative predictive tool for screening potential participants.

**References:**

1. Vincent JL et al. JAMA 321:1993–2002, 2019

**Fig. 1 (abstract 322).**
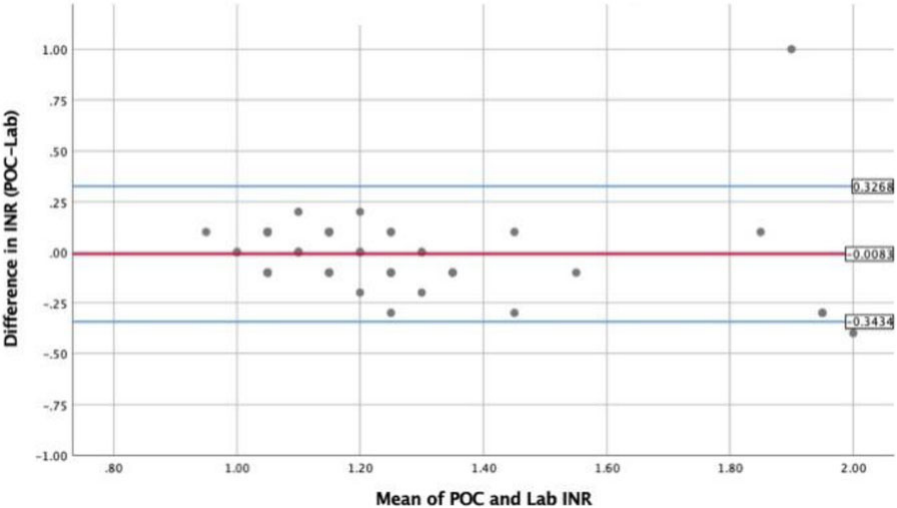
Bland-Altman plot of POC and Lab INR data. Red line - bias (0.01); Blue lines - limits of agreement (0.33)

## P323 MA/R predicts sepsis induced coagulopathy and mortality: a prospective observational study

### X Li

#### Fist affiliated Hospital of China Medical University, ICU, Shenyang, China

**Introduction:**

The diagnostic and prognostic value of thromboelastogram (TEG) in sepsis has not been determined. This study aimed to assess whether TEG is an early predictor of coagulopathy [1, 2] and is associated with mortality in patients with sepsis.

**Methods:**

In total, 518 patients with sepsis on intensive care unit admission were prospectively evaluated. We measured TEG and conventional coagulation tests(CCTs)on preadmission and observed for development of 1, 3 days and 1, 3, 7days respectively. Multivariable logistic regression was utilized to determine odds of ICU/hospital mortality. The parameter of TEG (maximum amplitude, reaction time; MA/R ratio) was calculated to evaluate sepsis-induced coagulopathy. The admission patients were divided into three groupsMA/R0 group(MA/R=5-14mm/min); MA/R1group(MA/R>14 mm/min)and MA/R2 group(MA/R<5mm/min).

**Results:**

410 patients with complete TEG during follow-up were included. At enrollment, 10.73%, 65.85%, and 23.41% of the patients had lower, normal, and higher MA/R state respectively. Compared with MA/R0 group, patients with lower and higher MA/R both had dignificantly increase risk of hospital mortality [HR2.83 (95%CI 1.577-5.079 ), P<0.01]; [HR1.982 (95%CI 1.073-3.66), P=0.029] respectively (adjusted with APACHEII score) and ICU mortality [HR 2.512 (95%CI 1.301-4.852), P= 0.006]; [HR1.644 (95%CI 1.024-2.639), P=0.002](adjusted with APACHEII score). Patients with higher MA/R had dignificantly increase risk of hospital mortality APACHEII score [HR 1.635 (95%CI1.016-2.632), P=0.043].

**Conclusions:**

In our cohort of patients with severe sepsis, coagulopathy defined by MA/R ratio was associated with increased risk of ICU/hospital mortality.

**References:**

1. Gando S et al. Nat Rev Dis Primers 2:16037, 2016.

2. Taylor FB et al. Thromb Haemost 86:1327-30, 2001

## P324 The hemostatic effect and quantification of arterial and venous blood sampling on clot microstructure in sepsis patients: Assessment of a functional biomarker

### S Pillai^1^, G Davies^2^, M Lawrence^2^, J Whitley^2^, PR Williams^3^, K Morris^4^, PA Evans^2^

#### ^1^Welsh Centre for Emergrency Medicine Research, Emergency Department, Swansea, United Kingdom; ^2^Welsh Centre for Emergrency Medicine Research, Swansea, United Kingdom; ^3^Swansea University, Swansea, SA2 8PP, United Kingdom; ^4^Cardiff Metropolitan University, Cardiff- CF5 2YB, United Kingdom

**Introduction:**

Blood sampling for coagulation assessment is often carried out in either arterial or venous samples in the Intensive Care Unit (ICU). There is controversy as to the accuracy of this method due to the inherent differences in physicochemical properties as well as the underlying effects of individual diseases in arterial and venous blood. Clot microstructure has shown to be a new biomarker (fractal dimension- d_f_) which encompasses the effects of diseases in all aspects of the coagulation system [1, 2]. In this study, we compared the effect of all these factors in venous and arterial blood to see if there is a difference in the clot microstructure and quality.

**Methods:**

45 patients admitted to a tertiary intensive care unit and busy teaching hospital were recruited. Arterial and venous blood was sampled from an arterial line and central venous catheter in situ from the same patient. Standard markers of coagulation (PT, aPTT, fibrinogen, full blood count), rotational thromboelastometry (ROTEM), whole blood impedance aggregometry and measured clot microstructure (d_f_) were measured on both arterial and venous samples.

**Results:**

No significant difference was observed in standard laboratory markers, ROTEM and platelet aggregation between arterial and venous blood. There were no differences in the fractal dimension (d_f_) between the arterial and venous blood samples (d_f_ 1.658 ± 0.10 vs 1.654 ± 0.08 respectively, p=0.830).

**Conclusions:**

Samples from patients with critical illness give comparable results from either arterial or venous blood despite their underlying pathophysiological process or treatment. This confirms blood for coagulation testing can be taken from arterial or venous blood.

**References:**

1. Evans et al. Blood 116:3341–3346, 2010.

2. Davies et al. Intensive Care Med 42:1990-1998, 2016.

## P325 The effect of recalcification of citrated blood on clot microstructure using a new biomarker of clot quality

### BL Selwyn^1^, M Lawrence^2^, PR Williams^3^, K Hawkins^4^, D Curtis^4^, K Morris^5^, PA Evans^1^

#### ^1^Welsh Center of Emergency Medicine, Swansea, United Kingdom; ^2^Welsh Center of Emergency Medicine, HBRU, Swansea, United Kingdom; ^3^Swansea University, School of Engineering, Swansea, United Kingdom; ^4^Swansea University, Swansea, United Kingdom; ^5^Cardiff Metropolitan University, Cardiff, United Kingdom

**Introduction:**

Clinicians in the emergency setting use a wide range of hemostatic markers to diagnose and monitor disease and treatment. Current methods rely on the anticoagulant effect of citrate on whole blood prior to laboratory analysis. Despite the well-recognized modulatory effects of citrate on hemostasis, the use of anticoagulated blood has clear analytical advantages, including repeat sampling and storage. However by altering the physiological state of the blood reproducibility and accuracy of the test is affected. Recent studies have shown the potential of a novel functional biomarker of clot formation: Fractal Dimension (*d*_*f*_), that may give an improved diagnostic accuracy. In this study we assessed the potential of this new biomarker in scientifically measuring the effects of recalcification of citrated samples.

**Methods:**

35 healthy volunteers were included. Unadulterated and sodium citrate samples of blood were taken from each volunteer. Citrated samples were recalcified using (1M CaCl_2_). In the study we compared unadulterated whole blood d_f_ results to *citrated d*_*f*_ results and repeated the *citrated* d_f_ experiments 5 times for each sample over a 2 hour period to ascertain reproducibility.

**Results:**

The *d*_*f*_ of citrated blood was significantly lower than that of unadulterated blood (1.57±0.04 vs 1.69±0.04, p<0.001). The results of the citrate samples when tested 5 times over 2 hrs gave a coefficient of variation of 1.16%.

**Conclusions:**

For the first time we show that a functional biomarker of clot microstructure, *d*_*f*_, can precisely quantify and measure accurately the direct effect that the addition of the anticoagulant sodium citrate has on whole blood clot microstructure. The study also shows that the test is reproducible and has potential utility as a biomarker of acute disease in the emergency setting in citrated blood. This procedure now needs to be evaluated in a group of acute disease states.

## P326 Thromboelastography in Dengue fever in emergency department (TiDE)

### MS Roslanuddin^1^, H Rossman^2^, FJ Sabariah^2^

#### ^1^Hospital Raja Permaisuri Bainun,Ipoh, Emergency Department, Ipoh, Perak, Malaysia; ^2^Hospital Sungai Buloh, Emergency Department, Selangor, Malaysia

**Introduction:**

In this study, we analyzed the hematological abnormalities of dengue patients by thromboelastography (TEG) at initial and 1-hour of fluid resuscitation.

**Methods:**

This is a cross-sectional study evaluating TEG readings of dengue patients with different severities presenting to the emergency department. Laboratory confirmed dengue patient (positive NS1 antigen or IgG/IgM) was consecutively sampled. TEG readings were taken at presentation and after 1-hour of fluid resuscitation.

**Results:**

Twenty dengue patients with varying severity had a median Reaction time (R), α -angle, K time, Maximum Amplitude (MA) and Lysis 30% (Ly30) of 0.495 min, 68.74^ο^, 3.58 min, 44.64 mm and 0.54% respectively. Mean fibrinogen was normal before and after fluid infusion. There is a non-significant reduction in MA with prolongation of other TEG parameters between different dengue severities. There is a statistically significant reduction of α-angle and MA between pre and post 1-hour fluid resuscitation (p=0.019 and p=0.040).

**Conclusions:**

Normal fibrinogen with low MA, which signifies a weak clot strength, may indicate either a platelet reduction, platelet dysfunction or both. Reduction in MA and α-angle post fluid resuscitation is an alarming finding. This is in contrast with previous TEG studies although none of it used normal saline exclusively, studied initial fluid resuscitation in emergency department settings or studied a subject with dengue. A bigger study, especially in severe dengue is needed to validate our findings.

## P327 Agreement between the thromboelastography reaction time parameter using fresh and citrated whole blood during extracorporeal membrane oxygenation with TEG®5000 and TEG®6s

### M Panigada, S De Falco, N Bottino, P Properzi, G Grasselli, A Pesenti

#### Fondazione IRCCS Ca´ Granda Ospedale Maggiore Policlinico, Intensive Care Unit, Milano, Italy

**Introduction:**

The R (reaction time) parameter of kaolin-activated thromboelastography (TEG) may be used to assess the degree of heparinization of blood during ECMO. A TEG analysis is usually performed on two types of samples: fresh (F) or citrated-recalcified (C) whole blood. TEG®5000 can perform the analysis on C and F whole blood, the new TEG®6s (Haemonetics Corp., MA, USA) only on C whole blood. Aim of the study was to compare the response of R to heparin using the two types of samples and two TEG devices

**Methods:**

During a three months period at Fondazione IRCCS Ca’ Granda – Policlinico of Milan, TEG was performed (using TEG5000® and TEG 6s® with and without heparinase, an enzyme that degrades heparin) on 13 consecutive ECMO patients (as part of the GATRA study, NCT03208270) and in 8 consecutive non-ECMO patients in whom a TEG was requested for clinical purposes. Bland Altman analysis and Lin's concordance correlation coefficient were used to assess agreement

**Results:**

A total of 84 paired samples were taken (74 in-ECMO and 10 off-ECMO). ECMO patients received 19.2 (12.6- 25.8) IU/kg/h of heparin. Among non-ECMO patients, 5 of them did not receive any dose of heparin, two of them a very low prophylactic dose (1.6 and 2.9 IU/kg/h, respectively), and one of them 13.1 IU/kg/h of heparin. Using TEG®5000, R was -21.0 (-65.5; 23.4) min shorter on C compared to F blood in patients receiving heparin (this difference disappeared using heparinase) and only -1.58 (-8.70; 5.54) min shorter in patients not-receiving heparin. R was -26.6 (-77.3; 24.2) min shorter using TEG®6s (which performs the analysis only on C blood) than TEG®5000 on F blood (Figure 1).

**Conclusions:**

When evaluating the effect of heparin using TEG, clinicians should be aware that results obtained using citrated-recalcified or fresh whole blood are not interchangeable. Using citrated-recalcified blood to perform TEG might lead to underestimation of the effect of heparin

**Fig. 1 (abstract 327).**
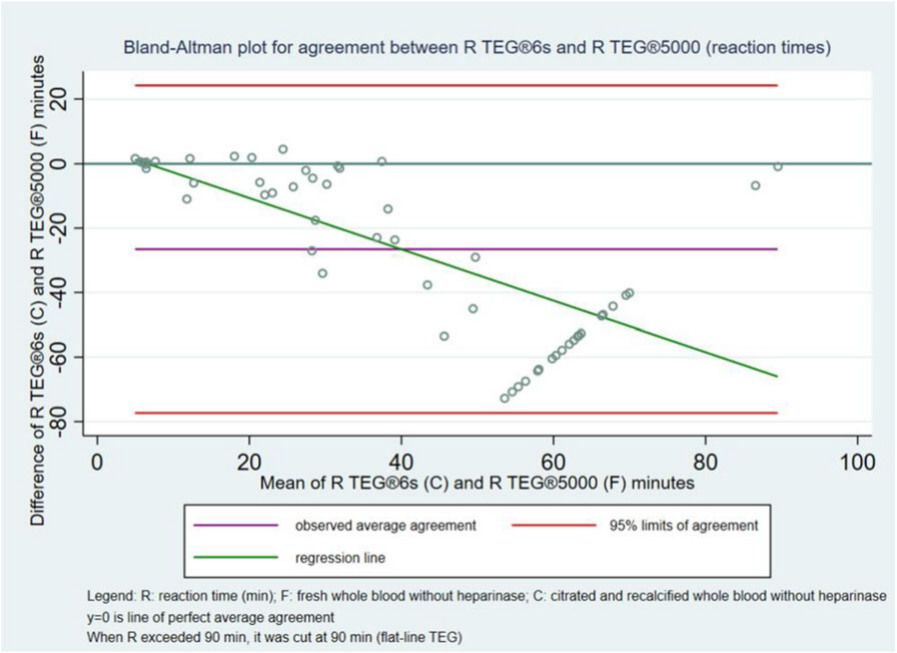
Agreement between TEG®6s and R TEG®5000 on citrated recalcified and fresh whole blood

## P328 Pharmacist-initiated protocol for antiXa-based enoxaparin dosing in trauma patients

### EN Morales^1^, CJ Richardson^2^, JT Jancik^1^

#### ^1^Hennepin County Medical Center, Pharmacy, Minneapolis, United States; ^2^Hennepin County Medical Center, Surgery, Minneapolis, United States

**Introduction:**

Trauma patients are at high risk for venous thromboembolism (VTE). The 2002 EAST guidelines recommend low molecular weight heparin (LMWH) for VTE prevention and antiXa monitoring after initiation of the medication or after adjusting doses in certain populations [1]. Studies have shown standard enoxaparin dosing of 30 mg every 12 hours may result in low antiXa levels [2]. This study aims to evaluate the efficacy of a pharmacist-lead protocol for adjusting enoxaparin dosing based on antiXa levels in trauma patients.

**Methods:**

This single center retrospective chart review included adult trauma patients admitted from 3/1/2018 to 9/20/2019. Per protocol, patients with body mass index (BMI) ≤40 kg/m^2^ were initiated on enoxaparin 30 mg twice daily, and patients with BMI > 40 kg/m^2^ were initiated on enoxaparin 40 mg twice daily. Peak antiXa levels were drawn 4 to 6 hours after at least the third dose of enoxaparin with a goal therapeutic range of 0.2-0.4 IU/mL. The primary objective was time in days to goal peak antiXa level. Secondary objectives include VTE occurrence, bleeding attributed to LMWH, and dosing regimens utilized. Subgroups were analyzed based on body mass index (BMI).

**Results:**

Of 511 patients identified, 375 patients met inclusion criteria. Median time to therapeutic antiXa level was 3 days (IQR 2-6). Of 337 patients with BMI ≤ 40 kg/m^2^, 319 patients (94.7%) were dosed initially per protocol and 153/319 patients (48.0%) met goal antiXa level at first check (Table 1). Of 38 patients with BMI > 40 kg/m^2^, 24 patients (63.1%) were dosed initially per protocol and 8/24 patients (33.3%) met goal antiXa level at first check.

**Conclusions:**

Our results indicate the protocol is safe due to lack of bleeding attributed to enoxaparin, but less than 50% of patients achieved goal antiXa level at first check. However, despite low rates of achieving goal antiXa level, VTE rates also remained low.

**References:**

1. Rogers FB et al. J Trauma 53:142-164, 2002.

2. Walker CK et al. Ann Pharmacother 51:323-331, 2017.

**Table 1 (abstract P328).** Results

	All Patients (N = 375)	BMI < 40 kg/m2 (n = 337)	BMI > 40 kg/m2 (n = 38)
Time to goal antiXa in days – median (IQR)	3 (2-6)	3 (2-5.8)	2.5 (1.8-8)
AntiXa within goal at first check	168/375 (44.8%)	153/337 (45.4%)	13/38 (34.2%)
Final dose (mg) to reach goal antiXa–mean +/- SD	34.5 +/- 8.0	33.2 +/- 5.9	46.5 +/- 13.5
Number of dose changes – mean +/- SD	0.35 +/- 0.6	0.33 +/- 0.55	0.64 +/- 0.93
Rates of VTE	8/375 (2.1%)	7/337 (2.1%)	1/38 (2.6%)
Rates of bleeding	0/375 (0.0%)	0/337 (0.0%)	0/38 (0.0%)

## P329 Anti factor Xa (AFXa) activity monitoring in a mixed ICU patient population – are we giving enough enoxaparin to our patients?

### TM Mann^1^, O Rahlin^2^, I Barnet^1^, M Izak^2^, AH Mayo^1^

#### ^1^Assuta Medical Center, Department of Intensive Care, Ashdod, Israel; ^2^Assuta Medical Center, Department of Hematology, Ashdod, Israel

**Introduction:**

Most patients in the ICU are given prophylactic anticoagulation with a fixed dose of 40 mg once daily of Enoxaparin (clexane) if CCT is normal and 20 mg if CCT is low. Studies on non ICU patients have shown that AFXa is below desired range for venous thromboembolism (VTE) prevention. In the ICU, many factors might influence AFXa levels including weight, creatinine clearance (CCT), shock and other medication. ATXa activity was not yet reported in a big mixed ICU population with variable morbidity. Our study hypothesis is that Enoxaparin is underdosed in most cases and routine AFXa activity should be monitored in all ICU patients.

**Methods:**

Preventive enoxaparin (40 mg qd) was given to all patients unless therapeutic dose was needed or contraindication existed. Levels of AFXa activity were taken 4 hours after the 3^rd^ dose. Therapeutic VTE preventive effect was defined as AFXa activity of 0.2-0.5. Patient data was collected from medical files.

**Results:**

The study is still ongoing, preliminary results were analyzed for 31 patients. 14 of 31 patients (45%) had AFXa activity below normal (subtherapeutic). Weight and CCT were negatively correlated with AFXa activity (Figure 1). Mean weight in the subtherapeutic AFXa was significantly higher than the therapeutic group (83.2 vs. 72.6 respectively, p=0.031). CCT in the subtherapeutic AFXa was significantly higher than the therapeutic group (95.1 vs. 60.4 respectively, p=0.003). The normal CCT group (>50) had significantly more patients with subtherapeutic AFXa (13 vs 8, p=0.017).

**Conclusions:**

In our ICU, 45% of the patients receive insufficient VTE prophylaxis. Overweight patients and patients with normal CCT should probably receive higher Enoxaprin dose. AFXa activity should be routinely monitored in ICU patients.

**Fig. 1 (abstract P329).**
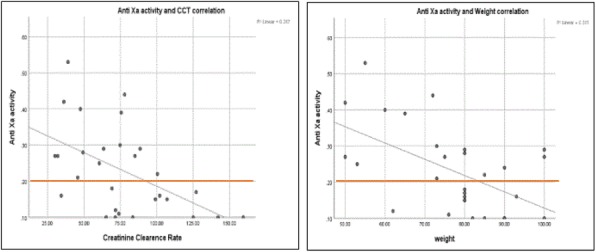
Correlation of anti factor Xa activity with patient CCT and weight. Anti FXa activity value below 0.2 (red line), was considered “non-effective prevention”

## P330 Gel point analysis quantifies the anticoagulant effect of rivaroxaban in first time DVT

### VJ Evans^1^, MJ Lawrence^1^, J Whitley^1^, C Johns^2^, SK Pillai^1^, PR Williams^3^, K Hawkins^4^, K Power^5^, K Morris^6^, PA Evans^1^

#### ^1^Welsh Centre for Emergency Medicine Research, Swansea, United Kingdom; ^2^Singleton Hospital, Acute GP Unit, Swansea, United Kingdom; ^3^Swansea University, College of Engineering, Swansea, United Kingdom; ^4^Swansea University, Medical School, Swansea, United Kingdom; ^5^Singleton Hospital, Pharmacy, Swansea, United Kingdom, ^6^Cardiff Metropolitan University, Cardiff School of Sport and Health Sciences, Cardiff, United Kingdom

**Introduction:**

In this study we use a new bedside biomarker to test its ability to measure anticoagulation effects on patients who present with acute first time deep vein thrombosis (DVT). DVT requires oral anticoagulants to prevent progression to potentially fatal pulmonary embolism and recurrence. Therapeutic efficacy monitoring of direct oral anticoagulants (DOAC) including rivaroxaban is problematic as no reliable test is currently available. Advances in hemorheological techniques have created a functional coagulation biomarker at the gel point (GP) which allows quantitative assessment of: time to the gel point (T_GP_), fractal dimension (*d*_*f*_) and elasticity (G’) [1, 2].

**Methods:**

The prospective observational cohort study measured T_GP_, *d*_*f*_, G’, standard coagulation and cellular markers in first time DVT patients at three sample points: pre-treatment and approximately 20 and 60 days following 15mg BD and 20mg OD rivaroxaban respectively. Strict inclusion and exclusion criteria applied.

**Results:**

40 DVT patients (mean age 64 years [SD±14.8]; 23 male, 17 female) and 177 non-DVT patients were well matched for age, gender and co-morbidities. Mean T_GP_ on admission was 267s (SD±63.3s) and 262.9s (SD±73.5s) for DVT and Non-DVT respectively. DOAC therapy significantly increased T_GP_ to 392.3s (SD±135.7s) after 20 days, and subsequently increased to 395.5s (SD±194.2s) at 60 days as shown in Table 1. *d*_*f*_, G’ and standard hemostatic markers all remain within the normal range.

**Conclusions:**

T_GP_ demonstrates its utility in determining the anticoagulant effect of rivaroxaban. The significant difference in T_GP_ between males and females needs further exploration. Localized stasis as a result of transient provoking factors appears not to generate a systemic strength enhancement in clot microstructure. Factors linked to increased clot strength in DVT patients include increased levels of hematocrit, fibrinogen, white cell count and male gender.

**References:**

1. Evans et al. Blood 116:3341–3346, 2010

2. Lawrence et al. BMJ 168:571–575, 2015

**Table 1 (abstract P330).** Time to the gel point (TGP) and prothrombin time (PT) results for DVT group at baseline and on treatment. Values reported as mean ± Standard Deviation. Significance values assessed using one-way ANOVA

	DVT baseline	Rivaroxaban 15mg BD dose	Rivaroxaban 20mg OD dose	Significance valve
TGP (s)	267.0 ± 63.3	392.3 ± 135.7	395.5 ± 194.2	<0.001
Prothrombin Time (s)	10.8 ± 0.58	13.6 ± 1.73	12.6 ± 1.47	<0.001

## P331 Impact of protocoled nursing care on quality performance measures in patients with severe trauma

### N Matsumoto^1^, M Sato^1^, M Toyodome^1^, Y Shimoikeda^1^, T Sawai^1^, M Murai^1^, J Yoshimura^2^, K Yamakawa^2^, S Fujimi^2^

#### ^1^Osaka General Medical Center, Department Of Nursing, Osaka, Japan; ^2^Osaka General Medical Center, Division of Trauma and Surgical Critical Care, Osaka, Japan

**Introduction:**

Trauma remains the leading cause of death all over the world. To better exploit the trauma care system, precise diagnosis of the injury site and prompt control of bleeding are essential. Here, we created a nursing protocol for initial medical care for trauma. The aim of this study was to evaluate the impact of protocoled nursing care for trauma on measures of quality performance.

**Methods:**

This was a retrospective historical control study, consisted of consecutive severe trauma patients (Injury Severity Score >16). People were divided into two groups: protocoled group (from April 2017 to March 2019) and control group (from April 2014 to March 2017). We set the primary endpoint as mortality for bleeding. The secondary endpoints included time allotted from arrival to start of CT scan and surgery, administration rate of several drugs (sedations, painkillers, preoperative antibiotics, and tranexamic acid). For the statistical analysis, continuous variables were expressed as median (interquartile range) and were compared by Wilcoxon rank sum tests given a non-normal distribution of the data.

**Results:**

We included 152 patients in the study: 84 in the control group before the introduction of the protocol, 68 in the protocoled group. As a primary endpoint, the mortality for bleeding was similar between two groups (5% in the control group and 5% in the protocoled group). As a secondary endpoint, the time to CT initiation [Group A 11 (7-16) min vs Group B 7 (5-10) min; p <0.001], and emergency procedure [Group A 41 (33-56) min vs group B (29-43); p <0.001] were shortened by the protocol introduction. Furthermore, the administration rates of sedations, painkillers, preoperative antibiotics, and tranexamic acid were increased in the protocoled group compared with the control group.

**Conclusions:**

Although the mortality as a patient-oriented outcome was not affected, improved quality of medical care by nursing protocol introduction may be suggested in this analysis.

## P332 Long-term physical function and quality of life in patients with unstable pelvic ring fractures

### N Saito^1^, T Seo^2^, H Iida^2^, H Matsumoto^2^

#### ^1^Nippon Medical School Chiba Hokusoh Hospital, Shock and Trauma Center, Inzai, Chiba, Pref, Japan; ^2^Nippon Medical School Chiba Hokusoh Hospital, Inzai, Chiba, Pref, Japan

**Introduction:**

Unstable pelvic ring fracture (UPRF: AO-OTA type B, C) were life-threatening injuries, but with recent advances in acute resuscitation have focused attention on physical function and quality of life after discharge. We aimed to clarify the long-term physical functional and quality of life in patients with UPRF.

**Methods:**

This single-institutional prospective study included 72 patients with UPRF who were admitted to the trauma surgical intensive care unit (TSICU) and survived until discharge to home between 2012 and 2017. We evaluated the activities of daily living after the discharge using physical and mental component scores of SF-36® and defined physical dysfunction (PD) as physical function (PF-N) score of 40 or less. We divided the patients in the PD (n=34) and control (without PD, n=38) groups and compared the groups.

**Results:**

The patients had experienced blunt injuries, including falls (38%) and pedestrian injuries (33%). The mean age was 59.9 years (men: 65.3%); the median injury severity score was 22 (interquartile range: 13–29); and the mean length of TSICU stay was 3.3 days. The average period from the injury until the survey was 34.1 months. There was no difference between the PD group and the control group in the patient characteristics, fracture type, pelvic fixation, and complications. At the time of the survey, the PD group had significantly more painful complaints than the control group (PD: 67.6%, C: 23.6%, P <0.01), and had more physical and mental problems. The SF-36®subscale score showed a significant positive correlation between physical function and body pain, mental health respectively. The percentage of those who were able to return to work was not different in both groups (PD:26.5%, C:47.4%). In the multivariate analysis of PD, only age (odds ratio: 1.039, 95% CI: 1.00-1.07, P = 0.03) was relevant.

**Conclusions:**

Long-term PD was observed in 47% of patients with UPRF. The elderly were particularly prominent, and there was an association between pain and mental health.

## P333 Electrochemically induced change in the morphology of red blood cells – a new method for the diagnosis of critical conditions

### A Evseev^1^, I Goroncharovskaya^1^, A Kuzovlev^2^, K Popugaev^1^, S Petrikov^1^

#### ^1^N.V. Sklifosofsky research institute of emergency medicine, Moscow, Russia; ^2^Federal research and clinical center of intensive care medicine and rehabilitology, Moscow, Russia

**Introduction:**

The development of new methods for diagnosing critical states is one of the most important tasks. Patients in critical condition are characterized by a violation of many body functions, affecting the morphofunctional characteristics of blood cells. In case of red blood cells (RBC) this can lead to inhibition of oxygen transport function and development of hypoxia. Currently used methods for analyzing the state of RBC either do not have sufficient accuracy or require lengthy analysis and expensive equipment. The use of a simpler and more informative electrochemical approach to assessing the state of RBC is very promising.

**Methods:**

Electrochemical measurements in RBC suspensions (~ 5 ∙ 109 cells / l) were carried out in a special electrochemical cell [1] in the potentiodynamic mode in the potential range from -0.5 to +1.2 V using the IPC Pro MF potentiostat (Kronas, Russia); optical measurements were performed using an Eclipse TS100 inverted microscope (Nikon, Japan), a CFI S Plan Fluor ELWD 60x / 0.70 lens (Nikon, Japan); RBC morphology was recorded in real time using a DS-Fi1 digital camera (Nikon, Japan).

**Results:**

When examining RBC of patients with severe multiple trauma a decrease in the ability of RBC to change their shape during electrochemical exposure was observed, indicating a decrease in deformability, which can lead to a disruption in the oxygen supply to tissues. At the same time, with the stabilization of the patient's condition a restoration of the ability of RBC to change morphology was detected which in turn could have a positive effect on the rheological characteristics of the blood (Fig. 1).

**Conclusions:**

The results of the analysis of red blood cells using electrochemical changes in their morphology can be used as an additional method for the diagnosis of critical conditions.

**References:**

1. Yu TA et al. Doklady Physical Chemistry 477:201-204, 2017

**Fig. 1 (abstract P333).**
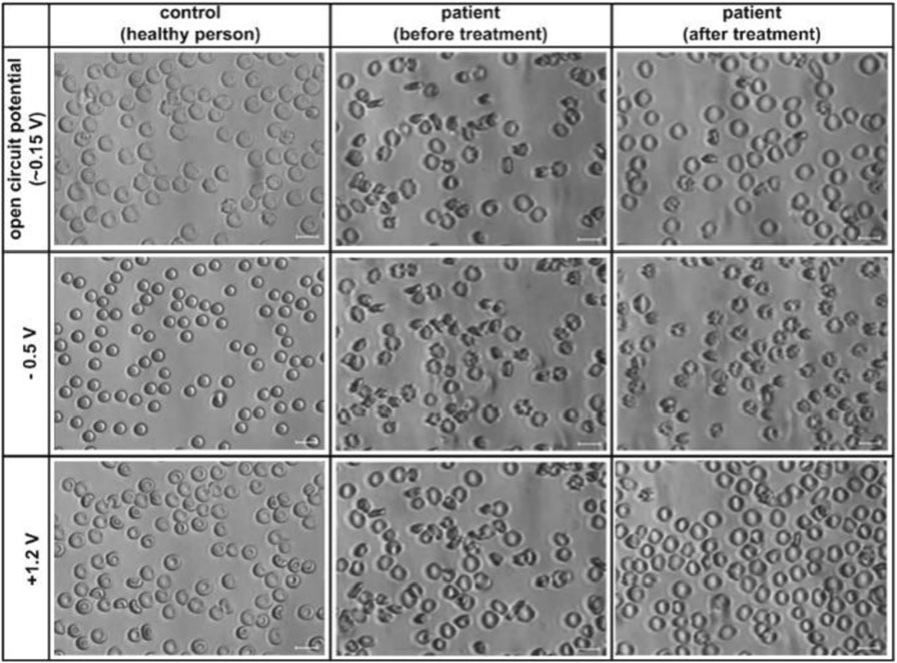
The effect of therapy on the electrochemically induced change in the morphology of red blood cells in patients with combined trauma

## P334 Deep neural network models for detection of severe trauma injuries using whole-body CT scout

### N Okada^1^, K Yamakawa^1^, S Inoue^2^, Y Umemura^1^, J Yoshimura^1^, Y Matsuzawa^3^, S Fujimi^1^

#### ^1^Osaka General Medical Center, Division of Trauma and Surgical Critical Care, Osaka, Japan; ^2^AI study group in the emergency room, Image analysis group, Tokyo, Japan; ^3^Brown University, Department of Mathematics, Rhode Island, United States

**Introduction:**

Severe trauma should be treated immediately. Whole-body CT (WBCT) is widely accepted to improve the accuracy of detecting injuries. However, it remains the problem of time-consuming. Therefore, we focused on the scout image taken in advance of WBCT. Detecting major traumatic injuries from a single scout image would reduce the time to start treatment. A previous study suggested that even specialists could not easily find chest and pelvic injuries using WBCT scout image alone. In this study, we aimed to develop and validate deep neural network (DNN) models detecting pneumo/hemothorax and pelvic fracture from WBCT scouts.

**Methods:**

We retrospectively collected 2088 anonymous WBCT scouts together with their clinical reports at the Osaka General Medical Center between January 1, 2013, and December 31, 2017. We excluded incomplete, younger than 7 years old, postoperative, and poorly depicted images. The part of this dataset from January 1, 2017, until December 31, 2017, was used for validation and the rest for training DNN models. Pneumo/hemothorax detection model and pelvic fracture detection model were trained respectively. Accuracy, and Areas under the receiver operating characteristic curves (AUCs) were used to assess the models.

**Results:**

The training dataset for pneumo/hemothorax contained 984 images (mean age 48 years; 30% female patients), and for pelvic fracture consisted of 783 images (48 years; 28%). The validation dataset for the former contained 258 images (54 years; 30%), and for the latter consisted of 186 images (55 years; 24%). The models achieved 59% accuracy and an AUC of 0.57 for detecting pneumo/hemothorax, 72% and 0.62 for pelvic fracture.

**Conclusions:**

Our results show that DNN models can potentially identify pneumo/hemothorax and pelvic fracture from WBCT scouts. Increasing the number of samples, DNN model could accurately detect severe trauma injuries using WBCT scout image.

## P335 Launch of clinical information system in intensive care unit – facilitating staff in transition from paper records to electronic database

### YC Wong, YL Lau, WT Yeung, PO Lei, KC Law, MC Cheung

#### Ruttonjee & Tang Shiu Kin Hospitals, Cardiac & Intensive Care Unit, Hong Kong SAR, Hong Kong

**Introduction:**

Clinical Information System (CIS) is a computer system used in collecting, processing, and presenting data for patient care. It can reduce staff workload and errors; help in monitoring quality of care; track staff’s compliance to care bundles; and provide data for research purpose. However, the transition from paper record format to electronic record involves changes in all kind of workflow in ICU. Therefore, an effective, efficient and evaluative rollout plan was required to minimize the risk that might arise from the new practice.

**Methods:**

1. Small groups training were provided. A working station with different case scenarios were set up for practices.
2. Individual tutorials were conducted to clarify questions. Emphasis on patient care was always top priority.
3. Contingency plans were available in case of server breakdown and power failure. Downtime drills were conducted to prepare the staff in emergency situations.
4. Step-by-step transition from paper record to electronic format was gradually carried out. A plan was discussed among CIS team with clear dates and goals.
5. New items in CIS were first reviewed and amended in team meeting until consensus was made; then were promulgated to all staffs during handover before implementation.
6. Staff compliance and outcomes were then monitored; further review and amendment would be possible if necessary.

**Results:**

CIS roll-out plan was smooth. All staffs were able to integrate CIS into the daily routine. The contingency plans were well acknowledged. New items were followed as planned. Ongoing enhancement in CIS was put forward on nursing orders, handover summary, and integration with Inpatient Medication Order Entry (IPMOE) system.

**Conclusions:**

With emerging benefits CIS brings along, our staff has more time to devote to direct patient care. Human input in data interpretation and clinical judgment on top of CIS play an irreplaceable role in patient care.

## P336 Reducing unnecessary laboratory testing in an intensive care unit of a tertiary hospital - what changed after one year?

### L Simvoulidis^1^, RC Costa^2^, CA Ávila^2^, TS Sória^2^, MM Menezes^2^, JR Rangel^2^, JP Pinho^2^, RP Pereira^2^, AP Porto^2^

#### ^1^Hospital Unimed Rio, UTI Geral, Rio De Janeiro, Brazil; ^2^Hospital Unimed Rio, UTI Geral, Rio De Janeiro, Brazil

**Introduction:**

The daily request for laboratory tests in intensive care units is a common practice. Although common, this strategy is not supported, since more than 50% of the exams requested with this rationale may be within the normal range [1]. Misconduct based on misleading results, anemia, delirium and unnecessary increase in costs may happen [2]. We have developed a strategy to reduce laboratory tests without clinical rationale.

**Methods:**

Observational retrospective study, from July 2018 to June 2019. The number and type of laboratory orders requested, the epidemiological profile of hospitalized patients, the use of advanced supports, the average length of ICU stay and the impact in outcomes such as mortality and hospital discharge at a private tertiary general hospital in the city of Rio de Janeiro / RJ - Brazil were analyzed.

**Results:**

A strategy was implemented to reduce the request for exams considered unnecessary. Approximately 1,300 patients underwent ICU during this period. The epidemiological profile and severity of patients admitted to the unit were similar to those observed historically. There was a significant reduction (> 50%) in the request for laboratory tests and there was no negative impact on outcomes such as mortality, mean length of stay and no greater use of invasive resources. Over the period evaluated, the estimated savings from reducing the need for unnecessary exams were approximately $ 150,000 per year.

**Conclusions:**

The rational use of resources in the ICU should be increasingly prioritized and the request for routine laboratory tests reviewed. A strategy that avoids such waste, when properly implemented, enables proper care, reducing costs and ensuring quality without compromising safety.

**References:**

1. Peixoto AA Jr et al. Crit Care 17(Suppl 3):P12, 2013

2. Choosing Wisely [Internet]. Philadelphia (PA), ABIM Foundation. 2018; [cited 2018 Jan 10]. Available from: www.choosingwisely.org.

## P337 Evaluating the medication reconciliation errors in 2 ICUs after implementing a hospital-wide integrated electronic health record system

### A Rosillette, R Shulman, Y Jani

#### University College Hospital, Centre for Medicines Optimisation Research and Education, London, United Kingdom

**Introduction:**

Medication errors in Intensive Care Unit (ICU) are frequent [1] and can arise from a number of causes including transition of care. Our aim was to investigate the impact of an integrated Electronic Health Record System (EHRS) on medication reconciliation (MR) errors occurring at 2 critical steps: during the transition from an ICU to the hospital ward and from the ward to hospital discharge. The objective was to examine the influence of ICU admission on long-term medication.

**Methods:**

We performed a monocentric study in 2 ICUs of a university-affiliated hospital using drug chart and medical notes review to identify MR errors before, during and after ICU admission. Data were collected retrospectively from EHRS for 50 consecutive patients discharged from the ICU between 1 June-31 July 2019, and who were newly initiated on specific drugs of interest.

**Results:**

129 drugs of interest were initiated in ICU. Many of these were continued after hospital discharge as shown in Table 1. There was appropriate discontinuation of all the antipsychotics newly initiated in ICU. Other than anticoagulants, there was no reason documented for continuation of the initiated drugs. The planned durations were documented more often after hospital discharge than ICU discharge for the following drug classes (% of patients with a plan after ICU discharge to the ward; % after home discharge): antibiotics (47.6%; 52.6%), and steroids (45.4%; 57.1%), but less so for analgesics (36.3%; 14.3%), insomnia (0.0%; 20.0%), and gastroprotective drugs (0.0%; 20.0%).

**Conclusions:**

Our study has shown that medications initiated in the ICU can be inadvertently continued at ICU and hospital discharge due to failure in documenting indication or duration. Systems are required to deprescribe ICU only drugs at discharge or communicate a plan for ongoing treatment.

**References:**

1. Moyen et al. Crit Care 12: 208-215, 2008

**Table 1 (abstract P337).** Drug classes initiated in ICU and continued after ICU discharge

Drug class	Drug class initiated in ICU	Drug class continued after transfer to the ward	Drug class continued on home discharge
Antibiotics	22	16 (72.7%)	9 (40.9%)
Steroids	10	8 (80.0%)	4 (40.0%)
Analgesics	19	17 (89.5%)	11 (57.9%)
Insomnia	7	6 (85.7%)	1 (14.3%)
Anticoagulants	2	2 (100.0%)	2 (100.0%)
Antipsychotics	4	2 (50.0%)	0 (0.0%)
Gastroprotective drugs	23	22 (95.6%)	12 (52.1%)

## P338 Sepsis bundle delivery and intensive care outcomes

### D Cottam, M Zuleika, F Wade-Smith, W Gray

#### Royal Surrey County Hospital, Intensive Care Unit, Guildford, United Kingdom

**Introduction:**

The Surviving Sepsis Campaign advocates the use of care bundles to guide the management of sepsis and septic shock [1]. Our study aim was to assess compliance with a locally introduced sepsis pathway and to review intensive care unit admission outcomes.

**Methods:**

We carried out a prospective audit of patients admitted to the ICU at Royal Surrey County Hospital with a diagnosis of sepsis between 19/3/19 and 19/11/19, assessing compliance with local sepsis bundle delivery, outcome of ICU admission and degree of associated organ dysfunction.

**Results:**

119 patients were identified, 71 male (59.7%), with a mean age of 65.7 (18-96). Mean 1^st^ 24 hour SOFA score on ICU was 6.65 (2-15). 81% of patients required vasopressors, with 67% requiring noradrenaline >0.1mcg/kg/min, and 19% requiring an additional vasopressor/inotrope. 36% required NIV, 32% invasive ventilation and 15% RRT. ICU mortality was 15%, in-hospital mortality 24%, mean ICU stay 8 days (1-49), and mean length of hospital stay 28 days (1-163). In the presence of septic shock mortality was 47% with post-resuscitation lactate >4, versus 21% in patients with no vasopressor requirement or lactate <2 (p<0.05). The sepsis bundle was delivered in one hour to 61 patients (51%). Where the bundle wasn’t completed, antibiotics were delayed in 26% of cases and blood cultures weren't taken in 66%. Where the bundle was fully delivered, unit mortality was 12% vs. 21% where it was not (p<0.05), but there was no significant difference in hospital mortality (26% vs. 30%, p>0.5) or rates of vasopressor requirement, NIV, IPPV or RRT.

**Conclusions:**

There is room for improvement in timely delivery of the sepsis bundle in our hospital and various measures are being instituted. Though there was no significant difference in hospital mortality, ICU mortality was significantly lower in patients when the bundle was fully delivered.

**References:**

1. Rhodes et al. Intensive Care Med 43:304, 2017

## P339 Compliance with 3-6h sepsis resuscitation bundle of surviving sepsis campaign in an emergency department

### MH Hagui^1^, RA Allani^2^, OD Djebbi^1^, Ml Ben Lassoued^1^, Kl Lamine^1^

#### ^1^Main Military Hospital of Tunis, Emergency departement, Montfleury, Tunisia; ^2^Main Military Hospital of Tunis, General Directorate of Military Health, Montfleury, Tunisia

**Introduction:**

Surviving sepsis campaign recommends 3h and 6 h sepsis resuscitation bundle for sepsis. The study was done to assess the feasibility of the guideline and the compliance to sepsis-3 recommendations at an emergency department.

**Methods:**

Prospective interventional study was conducted during one year. Were involved in the study all sepsis cases with a qSOFA ≥ 2. Were assessed a composite of six components (Measurement of serum lactate, Obtaining blood culture before antibiotic Administration and Provision of broad-spectrum antibiotic before the end of H3 and Provision of fluid bolus in hypotension, Attainment of target central venous pressure assessed by cardiac ultrasonography, Target lactate to normal level before the end of H6). Time base line was the first medical contact at triage zone. Secondary outcomes of study were the mortality rate and length of stay at Intensive care unit (ICU).

**Results:**

Were involved in the study, 128 patients (mean age 54±17 years, sex ration 2,3). Pulmonary infections were the main cause of sepsis (37%) and urinary tracts infections (22%). At H3 components were achieved in 79% of cases [lactates (100%), blood culture (82%) and provision of antibiotics (79%)]. At H6 components were executed in 64% of cases (fluid provision achievement in 89%, ultrasonography assessment in 64% and normal lactate target achieved in 71%) (Figure 1). The reliability-adjusted rate for completion of the 3 hours and 6 hours Bundle was at 68%. Patients compliant to composite bundle got the mortality benefit (odds ratios = 0.31, 95% [confidence interval, 0.11-0.72]). The study, however, did not show any benefits of mean intensive care unit (ICU) length of stay.

**Conclusions:**

Faisability of 3-6h Bundle ratio was at 68%. It has shown a significant improvement in adaptation and mortality benefit without reducing mean hospital/ICU length of stay. More adapted procedures are needed to improve results targeting full compliance of patients to the 3-6h Bundle sepsis management.

**Fig. 1 (abstract P339).**
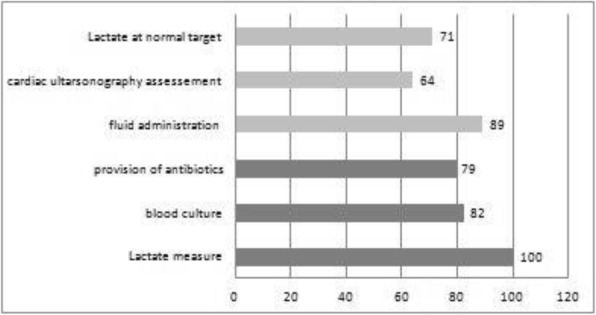
Sepsis 3-6h bundle components (% of goals achievment)

## P340 Patterns and outcome of critical care admissions with sepsis in a resource limited setting

### M Edirisooriya Maddumage^1^, Y Gunasekara^2^, D Priyankara^1^

#### ^1^National Hospital of Sri Lanka, Medical Intensive Care Unit, Colombo 10, Sri Lanka; ^2^Sri Jayawardenepura General Hospital, Department of Critical Care, Nugegoda, Sri Lanka

**Introduction:**

Paucity of epidemiological data is a major barrier in expansion of critical care services, especially in resource limited settings. We evaluated the patterns and the outcome of critically ill patients with sepsis admitted to a level 3 Medical Intensive Care Unit in Sri Lanka.

**Methods:**

A retrospective cohort study was performed to describe the characteristics and outcome of patients with sepsis, admitted to a medical intensive care unit. Sepsis is defined according to sepsis 3 definition.

**Results:**

We examined 360 critically ill patients admitted over a period of 6 months. Sepsis was the commonest presentation, accounted for 63.6 % of all admissions. Mean age was 49.2 ± 16.1 years. Septic shock was present in 41.4% on admission. Pneumonia (35.4%) was the commonest cause, while Leptospirosis (17.9%) and meningoencephalitis (12.2%) accounted for second and third commonest causes of sepsis respectively. The SOFA score on admission (8.8 ± 5.0 vs 7.5 ± 4.9, P< 0.025), occurrence of AKI (62% vs 49.6%, p<0.02) and the length of ICU stay (8.8 days vs 6.2 days, p <0.001), were significantly higher in sepsis than in patients without sepsis. ICU mortality in sepsis (n=92) did not show a significant difference to nortality (n= 45) in those without sepsis (40% vs 35%, p= 0.5). Patients with leptospirosis had a mean SOFA score of 13.3, however the mortality (37.5% vs 40%, p = 0.75) was similar to others with sepsis. In contrast, mortality related to sepsis was significantly high ( 60%, p< 0.02) in the packground of immunosuppression (n= 35).

**Conclusions:**

Respiratory failure secondary to pneumonia was the commonest cause of critical care admission with sepsis. Sepsis related ICU mortality was high in the background of immunosuppression.

## P341 Improving the safe use of nasogastric tubes in the ICU: from computer to classroom

### A Chapman^1^, M Perera^2^, M Perera^2^, P Beddoes^3^, DJ Melia^4^

#### ^1^Lister Hospital, Department of Anaesthesia, Hertfordshire, United Kingdom; ^2^Eastbourne Hospital, East Sussex, United Kingdom; ^3^Whipps Cross University Hospital, Department of Anaesthesia & Critical Care, London, United Kingdom; ^4^Whipps Cross University Hospital, Dept of Anaesthesia and Critical Care, London, United Kingdom

**Introduction:**

Training in placement, and the subsequent safe confirmation of position, of a nasogastric (NG) tube, relies on clinicians completing an e-Learning module at our trust. Feeding through an incorrectly placed NG tube is a 'Never Event,' associated with significant morbidity and mortality [1]. Analysis of these incidents reveal that the misinterpretation of chest radiographs, by medical staff, who had not received competency-based training, is the most frequent cause [2]_._ e-Learning has revolutionized the delivery of medical education [3], however, there are barriers to its use [4]. We hypothesized that, by taking e-Learning content, and delivering it face-to-face, we would improve training rates, and thus patient safety.

**Methods:**

A questionnaire was completed by 50 critical care doctors, concerning their knowledge of the existence of the e-learning module, whether they had completed formal training in NG tube placement, and how confident they were, on confirming correct positioning, using a 5 point Likert scale. All clinicians underwent training in the interpretation of NG placement, using chest radiographs. After the session they were asked to re-appraise how confident they felt. Results were compared using paired t tests.

**Results:**

Confidence improved in all, rising from a pre-test average score of 3.74 (SD=0.92), to post-session 4.76 (SD=0.48), p=<0.0001. Prior to the intervention, 63% of the doctors were aware of the trust guidelines, but only 17% had completed the training. After the session, 100% were aware of the guidelines, and 100% had completed the training (Figure 1).

**Conclusions:**

e-Learning is a useful tool, but has its limitations. By using course content, delivered with more traditional learning methods, we improved the number of appropriately trained clinicians, and thus the safe use of NG tubes in our unit.

**References:**

1. Rassias AJ et al. Crit Care 2:25-28, 1998 2. NHS Improvement Patient Safety Alert. Ref: NHS/PSA/RE/2016/006

3. https://www.e-lfh.org.uk/about/

4. Assareh A et al. Procedia CS 3:791-795, 2011

**Fig. 1 (abstract P341).**
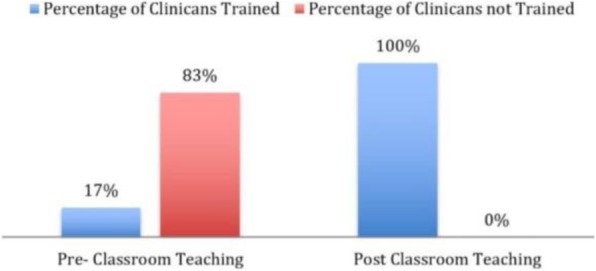
Percentage of clinicians trained before and after intervention

## P342 A systematic review of anticoagulation strategies for patients with atrial fibrillation in critical care

### A Nelson, B Johnston, A Waite, I Welters, G Lemma

#### University of Liverpool, Liverpool, United Kingdom

**Introduction:**

There is a paucity of data assessing the impact on clinical outcomes of anticoagulation strategies for atrial fibrillation (AF) in the critical care population. This review aims to assess the existing literature to evaluate the effectiveness of anticoagulation strategies used in critical care for atrial fibrillation.

**Methods:**

A systematic literature search was conducted by searching MEDLINE, EMBASE, CENTRAL and PUBMED databases. Studies of adults based on critical care with a diagnosis of AF were eligible.

**Results:**

1119 studies were identified through the databases and 38 were reviewed by full text. 4 studies were selected for data extraction. A total of 44087 patients were identified with AF, of which 17.8-49.4 % of patients received anticoagulation. The incidence of thromboembolic events ranged from 0-1.4 % in the anticoagulated population and 0-1.3 % in the non-anticoagulated population. Major bleeding events were reported in 3 studies and occurred in 8.6 % of each of the anticoagulated populations. Major bleeding events occurred in 0-7.1 % in the non-anticoagulated groups. 30-day mortality in patients treated with vitamin K antagonists (VKA) was 22.9 % (21.3-24.6) and 30.6 % (28.9-32.4) for untreated patients. Annual mortality was 35.4 % (33.5-37.3) with VKA treatment and 46.3 % (44.4-48.3) without.

**Conclusions:**

Only 4 studies contained analysable data. Anticoagulated patients had a lower mortality at 30 days and 365 days post admission to critical care, however there was an increased incidence of major bleeding events compared to the non-anticoagulated population. Thromboembolic events were comparable in both cohorts. Data from current literature is scarce and inferences regarding the effectiveness of anticoagulation in patients in critical care with AF requires further investigation and research.

## P343 A review of handovers on the intensive care unit

### EJ Jones^1^, DH Hepburn^2^

#### ^1^Cardiff University, Medical School, Cardiff, United Kingdom; ^2^Royal Gwent Hospital, Intensive Care Medicine, Newport, United Kingdom

**Introduction:**

Every new admission to the ICU prompts a handover from the referring department to the ICU staff. This step in the patient pathway provides an opportunity for information to be lost and for patient care to be compromised. Mortality rates in Intensive Care have fallen over the last twenty years, however, 20% of patients admitted to an ICU will die during their admission [1]. Communication errors contribute to approximately two-thirds of notable clinical incidents; over half of these are related to a handover [2]. NICE have concluded that structured handovers can result in reduced mortality, reduced length of hospital stay and improvements in senior Clinical Staff and Nurse satisfaction [3].

**Methods:**

A checklist was created to review the information shared and to score the handover. This checklist was created with Doctors and Nurses and is relevant for handovers between all staff members. Information was gathered prospectively by directly observing 17 handovers on the ICU.

**Results:**

There is a notable discrepancy in the quality of handovers of new patients (Figure 1). This is true of handovers between Doctors, Nurses and a combination of the two. It is also true of all staff grades. Whilst a Doctor may have reviewed the patient prior to their arrival, 41% (n=7) of patients weren’t handed over to a doctor. The most commonly missed pieces of information were details of the patient’s weight (96%, n=16), their height (100%, n=17), whether the patient has previously been admitted to an ICU (78%, n=15) and whether the patient has any allergies (71%, n=12).

**Conclusions:**

The handover of new patients to the ICU is often unstructured and important information is missed. This can be said for all staff members and grades, and for handovers from all hospital departments.

**References:**

1. ICS. Guidelines for the Provision of Intensive Care Services, Version 2. ICS; 2018.

2. Starmer AJS et al. Pediatrics 129 :201-4, 2012

3. Chapter 32, Structured Patient Handovers, (2018).

**Fig. 1 (abstract P343).**
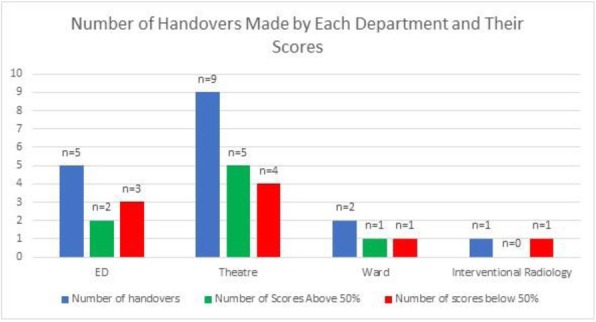
Each handover was scored according to the information accurately given to ICU staff

## P344 Caregiver burden after prolonged cardiothoracic critical care

### P Henderson^1^, I Quasim^2^, A Asher^3^, L Campbell^3^, M Shaw^4^, T Quasim^5^, J McPeake^5^

#### ^1^University of Glasgow, Department of Anaesthesia and Intensive Care medicine, Glasgow, United Kingdom; ^2^Golden Jubilee National Hospital, Cardiothoracic Anaesthesia and Critical Care, Glasgow, United Kingdom; ^3^Golden Jubilee National Hospital, Cardiothoracic Critical Care, Glasgow, United Kingdom; ^4^NHS Greater Glasgow & Clyde, Department of Clinical Physics and Bioengineering, Glasgow, United Kingdom; ^5^University of Glasgow, Department of Anaesthesia and Intensive Care Medicine, Glasgow, United Kingdom

**Introduction:**

Post intensive care syndrome–family (PICS-F) describes new or worsening psychological distress in family and caregivers after critical illness but remains poorly studied within specialist groups [1]. We aim to define the degree of PICS-F within our tertiary referral cardiothoracic centre and map change over the course of 12 months.

**Methods:**

Caregivers attended a 5-week multi-professional clinic alongside patients. Peer support was facilitated through a café area and a caregiver group psychology session was offered with individual appointments if required. Caregiver surveys were completed including: caregiver strain index; hospital anxiety and depression scale (HADS); and insomnia severity index. Patients also completed HADS questionnaires. Repeat surveys were completed at 3 and 12 months.

**Results:**

Over 5 cohorts, 23 caregivers attended, of which 17 were spouses (74%), 3 children (13%), and 3 others (13%), with 13 caregivers completing surveys at 12 months. Patients' median APACHE 2 score was 17 (IQR 14-18.5) and median ICU length of stay was 11 days (IQR 8-23.5). Most admissions were from scheduled operations (56%). Severe caregiver strain was present in 4/23 (17%) with changes to personal plans (35%) the most common sub category. HADS demonstrated 13 caregivers (57%) with anxiety and 8 (35%) with depression. Caregiver anxiety exceded that of patients', only reaching similar levels at 12 months, while depression remained static (Figure 1). Median number of nights with ‘bothered’ sleep was 5 (IQR 1-6.75) and 77% of caregivers expressed problems with sleep.

**Conclusions:**

Significant psychological morbidity in caregivers from our tertiary cardiothoracic centre is in keeping with the general ICU population [2]. Caregiver strain was reduced suggesting higher levels of resilience. Future work should address mental wellbeing, particularly anxiety, to minimise the effects of PICS-F.

**References:**

1. van Beusekom et al. Crit Care 20:16, 2015

2. McPeake et al. J Crit Care 35:180-4, 2016

**Fig. 1 (abstract P344).**
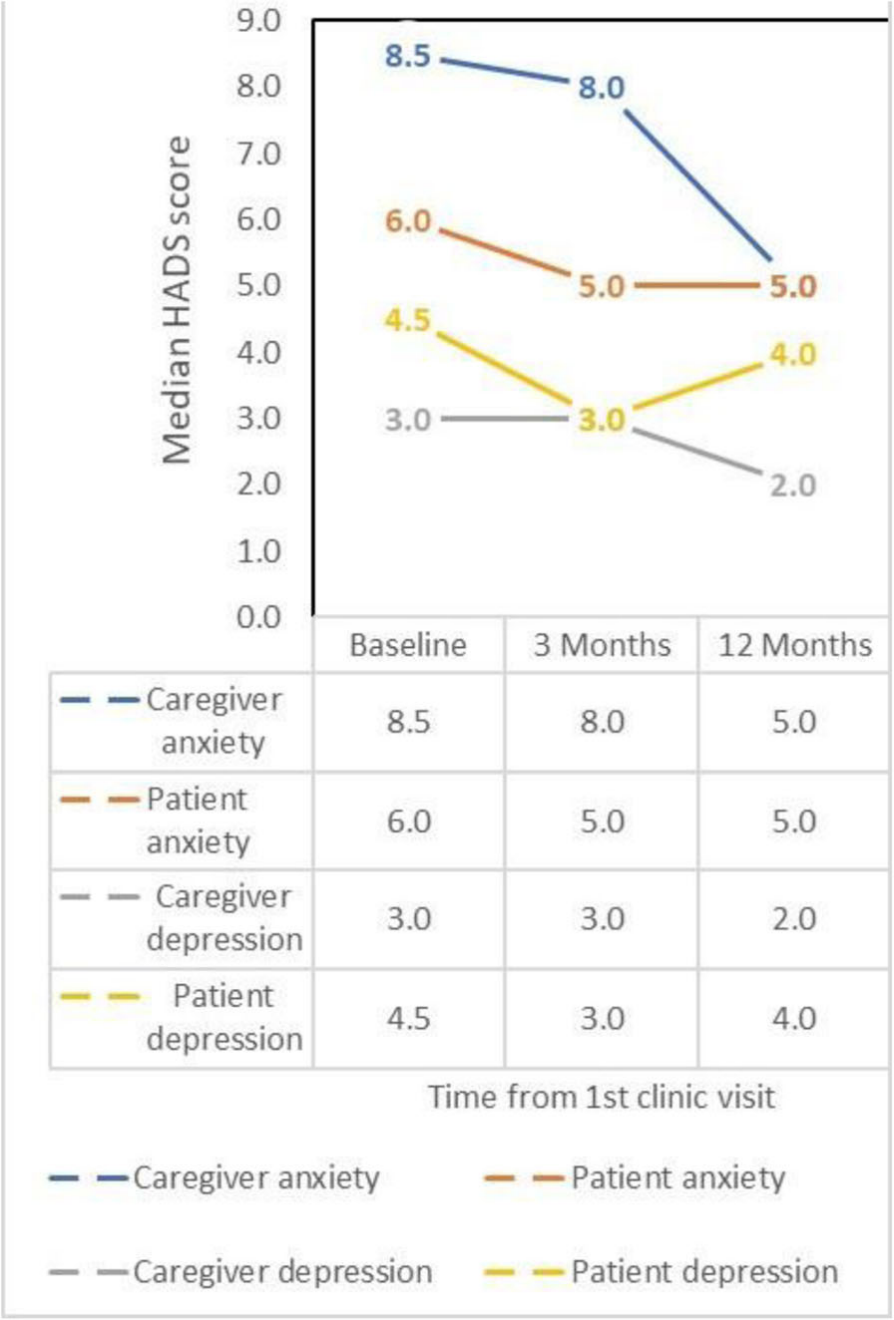
Hospital anxiety and depression scale (HADS) scores for patients and caregivers at baseline, 3 months, and 12 months

## P345 Evaluation of burnout in a Brazilian intensive care center

### E Perecmanis

#### Hospital Caxias Dor, Intensive Care, Duque de Caxias, Brazil

**Introduction:**

Burnout syndrome is an illness that has increasingly affected health professionals. It is characterized by great emotional stress, physical and mental exhaustion and depersonalization of the individual. More serious cases can lead to job loss or even suicide. The described work identifies the burnout level of the multidisciplinary team through a specific questionnaireBurnout syndrome is an illness that has increasingly affected health professionals. It is characterized by great emotional stress, physical and mental exhaustion and depersonalization of the individual. More serious cases can lead to job loss or even suicide. The described work identifies the burnout level of the multidisciplinary team through a specific questionnaire

**Methods:**

Application of a questionnaire suitable for the multidisciplinary group in November 2019. The same was answered by 85 professionals among physicians and nursing team. There was no identification of employees.

**Results:**

After analysis of the results it is observed that 50% of the group presents initial burnout, 30% with the syndrome installed and about 5% with characteristics of greater severity. Main factors found were: mental and physical exhaustion during the work day, the level of responsibility existing in the activity and the perception of disproportionate remuneration by work performed.

**Conclusions:**

All interviewees presented some degree of burnout or high risk to develop it. The most severe cases should be traced through occupational medicine and anti-stress measures with reorganization of work performance should be discussed in order to reduce the prevalence of this syndrome.

## P346 Depression and burnout in the ICU: an urban perspective of prevalence and trend

### I Greaves^1^, R Maharaj^2^

#### ^1^King College Hospital, Critical Care, London, United Kingdom; ^2^King College Hospital, London, United Kingdom

**Introduction:**

Burnout affecting the psychological and physical state of healthcare workers is recognized in the last 10 years. Burnout has been shown to affect the quality of care. Whilst some risk factors have been identified, there are gaps within the literature related to mental health and burnout. The aim of this study is to measure levels of burnout across 3 ICU units in the metropolitan setting.

**Methods:**

To determine the level of burnout we used 2 surveys, the Maslach Burnout Inventory human services survey (MBI-hss) and the Centre for Epidemiologic Studies Depression Scale (CES-D). With the MBI-hss we analysed 3 different variables of burnout; exhaustion, cynicism and emotional exhaustion. Basic demographic data and information regarding workout schedules were collected. We studied prevalence and contributing risk factors using and analysing the outcomes of the 2 self-scoring questionnaires. Analysis was performed using descriptive statistical analysis.

**Results:**

There were 90 respondents, 36% scored the threshold for depressive symptoms on the CES-D depression scale. Interestingly, 40% (CI 25.4-57.7%) of those meeting the score for depressive symptoms identified as having frequent restless sleep compared with 11% (4.6-21.8 %) from those not meeting. Gender did not affect depressive symptoms 35% of females and 37% of males met the threshold. With the MBI-hss for exhaustion the mean was 17.16 (SD 4.6) which is a high level of exhaustion, the second variable cynicism the mean score was 14.08 (SD 4.2), which was considered high. The final variable was emotional exhaustion the mean was 25.16 (SD 9.90), this is considered moderate levels of emotional exhaustion.

**Conclusions:**

There was high prevalence of burnout in ICU  in all different categories as well as depressive symptoms. Age and gender had no affect on burnout. Interestingly, we identified that sleep and shift variables were linked to increased burnout.

## P347

**Withdrawn**

## P348 Analysis of clinical pharmacists’ contributions in an intensive care unit after implementing an hospital-wide fully integrated electronic health record system

### A Rosillette, R Shulman, Y Jani

#### University College Hospital, Centre for Medicines Optimisation Research and Education, London, United Kingdom

**Introduction:**

Patient and medication safety are important components of critical care. Safety strategies include the contribution of a clinical pharmacist and the implementation of an Electronic Health Record System (EHRS). The study aimed to analyse clinical pharmacists’ contributions in Intensive Care Unit (ICU) after implementing a new hospital-wide EHRS.

**Methods:**

Following the implementation of a fully integrated EHRS on 31 March 2019 at our university-affiliated hospital we conducted a prospective study in 2 ICUs by analysing pharmacists’ contributions during 4 data collection periods of 5 days at 10, 15, 19 and 21 weeks post implementation. A pharmacists’ contribution was defined as contacting the physician to make a recommendation in a change of therapy/monitoring [1]. The 3 types of contribution were: a medication error - rectification of an error in the medication process; an optimization - proactive contribution that sought to enhance patient care, and a consult - reactive intervention in response to a request. A panel of experts composed of a senior pharmacist, a consultant, a nurse, and a pharmacy student assessed the impact of each contribution, scoring low impact, moderate impact or high impact.

**Results:**

There were 160 pharmacist contributions recorded in the 4 periods. Of these, 66 (41.2%) were medication errors, 93 (58.1%) were optimizations, and 1 (0.6%) was a consult (Table 1). 37% of the contributions were assessed as having medium impact, 36% as high impact and 27% as low impact. In general, the consultant assessed fewer contributions as having high impact compared to other members of the panel, with 7 contributions assessed as high impact by the consultant versus 97 by the senior pharmacist.

**Conclusions:**

Implementing an EHRS in combination with contributions of clinical pharmacists can prevent medication related issues. Interestingly the types of incident did not change over time.

**References:**

1. Shulman et al. J Crit Care 30 :808–813, 2015

**Table 1 (abstract P348).** Number and type of contribution for each period

Type of contribution	Medication error	Optimization	Consult	Total
Period 1	21	26	0	47
Period 2	12	12	1	25
Period 3	17	21	0	38
Period 4	16	34	0	50
Total	66	93	1	160

## P349 Evaluation of noise level in intensive care unit

### L Karabýyýk, BS Kalýn, M Çimen, Ö Nadastepe

#### Gazi University School of Medicine, Anesthesiology and Intensive Care, Ankara, Turkey

**Introduction:**

Most ICU’s are noisy and may adversely affect patients outcomes and staff performance [1]. WHO reports that the noise level in hospitals should not exceed 35 dB at daylight and 30 dB at night. The aim of this study is to evaluate the noise levels in intensive care unit, to apply awareness training to intensive care staff in terms of noise and to compare the noise levels before and after education.

**Methods:**

Noise measurement areas are separated into 17 points including 12 patient bedsides, nurse desk, staff desk, wareroom, corridor and entrance of intensive care unite. Measurements were performed 14 times per day. After 10 day, awareness training were given to staff in terms of harmful effects of noise. After the training, noise measurements were repeated during 10 days. After total 20 days the measurements were terminated. Noise was measured with incubator analyzer (FLUKE model: Bio-tek serial no:6050274).

**Results:**

The mean noise values before and after the training were not statistically different from the mean average noise values (p>0.05). When the time of measurement were compared, the noise levels were higher between 10-16 hours to other measurements before and after the training statistically (p=0.001). Seventeen different noise measurement areas were compared in terms of noise level, there was no statistically significant difference (p>0.05). The differences were examined at the same hours between before and after training. Contrary to expectations, noise levels were found to be higher after training statistically (p<0.05). All of noise measurements were higher than the threshold values that WHO recommended.

**Conclusions:**

Increased noise levels in critical care units may lead to harmful health effects for both patients and staff. Our results suggest that much noise in the ICU is largely attributable to environmental factors and behavior modifications due to education have not a meaningful effect.

**References:**

1. Darbyshire JL et al. BMJ 353:i1956, 2016

## P350 Clinical profile and outcomes after admission in a tertiary level critical care facility in a low income country

### E Shimber

#### Hawassa University Comprehensive Specialized Hospital, Emergency and Critical Care Medicine, Hawassa, Ethiopia

**Introduction:**

Critical care medicine has focused on continuous, multidisciplinary care for patients with organ insufficiency in the face of life-threatening illness. Despite significant resource limitation low income countries carry a huge burden of critical illness. Available data is insufficient to clearly show the burden and outcomes of intensive care units in these developing countries [1]. The objective of our study is to evaluate the morbidity and outcomes of patients admitted to the Intensive Care Unit of a tertiary university hospital in Hawassa, Ethiopia.

**Methods:**

This was a prospective observational study. Data was registered and analysed starting from patient admission to discharge during a 12 month period beginning September 2018. Data regarding demographics, sources of admission, diagnosis, length-of-stay and outcomes were analysed.

**Results:**

The total number of patients admitted to the ICU was 218, with 71 patients dying over a one year period. The highest admission was from emergency medical unit, 36% and the lowest source was from pediatrics department, 8%. Out of these, 69.8% were males. The mean age was 27 years (2-62). The most frequent aetiologies of morbidity in the admitted patients were traumatic brain injury (24.6%), acute respiratory distress syndrome (22.9%) and seizure disorder (8%). Average median length of stay was 3.0 days (interquartile range: 1.0 - 27.0). The overall mortality rate was 32.5%. The top four causes of death in the ICU were respiratory illness at 24% followed by sepsis with multiorgan failure at 20%, trauma (16%) and central nervous system infection (12%).

**Conclusions:**

Infection morbidity and mortality remains very high and needs institution of aggressive preventive strategies. The increase in frequency of trauma patients need to receive due attention. Sepsis causes a high number of deaths, though overtaken by respiratory illnesses. Improving the overall system of ICU may achieve better outcomes in resource limited countries.

**References:**

**1**. Marshall JC et al. J Crit Care 37:270-76, 2017

## P351 Early mortality in critically ill patients admitted to ICU

### A Estella, M Gracia Romero, M Recuerda Nuñez

#### University Hospital of Jerez, Intensive & Critical Care, Cadiz, Spain

**Introduction:**

ICU mortality has been widely studied in the literature in relation to outcome index that primarily value organic failure [1]. However, early mortality, in the first 48 hours of admission has been little documented in the literature. The aim of this study is to analyze factors related to early mortality in ICU.

**Methods:**

Retrospective study at a second-level hospital. Time of study was 12 months. Patients who died in ICU were included, patients were classified according timing of dead, including those who died within the first 48 hours of ICU admission. The variables analyzed were age, sex, comorbidity, Charlson index, APACHE II, need for supportive treatments, more frequent admission diagnosis, origin and support treatment limitation decisions. The statistical study was carried out using the SPSS statistical program.

**Results:**

138 patients were included during the study period, 72 (52.2%) died within the first 48 hours of admission. No differences in the needs of support treatments were observed, more than 90% of patients received mechanical ventilation and vasoactive therapies. Table 1 shows characteristics of patients.

**Conclusions:**

Half of ICU deaths occur within the first 48 hours of admission. Severity at ICU admisison was the main factor related with early mortality.

Severe stroke and coronary disease were the most frequent causes of early deaths in ICU.

**References:**

1. Daviaud F et al. Ann Intensive Care 5:16, 2015

**Table 1 (abstract P351).** Clinical characteristics of patients

	Dead first 48 hours. n: 72	Dead after 48 hours . n: 66
Age	65.16±16.7	66.01±10.8
Charlson Index	4.1±2.28	4.8±2.19
Apache II	27.31±7.84	19.98±7.66
Gender (% male)	59.7%	74.2%
Origin: Medical ward Surgical ward	26.4% 12.5% 48.6% 8.3%	34.8% 15.2% 33.3% 16.7%
Most frequent admission diagnosis	Septic shock 16.7% Stroke 16.7% Cardiac arrest15%	Septic shock 25.8% Stroke 19.7% Surgery 15.2%
Limitation support therapies	19.4%	37.9%

## P352 Are you ready? - an audit of the 2018 guidelines in maternal critical care in a major obstetric centre

### M Smith^1^, Y Metodiev^2^, H Lynes^3^, D O´Neil^3^

#### ^1^Department of Anaesthetics and Intensive Care, Anaesthetics/ICU, Leicester, United Kingdom; ^2^Department of Anaesthetics and Intensive Care, Department of Anaesthetics, Leicester, United Kingdom; ^3^Department of Anaesthetics and Intensive Care, Leicester, United Kingdom

**Introduction:**

In August 2018 The Royal College of Anaesthetists published guidelines on care of the critically ill woman in childbirth and enhanced maternal care [1]. Approximately 11000 babies are born across the area covered by Leicester University Hospitals that includes two large maternity units and is part of the UK ECMO network. This audit sets out to assess current practice and form a basis for future planning, which will likely be representative to most major obstetric centres.

**Methods:**

A retrospective audit of all patients admitted to ‘intensive care units’ in Leicester over a 12 month period following publication of the guidelines. The focus was on patients admitted to general adult intensive care and excludes all patients cared for in ‘enhanced obstetric care’ units. 9 simple standards were proposed relating to accessibility, resuscitation, follow up and multi-disciplinary learning.

**Results:**

In total 49 women were identified with a broad range of diagnosis. The intensive care services are split across 3 hospitals and we found this led to a number of problems. The presence of trained staff to resuscitate a newborn were easily accessible, no steps to provide necessary equipment pre-emptively were present in any centre. None of our critical care units had a plan for perimortem section. On-going reviews by the obstetric and midwifery teams were very variable. Contact with the infant and breastfeeding support was also poor.

**Conclusions:**

Despite the large number of deliveries significant work needs to be done in order to come in line with the new national guidelines for critically ill woman in childbirth. Clearly defined pathways around escalation of care, resuscitation of both the mother and baby, integrating care of the mother and the infant in the first few days of life, and multidisciplinary learning events are being produced *de novo* in response to these guidelines, some of which will be illustrated in the associated poster.

**References:**

1. Care of the critically ill woman in childbirth; enhanced maternal care. *RCoA*, 2018

## P353 Communication, interprofessional co-operation of ICU staff, and professional satisfaction

### T Bekiarii^1^, D Papadopoulos^2^, A Georgakis^3^, P Papamichalis^2^, P Katsiafylloudis^2^, E Neou^2^, M Chatzimichail^2^, A Palioura^2^, M Malliarou^4^, A Komnos^2^

#### ^1^Prefecture Of Thessaly, Department Of Public Health, Larisa, Greece; ^2^General Hospital Of Larisa, Intensive Care Unit, Larisa, Greece; ^3^Prefecture Of Thesssaly, Statistician/Mathematician, Larisa, Greece; ^4^University Of Thessaly, Nursing School/ Health Sciences, Larisa, Greece

**Introduction:**

Studies suggest that, when health professionals possess communication and conflict management skills, interprofessional co-operation is ensured. Τhis supports the proper functioning of the hospital and, by extension, the satisfaction of the nurses from their working environment

**Methods:**

Population consisted of 45 individuals. The tools used at this study were: a)The Jefferson Scale of Attitudes towards Physician-Nurse Collaboration [1], b)Professional Quality of Life Scale [2] and c) Interprofessional collaboration Scale [3]. Data were analyzed with IBM SPSS 25.0

**Results:**

It was found that cooperative attitudes with an average score of 49 to 75 are considered to be of average significance. Interprofessional cooperation at an average score of 2,568 states that the level of cooperation is high and the quality of working life averages 125 to 150, suggesting that it is very good. As far as professional satisfaction is concerned, nurses are happy, content and satisfied with their work, despite workload and burnout

**Conclusions:**

Interprofessional cooperation at the ICU of the General Hospital of Larissa is high, but satisfaction from wages, resources, working environment and conditions is low. In addition, the results showed that improvements in hospital communication between staff, has a positive impact on the quality of professional life

**References:**

1. Hojat M et al. Int J Nurs Studies 40: 427-435, 2003

2. Stamm BH (2005) The ProQOL manual: The professional quality of life scale:Compassion satisfaction, burnout & compassion fatigue/secondary trauma scales. [e-book] Lutherville, MD, Sidran Press

3. Kenaszchuk C et al. BMC Health Services Res 10:83, 2010

## P354 Longitudinal trends in intensive care admission according to age in Wales: 2008-2017

### R Pugh^1^, M Al Sallakh^2^, A Akbari^2^, R Bailey^2^, R Griffiths^2^, C Subbe^3^, C Thorpe^4^, C Battle^2^, T Szakmany^5^, R Lyons^2^

#### ^1^Department of Anaesthetics, Glan Clwyd Hospital, Bodelwyddan, United Kingdom; ^2^Swansea University, Swansea, United Kingdom; ^3^Bangor University, Bangor, United Kingdom, ^4^Ysbyty Gwynedd, Bangor, United Kingdom; ^5^Cardiff University, Cardiff, United Kingdom

**Introduction:**

As national populations age, demands upon critical care services are expected to increase. However, while increased admission of older patients (>80 years) to ICU has been observed in Australia and some European countries, decline observed elsewhere may indicate rationing according to chronological age. This effect was explored for Wales, a nation with historically low numbers of ICU beds (5.9 per 100,000), by analysing ICU admissions over a 10-year period.

**Methods:**

Welsh adult ICU episodes 2008-17 were examined via the Secure Anonymised Information Linkage (SAIL) Databank, Swansea University. Modified Charlson’s Comorbidity Index (MCCI) was calculated using ICD-10 codes. Electronic Frailty Index (eFI) was calculated using an algorithm based upon primary care events. Patients categorized according to age as follows: 18-64 (youngest), 64-79 (older) and 80 (oldest) years and over.

**Results:**

85, 629 ICU episodes were identified. Number and proportion of older and oldest patients did not change significantly over the period. However, ICU admissions per 10,000 population fell from 93.9 to 81.4 and from 90.0 to 78.4 in the oldest and older cohorts, and increased from 21.7 to 22.2 in the youngest cohort. Across all age patients, there was a significant increase in admissions with moderate comorbidity (p=0.025) and a decrease in those with high comorbidity (p=0.022). There were no significant trends in organ support, whether overall or within age cohorts. A significant decrease in ICU mortality (from 23.5% to 20.5%, p=0.028) and hospital mortality (from 36.6% to 33.4%, p=0.017) were observed in the oldest cohort, though not 1-year mortality; this trend was not observed in other age cohorts (Table 1).

**Conclusions:**

Contrasting with previous reports, decreased admissions per unit population in older and oldest age groups, and those with high comorbidity, suggest resource constraints may have influenced admission discussion and decision-making over the 10-year study period in Wales. Further investigation is warranted.

**Table 1 (abstract P354).** Background characteristics and outcomes

Age (years)	18-64	65-79	80+
Episodes	39,551 (46.2%)	32,928 (38.5%)	13,150 (15.4%)
Female	47.1%	42.9%	49.5%
Moderate-severe comorbidity	76.1%	93.4%	94.5%
Frailty (moderate-severe eFI)	8.7%	23.7%	36.5%
Invasive ventilation	44.9%	38.1%	31.2%
Hospital mortality	14.5%	25.0%	33.5%
1-year mortaility	25.7%	45.9%	62.7%

## P355 ICU discharge into weekends and public holidays: an observational study of mortality

### N Mawhood, T Campbell, S Hollis-Smith, K Rooney

#### Bristol Royal Infirmary, General Intensive Care Unit, Bristol, United Kingdom

**Introduction:**

Up to a third of in-hospital deaths in ICU patients occurs following ward stepdown [1]. Discharge time seems to be associated with in-hospital prognosis, but meta-analyses have not shown a difference in weekday compared to weekend discharge [2,3]. However, papers that examined discharge ‘into’ out-of-hours days, particularly on Fridays, have found differences [3]. Our aim was to assess whether discharge from ICU ‘into’ out-of-hours (OOH - weekends and public holidays) is associated with in-hospital mortality or re-admission to ICU, and whether these patients were seen on the wards OOH by medical staff.

**Methods:**

All adults discharged from the general ICU to a ward at the Bristol Royal Infirmary in December 2016-18 were included. In-hospital mortality rates were assessed for each day, with ‘into weekdays’ defined as Sunday to Thursday and ‘into OOH’ Friday, Saturday and the day before a public holiday. A subset of patients with data on readmission rate to ICU was also examined. All available notes from patients discharged into OOH in 2018 were reviewed.

**Results:**

The study included 1732 patients with a subset of 443 with readmission data. 117 sets of notes were reviewed from patients discharged into OOH (Figure 1). The in-hospital mortality was significantly higher in patients discharged into OOH (5.1% vs 7.6%, P=0.012). Within the subset, OOH was associated with in-hospital mortality or readmission to ICU (6.8% vs 11.9%, P=0.044), though readmission rate alone was not (1.7% vs 2%, P=0.35). Of patients discharged into OOH, once on a ward 64% were reviewed by a specialty doctor but 20.5% were not seen.

**Conclusions:**

This is the first study to examine ICU discharge 'into' OOH days including public holidays. We found increased hospital mortality in OOH, similar to other studies [3]. Up to a fifth of high-risk ICU stepdown patients were not reviewed by a doctor on OOH days.

**References:**

1. Beck DH et al. Crit Care Med 25:9-15, 1997

2. Yang S et al. Crit Care 20:390, 2016

3. Vollam S et al. Intensive Care Med 44:1115-1129, 2018

**Fig. 1 (abstract P355).**
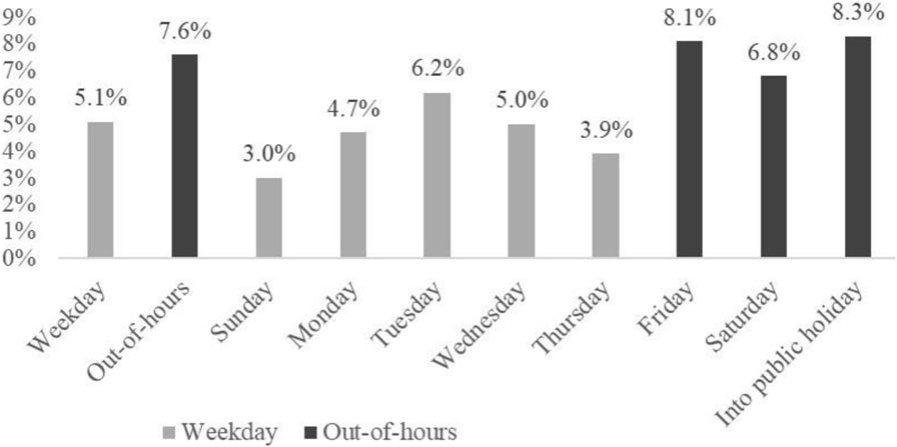
Percentage mortality according to day of discharge from ICU

## P356 Follow-up of psycho-emotional effects of organ procurement: difficult to study family members opposed to organ donation

### G Piemonte^1^, L Cecci^1^, C Di Pasquale^2^, L Quarta^2^, L Rasero^1^, M Bombardi^3^, C Guetti^4^, ML Migliaccio^3^, A Peris^5^

#### ^1^University of Florence, Department of Health Science, Florence, Italy; ^2^University of Pisa, Department of Culture and Forms of Knowledge, Pisa, Italy; ^3^Careggi Teaching Hospital, Regional Centre for Organ and Tissue Allocation, Florence, Italy; ^4^Careggi Teaching Hospital, Intensive Care Unit and Regional ECMO Referral Center, Florence, Italy; ^5^Tuscany Region, Tuscany Transplant Authority, Florence, Italy

**Introduction:**

Exploring the experiences of potential donors’ family members (FM) in a follow up clinic is crucial to analyze the effects of organ procurement (OP) on the bereavement process, to gain insight on the reasons of family refusals (FR), and to improve family care during OP.

**Methods:**

A mixed-method study involving FM at 3 and 12 months after patients’ death was developed and approved by Local Ethics Committee. FM of potential donors after brain (DBD) and cardiac death (DCD) treated in Careggi Teaching Hospital, Florence (Italy) were eligible if adult and consenting. Invitation letters were sent to the entitled 2 months after death and those who actively responded were involved in an encounter with a multidisciplinary group including a clinical psychologist, two nurses and two cultural anthropologists with expertise in OP. Psychological profile and satisfaction with care was assessed with validated tools, while the experiences of organ donation (OD) request and traumatic memories were measured with close and open ended questions.

**Results:**

90 envelopes were sent to the entitled of 79 DBD and 11 DCD. Responders were 29, 31.0% were FM of DCD. Reasons not to participate were logistical problems, linguistic barriers, dissatisfaction with care, lack of interest, perceived excessive burden. Of all FM, 18 (62.1%) were female, mean age 54.9 (DS 15.7). Encounters included from 1 to 4 FM; 11 were spouse (37.9%), 10 (34.5%) sons, 4 (13.8%) parents, 4 (13.8%) other relatives. 28 FM were Italian, 1 from USA; 28 consented to OD, only one refused. Three FM completed 12 months evaluation.

**Conclusions:**

The study is ongoing. The enrollment of not consenting FM was a relevant obstacle to the collection of data that could help to better understand FR. In order to reach an adequate sample size to draw consistent conclusions, we extended the study to all the ICUs of the Tuscany Region with the aim of recruiting an appropriate number of FM who refused OD.

## P357 Exploring psycho-emotional effects of organ procurement on potential donors’ relatives: preliminary results

### L Cecci^1^, G Piemonte^1^, L Rasero^1^, A Giustini^2^, M Bombardi^3^, C Di Pasquale^4^, L Quarta^4^, ML Migliaccio^3^, A Peris^5^

#### ^1^University of Florence, Department of Health Science, Florence, Italy; ^2^Careggi Teaching Hospital, Organ and Tissue Procurement Service, Florence, Italy; ^3^Careggi Teaching Hospital, Regional Centre for Organ and Tissue Allocation, Florence, Italy; ^4^University of Pisa, Department of Culture and Forms of Knowledge, Pisa, Italy; ^5^Tuscany Region, Tuscany Transplant Authority, Florence, Italy

**Introduction:**

Organ procurement (OP) is a complex process in which clinical, organizational and psycho-social issues are at play. Understanding the experiences and related memories of potential donors’ family members (FM) in a follow up clinic is crucial to identify the most relevant factors affecting OP and the associated outcomes. Our aims were to describe the effects of all phases of OP on the psycho-social wellbeing of FM, and then to gain insight into the possible causes of family refusals to organ donation (OD).

**Methods:**

Mixed-method study involving FM at 3 and 12 months after patients’ death. Psychological profile was evaluated with validated tools: PWBS (psychological well-being scale), STAI-Y (state trait anxiety inventory – form Y), BDI-II (Beck Depression Inventory - II), IES-R (Impact of Event Scale – Revised), ICG (Inventory of Complicated Grief). Traumatic memories were assessed with a 4-point scale. Satisfaction with care was measured with FS-ICU (family-satisfaction ICU). Content analysis was based on audio-taped semi-structured interviews.

**Results:**

29 FM were enrolled; 18 (62.1%) were female, mean age 54.9 (DS 15.7); 11 were spouse (37.9%), 10 (34.5%) sons, 4 (13.8%) parents, 4 (13.8%) other relatives. All FM but one were Italian, 96.5% consented to OD. DBD and DCD processes were analyzed. Quality of Life’s more affected dimensions were personal growth and autonomy; mean values of BDI-II (10.2), IES-R (25.2) and ICG (21.7) were below the clinical cut-off. FM showed slight symptoms of anxiety. Traumatic memories were more frequent and intense in relation with the very early stages of the process (out-of-hospital rescuing/emergency department) and with the communication of bad prognosis and death (Figure 1). Satisfaction with care was fair good.

**Conclusions:**

Preliminary data suggests that the early stages of OP and the communication of bad news deserve attention. The recruitment of not consenting FM was a relevant obstacle.

**Fig. 1 (abstract P357).**
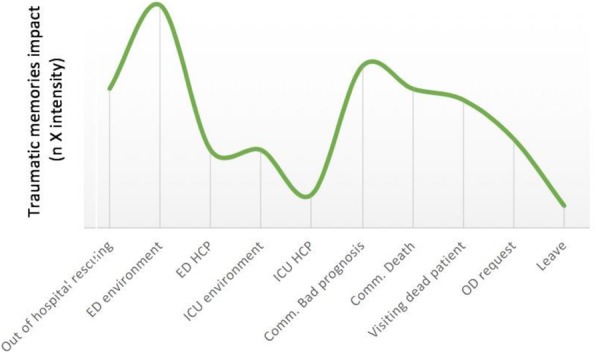
Traumatic memories impact on potential donors´ FM

## P358 ICU readmission in adult Egyptian patients undergoing living donor liver transplant: incidence,causes and outcomes: A single centre retrospective observational study

### HA Elgendy^1^, GM Elewa^1^, AA Korraa^1^, HA Ahmed^1^, MA El-metini^2^, MM Ahmed^2^, IM Montasser^3^, HM Dabbous^3^, MM Salah Eldin^3^, MF Abdelgafar^2^

#### ^1^Faculty of Medicine Ain Shams University, Anesthesia and Intensive Care, Cairo, Egypt; ^2^Faculty of Medicine Ain Shams University, General Surgery, Cairo, Egypt; ^3^Faculty of Medicine Ain Shams University, Tropical Medicine, Cairo, Egypt

**Introduction:**

Liver transplantation being a major abdominal surgery,may have postoperative complications which may be life threatening and require ICU readmission. We coducted this retrospective observational single centre study to identify incidence, causes and outcomes of ICU readmission after living donor liver transplant ( LDLT).

**Methods:**

This retrospective observational study was conducted in Ain Shams University Specialized Hospital on 335 adult patients ( > 18 years old) who underwent their initial LDLT from 2008 to 2018 after approval of Institutional Review Board and written informed consent from patients or 1st kin. Patients demographic data, preoperative variables, intraoperative variables, postoperative stay and complications, causes,incidence and outcomes of ICU readmission were recorded. Readmission is defined as ICU readmission within ≤ 3 months after initial ICU discharge.The primary endpoints were the incidence and causes of ICU readmission and secondary endpoint was one year survival.

**Results:**

A detailed review of 335 patients was made, 26 patients ( 7,76%) were readmitted, 4 patients ( 1.19 % of total patients), ( 15.3% of readmitted patients) were readmitted twice due to mesothelioma, sepsis, CVS and graft failure.Out of 26 readmitted patients, 11 patients ( 42.3 %) were discharged and 15 ( 57.7%) died and 25 % of twice readmitted patients discharged and 75 % died. The main causes of death were renal failure, hematemesis, sepsis, CVS, pulmonary complications, vascular complications and graft failure.

**Conclusions:**

Causes of ICU readmission post LDLT were variables, may be related to preoperative comorbidities, surgical complications, immunosuppression, sepsis and other causes. Readmission carries a greater risk of bad outcome. The more the times of ICU readmission the worse the prognosis.

## P359 Excellent results in kidney transplantation from donors with multiple organ failure - an unused resource in times of organ shortage

### LJ Lehner^1^, M Dürr^1^, K Budde^1^, J Kruse^1^, D Zickler^1^, F Friedersdorff^2^, R Öllinger^3^, F Halleck^1^, D Khadzhynov^1^

#### ^1^Charité Universitätsmedizin Berlin, Dept. of Nephrology and Medical Intensive Care, Berlin, Germany; ^2^Charité Universitätsmedizin Berlin, Dept. of Urology, Berlin, Germany; ^3^Charité Universitätsmedizin Berlin, Dept. of Surgery, Berlin, Germany

**Introduction:**

Organ replacement procedures such as ECMO (extracorporeal membrane oxygenation), LVAD (left ventricular assist device) and dialysis are routinely used to treat multi-organ failure (MOV). Globally transplantation programs struggle with increasing organ shortage. Patients (pts) with MOV are a potential source for procurement. However, outcome data after kidney transplantation (KTX) from such donors are sparse.

**Methods:**

We retrospectively studied the cadaveric KTX at the Charité Berlin in 2018 and identified donors with ongoing organ replacement procedures. Donor and recipient risk factors were assessed. Overall patient and graft outcomes were analyzed at 12 months post-transplant.

**Results:**

A total of 220 kidneys were transplanted. We identified 11 KTX from 7 donors with MOV (6 following cardio-pulmonary resuscitation, 5 with acute renal failure - 4 on dialysis) (Figure 1). In 3 donors, a veno-arterial ECMO was implanted during ECLS-resuscitation. One donor needed a veno-venous ECMO due to ARDS, and 1 donor had a LVAD implanted due to cardiac failure. The donor age was 41 ± 10.5 years (yrs). In addition, 6 donors had at least one cardiac risk factor. The kidney donor risk index averaged 0.94 (SD ± 0.14) and S-creatinine prior to KTX was 2.41 (SD ± 1.27). The recipients were 49.2 (SD ± 8.2) yrs of age and cumulated an average waiting time of 7.7 ± 2.9 yrs. Delayed graft function (DGF - dialysis within the first 7 days after KTX) occurred in 91% of patients (pts) (vs. 43.7% DGF in the overall 2018 cohort). All pts gained renal function at a median of 7 (IQR 5.25-9) days (100% vs. 96.3% 2018 cohort). Mean S-creatinine 12 months post-transplant was 1.30 ± 0.45 mg/dL resembling an estimated GFR of 58.86 ± 17.9 mL/min/1.73m2. One pt died unexpectedly 44 days after KTX with a functioning graft.

**Conclusions:**

The results of organs from donors with MOV in the first year after KTX were excellent. Especially the young donor age together with a targeted benefit-risk assessment enables the usage of such organs.

**Fig. 1 (abstract 359).**
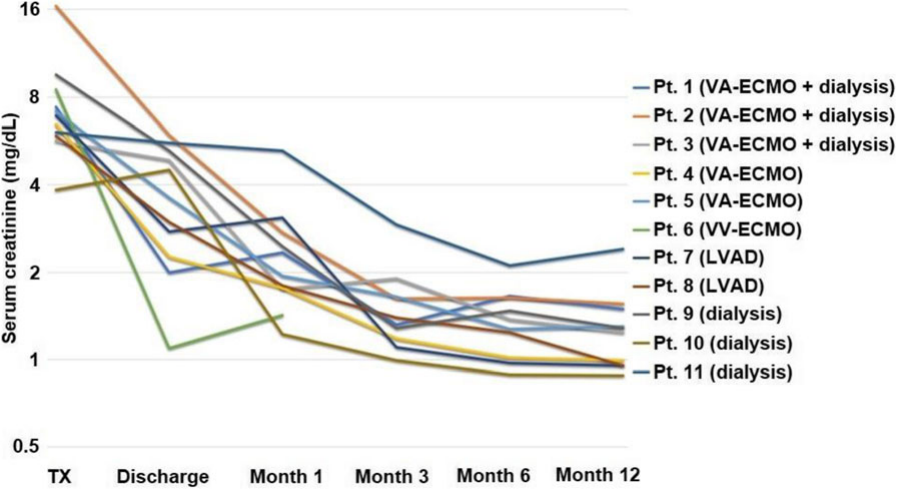
Graft function in the first year post-transplant

## P360 Apheresis or not apheresis in controlled liver donation after circulatory death: a single centre experience in Italy

### C Dallai^1^, S Baroni^2^, A Marudi^2^, S Ghedini^1^, M Talamonti^1^, S Rinaldi^2^, G Melegari^2^, E Bertellini^2^

#### ^1^Università degli Studi di Modena e Reggio Emilia, Anestesia, Rianimazione e Terapia Intensiva e del dolore, Modena, Italy; ^2^Azienda Ospedaliero-Universitaria di Modena, Departement of Anesthesia and Intensive Care, Modena, Italy

**Introduction:**

One way to expand the potential donor pool is Donation after Circulatory Death (DCD), and a strategy to reduce the complications related to the ischemic time is the use of Normothermic Regional Perfusion (NRP) with extracorporeal membranous oxygenation (ECMO) [1,2].

We compare the use of standard NRP with an effective adsorption system inflammatory mediators (CytoSorb®) in the regional normothermic reperfusion phase via regional ECMO, that involves a reduction in cellular oxidative damage, assessed as a reduction in levels of proinflammatory substances.

**Methods:**

We report a case series of 9 DCD-Maastricht IIIA category donors, treated in ECMO with NRP, to maintain circulation before organ retrieval, in association with CytoSorb® in 5 patients. During perfusion, from starting NRP (T0), blood samples are collected 3 times, every 60 minutes (T1, T2, T3).

**Results:**

During treatment with Cytosorb®, lactate levels progressively decrease, AST and ALT increase less than without Cytosorb®, as sign of improvement in organs perfusion (Figure 1).

**Conclusions:**

NRP with CytoSorb® might help to successfully limit irreversible organ damages and improve transplantation outcome [2]. Development and implementation of uniform guidelines will be necessary to guarantee the clinical use of these donor pools.

**References:**

1. Boteon YL et al. World J Transplant 9 :14–20, 2019

2. Miñambres E et al. Am J Transplant 17 :2165–2172, 2017

**Fig. 1 (abstract P360).**
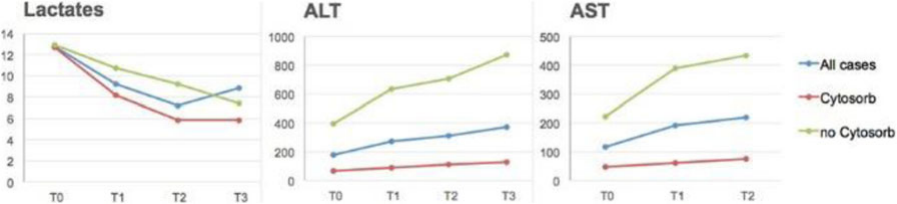
Lactate, AST and ALT levels during treatment

## P361 Gene expression profiles predict the likelihood of mortality in patients with postsurgical shock

### P Martínez-paz^1^, M Aragón-Camino^2^, A Fadrique-Fuentes^3^, M Heredia-Rodríguez^4^, E Gómez-Sánchez^2^, M Lorenzo-López^2^, E Gómez-Pesquera^2^, H Gonzalo-Benito^5^, E García-Morán^6^, E Tamayo^1^

#### ^1^Faculty of Medicine. University of Valladolid, Department of Surgery, Valladolid, Spain; ^2^Clinic University Hospital of Valladolid, Anesthesiology and Reanimation Service. Clinic University Hospital of Valladolid, Valladolid, Spain; ^3^Hospital of Medina del Campo, Anesthesiology and Reanimation Sevice, Hospital of Medina del Campo, Medina del Campo, Spain; ^4^University Hospital of Salamanca, Anesthesiology and Reanimation Service. University Hospital of Salamanca, Salamanca, Spain; ^5^Instituto de Estudios en Ciencias de la Salud de Castilla y León, Instituto de Estudios en Ciencias de la Salud de Castilla y León, Valladolid, Spain; ^6^Clinic University Hospital of Valladolid, Cardiology Service, Valladolid, Spain

**Introduction:**

Shock is a common complication of critical illness in patients in intensive care units (ICUs), who are undergoing major surgery. This condition is the most common cause of death in postsurgical ICUs. Nowadays, there are different ICU scoring systems for predicting the likelihood of mortality, such as APACHE or SOFA. Nevertheless, they are used rarely because they also depend on the reliability and predictions of physicians. In these sense, gene expression signatures can be used to evaluate the survival of patients with postsurgical shock.

**Methods:**

mRNA levels in the discovery cohort were evaluated by microarray to select the most differentially expressed genes (DEGs) between groups of those that survived and did not survive 30 days after their operation. Selected DEGs were evaluated by quantitative real time polymerase chain reactions (qPCR) for the validation cohort to determine the reliability of the expression data and compare their predictive capacity to that of established risk scales.

**Results:**

*IL1R2*, *CD177*, *RETN*, and *OLFM4* genes were upregulated in the non-survival group of the discovery cohort. Further confirmation of the predictive power of these genes was found in the validation cohort. Areas under the receiver operating characteristics curves (AUC) were 0.653 (0.535–0.771), 0.669 (0.544–0.794), 0.739 (0.628–0.850) and 0.782 (0.682–0.877) for IL1R2, CD177, RETN, and OLFM4, respectively. These values were more instructive than the classical mortality risk scores from the APACHE (0.647; 0.543–0.751) and SOFA (0.580; 0.456–0.705). Finally, a regression model was performed including gene expression values and different adjust variables, improving the AUC value up to 0.800 (0.693–0.906).

**Conclusions:**

This study offered new biomarkers based on transcriptional patterns for classifying patients with postsurgical shock as either at low or high risk of death. The results were more accurate than the other mortality risk scores APACHE and SOFA.

## P362 The prognostic ability of frailty and comorbid illness severity scores in septic shock

### M Chotalia, M Bangash, T Matthews, D Parekh, J Patel

#### University Hospitals Birmingham, NHS Foundation Trust, Critical Care and Anaesthesia, Birmingham, United Kingdom

**Introduction:**

This study evaluates the prognostic ability of frailty and comorbidity scores in patients with septic shock. The 90-day mortality rate of individual medical conditions are also compared. The burden of comorbid illness and frailty is increasing in the critical care patient population [1]. Outcomes from septic shock in patients with chronic ill-health is poorly understood.

**Methods:**

Patients admitted to the Queen Elizabeth Hospital Birmingham ICU between April 2016 and July 2019 with a diagnosis of septic shock (Sepsis 3.0) were included. Charlson Comorbidity Index (CCI) and performance status (PS) were calculated. The primary outcome was 90-day mortality. Categorical variables are presented as percentage and analysed using a chi squared test. Continuous variables are presented as median (IQR) and analysed using a Mann-Whitney test. A multivariate binary logistic regression analysis was conducted with CCI and PS, using age, APACHE II score, initial lactate, white blood cell count and C-reactive protein as other covariates. A Hosmer-Lemeshow test of >0.05 indicated good fit.

**Results:**

846 patients were admitted with septic shock. Patients were 61% male, with a median age of 60 (48-73). 35% had a hemato-oncological malignancy and 90-day mortality was 43% (Table 1). Patients who died had a higher CCI (5 [3-7] vs. 3 [1-4]; p = 0.03) and higher PS (2 [1-3] vs. 1 [0-2]; p = 0.02) than survivors. Multivariate logistic regression analysis identified that CCI (OR 1.46 [1.24-1.71]; p <0.0001) and PS (OR 1.65 [1.17-2.35]; p = 0.005) were independently associated with mortality in septic shock.

**Conclusions:**

Comorbid illness and frailty are both independently associated with mortality from septic shock. Hematological and metastatic solid organ malignancies were the medical conditions associated with the highest mortality.

**References:**

1. Haas B et al. Curr Opin Crit Care 22:500-5, 2016

**Table 1 (abstract P362).** 90-day mortality of medical conditions compared to the remaining cohort

	90-day mortality of medical condition	90-day mortality of the remaining cohort	Relative risk (+95% CI)
Hematological malignancy	66% (54/81)	40% (309/765)	1.51 (1.28-1.80); p<0.0001
Solid organ cancer	49% (105/213)	41% (258/633)	1.21 (1.03-1.42); p=0.04
Metastatic solid organ cancer	60% (34/58)	42% (329/788)	1.40 (1.11-1.77); p=0.02
End stage renal disease	52% (56/107)	42% (307/739)	1.26 (1.03-1.54); p=0.045
Chronic obstructive pulmonary disease	54% (45/83)	42% (318/763)	1.3 (1.05-1.61); p=0.04
No comorbidities	11% (6/53)	45% (357/793)	0.25 (0.12-0.54); p<0.0001
Age >80	60% (51/85)	41% (312/761)	1.46 (1.20-1.78); p=0.001

## P363 Two years follow up of 196 interstitial lung diseases patients after intensive care unit stay

### Y Tandjaoui-lambiotte^1^, F Gonzalez^1^, M Boubaya^2^, J Oziel^1^, G Van Der Meersch^1^, P Karoubi^1^, Y Uzunhan^3^, S Gaudry^1^, H Nunes^3^, Y Cohen^1^

#### ^1^Avicenne Hospital, Intensive Care Unit, Bobigny, France; ^2^Avicenne Hospital, Statistics Department, Bobigny, France; ^3^Avicenne Hospital, Pulmunology, Bobigny, France

**Introduction:**

Interstitial lung disease is a group of diseases associated with poor prognosis in the intensive care unit despite major improvement in respiratory care in the last decade. The aim of our study is to assess factors associated with hospital mortality in interstitial lung disease patients admitted in the intensive care unit and to investigate the long-term outcome of these patients.

**Methods:**

We performed a retrospective study in an intensive care unit of teaching hospital highly specialized in interstitial lung disease management between 2000 and 2014.

**Results:**

A total of 196 interstitial lung disease patients were admitted in the intensive care unit during the study period. Overall hospital mortality was 55%. Two years after intensive care unit admission, 70/196 patients were still alive (36%). One hundred eight patients (55%) required invasive mechanical ventilation of whom 80% died in the hospital (Figure 1). Acute exacerbation of interstitial lung disease was associated with hospital mortality (OR=5.4 [1.9-15.5]), especially in case of acute exacerbation of idiopathic pulmonary fibrosis. Multiorgan failure (invasive mechanical ventilation with vasopressor infusion and/or renal replacement therapy) was associated with very high hospital mortality (64/66; 97%).

**Conclusions:**

Survival after intensive care unit stay of patients with interstitial lung disease is good enough for not denying them from invasive mechanical ventilation, except in case of acute exacerbation for idiopathic pulmonary fibrosis patients. If urgent lung transplantation or extracorporeal membrane oxygenation are ruled out, multiorgan failure should lead to consider withholding or withdrawal life support therapies.

**Fig. 1 (abstract P363).**
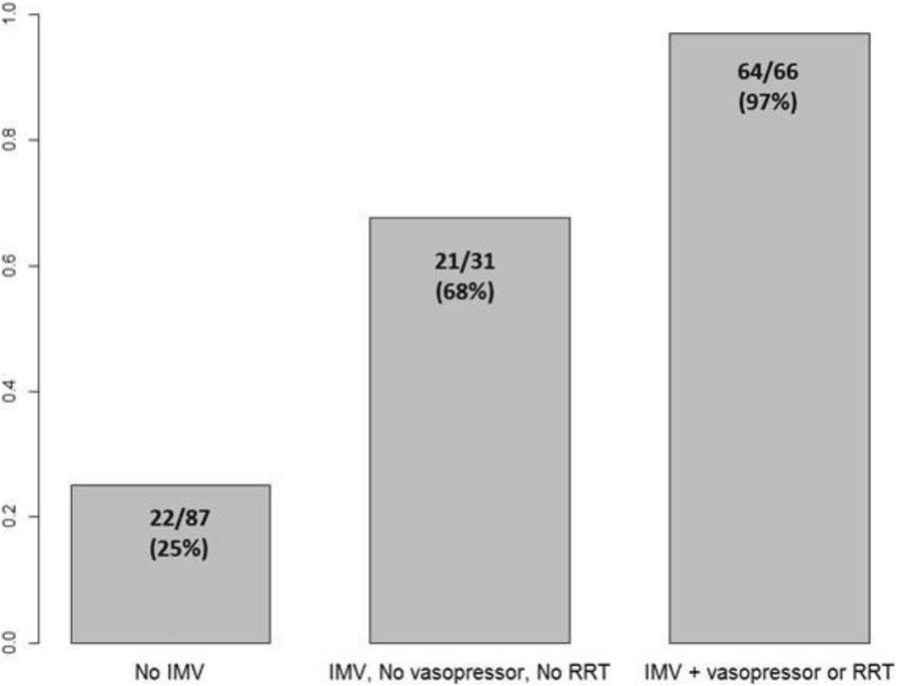
Hospital Mortality of ILD patients in ICU without ECMO according to organ support therapies

## P364 Acute gastrointestinal injury (AGI) as a predictor of malnutrition and 1-year mortality in intensive care unit (ICU). A retrospective single center study

### A Kasti^1^, M Nikolaki^2^, K Katsas^2^, F Solakis^2^, S Fotiou^2^, K Triantafylou^3^, AE Zouridaki^4^, S Patsilinakou^5^, A Armaganidis^5^, M Theodorakopoulou^6^

#### ^1^Department of Nutrition and Dietetics, Attikon University Hospital, 1. Department of Nutrition and Dietetics, Attikon University Hospital, Athens, Greece; ^2^Department of Nutrition and Dietetics, Attikon University Hospital, Athens, Greece; ^3^2nd Department of Internal Medicine, Hepatogastroenterology Unit, Attikon University Hospital, 2nd Department of Internal Medicine, Hepatogastroenterology Unit, Attikon University Hospital, Athens, Greece; ^4^Department of Human biology and Health Science University of Toronto, Toronto, Canada; ^5^2nd ICU Department, Attikon University Hospital, Athens, Greece; ^6^2nd ICU Department, Attikon University Hospital, Intensive Care Medicine, Athens, Greece

**Introduction:**

ΑGI is a malfunctioning of the GI tract in ICU patients associated with prolonged mechanical ventilation, enteral feeding failure and high mortality risk. The WGAP of ESICM proposed a grading system for AGI. Four grades of severity were identified: AGI grade I, a self-limiting condition; AGI grade II (GI dysfunction), interventions are required to restore GI function; AGI grade III (GI failure); AGI grade IV, GI failure that is immediately life threatening. The aim was to evaluate the feasibility of using AGI grades I and II as predictors of malnutrition and 1-year mortality in critically ill patients

**Methods:**

Single-center retrospective cohort study in a tertiary university hospital (2015 – 2017). AGI Grade III and IV patients were excluded. Αnthropometric data, GI symptoms (vomiting,diarrhea), feeding intolerance, gastric residual volumes and abdominal hypertension were recorded. Daily prescribed caloric intake was calculated using a standard protocol and daily achievement of caloric intake was recorded. mNutric score was calculated for all patients. A score ≤5 was used to diagnose malnutrition.

**Results:**

200 patients (59% men, mean age 65 years) that stayed in the ICU for >48 hours were included in the study. 52% were at high nutritional risk. 1-year mortality was 31%. The prevalence of AGI II was 14%. Age, gender, BMI, mortality and energy intake did not differ significantly between patients with AGI II and those with AGI I (Table 1). Logistic regression corrected for gender and age revealed that AGI II (OR: 2.91; 95%CI 1.01-8.40) was independent predictor of malnutrition.

**Conclusions:**

The AGI grading system seems useful in identifying GI dysfunction and could be used as a predictor of impaired outcomes. In this study, AGI II patients were prone to develop malnutrition with a prevalence of 14%. However association between AGI II and mortality was not established

**Table 1 (abstract P364).** Results

	AGI Grade I(N-172)	AGI Grade II(N=28)	p
Age	65(51-75)	70(57-79)	0.11
Male Gender(%)	59	50	0.35
Malnutrition Risk(%)	53	75	0.03
BMI (kg/m2)	26.4(23.5-29.9)	24.9(22.4-31)	0.70
Patients achieved energy goal (%)	39	39	0.90
1-year mortality (%)	29	41	0.3

## P365 The effect of ICU admission on frailty and activities of daily living at 6-months in patients ≥ 80 years of age

### A Smith^1^, H Flaaten^2^, B Marsh^1^

#### ^1^Mater Misercordiae University Hospital, Critical Care Medicine, Dublin, Ireland; ^2^Haukeland University Hospital, Department of Anaesthesia, Intensive Care and Clinical Medicine, Bergen, Norway

**Introduction:**

The study aimed to assess the effects of ICU admission on frailty and activities of daily living in the ≥80’s population at 6-months.

**Methods:**

A prospective observational study with data used as a subset of the VIP-2 trial [1]. Research ethics committee approval from the Mater Misercordiae University Hospital (MMUH). Inclusion criteria - ≥ 80 years of age and acute admission to ICU from May to July 2018. Data collected on 20 consecutive patients. Frailty and activities of daily living (ADL) were assessed using the Clinical Frailty Score (CFS) and the Katz Index of Independence in Activities of Daily Living (KATZ).

**Results:**

CSF pre-admission frailty was present in 60% of patients, increasing to 93% at 6 months (Figure 1). 74% of survivors at 6-months had a CFS score increase by ≥ 1 point. Pre-frail and frail CFS patients suffered an average 2-point deterioration in their Instrumental Activities of Daily Living (IADL). 60% of KATZ patients were fully functional pre-admission, deteriorating to 13% at 6 months. 74% of patients declined by 1 ADL at 6 months. 60% of the deceased were deemed fully functional initially.

**Conclusions:**

We demonstrate an association between an ICU admission event and enduring functional decline at 6 months. ICU admission resulted in patients acquiring on average 1.5 new IADL limitations despite their initial CFS. This is echoed in a study by Iwasyna et al. who also showed similar deteriorations in IADL and cognitive impairment [2]. KATZ benefits may be best used in describing functional decline. 74% of patients developed at least one new limitation. However, the CFS takes into account IADL’s and thus may be more sensitive in predicting the functional outcomes of an ICU event at 6 months.

**References:**

1. Guidet B et al. Intensive Care Med 46:57-69, 2020

2. Iwashyna TJ et al. JAMA 304:1787-94, 2010

**Fig. 1 (abstract P365).**
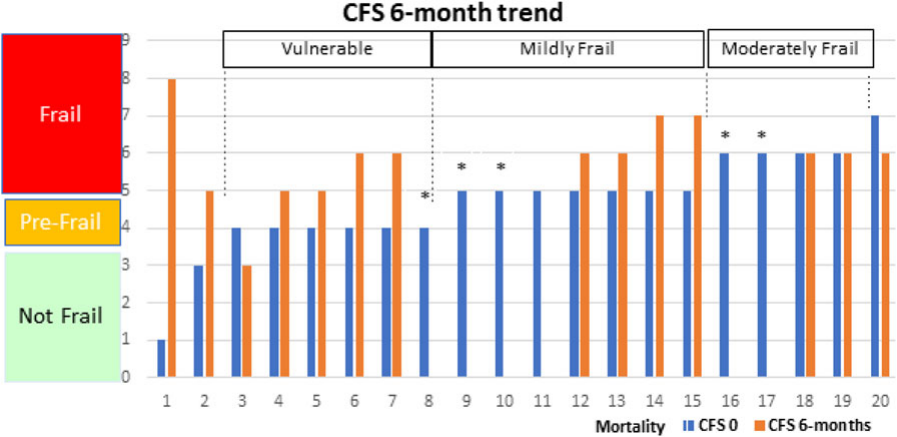
Clinical frailty score 6-month trend

## P366 Frailty: an independent factor in predicting length of stay for critically ill

### T Chandler, R Sarkar, A Bowman, P Hayden

#### Medway Maritime Hospital, Critical Care, Gillingham, United Kingdom

**Introduction:**

Frailty has attracted attention in the healthcare community in recent years, as it is associated with worse outcomes and increased healthcare costs [1]. Our objective was to study the impact of frailty as recorded by clinical frailty scale(CFS) to prospectively evaluate the effect of frailty on hospital length of stay (LOS).

**Methods:**

A retrospective analysis of consecutively admitted critical care (CC) patients’ data (Jan’19-Oct’19) was performed. Electronic health records were used to collect demographics, CFS and clinical outcomes. Statistical analysis was performed using STATA. Students T-test, simple and multiple (adjusted for age, disease severity/ICNARC score) linear regression were used for comparison between groups and to see group effect. We excluded extreme outliers (LOS>50 days; n=13). Frailty was defined as CFS>4.

**Results:**

Out of the 848 patients (male 58%), 554(66%) were emergency admissions, the rest elective (Table 1). 288(40%) were non-frail. The mean LOS were 15 days (d) ±12 and 10d±9 (P<0.0001) in the frail and non-frail patients respectively. For emergency patients, LOS were 16d(±12) and 10d(±10) for the groups, (P<0.0001). For elective patients; LOS were 12d(±10) and LOS 8d(±7), (P=0.01) for frail and non-frail respectively. After adjusting, LOS was significantly higher in frail patients by 5 days (95%CI 3,7; P<0.0001), by 4 days (95%CI 1,6; P=0.002) and by 5 days (95%CI 4,8; P<0.0001) for total cohort, elective and emergency admissions respectively. The LOS was 6 days higher in frail than non-frail (P<0.001) for CC survivors.

**Conclusions:**

Frailty was associated with significantly increased LOS in this cohort, independent of age and illness severity. Hospital capacity planning should take this into consideration when modelling bed allocation for different patient groups. Targeted resource allocation may improve this and increase healthcare utilisation.

**References:**

1. Fernando et al. Crit Care Med 47:e669-e676, 2019

**Table 1 (abstract P366).** Impact of frailty on hospital length of stay

	Non-frail	Frail	P value	Additional LOS in frail (adjusted model)
All Patient LOS (Days)	10(±9)	15(±12)	<0.0001	5.5(4,7), p<<0.0001
Elective LOS (Days)	8(±7)	12(±10)	0.001	4(1,6), p=0.002
Emergency LOS (Days)	10(±10)	16(±12)	<0.0001	6(4, 8), p<0.0001
Critical Care Survivors LOS (Days)	10 (±9)	17 (±12)	<0.0001	6(4, 8), p<0.0001

## P367 Incorporating trend analysis into the intensive care individual mortality review process

### M Titterington, M Moore, D Spray, A Crerar-Gilbert, A Dewhurst

#### St George´s University Hospitals NHS Foundation Trust, Cardiothoracic Intensive Care Unit, London, United Kingdom

**Introduction:**

Robust clinical governance requires analysis of patient outcomes during an ICU admission [1]. On one adult ICU weekly mortality meetings are used for this purpose and aid multidisciplinary reflections on individual patient deaths. However, such reviews run the risk of being subjective and fail to acknowledge themes which may relate to preceding or subsequent deaths. This paper describes a new mortality review process in which: a) reviews are structured using the Structured Judgement Review (SJR) framework [2]; and b) themes are generated over an extended period of time to create longitudinal learning from death.

**Methods:**

The SJR framework has been developed by NHS improvement for the new medical examiner role, looking at inpatient deaths. We adapted this to better suit the ICU creating a novel review structure. This involves explicit judgement comments being recorded, and the use of a scoring system to analyse the quality of care during the patient’s stay with a focus on elements of care delivered on the ICU. Tabulation of this information allows analysis over time, identifying trends across all patients, and in specific subgroups.

**Results:**

This framework has been rolled out at the St George's Cardiothoracic ICU weekly mortality meetings. Themes that have emerged include parent team ownership, delayed palliative care referrals and inadequate documentation of mental capacity. This will continue as part of a three-month trial and following review of this trial may be extended to other critical care units in the trust.

**Conclusions:**

This system allows greater insight into patient deaths in a longitudinal fashion and facilitates local identification of problems at an early stage in a way that is not possible within the traditional mortality review format. The nature of the process means that key areas for change can be identified as a routine part of the clinical week.

**References:**

1. Guidelines for the Provision of Intensive Care Services (GPICS) 2^nd^ ed (2019)

2. Hutchinson et al. BMJ Qual Saf 22:1032-40, 2013

## P368 Prediction of postoperative patient deterioration using machine learning

### T Bakkes^1^, E Mestrom^2^, N Ourahou^3^, M Mischi^1^, E Korsten^3^, P Serra^4^, A Bouwman^5^, S Turco^1^

#### ^1^Eindhoven University of Technology, Electrical Engineering, Eindhoven, Netherlands; ^2^Catharina Hospital, Intensive Care, Eindhoven, Netherlands; ^3^Catharina Hospital, Eindhoven, Netherlands; ^4^Eindhoven University of Technology, Stochastics W&I group, Eindhoven, Netherlands; ^5^Catharina Hospital, Anesthesiology, Eindhoven, Netherlands

**Introduction:**

An unexpected Intensive Care Unit (ICU) admission is indicative of a serious complication in postoperative patients. Recent studies have shown that variables relevant for the prediction of adverse events are present in the electronic patient records [1]. In this study, we evaluated three distinct machine-learning methods for predicting possible patient deterioration after surgery.

**Methods:**

The data was collected retrospectively from the Catharina hospital in Eindhoven. This dataset contained all the surgeries conducted in the hospital from 2013 up to 2017. The variables in this dataset were tested on their ability to differentiate between patients with a normal recovery versus patients with an unplanned ICU admission after being admitted to the ward. The dataset contained 44 variables related to either the preoperative screening, surgery or recovery room. All variables were tested for statistical significance using a univariate logistic regression (LR), from which a subset of 34 statistically significant (p<0.05) variables was created. These variables were used to train three different types of models, namely, the LR, support vector machine (SVM) and Bayesian network (BN). The network structure of the BN was designed using expert knowledge and the probabilities were inferred using the data.

**Results:**

The three models were validated using five-fold cross-validation, resulting in the following areas under the receiver operating characteristic curve: 0.82(0.79-0.86) for LR, 0.82(0.78-0.88) for SVM and 0.65(0.63-0.68) for BN (Fig. 1).

**Conclusions:**

The results indicate that machine learning is a promising tool for early prediction of patient deterioration. The BN was included because it permits incorporating clinical domain knowledge into the learning process. However, its performance resulted inferior to the LR and SVM. In future work, we will investigate alternative domain-aware methods, and compare the performance with that of the clinical experts.

**References:**

1. Petersen MK et al. ANZ J. Surg*.* 87:457-461, 2017

**Fig. 1 (abstract P368).**
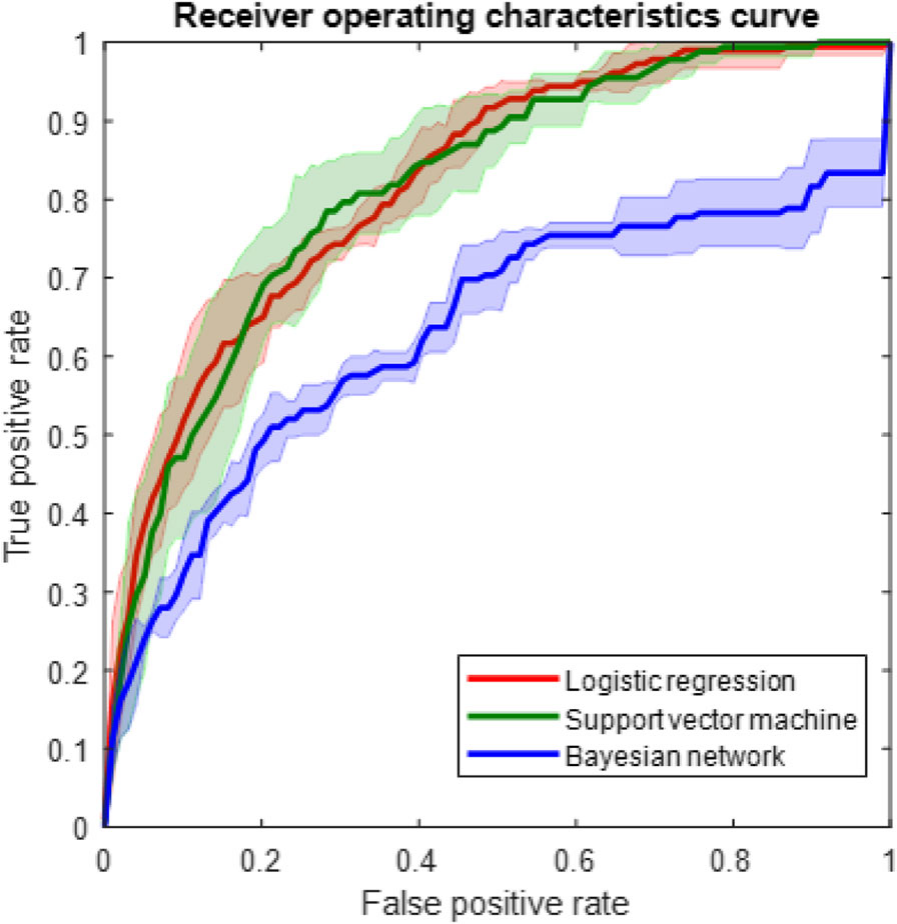
Plot of the receiver operating characteristics curves for each of the three models. The lines represents the mean of the five folds and the shaded area is the standard deviation

## P369 One-year mortality of patients with a malignancy admitted unplanned to the ICU: a large cohort study between 2008 and 2017 in the Netherlands

### EN Van der zee^1^, F Termorshuizen^2^, J Bakker^1^, DD Benoit^3^, NF De Keizer^2^, EJ Kompanje^4^, WJ Rietdijk^4^, JL Epker^4^

#### ^1^Erasmus MC University Medical Center, 1. Department of Intensive Care, Rotterdam, Netherlands; ^2^Amsterdam University Medical Center, Department of Medical Informatics, Amsterdam, Netherlands; ^3^Ghent University Hospital, 7. Department of Intensive Care, Ghent, Belgium; ^4^Erasmus MC University Medical Center, Department of Intensive Care, Rotterdam, Netherlands

**Introduction:**

Intensive Care Unit (ICU) admission decisions of patients with a malignancy can be difficult as clinicians have concerns about unfavourable outcomes, such as mortality [1]. A diagnosis of a malignancy is associated with an almost 6-fold increased likelihood of refusal of ICU admission [1]. Recent large long-term mortality studies of patients with a malignancy admitted to the ICU are scarce. Therefore, our aim was to compare mortality of patients with either a hematological or a solid malignancy to the general ICU population, all with an unplanned ICU admission.

**Methods:**

All adult patients registered in a National Intensive Care Evaluation registry with an unplanned ICU admission from 2008 to 2017 were included. Subsequently, we divided these patients into 3 cohorts: Cohort 1 (all patients with a hematological malignancy), cohort 2 (all patients with a solid malignancy), and cohort 3 (a general ICU population without malignancy). As primary outcome, we used 1-year mortality, and as secondary outcome, ICU and hospital mortality.

**Results:**

We included 10,401 (2.2 %) patients in cohort 1, 35,920 (7.6%) patients in cohort 2 and 423,984 (90.2%) in cohort 3 (Table 1). The 1-year mortality of patients of cohort 1, 2, and 3 was 60.1%, 46.2% and 28.3%, respectively (p<0.001). Age, comorbidities, organ failure, and type of admission (i.e. surgical or medical) were positively associated with 1-year mortality in all cohorts (p <0.05).

**Conclusions:**

One-year mortality is higher in both patients with a hematological malignancy and patients with a solid malignancy compared to the general ICU population. In addition, several factors were positively associated with 1-year mortality, i.e., age, comorbidities, medical ICU admission, and organ failure. Future research should focus on predictive modelling in order to identify patients with a malignancy that may benefit from ICU admission.

**References:**

1. Shimabukuro-Vornhagen A et al. CA Cancer J Clin 66:496-517, 2016

**Table 1 (abstract P369).** ICU, hospital and 1-year mortality of all cohorts. All p-values were highly significant with a p<0.001

	Cohort 1 Hematological malignancy (n=10,401)	Cohort 2 Solid malignancy (n=35,920)	Cohort 3 General ICU population (n=423,984)
ICU mortality	2969 (28.6%)	4890 (13.6%)	52864 (12.5%)
Hospital mortality	3724 (37.9%)	6916 (20%)	65026 (16.4%)
1-year mortality	5916 (60.1%)	15606 (46.2%)	111328 (28.3%)
Mean LOS ICU (days)	5.6	3.1	3.9

## P370 Drug abusers in the ICU – do they fare worse than non-abusers? A comparative retrospective study

### TM Mann^1^, M Salim^2^, N Kumar^3^, L Yu^4^, P Lin^4^, K Ho^4^, KK Kaye^3^

#### ^1^Assuta Medical Center, Department of Intensive Care, Ashdod, Israel; ^2^Detroit Medical Center, Department of Medicine, Detroit, United States; ^3^Detroit Medical Center, Department of Medicine, Detroit, United States; ^4^Wayne State University, Detroit, United States

**Introduction:**

Drug abuse is associated with immunosuppression in multiple mechanisms. Despite that, the only study retrospectively reviewing drug abusers in the ICU demonstrated less infections and better outcomes. We compared matched patient populations in order to fully understand whether drug abuse is a risk factor for infection and a predictor of poorer prognosis as is perceived by most physicians. We hypothesized that the drug abusers admitted to the ICU will fare as good as or better than non-abuser ICU patient populations.

**Methods:**

This is a prospective study done between the years 2010-2012 on the entire patient population of the Detroit Medical Center. After the drug abuse population was identified, controls were matched according to age and admission ICU units. Patients charts were reviewed and data regarding baseline demographics, infectious complication and outcome was extracted.

**Results:**

Data was retrospectively collected for 323 drug abusers and 305 matched controls. Comorbidities and hospital admission diagnosis were significantly different between the two groups. Disease severity scores were significantly higher in the drug abuser’s patient group (DAPG) on admission and during the ICU stay. DAPG had significantly more organ failure: more need for ventilation (30.5% vs 46.4% in the DAPG (p<0.001)), more ARDS (1% vs 3.7%, p=0.03), more renal failure (33% vs 45.5%, p=0.002) and more need for renal replacement therapy (6.6% vs 11.2%, p<0.05) .They had longer hospital length of stay (LOS). There was no difference in ICU or hospital mortality. Multivariable modeling did not find drug abuse to be an independent risk factor for hospital mortality, ICU mortality (Hosp: OR = 1.37, *P* = 0.3397; ICU: OR=1.43, *PP* = 0.07), but was a risk factor for a longer hospital LOS (ME=1.46, *P* < 0.0001).

**Conclusions:**

Drug abuse is not an independent risk factor for mortality or ICU LOS. Drug abusers should be evaluated like other patients based on baseline comorbidities and disease severity.

## P371 Treatment escalation plans in discharged patients from neuro and cardiac ICU

### AR Rondal Rivera

#### St George´s Hospital NHS Trust, Cardiothoracic Intensive Care Unit, London, United Kingdom

**Introduction:**

Treatment escalation plans (TEPs) are vital in communicating a ceiling of care in a case of deterioration [1]. Having a clear plan in place reduces anxiety and eases decision making for both patients and clinicians particularly at time when patient loses their capacity to participate in the decision making.

**Methods:**

This retrospective audit looked at patients in Neuro and Cardiac ICU with a 7 day stay or longer between 1/7/19 – 20/08/19. This specific length of stay is due to both units having planned admissions with a one night stay and not suitable for audit. Local database ‘Ward Watcher’ which records patient stay in ICU was used to find suitable patients. Electronic medical records were then accessed via ‘iCLIP’ to view if discharge summaries included a TEP.

**Results:**

Between 1/7/19 to 20/08/19 in Cardiac ICU 24 patients who fitted the audit criteria were identified. 10/24 (41.6%) had a TEP in place. In Neuro ICU 15 patients were found and 12 (80%) had a TEP. The combined result is 22 patients out of 39 (56%) had escalation plans in place. Few TEP were specific to what treatment would be suitable in a case of deterioration i.e fluids, non-invasive ventilation, etc.

**Conclusions:**

This is a small audit which although it did not include general ICU still reflects the need for encouraging clinicians and patients to speak freely regarding escalation plans. Medical decsions is clinician led however this audit was carried by nursing staff as we have a duty to be advocate for our patients involvement in medical care [2].

**References:**

1. Sayma M et al. Postgrad Med J 94:404-410, 2018

2. NMC (2018) ‘The Code: Professional Standards of practice and behaviour for nurses, midwives and nursing associates’.

## P372 A retrospective analysis of independent risk factors of late death in septic shock survivors

### C Sivakorn^1^, C Permpikul^2^, S Tongyoo^2^

#### ^1^Faculty of Tropical Medicine, Mahidol University, Department of Clinical Tropical Medicine, Bangkok, Thailand; ^2^Faculty of Medicine Siriraj Hospital, Mahidol University, Bangkok, Thailand

**Introduction:**

Advances in sepsis resuscitation significantly improves hemodynamic restoration. However, substantial number of patients die on the later days. We conducted a retrospective review to examine late death in these sepsis survivors.

**Methods:**

Medical records from the Department of Medicine, Siriraj Hospital during January 2012 to January 2018 were analyzed to investigate late death in sepsis survivors who had shock reversed and vasopressor weaned off more than 72 hours. Factors associated with death were also determined.

**Results:**

Of 280 sepsis survivors, 45 (16.1%) died on 28 days and 80 (28.6%) died on hospital discharge. Following shock reversal; acute kidney injury, metabolic acidosis, cardiogenic pulmonary and hospital acquired infection were the common complications. Significant independent risk factors for in-hospital mortality were hospital acquired pneumonia (HR, 3.24; 95%CI, 1.94-5.43; P<0.001), higher SOFA score after weaning off norepinephrine for 72 hours (HR, 1.12; 95%CI, 1.04-1.19; P=0.001), increased amount of fluid at stabilization or de-escalation phase (HR, 1.01; 95%CI, 1.01-1.02; P=0.008) and lower albumin (HR, 0.57; 95%CI, 0.39-0.84; P=0.004) (Table 1).

**Conclusions:**

Mortality after septic shock reversal was substantial and the main contributing factors were hospital acquired pneumonia, higher SOFA score after weaning off norepinephrine for 72 hours, increased amount of fluid at stabilization or de-escalation phase and lower albumin level. Measures to mitigate these events would be beneficial for rendering better outcomes.

**Table 1 (abstract P372).** Multivariate risk factors of late death in septic shock survivors by Cox regression analysis

Variables	Hospital Mortality Adjusted HR (95%CI)	p-value
HAP/VAP	3.24 (1.94, 5.43)	<0.001*
SOFA score at 72 hours after weaning off NE	1.12 (1.04, 1.19)	0.001*
Fluid (ml) 72 hours after discontinuation of NE	1.01 (1.01, 1.02)	0.008*
Albumin level	0.57 (0.39, 0.84)	0.004*

## P373 Can we rely on functional capacity to predict ICU mortality?

### JC Garbuglio Araujo da Silva^1^, C Coutinho^1^, MA Filho^2^, D Fernandes^1^, T Giraldi^1^, T Santos^1^

#### ^1^University of Campinas, School of medical sciences, Campinas, Brazil; ^2^University Medical Center Groningen, University Medical Center Groningen, Holland, Netherlands

**Introduction:**

The scores traditionally adopted to predict Intensive Care Unit (ICU) mortality do not take into account the functional capacity of patients. On the other hand, there are several scales for this purpose that are used in other clinical situations. This study aimed to evaluate whether the Charlson Comorbidity Index (CCI), Palliative Prognostic Score (PaP), Karnofsky (KPS) and Daily Living Activity Independence Scale (Katz Scale) can predict mortality in acutely ill medical patients.

**Methods:**

This prospective cohort study was conducted in a Medical Intensive Care Unit of a University Hospital. In six months, 114 patients were included. In addition to the above, the scales used were: Sequential Organ Failure (SOFA), Simplified Acute Physiology Scores III (SAPS III) and Acute Physiology and Chronic Health Evaluation II (APACHE II). Information on functional capacity prior to the acute illness that led to hospitalization was obtained through an interview with family members. The Research Ethics Committee of the University of Campinas approved this study.

**Results:**

The results of multivariate logistic regression analysis showed that for each point added in PaP, there was a greater chance of 37.8% for mortality (OR: 1.378; CI: 1.108 - 1.714; p= .0124). As expected, the same occurred in APACHE II, where each additional point was related to a 14.6% higher chance of mortality. (OR: 1,146; CI: 1,056-1,242; p <.0001). Regarding the Katz scale, each less point was associated with a 61.8% greater chance of mortality (OR: 1.618; CI: 1.012 - 2.381; p= .0142) (Fig. 1).

**Conclusions:**

The PaP and Katz scales seem to be adequate for predicting mortality of critically ill patients admitted to a medical ICU. This finding may help in the elaboration of future ICU mortality scoring systems, as well as in more rational use of resources. However, further multicenter studies are needed to better elucidate these results.

**Fig. 1 (abstract P373).**
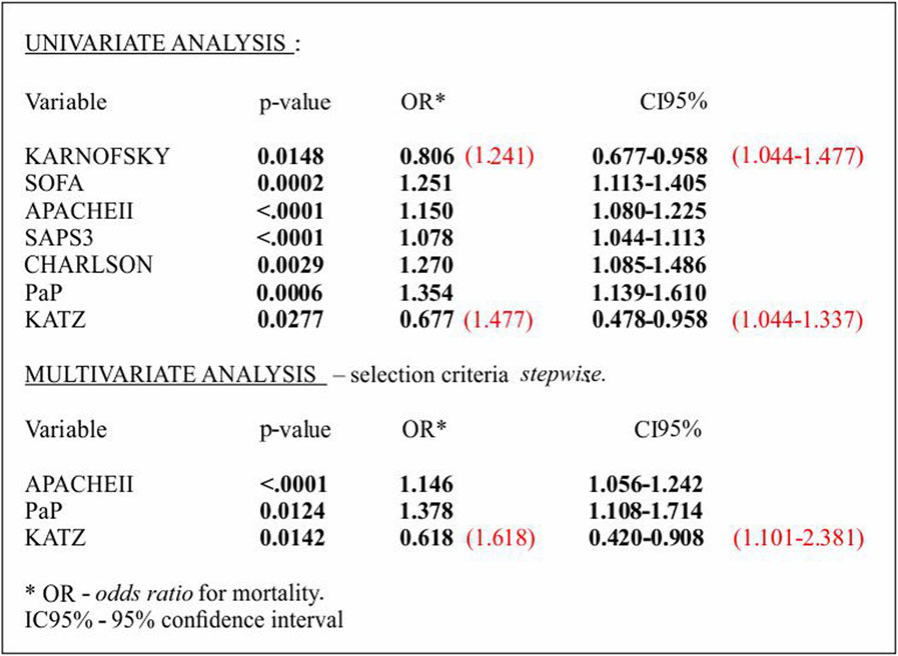
Univariate and multivariate analyses

## P374 Adherence to regulations regarding do-not resuscitate orders in a Swedish hospital

### E Piscator^1^, K Göransson^2^, E Boström^3^, K Rakovic^4^, S Forsberg^5^, J Herlitz^6^, M Holzmann^7^, T Djärv^5^

#### ^1^Karolinska Institutet, Center for Resuscitation Science, Department of Medicine Solna, Stockholm, Sweden; ^2^Karolinska Institutet, Department of Medicine, Solna, Stockholm, Sweden; ^3^Karolinska University Hospital, Function of Emergency Medicine, Solna, Stockholm, Sweden; ^4^Karolinska University Hospital, Function of Emergency Medicine, Stockholm, Sweden; ^5^Karolinska Institutet, Center for Resuscitation Science, Department of Medicine, Solna, Stockholm, Sweden; ^6^University of Borås, Center of Prehospital Research, Faculty of Caring Science, Work-life and Welfare, Borås, Sweden; ^7^Karolinska Institutet, Department of Medicine, Huddinge, Stockholm, Sweden

**Introduction:**

The purpose of this study was to establish the characteristics of patients with a do-not attempt resuscitation (DNAR) order in a Swedish hospital and to evaluate adherence to the regulations regarding DNAR orders published by the Swedish authorities. The regulations state that to the greatest extent possible, a DNAR order should be made in consent with the patient or next of kin (if consent with the patient is not achievable). Further the decision should be made after consultation with at least one other licensed caregiver.

**Methods:**

This was a retrospective analysis of a cohort of adult patients admitted to Karolinska University Hospital through the emergency department (ED) from 1^st^ of January to 31^st^ of October 2015, where at least one DNAR order was issued during the admission. Adherence to regulations was evaluated through analysis of a mandatory DNAR form filled out for every decision.

**Results:**

11% of admissions through the ED received at least one DNAR order. In total 3583 DNAR documents were issued out of whom 47% were male (p<0.001 contrasting female sex), mean age was 77 years, 47% were admitted to a general ward, 45% to a high dependency unit and the remaining to intermediate care or intensive care units. The most prevalent comorbidities were malignancy (25%), diabetes (20%) and heart failure (16%). Although 59% were discharged, one-year mortality was 79%. Consent was not possible to achieve in 40%. DNAR decisions were made without any documentation regarding consent in 43%, see table 1. Decisions were made in consultation with at least one other licensed care giver in 31%.

**Conclusions:**

Adherence to regulations by the Swedish authorities is poor and educational efforts are called upon to ensure the integrity of the patient in regards to the ethical principles of resuscitation.

**Table 1 (abstract P374).** DNAR documents for patients admitted through the emergency department at Karolinska University Hospital 1st January to 31st October 2015

DNAR documents total, No.	3583
Documented consent with pat, No.	600
Documented consent not achievable, No.	1432
Documentation regarding consent absent, No.	1551
Documented consent with pat and next of kin, No	114
Documented consent with next of kin, No	664
Documented consent other licensed caregiver, No	1101
Abbreviation: DNAR, do-not attempt resuscitation	

## P375 Ethical conflicts in decision-making about respiratory support in critical situations

### Á Estella^1^, R Viciana^2^

#### ^1^University Hospital of Jerez, Critical Care Unit. Hospital of Jerez., Jerez, Spain; ^2^University Hospital of Jerez, Oncology, Jerez, Spain

**Introduction:**

Ethic conflicts regarding emergency medicine and life-threatening situations. Complexity in the decision-making is a challenge. The aim of the present study are: To describe clinical decision making in emergency situations in different ethical scenarios that include the end of life; to analyze the influence that has the experience and the different perspectives of the healthcare professionals in the decision-making in critical situations.

**Methods:**

Cross sectional study design with closed-ended questionnaire including with four clinical cases with four answer choices describing clinical decisions among those that had to choose only one at the discretion of the participant. The health care professionals surveyed were emergency physicians, intensive care physicians, emergency nursers, Intensive Care nurses, medical residents, medicine students and master in Bioethics. This last group was chosen because of its experience and specific training in the field of bioethics as a control group or reference.

**Results:**

A total of 444 respondents participated in the study. 22.2% were emergency physicians, 14.8% intensivists, 11.2% emergency nursing, 6.2% ICU nursing, 24.9% resident doctors, 13.8% medical students and 6.9% other professions. We observed variability in the responses observed not only between different groups of professionals but even within the same group reflecting the difficulty in decision making.

**Conclusions:**

Variability was observed regarding decisions in end of life ethics conflicts. A high degree of similarity with the group of Master in Bioethics was observed in the responses issued by medicine students.

## P376 The Norwegian ETHICUS II data

### A Robertsen^1^, TA Aasmundstad^1^, BÅ Sjøbø^2^, T Legernæs^3^, M Flückiger^4^, E Søreide^5^, K Dybwik^6^, J Røe Ballestad^7^, A Haavind^8^, P Klepstad on behalf of the Norwegian ETHICUS II investigators^9^

#### ^1^Oslo University Hospital, Department of Anesthesiology and Critical Care, Oslo, Norway; ^2^Haukeland University Hospital, Department of Anesthesiology and Critical Care, Bergen, Norway; ^3^Hamar Hospital, Department of Anesthesiology and Critical Care, Hamar, Norway; ^4^Akershus University Hospital, Department of Anesthesiology and Critical Care, Oslo, Norway; ^5^Stavanger University Hospital, Department of Anesthesiology and Critical Care, Stavanger, Norway; ^6^Nordland University Hospital, Department of Anesthesiology and Critical Care, Bodø, Norway; ^7^Drammen Hospital, Department of Anesthesiology and Critical Care, Drammen, Norway; ^8^University Hospital of Northern Norway, Department of Anesthesiology and Critical Care, Tromsø, Norway; ^9^St Olav University Hospital, Department of Anesthesiology and Critical Care, Trondheim, Norway

**Introduction:**

End-of-life (E-o-L) practices varies between countries and regions [1]. Norway is an affluent, equality-based Northern European society with a public health care system. Legal frames and national guidelines on limitations of life-sustaining treatment exist. Four per cent of Norwegians dies within an ICU.

**Methods:**

An a priori post-hoc analysis of 11 Norwegian sites participating in ETHICUS II - a prospective, observational study including all consecutive ICU patients who died or had limitations of life-sustaining treatment from September 2015 to February 2016 [1].

**Results:**

Of 3298 admitted ICU patients 450 (14%) died or had limitations. Patients’ mean age was 69 years (16-96) and 90% had a chronic disease in addition to the acute condition that brought them to the ICU. Eleven per cent were assessed mentally competent. Eighty-eight per cent had limitations, 35% in the form of a sequence - decisions at multiple time points. Mutually exclusive E-o-L categories were; 33% withholding, 55% withdrawing, 0% shortening of the dying process, 4 % brain deaths, 8% failed CPR. Primary reasons behind decisions are in Table 1. E-o-L discussions were initiated in 1% by the patient, in 2% by family, in 3% by nurses and in 94% by physicians. In 57% nurses participated in discussions. In 35% physicians asked for or received information about patients’ preferences, only 2 patients had Advance Directives. Time from admittance to first limitation was 38h (25-75 percentile 6 – 133 h), from first limitation to death 21h (25-75 percentile 4-69 h). Major difficulties or disagreements occurred in 7%. Among patients with limitations the study survival (to ICU discharge or end of follow-up at 2 months from decision) was 20%.

**Conclusions:**

The Norwegian ETHICUS II cohort describe a proactive, but still somehow paternalistic E-o-L practice with room for improvement in nurse involvement and more active engagement of patients and families.

**References:**

1. Sprung C el al JAMA 322:1692-1704, 2019

**Table 1 (abstract 376).** Primary reasons behind decisions to withhold or withdraw

Reason	N	%
Unresponsive to maximal therapy	153	39
Neurological prognosis	108	27
Poor quality of life	26	7
Chronic disease	40	10
Age	9	2
Patient request	7	2
Other	53	13

## P377 Barriers and facilitators to framing inpatient goals of care: physicians’ perceptions

### A Ghosh^1^, S Lussier^2^

#### ^1^The Northern Hospital, Intensive Care Unit, VicToria, Australia; ^2^The Northern Hospital, Intensive Care, State: Victoria, Australia

**Introduction:**

Barriers and facilitators to framing goals of patient care (GOPC) and factors motivating decision making is relatively unexplored [1,2,3].

**Methods:**

A three part survey of physicians at an Australian hospital in a culturally and linguistically diverse suburb (Table 1). Identification of levels of confidence and barriers and facilitators to GOPC discussion and decision making was the main outcome measure. Factors influencing decision-making was analysed through scenarios.

**Results:**

22 out of 96 eligible participants responded; 12 female, 10 male, clinical experience 4-31 years. Level of confidence was ranked between “somewhat confident and very confident.” All but one respondent had six months of ICU experience. No differences in the level of confidence among Physician groups. 14 barriers and 8 facilitators were identified; poor prognosis and patient or family request were most common facilitators; conflict between treating teams and the patient/surrogate and language barriers were most common barriers. Factors driving GOPC decision-making included clinical, value judgement, communication, prognostication, justice and avoidance.

**Conclusions:**

Numerous barriers and facilitators were identified. Factors driving decision making did not just consider clinical factors; conflict and prognostication were important. A larger study could explore these themes and confirm findings. Factors associated with confidence could inform future training schemes.

**References:**

1. Sulmasy DP et al. J Med Ethics 34:96-101, 2008

2. Piggott KL et al. BMC Cancer 19:130, 2019

3. Schulman-Green D et al. J Palliat Care 33:143-8, 2018

**Table 1 (abstract 377).** Summary of survey questions

Demographics	Role, Qualifications, Gender, speciality training
Decision making and GOPC	Confidence, responsibility, barriers, facilitators
Discussion of GOPC	Levels of confidence, barriers, facilitators

## P378 Physician-related variability in end-of-life decisions

### LB Block^1,2^, AS Nordenskjöld Syrous^1^

#### ^1^Dpt of Anesthesia and Intensive Care, University of Gothenburg, Gothenburg, Sweden; ^2^Dpt of Anesthesia and Intensive Care, Sahlgrenska University Hospital, Gothenburg, Sweden.

**Introduction:**

We aimed to investigate physician-related factors contributing to individual variability in end-of-life (EoL) decision-making in the intensive care unit (ICU).

**Methods:**

Qualitative study with semi-structured interviews with 19 specialists in critical care, (experience 2-32 years) from 5 Swedish ICUs. Data was analyzed in accordance to principles of thematic analyses.

**Results:**

Most of the respondents felt that the intensivist’s personality played a major role in EoL decisions (Table 1). Individual variability was considered inevitable.

***Views on acceptable outcome:*** Respondents experienced that the possible outcome for patients was interpreted very differently and subjectively among colleagues, and what seemed an acceptable patient-outcome for one doctor, was not acceptable for another.

***Values:*** Most of the respondents were well aware that they might be affected by their own values and attitudes in the decision-making process. Interestingly, several respondents mentioned that they thought that patients that were marginalized by society, especially drug-abusers could be at risk for receiving decisions to limit life sustaining treatments (LST) more often than others. None of the respondents thought that their own religious beliefs played any part in decision making.

***Fear of criticism:*** Among the less experienced respondents there was a clear sense of fear of making a questionable assessment of the patient’s medical prognosis. There was a fear for criticism from colleagues that were not directly involved in the decision-making, and may have made another decision. This created a wish among younger respondents to defer or avoid participating in decision-making.

**Conclusions:**

Physician-related, individual variability in EoL decisions primarily consisted of differing views on acceptable outcome, values and fear of criticism.

**Table 1 (abstract P378).** Physician-related individual variability in end-of-life decisions

Theme	Codes	Quotes
Individual variability	Views on acceptable outcomes	“Will this patient really leave the hospital alive, and in that case, in what condition will the patient be?” (R 1)
Individual variability	Values	“I am trying to actively think about not treating marginalized people in any other way than others. But I think perhaps it may be that people like heavy drug abusers may receive more limitations than others, based on medical factors, like they probably have low quality of life, small physiological reserves and may have low compliance to any aftercare”. (R16)
Individual variability	Fear of criticism	“The fear consists of that I may misjudge the patient’s possibilities for recovery and therefore withdraw LST too early. That it could evoke criticism from colleagues that were not directly involved and might have made another decision. Also there is this fear of liability, of being subject to a process of some kind” (R4).

## P379 Can the functional capacity prior to hospitalization predict limitation of life-sustaining therapy in medical ICU patients?

### JC Garbuglio araujo da Silva^1^, C Coutinho^1^, MA Filho^2^, D Fernandes^1^, T Giraldi^1^, T Santos^1^

#### ^1^University of Campinas, School of medical sciences, Campinas, Brazil; ^2^University Medical Center Groningen, University Medical Center Groningen, Holland, Netherlands

**Introduction:**

Prognostic score systems have been widely used in intensive care units (ICU) to predict the likelihood of death [1]. However, these scores may require several laboratory data and don’t consider functional capacity. Our objective was to evaluate if the functional capacity prior to the acute illness that led to hospitalization could predict limitation of life-sustaining therapy (LLST) in critically ill medical patients.

**Methods:**

We conducted a prospective cohort study at a Medical Intensive Care Unit of a University Hospital. A total of 114 patients were included. The scales used were: Charlson Comorbidity Index (CCI), Palliative Prognostic Score (PaP), Karnofsky (KPS) and Independence Scale in Activities of Daily Living (Katz Scale). Information on functional capacity prior to hospitalization was obtained through interviews with family members. The researchers had no access to prognostic scale scores (Sequential Organ Failure -SOFA, Simplified Acute Physiology Scores III- SAPS III, Acute Physiology and Chronic Health Evaluation II- APACHE II). The LLST decisions were based on discussions during medical rounds and/or multidisciplinary clinical-bioethical meetings, when indicated. The Research Ethics Committee of the University of Campinas approved this study.

**Results:**

Multivariate logistic regression analysis showed that there is a greater 37.7% chance for LLST every 10 points less on the Karnofsky Scale (OR: 1.377; 95%; CI: 1.244 - 1.770; p= .0126) (Figure 1).

**Conclusions:**

Patients undergoing limitation of life-sustaining therapy had lower Karnofsky Scale scores. Therefore, this scale may be useful to guide end-of-life decisions in the future, but further studies with larger number of patients are needed.

**References:**

1. Batista CC et al. Brazilian Journal of Intensive Care, 21: 247-254, 2009.

**Fig. 1 (abstract P379).**
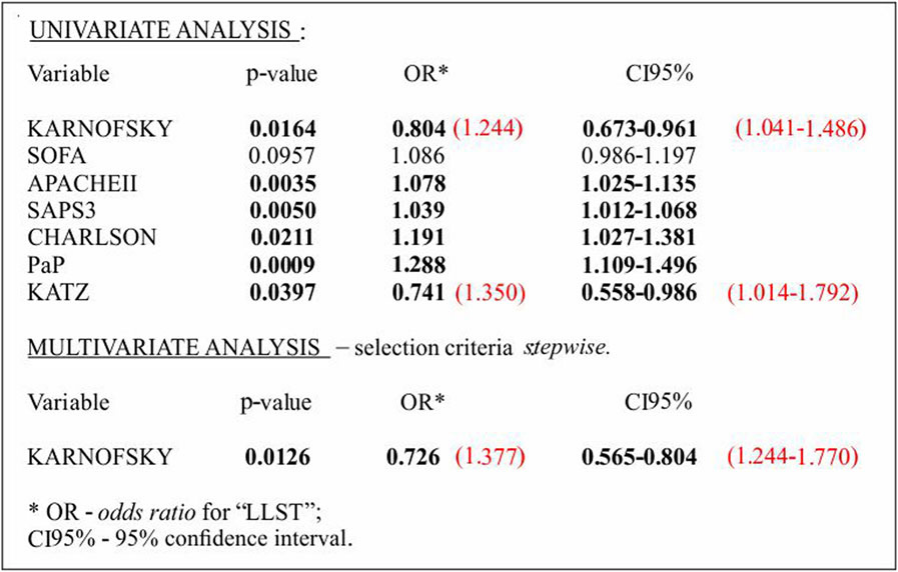
Univariate and multivariate analyses

## P380 Variation in SOFA predicted mortality based on infection site

### RD Pawar^1^, J Shih^1^, L Balaji^2^, A Grossestreuer^2^, P Patel^3^, M Donnino^4^, A Moskowitz^5^

#### ^1^Beth Israel Deaconess Medical center, Internal Medicine, Boston, United States; ^2^Beth Israel Deaconess Medical center, Emergency Medicine, Boston, United States; ^3^Beth Israel Deaconess Medical center, Pharmacy, Boston, United States; ^4^Beth Israel Deaconess Medical center, Emergency and Critical Care Medicine, Boston, United States; ^5^Beth Israel Deaconess Medical center, Pulmonary and Critical Care Medicine, Boston, United States

**Introduction:**

In this study, we investigated whether ​sequential organ failure assessment (SOFA) score performance differs based on disease state amongst patients admitted to the intensive care unit (ICU) with infection.

**Methods:**

This was a single center retrospective study of adult ICU patients admitted with infection between 2008 and 2018. Patients were uniquely classified into different disease states based on ICD9/10 codes. Disease states included were pneumonia, meningitis, isolated bacteremia, cellulitis, cholangitis/cholecystitis, intestinal and diarrheal disease, endocarditis, urinary tract infection and peritonitis. SOFA score performance was compared across disease states.

**Results:**

A total of 12,283 patients were included. Of these, 50.6% were female and the median age was 70 (IQR 57-82). The most common disease states were pneumonia (32.2%) and UTI (31.0%). Overall, 1703 (13.9%) patients died prior to hospital discharge. The mean SOFA score for the cohort was 5.4 ​(95%CI: 5.3-5.4). Patients with peritonitis had the highest mean SOFA score (6.7, 95%CI: 6.3-7.0) and patients with cellulitis had the lowest mean SOFA score (4.7, 95%CI: 4.5-5.0). SOFA score discrimination was highest among patients with endocarditis [area under the curve (AUC) 0.79, 95%CI: 0.69-0.90] and lowest for patients with isolated bacteremia (AUC 0.59, 95%CI: 0.49-0.70). Within each quartile of SOFA score, mortality was highest in patients with pneumonia and peritonitis and lowest in patients with cellulitis (see Figure 1). The addition of disease state to SOFA for the prediction of in-hospital mortality improved model discrimination (AUC 0.69, 95%CI: 0.68-0.70 vs. 0.71, 95%CI: 0.70-0.73, p<0.01).

**Conclusions:**

Site of infection is an important consideration when interpreting the SOFA score. This is an important finding as SOFA is utilized as an important tool in the definition and prognostication of sepsis.

**Fig. 1 (abstract P380).**
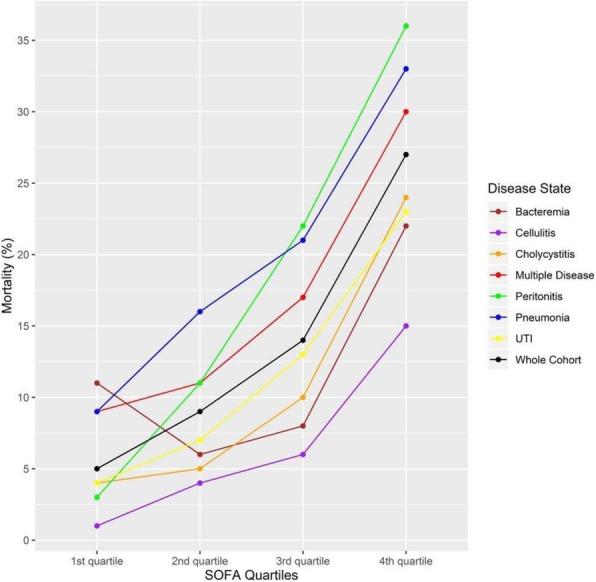
Mortality rate for disease specific SOFA score

## P381 Rethinking markers of organ failure

### D Plecko^1^, N Bennett^1^, IF Ukor^2^, R Bellomo^3^

#### ^1^ETH Zürich, Seminar für Statistik, Zürich, Switzerland; ^2^East Kent University Hospital, East Kent University Hospital, Kent, United Kingdom; ^3^Monash University, Faculty of Medicine, Melbourne, Australia

**Introduction:**

The Sepsis-3 Consensus definition identified organ dysfunction as the hallmark feature of sepsis [1]. In developing Sepsis-3, the sequential organ failure assessment (SOFA) score was chosen for its prognostic value and relative ease of implementation clinically [2]. We propose an update based on epidemiologic data from two intensive care databases that more effectively captures organ dysfunction in the context of Sepsis-3.

**Methods:**

Using the MIMIC-III (exploration) and e-ICU (validation) databases, we extracted patients with suspicion of infection to form the study cohort. The predictive power of each SOFA component was assessed using the area under the curve (AUC) for in-hospital mortality. A logistic model with the LASSO penalty was used to find an alternative statistically optimal score.

**Results:**

By utilising alternate markers of organ dysfunction (e.g. lactate, pH, urea nitrogen) we demonstrated a significant improvement in AUC for several versions of the new score, SOFA2.0 (Figure 1).

**Conclusions:**

The SOFA score can be updated to reflect current advances in clinical practice. Using epidemiologic data, we have shown that substitution of existing components with more powerful measures of organ dysfunction may provide an improved score with greater predictive power. Moreover, SOFA 2.0 exhibits equivalent ease of implementation, but better reflects organ dysfunction in the context of Sepsis-3.

**References:**

1 Singer M et al. JAMA 315: 801-810, 2016

2 Vincent JL et al. Intensive Care Med 22: 707-710, 1996

**Fig. 1 (abstract P381).**
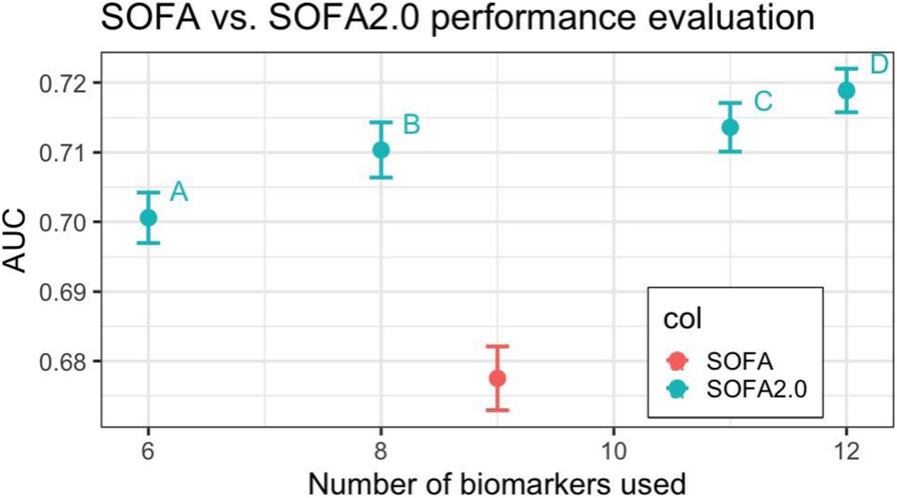
Standard deviations shown as vertical bars. SOFA2.0 version B, for instance, outperforms the SOFA score using fewer biomarkers

## P382 Acute organ failure in cancer patients while on systemic cancer treatment, a cohort study

### S Coelho^1^, MT Ribeiro^2^, I Pereira^1^, D Duarte^2^, A Afonso^1^, I Vieira^1^, S Pinelas^1^, B Pereira^3^, N Sousa^1^, F Filomena^3^

#### ^1^Instituto Português de Oncologia do Porto, Medical Oncology, Porto, Portugal; ^2^Instituto Português de Oncologia do Porto, Oncohematology, Porto, Portugal; ^3^Instituto Português de Oncologia do Porto, Intensive Care Unit, Porto, Portugal

**Introduction:**

Risk of acute organ failure (AOF) in cancer patients(pts) on systemic cancer treatment isunknown. However, 5% of non-hematologic and 15% of hematologic cancer pts will need admission to intensive care unit (ICU). IPOP-SCI-2017/01 is a prospective cohort study designed to ascertain the cumulative incidence of AOF in adult cancer pts.

**Methods:**

Single centre prospective cohort study with consecutive sampling of adult cancer pts admitted for unscheduled inpatient care while on, or up to 8 weeks after, systemic cancer treatment. Primary endpoint was AOF as defined by quick SOFA. Six months accrual expected an accrual of 400 pts to infera population risk AOF with a standard error of 1%.

**Results:**

Between 08/2018 and 02/2019 10392 pts were on systemic anticancer treatment, 358 had unscheduled inpatient care and were eligible for inclusion and 285 were included. Median age was 64 years, 51% were male, 52% had adjusted Charlson Comorbidity Index (CCI) &gt; 3 and hematologic cancers accounted for 22% of pts. The cumulative risk of AOF on hospital admission was 35% (95%CI: 31-39); and of AOF during hospital stay was 40% (95%CI: 35-44). AOF was associated with older age, CCI &gt; 3,hematologic malignancy, shorter median time from diagnosis and &gt; 1 prior line of therapy. On admission, 62% of pts were considered not eligible for artificial organ replacement therapy (noAORT) and 34% of pts who developed AOF while inhospital were judged noAORT. Overall, 17 (15%) of AOF pts wereadmitted to ICU, 31.5% for AORT. Median follow up 9.5 months (min 6; max 12). Inpatient mortalitywas 18%, with ICU mortality rate of 59%, with median cohort survival 4.5 months (95%CI: 3.5-5.4). On multivariate analysis, AOF was an independent poor prognostic factor (HR 1.6; 95%CI 1.2-2.1).

**Conclusions:**

Risk of AOF in cancer pts admitted for unscheduled inpatient care while on systemictreatment is 35%, and risk of ICU is 15%. AOF in cancer pts was an independent poor prognostic factor.

## P383 A severity-of-illness score in patients with tuberculosis requiring intensive care

### U Lalla, E Irusen, B Allwood, J Taljaard, C Koegelenberg

#### Tygerberg Academic Hospital, Internal Medicine, Division of Pulmonology and ICU, Cape Town, South Africa

**Introduction:**

We previously retrospectively validated a 6-point severity-of-illness score aimed at identifying patients at risk of dying of tuberculosis (TB) in the intensive care unit (ICU). Parameters included septic shock, human immunodeficiency virus with CD_4_<200/mm^3^, renal dysfunction, ratio of partial pressure of arterial oxygen to fraction of inspired oxygen (PaO_2_:FiO_2_) <200mmHg, diffuse parenchymal infiltrates and no TB treatment on admission. The aim of this study was to validate and refine the severity-of-illness score in patients with tuberculosis requiring intensive care.

**Methods:**

We performed a prospective observational study with a planned *post-hoc* retrospective analysis**,** enrolling all adult patients with confirmed TB admitted to the medical intensive care unit from 1 February 2015 to 31 July 2018. Descriptive statistics and Chi-square or Fisher’s exact tests were performed on dichotomous categorical variables, and t-tests on continuous data. Patients were categorized as hospital survivors or non-survivors. The 6-point score and the refined 4-point score were calculated from data obtained on ICU admission.

**Results:**

Forty-one of 78 patients (52.6%) died. The 6-point scores of non-survivors were higher (3.5+/-1.3 vs 2.7+/-1.2; p=0.01). A score ≥3 vs. <3 was associated with increased mortality (64.0% vs. 32.1%; OR 3.75; 95%CI, 1.25-10.01; p=0.01)(Table 1). *Post-hoc*, a PaO_2_:FiO_2_ <200mmHg and no TB treatment on admission failed to predict mortality whereas any immunosuppression did. A revised 4-point score (septic shock, any immunosuppression, acute kidney injury and lack of lobar consolidation) demonstrated higher scores in non-survivors (2.8+/-1.1 vs. 1.6+/-1.1; p<0.001). A score ≥3 vs. ≤2 was associated with a higher mortality (78.4% vs. 29.3%; OR 8.76; 95%CI, 3.12-24.59; p<0.001) (Table 1).

**Conclusions:**

The 6-point severity-of-illness score identified patients at higher risk of death. We were able to derive and retrospectively validate a simplified 4-point score with a superior predictive power.

**Table 1 (abstract P383).** Mortality rates: survivors vs. non-survivors (per patient, n=78)

	Total (n=78)	Survivors (n=37)	Non-survivors (n=41)	Mortality (%)
6-point severity-of-illness score:				
<3	28	19	9	32.1%
≥3	50	18	32	64.0%
Revised 4-point severity-of-illness score:				
≤2	41	29	12	29.3%
≥3	37	8	29	78.4%

## P384 The impact of chronic critical illness on survival and quality of life of patients after ICU discharge - a defining analysis of chronic critical illness

### P Berto^1^, J Haas^2^, R Rosa^3^, C Teixeira^1^, T Cavalcanti^3^, D Sganzerla^3^, T Lisboa^1^, G Friedman^4^

#### ^1^Universidade Federal do Rio Grande do Sul, Porto Alegre, Brazil; ^2^Hospital de Clínicas de Porto Alegre, Porto Alegre, Brazil; ^3^Hospital Moinhos de Vento, Porto Alegre, Brazil; ^4^Universidade Federal do Rio Grande do Sul, PPG Ciências Pneumológicas, Porto Alegre, Brazil

**Introduction:**

Chronic critical illness remains a scientific challenge, from its conceptualization to its impact on patient prognosis [1]. We evaluated the long-term evolution of ICU survivors by identifying the real burden of prolonged critical illness on survival, quality of life and hospital readmissions.

**Methods:**

We conducted a prospective cohort in 16 Brazilian hospitals including 1616 ICU survivors with an ICU stay > 72h. We compared the patients diagnosed with chronic critical illness with the other patients. Telephone Follow-up at 3 and 6 months. Quality of life was measured by the SF-12 questionnaire.

**Results:**

It was observed that 38% of patients had some definition of chronic critical illness. Chronic critically ill patients had higher mortality at 6 months (p=0.012). This difference is mainly due to higher intrahospital mortality (p=0.0001). Mortality after hospital discharge was similar between groups. There was no difference in hospital readmission rate at 6 months. Regarding quality of life, patients had similar values in both mental and physical domains.

**Conclusions:**

Chronic critically ill patients have a shorter survival when compared to patients with ICU stay greater than 72h. However, among survivors, there was no difference in hospital readmission rates and quality of life at 6-month follow-up.

**References:**

1: Rosa RG et al. Crit Care Med 48 :64-72, 2020

## P385 Simple predictive score for pulmonary complications in mechanically ventilated patients in surgical intensive unit

### A Piriyapatsom, O Chintabanyat, A Trisukhonth, O Chaiwat, S Kongsayreepong

#### Faculty of Medicine Siriraj Hospital, Anesthesiology, Bangkok, Thailand

**Introduction:**

Various scores are developed to predict pulmonary complications such as ARISCAT for patients at-risk of postoperative pulmonary complication [1] and LIPS for patients at-risk of lung injury [2]. The aim of this study was to compare these scores with ours for predicting pulmonary complications in mechanically ventilated patients in SICU.

**Methods:**

This prospective observational study was conducted in SICU at a university hospital. Adult patients admitted to SICU and required mechanical ventilation >24 hours were included. Primary endpoint was the composite of pulmonary complications including pneumonia, ARDS, atelectasis, reintubation, and tracheostomy. Multivariate analysis was performed to identify risk factors of pulmonary complications and the predictive score was developed. The ROC analysis was performed to compare power of ARISCAT, LIPS and our newly developed score for predicting pulmonary complications.

**Results:**

276 patients were included in this study. Pulmonary complications occurred in 86 (31.2%) patients. Independent risk factors for pulmonary complications included age >65 years old (OR 1.80, 95% CI 1.02-3.19), P/F ratio <300 (OR 2.32, 95% CI 1.31-4.13) and abnormal chest radiograph (OR 2.72, 95% CI 1.48-5.01). The AUROC of ARISCAT, LIPS and our score for predicting pulmonary complications were 0.51 (95% CI 0.44-0.59), 0.58 (95% CI 0.51-0.65) and 0.70 (95% CI 0.63-0.77), respectively. For our predictive score, 0.6, 0.8 and 1.0 point were assigned for presenting of age >65 years old, P/F ratio <300, and abnormal chest radiography, respectively. The score of 0.7 yielded sensitivity and specificity of 62.8% (95% CI 51.6%-72.8%) and 64.7% (95% CI 57.4-71.4%), respectively.

**Conclusions:**

Compared to ARISCAT and LIPS, our simple score had better power for predicting pulmonary complications in mechanically ventilated patients in SICU.

**References:**

1. Mazo V et al. Anesthesiology 121:219-231, 2014

2. Gajic O et al. Am J Respir Crit Care Med 183:462-470, 2011

## P386 Five-year impact of ICU-acquired neuromuscular complications: a prospective, observational study

### N Van Aerde^1^, P Meersseman^2^, Y Debaveye^1^, A Wilmer^1^, J Gunst^1^, MP Casaer^1^, F Bruyninckx^3^, R Gosselink^4^, G Van den Berghe^1^, G Hermans^1^

#### ^1^KU Leuven, Department of Cellular and Molecular Medicine, Leuven, Belgium; ^2^KU Leuven, Medical Intensive Care Unit, Leuven, Belgium; ^3^UZ Leuven, Department of Physical Medicine and Rehabilitation, Leuven, Belgium; ^4^KU Leuven, Department of Rehabilitation Sciences, Leuven, Belgium

**Introduction:**

We assessed the independent association of ICU-acquired neuromuscular dysfunction with 5-year mortality and morbidity. We explored the optimal threshold of the Medical-Research-Council (MRC) sum-score predicting 5-year outcome.

**Methods:**

Prospective, 5-year follow-up study of 883 EPaNIC patients (Clinicaltrials.gov:NCT00512122), systematically screened in-ICU for strength with MRC (‘MRC-cohort’, N=600), for electrophysiology changes with compound muscle action potential on day 8±1 (‘CMAP-cohort’, N=689), or both (‘MRC&CMAP-cohort’, N=415). Associations of MRC close to ICU discharge and abnormal CMAP on day 8±1 with 5-year mortality, hand-grip-strength (HGF, %pred), six-minute-walk-distance (6-MWD, %pred), and physical function of the SF-36 quality-of-life questionnaire (PF SF-36) at 5-year follow-up were assessed with adjusted Cox proportional hazards and linear regression. The optimal threshold for MRC close to ICU-discharge predicting adverse 5-year outcome was derived from Local-regrESSion lines on residual- and scatterplots.

**Results:**

Lower MRC [HR, per-point-increase:0.946 (95%CI:0.928-0.968), p=0.001] and abnormal CMAP [HR:1.568 (95%CI:1.165-2.186), p=0.004] independently associated with 5-year mortality in respective cohorts. In the MRC&CMAP-cohort, only lower MRC [HR:0.956 (95%CI:0.934-0.980), p=0.001] independently associated with 5-year mortality. Among 205 5-year survivors, only lower MRC independently associated with low HGF [0.866 (95%CI:0.237-1.527), p=0.004], low 6-MWD [105.1 (95%CI:12.1-212.9), p=0.043], and low PF-SF-36 [-0.119 (95%CI:-0.186 to -0.057), p=0.002]. MRC≤55 best predicted poor 5-year outcome. Both MRC≤55 and abnormal CMAP independently associated with 5-year mortality.

**Conclusions:**

ICU-acquired neuromuscular complications may impact 5-year morbidity and mortality. MRC if slightly reduced, may affect long-term mortality, strength, functional capacity and physical function, whereas abnormal CMAP only related to long-term mortality.

## P387 Mortality in high-risk admissions in low-volume versus high-volume intensive care units

### D Burke^1^, R Dwyer^2^

#### ^1^Beaumont Hospital, Intensive Care Unit, Dublin, Ireland; ^2^Beaumont Hospital, Beaumont Hospital, Dublin, Ireland

**Introduction:**

Outcomes in intensive care units have been reported to be better in higher-volume units [1,2]. We compared outcomes for high-risk patients between low and higher volume units.

**Methods:**

Audit data from Irish ICUs is analysed and reported by the Intensive Care National Audit & Research Centre (ICNARC) in London. ICNARC report risk-adjusted mortality rates in all patients and in low-risk patients(predicted mortality rate <20%) for each Unit, using the ICNARCH-2015 model to predict the risk of death. We used this data to calculate the proportion of high-risk patients(predicted mortality >20%) in each Unit, the mortality rate for high-risk patients, the risk-adjusted mortality rate and we compared the overall risk-adjusted mortality between low and high volume units.

**Results:**

The median number of annual new-patient admissions among 18 participating Units was 390; units below this were defined as low-volume and those above as high-volume Units. The proportion of all admissions to each Unit who were high-risk ranged from 8% to 54%(mean 34%). Unit mortality rates for high-risk patients ranged from 33% to 69%. The ratio of observed to expected mortality(Standardized Mortality Ratio - SMR) for high risk admissions in each Unit ranged from 0.87 to 1.34(mean 1.07). In Fig. 1 Units are ordered 1 to 18 on the x axis according to increasing volume of admissions i.e. Unit 1 was smallest and Unit 18 largest. There was no definite relationship between volume of patients admitted to a Unit and SMR in high risk admissions. Larger units had more variability in SMR. The overall SMR for all high-risk patients in all low-volume Units was 1.07 versus 1.03 for all patients in all high-volume Units.

**Conclusions:**

There was no difference in mortality for high-risk patients between high-volume and low-volume Units.

**References:**

1. Pronovost PJ et al. JAMA 281:1310-7, 1999

2. Kahn JL et al. N Eengl J Med 355:41-50, 2006

**Fig. 1 (abstract P387).**
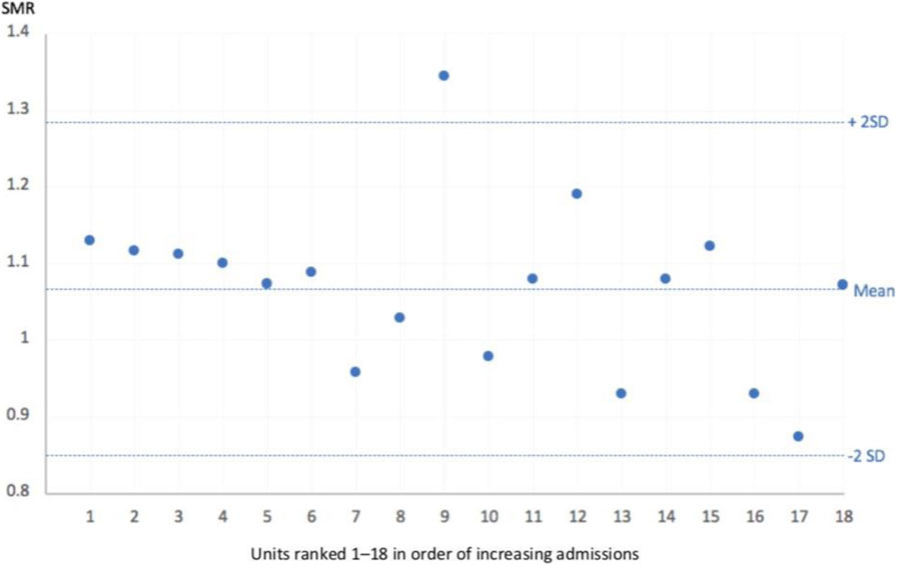
Risk adjusted mortality for high risk admissions

## P388 Cognitive and mental assessment in a general intensive care unit (ICU) population one year after hospital discharge: preliminary results of a prospective study

### V Mantziou^1^, A Kotanidou^1^, E Jahaj^1^, AG Vassiliou^2^, E Kampisiouli^1^, I Dimopoulou^1^

#### ^1^National & Kapodistrian University of Athens, 1st Department of Critical Care Medicine & Pulmonary Services, Athens, Greece; ^2^National & Kapodistrian University of Athens, 1st Department of Critical Care Medicine & Pulmonary Services, GP Livanos and M Simou Laboratories, Athens, Greece

**Introduction:**

Critical illness survivorship increases and patients and health care providers have to face its long-term sequelae. Post Intensive Care Syndrome (PICS) describes emerging or worsening physical, cognitive or mental dysfunctions, arising after severe illness and persisting for a long time following hospital discharge, interfering with patients’ quality of life.

**Methods:**

In this prospective study we evaluated cognitive ability and mental health status in ICU survivors one year after hospital discharge. Eligible patients were 18-70 years old, requiring mechanical ventilation for more than 3 days. Patients completed the Mini-Mental State Examination (MMSE). The maximum MMSE score is 30 points. A score of 20-24 suggests mild dysfunction, 13-20 moderate disability, and <12 severe dysfunction. Patients also completed the Centre for Epidemiological Studies-Depression Scale (CES-D). Scores ≥16 indicate the presence of depressive symptoms. Finally, the State-Trail Inventory (STAI), comprised of two separate scales for measuring state (S) or trait (T) anxiety, was also completed. A cut-off of 40 detects clinically significant symptoms. Questionnaires were completed in the presence of an experienced psychologist.

**Results:**

Forty-one (23 F) ICU survivors with a mean age of 43±16 years were studied. They consisted of medical (n=17), trauma (n=15), surgical (n=8), or burn (n=1) patients. Median ICU stay was 12 (IQR: 4-66) days. MMSE score was 28±3; in 5 patients (12%) MMSE score was ≤ 24. CES-D was 17±12; 18 patients (44%) had high scores. Mean S-anxiety scale was 50±8; 39 patients (95%) had high scores. Mean T-anxiety scale was 44±6.

**Conclusions:**

One year after hospital discharge, ICU survivors exhibit symptoms consistent with PICS, involving primarily mental health, with anxiety and/or depression being prominent. Cognitive ability seems to be less commonly affected. It remains yet unclear whether interventions can ameliorate functional capacity in ICU survivors.

## P389 The predictive value of phase angle on 6 month survival after ICU admission

### F Stellingwerf, M Koopmans, H Buter, EC Boerma

#### Medical Center Leeuwarden, Intensive Care, Leeuwarden, Netherlands

**Introduction:**

Phase angle, derived from bioimpedance analysis (BIA), reflects tissue quality and quantity in which cell mass, membrane integrity and hydration state are represented. Phase angle, as a measure of body composition, changes with the physical condition of patients and is associated with survival in several disease states and during ICU admission. We questioned whether phase angle can be used as a predictor of long term ICU outcome.

**Methods:**

A single-center prospective observational cohort with consecutive patients, admitted to the ICU between June 2018 and June 2019. Demographic data, APACHE III, comorbidity and phase angle in the first 6 hours after ICU admission were collected and the ICU-, hospital- and 6 months survival were registered.

**Results:**

Of 1025 patients included 8.5% (87) died within 6 months after ICU admission. Patients who died had a higher APACHE III score (95 [71-128] versus 56 [46-68] p<0.001), and more often sepsis or malignancy. The phase angle was lower among non survivors (4.9˚ [4.0-5.9] versus 5.4˚ [4.7-6.4] p< 0.001). The cut-off value for the phase angle, determined with the ROC is 4.6˚. The Kaplan Meier curve (Figure 1) showed a significant difference (Log rank p<0.001) in mortality between patients with a phase angle beneath 4.6˚.

**Conclusions:**

A low phase angle at time of ICU admission is a predictor of long term survival, with an optimal cut-off value of 4.6˚.

**Fig. 1 (abstract P389).**
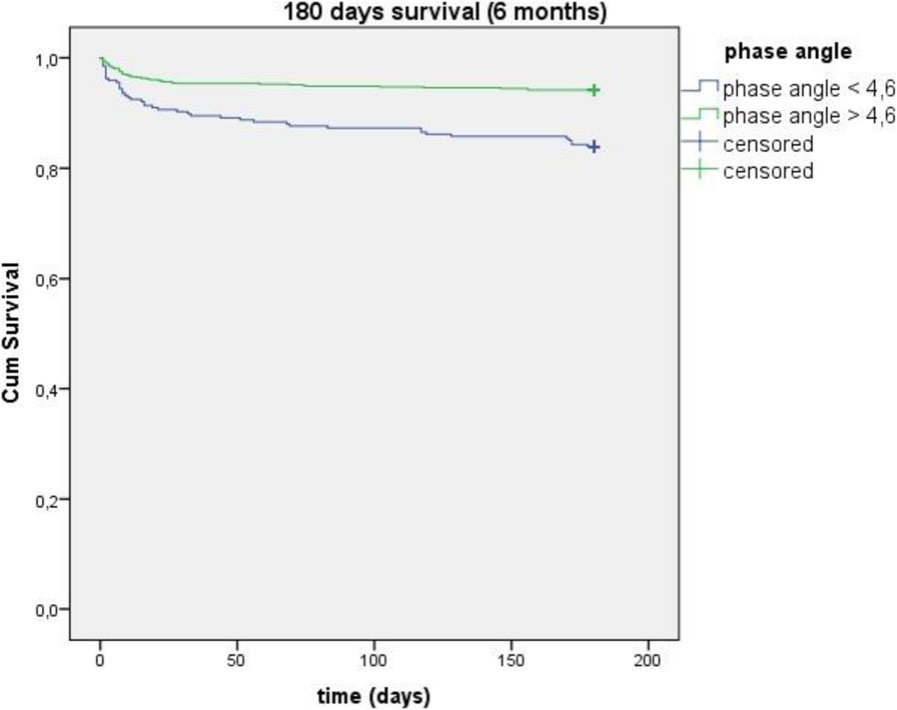
Kaplan Meier curve of 6 months survival phase angle

## P390 Risk factors for weakening of activity of daily living during admission after acute care with infection

### H Nakano, M Mochizuki, H Naraba, Y Takahashi, T Sonoo, H Hashimoto, K Nakamura

#### Hitachi General Hospital, Department of Emergency and Critical Care Medicine, Hitachi, Japan

**Introduction:**

ADL weakening is often seen after intensive care and called post-intensive-care syndrome (PICS). This is also seen in even outside ICU and proposed to be called post-acute-care syndrome (PACS), especially in elderly patients. In patients with infection, SOFA score is famous for predicting in-hospital mortality, but there are no tools for predicting ADL weakening during admission. To search for risk factors for ADL weakening during admission other than the age, we conducted a retrospective observational study.

**Methods:**

The subjects were surviving patients with infection, aged from 16 to 89 who were admitted to our department from April 1, 2018 to May 31, 2019. Information of basic characteristics, laboratory data on admission and adjunctive therapies were extracted from our database. We use Barthel Index (BI) as ADL evaluation, and the BI at discharge were evaluated by nurses. We stratified patients by BI at discharge of over 60 or not, and investigated factors that predicted it. We compared each factor between 2 groups, and perform a logistic regression analysis with those that had a significant effect clinically or statistically.

**Results:**

There were total 2170 patients and 515 had infection. The number of surviving patients were 397. Average age was 73.18, female was 41.6% and median SOFA was 4. BI was over 60 in 192 patients. Factors which had significant difference between 2 groups were age, existence of DIC and laboratory data on admission such as ALB, BUN, FDP, TAT, soluble fibrinmonomer complex and thrombomodulin. There was no relationship between ADL weakening during admission and ICU admission. We performed a logistic regression analysis with age, sex, SOFA, DIC, mechanical ventilation, renal replacement therapy, ECMO, ALB, lymphocyte count and CRP. Independent risk factors were age, sex, and ALB.

**Conclusions:**

Among patients with infection, there was ADL weakening as PACS, and its risk factor other than age was female sex and poor nutritional status on admission.

## P391 Does cognitive function play a role in readmission after ICU discharge? A pilot study

### C Robinson^1^, JM McPeake^1^, P MacTavish^1^, T Quasim^2^, P Henderson^2^

#### ^1^NHS Greater Glasgow and Clyde, NHS Greater Glasgow and Clyde, Glasgow, United Kingdom; ^2^University of Glasgow, NHS Greater Glasgow and Clyde, Glasgow, United Kingdom

**Introduction:**

Unplanned readmission to acute care following hospital discharge after critical illness has a significant impact for patients, caregivers and the healthcare system [1]. Drivers for unplanned readmission include pre-ICU functional status as well as the social circumstances which a patient returns to [2]. At present, there is limited understanding of the impact of cognitive function and whether this plays a part in the readmission process. The aim of this study was to undertake cognitive screening during the readmission episode to understand if ongoing cognitive impairment was still present in this patient group.

**Methods:**

Ethical approval was granted from the West of Scotland Research Ethics Service (approval number 19/WS/0091). We used a single centre cohort study design. Using a clinical database, we identified those patients who had an ICU admission ≥ 3 days who were readmitted to acute care within 90 days of hospital discharge. The researcher attended the ward and after discussion with the current direct care team and screened patients using the Mini Mental State Exam (MMSE) tool [3].

**Results:**

The cut-off scores were: 24-30- have normal cognition; 19-2- mild cognitive impairment; 10-18- moderate impairment and ≤ 9 severe impairment. Baseline demographics of this cohort are shown in Figure 1. 11 patients were screened during a readmission to acute care. 8 patients were found to have normal cognition (72.7%). 23.3% of patients had some degree of cognitive impairment: 2 (18.2%) had mild cognitive impairment and 1(9.1%) had moderate impairment.

**Conclusions:**

In conclusion, patients discharged from ICU have continuing cognitive impairment. Future research should examine to establish whether cognitive dysfunction after ICU is a driver for readmission to acute care.

**References:**

1. Lone NI et al. Thorax 74:1046-1054, 2019

2. Donaghy E et al. BMJ Qual Saf 27:915-927, 2018

3. Folstein M et al. J Psychiatr Res 3:189-198, 1975

**Fig. 1 (abstract P391).**
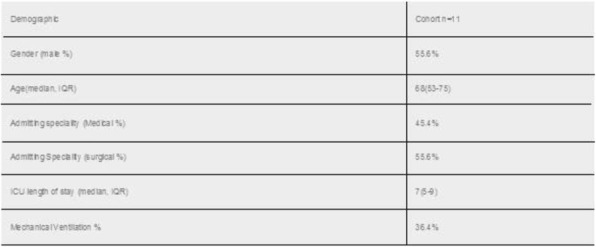
Baseline demographics

## P392 Readmission after discharge home from critical care: a qualitative study

### C Robinson^1^, F Nicolson^1^, P MacTavish^1^, T Quasim^2^, JM McPeake^1^

#### ^1^NHS Greater Glasgow and Clyde, NHS Greater Glasgow and Clyde, Glasgow, United Kingdom; ^2^University of Glasgow, NHS Greater Glasgow and Clyde, Glasgow, United Kingdom

**Introduction:**

Readmissions to acute care occur in a high number of critically ill patients within 90 days of hospital discharge [1]. Biomedical drivers such as frailty and pre-existing co-morbidities have been identified as drivers for readmission. However at present there is limited data on the influence of social problems on readmission.

**Methods:**

This study, using a grounded theory approach, sought to understand from a patient/caregiver perspective what the drivers for readmission to acute care were. Ethical approval was granted from the West of Scotland Research Ethics Service (19/WS/0091). A grounded theory approach was used to explore from a patient and caregiver perspective what the drivers for readmission are [2]. Using a clinical database, we identified those patients who had an ICU admission ≥ 3 days who were readmitted to acute care within 90 days of hospital discharge. The researcher attended the ward and after discussion with the direct care team conducted a semi-structured interview with patient and/or caregiver. The interview was recorded and transcribed verbatim. The transcripts were analysed to generate initial codes, followed by the development categories and sub-categories. Theoretical sampling was undertaken.

**Results:**

15 participants were interviewed.10(66.6%) were patients and 5 (33.3%) were caregivers. The themes that have emerged from the data were: Pain and polypharmacy; lack of social support and/or isolation; strained relationships with primary care providers and information provision across the patient journey. Subsequent theory development is underway to understand how this learning could help reduce readmissions in future.

**Conclusions:**

In conclusion, both social and biomedical drivers are likely to contribute to acute care readmission in this group. Future interventional work is required in order to identify modifiable factors to reduce this burden for patients and the healthcare service.

**References:**

1. Lone NI et al. Thorax 74:1046-1054, 2019

2.  McCallin AM. Nurs Crit Care 8:203-8, 2003

## P393 Frailty screening tools comparison in critically ill patients: a post-hoc analysis of a multicenter prospective cohort

### LU Taniguchi^1^, Q Ibrahim^2^, LC Azevedo^3^, H Stelfox^4^, S Bagshaw^5^

#### ^1^Hospital Sirio Libanes, Intensive Care Unit, Sao Paulo, Brazil; ^2^McMaster University, Department of Health Research Methods, Evidence, and Impact, Hamilton, Canada; ^3^Hospital Sirio Libanes, Instituto Sirio Libanes de Ensino e Pesquisa, Sao Paulo, Brazil; ^4^University of Calgary, Department of Critical Care Medicine, Cummings School of Medicine, Calgary, Canada; ^5^University of Alberta, Department of Critical Care Medicine, Faculty of Medicine and Dentistry, Alberta, Canada

**Introduction:**

Frailty has shown to have prognostic relevance for patients with critical illness. Since a wide range of tools has been described to screen for frailty, we aimed to describe the association of two frailty screening tools, the Clinical Frailty Scale (CFS) score and the modified Frailty Index (mFI) in critically ill patients.

**Methods:**

We performed a post-hoc analysis of a multicenter cohort of patients admitted to six Canadian Intensive Care Units (ICU) between February 2010 and July 2011. Frailty was identified using the Clinical Frailty Scale (CFS) and the modified Frailty Index (mFI). Concordance of the frailty screening tools was evaluated with partial Spearman rank correlation and intraclass correlation (ICC). Discrimination and predictive ability of the tools for hospital mortality, 1-year mortality, hospital readmission and adverse events were compared using concordance statistic (C-statistic) and calibration plot adjusting for age, sex, Sequential Organ Failure Assessment (SOFA) score and ICU admission source, respectively.

**Results:**

The cohort included 421 patients. Prevalence of frailty was 32.8% (95% confidence interval [CI] 28.3%–37.5%) with the CFS and 39.2% (95% CI 34.5%–44.0%) with the mFI. Concordance between the two tools was low [(ICC of 0.37; 95% CI 0.29-0.45) and partial correlation coefficient of 0.38 (95% CI 0.29-0.47)], even after adjustment. Hospital and 1-year mortality were greater for frail compared to non-frail patients using of both tools. Similarly, both tools found frail patients were less likely to be living independently after hospital discharge, and more likely to be rehospitalized when compared to non-frail patients.

**Conclusions:**

While the CFS and mFI show low concordance, both showed good discrimination and predictive validity for hospital mortality. Both tools identify a subgroup of patients more likely to have worse clinical outcomes.

## P394 Impact of a post-intensive care syndrome (PICS) prevention bundle in critically ill patients at ICU discharge and follow-up

### LA Costa hirai, CR Dos Santos, F De Souza Trindade Neto, VF Silva de Araújo, A Da Luz Leitão, W Sousa Montenegro, KT Passos Nishiwaki, M Martins Silva, JR Araújo de Azevedo

#### São Domingos Hospital, Intensive Care Unit, São Luís, Brazil

**Introduction:**

The Post-Intensive Care Syndrome (PICS) is a myriad of physical, psychiatric and cognitive disorders secondary to critical illness, leading to a decreased quality of life and an important socioeconomic burden. This study aimed to identify if the conformity to a PICS Prevention Bundle was able to reduce the incidence of the syndrome at ICU discharge.

**Methods:**

All patients admitted to the ICU from January 1^st^ to December 31^st^ 2018 were included. The conformity to each of the ten components of the PICS Prevention Bundle was assessed daily, and the patients were evaluated for anxiety, depression, cognitive dysfunction, muscular weakness, mobility impairment and nutritional risk at ICU discharge and at a 3-to-6-months follow-up consultation. The patient cohort was divided in terciles according to bundle conformity for the analysis.

**Results:**

From the 1145 enrolled patients, 352 (31%) were evaluated at ICU discharge, and 103 (29%) attended to the follow-up consultation. There was no difference in baseline characteristics between the cohorts. There was no correlation between the prevalence of PICS at discharge and Bundle conformity during ICU stay (90% vs. 87% vs 87%, *p* 0.834), though there was a decrease in nutritional risk and days in mechanical ventilation (Table 1). After 3 to 6 months there was a reduction on the prevalence of any kind of PICS, mobility impairment, muscular weakness and nutritional risk. The patients that developed PICS were older and had a higher Simplified Acute Physiology Score III at ICU admission.

**Conclusions:**

A higher adhesion to a PICS Prevention Bundle was not able to prevent the occurrence of the syndrome.

**Table 1 (abstract P394).** Differences in Post-Intensive Care Syndrome (PICS) prevalence according to terciles of conformity to the PICS Prevention Bundle

	Low (n=122)	Intermediate (n=113)	High (n=117)	p-value
PICS, % ± SD	90 ± 73	87 ± 76	87 ± 74	0.834
Anxiety, % ± SD	31 ± 26	25 ± 23	34 ± 29	0.578
Depression, % ± SD	20 ± 17	21 ± 19	19 ± 16	0.816
Cognitive dysfunction, % ± SD	64 ± 54	58 ± 54	64 ± 57	0.837
Mobility, % ± SD	48 ± 40	44 ± 40	40 ± 35	0.641
Strength, % ± SD	9 ± 7	7 ± 6	4 ± 3	0.408
Nutrition, % ± SD	21 ± 17	8 ± 7	7 ± 5	0.007

## P395 Does sleep deprivation continue beyond intensive care in critical illness survivors: findings from a larger phenomenological study

### AC Tembo

#### University of Sydney, Faculty of Health and Medicine, Susan Wakil Nursing School, Sydney, Australia

**Introduction:**

Despite improved outcomes of intensive care unit (ICU) patients, sleep deprivation remains a major concern after ICU discharge. Multifaceted causes make it difficult to treat and understand [1]. Not many studies have explored sleep deprivation beyond ICU. This is evidenced by findings from a recent systematic review [2] which included 8 studies with only one study [3] reporting sleep deprivation beyond ICU. The aim of this paper is to present findings of sleep deprivation beyond ICU from a larger study that examined the experience of critical illness in ICU and beyond in the context of daily sedation interruption.

**Methods:**

Hermeneutic phenomenology was used to conduct the study. 12 participants aged 18 years and above who fulfilled the enrolment criteria were enrolled into the study. The cohort comprised 7 male and 5 female participants. In-depth face to face interviews at two weeks after discharge were conducted and repeated at six to eleven months. Interviews were audio taped, transcribed and thematically analysed. Significant statements were highlighted and categorized for emergent themes.

**Results:**

Six participants continued to experience sleep deprivation up to eleven months after ICU. Two cited dreams about ICU, three could not explain why they continued to fail to sleep and one stated that he continued hearing ICU alarms in the silence of the night.

**Conclusions:**

Sleep deprivation continues beyond ICU due to nightmares, delusional memories and unexplained reasons. Further research is needed to establish causes of sleep deprivation and explore ways to promote sleep in critical illness survivors after ICU discharge.

**References:**

1**.** Mannion H et al. Int J Environ Res Public Health 16:3577, 2019

2. Medrzycka-Dabrowska W et al. Open Medicine 13:384–393, 2018

3. Tembo et al. Pub Med 29:310–316, 2013

## P396 Post intensive care syndrome after cardiothoracic critical care: 1 year outcomes and the effect of a complex multi-professional intervention

### P Henderson^1^, I Quasim^2^, L Davey^3^, M Shaw^4^, T Quasim^5^, J McPeake^5^

#### ^1^University of Glasgow, Department of Anaesthesia and Intensive Care medicine, Glasgow, United Kingdom; ^2^Golden Jubilee National Hospital, Cardiothoracic Anaesthesia and Critical Care, Glasgow, United Kingdom; ^3^Golden Jubilee National Hospital, Cardiothoracic Critical Care, Glasgow, United Kingdom; ^4^NHS Greater Glasgow & Clyde, Department of Clinical Physics and Bioengineering, Glasgow, United Kingdom; ^5^University of Glasgow, Department of Anaesthesia and Intensive Care Medicine, Glasgow, United Kingdom

**Introduction:**

Post intensive care syndrome (PICS) is well recognized following general ICU care [1]. Intensive Care Syndrome:Promoting Independence and Return to Employment (InS:PIRE) is a multidisciplinary complex intervention designed to address PICS [2]. With a paucity of evidence on PICS after cardiothoracic intensive care, we aim to evaluate PICS and the feasibility of the InS:PIRE intervention in this population.

**Methods:**

Those attending the clinic received 5 weeks of intervention including individual appointments with ICM nurse, physician, pharmacist, and physiotherapist. A café area facilitated peer support alongside psychology group sessions. Primary outcome was quality of life measured by EQ-5D-5L. Further surveys included: pain, mental health, and self-efficacy. Questionnaires were taken at baseline, 3 and 12 months.

**Results:**

Over 5 cohorts, 27 patients attended, 67% male, median age 66 years (IQR 61–75), median APACHE 2 score of 17 (IQR 14-18.5), and median ICU length of stay was 13 days (IQR 9-21). A total of 14 (53%) patients completed surveys at one year. Scheduled admissions represented 56% of those attending. Mean EuroQol EQ-VAS score was 70/100 (SD +/- 18) at baseline increasing to 78/100 (SD +/- 16) by 1 year (Table 1). Those with problems in at least one domain of EQ-5D-5L fell from 92% at baseline to 73% at 1-year with the breakdown shown in table 1. Severe problems were seen in 22% falling to 18% at 1 year. HADS demonstrated an anxiety or depression rate of 44%. Brief pain inventory identified 14 patients (52%) with ongoing chronic pain. Mean self-efficacy was 32/40 (SD +/-6) at baseline and 34/40 (SD +/-5) at 1 year.

**Conclusions:**

Cardiothoracic intensive care patients have ongoing and persistent features of PICS with significant effects on health-related quality of life. Further, the InS:PIRE multi-professional complex intervention is feasible within this specialist group.

**References:**

1. Needham DM et al. Crit Care Med 40:502-9, 2012

2. McPeake J et al. PloS one. 12:e0188028, 2017

**Table 1 (abstract P396).** Patients expressing problems within each domain of EQ-5D-5L and overall quality of life using EQ-VAS. Measured at 1st clinic visit (baseline), 3 months and 12 months

EQ-5D-5L Domain	Clinic baseline	3 months	12 months
Problems with mobility (%)	56	50	45
Problems with self-care (%)	40	33	36
Problems with usual activities (%)	80	61	55
Pain / discomfort (%)	76	72	64
Anxiety / depression (%)	64	39	45
Mean EQ-VAS score (0-100)	70	78	78

## P397 Frailty: a predictor of mortality amongst critical care survivors independent of age

### A Munro, P Hayden, R Sarkar

#### Medway Maritime Hospital, Intensive Care Unit, Gillingham, United Kingdom

**Introduction:**

Frailty is being increasingly seen as an independent syndrome. Frail patients now account for an increasing proportion of hospital and critical care admissions [1]. We aimed to compare frailty and mortality in our intensive care unit.

**Methods:**

Clinical Frailty Score (CFS) was incorporated within the electronic health record (EHR) 2019. We performed this retrospective analysis on the data collected between Jan’19 and Oct’19. The predictor and outcome for this study were frailty and hospital mortality respectively. All demographic data, acute physiology score, critical care and hospital outcome data were automatically collected in the EHR and recorded. We used a cut off of CFS>3 and above to define non-frail and frail respectively. Chi-squared test, simple and multiple logistic regression were used. Adjustment was done for ICNARC score and age.

**Results:**

Total number of patients was 848, of which 140 (16.5%) died in hospital. Within the patients<65 years (n=392), 79 (20%) were recorded as frail or vulnerable. The number of elective and emergency admission were 292(34%) and 556(66%) respectively. In the frail and non-frail, mortality rates were 30% and 9.5% (p<0.001) respectively, with odds ratio of 4.14 (95% CI 2.8, 6; p<0.001) (Table 1). For emergency patients, mortality in the frail and non-frail were 36.3% and 16.5% respectively (p<0.001), with odds ratio 2.9 (95% CI 1.9, 4.3; p<0.001) but after adjustment, it was not significant [OR 1.64 (95% CI 0.97, 1.77); p=0.06]. For patients, who were discharged from critical care, mortality in the frail and non-frail were 11.8% and 1.2% respectively (p<0.001) with odds ratio 11.8 (95% CI 4.6, 27.9; p<0.001) and adjusted OR 4.19 (95% CI 4.16, 11.1; p=0.06).

**Conclusions:**

Frailty is a risk factor for hospital mortality for critical care survivors, independent of age. It is possible that enhanced targeted resource allocation and appropriate placement for these patients could alter adverse outcome.

**References:**

1) Flatten H et al. Intensive Care Med 43:1820-1828, 2017

**Table 1 (abstract P397).** Mortality rate in frail versus non frail patient population with unadjusted and adjusted odds ratio (OR)

	Mortality rate in frail	Mortality rate in non-frail	Unadjusted OR	Adjusted OR
All patients	30.2%	9.5%	4.14 (2.84, 6.05) P<0.001	2.25 (1.36,3.71) P<0.001
Emergency patients	36.3%	16.5%	2.9 (1.95,4.31) P<0.001	1.64 (0.97, 2.77) P=0.06
Critical care survivors	11.8%	1.2%	11.4 (4.6, 27.9) P<0.001	4.19 (1.6, 11.1) P=0.004

## P398 Age versus clinical frailty score for prognostication in emergency critical care admissions

### C Donnelly, P Hayden, R Sarkar, A Bowman

#### Medway Maritime Hospital NHS Foundation Trust, Critical Care, Gillingham, United Kingdom

**Introduction:**

Age is a well-known risk factor for Critical Care (CC) outcome and is incorporated into many prognostic tools; however, this has been criticized for assumption of normal physiology for young at baseline. In recent years, frailty in CC prognostication has been of interest, with meta-analysis correlating worsening outcomes with increasing frailty [1].  In this study, we compared the effect of frailty versus age for determining hospital survival for critically ill patients.

**Methods:**

This study was conducted in a UK District General Hospital, with 19 CC beds, where the Rockwood Clinical Frailty Score (CFS) is collected on all adult admissions. Retrospective analysis of data, adjusted for ICNARC score was performed on CC emergency admissions using STATA 14, combining CFS, patient demographics and outcome data from our electronic records. Logistic regression was used for mortality prediction. We defined non-frail as CFS<4. An age cut-off of 70 years was used for the age adjusted model, as remaining life expectancy (RLE) at age 70 years has recently been shown to be equal to RLE at age 65 years before [2].

**Results:**

Total number of patients was 556 (male- 58%) of which 140 (16.5%) died in hospital. Importantly, in the patients <70 years (n=529), 137(48%) had CFS<4. The number of elective and emergency admission were 292(34%) and 556(66%) respectively. The risk for mortality (OR) in the adjusted model with age and CFS cut off were 2.65 (1.61, 4.35) and 2.35 (1.44, 2.83) respectively. Both of these mortality prediction models, one incorporating age cut-off 70 and the other based on CFS, show equal performance of discrimination (AUROC 0.88) (Figure 1).

**Conclusions:**

Our data supports an association between increasing CFS and mortality for the CC population independent of age.  Frailty should be incorporated in clinical decision making in addition to utilisation of arbitrary cut offs of age.

**References:**

1. Muscedere J et al. Intensive Care Med 43:1105-1122, 2017

2. Office for National Statistics

**Fig. 1 (abstract P398).**
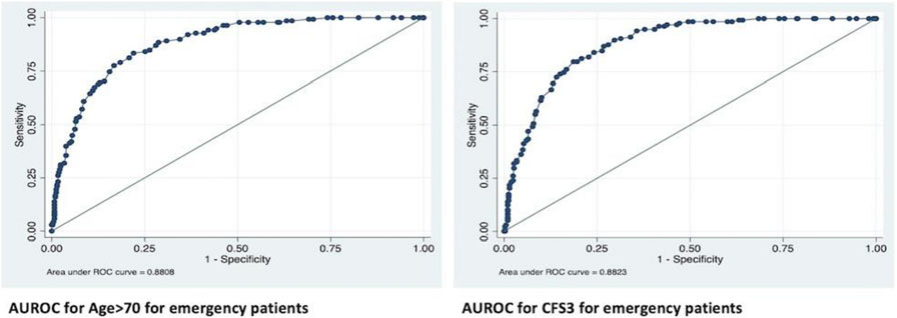
Age vs CFS emergency critical care admissions

## P399 The impact of chronic critical illness on the development of post ICU syndrome: a Brazilian cohort study

### P Berto^1^, S Glaeser^2^, LF Martins^2^, J Haas^3^, D Sganzerla^2^, R Rosa^2^, T Lisboa^1^, C Teixeira^1^, G Friedman^4^

#### ^1^Universidade Federal do Rio Grande do Sul, Porto Alegre, Brazil; ^2^Hospital Moinhos de Vento, Porto Alegre, Brazil; ^3^Hospital de Cínicas de Porto Alegre, Porto Alegre, Brazil; ^4^Universidade Federal do Rio Grande do Sul, PPG Ciências Pneumológicas, Porto Alegre, Brazil

**Introduction:**

ICU survivors are discharged from hospital with varying degrees of frailty. The impact of ICU length of stay on the development of post-ICU Syndrome symptoms is still controversial. Through a follow-up study after hospital discharge [1], we assessed the impact of critical care on the psychological symptoms, functional outcomes and quality of life of patients

**Methods:**

We conducted a prospective cohort in 16 Brazilian hospitals including 1616 survivors of an ICU stay > 72h. We compared chronic critically ill patients (ICU stay> 10 days) and the other patients. We performed psychological and functional presential assessment in patients within 48 hours of ICU discharge and by telephone at 3 and 6 months.

**Results:**

The prevalence of chronic critically ill patients was 26%. Regarding outcomes, chronic critically ill patients had a higher incidence of depressive symptoms than other patients in the immediate post-ICU discharge (p = 0.004), as well as a higher incidence of muscle weakness (p <0.001). However, in subsequent evaluations, we found no difference between groups regarding psychological symptoms - depression, anxiety and post-traumatic stress. Higher functional dependence was observed in critically ill patients, but without difference in the quality of life score, both in the physical (p = 0.87) and mental (p = 0.84) domains.

**Conclusions:**

Chronic critically ill patients, when compared to patients with stay> 72h, have a higher incidence of depressive symptoms at ICU discharge. This difference disappears in the follow up. Chronic critically ill patients present higher levels of functional dependence but without repercussions on quality of life scores.

**References:**

1. Robinson CC et al. Rev Bras Ter Intensiva 30:405, 2018

## P400 Association of white blood cell count with one-year mortality after cardiac arrest

### H Vuopio^1^, MB Skrifvars^1^, R Raj^2^, S Bendel^3^, M Reinikainen^4^, PT Pekkarinen^5^

#### ^1^Helsinki University Hospital, Department of Emergency Care Services, Helsinki, Finland; ^2^Helsinki University Hospital, Department of Neurosurgery, Helsinki, Finland; ^3^Kuopio University Hospital, Division of Intensive Care Medicine, Kuopio, Finland; ^4^Kuopio University Hospital, University of Eastern Finland, Kuopio, Finland; ^5^Helsinki University Hospital, Division of Intensive Care Medicine, Department of Anaesthesiology, Helsinki, Finland

**Introduction:**

Activation of the inflammatory response after cardiac arrest (CA) is a well-documented phenomenon that may lead to multi-organ failure and death. We hypothesized that white blood cell count (WBC), one marker of inflammation, is associated with one-year mortality in ICU treated CA patients.

**Methods:**

We used a nationwide registry with data from five academic ICUs to identify adult CA patients treated between January 1^st^ 2003 and December 31^st^ 2013. We evaluated the association between the most abnormal WBC within 24 hours of hospital admission and one-year mortality. We accounted for baseline risk of death using multivariable logistic regression (adjusted for age, gender and 24h sequential organ failure assessment [SOFA] score).

**Results:**

A total of 5,543 patients were included in the analysis. Of those patients 2,387 (43%) were alive one year after CA. We plotted WBC against baseline risk of death and through graphic examination of a locally weighted scatterplot smoothing (lowess) curve found the lowest risk of death to be associated with a WBC of 12 (E^9^/l) (Figure 1). We used this value as a cut-off point and ran separate multivariable regression analyses, accounting for baseline risk, for patients with a WBC over or under 12. For those with a WBC under 12, an increasing WBC was associated with a reduced risk of death (OR 0.969, 95% confidence interval [CI] 0.940–0.999). For those with a WBC over 12, an increasing WBC was associated with an increased risk of death (OR 1.018, 95% CI 1.004–1.034).

**Conclusions:**

A WBC deviating from 12 E^9^/l is associated with a small but statistically significant increase in probability of death in CA patients. Mortality increases with both high and low WBCs. The lowest mortality was observed at WBC 12 which is higher than healthy population reference value (WBC 3.4 -8.2), suggesting that moderate leukocytosis after CA may not be harmful.

**Fig. 1 (abstract P400).**
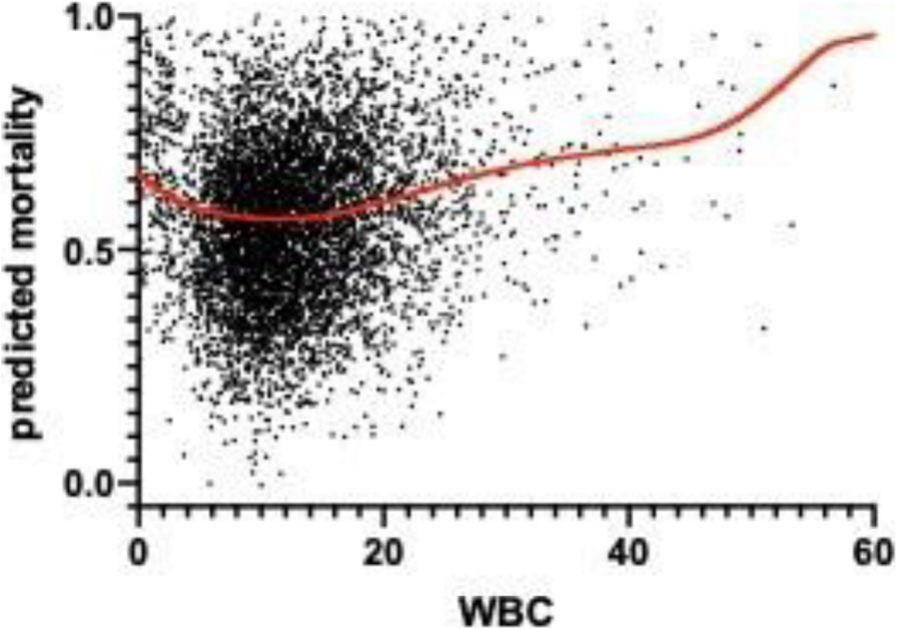
Association of white blood cell count with predicted mortality

## P401 Risk factors for dysphagia in ICU patients following invasive mechanical ventilation

### P Zuercher, NV Schenk, C Moret, D Berger, R Abegglen, JC Schefold

#### University Hospital Bern, Department of Intensive Care Medicine, Bern, Switzerland

**Introduction:**

Dysphagia is common and independently predicts death in ICU patients. Risk factors for dysphagia are largely unknown with sparse data available only from mostly small cohorts without systematic dysphagia screening. We investigated risk factors for dysphagia in ICU patients post invasive mechanical ventilation.

**Methods:**

Post-hoc analysis of data from a large monocentric prospective observational study (“Dysphagia in mechanically ventilated ICU patients, DYnAMICS” [1]) using comprehensive statistical models to identify potential risk factors for dysphagia in 933 primary ICU admissions. In DYnAMICS, patients were systematically screened for dysphagia at the bedside within 3 hours from extubation using the two-step Bernese ICU Dysphagia Algorithm. Screening-positivity was followed within 24 hours by a confirmatory exam, identifying 116 ICU patients suffering from dysphagia.

**Results:**

After adjustment for typical confounders, baseline neurological disease (OR 4.45, 95%-CI: 2.74-7.24, p<0.01), emergency admission (OR 2.04, 95%-CI: 1.15-3.59, p<0.01), days on mechanical ventilation (OR 1.19, 95%-CI: 1.06-1.34, p<0.01), days on renal replacement therapy (OR 1.1, 95%-CI: 1-1.23, p=0.03), and disease severity (APACHE II score within first 24 hours; OR 1.03, 95%-CI: 0.99-1.07, p<0.01) remained independent risk factors for dysphagia post mechanical ventilation (Figure 1). Increased body mass index reduced the risk for dysphagia (6% per step increase, OR 0.94, 95%-CI: 0.9-0.99, p=0.03).

**Conclusions:**

In ICU patients, baseline neurological disease, emergency admission and duration of invasive mechanical ventilation appeared as prominent independent risk factors for dysphagia. Although screening for dysphagia should be performed in all ICU patients, a risk-based screening approach might be implemented whenever screening of the total ICU population is not deemed feasible.

**References:**

1. Schefold JC et al. Crit Care Med 45:2061-2069, 2017

**Fig. 1 (abstract P401).**
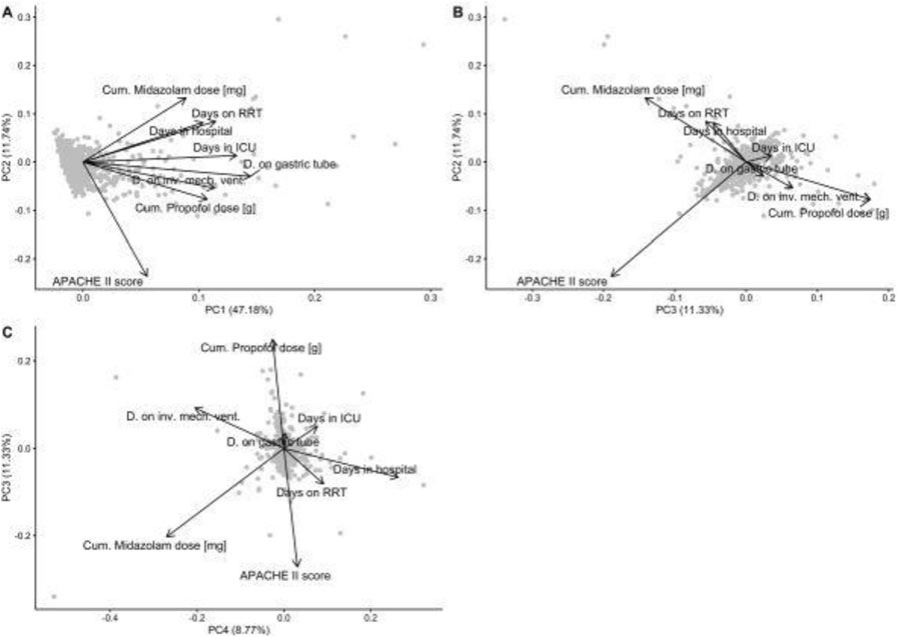
PCA of the correlated cluster of variables days on RRT, days in hospital, cumulative midazolam dose, days on ICU, days on gastric tube, days on invasive mechanical ventilation, cumulative midazolam dose and APACHE II score. PCs 1 to 4 (A-C) are shown on horizontal and vertical axes. Based on PC 1 to 3 (explaining 70.25% of the variance), three groups of swappable variables were identified: (1) days on RRT, days in hospital and cumulative midazolam dose, (2) days in ICU, days on gastric tube, days on invasive mechanical ventilation and cumulative propofol dose and (3) APACHE II score. The first member of each group was selected as representative variable

## P402 Optimizing Subsequent CARdiovascular Medication Re-introduction in the Intensive Care Unit: a retrospective study (OSCAR)

### ME Boisjoly^1^, C Lavigne^1^, L Burry^2^, N Tabbara^2^, HT Wang^3^

#### ^1^Maisonneuve-Rosemont hospital, Department of medicine, division of internal medicine, Montreal, Canada; ^2^Sinai Health and University of Toronto, Leslie Dan Faculty of Pharmacy, Toronto, Canada; ^3^Maisonneuve-Rosemont Research Centre, Department of medicine, division of internal and critical care medicine, Montreal, Canada

**Introduction:**

Admission and transitioning between services creates break points in communication and can lead to unintentional medication discrepancies during hospital stay [1]. Cardiovascular medications may be discontinued on ICU admission. Whether they are appropriately re-initiated after the acute illness and on hospital discharge is unclear.

**Methods:**

We performed a retrospective chart review study in a tertiary ICU from April 2016 to April 2017. Our medications of interest were ARBs and ACEIs. We collected baseline characteristics, APACHE II and SOFA scores on admission and whether patients were on chronic cardiovascular medications before hospitalization. Our outcome of interest was the re-initiation of the same class of cardiovascular medication at hospital discharge.

**Results:**

Data from the first 199 ICU survivors were analyzed. Median age was 68 (interquatile range (IQR)=18) years and 42.2% were female. The APACHE II and SOFA scores on admission were 14 (IQR=11) and 4 (IQR=4) respectively. 99 (49.8%) of patients were mechanically ventilated and 77 (38.7%) needed vasopressor support during their ICU stay. There was a total of 80 patients on either ACEIs or ARBs before hospitalization. Among them, 40 patients (50%) was re-prescribed an ACEIs or ARBs at discharge and none of them stayed on renal replacement therapy at discharge. Univariate logistic regression showed that SOFA score (odds ratio (OR)=1.20 [1.004-1.425], p=0.045) and the need of vasopressor support (OR=3.66 [1.419-9.467], p=0.0073) were associated with ACEIs and ARBS not being re-initiated (Table 1).

**Conclusions:**

A significant proportion of ACEs and ARBs are not re-initiated at discharge. Future studies need to address this discrepancy.

**References:**

1. Bonaudo M et al. PLoS One 13:e0191028, 2018

**Table 1 (abstract P402).** Results. CKD1 : Chronic kidney disease stage 3 and more, CAD : Coronary arterial disease, CI : Confidence limits

Variable	Represcribed N=40	Not represcribed N=40	Odds ratio (95 % CI)	p-value
Age, median (IQR)	72 (12.5)	75 (12.5)	1.04 (0.99-1.10)	0.10
CKD, N (%)	7 (17.5)	13 (32.5)	2.15 (0.78-5.92)	0.14
CAD, N (%)	10 (25.0)	17 (42.5)	2.22 (0.86-5.74)	0.10
SOFA score, median (IQR)	3 (3.0)	5 (3.0)	1.20 (1.00-1.43)	0.045
APACHE II score, median (IQR)	10 (13.0)	16.5 (11.0)	1.04 (0.98-1.11)	0.17
Vasopressor, N (%)	10 (25.0)	22 (55.0)	3.67 (1.42-9.47)	0.007

## P403 Predicting medication related problems in a post-ICU population using demographic and in-ICU clinical data

### C Purdie^1^, P MacTavish^2^, T Quasim^1^, P Henderson^1^, J McPeake^2^, M Shaw^3^

#### ^1^University of Glasgow, School of Medicine, Glasgow, United Kingdom; ^2^Glasgow Royal Infirmary, Intensive Care Unit, Glasgow, United Kingdom; ^3^Glasgow Royal Infirmary, Clinical Physics Department, Glasgow, United Kingdom

**Introduction:**

During critical illness, chronic therapies are often withheld and subsequently not restarted, and ICU-specific treatments can be inappropriately continued once discharged. Critically ill patients also experience many transitions of care, which may also cause issues with on-going medicine management. Additionally, patients are often prescribed new medications, which they may not understand how to take effectively, or which may cause adverse events. We aimed to determine if we could predict Medication-Related Problems (MRPs), using demographic and in-ICU clinical data.

**Methods:**

183 patients enrolled in a post-intensive care programme between September 2016 and June 2018. Intensive Care Syndrome: Promoting Independence and Return to Employment (InS:PIRE), is a 5-week multicentre, multidisciplinary rehabilitation programme for ICU survivors. MRPs were identified by a specialist ICU pharmacist during this programme and classified by their significance on a scale of one to four. Logistic regression was used to determine if demographic factors were associated with the occurrence of a clinically significant MRP - a significance score of two or above (Figure 1).

**Results:**

The adjusted model included age, ICU LOS, hospital LOS, APACHE II, number of days of renal replacement therapy, number of days of ventilation, the number of medications prescribed at ICU discharge, and the WHO analgesia classification at InS:PIRE. There were increased odds of having a clinically significant MRP for hospital LOS (OR 1.03 per day, 95% CI: 1.01-1.05, p=0.02), number of medications at ICU discharge (OR 1.15 per medication, 95% CI: 1.04-1.28, p=0.01), and WHO Step 2 analgesia at the InS:PIRE clinic (OR 5.20 vs WHO level 0, 95% CI: 2.07-13.20, p=0.001).

**Conclusions:**

Length of days in hospital, the number of medications at ICU discharge, and WHO step 2 analgesia prescription are all associated with increased odds of developing an MRP. Patients with long stays and complex polypharmacy should be reviewed in the post-ICU recovery period.

**Fig. 1 (abstract P403).**
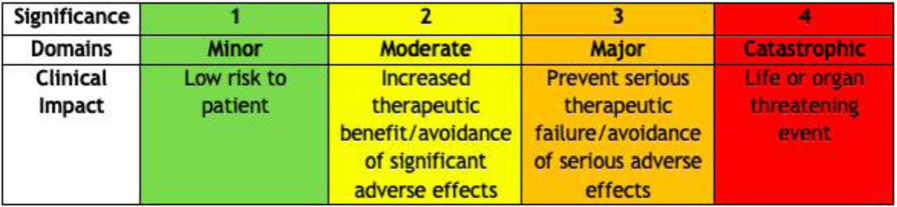
Significance scale of MRPs

## P404 Categorising medication related problems in a post-ICU population

### C Purdie^1^, P Henderson^1^, T Quasim^1^, J McPeake^2^, M Shaw^3^, P MacTavish^2^

#### ^1^University of Glasgow, School of Medicine, Glasgow, United Kingdom; ^2^Glasgow Royal Infirmary, Intensive Care Unit, Glasgow, United Kingdom; ^3^Glasgow Royal Infirmary, Clinical Physics Department, Glasgow, United Kingdom

**Introduction:**

During critical illness, chronic therapies are often withheld and subsequently not restarted, and ICU-specific treatments can be inappropriately continued once discharged. Critically ill patients are also experience higher numbers of transitions of care, which may also cause issues with on-going medicine management. Additionally, patients are often prescribed new medications which they may not understand how to take effectively, or which may cause adverse events. We aimed to categorise the nature of the Medication Related Problems (MRPs) found in this population.

**Methods:**

183 patients enrolled in a post-intensive care programme between September 2016 and June 2018. Intensive Care Syndrome: Promoting Independence and Return to Employment (InS:PIRE), is a 5-week multicentre, multidisciplinary rehabilitation programme for ICU survivors and their caregivers. MRPs were identified by a specialist ICU pharmacist during this programme. This MRP was then categorized by type and by British National Formulary (BNF) category.

**Results:**

62·8% (n=115) of patients required at least one pharmacy intervention. The median number of interventions required per patient was one (IQR 0-2); the maximum number was six. 198 MRPs were recorded in this cohort.  The most common intervention was clarifying duration of treatment (n=44), followed by education (n=33), and correcting drug omissions (n=27). The BNF drug class most frequently associated with MRPs was neurological (n=65), which comprises analgesics (n=45) and psychiatric medications (n=20) (Figure 1). This was followed by cardiovascular medications (n=40), gastrointestinal medications (n=34), nutritional medications (n=25), and others (n=34).

**Conclusions:**

Many ICU survivors experience MRPs. The most common class of MRP was neurological, reflecting the high incidence of chronic pain and psychiatric illness in this population

**Fig. 1 (abstract P404).**
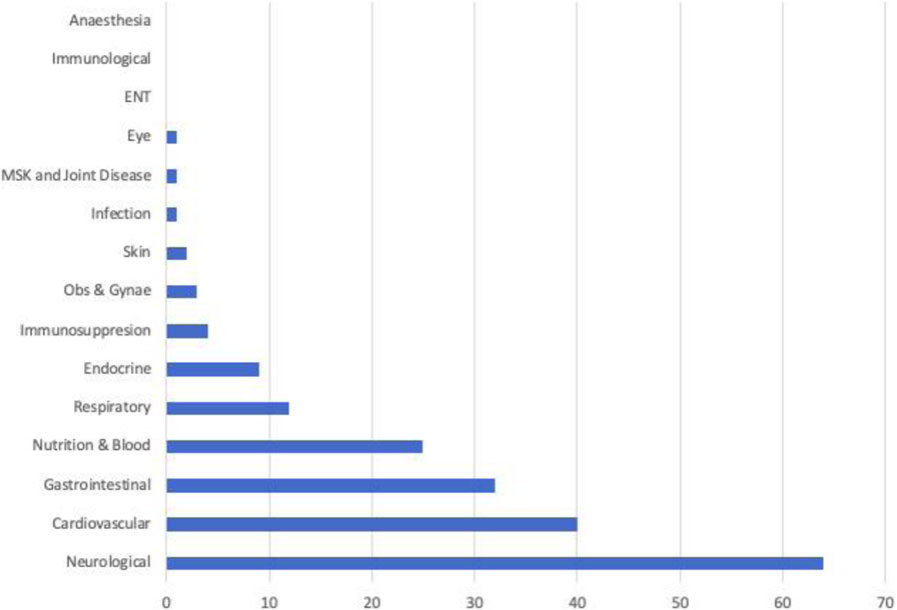
BNF category of MRPs

## P405 Implementation of physiotherapy early after cardiac surgery: the Onassis cardiac surgery center experience

### V Raidou^1^, S Dimopoulos^2^, F Chatzivasiloglou^1^, K Sakki^1^, A Robola^2^, K Papadopoulos^2^, A Tasouli^2^, I Kriaras^2^, S Nanas^1^, A Karabinis^2^

#### ^1^National and Kapodestrian University of Athens, Clinical Ergospirometry, Exercise and Rehabilitation Laboratory, Athens, Greece; ^2^Onassis Cardiac Surgery Center, Cardiac Surgery ICU, Athens, Greece

**Introduction:**

Early mobilization of Intensive Care Unit (ICU) patients is effective in accelerating ICU discharge. However data on mobilization practices on ICU patients are inadequate. The aim of this study was to document physiotherapy intervention practices on ICU Cardiac Surgery patients at the Onassis Cardiac Surgery Center.

**Methods:**

For this study, of the 221 patients that were subjected to cardiothoracic surgery, 159 were assessed for the effects of early mobilization on the duration of ICU hospitalization. Physiotherapy intervention was recorded on the 1st, 3rd, 5th and 7th day of ICU admission.

**Results:**

Physiotherapy practice consisted of respiratory physiotherapy and mobilization interventions such as active and passive limb mobilization, sitting in bed and transferring from bed to chair. The proportion of patients mobilized, was 18% (Ν=29/159) within 24 hours from ICU admission, 53% (Ν=46/87) in day 3, 54% (Ν=22/41) in day 5 and 62% (N= 15/24) in day 7. The duration of stay in the ICU was reduced in patients who were mobilized compared to bedridden, (28 ± 37 vs 66 ± 92 hours, p = 0.001).

**Conclusions:**

Early mobilization rates of patients undergoing cardiothoracic surgery appear reduced in the ICU Cardiac Surgery compared to international literature. Physiotherapy interventions practices are associated with decreased ICU length of stay.

## P406 Ventilator associated pneumonia (VAP) in tracheoventilated home patients

### F Righetti^1^, E Colombaroli^2^

#### ^1^Intensive Care Unit, Fracastoro Hospital, Emergency Department, San Bonifacio, Verona, Italy; ^2^Intensive Care Unit, Fracastoro Hospital, San Bonifacio, Verona, Italy

**Introduction:**

The purpose of our retrospective study is to compare 2 groups (patients with amyotrophic lateral sclerosis - ALS-  or neurological disease and patients non neurological disease) of tracheoventilated patients in a considered period to highlight differences in ICU recovery for VAP [1] and mortality, due to VAP or other causes - ex. acute renal failure or cardiac diseases.

**Methods:**

24 tracheostomized adult patients, cuffed cannulae, 24 hours of home ventilation, cannula change every 60 days at home, in the period 2015-2019, classified in 2 groups (group A: patients with ALS or neurological diseases - 62.5% - and group B: patients without neurological diseases ex. pulmonary diseases - 37.5%). We consider VAP with ICU recovery and mortality due to VAP or other causes in the 2 groups. Clinical, radiological and microbiological criteria have been used for diagnosis of VAP. The results is expressed as percentage and as average and standard deviation.

**Results:**

VAP with ICU recovery involved 50% (12/24) of the patients [average 1.83 and standard deviation 0.79] (66% group A vs 22% group B). The deaths for VAP 4/12 (33% - 26% group A vs 0% group B). Mortality for other causes 5/24 (20.5% - 13% group A vs 33% group B).

**Conclusions:**

Comparing the 2 groups, VAP with ICU recovery and mortality for VAP in ICU are more frequent in group of neurological patients; mortality for other causes is higher in the group of non neurological patients.

**References:**

1. Timsit JF. F1000Res 6:2061, 2017

## P407 Prognostic factors of heterotopic ossification in critically ill patients

### AG Vassiliou^1^, I Dimopoulou^2^, E Jahaj^2^, Z Mastora^2^, SE Orfanos^2^, A Kotanidou^2^

#### ^1^National & Kapodistrian University of Athens, 1st Department of Critical Care Medicine & Pulmonary Services, GP Livanos and M Simou Laboratories, Athens, Greece; ^2^National & Kapodistrian University of Athens, 1st Department of Critical Care Medicine & Pulmonary Services, Athens, Greece

**Introduction:**

Heterotopic ossification (HO) is the formation of bone in soft tissues and constitutes a potential complication of patients hospitalized in the ICU. The exact pathogenetic mechanisms of HO are unknown. Bone morphogenic proteins (BMPs) induce bone formation, while signaling through the receptor activator of NF-κB (RANK) and its ligand (RANKL) regulates osteoclast formation, activation and survival in normal bone modeling and remodeling. Osteoprotegerin (OPG) protects bone from excessive bone loss by preventing RANKL from binding to RANK. In the present study we investigated these molecules as possible biomarkers of HO development in ICU patients.

**Methods:**

We measured the levels of BMP-2, RANKL and OPG on ICU admission, and thereafter on days 7 and 30 in the sera of 28 critically ill patients using ELISA.

**Results:**

Nine of the 28 patients developed HO in the ICU or during the 30-day follow-up period. Our results showed that on admission to the ICU, the patients who developed HO had significantly lower BMP-2 levels compared to patients who didn’t [566.7 (216.7-883.3) pg/ml vs. 1300 (566.7-2817) pg/ml, respectively, p= 0.037]. RANKL levels were also decreased in the HO patients on admission [1.495 (0.285-2.465) ng/ml vs. 2.465 (1.535-6.67) ng/ml, p= 0.048], while OPG levels were similar in both groups. In the HO patients, on day 7, the levels of BMP-2 were slightly elevated, whereas on day 30 they tended to increase (p= 0.064). The patients who did not develop HO had stably elevated levels during the 30 days. A small elevation was noted in RANKL levels in the HO patients, while OPG levels remained unaltered at all time points, for both groups.

**Conclusions:**

In our cohort of critically ill patients, those who will develop heterotropic ossification have significantly decreased levels of BMP-2 and RANKL on admission, suggesting that these molecules may be used as prognostic markers to identify patients who will develop this debilitating condition.

## P408 Improving the transition of care from the intensive care unit to wards

### C Cunningham, M McKenna, C Patterson, M Doohan, M Duffy

#### Mater Infirmorium Hospital, Belfast, United Kingdom

**Introduction:**

The transfer of patients from the intensive care unit (ICU) to the general wards is a challenging transition of care. This project focused on the handover of patients between ICU and the general wards in a district general hospital where there was no formal handover system in place. A baseline survey found 100% of foundation year 1 (FY1) doctors felt medications were unclear when rewriting ICU drug charts from the electronic notes. Junior doctors were often unaware patients had been transferred. This resulted in significant delays in drug charts being rewritten and missed or delayed medications. Our aim was to reduce the median time delay in drug charts being rewritten by 50% in 4 months.

**Methods:**

Following discussion with ICU staff, ward staff and FY1 doctors, a formal standardized handover system was introduced. This involved a verbal handover to the appropriate FY1 by an ICU doctor and the patient drug chart to be rewritten in ICU at the time of handover. The next change was to display posters on the wards to alert staff that the medical team are to be contacted when a patient comes to the ward from ICU and to ensure the drug chart is completed.

**Results:**

The baseline data showed a median time delay of 4 hours, with one patient waiting 14 hours for a drug chart. Following the interventions the median time delay has decreased to 0 hours within 4 months as demonstrated in Figure 1. The changes have received positive feedback from ICU staff, ward staff and FY1 doctors.

**Conclusions:**

The aim of reducing the time delay by 50% has been achieved with the median time delay now 0 hours. This has improved patient safety by significantly reduced delays in medications and through the introduction of a standardized handover. This has also provided an opportunity for junior doctors on the wards to seek clarification regarding medications and the clinical management plan for the patient. This has established a communication channel between ICU and the wards making patient care safer and more effective.

**Fig. 1 (abstract P408).**
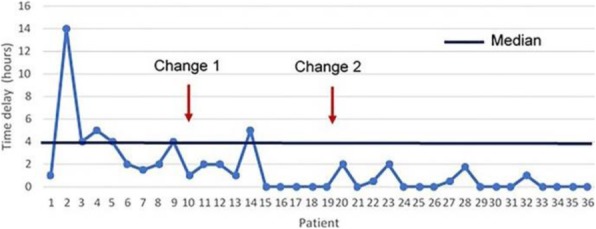
Run chart showing time delay in rewriting ICU drug chart in patients being discharged from ICU

## P409 E-health outside the ICU: a wearable in telemonitoring respiratory and heart rates as part of the Early Warning Score

### EJ Van lieshout^1^, J Hoekstra^2^, R Voorn^3^, E Maij^4^, M Regeling^4^, I Bruining^5^, R Lo^5^

#### ^1^Amsterdam University Medical Center, Intensive Care and Mobile Intensive Care Unit, Amsterdam, Netherlands; ^2^Amsterdam University Medical Center, dept. of Hematology, Amsterdam, Netherlands; ^3^Amsterdam University Medical Center, Dept. of medical engineering, Amsterdam, Netherlands; ^4^Amsterdam University Medical Center, Dept. of EPIC Services, Amsterdam, Netherlands; ^5^Amsterdam University Medical Center, Dept. of ICT, Amsterdam, Netherlands

**Introduction:**

Telemonitoring outside the ICU is scarce. But with innovative wearables measuring respiratory and heart rate wirelessly, culture on intrahospital telemonitoring should definitely change. However, culture has been known to be one of the most crucial success factors in innovation, especially in health care. Human design thinking is a promising tool in health care innovation but rarely used in a multidisciplinary team to initiate an innovation culture and stimulate sustainable collaboration. The aim of this study was to initiate a pilot project with a multidisciplinary team to start using wearables for Early Warning Score (EWS) on a clinical ward.

**Methods:**

Human Design Thinking was used to write a value proposition on wearables in clinically admitted neutropenic hematologic patients in an academic center. A multidisciplinary team was performed to cover all disciplines involved in the technical, clinical and administrative parts of the project. A vendor was chosen based on its product specifications in relation to the present hospital monitoring infrastructure. In design thinking sessions, critical appraisal of multiple telemonitoring factors was performed by sub teams and a Canvas projectplan was constructed.

**Results:**

The project team was formed of registered nurses, physicians, IT-specialists, Electronic Health Record consultants; a critical care physician was appointed as project leader. The main critical factors were:

- unseamlessly transmitting of both heart and respiratory rates including appropriate movements filtering to the nurse's smartphones
- direct uploading into electronic health record with automated EWS calculation
- nurse driven protocol on EWS follow up.

Philips Healthcare with their IntelliVue Guardian wearable biosensor was the chosen vendor (Figure 1).

**Conclusions:**

Design thinking in a multidisciplinary health care team could positively influence the innovation culture. Scientific evaluation of this wearable will focus on both nurse's acceptance and data storage and is expected in the summer of 2020.

**Fig. 1 (abstract P409).**
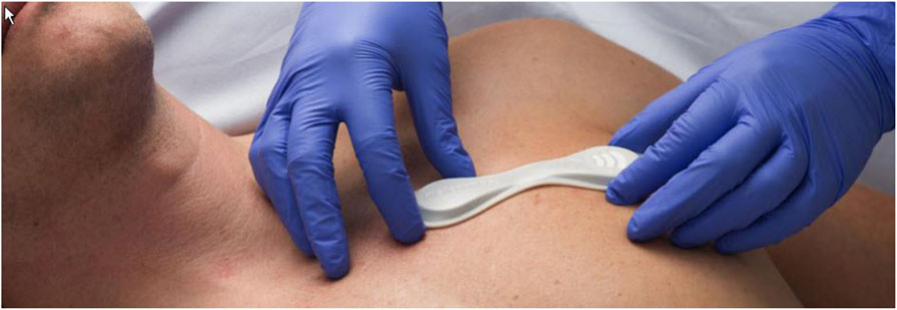
Wearable biosensor

## P410 Hospital discharges: from the intensive care unit to home

### CE Bosso^1^, PC Dutil Ribeiro^2^, FA Falconi de Oliveira Cícero^2^, RD Caetano^3^, A Pireneus Cardoso^4^, L Manata Vanzella^5^, LC Marques Vanderlei^5^

#### ^1^Instituto do Coração de Presidente Prudente, Cardiology, Presidente Prudente, Brazil; ^2^UNOESTE, Presidente Prudente, Brazil; ^3^Santa Casa de Misericórdia de Presidente Prudente, Presidente Prudente, Brazil; ^4^Instituto do Coração de Presidente Prudente, Presidente Prudente, Brazil; ^5^UNESP, Presidente Prudente, Brazil

**Introduction:**

The aim is to determine the epidemiological profile and clinical outcomes of patients receiving discharges directly to home (DDH) in a coronary intensive care unit (CICU). There is an increasing proportion of DDH from intensive care units. Secure DDH can improve health system, resource utilization, and costs.

**Methods:**

We evaluated the characteristics and clinical outcomes of 3118 patients admitted to the CICU between 2014 and 2019. The sample was divided into two groups: DDH group=373; discharge after ward (DAW) group=2745. The analysis was performed by SPSS version 22.0 software, significance level of p <0.05. Data normality was assessed by the Kolmogorov-Smirnov test. For the comparison between continuous variables the Wilcoxon test was used and for the categorical variables the chi-square test was used.

**Results:**

Discharge directly to home increased from 49 (1.57% of all survivors) patients in 2014 to 82 (2.82%) patients in 2019**.** Regarding the baseline characteristics of DDH group, it was found mean age 66.23±13.93, male predominance 57.10%, main hospitalization diagnoses: arrhythmias 24.10%, stent implantation 24.90%, unstable angina 13.40%. Regarding on clinical outcomes: ICU length of stay (DDH=2.59±2.64; DAW: 3.30±3.99; P=0.000), severity by SAPS 3 points (DDH: 40.75±9.99; DAW: 43.42±12.64; P=0.000), readmission rate (DDH: 6.20%; DAW: 9.30%; P=0.043), as well as the need for invasive mechanical ventilation (DDH: 2.90%; DAW: 18.90%; P=0.000), use of vassopressors (DDH: 13,90%; DAW: 32.80%; P=0.000) and blood transfusion (DDH: 1.90%; DAW: 4.10%; P=0.032) were statistically smaller in DDH group (Table1).

**Conclusions:**

Severity, readmission and lengh of stay were lower in patients receiving discharges directly to home. It seems like a safe way to discharge low-risk short stay patients. It seems to save resources and reduce costs, as well as the need for hospital beds. However, futher estudies are needed to actualy evaluate this safety.

**Table 1 (abstract P410).** Characteristics and clinical outcomes related to type of discharge

	Discharge to home	Discharge to ward	P
Age	66.23±13.93	66.76±13.74	0.398
Gender	42.90% F / 57.10% M	37.60% / F 62.40% M	0.044
SAPS3 score	40.75±9.99	43.42±12.64	0.000
Mechanical ventilation	2.90%	18.90%	0.000
Vasopressors	13.90%	32.80%	0.000
Blood transfusion	1.90%	4.10%	0.032
ICU lengh of stay	2.59±2.64	3.30±3.99	0.000

## P411 Appropriateness of antimicrobial use among septic patients managed by the critical care response team: an opportunity for improvement through de-escalation

### S Al Qahtani^1^, H Balkhy^2^, A Ali^3^, K Aljufy^4^, A Alsaed^5^

#### ^1^King Abdulaziz Medical City, ICU, Riyadh, Saudi Arabia; ^2^King Abdulaziz Medical City, WHO, Riyadh, Saudi Arabia; ^3^King Abdulaziz Medical City, IM, Riyadh, Saudi Arabia; ^4^King Abdulaziz Medical City, Pharmacy, Riyadh, Saudi Arabia; ^5^King Abdulaziz Medical City, Biostatistics, Riyadh, Saudi Arabia

**Introduction:**

Critical Care Response Teams (CCRTs) were designed to quickly assess and transfer, rapidly deteriorating ward patients to the intensive care unit. The ultimate goal of a CCRT is to prevent cardiopulmonary arrest, stabilize patients’ condition and help in optimizing the care provided by the primary team in general wards. Septic patients managed by CCRT are most likely to be initiated on one or more antimicrobials, frequently on an empiric basis. The objective of this study was to compare the quantity and quality of antimicrobial use among CCRT patients during three identified stages of their management; before CCRT activation, during and on day four after CCRT activation [1].

**Methods:**

Two hospitals in Riyadh, Saudi Arabia were utilized for this study. Tertiary care hospitals serve over 750,000. Include 1300-beds, 15 different intensive care units, and 50-inpatient wards. All patients above 15 years and managed by the CCRT during the study period were eligible to be included in the study. Case-control design was used to compare septic (cases) and non-septic (controls) CCRT patients, recruited prospectively for five months (January to May 2018).

**Results:**

A total of 315 CCRT patients were included: 157 with sepsis (cases) and 158 without sepsis (controls). Average age was 61.1 ± 20.4 years and 54.6% of the patients were males. Among the 157 septic patients, 97 (61.8%) had a defined septic focus with pneumonia the main diagnosis (33.6%). 60 (38.2%) had no defined septic focus. The most frequent triggers for CCRT activation were changes in respiratory rates (29.5%). An antimicrobial was used during CCRT activation in 96.2% of cases and 65.2% of controls (p < 0.001). Meropenem (51.4%), vancomycin (41.3%), and piperacillin/tazobactam (28.6%) were the most frequently used antimicrobials. More cases were likely to be admitted to ICU than controls (43.9% versus 28.5%, p=0.004). There were no differences in the development of antimicrobial adverse events (31.8% versus 27.2%, p = 0.270) or in mortality (4.5% versus 1.3%, p = 0.104) between cases and controls.

**Conclusions:**

The fact of no de-escalation of antimicrobials were noticed in majority of CCRT patients; this led to the importance of establishing an antimicrobial stewardship hospital wide program.

**References :**

1. Al Qahtani SM et al. Antimicrob Resist Infect Control 8 :186, 2019

## P412 Dynamic colonization with multidrug-resistant bacteria in an infectious diseases intensive care unit

### D Miclaus^1^, L Herbel^1^, T Szilagyi^2^, M Flonta^3^, A Dicea^2^, A Muntean^1^, M Lupse^2^

#### ^1^University Hospital of Infectious Diseases, ICU, Cluj-Napoca, Romania; ^2^University Hospital of Infectious Diseases, Infectious Diseases, Cluj-Napoca, Romania; ^3^University Hospital of Infectious Diseases, Laboratory, Cluj-Napoca, Romania

**Introduction:**

Bacterial resistance is an important issue in Intensive Care Units. Screening patients admitted into ICU for multidrug-resistant bacteria (MDR) nasal and fecal carriage, has become a rule in Romania. Our objective was to weekly monitorize colonization with MDR bacteria and the persistance of carriage of such pathogens, among patients admitted to our hospital ICU and also, to determine the incidence for nosocomial infections associated with this colonizations,considering that colonization precedes infection.

**Methods:**

Prospective observational study:from 01/10/2017 to 15/08/2018 - we have included all adult patients admitted directly to ICU (first admittance only) or with previous general wards admittance for less then 48 hours. We performed nasal and rectal swap screening for MDR bacteria: Meticilin Resistant Staphilococcus Aureus (MRSA), ESBL producing Enterobacteriaceae (ESBL-EN), Carbapenemases Producing Gram negative Bacteria (CBP-GNB), Vancomycin resistant Enterococcus (VRE), during hospitalisation, on day 0,7 and 14.

**Results:**

We have included 75 patients, 34 women, average age 62,9 yrs with average length of hospitalisation of 15 days. Upon admission: 35 patients were colonized with MDR bacteria, in day 7- 65 colonized patients and 39 in day 14.Upon admission we had 5 MRSA, 25 ESBL, 6 CBP and 18 VRE strains. 13 patients had more then one strain colonization.

**Conclusions:**

MDR bacterial colonization is frequent in patients admitted to ICU. Colonization with MDR bacteria during hospitalisation in ICU increased with the length of stay. Colonization could represent a risk factor for increased rates of infection, especially with CBP-GNB and VRE. Active surveillance program for indentification of MDR bacteria colonized patients is necessary and useful.

## P413 Impact of candidaemia on mortality of critically ill burned patients

### A Agrifoglio, L Cachafeiro, E Herrero, M Sánchez, A García de Lorenzo

#### Hospital Universitario La Paz, Intensive Care Medicine Service, Madrid, Spain

**Introduction:**

Sepsis due to Candida is an uncommon but a significant cause of death in burns patients. *Candida* spp. represents the main cause of fungal colonization in burned patients. The incidence of invasive candidiasis occurs between 2 and 21% of burned patients, with an attributable mortality between 30-90% and the incidence of candidaemia is around 3-5%. The aim was analyze the incidence, mortality and risk factors associated with the development of candidaemia in a population of burned patients.

**Methods:**

A retrospective-observational study was conducted (2012-2019) about all patients admitted to our Critical Care Burn Unit and had any microbiological isolation by any *Candida* species. We collected: demographic and epidemiological data, severity scores, characteristics of burns, risk factors, organic supports, days of hospital stay and mortality.

**Results:**

588 burned patients were admitted. 8 candidaemia were diagnosed: 5 *C. albicans*, 1 *C. parapsilosis,* 1 *C. tropicalis,* 1 *C. glabrata*. 5 patients were colonized by yeasts in their burns. The candidaemia were treated empirically with echinocandins. 84% of the patients the mechanism of the burn was the flame. The medians were: age: 47 years; 6 men; TBSA: 42%; depth, 32%; ABSI: 8, APACHE-II: 18. The average hospital stay in the ICU was 45 ± 9 days. All patients on mechanical ventilation at the time of the diagnosis of candidaemia, and all of them also required norepinephrine for septic shock. All had a central venous catheter, urinary catheter, parenteral nutrition and broad-spectrum antibiotic therapy. 3 patients (38%) died, both with candidaemia due to *C. albicans*.

**Conclusions:**

Although the incidence of candidaemia in our population is very low, almost half of the patients who develop it die during admission for refractary septic shock. TBSA > 30%, multiple colonization by *Candida*, parenteral nutrition, broad spectrum antibiotic therapy, central venous catheter and urinary catheterization continue to be the main risk factors for opportunistic fungal infections.

## P414 A cost minimisation analysis of PCR point of care testing for influenza versus PCR lab testing

### M Mclaughlin^1^, D Boyers^2^

#### ^1^Glasgow Royal Infirmary, Anaesthetics/Critical Care, Glasgow, United Kingdom; ^2^Health Economics Research Unit, Health Economist, Aberdeen, United Kingdom

**Introduction:**

Influenza is an acute viral illness with a significant financial burden. Point of care testing for influenza is available and has demonstrated accuracy [1,2], the current gap in knowledge is the question around the opportunity cost of influenza testing. If POCT is financially a less costly test this could free up scarce resource.

**Methods:**

The study adopts a cost minimisation approach. The point of care test is the Roche Cobas® Liat® machine which can detect flu A/B and is compared with the West of Scotland Specialist Virology centre’s established in house multiplex real time PCR assay.The model was developed using Microsoft Excel and has 2 arms comparing analysis of the above mentioned tests.

**Results:**

The model estimates that the total cost of POCT per patient tested is £3926.33 compared with £4053.92 for lab testing (Figure 1). This is a saving of £127.60 per patient when POCT is used. The result swings in favour of the lab test when POCT specificity falls to 95.72%. If the lab could provide the result of influenza testing within 12 hours the result would swing in favour of lab testing. Zanamivir which will potentially be used increasingly in the intensive care setting can more than double the difference between the 2 tests in favour of POCT.

**Conclusions:**

This research suggests that POCT offers potential cost savings in the ICU setting. This is the case as long as POCT specificity is higher than a threshold of 97.52% and the lab take longer that 12 hours to return the result. The sensitivity analysis should allow for external validity given the usual variations in ICU practice.

**References:**

1. Binnicker MJ et al. J Clin Microbiol 53:2353-4, 2015

2. Merckx J et al. Ann Intern Med 167:394-409, 2017

**Fig. 1 (abstract P414).**
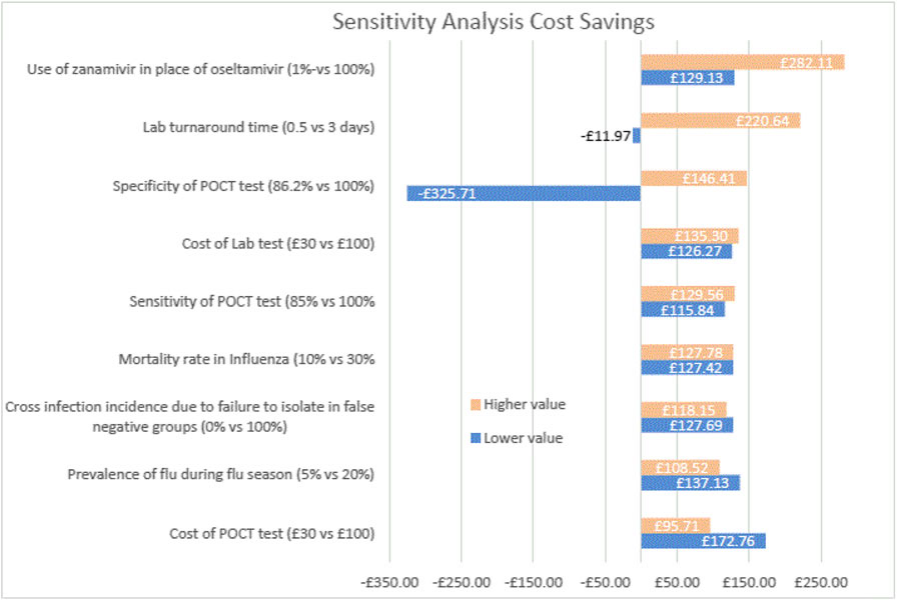
Sensitivity analysis of the cost savings associated with POCT vs. formal lab testing. This table highlights what factors have the largest impact on cost between these 2 methods

## P415 Monobacterial v/s polibacterial multidrug-resistant Klebsiella spp. ventilator-associated pneumonia: factors associated with mortality

### D Adukauskiene, A Dambrauskiene, A Ciginskiene

#### Lithuanian University of Health Sciences, Medical Academy, Kaunas, Lithuania

**Introduction:**

The aim of this study was to compare factors associated with the ICU mortality for VAP due to multidrug-resistant (MDR) *Klebsiella spp.* in case of monobacterial (MO) vs polibacterial (PO) origin.

**Methods:**

Retrospective data analysis of patients treated in ICU with MDR *Klebsiella spp.* strains as pathogens of VAP during three year period was carried out.

**Results:**

Data of 71 patients were evaluated. MO vs PO of MDR *Klebsiella spp.* VAP cases was found to be 35 (49.3 %) vs 36 (50.7%), p = 0.906. The ICU mortality was 9/35 (25.7%) in MO, and 17/36 (47.2%) in PO one, p = 0.060. Statistical significant differences of survivors vs non-survivors in MO and PO VAP due to MDR *Klebsiella spp.* were found in medians of neutrophilosis 78.1% (IQR 72.4 – 82.2) vs 84.2% (IQR 79.7 – 93.3), p = 0.011 and 83.1% (IQR 80.0 – 86.2) vs 87.9% (IQR 83.0 – 89.3), p = 0.029; in proportions of septic shock on diagnosis day 9/26 (34.6%) vs 8/9 (88.9%), p = 0.005 and 1/19 (5.3%) vs 6/17 (35.3%), p = 0.023, renal failure 12/26 (46.2%) vs 8/9 (88.9%), p = 0.026 and 8/19 (42.1%) vs 13/17 (76.5%), p = 0.037. Differences were found in proportions of severe hypoxaemia 1/26 (3.8%) vs 3/9 (33.3%), p = 0.044 only in MO VAP, and RRT 2/19 (10.5%) vs 7/17 (41.2%), p = 0.034, also cardiovascular disease 9/19 (47.4%) vs 16/17 (94.1%), p = 0.002 only in PO one.

**Conclusions:**

The ICU mortality was almost twice higher in polybacterial than monobacterial origin of VAP due to MDR *Klebsiella spp.* Septic shock, renal failure and neutrophilosis were found to be associated with the ICU mortality in both mono/polybacterial origin; severity of hypoxaemia in case of monobacterial, but comorbidities in polybacterial onset.

## P416 Ventilator-associated pneumonia due to multidrug-resistant Klebsiella spp.: antibacterial resistance and predictors of ICU mortality

### D Adukauskiene, A Dambrauskiene, A Ciginskiene

#### Lithuanian University of Health Sciences, Medical Academy, Kaunas, Lithuania

**Introduction:**

The aim of the study was to evaluate the antibacterial resistance of multidrug-resistant (MDR) *Klebsiella spp.* isolates as pathogens of ventilator-associated pneumonia (VAP) and to determinate predictors of ICU mortality.

**Methods:**

Retrospective data analysis of patients (pt) treated in ICU with MDR *Klebsiella spp.* strains as pathogens of VAP during three years period was carried out.

**Results:**

Data of 71 pt were evaluated. All tested strains of MDR *Klebsiella spp.* were resistant to ampicillin, piperacillin, cefuroxime. Resistance to amoxicillin/clavulanate was 35/36 (97.2%), ampicillin/sulbactam and cefotaxime 70/71 (98.6%), piperacillin/tazobactam 43/71 (60.6%), meropenem and imipenem 0/71 (0%), ertapenem 2/30 (6.7%), amikacin 4/59 (6.8%), gentamicin 40/71 (56.3%), ciprofloxacin 51/68 (75.0%), cefoperazon/sulbactam14/45 (31.1%).The ICU mortality was 26/71 (36.6%). Statistical significant differences of survivors vs non-survivors were found in median of neutrophilosis on diagnosis day 80.9% (IQR 72.6 -83.6) vs 87.5% (IQR 81.4 -9.7), p < 0.001,and between proportions of septic shock on diagnosis day10 (22.2 %) vs 14 (53.8%), p = 0.007, renal failure 20 (44.4%) vs 21 (80.8%), p = 0.003, renal replacement therapy (RRT) 4 (8.9%) vs 10 (38.5%), p = 0.003, Charlson Comorbidity Index ≥ 5 – 15 (33% ) vs 15 (57.7%), p = 0.045, cardiovascular 28 (62.2%) vs 25 (96.2%), p = 0.002, kidney 4 (8.9%) vs 9 (34.6%), p = 0.007 and autoimmune 0 (0%) vs 5 (19.2%), p = 0.005 disease. Odds ratio (OR) for ICU mortality for RRT was OR 4.64 (95% CI 1.16-18.62), for septic shock OR 4.01 (95% CI 1.17 -13.67), and for neutrophilosis OR 1.16 (95% CI 1.05 -1.28).

**Conclusions:**

VAP due to MDR Klebsiella spp. was associated with the high rate of antibacterial resistance to aminopenicillins, 3rd generation of cephalosporins, fluoroquinalons, aminoglycosides, but carbapenems. The ICU mortality was 36.6%. The RRT, septic shock and neutrophilosis on diagnosis were predictorsfor ICU mortality in patients with VAP due to MDR Klebsiella spp.

## P417 Role of deoxyribonucleic acid in microbial biofilm in pathogenesis of bacterial infection

### V Ziamko^1^, A Dzyadzko^2^, V Okulich^1^

#### ^1^Vitebsk State Medical University, Vitebsk, Belarus; ^2^Minsk Scientific and Practical Center of Surgery, Transplantation and Hematology, Minsk, Belarus

**Introduction:**

It has been known about possibility of microorganisms to create specific multi-layered structures called biofilms. Non-cellular deoxyribonucleic acid actively participates in regulation of properties of biofilms. Thus, in biofilms transfer of genetic information including genes responsible for sensitivity to antibacterial drugs occurs much more often than in single-living bacterial cells. However, despite the involvement of extracellular deoxyribonucleic acid in adhesive processes and intercellular interactions, its role has not been fully understood.

**Methods:**

238 isolates isolated from sputum and pharynx of 175 patients during 2016-2019 were studied. Patients were divided into two groups: the 1st group of 139 people (79,4%) had severe respiratory infections, the 2nd of 36 people (20,6%) - respiratory infections of moderate severity.

**Results:**

A method was developed for determining percentage of deoxyribonucleic acid in microbial community using 4'6-diamidino-2-phenylindole dihydrochloride. Average age of the 1st group was higher than the second (p<0.05). *Pseudomonas aeruginosa* had the largest mass of biofilm and percentage of deoxyribonucleic acid in group 1, 48,25 [30.5-70.1] mcg/ml and 5.21 [2.17-7.67] %, p = 0.04. A strong relationship was found between percentage of deoxyribonucleic acid in *Pseudomonas aeruginosa* and severity of disease, r = 0.73, p<0.05. The incidence of adverse outcomes in isolating antibiotic resistant isolates was higher than in antibiotic sensitive (p<0.05). Analysis of results made it possible to propose fatal outcome when mass of microbial biofilm is > 47.5 mcg/well and percentage of deoxyribonucleic acid is >2.33% (p<0.01).

**Conclusions:**

Pathogens with high biofilm weight and percent of deoxyribonucleic acid cause disease progression and death.

## P418 The clinical impact of 24h/7day blood culture identification using the genmark ePlex blood culture panels

### A Krifors^1^, D Heimer^2^, S Golbob^2^, Z Omar^2^, C Svensson^2^, G Rådberg^2^, C Carlander^3^

#### ^1^Västmanlands sjukhus Västerås, Department of Infectious Diseases, Västerås, Sweden; ^2^Department of Clinical Microbiology, Department of Clinical Microbiology, Västerås, Sweden; ^3^Department of Infectious Diseases, Department of Infectious Diseases, Västerås, Sweden

**Introduction:**

Implementation of rapid molecular blood culture diagnostics in the clinical management of sepsis is essential in providing fast pathogen identification and resistance gene testing.The Genmark ePlex blood culture panels offer a broad antimicrobial spectrum with minimal hands-on time and around 1.5h time to result. By implementing ePlex analysis when the department of clinical microbiology is closed, the test can be optimally used in terms of clinical impact and costs.

**Methods:**

Prospective ePlex testing on consecutive non-duplicate positive blood cultures, primarily from the emergency room, incubated at a satellite incubator at the 24h/7day open department of Clinical Chemistry. The routine bio-medical analysts performed the analyses. The result, except findings of CoNS, was made available to the attending physician. Cultures were transferred to the Department of Clinical Microbiology for conventional diagnostics the next day. The ePlex results were compared to conventional diagnostics for pathogen identification and time to result. The clinical impact was assessed by investigation of the patients´medical records.

**Results:**

Forty-four cultures were analyzed with ePlex (Figure 1). Complete agreement with conventional diagnostics was observed in 38/44 cases. No false-positive results were observed, yielding a sensitivity and specificity of 90% and 100% respectively for target pathogens. Time to result was, on average, 10.4 h faster with ePlex compared to conventional diagnostics. Antimicrobial therapy could have been optimized in 5 patients based on the ePlex result, but treatment was only changed in one case (E.coli CTX-M+) receiving meropenem 8.5 h before the antibiogram was available.

**Conclusions:**

The ePlex blood culture panels provide high accuracy and significantly faster results. The current implementation offers substantial potential value at a minimal cost, and is a feasible approach to 24-h/7 days blood culture diagnostics in many hospital settings. However, efforts to increase adherence are needed.

**Fig. 1 (abstract P418).**
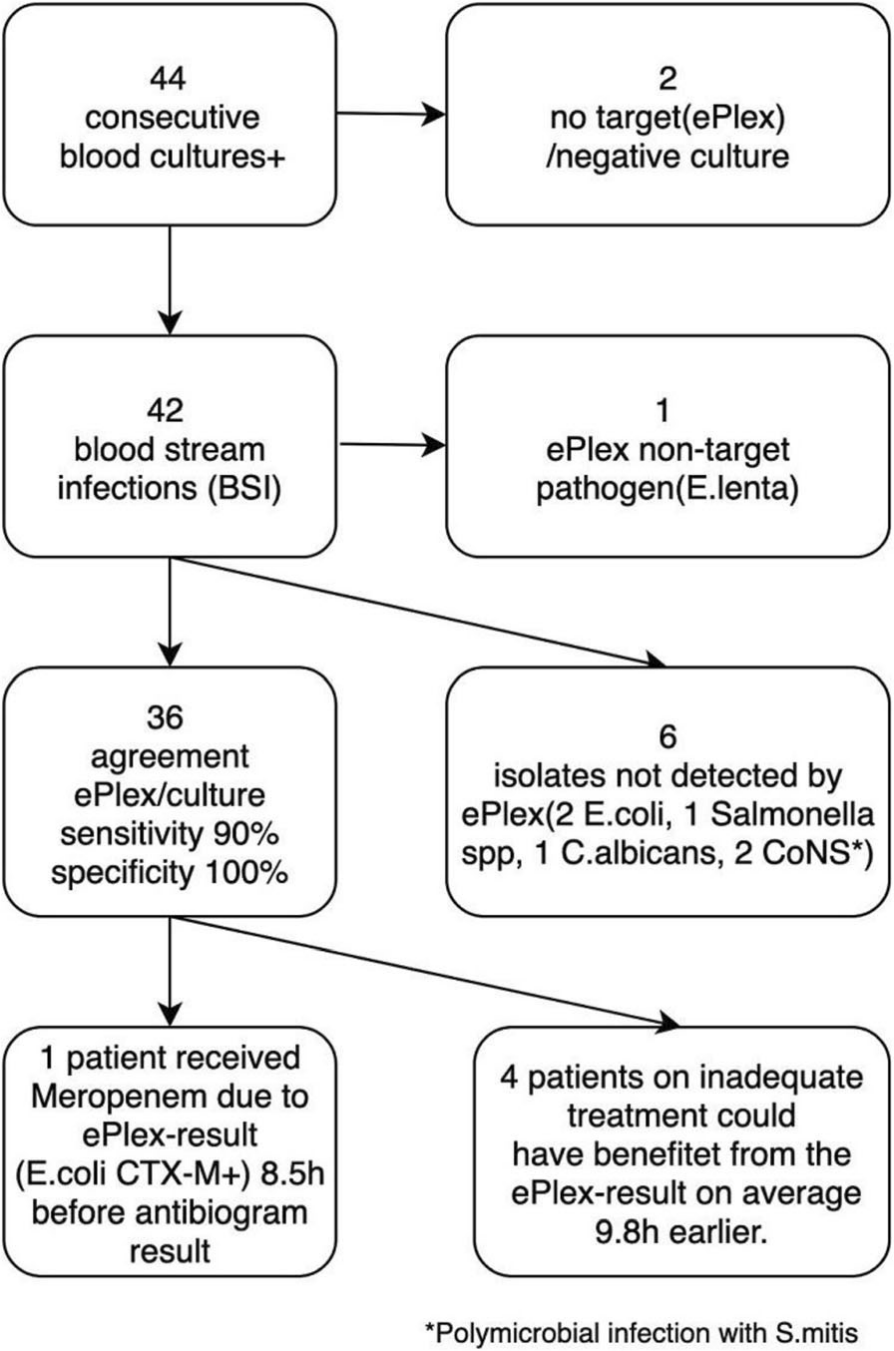
Result of consecutive positive blood cultures analyzed with ePlex

## P419 Impact of rising Klebsiella pneumoniae infections and its resistance on the outcome of hospitalized patients in the intensive care unit of a tertiary healthcare hospital, Saudi Arabia

### A Al bshabshe^1^, M R.P. Joseph^2^, A Assiri^3^, M Hamid^2^, M Bahis^4^, I Assiry^5^, A Al-Hakami^6^

#### ^1^King Khalid university, Medicine/Critical Care, Abha, Saudi Arabia; ^2^King Khalid university, Department of Microbiology, Abha, Saudi Arabia; ^3^King Khalid university, Critical Care Department, Abha, Saudi Arabia; ^4^Aseer central hospital, critical Care Department, Abha, Saudi Arabia; ^5^Aseer central hospital, Abha, Saudi Arabia; ^6^King Khalid university, department of Microbiology, Abha, Saudi Arabia

**Introduction:**

Nosocomial infections caused by *Klebsiella pneumoniae, Acinetobacter baumannii* and other gram-negative organisms have emerged as a major health problem globallyespecially in intensive care units (ICU). Continued surveillance and finding a suitable drug is of central priority. This study examines *Klebsiella pneumoniae* and other nosocomial infections in the intensive care unit of Aseer Central Hospital, determines their antibiogram, and evaluates predictors of mortality.

**Methods:**

The present investigation was a retrospective cross-sectional study based on data collected from patients in the intensive care unit (ICU) of Aseer Central Hospital, Saudi Arabia (2018-2019). Demographic, microbiologic, antimicrobial treatment and outcomes were collected from 150 patients with bacteremia, pneumonia, and other infections. A total of 150 patients were included (2018, n= 68; 2019, n= 82). Identification of isolates and *in vitro* susceptibilities were done by Vitek 2 system. Collected data were analyzed by descriptive statistics and regression models.

**Results:**

One hundred and fifty patients were included in our study (51 females and 99 males). The clinical specimens were mainly derived from respiratory (60.0%), followed by blood (16.7%), urine (11.3%), wound and skin swab (4.7%), CSF (2.7%), pus, discharge and swab (2.7%), and abdominal fluid and aspirate ((2.7%). Mortality was 36.0% (54/150*100); *Klebsiella pneumoniae* was responsible for 27 case (27/54*100 = 50%). *Acinetobacter*spp. (35.2%) and *Pseudomonas aeruginosa* (9.3%) infections while the remaining organisms accounted for 5.6%. The overall *in vitro*resistance was 49.5% and shown an increase in 2019 (p <0.01) (fig 1).

**Conclusions:**

Half of the ICU mortalities were caused by *K. pneumoniae* . The frequency of *K. pneumoniae* and its resistance to antimicrobials was high and obviously rising as for other gram-negative bacteria. Our results suggest that attention should be paid to strengthen measures for better infection control measures.

**Fig. 1 (abstract P419).**
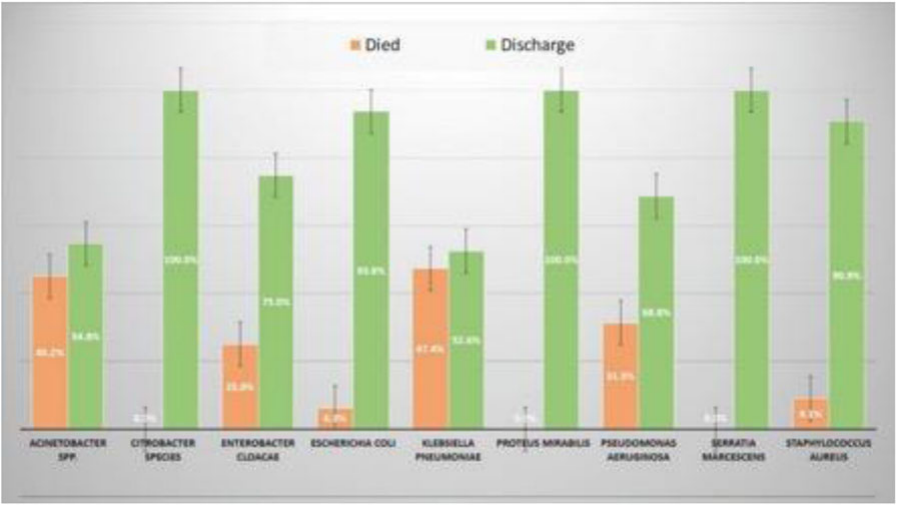
Clustered bar count of dominant microorganisms causing ICU infections according to patient’s outcomes (2018-2019)

## P420 Patterns of presentation and predictors of ICU mortality among HIV infected patients

### AE Laher^1^, RW Maphula^1^, GA Richards^2^

#### ^1^University of the Witwatersrand, Emergency Medicine, Parktown, Johannesburg, South Africa; ^2^University of the Witwatersrand, Critical Care, Parktown, Johannesburg, South Africa

**Introduction:**

Data on the outcomes of HIV infected intensive care unit (ICU) patients in developing countries is limited. There are more people living with HIV in South Africa than any other country in the world. This study aimed to describe the patterns of presentation as well as determine factors that influences mortality in HIV infected patients at an ICU facility in Johannesburg.

**Methods:**

Consecutive medical records of 204 HIV infected individuals admitted to the Charlotte Maxeke Johannesburg Academic Hospital adult ICU during 2017 were reviewed. Data was described and subjected to univariate and multivariate analysis.

**Results:**

Of the 903 total admissions during the period analysed, 204 (22.6%) were HIV infected. Ninety-five (46.6%) patients were admitted for sepsis related illnesses, 69 (33.8%) for post-operative care and 40 (19.6%) for non-sepsis related illnesses. HIV infected patients had a median length of ICU stay of 5 (2-9) days and ICU mortality of 33.3% (n=68). On multivariate analysis of significant parameters identified from the univariate analysis, requirement for inotropes/vasopressors (p=0.009), mechanical ventilation (p=0.037) and a non-sepsis related diagnosis (p=0.030) were associated with an increase in ICU mortality. The Risk Adjusted Mortality Ratio (RAMR – observed ICU mortality rate/ APAHCE II predicted ICU mortality rate) was 0.51, which indicates that the APACHE II score overestimated ICU mortality in HIV infected patients by approximately two-fold.

**Conclusions:**

Inotrope/vasopressor administration, mechanical ventilation and a non-sepsis related diagnosis is significantly associated with mortality in HIV infected patients in the ICU. The APACHE II score overestimates mortality in HIV infected ICU patients.

## P421 Impact of beta-lactamase detection reagent on rapid diagnosis of ESBL-producing pathogens using urine samples in patients with gram-negative bacteriuria

### J Yoshimura^1^, Y Ooi^2^, Y Umemura^1^, K Yamakawa^1^, S Fujimi^1^

#### ^1^Osaka General Medical Center, Division of Trauma and Surgical Critical Care, Osaka, Japan; ^2^Osaka General Medical Center, Department of Clinical Laboratory, Osaka, Japan

**Introduction:**

The rapid increase of extended spectrum β-lactamases (ESBL)-producing pathogens worldwide makes it difficult to choose appropriate antibiotics in patients with Gram-negative bacterial infection. Cica-beta reagent (KANTO CHEMICAL, Tokyo, Japan) is a chromogenic test to detect beta-lactamases such as ESBL from bacterial colonies. The purpose of the study was to reveal whether Cica-beta reagent could detect ESBL-producing pathogens directly from urine rather than bacterial colonies to make a rapid bedside diagnosis of the antibiotic susceptibility of Gram-negative pathogens.

**Methods:**

We conducted a prospective observational study from July 2019 to October 2019. Patients were eligible if they were performed urinary culture tests and Gram negative pathogens were detected at least 1+ from their urine samples. The urine sample was centrifugated at 200 x g for 5min. The supernatant of sample was re-centrifugated at 1500 x g for 5min and the pellet was mixed with Cica-beta reagent. The test was considered positive when the enzymatic reaction turned from yellow to red or orange. The reference standard was the result of urine culture and the antibiotic susceptibility test according to Clinical and Laboratory Standards Institute.

**Results:**

We included 331 urine samples during the study period. One hundred ninety eight patients diagnosed UTI among them. ESBL producing *Enterobacteriaceae* was isolated from 73 samples. Cica-beta reagent showed sensitivity of 79.5% and specificity of 99.2% in patients with Gram-negative bacteriuria. Sensitivity and specificity were improved 89.7% and 100%, respectively when it was performed in patients with UTI.

**Conclusions:**

Cica-beta reagent would be an efficient test for the detection of ESBL producing pathogens in urine. Because it could provide immediate information about ESBL, it might be a point-of-care test to predict causative pathogens and guide appropriate antibiotics in patients with UTI.

## P422 Effect of bundle approach on preventing hospital-acquisition of drug-resistant pathogens in intensive care units

### A Ota^1^, M Kawanami^1^, J Yoshimura^2^, Y Umemura^2^, K Yamakawa^2^, S Fujimi^2^

#### ^1^Osaka General Medical Center, Nursing Department, Osaka, Japan; ^2^Osaka General Medical Center, Division of Trauma and Surgical Critical Care, Osaka, Japan

**Introduction:**

The increasing emergence and spread of drug-resistant pathogens all over the world has become catastrophic world health problems. In fact, we experienced an uncontrollable outbreak of multidrug-resistant pathogens and ICU closure for 3 weeks in January 2013. Based on the experience, we built an infection control bundle including contact precautions for every patient in the ICU, carrying personal alcohol-based hand rubs, cohorting patients who acquired drug-resistant pathogens, active surveillance cultures once a week, and enhanced environmental disinfection in January 2013. The purpose of this study is to evaluate the effectiveness of the systemic infection control bundle approach against acquisition of drug-resistant pathogens.

**Methods:**

This was a retrospective historical control study conducted from April 2008 to March 2019 in 18-bed ICU of a tertiary care hospital in Japan. Every patient admitted to the ICU during the study period was eligible. Patients were divided into 2 groups: Conventional (from April 2008 to December 2012) or Intervention (from January 2013 to March 2019).The primary outcome was the number of hospital-acquired MRSA. We defined hospital-acquisition as the isolation of MRSA after 48 hours of hospitalisation.

**Results:**

A total of 13,047 patients were included during the study period. There were 5,128 patients in the control group (39.3%) from 2008 to 2012 and 7,919 patients in the intervention group (61.7%) from 2013 to 2019. The hospital-acquisition of MRSA was observed 284 patients (5.5%) in the control group and 150 patients (1.9%) in the intervention group. The interrupted time-series analysis suggested that the intervention was significantly associated with reduced hospital-acquisition of MRSA [risk ratio, 0.342(95% confidence interval, 0.282-0.416);< 0.01] (Fig.1).

**Conclusions:**

The Bundle approach could be an effective strategy to prevent hospital-acquisition of drug-resistant pathogens in ICUs.

**Fig. 1 (abstract P422).**
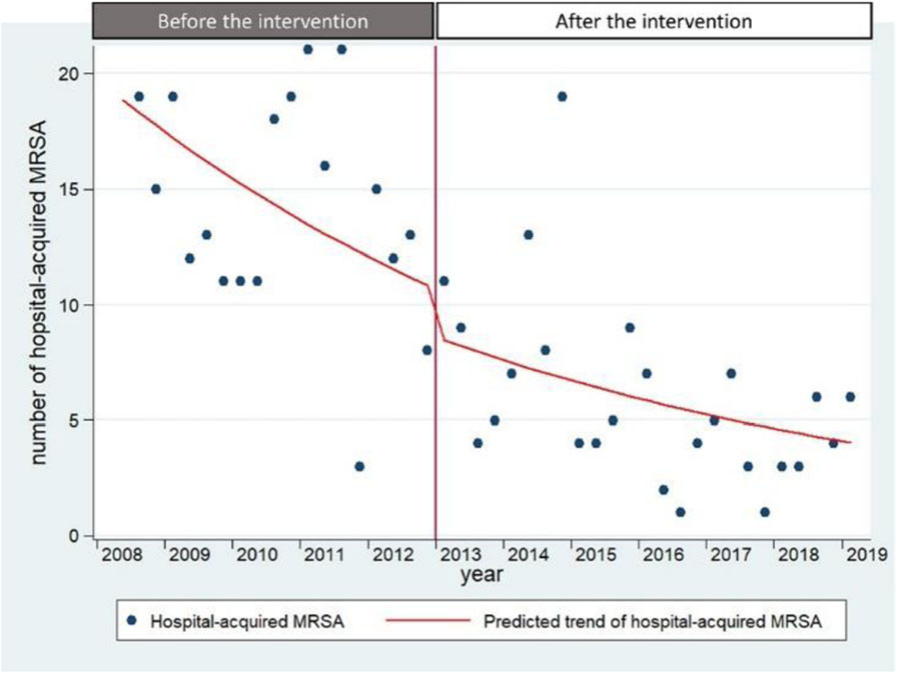
Predicted trend of hospital-acquired MRSA by interrupted time-series analysis

## P423 Outcomes in patients (pts) with failure of initial antibiotic therapy for hospital-acquired/ventilator-associated bacterial pneumonia (HABP/VABP) prior to enrollment in the phase 3 ASPECT-NP trial of ceftolozane/tazobactam (C/T) vs. meropenem (MEM)

### M Kollef^1^, JF Timsit^2^, I Martin-Loeches^3^, RG Wunderink^4^, JA Huntington^5^, E Jensen^5^, CJ Bruno^5^, B Yu^5^, DJ Wolf^5^, EG Rhee^5^

#### ^1^Washington University School of Medicine, St. Louis, MO, United States; ^2^Université Paris Diderot/Hôpital Bichat, Paris, France; ^3^St James´ Hospital, Dublin, Ireland; ^4^Northwestern University Feinberg School of Medicine, Chicago, IL, United States; ^5^Merck & Co., Inc., Kenilworth, NJ, United States

**Introduction:**

In the ASPECT-NP trial, C/T was noninferior to MEM for the treatment of HABP/VABP. We evaluated outcomes from that study in the subgroup of pts failing current antibacterial therapy for HABP/VABP at enrollment.

**Methods:**

ASPECT-NP was a randomized, controlled, double-blind, phase 3 trial in which mechanically ventilated pts with HABP/VABP received 3 g C/T or 1 g MEM every 8 h for 8-14 days. Pts with >24 h of active gram-negative antibacterial therapy within 72 h prior to first dose of study therapy were excluded, except those pts failing current treatment (i.e. signs/symptoms of the current HABP/VABP were persisting/worsening despite ≥48h of antibiotic treatment). Primary and key secondary endpoints, respectively, were 28-day all-cause mortality (ACM) and clinical response at test of cure (TOC; 7-14 days after end of therapy) in the intent to treat (ITT) population. Pts failing current antibacterial therapy for HABP/VABP were prospectively categorized as a clinically relevant subgroup.

**Results:**

At baseline, failing current therapy for HABP/VABP was reported in 53/362 (15%) C/T and 40/364 (11%) MEM ITT pts, mostly piperacillin/tazobactam (34%), 3rd/4th-generation cephalosporins (31%), fluoroquinolones (29%), and aminoglycosides (12%). Baseline demographic and clinical characteristics in this subgroup, including prior therapy regimen, were generally similar between treatment arms. There were greater proportions of patients with ESBL+ Enterobacterales (30%) and *Pseudomonas aeruginosa* (25%) in the C/T arm than the MEM arm (20% and 13%, respectively). Lower 28-day ACM was seen with C/T than MEM, as evidenced by 95% confidence intervals for treatment differences that excluded zero (Figure 1); statistical significance cannot be assumed because subgroup analyses in this study were not corrected for multiplicity.

**Conclusions:**

C/T was an effective treatment for HABP/VABP pts who had failed initial therapy.

**Fig. 1 (abstract P423).**
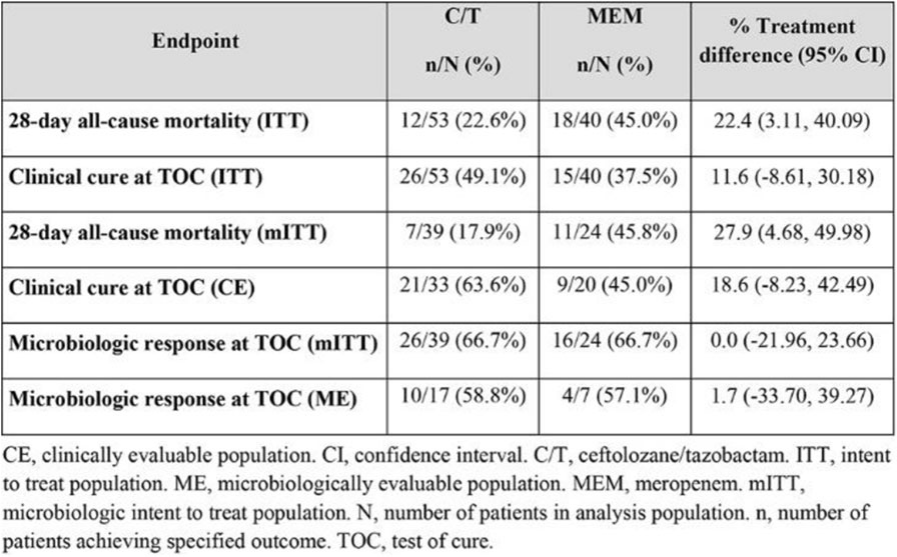
Efficacy outcomes in patients who were failing current therapy for HABP/VABP at baseline

## P424 Impact of team-based approach on preventing catheter-related bloodstream infection in critically ill setting

### A Hamana^1^, A Sawada^1^, J Yoshimura^2^, Y Umemura^2^, K Yamakawa^2^, S Fujimi^2^, Y Sakajo^1^

#### ^1^Osaka General Medical Center, Department of Pharmacy, Osaka, Japan; ^2^Osaka General Medical Center, Division of Trauma and Surgical Critical Care, Osaka, Japan

**Introduction:**

Catheter-related blood stream infection (CRBSI) is common serious infections and associated with increased mortality in intensive care units (ICU). One of the most important strategy to prevent CRBSI is to minimize the duration of central venous catheterization. We built a medical team consisting of doctors, nurses and pharmacists in ICU to discuss whether patients needed central venous catheter (CVC) in terms of monitoring hemodynamics and administering drugs, and recommend catheter removal to attending physicians every day in April 2019. The purpose of this study is to evaluate whether our team-based approach could shorten the total duration of catheterization and reduce CRBSI.

**Methods:**

This was a retrospective historical control study conducted from April 2018 to October 2019 in the ICU of a tertiary care hospital in Japan. Every patient admitted to the ICU during the study period was eligible if they were inserted CVC. Patients were divided into 2 groups: Conventional (from April 2018 to March 2019) or Intervention (from April 2019 to October 2019). We set the primary endpoint as onset of CRBSI. The secondary endpoints included the duration of central venous catheterization, the length of ICU stay and hospital mortality. CRBSI was defined as bloodstream infection in patients with CVC, not related to another site.

**Results:**

We included 428 patients: 259 in the Conventional group and 169 in the Intervention group. The reduced, though nonsignificant, tendency of CRBSI was observed in the Intervention group [hazard ratio, 0.341(95% confidence interval, 0.074-1.557; p = 0.213)]. The Intervention group was significantly associated with reduced duration of central venous catheterization (5 days vs 7 days; p < 0.01). No difference was observed in the length of ICU stay and in-hospital mortality between groups.

**Conclusions:**

The team-based approach to assess CVC necessity could shorten the duration of central venous catheterization and might reduce CRBSI.

## P425 Assessment of antimicrobial susceptibility of the most common Gram-negative intensive care unit (ICU) respiratory pathogens in deciding antimicrobial therapy

### P Moise^1^, M Gonzalez^1^, I Alekseeva^1^, D Lopez^2^, B Akrich^3^, B Akrich^3^, A DeRyke^1^, M Hackel^4^, M Motyl^5^

#### ^1^Merck & Co., Inc, Medical Affairs, Kenilworth, United States; ^2^MSD, Medical Affairs, Madrid, Spain; ^3^MSD France, Medical Affairs, Paris, France; ^4^IHMA, Microbiology, Schaumburg, United States; ^5^Merck & Co., Inc, Microbiology, Kenilworth, United States

**Introduction:**

Empiric antibiotic therapy decisions are based upon a combined prediction of infecting pathogen and local antibiotic susceptibility, adapted to patients’ characteristics. The objective of this study was to describe the pathogen predominance and to evaluate the probability of covering the most common Gram-negative pathogens in ICU patients with respiratory infections.

**Methods:**

Data were collected from multiple US and European hospitals as part of the SMART Surveillance Program (2018). MIC (mg/L) testing was performed by broth microdilution, with susceptibility defined as follows for *P. aeruginosa* & Enterobacterales: ceftolozane/tazobactam MIC ≤4 & ≤2; piperacillin/tazobactam MIC ≤16 & ≤8; meropenem MIC ≤2 & ≤1; ceftazidime MIC ≤8 & ≤8, respectively.

**Results:**

78 hospitals from 24 countries provided 3384 Gram-negative respiratory isolates from patients located in an ICU in the US (22%), Eastern Europe (40%) and Western Europe (38%) in 2018. The 4 most common pathogens isolated were *P. aeruginosa* (26%), *K. pneumoniae* (15%), *E. coli* (13%), and *A. baumannii* (10%). Among Enterobacterales, 30% (588/1955) were ESBL positive. Figure 1 provides the probability of covering the most common respiratory Gram-negative pathogens from ICU patients. Co-resistance between commonly prescribed first line β-lactam antibiotics is common: when non-susceptibility (NS) of one agent was present, susceptibility to other β-lactams was generally <35%. Ceftolozane/tazobactam provided the most reliable in vitro activity in both empiric and adjustment prescribing scenarios compared to other β-lactam antibiotics.

**Conclusions:**

Ceftolozane/tazobactam ensured a wide coverage of the most common Gram-negative respiratory pathogens demonstrating high susceptibility levels and provided the most reliable in vitro activity in both empiric and adjustment antibiotic prescribing scenarios. Further studies are needed to define the clinical benefits that may translate from these findings.

**Fig. 1 (abstract P425).**
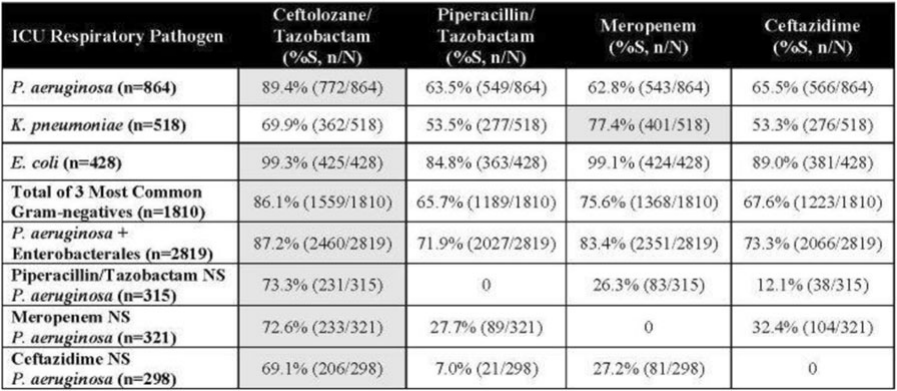
Probability of covering the most common respiratory Gram-negative pathogens from ICU patients

## P426 Evaluation of compliance of ICU staff for VAP prevention strategies on the outcome of patients

### A Kaur

#### Fortis Hospital, Critical Care, Mohali, India

**Introduction:**

Ventilator-associated pneumonia is the most common nosocomial infection diagnosed in adult critical care units. It is associated with prolonged duration of mechanical ventilation, increased ICU stay and increased mortality. It continues to be a major challenge to the critical care physicians despite advances in diagnostic and treatment modalities. The primary objective of the study was to determine the compliance of ICU staff towards VAP prevention bundle and secondary objective was to determine the incidence, risk factors and outcome of VAP patients.

**Methods:**

Single center, prospective, observational study carried out from February 2017 to July 2018. Patients mechanically ventilated for more than 48 hours and satisfying the inclusion and exclusion criteria were enrolled in the study. VAP was diagnosed using the CDC criteria and clinical pulmonary infection score. VAP preventive strategies were employed and compliance of ICU staff was assessed.

**Results:**

A total of 1617 patients were admitted to ICU over the set time period and out of them 483 patients were ventilated for more than 48 hours. Among them only 166 patients fulfilled the inclusion and exclusion criteria and were enrolled in the present study. Excellent compliance was observed in head end elevation, sedation vacation, stress ulcer prophylaxis, and heat moist exchanger filter use, good compliance in oral care and hand hygiene and moderate to poor compliance in subglottic suctioning. The incidence of VAP was 24.7% with a VAP rate of 30.87/1000 ventilator days. There was a significant correlation between primary diagnosis, hemodialysis, massive blood transfusion and development of VAP (p<0.05)). Mean duration of ventilation (p<0.001) and mortality (p<0.05) were highly significant in VAP patients.

**Conclusions:**

Improvement in compliance towards VAP bundle and reduction of risk factors can help decrease incidence of VAP and related morbidity and mortality.

## P427 Ventilation-associated pneumonia prevention bundle (VAP-p) efficacy in critically ill children mechanically ventilated for more than 48 hours

### I Vitali^1^, AM Dell´Anna^2^, L Dassatti^3^, O Genovese^1^, M Piastra^1^, P Filetici^3^, M Antonelli^2^, G Conti^1^

#### ^1^Fondazione Policlinico Universitario A. Gemelli IRCCS, Paediatric Intensive Care Unit, Roma, Italy; ^2^Fondazione Policlinico Universitario A. Gemelli IRCCS, Intensive Care Unit, Roma, Italy; ^3^Fondazione Policlinico Universitario A. Gemelli IRCCS, Oral Surgery, Roma, Italy

**Introduction:**

Preventive strategies are effective in reducing ventilation-associated pneumonia (VAP) in adults [1, 2]. In paediatric population there are no data about VAP prevention, so we introduced a new bundle (VAP-p) based on the available evidence for adults.

**Methods:**

This was designed as a before-after study. We enrolled all patients admitted to 8-bed medical-surgical paediatric ICU at Gemelli Hospital in Rome, requiring mechanical ventilation for at least 48 hours. Patients with pre-existing tracheostomy were excluded. VAP-p has been introduced since 2018 in order to improve quality of assistance. Our bundle consisted in twice a day oral hygiene with chlorhexidine swab, daily check of oral bacterial colonization and aspiration prevention. Comparison was made with an historical group including patients admitted before VAP-p introduction (since 2016 to 2017). All data about demographics, antimicrobial therapy, ICU stay and treatments, were collected.

**Results:**

162 patients were included (82 after and 80 before VAP-p introduction). 5(6%) events of VAP were recorded in VAP-p group compared to 16 (20%, p=0.01) VAP-p group had less VAP per days of mechanical ventilation (1/100 compared to 3.3/100 p=0.01). Multivariate analysis yielded an OR of 0.23 (95%CI 0.07-0.81) for VAP incidence after bundle introduction. Mortality rate was slightly reduced in VAP-p group (2.4%vs 6.2% p=ns). Patients who developed VAP required more days on mechanical ventilation and had higher mortality rate (12 vs 5 days p<0.001 and 14%vs 3% p=0.047, respectively).

**Conclusions:**

Our VAP-p seems effective in reducing VAP incidence in critically ill paediatric population.

**References:**

1. Torres A et al. Eur Respir J 10 :50, 2017

2. Kalil A et al. Clin Infect Dis 63: e61-e111, 2016

## P428

**Withdrawn**

## P429 ICU-related Gram-negative bacteremia: outcome and the role of colistin susceptibility

### M Karvouniaris, T Zafeiridis, P Katsiafylloudis, A Palioura, M Chatzimichail, A Papadogoulas, P Papamichalis, D Papadopoulos, S Karagiannis, A Komnos

#### General Hospital of Larissa, ICU, Larisa, Greece

**Introduction:**

The aim of the present study is to describe the demographic, clinical, microbiological aspects and the outcome of patients with intensive care unit-related (ICU-related) bacteremia. Moreover, we aimed to study the patient outcome in association with colistin susceptibility.

**Methods:**

Retrospective, single-center study in a 16-bed ICU for 12 months, from 1/10/2018 to 30/9/2019. ICU-related bacteremia was defined as bacteremia in patients with ICU stay >48 hours or ICU readmission (first admission ≥ 1 month before). Only the first episode of bacteremia was considered. The primary outcome was 28-day mortality. Data regarding clinical, demographic and outcome characteristics were retrieved from the patient files. The hospital’s ethics committee approved the present protocol. Moreover, the patients with bacteremia due to colistin-resistant pathogens were compared with the patients affected by colistin sensitive microbes.

**Results:**

Forty episodes of Gram-negative ICU bacteremia were collected during the aforementioned period in 40 patients (61.5% male) with a mean age and APACHE II of 60.6±16.8 years and 18±8.1, respectively. The event had taken place at an average of 13.7 days. The responsible isolates were resistant to carbapenems in 82.5% of the episodes. The majority of the events were due to a single isolate (85%). *Acinetobacter baumannii* and *Klebsiella pneumoniae* presented the majority of the implicated microbes (35% and 37.5%, respectively). The crude 28-day mortality was 30%. Finally, we could not detect any difference in mortality between the colistin sensitive and the colistin-resistant pathogens (Figure 1).

**Conclusions:**

The present study denotes that, in a setting of extremely drug-resistant pathogens with limited treatment options, Gram-negative bacteremia in the ICU is associated with increased mortality.

**Image 1 :**

**Fig. 1 (abstract P429).**
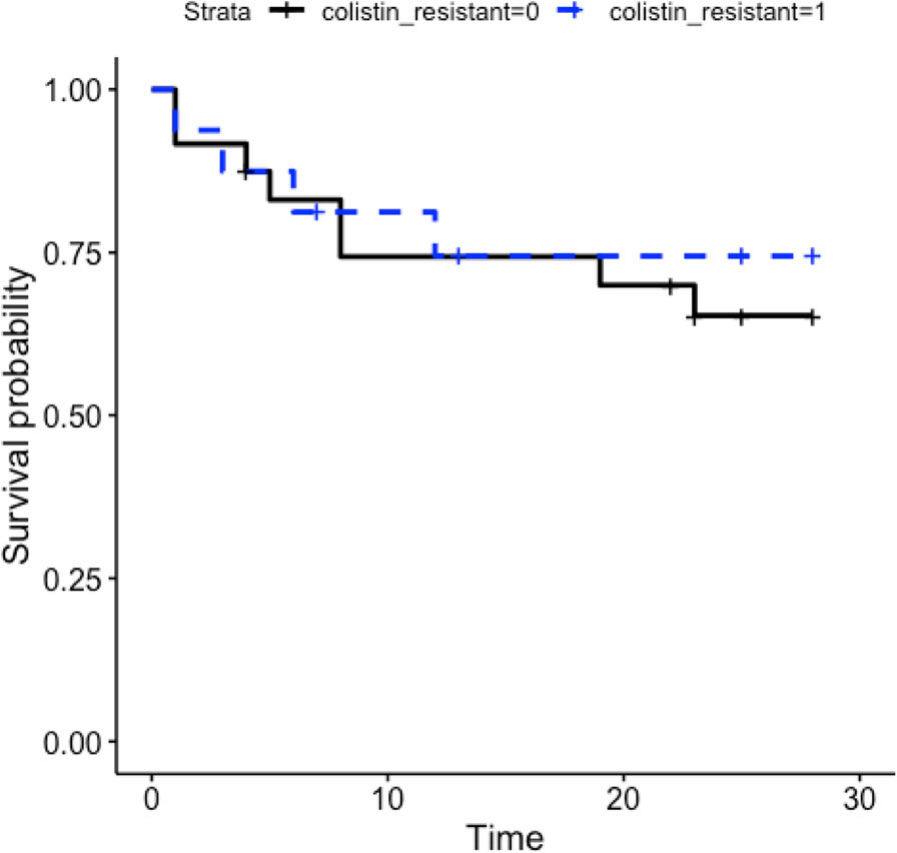
Kaplan-Meier survival curve (Black solid and blue dashed line present patients with colistin sensitive and colistin resistant pathogens, respectively)

## P430 Characterization of resistance mechanisms affecting ceftolozane/tazobactam in Enterobacterales and Pseudomonas aeruginosa ICU isolates using whole genome sequencing (STEP study)

### M Hernández-garcia^1^, CC Chaves^2^, JM Melo-Cristino^3^, DS Silva^4^, AR Vieira^5^, MP F. Pinto^6^, JD Diogo^7^, EG Gonçalves^8^, JR Romano^9^, RC Cantón^1^

#### ^1^Hospital Ramón y Cajal-IRYCIS, Microbiology Department, Madrid, Spain; ^2^Centro Hospitalar Universitário de Coimbra, Microbiology Department, Coimbra, Portugal; ^3^Centro Hospitalar Universitário Lisboa Norte, Microbiology Department, Lisboa, Portugal; ^4^Centro Hospitalar Universitário do Porto, Microbiology Department, Porto, Portugal; ^5^Centro Hospitalar Universitário São João, Microbiology Department, Porto, Portugal; ^6^Centro Hospitalar Universitário Lisboa Central, Microbiology Department, Lisboa, Portugal; ^7^Hospital Garcia de Orta, Microbiology Department, Almada, Portugal; ^8^Centro Hospitalar de Lisboa Ocidental, Microbiology Department, Lisboa, Portugal; ^9^MSD Portugal, Medical Affairs, Paço de Arco, Portugal

**Introduction:**

We study the population structure and resistome of MDR *Enterobacterales* and *Pseudomonas aeruginosa* isolates, C/T-susceptible or -resistant, recovered from low respiratory, intraabdominal and urinary tract infections of ICU patients of 11 Portuguese Hospitals (STEP study, 2016-2017).

**Methods:**

MICs were determined (ISO-broth microdilution, EUCAST) and 30 *Escherichia coli*, 79 *Klebsiella* spp. and 42 *P. aeruginosa* isolates were selected for Whole Genome Sequencing (Illumina-NovaSeq 6000, OGC, UK). Assembly (SPAdes), annotation (Prokka) and *in silico* MLST were performed. Resistance genes were identified (Abricate). *E. coli* phylogroup (ClermonTyping), serotypes (SerotypeFinder) and fimH-types (FimTyper) were determined. *Klebsiella* spp. capsule (K) and LPS (O) serotype were predicted (Kleborate). *P. aeruginosa* O-specific antigen was identified (Blastn).

**Results:**

In *E. coli*, two VIM-2 producers were found (ST131-B2-H30-O25:H4-CTX-M-15 and ST88-C-H39-O22:H4) (C/T-MIC=0.5/4-1/4 mg/L). A KPC-3-ST5463-cladeV-H160-O164:H56 (16/4 mg/L) was also detected. The most frequent ESBL-*E. coli* clone was ST131-B2-H30-O25:H4 (n=14) (0.25/4-1/4 mg/L), related to CTX-M-15 (10/14) or CTX-M-27 (4/14). Ninety-five percent of carbapenemase-*Klebsiella* isolates (n=21) were C/T resistant (2/4->64/4 mg/L). KPC-3-ST13-KL3-O1v2 (n=5), KPC-3-ST5-KL112-O1v1 (n=2), KPC-3-ST231-KL51-O1v2 (n=2), OXA-181-ST17-KL25-O5 (n=2) and OXA-48-ST215-KL16-O1v1 (n=2) were the most frequent clones. A high diversity was found in ESBL (n=41) and non-ESBL (n=17) strains (0.5/4->64/4 mg/L). In *P. aeruginosa*, the most frequent clone was GES-13-ST235-O11 (n=13) (32/4->64/4 mg/L). VIM-2 was found in ST244-O5 and ST179-O6 (>64/4 mg/L) and KPC-3 in ST499-O1 and ST253-O12 (1/4 mg/L). A high clonal diversity was found in non-carbapenemase producers (0.5/4-4/4 mg/L).

**Conclusions:**

Carbapenemase genes are not always associated with C/T resistance in *Enterobacterales* and *P. aeruginosa*, but other mechanisms might be involved.

## P431 Clostridium difficile infection in Lithuania

### D Adukauskienė, R Mickus, A Dambrauskienė

#### Medical Academy, Lithuanian University of Health Sciences, Kaunas, Lithuania

**Introduction:**

*Clostridium difficile* infection (CDI) is the main cause of hospital acquired diarrhoea [1]. The aim of this study was to compare characteristics of CDI during yr 2011 and 2015.

**Methods:**

A retrospective observational study was carried out in Lithuanian University of Health Sciences hospital - the largest teaching facility of tertiary care in country. According to Department of infection control records, patients (pt) with (w.) diarrhoea and the first positive stool test for *C.difficile* toxin A/B were included. Age, *Charlson Comorbidity Index* (CCI) score, profile of hospital department (medical (MD), surgical or ICU) where CDI was diagnosed, type of CDI (healthcare-associated (HA), hospital or community-acquired) and rate of risk factors (RF) have been estimated in both 2011 and 2015. IBM SPSS 23.0; Pearson’s Chi-square, Fisher’s exact tests were used for statistics. P < 0.05 was statistically significant.

**Results:**

In total 7 pt from 2011, 72 from 2015 were enrolled. In 2011 n=4 (57%) pt were ≥65 yr old, in 2015 - n=45 (63%), (p=0.045). In 2011 CCI>5 was estimated in n=6 (86%) pt in comparison of n=46 (64%) in 2015, (p=0.025). In 2011 n=0 (0%) of CDI cases were HA, in 2015 – n=12 (17%), (p=0.01). In 2011 n=5 (71%) of CDI were diagnosed in MD in comparison of n=60 (83%) in 2015, (p=0.01). In 2011 12 weeks prior to CDI n=5 (71%) pt have been admitted to hospitals, n=7 (100%) have been treated w. antibiotics, n=4 (50%) - w. PPIs, n=5 (36%) - w. H2 antagonists, n=3 (43%) - w. immunosupressants in comparison of n=51 (71%), n=69 (96%), n=36 (57%), n=26 (71%) and n=21 (29%) in 2015, respectively, (p>0.05).

**Conclusions:**

Overall rate of CDI cases among in-hospital patients increased ten-fold by yr 2011 and 2015. In 2015, more elderly patients had CDI and severe comorbidities were less frequent in comparison with 2011. In 2015, more cases of CDI were hospital-acquired and have occured in medical departments. Rate of risk factors of CDI remained unchanged.

**References:**

1. McDonald LC et al. Clin Infect Dis 66:e1–48, 2018

## P432 Ceftolozane/tazobactam pharmacokinetics in critically ill patients on continuous venovenous hemodiafiltration

### SM Martinez castro^1^, EB Bárcena Barreto^2^, EU Utrera^2^, JA Carbonell^2^, RF Ferriols^3^, GA Aguilar^2^

#### ^1^Hospital Clinico Universitario, Department of Anesthesiology and Intensive Care, Valencia, Spain; ^2^Hospital Clínico Universitario De Valencia, Servicio De Anestesiología, Reanimación Y Tratamiento Del Dolor, Valencia, Spain; ^3^Hospital Clínico Universitario De Valencia, Pharmacy, Valencia, Spain

**Introduction:**

Ceftolozane/Tazobactam (C/T) is a new antibiotic against MDR Gram-negative bacteria infections, whose target population are the critically ill patients. Even though 2/1 g dose safety administered as a 3-hour-infusion has been already assessed, these patients can be under renal replacement therapy (RRT) and suffer changes in their volume of distribution (Vd) that may affect antibiotic concentrations.

**Methods:**

The objective was to determine concentration reached by 3g C/T (3-hour infusion) in septic patients on RRT (CVVHDF) and interdose behavior. We have used RRT machine PRISMAFLEX with Oxyris filter and M100. HPLC-UV method was used for simultaneous quantification of C/T. Study population consisted of three obese critically ill patients with sepsis, on CVVHDF while receiving 3g C/T every 8 hours. Samples were taken of prefilter, post filter blood and effluent, 30 min before infusion and 1, 2 and 4 hours after the end of it.

**Results:**

We found great interpatient variability with the lowest C-concentration values in the patient with more hemodynamic instability using Oxyris filter. Even though Cmax was less than reported in healthy subjects, we found similar values of AUC and t ½ in comparison with healthy population studies. Cmax of T was also compromised in comparison with values reported in healthy subjects, but with higher AUC and t ½. CVVHDF contributes to C/T clearance. M100 filter showed the least clearance and higher values of AUC and t ½. Extraction rate was similar in all patients and filters (Figure 1).

**Conclusions:**

Cmax achieved may be impaired because of the varying Vd caused by obesity and RRT, but not affecting the antibiotic characteristics and behaviour. We conclude that because of the variety of clinical conditions, C-concentration is compromised particularly in hemodynamically unstable patients. However, the small sample doesn´t let us extrapolate these results. The extended infusion seems to be adequate to achieve the interdose antibiotic concentration.

**Fig. 1 (abstract P432).**
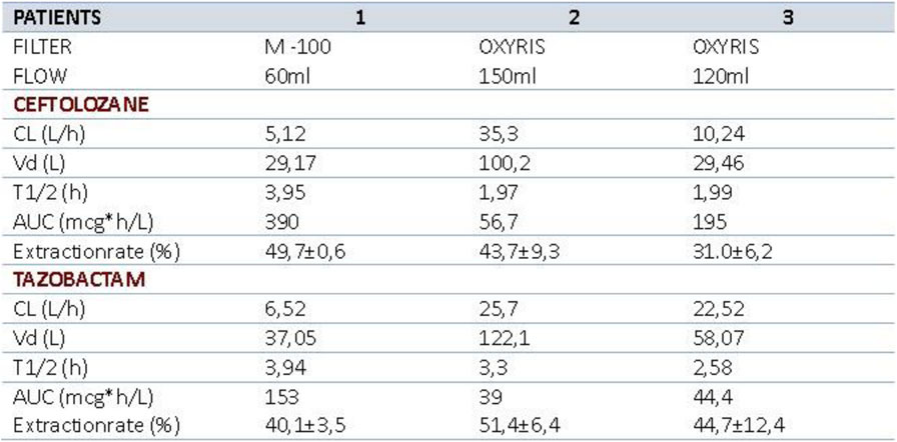
Patient PK/PD measurements

## P433 Alfatorquetenovirus: a new sepsis biomarker?

### SM Martinez castro^1^, N Segura Marín^2^, E Albert^3^, D Navarro^3^, P Pardo^2^, Ja Carbonell^2^, E Carbonell^4^, G Aguilar^2^

#### ^1^Hospital Clinico Universitario, Department Of Anesthesiology And Intensive Care, Valencia, Spain; ^2^Hospital Clínico Universitario De Valencia, Servicio De Anestesiología, Reanimación Y Tratamiento Del Dolor, Valencia, Spain; ^3^Hospital Clínico Universitario De Valencia, Microbiology Department, Valencia, Spain; ^4^Hospital Clínico Universitario De Valencia, Intensive Care Unit, Valencia, Spain

**Introduction:**

The use of biomarkers in sepsis is useful for early diagnosis and prognosis. The desired marker should be sensitive, specific, fast and accurate. Procalcitonin (PCT) measurement is approved by the FDA even its efficacy is still under question. The determination of alfatorquetenovirus (TTV) could be a useful marker [1].

**Methods:**

We analyzed 55 samples from 23 patients admitted to ICU with clinical suspicion of sepsis. Analytical data of C-reactive protein (CRP), neutrophils and procalcitonin were collected. The SOFA and APACHE II scales were calculated and patients stratified according to these values ​​in good and poor prognosis. TTV quantitative determination was carried by using a quantitative CRP^2^. We calculated area under the curve (AUC) of TTV plasma levels ​​as a function of time. The statistical analysis involved U-Mann-Whitney and Spearman test, using Chi^2^ for qualitative variables.

**Results:**

Results showed a not significant (NS) inverse relationship between the TTV AUC and the patient proinflammatory level. A tendency (NS) was found between poor prognosis and the PCT median values and CRP being higher in the poor prognosis.group. A trend showed lower TTV DNA count related to worse prognosis. An inverse relationship was found between PCT and CRP values ​​and the TTV copies /ml plasma, NS correlation in the case of PCT. There was a clear trend between the neutrophils´ expansion and the regression line slope, obtained between TTV loads in the first two study steps.

These results indicate a possible relationship between TTV DNA count and immunological alteration.

**Conclusions:**

The TTV quantitative determination could be useful as a proinflammatory marker in sepsis, with some benefits: low cost, easy determination and good correlation with immune system functionalit. It will be necessary to perform a larger study to check our hypothesis and to establish a TTV level threshold that may allow to anticípate the disease prognosis.

**References:**

1. Focosi D et al. Clin Microbiol Infect. 22:589-93, 2016

## P434 Identifying synergistically nephrotoxic antimicrobial regimens in patients with sepsis: two nationwide multicenter registries in Japan

### N Meguro^1^, J Yoshimura^1^, K Yamakawa^2^, Y Umemura^2^, S Fujimi^1^, H Ogura^2^, T Abe^2^, S Gando^2^, Y Otomo^2^

#### ^1^Division of Trauma and Surgical Critical Care, Osaka General Medical Center, emergency, Osaka, Japan; ^2^Japanese Association for Acute Medicine Sepsis Prognostication in Intensive Care Unit and Emergency Room (SPICE) study group, emergency, Tokyo, Japan

**Introduction:**

Acute kidney injury (AKI) is a serious complication in sepsis and associated with high morbidity and mortality. The combination antimicrobial regimens with vancomycin (VCM) and broad-spectrum beta-lactams (BSBL), such as piperacillin tazobactam and cefepime, have been identified as potentially nephrotoxic combinations, but existing studies have not provided sufficient evidence. The aim of this study was to evaluate detailed association between the combination antimicrobial therapy and the risk of AKI in septic patients.

**Methods:**

This investigation was a post hoc analysis of 2 prospective nationwide cohorts enrolling consecutive adult patients with sepsis in intensive care units in Japan. In this study, progression of AKI was defined as one or more elevation of renal sub-score in Sequential Organ Failure Assessment score from day 1 to day 4. We regarded anti-pseudomonal penicillins, fourth generation cephalosporines, and carbapenems as BSBL. Multivariable logistic regression analysis including a two-way interaction term (VCM x BSBL) was performed to assess the add-on effects of each antimicrobial agent on the progression of AKI.

**Results:**

The final study cohort comprised 1837 patients with sepsis. Among them, 45 received VCM without BSBL, 1055 received BSBL without VCM, 249 received both VCM and BSBL, and 488 received other type of antimicrobials. The administration of VCM was associated with an increased risk of AKI in patients with BSBL [odds ratio (OR), 1.57 (0.96-2.57); p=0.072]. However, the tendency was not evident in patients without BSBL [OR, 0.23 (0.03-1.56); p=0.133]. The interaction effect on the progression of AKI between VCM and BSBL were statistically significant (p for interaction=0.038).

**Conclusions:**

The regression model including two-way interaction term suggested that the combination of VCM and BSBL might synergistically increase the risk of AKI in patients with sepsis.

## P435 Epidemiology of nosocomial infections caused by carbapenem resistance Klebsiella pneumoniae producing KPC, NDM and OXA-48 carbapenemases in burn ICU

### V Bagin, M Astafyeva, N Nevskaya, E Lukina, I Korobko, V Rudnov

#### City Clinical Hospital No 40, ICU, Yekaterinburg, Russia

**Introduction:**

Increasing resistance to carbapenems due to carbapenemase production – one of main actual problems of antibacterial resistance in burn ICU. Production of several types of carbapenemases (KPC, NDM and OXA-48) is common in K. pneumoniae strains. Carbapemenase production is a marker of extreme antibacterial resistance. The aim of our study was to investigate the epidemiology of nosocomial infections caused by producing KPC, NDM and OXA-48 K. pneumonia strains in burn ICU.

**Methods:**

Total of 26 patients with nosocomial infections caused by 26 carbapenem resistance strains of K. pneumoniae were included in the study, from whom 3 had lower respiratory tract infection, 23 had skin and skin structure infection. Initial identification of isolates was performed in laboratory by automatic microbiological analyzer. For all of K. pneumoniae isolates presence of bla_NDM_, bla_OXA-48_ and bla_KPC_ – genes were examined by PCR method.

**Results:**

Baseline characteristics of patients: Me (IQR) of age – 50 (39; 64) years, Me (IQR) of TBSA – 40 (29; 52) percent, Me (IQR) of ICU LOS – 30 (21; 35) days. Inhalation injury was diagnosed in 10 (38.4%) patients. Total of 11 patients died, mortality rate was 42.3%. All patients were diagnosed with nosocomial infection caused by K. pneumoniae. From 26 K. pneumonia strains 1 (3.8%) were found to be producing KPC, 3 (11.5%) – producing NDM and 20 (76.9%) –producing OXA48. Only 5 (19.2%) carbapenem resistance K. pneumoniae isolates were not producing carbapenemases. From 20 patients infected by OXA48 producing K. pneumoniae 20 patients died, mortality rate was 40%. From 23 patients infected by OXA48 or NDM producing K. pneumoniae 10 patients died, mortality rate was 43.5%. From 5 patients infected by non-carbapenemase producing K. pneumonia no one died.

**Conclusions:**

Carbapenemase producing strains are widely spread among carbapenem resistance strains of K. pneumoniae in burn ICU. Mortality of patients infected by producing OXA48 or NDM K. pneumoniae strains reaches 43.5%.

## P436 Vancomycin adsorption during in vitro model of hemoperfusion with HA330 cartridge

### I Godi^1^, A Lorenzin^2^, S De Rosa^3^, A Sandini^4^, M De Cal^5^, P Navalesi^1^, C Ronco^5^

#### ^1^Department of Medicine - DIMED, Section of Anesthesiology and Intensive Care, PADUA, Italy; ^2^San Bortolo Hospital, International Renal Research Institute of Vicenza, Vicenza, Italy; ^3^San Bortolo Hospital, Section of Anesthesiology and Intensive Care, Vicenza, Italy; ^4^San Bortolo Hospital, Department of Trasfusional Medicine, Vicenza, Italy; ^5^San Bortolo Hospital, Department of Nephrology, Dialysis and Transplantation, Vicenza, Italy

**Introduction:**

The rationale for blood purification as adjunctive therapy during sepsis involved the capacity in removing endogenous and exogenous toxins, but currently no recommendations exists [1]. A critical point may be the potential interaction with antimicrobial therapy, which remains the mainstay of sepsis treatment. The aim of our study was to investigate the vancomycin (VAN) removal during blood purification using an *in vitro* model of hemoperfusion (HP) with HA330 cartridge (Jafron, Zhuhai City, China), most widely used in China and actually available in Europe.

**Methods:**

This is an experimental study. Three independent experiments were performed: we injected 250 mg of VAN in 500ml of whole blood from healthy donors (experiment 1 and 2) or in 500ml of balanced solution (experiment 3) in order to assess membrane saturation. A closed-circuit (blood flow of 250ml/min) simulating HP ran using HA330. Samples were collected from arterial line at 0, 5, 10, 15, 30, 45, 60, 90, 120 minutes; VAN plasma concentrations were measured and removal was evaluated using mass balance analysis. Differences in mass removal was assessed using Kruskal-Wallis test.

**Results:**

Figure 1 shows VAN mass at each timepoints. We observed no difference between in blood and in balanced solution experiments (p-value>0.05), suggesting that the adsorptive mechanism wasn't primarily mediated by plasma protein. HA330 was saturated after adsorption of a total of 280.28*±12.33* mg of VAN. The adsorptive kinetics showed an exponential reduction of VAN mass that reached a plateau after 30 minutes of circulation.

**Conclusions:**

In our study, simulating in vivo conditions of HP using HA330 during sepsis, a rapid and clinically relevant removal of VAN has been shown. After 2 hours of HP, we suggest to assess VAN plasma concentration and a loading dose of VAN should be considered. However, not knowing the potential interactions with other drugs, further in vivo studies are warranted to confirm these findings.

**References:**

1. Rhodes A et al. Crit Care Med 45:486-552. 2017

**Fig. 1 (abstract 436).**
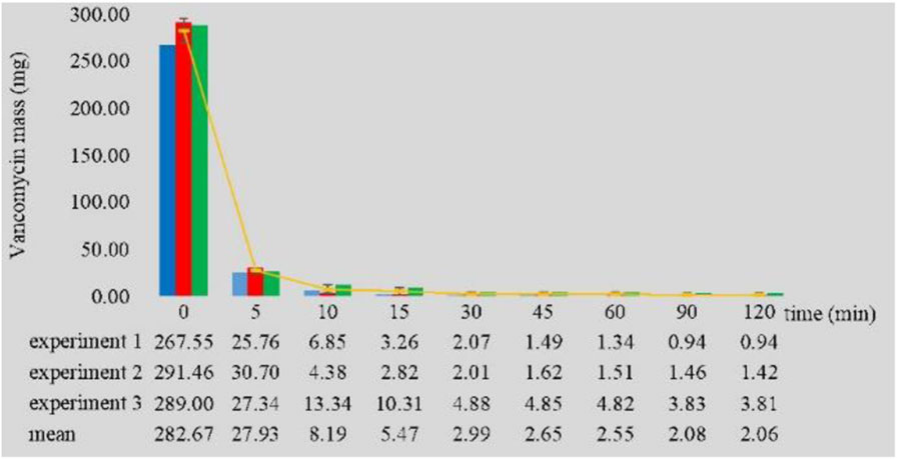
Vancomycin mass removal over 120 minutes of hemoperfusion using HA330. Bars refer to vancomycin mass (mg): blue (experiment 1) and red (experiment2) bars using blood while green (experiment 3) bar using balanced solution. Yellow dashes are mean mass values of the three experiments (with standard deviations) and yellow line represents the reduction curve over time

## P437 The value of routine blood-borne virus (BBV) testing in the intensive care unit (ICU)

### YY Tan^1^, M Booth^2^

#### ^1^University of Glasgow, Glasgow, United Kingdom; ^2^Glasgow Royal Infirmary, Anaesthesia And Intensive Care, Glasgow, United Kingdom

**Introduction:**

The aim of this study is to determine if routine BBV testing in the ICU contributes to the discovery of undiagnosed BBV infections. ICU patients may require renal replacement therapy (RRT). Sharing RRT equipment carries a risk of BBV transmission, which mainly relates to Hepatitis B (HBV), Hepatitis C (HCV) and HIV. Since 2012, all Glasgow Royal Infirmary ICU patients undergo routine BBV screening, with RRT machines allocated for patients with specific BBV statuses. Routine BBV testing is beneficial to both the individual and society. HCV is a pertinent health issue in Scotland. The Scottish government aims to eliminate HCV by 2030 and is researching innovative and cost-effective methods to identify undiagnosed infections.

**Methods:**

This single-centre retrospective observational study examined prospectively collected clinical data from 1069 ICU admissions. Proportions were compared using a two-proportion z-test and a logistic regression model was carried out to determine if deprivation quintile was independently associated with the seroprevalence of BBVs.

**Results:**

The BBV seroprevalence in the cohort studied: 0.45% (HBV), 11.7% (HCV), 0.91% (HIV). The seroprevalence of HBV in the cohort studied was similar to that of Scotland (p=0.11), but the seroprevalence of HCV (p<0.001) and HIV (p=0.01) were statistically significantly higher than that of Scotland. Due to the small number of reactive test results for HBV and HIV, the relationship between deprivation and BBV seroprevalence was explored for HCV only. The only independent variable associated with a reactive anti-HCV test result was “current or previous illicit drug use” (adjusted odds ratio of 40.2; 95% confidence interval of 21.1-76.4; p<0.001).

**Conclusions:**

This study shows that routine BBV testing in the ICU is useful in discovering new BBV infections. This is the first observational study focusing on the value of routine BBV testing in an ICU setting to our knowledge.

## P438 Assessment of the clinical significance of bacteremia causative pathogens in ICU patients

### M Dementienko^1^, V Gusarov^1^, D Shilkin^1^, R Amaeva^1^, E Gricenko^1^, M Luzin^1^, I Plaksin^1^, D Menshikov^1^, D Kamyshova^2^, M Zamyatin^1^

#### ^1^N. Pirogov National Medical Surgical Center, ICU, Moscow, Russia; ^2^N. Pirogov National Medical Surgical Center, Pharmakologist, Moscow, Russia

**Introduction:**

It was shown that bacteremia in ICU patient with infection of different localization becomes an independent risk factor for adverse outcome, but it remains unclear how clinical outcome of bacteremia depends on causative pathogen and its features. The aim of the study was to compare mortality rate and clinical outcomes of ICU patients with bacteremia complicated infections according to pathogen and its properties.

**Methods:**

We conducted a retrospective cohort study from January 2011 to December 2017 in four general ICUs of a multidisciplinary hospital. 7560 patients were screened, 374 patients with infection complicated by bacteremia were included in the study. The average age was 61±14.5 years, male 63.1%, APACHE2 score 16 (11-21), SOFA 5 (2-7). According to the causative agent the cohort was divided into subgroups: ESKAPE (136 patients), MDR *Pseudomonas aeruginosa* (18 patients), CPR *Klebsiella pneumoniae* (32 patients), Candida spp. (21 patients). The comparison subgroup consisted of 217 patients with bacteremia caused by non-ESCAPE pathogens. We evaluated the days of mechanical ventilation, duration of antibiotic therapy (AMT), ICU length of stay (LOS), hospital LOS and mortality (Table 1).

**Results:**

Mortality in patients with bacteremia caused by non-ESKAPE pathogens was 23.5%, *Candida* spp. – 42.9% (p=0.052), in MDR *Ps.aeruginosa* subgroup – 44.4% (p=0.084), ESKAPE – 50.7% (p<0.0001), CPR *Kl.pneumoniae –* 68.75% (p<0.0001).

**Conclusions:**

Bacteremia caused by CPR Klebsiella pneumoniae or all ESKAPE group pathogens, as well as candidemia, caused by *Candida* spp. significantly worsens the clinical outcomes and increases mortality of ICU patients.

**Table 1 (abstract P438).** Results. * p-value versus non-ESKAPE

Subgroup	Mechanical ventilation, days*	AMT, days*	ICU LOS, days*	Hospital LOS, days*
non-ESKAPE	5 (0-15)	11.5 (7-20)	12 (5-22)	30 (17-55)
ESKAPE	7.5 (2-24) р=0.002	12 (8-21) р=0.162	15 (7-28.5) р=0.019	32 (20-57) р=0.247
CPR Kl.pneumoniae	7.5 (2-26) р=0.043	12 (9-28) р=0.286	15 (8-27) р=0.169	31 (21.5-58) р=0.481
MDR Ps.aeruginosa	11 (6-27) р=0.023	14 (8-21) р=0.279	18 (8-30) р=0.099	32.5 (15-49) р=0.717
Candida spp.	12.5 (2-41) р=0.023	-	19.5 (9-41) р=0.03	44.5 (18-75) р=0.263

## P439 Assessing the volume of blood taken for blood culture and culture positivity – do we need to take less blood?

### TM Mann^1^, M Salim^2^, S Haider^2^, AH Mayo^1^, KK Kaye^2^

#### ^1^Assuta medical center, Department of Intensive Care, Ashdod, Israel; ^2^Detroit Medical Center, Department of Medicine, Detroit, United States

**Introduction:**

It is commonly accepted that larger blood culture (BC) volumes (BCV) increase the yield of true positive cultures, and optimally 20 cc of blood should be obtained per set (2 bottles). Only scarce data exists on the matter of optimal BCV. It is unknown what is the minimal volume that is acceptable for BC. The objective of this study was to determine the association between BCV and the rate of positive BC.

**Methods:**

Blood taken for cultures in BD BACTEC Plus Aerobic/F negative bottles was collected from ICUs and acute care floors at 8 hospitals at the DMC over 6 months. Blood volume was estimated automatically from blood background signal data in the BD BACTEC FX instrument. Cultures were analyzed for each bottle. Data was summarized for every month as the average volume and number of cultures taken and rate of positive BC for every unit. Units were classified according to unit type (ICU, Medicine, Surgery, Mixed, Emergency Department (ED), Organ/BMT or "other" which did not fit the previous categories) and analyzed as a group.

**Results:**

A total of 23795 cultures were taken in 84 units. There is a positive association between BV and positive BC rate for ED and “other” units (IRR=1.27, p=0.006 for the ED, IRR=5.00, p<0.001 for “other” unit). All other units had no association between BV and positive BC rate (Figure 1). Secondary analysis, excluding Pediatric units, gave very similar results. When comparing BV between unit types, the ED and “other” unit had significantly lower BV (2.4 ml in the ED and 3.5 ml in “other” unit compared to 4.9 ml in the ICU, 4.7 ml in surgery, 4.2 ml in mixed and 7.7 ml in BMT).

**Conclusions:**

The correlation between BV and positive BC rate is probably limited to units taking very low BV for cultures. Units taking volumes above 4 ml show no improvement in positive BC rate when higher volumes are taken. Better prospective studies should be done to further establish the minimal BCV needed and spare unnecessary blood loss to hospitalized patients without compromising BC yield.

**Fig. 1 (abstract P439).**
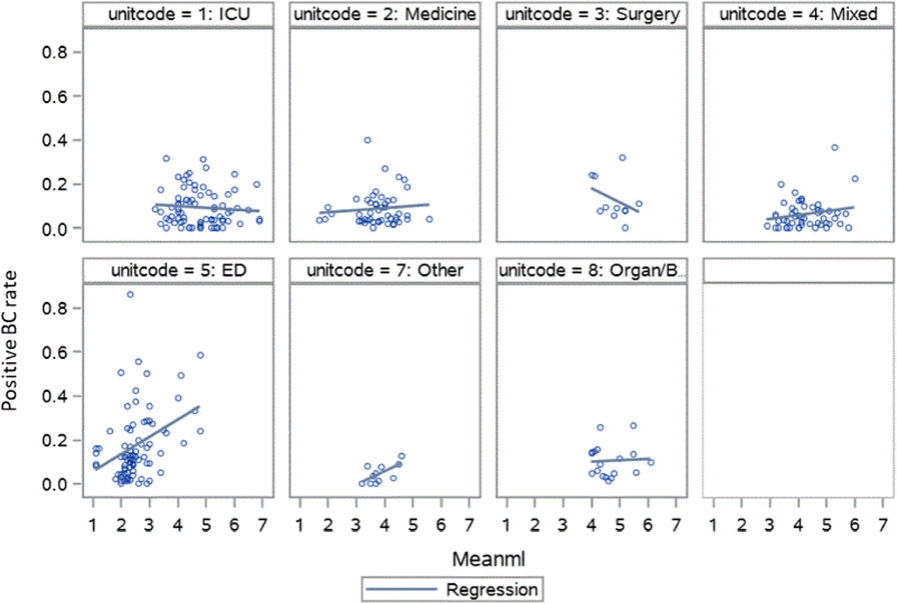
Correlation of blood culture positivity rate with blood culture volume by unit type

## P440 De-escalating antibiotics in sepsis with the use of T2MR in a 35-bed Greek university ICU

### C Vrettou, E Douka, I Papachatzakis, K Sarri, E Gavrielatou, E Mizi, S Zakynthinos

#### 1st ICU Department, University of Athens, Evangelismos General Hospital, ICU, Athens, Greece

**Introduction:**

In septic patients, the early use of appropriate empiric antibiotic therapy reduces morbidity and mortality. De-escalation refers to narrowing the broad-spectrum antibiotics once the pathogen and sensitivities are known. T2 Magnetic Resonance (T2MR) is a novel method of detecting ESKAPE pathogens. We aim at investigating if using T2MR technology can expedite de-escalation of broad spectrum antibiotics.

**Methods:**

This is a prospective observational study conducted in our 35-bed university ICU. Inclusion criteria were critically ill patients age>18 y.o., with newly diagnosed sepsis and clinical suspicion of ESKAPE bloodstream infection. A sample for T2MR and a blood culture (BC) sample were collected simultaneously from the patients enrolled. The T2MR Bacteria panel test was run according to the manufacturer’s guidelines and the BCs were processed according to the hospital standard procedures. We recorded clinical data and administered antibiotics.

**Results:**

26 patients were included in the study. Mean time to culture positivity was 84 hours while mean time to T2MR result was 3.5 hours. In 20 patients the results of T2MR were in concordance with the BCs. In the remaining 6 cases, the BCs were negative while the T2 MR detected one or more ESKAPE pathogens. There were no false negative results. De-escalation in at least one drug was applied to 8 patients (30.8%). No escalation was applied to 15 patients (57.7%) and antibiotic escalation in 3 (11.5%).

**Conclusions:**

T2MR provides a quicker detection time that could shorten the time to targeted therapy. In our population this corresponded to early (within 6-12h) antibiotic de-escalation in approximately 1/3 of the included patients.

## P441 Antibiotic stewardship in ICU. A single experience

### L Forcelledo^1^, E García-Prieto^1^, L López-Amor^1^, E Salgado^1^, J Fernández Dominguez^2^, M Alaguero^3^, E García-Carús^4^, M Telenti^5^, L Cofiño^1^, D Escudero^1^

#### ^1^Hospital Universitario Central de Asturias, Intensive Care Department, Oviedo, Spain; ^2^Hospital Universitario Central de Asturias, Microbiology department, Oviedo, Spain; ^3^Hospital Universitario Central de Asturias, Pharmacology department, Oviedo, Spain; ^4^Hospital Universitario Central de Asturias, Infectious Diseases Unit, Oviedo, Spain, ^5^Hospital Universitario Central de Asturias, Infectious Disease Unit, Oviedo, Spain

**Introduction:**

The increasing antibiotic resistance in microorganisms urged interventions such as the antibiotic stewardship programs in ICU focused on reducing the inappropriate use of antibiotics by improving the antibiotic selection, the dosage, administration route and length as well as improving clinical outcomes and reducing antibiotic resistance.

**Methods:**

Retrospective study where antibiotic consumption was analysed and measured in days of therapy (DOTs) between 2015 and 2018 in a medical-surgical ICU of a university hospital where a multimodal educational program was established. Specific training in infectious diseases in critically ill patients, periodic clinical and formative sessions were performed for ICU staff and specific leaders within the ICU staff designated.

**Results:**

4128 patients were admitted to ICU. There was a reduction of 20,5% in DOTs (Figure 1), reduction in antimicrobial resistance rates (14,36 in 2015, 7,4 in 2018 [days of resistant microorganism/1000 patient-days]) without an impact in ICU global mortality (19,7% in 2015, 19,4% in 2018). The resistant bacteria registered were *Acinetobacter baumannii, S. aureus* MR, BLEE and carbapenemase-producing Enterobacteriaceae, *Pseudomonas aeruginosa* MR and *Clostridium difficil*e. The safe in antimicrobial consumption was 391500€ (70% reduction). The ICU stay decreased from 8,2 days (2015) to 6,9 (2018), with no variation in mean APACHE II (17,8). The bigger decrease in antibiotic consumption was in Colistin related to the reduction in resistance bacteria, in special *Acinetobacter baumannii*, in linezolid and in piperacilin/tazobactam, even more remarkable in 2018 due to shortage of supplies which meant an increase in meropenem.

**Conclusions:**

The application of an antibiotic stewardship program in ICU succeeded in reducing antibiotic consumption, antibiotic resistance and costs without an impact in clinical outcomes like mortality or ICU stay.

**Fig. 1 (abstract P441).**
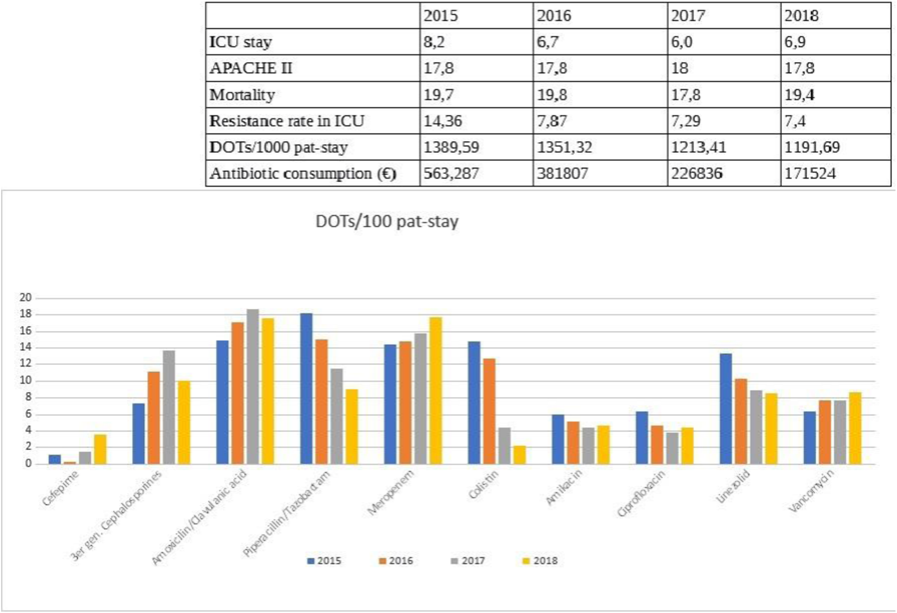
Antibiotic consumption and general epidemiology in ICU

## P442 Clinical outcomes of isavuconazole versus voriconazole for the primary treatment of invasive aspergillosis: subset analysis of Indian data from SECURE trial

### P Kundu, S Kamat, A Mane

#### Pfizer Limited, Medical Affairs, Mumbai, India

**Introduction:**

The SECURE trial was designed to compare the safety and efficacy of Isavuconazole (A) versus Voriconazole (V) for primary treatment of invasive mould disease caused by *Aspergillus* and other filamentous fungi. The present analysis is aimed at comparing the Indian subset of patients with that of the overall trial population and to ascertain any similarity or difference in the primary efficacy endpoint and safety/tolerability in these two groups.

**Methods:**

In SECURE trial, 258 patients in one group received (I) & another 258 patients received (V). The Indian subset had 29 patients. We have done a qualitative analysis as the sample size of the Indian subset was small. Non-inferiority of (I) to (V) in terms of all cause mortality from first dose to day 42 was assessed in overall patients. The treatment difference between (I) and (V) group in the Indian subset of patients was analyzed. Proportion of patients who had to discontinue treatment due to TEAEs was analyzed.

**Results:**

The all-cause mortality in the overall trial population met non-inferiority margin (Table 1). In the Indian subset, it was higher for (I) than (V). There was a lower incidence of ocular, hepatobiliary, skin & subcutaneous tissue disorders in the (I) treated patients (see Table 1). In Indian subset, the above adverse events were less in the (I) group, but statistical inference could not be done due to small sample size. However, similar trend of less number of patients discontinuing therapy due to TEAEs in the (I) treated patients was seen in the overall patients & the Indian subset.

**Conclusions:**

The all-cause mortality in the Indian subset was higher in the (I) patients. A trend similar to the overall population regarding safety parameters favoring (I) was seen in the Indian patients. Considering the significantly higher prevalence of IA in India, suitably powered study design is necessary to draw definitive conclusions on the non-inferior efficacy & better safety & tolerability of (I) over (V) in patients of IA.

**Table 1 (abstract P442).** Comparison of primary efficacy endpoint and safety parameters between overall trial population and Indian subset of patients

Parameters	Overall Population (n=258)	Overall Population (n=258)	Indian Subset (n=12)	Indian Subset (n=17)
All- cause mortality (Day 42)	48 (19)	52 (20)	5 (41.6)	5 (29.4)
Adjusted Treatment Difference	-1%		12.2%	
TEAE leading to treatment discontinuation	37 (14)	59 (23)	2 (16.7)	4 (23.5)
Ocular toxicity	39 (15)	69 (27)	0	2 (11.8)
Skin and Subcutaneous Tissue disorder	86 (33)	110 (42)	1 (8.3)	1 (5.9)
Hepatobiliary disorder	23 (9)	42 (16)	0	1 (5.9)

## P443 Ceftazidim/avibactam on OXA-23 A. baumannii ventilator associated pneumonia – does it make any sense?

### C Cobilinschi, M Țigliș, A Băetu, AM Cotae, OM Melente, M Costache, T Hurmuzache, E Ciobanu, IM Grințescu

#### Clinical Emergency Hospital Bucharest, Anesthesiology and Intensive Care, Bucharest, Romania

**Introduction:**

Ventilator-associated pneumonia (VAP) is one of the most frequent healthcare-associated infections, correlated with increased mortality,extended hospital stay and prolonged mechanical ventilation. Considering the latest outbreak of multiresistant *A. baumannii* infections in the critically ill patients with VAP, there is a growing concern regarding challenges of the antibiotherapy in these patients. Although ceftazidim-avibactam is considered to have limited effects on *A. baumannii,* it is reported to have a synergic activity in combination with other antibiotics.

**Methods:**

We performed a retrospective, observational study which included 24 ICU patients diagnosed with VAP(CPIS > 6). OXA 23 *A. baumannii* was isolated from the tracheal secretions using a rapid molecular diagnostic platform(Unyvero A50 System). Patients were divided in two groups according to the antibiotherapy:group A Meropenem + Colistin and group B Meropenem + Colistin + Ceftazidim-avibactam.Statistical analysis was performed using GraphPad6 applying T-test and Kaplan-Meier curves, having the in-hospital mortality as primary outcome and days of mechanical ventilation and hospital stay as secondary outcomes.

**Results:**

Mean age(y.o) in group A was 46 and 52 in group B and in both groups mean Charlson comorbidity index was 3 points. Survival percent was higher in the group treated with Ceftazidim-avibactam (67 % vs 57 %, p = 0.08)-(Fig. 1). Length of stay was significantly decreased in group B (26.5 days vs 43 days in group A, p = 0.046). Number of days under mechanical ventilation was also decreased in the ceftazidim-avibactam group (19 vs 22) but the data was not statistically significant.

**Conclusions:**

In light of the important thread of multiresistant *A. baumannii* and the lack of therapeutic measures, the synergistic activity of Ceftazidim-avibactam use in combination with other antibiotics may be a promising approach to lower the mortality and hospitalization in critically ill patients diagnosed with VAP.

**Fig. 1 (abstract P443).**
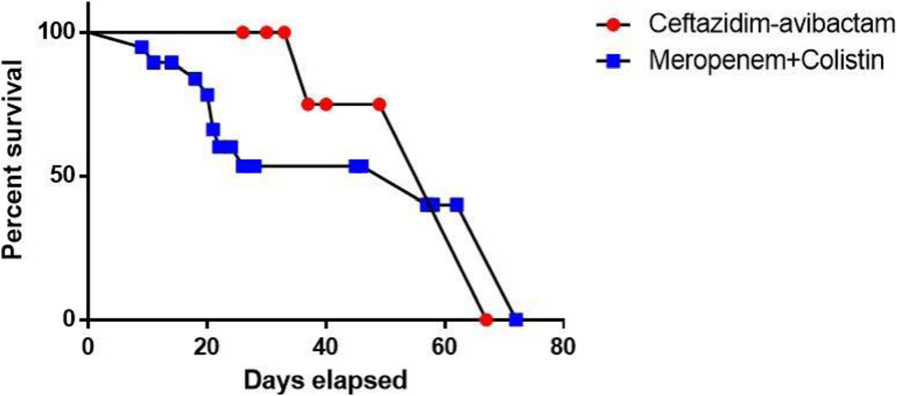
Survival proportions : Survival of two groups. Blue - Group A, Red - Group B

## P444 Impact of patient colonization on admission to intensive care on 28 and 90 days mortality

### G Dabar^1^, C Harmouch^2^, E Nasser Ayoub^3^, Y Habli^4^, G Sleilaty^5^, J Choucair^6^

#### ^1^Hotel Dieu de France Hospital and Saint Joseph University, Pulmonary and Critical Care, Beirut, Lebanon; ^2^Hotel Dieu de France Hospital and Saint Joseph University, Beirut, Lebanon; ^3^Hotel Dieu de France Hospital and Saint Joseph University, Anesthesia and Critical Care, Beirut, Lebanon; ^4^Hotel Dieu de France Hospital and Saint Joseph University, Pulmonary and Critical Care Section, Beirut, Lebanon; ^5^Hotel Dieu de France Hospital and Saint Joseph University, Biostatistics section, Beirut, Lebanon; ^6^Hotel Dieu de France Hospital and Saint Joseph University, Infectious Diseases, Beirut, Lebanon

**Introduction:**

Infections caused by Multi Resistant Bacteria are a major health problem, especially in ICUs, and it may be associated with high mortality rates.   Colonization precedes infection in most instances; therefore it may be a marker of a poor outcome. We tried to determine the impact of colonization on mortality at 28 and 90 days in a population of patients admitted to one medical and one surgical ICU in the same institution.

**Methods:**

Medical records review over three years 2016-2018 of all patients admitted to one surgical et one medical ICU at Hotel Dieu de France Hospital staying more than 24h. Colonization to resistant bacteria was defined as MRSA, ESBL, MDR, and VRE. All patient received a nasal and rectal screen on ICU admission, in intubated patients tracheal aspirate was considered as colonization in the absence of clinical respiratory tract infection. Demographics, APACHE, SOFA, immunosupression, Charleston comorbidity index, length of stay, mechanical ventilation, hospitalization and antibiotic use in the previous 3 month were collected. Mortality at 28 and 90 days was assessed through medical records or phone call. Pearson Chi-Square was calculated for the association of colonization and mortality at 28 and 90 days, and subsequently odd ratio was estimated.

**Results:**

1337 patients fulfilled our study criteria. 413 0r 53.3% were colonized, 28 patients or 2.8%  of the population had MRSA on nasal screen. Rectal swab identified 44.5 % of ESBL, 3% of MDR and 4.8% of VRE. Mortality at 28 and 90 days were 19% and 30% respectively. No significant difference was found between either nasal or rectal or any colonization and mortality at 28 and 90 days. Odd ratio for 28 days mortality in colonized patient was calculated to 0.98 (CI:0.74-1.29) and for 90 days mortality 1.09 (CI:0.85-1.39).

**Conclusions:**

In this study we concluded that colonization with resistant bacteria  is not a risk factor for mortality at 28 and 90 days.

## P445 Continuous infusion vancomycin: A better option in critical care?

### M Murphy^1^, Z Alrifai^2^

#### ^1^Nottingham University Hospitals, Critical Care, Nottingham, United Kingdom; ^2^Nottingham University Hospitals, Intensive Care Medicine, Nottingham, United Kingdom

**Introduction:**

Critically unwell patients have been observed to respond unpredictably to traditional intermittent dosing (ID) schedules of vancomycin, likely due to the complex physiological derangements caused by critical illness. Continuous infusion (CI) of vancomycin has been suggested to overcome such problems by allowing more regular therapeutic drug monitoring and subsequent effective dose titration [1].

**Methods:**

This study conducted at a tertiary intensive care unit, reports our experience following implementation of a continuous vancomycin infusion protocol. Prospective data was collected over two consecuative periods of three months, initially capturing plasma levels for ID (target level of 15-20mg/L) followed by reviewing plasma concentration levels in a CI protocol (target level of 20-25 mg/L). Patients recieving renal replacement therapy were excluded.

**Results:**

A total of 22 intermittent vancomycin prescriptions were administered and dosing levels observed. In the three month CI period, 26 patients received CI vancomycin and levels subsequently checked. The CI protocol resulted in increased blood sampling (107 samples in CI group vs. 73 samples in ID cohort). Two non serious incidents were reported in the CI cohort relating to preparation of vancomycin. Both groups had a comparable median time to therapeutic range (48 hours). However, CI vancomycin group had a greater proportion of first samples outside the desired therapeutic range (70%vs 36%) (Figure 1). As the therapy continued, CI vancomycin demonstrated a greater propensity towards consistent therapeutic levels than that observed with ID. 83% of patients on a CI regime achieve the desired target levels compared to 77% in the ID cohort

**Conclusions:**

Continuous infusion vancomycin protocol is a safe, acceptable and effective alternative to intermittent dosing of vancomycin in critical care.

**References:**

1. Roberts JA et al Antimicrob Agents Chemother 55:2704-9, 2011

**Fig. 1 (abstract P445).**
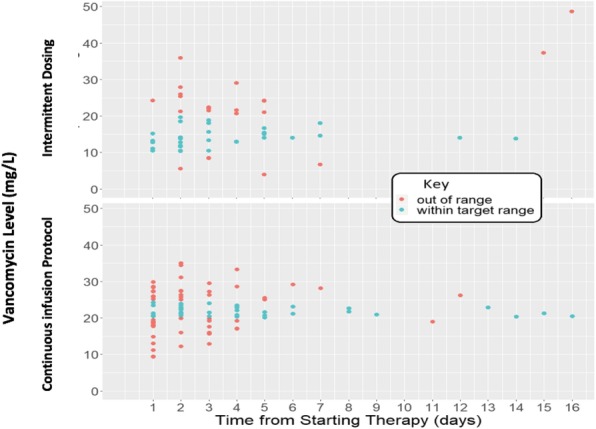
Scatter plot of vancomycin plasma levels during intermittent and continuous infusion protocol periods

## P446 Prospective cohort study of impact of BAL Biofire® Filmarray® pneumonia panel on microbial diagnosis and antibiotic prescription in ICU

### M Sircar^1^, O Jha^1^, J Singh^1^, S Yadav^1^, R Kaur^2^

#### ^1^Fortis Hospital, Pulmonology and Critical Care, Noida, UP, India; ^2^Fortis Hospital, Microbiology, SRL Diagnostics laboratory, Noida, UP, India

**Introduction:**

Biofire® Filmarray® multiplex PCR pneumonia panel (BFPCR) can shorten microbial detection time.

**Methods:**

Six months comparison of time to microbial diagnosis and treatment changes in pneumonia cases, between in-house bronchoalveolar lavage (BAL) stains and cultures and BFPCR done by remote laboratories [Dr. Dangs Lab, New Delhi (Lab 1) and SRL Mumbai (Lab 2)].

**Results:**

Thirty-four BAL samples obtained from 32 patients were sent for stains, culture and BFPCR. The mean time to results was less for BFPCR (12.9±14.5 hrs) (3.3±1.1 hrs for Lab 1 and 30.8±9.6 hrs for Lab 2) in comparison to culture (48±5 hrs) (p<0.01). Lab 1 time was less than reporting time of stains (4.0±1.8 hrs; p=0.04). BFPCR was positive in 31(91.2%) samples and reported higher (p<0.01) yield of microbes (77 vs 25) and bacterial resistances (61 vs. 10) than cultures (Fig. 1). It was positive for single or multiple microbes in 9(26.5%) and 22(64.7%) samples respectively. Single or multiple resistance genes were detected in 5(25%) and 20(80%) samples respectively. BFPCR was positive only for bacteria in 13(38.2%), virus in 2(5.9%) and for both in 16(47.1%) cases. Influenza A was found in 10(29.4%) cases. The most common organisms in community and hospital acquired pneumonia were Streptococcus pneumoniae (4/12) and A. baumannii (10/22) respectively. Bacterial cultures were concordant with BFPCR in 11/11 (100%) of positive cases. Decisions to change antibiotics could be taken earlier based on BFPCR (p< 0.001) than if were based solely on cultures – both in culture positive (9.7±14.3 vs 50.03±6.0 hrs) and negative cases (14.7±14.9 vs 48.0+4.3 hrs) where antibiotics would have remained unchanged. Based on BFPCR antibiotics were escalated in 17(50%) patients and teicoplanin (11/19) was most often stopped.

**Conclusions:**

BAL BFPCR were obtained significantly earlier, identified more organisms and bacterial resistance than culture reports and lead to more frequent and earlier antibiotic changes.

**Fig. 1 (abstract P446).**
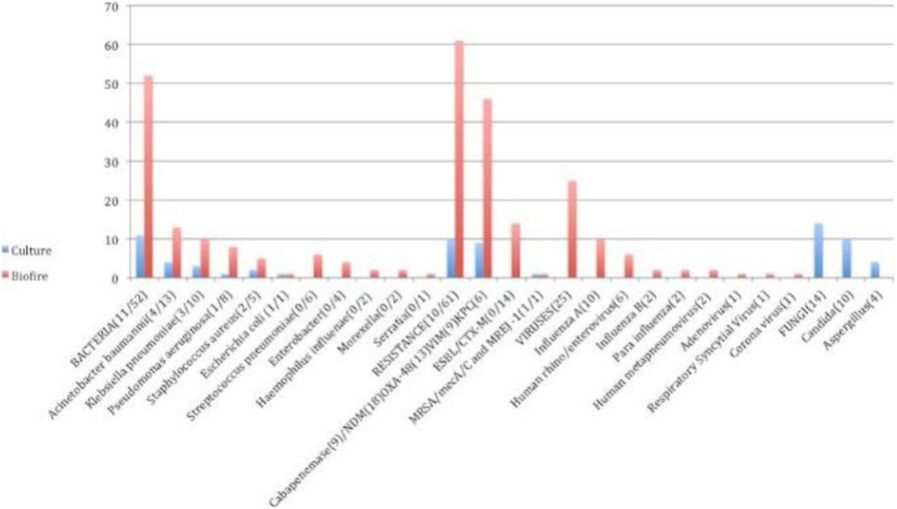
Frequency of organisms and bacterial resistances in culture and Biofire

## P447 Ceftaroline fosamil for treatment of severe community acquired pneumonia

### S Gallego zarzosa, M López de Olivencia, J Higuera Lucas, V Quinteros Fiel, M García Godes, C Soriano Cuesta, S García Plaza, R De Pablo Sánchez

#### H. U. Ramón y Cajal, Intensive Medicine, Madrid, Spain

**Introduction:**

Severe community-acquired pneumonia (SCAP) is a frequent cause of hospitalization and mortality. Ceftaroline is efficacious for treatment of CAP (PORT risk class III or IV). Most severe patients were excluded from the clinical trials, so the efficacy of ceftaroline in these kind of patients is unknown

**Methods:**

This is a health record-based retrospective before-after study in a tertiary care hospital. All SCAP patients admitted in ICU between November 2017 and February 2019 receiving ceftaroline were included. Control group included patients with same inclusion criteria but receiving ceftriaxone. Propensity scores to adjust for potential baseline differences between groups were performed. Levofloxacin or azythromicin were administered in both groups. Primary outcome was the change in SOFA score over the first 96h and secondary were days of mechanical ventilation, respiratory failure at 96h, need of rescue antibiotics, length of stay and mortality

**Results:**

There were 28 patients in ceftaroline group and 43 in ceftriaxone group. Baseline characteristics were similar except from more intubated patients in ceftaroline group (Figure 1). There were less respiratory failure at 96h in patients with ceftaroline treatment (-73.3% *vs.* -51.6%; p 0,015), but no differences in other organ failures, mortality, days of mechanical ventilation or LOS. There were more need of rescue antibiotics in ceftriaxone group (7.1% *vs* .46.5%; p 0,001). We found more *Streptococcus pneumoniae* isolation in ceftaroline group (18 (64.2%) *vs* 11 (25.5%); p = 0.001); more empiric use of oseltamir (16 (57.1%) *vs* 11 (25.5%); p = 0.012), but no more influenzae infections (11 (39.2%) *vs* 8 (18.6%); p = 0.101). *S. aureus* was detected in 1 patient in ceftaroline group and in 5 in ceftriaxone group.

**Conclusions:**

Ceftaroline is an efficacious treatment in patients with severe CAP, admitted in ICU. It relates to earlier resolution of respiratory failure and less rescue antibiotics. We need an adequately pragmatic trial to confirm our findings

**Fig. 1 (abstract P447).**
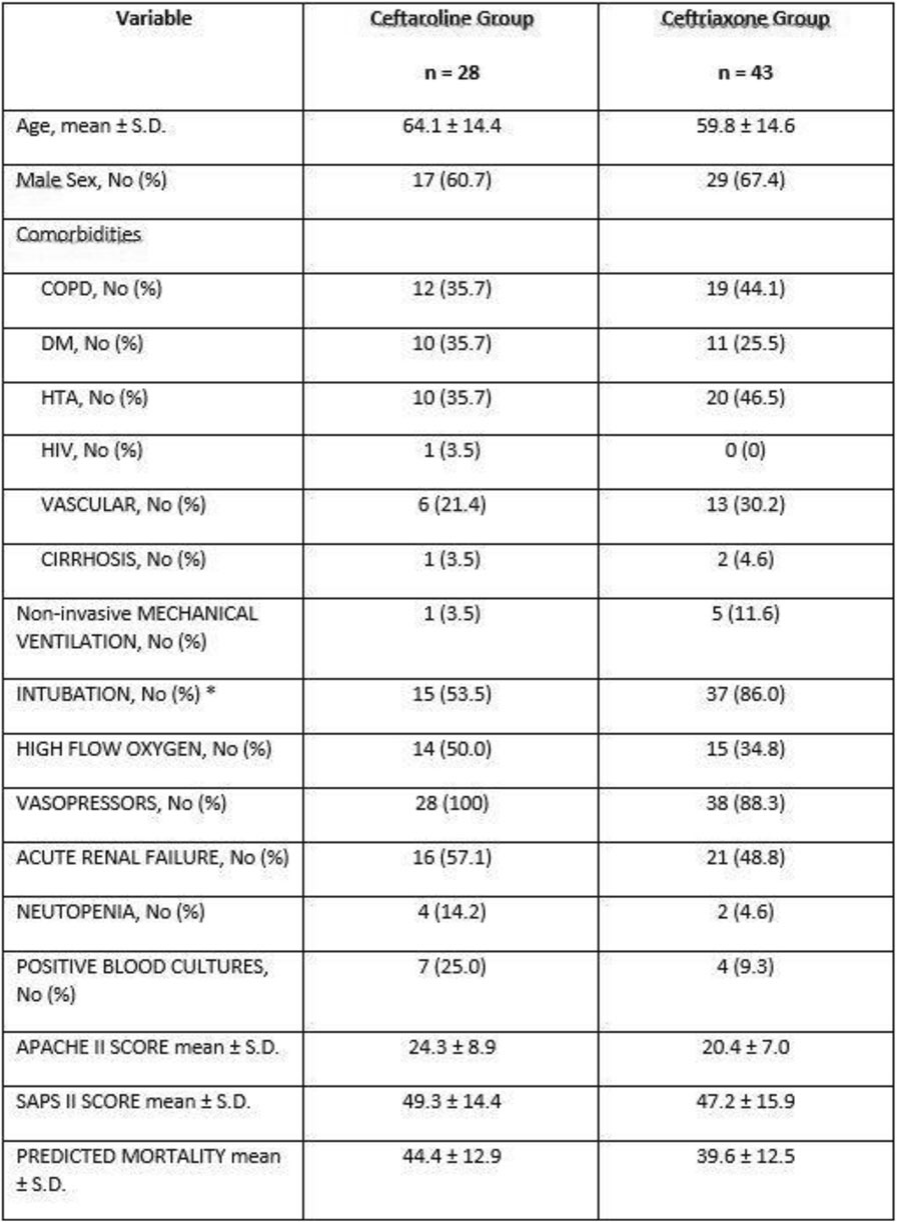
Baseline characteristics of ceftaroline and ceftriaxone groups

## P448 Organ dysfunction in scrub typhus, incidence and risk factor

### A Sarkar^1^, A Guha^2^, R Dey^3^

#### ^1^Peerless Hospital and B.K.Roy research center, Clinical Director of Critical care unit, Kolkata, India; ^2^Peerless Hospital and B.K.Roy research center, DNB Internal Medicine Postgraduate Trainee, Kolkata, India; ^3^Peerless Hospital and B.K.Roy research center, Consultant Critical care unit, Kolkata, India

**Introduction:**

Scrub typhus is caused by Orientia tsutsugamushi [1,2,3,4,5]. Its preads by bite of larval stageof thromboculid mites or chigger [1]. Clinical features may include fever, headache, myalgia, lymphadenopathy, eschar, skinrash. It may also cause pneumonia, renal failure, shock, meningoencephalitis, multiple organ failure[1,2]. Our study aims to discuss the incidence of organ dysfunction in a comprehensive way taking the overall population of patients with identified scrub typhus infection. There is lack of data in eastern India regarding the incidence and risk factors of developing multiorgan dysfunction syndrome (MODS) in scrub typhus.

**Methods:**

In this retrospective study we studied the incidence of various organ involvement and the risk factors associated with the development of MODS in scrub typhus. We collected data from December 2016 to November 2019 in tertiary care hospital at Kolkata. We have included all patients who are having fever, scrub typhus IgM antibody positive, age more than 14 years. SOFA score was used in evaluating patients with MODS. Exclusion criteria involves patient who are having co-infectional ong with scrub typhus.

**Results:**

In a cohort (n=114), patients with multiorgan dysfunction syndrome was seen in 27 patients (23.68%), the mean age in group of patients with MODS was 50.0+/-14.96 years (mean+/-SD). In group of patients with MODS, fever duration in days was of 11+/-3.58 days (mean+/-SD), interval from treatment to defervescenc in days was 5.11+/-2.39 days (mean +/-SD). Among patients with MODS, hematologic involvement was seen in 7 patients (25.92%), hepatic involvement was seen in 19 patients (70.37%), renal involvement was seen in 17 patients (62.96%), neurologic involvement was seen in 24 patients (88%), respiratory involvement was seen in 14 patients (51.85%), cardiovascular was seen in 8 patients (29.63%), ICU shifting was necessary in 20 patients (74.07%), mechanical intubation was needed in 14 patients (51.85%) in multiorgan dysfunction syndrome patients. Hospital mortality in patients with MODS was 3 patients (11.11%). No mortality was seen in patients without MODS. Other parameters were evaluated among patients with MODS. They include eschar in 1 patient (3.7%), seizure in 7 patients (25.93%), hepatoslenomegaly in 26 patients (96.3%), leucopenia in 3 patients (11.11%), leucocytosis in 13 patients (48.14%), thromnbocytopenia in 7 patients (25.92%),decreased hemoglobin in 22 patients (81.48%), transaminitis in 19 patients (70.37%). The risk factors associated with the development of MODS are platelet counts, bilirubin, transaminitis, Glasgow coma scale, time interval from treatment to defervescence, hemoglobin, total leucocyte count and fever duration.

**Conclusions:**

Scrub typhus is an important cause of acute febrile illness in this part of the country and is frequently associated with organ dysfunction. However, the overall mortality is low which is similar to other studies done before [2].

**References:**

1. Rungta N. Indian J Crit Care Med 18:489, 2014

2. Griffith M et al. Indian J Crit Care Med 18:497-502, 2014

3. Tsay RW et al. QJM 95:126-8, 2002

4. Takhar RP et al. National Medical Journal of India 30:69, 2017

5. Zhao D et al. J Pediatrics 63:167-73, 2016

## P449 Tuberculosis in intensive care medicine

### T Isidoro Duarte^1^, M Santos^2^, A Dias^2^, N Germano^1^

#### ^1^Curry Cabral Hospital, Central Lisbon University Hospital Center, Intensive Care Medicine Department, Lisboa, Portugal; ^2^Curry Cabral Hospital, Central Lisbon University Hospital Center, Infectious Diseases Department, Lisboa, Portugal

**Introduction:**

Acute respiratory failure (ARF) due to pulmonary infections is a usual cause of intensive care unit (ICU) admission. Immigration patterns and iatrogenic immune-suppression have made tuberculosis (TB) a common disease in Western Europe. Severe TB requiring ICU care is rare. Nevertheless, mortality associated with active TB and ARF is poor [1].

**Methods:**

Adult patients with TB admitted to ICU from 2014-2018 were identified retrospectively. Diagnosis was based on: positive cultures of sputum, bronchial aspirates or bronchioalveolar lavage fluid. Demographic characteristics, reasons for admission, HIV status, anti-TB treatment and mortality were recorded.

**Results:**

Total of 25 patients with TB were admitted to ICU. Mean APACHE II score was 20,2±6,9. Sixteen were male. Mean age 49,1±14,7 years. Eight (32%) were HIV-positive, 3 (12%) diabetes *mellitus* type 2, 3 (12%) chronic liver disease. Six (24%) had other causes of immune-suppression. Main causes for ICU admission were ARF due to non-*Mycobacterium tuberculosis* pathogens in 64%, acute liver failure in 12%, septic shock due to non-respiratory cause in 8%. Overall, 52% were on anti-TB treatment at time of admission. TB involved the lung parenchyma in all patients. Pleural involvement was present in 12% and lymph node in 20%. Extrapulmonary sites were present in 28%: urogenital, gastrointestinal, bone marrow. Pathogens identified in over-infections: 16% gram positive coccus, 20% gram negative bacilli, 16% fungal, 4% MDR-pathogen. One patient HIV-positive suffered ARF due to *Pneumocystis jiroveci*. Overall, 64% died during ICU stay.

**Conclusions:**

Besides its latent evolution, mortality of TB patients admitted to ICU is extremely high. ARF due to over-infection seems to be the main cause for ICU admission and mortality. Better preventive approach of these patients may improve their outcome.

**References:**

1. Erbes R et al. Eur Resp J 27:1223-1228, 2006

## P450 Critical care management of fulminant East African sleeping sickness

### D Hall^1^, J Harrington^1^, J Eapen^2^, S Chanda^3^

#### ^1^Royal Infirmary of Edinburgh, Edinburgh, United Kingdom; ^2^NYU Langone Medical Center, Division of Infectious Disease, New York, United States; ^3^University Teaching Hospital, Lusaka, Zambia

**Introduction:**

Human African Trypanosomiasis (HAT) is rarely encountered by critical care clinicians, but is an important differential for fever in the returning tropical traveler. Late disease is characterized by seizures, fever and multi-organ failure [1, 2]. We present an anonymized case presenting from an endemic area in Zambia referred for tertiary critical care management. The patient was too obtunded to give informed consent and his relatives could not be contacted despite extensive efforts.

**Methods:**

A middle-aged man with no past medical history from rural Zambia presented to a local clinical officer post with fever and arthralgia. He was treated twice with anti-malarial medication without resolution of symptoms. Two months later he was admitted febrile and obtunded to a local hospital with worsening confusion. He was transferred 8 hours by ambulance to our facility in Lusaka, which is the only public tertiary critical care unit in Zambia

**Results:**

GCS on arrival was E3M4V2 without localizing neurology. Microbiology investigations were negative, including for toxoplasma, cryptococcus, HIV or malaria. The patient suffered a generalized seizure followed by a sustained GCS of 3 and was admitted to the ICU for invasive ventilation and seizure control. Peripheral blood smears demonstrated trypanosomes consistent with HAT secondary to *Trypanosoma brucei rhodesiense.* He was commenced on melarsoprol but rapidly deteriorated, with signs of melarsoprol-induced arsenic encephalopathy and subsequent tonsillar herniation. His death was confirmed by neurological criteria.

**Conclusions:**

ICU management of fulminant HAT involves supportive neurocritical care plus melarsoprol, a toxic arsenic compound with common side effects of hepatotoxicity and dysrhythmia. Arsenic encephalopathy occurs in 10% of late HAT, with a fatality rate of 70% [1]. Early diagnosis is associated with a 95% survival rate in developed world travelers repatriated from endemic areas [2].

**References:**

1. Checkley J et al. Trans R Soc Trop Med Hyg 101:523–526, 2007

2. Frean J et al. Int J Infect Dis 75:101-108, 2018

## P451 Lithium chloride to prevent endothelial damage by serum from septic shock patients (in vitro study)

### A Kuzovlev^1^, A Shabanov^2^, O Grebenchikov^1^, I Kasatkina^1^

#### ^1^Federal research and clinical center of intensive care medicine and rehabilitology, Moscow, Russia; ^2^Federal research and clinical center of intensive care medicine and rehabilitology; N.V. Sklifosofsky research institute of emergency medicine, Moscow, Russia

**Introduction:**

The aim of the study was to investigate into effectiveness of lithium chloride (LiCl) as agent that prevents damage to the monolayer of endothelial cells under the action of serum from multiple trauma patients with septic shock.

**Methods:**

Serum from 5 pts with septic shock (Sepsis-3) and 5 healthy donors was withdrawn. Monolayer of Ea.hy926 endothelial cells were incubated for 3 hrs at 37 ° C with healthy person’s serum and with septic patient’s serum without LiCl and with it at concentrations of 0.01 mmol, 0.1 mmol, 1 mmol, 10 mmol. LiCl was added 1 hour before the change of serum. After incubation cells were washed and fixed with 2% paraform solution and permeabilized with 1% Triton X-100 solution. Fixed cells were stained with primary antibodies to VE-cadherin and then incubated with secondary antibodies conjugated with Oregon Green 488 fluorescent dye as well as with phalloid red and Hoechst dye 33342. Images were processed by fluorescence microscope and ImageJ 1.44p and MetaVue 4.6 programs. Western blotting was used to detect antibodies to VE-cadherin, claudin and GSK-3beta. Statistics included Mann-Whitney test and chi-square test.

**Results:**

Incubation of a monolayer of endothelial cells with 5% serum of septic shock patients led to loss of VE-cadherin contacts and decrease of claudine. Preincubation with LiCl 0.01 mmol did not prevent dismantling of claudine, actin, VE-cadherins; 0.1 mmol LiCl prevented it (p>0.05), but at higher concentrations (1 mmol, 10 mmol) almost completely protected endothelial monolayer from destruction of intercellular contacts (p<0.05). Serum had almost no effect on the phospho-GSK-3β level after 5 min, 15 min, 30 min and 1 hr, but caused a significant (60%) decrease in its level after 2 and 4 hrs. LiCl (1 mmol) caused a significant increase in phospho-GSK-3β already 15 mins and up to 4 hrs after exposure.

**Conclusions:**

LiCl prevents septic damage to the monolayer of endothelial cells in vitro in a GSK-3beta mediated way.

## P452 Autonomic nervous system, arterial stiffness and peripheral vascular tone alterations in septic shock: are they restored after resuscitation?

### M Carrara^1^, A Herpain^2^, G Baselli^1^, M Ferrario^3^

#### ^1^Politecnico di Milano, Milano, Italy; ^2^Université Libre de Bruxelles, Erasme University Hospital, Brussels, Belgium; ^3^Politecnico di Milano, Dept. of Electronics, Information and Bioengineering, Milano, Italy

**Introduction:**

The autonomic nervous system (ANS) controls both heart rate and vascular tone, which are known to be impaired during septic shock (SS) . Acute inflammation is presumed to increase arterial stiffness of large arteries in experimental studies [1]. The objectives of this work are to verify if standard SS resuscitation modulate mechanical vascular properties and to verify if alterations in these vascular properties and ANS activity are correlated.

**Methods:**

A protocol of fecal peritonitis septic shock and standard resuscitation (fluids and noradrenaline) was applied on 6 pigs. The arterial blood pressure waveform was recorded in the central aorta and in the femoral and radial arteries. The characteristic arterial time constant *tau* was computed at the three arterial sites, based on the two-element Windkessel model [2]. The total arterial compliance (AC) and the total peripheral resistance (TPR) were also estimated. Baroreflex sensitivity (BRS), low frequency (LF, 0.04-0.15 Hz) spectral power of diastolic blood pressure, and indices of heart rate variability (HRV) were computed to assess ANS functionality.

**Results:**

Septic shock induced a severe vascular disarray, decoupling the usual pressure wave propagation from central to peripheral sites, as shown by the inversion of pulse pressure (PP) amplification, with a higher PP in the central aorta than in the peripheral arteries during shock. The time constant *tau* together with AC and TPR were independently decreased. A decrease in BRS, LF power, and HRV describe an ANS dysfunction. After the administration of fluids and noradrenaline, both vascular and autonomic dysfunction persisted and these were found to be significantly correlated.

**Conclusions:**

Measures of mechanical vascular function and ANS activity could represent an useful end-point to guide further clinical investigations and refine our understanding of SS mechanisms, especially under medical treatment.

**References:**

1. Hatib et al. J Appl Physiol 111: 853-60, 2011

2. Mukkamala et al. IEEE TBME 53 :459–467, 2006.

## P453 Translational value of the microbial profile in experimental sepsis studies

### SP Tallósy^1^, A Rutai^1^, L Juhász^1^, MZ Poles^1^, K Burián^2^, D Érces^1^, A Szabó^1^, M Boros^1^, J Kaszaki^1^

#### ^1^University of Szeged, Faculty of Medicine, Institute of Surgical Research, Szeged, Hungary; ^2^University of Szeged, Faculty of Medicine, Institute of Clinical Microbiology, Szeged, Hungary

**Introduction:**

Adequate animal models are clearly needed for the development of truly effective clinical strategies for the treatment of sepsis. The recently accepted recommendations (Minimum Quality Threshold in Pre-Clinical Sepsis Studies - MQTiPSS, Osuchowski et al. Shock 2018) are defining several aspects of rodent sepsis models, but the microbiological background received relatively less attention. In this line our aim was to explore the significance of microbial profile in the outcome of peritonitis-induced sepsis models.

**Methods:**

Fecal peritonitis was induced in Sprague-Dawley rats (n=22, with 1.22×10^6^ – 5.9×10^6^ CFU ip) and Vietnamese minipigs (n=18. with 3.8×10^7^-6.2×10^9^ CFU). Invasive hemodynamic monitoring and blood gas analyses were performed on anesthetized animals between 18-24h of sepsis. The respiratory, cardiovascular, renal, hepatic and metabolic dysfunctions were evaluated with the species-specific Sequential Organ Failure Assessment (ssSOFA) score, the microbial profile was determined with selective media and MALDI-TOF MS in the initial inoculum and in the abdominal fluid taken 20h after sepsis induction.

**Results:**

Strong correlation was found between the initial dose of the inoculum (CFU) and the ssSOFA scores for organ dysfunction (rats: r = 0.656, P=0.0186; pigs: r=0.570, P = 0.0391). Similar to the human microbiota Protebacteria (rat: 53%; pig: 27%) and Firmicutes (rat: 40%; pig: 55%) were the predominant phyla in the induction inoculum in both model.

**Conclusions:**

Taxonomical similarity was present in both peritonitis model with human microbiota and the severity of organ dysfunction was associated with the initial bacterial concentration in both species. These findings can advance the process of refining the animal models to follow the latest guidelines.

**Grant supports:** NKFIH K116689, GINOP-2.3.2-15-2016-00034; EFOP-3.6.2-16-2017-00006

## P454 Intra-abdominal lipopolysaccharide (LPS) clearance and inactivation in peritonitis: key roles for lipoproteins and phospholipid transfer protein (PLTP)

### M Nguyen^1^, G Pallot^2^, A Tavernier^2^, A Dusuel^2^, D Masson^2^, L Lagrost^2^, PG Guinot^1^, T Gauthier^2^, B Bouhemad^1^

#### ^1^Dijon University Medical Center, Anesthesiology and Intensive Care, Dijon, France; ^2^University Bourgogne Franche-Comté, INSERM, LNC UMR1231, Dijon, France

**Introduction:**

Lipopolysaccharide (LPS), is a component of gram-negative bacteria known for its activation of the host immune system. The phospholipid transfer protein (PLTP) has previously been shown to promote the binding of LPS to lipoproteins, to limit inflammation and to lower mortality following injections of LPS or bacterial infection. The aim of the present study was to investigate the role of PLTP and lipoproteins in the detoxification of LPS from the peritoneal cavity.

**Methods:**

Injection of LPS intra-peritoneally (IP) (1mg/kg) to wild type (WT) and PLTP knocked-out mice (PLTP-KO) (n = 9 per group). Mass concentration and activity of LPS were quantitated by LCMSMS analysis of 3-hydroxymyristate and LAL bioassay, respectively. Lipoprotein fractions in plasma were separated by ultracentrifugation (n=10 vs n =12).

**Results:**

Following intra-peritoneal injection, clearance of intra-abdominal LPS was faster and plasma neutralization was more efficient in WT than in PLTP-KO mice (Figure 1). Indeed, LPS found in plasma of WT mice was proportionally less active, sustaining a higher capacity for WT mice to neutralize LPS (Figure 1B). Quantitative dosage of LPS in portal blood, 15 minutes after IP injection, revealed that plasma LPS associates rapidly with the lipoprotein fraction (HDL plus LDL), and in higher proportions as compared to PLTP-KO mice (66 [62-72] % vs 50 [41-54] %, respectively; p < 0.01). In line with previous studies, these observations now indicate that, LPS readily associates with lipoproteins in a neutralizing process PLTP mediated. Finally, even with a heavy LPS load (25 mg/kg), the bulk of LPS was still found in the lipoprotein fraction (80 [80-90] %), suggesting that lipoproteins plus PLTP in WT mice have a high capacity to detoxify intraperitoneal LPS.

**Conclusions:**

In a model of peritonitis, lipoproteins and PLTP were found to constitute key playors for peritoneal clearance and neutralization of LPS. It emerges as a key pathway for the resolution of the inflammatory response in peritonitis.

**Fig. 1 (abstract P454).**
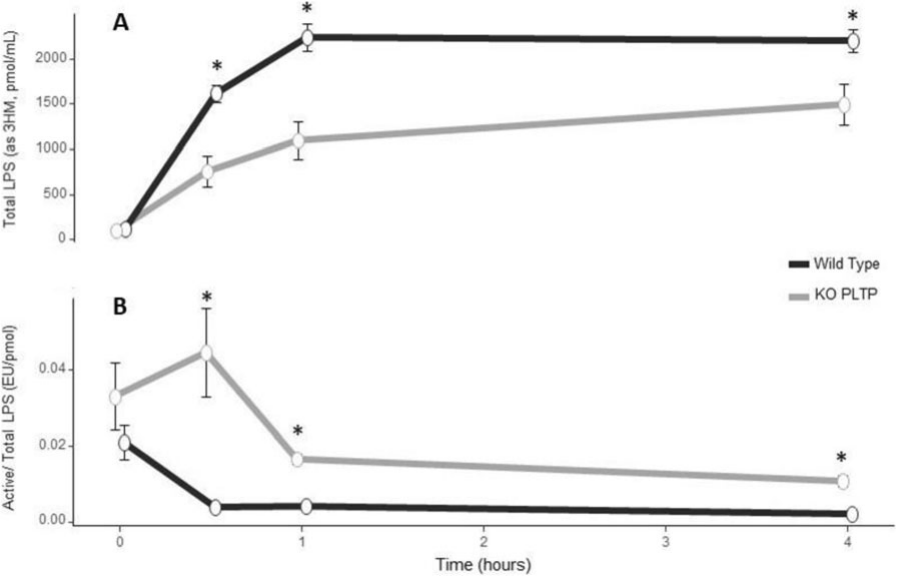
Time course of total concentration of LPS (A) and active to total concentration ratio (B) after intra-peritoneal injection in wild-type and PLTP knocked-out mice. Results are represented as mean and standard error of the mean * Significantly different LPS: Lipopolysaccharide; PLTP: Phospholipid transfer protein; PLTP-KO: PLTP knocked-out mice

## P455 Reduced ATX levels protect mice from LPS-induced endotoxemia

### I Nikitopoulou^1^, M Theodorakopoulou^2^, A Katsifa^3^, SE Orfanos^2^, V Aidinis^3^, A Kotanidou^4^

#### ^1^GP Livanos and M Simou Laboratories, 1st Department of Critical Care & Pulmonary Services, Medical School, National & Kapodistrian University of Athens, Athens, Greece; ^2^2nd Department of Critical Care, Attikon University Hospital, Athens, Greece; ^3^BSRC Alexander Fleming, Athens, Greece; ^4^1st Department of Critical Care & Pulmonary Services, Medical School, National & Kapodistrian University of Athens, Evangelismos Hospital, Athens, Greece

**Introduction:**

Autotaxin (ATX, Enpp2) is a secreted enzyme present in biological fluids that catalyses the production of lysophosphatidic acid (LPA). LPA is a bioactive phospholipid evoking various cellular responses in most cell types. Upregulated ATX levels have been reported in various chronic inflammatory diseases. Given the established role of LPA in the inflammatory response, we investigated a possible role for the ATX/LPA axis in LPS-induced endotoxemia.

**Methods:**

LPS was injected intraperitoneally (20 mg/kg) in mice producing 50% ATX levels (ATX^df/+^, heterozygous null mutant mice), in mice producing 20-30% reduced ATX levels upon inducible inactivation (R26CreER^T2^/Enpp2^n/n^ mice) and in mice expressing 150-200% increased ATX levels (Enpp2-Tg mice). Kaplan-Meier survival analysis was performed. ATX activity was measured using the TOOS activity assay.

**Results:**

ATX^df/+^ mice that produce almost 50% reduced serum ATX levels show increased survival compared to their littermate controls. For the inducible inactivation of ATX, Enpp2^n/n^ targeted mice were crossed with the R26Cre-ER^T2^ mice and tamoxifen induction enabled temporal control of floxed gene expression. R26CreER^T2^/Enpp2^n/n^ mice were more protected against LPS-induced endotoxemia compared to control mice. Enpp2-Tg mice overexpressing autotaxin and showing a 2-fold increase in plasma levels do not display improved survival rates compared to control group.

**Conclusions:**

ATX participates in systemic inflammation, as reduced ATX levels in circulation decrease lethality of mice from caused by LPS. The excess amount of circulating ATX does not exacerbate the systemic inflammatory response to LPS.

## P456 Neutrophil-related genes and pneumonia

### M Khadzhieva^1^, A Kuzovlev^2^, A Gracheva^3^, L Salnikova^1^

#### ^1^Federal Research and Clinical Center of Intensive Care Medicine and Rehabilitology; N.I. Vavilov Institute of General Genetics, Russian Academy of Sciences; Dmitry Rogachev National Research Center of Pediatric Hematology, Oncology and Immunology, Moscow, Russia; ^2^Federal research and clinical center of intensive care medicine and rehabilitology, Moscow, Russia; ^3^Federal research and clinical center of intensive care medicine and rehabilitology; N.I. Vavilov Institute of General Genetics, Russian Academy of Sciences, Moscow, Russia

**Introduction:**

Pneumonia (Pn) is a prevalent and severe infectious lung disease. Host genetics plays an essential role in the pathogenesis of infectious diseases including Pn [1]. The aim of the study was to analyze the variability of genes associated with neutrophil activation in pneumonia.

**Methods:**

To identify differential expressed genes (DEGs) in community-acquired (CAP) and hospital-acquired pneumonia (HAP) dataset «Genome-wide blood transcriptional profiling in critically ill patients - MARS consortium» (GSE65682) from Gene Expression Omnibus was analyzed (logFC≥2.0, FDR-corrected *P*-value<0.05). DEGs associated with neutrophil activation were selected according to gene ontology GO:0042119 («neutrophil activation»). With the use of GTEx Portal and Blood eQTL browser, we searched for eSNPs (expression single nucleotide polymorphisms) in whole blood for neutrophil activation genes differentially expressed in CAP/HAP. These eSNPs were further analyzed for their association with Pn via the Global Biobank Engine (GBE).

**Results:**

A total of 46 DEGs from GSE65682 correspond to GO:0042119 genes (43 up- and 3 down-regulated) of which 39 genes were common to CAP and HAP. Functional enrichment of 46 DEGs based on DisGeNET detected top-5 diseases associated with these genes (FDR-corrected *P*-value<0.05): Myeloid Leukemia, chronic; Sepsis; Asthma; Lung diseases; Allergic asthma. For these 46 genes 1366 eSNPs common to GTEx Portal and Blood eQTL browser were identified. More than half of all variants were located on the second chromosome and influenced the expression of *TNFAIP6* and *IL18RAP* genes. Among all eSNPs we identified variants associated with Pn in the GBE (Table 1).

**Conclusions:**

We identified genes related to neutrophil activation, genetic variability of which was associated with pneumonia.

**References:**

1. Dela Cruz CS et al. Am J Respir Crit Care Med 198:256-263; 2018

**Table 1 (abstract P456).** eSNPs associated with pneumonia in the Global Biobank Engine

eSNP, allele	Gene (up (↑)/down (↓) regulation for eSNP allele)	Resource (GTEx or eQTL blood)	P-value (GTEx / eQTL blood)	Risk allele for pneumonia in GBE (P-value)
rs11692150-C	TNFAIP6(↓)	GTEx/eQTL blood	3.0E-7 / 6.70E-17	rs11692150-C (0.00272)
rs9923575-T	HP(↓)	GTEx/eQTL blood	8.3E-5 / 7.14E-71	rs9923575-T (0.00244)
rs581623-C	FCAR(↓)	GTEx/eQTL blood	8.5E-6 / 2.87E-5	rs581623-C (0.00540)
rs269938-T	FCAR(↑)	eQTL blood	2.24E-5	rs269938-T (0.00461)
rs8111613-C	PGLYRP1(↓)	eQTL blood	1.89E-5	rs8111613-C (0.00342)

## P457 Association of host-derived hydrogen sulfide with neutrophil phagocytosis of multidrug resistant Pseudomonas aeruginosa

### G Renieris^1^, P Koufargyris^2^, D Droggiti^2^, T Gkavogianni^2^, L Sabrakos^2^, E Jentho^3^, S Weis^3^, M Bauer^3^, A Papapetropoulos^4^, E Giamarellos-Bourboulis^2^

#### ^1^4th Department of Internal Medicine, National and Kapodistrian University of Athens, Medical School, 4th Department of Internal Medicine, National and Kapodistrian University of Athens, Medical School, Athens, Greece; ^2^4th Department of Internal Medicine, National and Kapodistrian University of Athens, Medical School, Athens, Greece; ^3^Department of Anesthesiology and Intensive Care, Institute for Infectious Disease and Infection Control, Center for Sepsis Control and Care, Jena University Hospital, Jena, Germany; ^4^Laboratory of Pharmacology, Faculty of Pharmacy, National and Kapodistrian University of Athens, Athens, Greece

**Introduction:**

The role of hydrogen sulfide (H_2_S) in the pathogenesis of severe bacterial infections is unclear. We investigated the role of host-produced H_2_S through cystathionine-γ-lyase (Cse) in an animal infection model of multi-drug resistant (MDR) *Pseudomonas (P.) aeruginosa*.

**Methods:**

Sepsis was induced in wild-type C57Bl6 mice (n=41) and *Cse* knockout mice (n=41) by *i.p.* injection of 10^8^ cfu/mice MDR *P. aeruginosa*. Similar experiments were repeated after cyclophosphamide induced neutropenia. Survival was recorded for 7 days. Mice were sacrificed for determination of bacterial load and myeloperoxidase (MPO) activity as a surrogate marker of myeloid cell recruitment. Cytokines were measured in serum by Legendplex inflammatory panel. Total leukocytes from mice spleens, with or without pretreatment with the H_2_S donor GYY3147, were incubated with 1 x 10^4^ cfu/mL MDR *P. aeruginosa*. Bacterial clearance was recorded.

**Results:**

We observed a significant decrease in survival of *Cse*^*-/-*^ mice as compared to *Cse*^*+/+*^ mice (12% vs. 47%; p: 0.025). This survival advantage was eliminated in neutropenic mice (17% for both groups, p: 0.873). *Cse*^*-/-*^ mice had increased pathogen load in the liver (6.57 ± 0.13 vs 5.26 ± 0.50, p: 0.029) and lung (6.70 ± 0.17 vs 5.29 ± 0.55, p: 0.035). MPO activity was lower in *Cse*^*-/-*^ mice in the liver (634 ± 71 vs 1029± 179, p: 0.048) and lung (7627 ± 585 vs 11121 ± 1468, p: 0.34). *Cse*^*+/+*^ mice had increased serum levels of IL- 23 (121.13 ± 33.68 vs 31.41 ± 7.02 of *Cse*^*-/-*^, p: 0.001); MCP-1 (4769.91 ± 908.83 vs 1940.37 ± 1062.65, p: 0.026) and GM-CSF (22.91 ± 4.66 vs 8.11 ± 1.92, p: 0.004). Phagocytic activity of leukocytes from *Cse*^*-/-*^ mice was reduced compared to *Cse*^*+/+*^ mice. This deficit was eliminated after GYY4137 pretreatment (Fig. 1).

**Conclusions:**

Deficiency of host- derived H_2_S leads to increased susceptibility to MDR *P. aeruginosa* infection due to an inefficient neutrophil chemotaxis and neutrophil mediated phagocytosis.

Acknowledgement

Funded by the ITN Horizon 2020 Marie-Curie European Sepsis Academy

**Fig. 1 (abstract P457).**
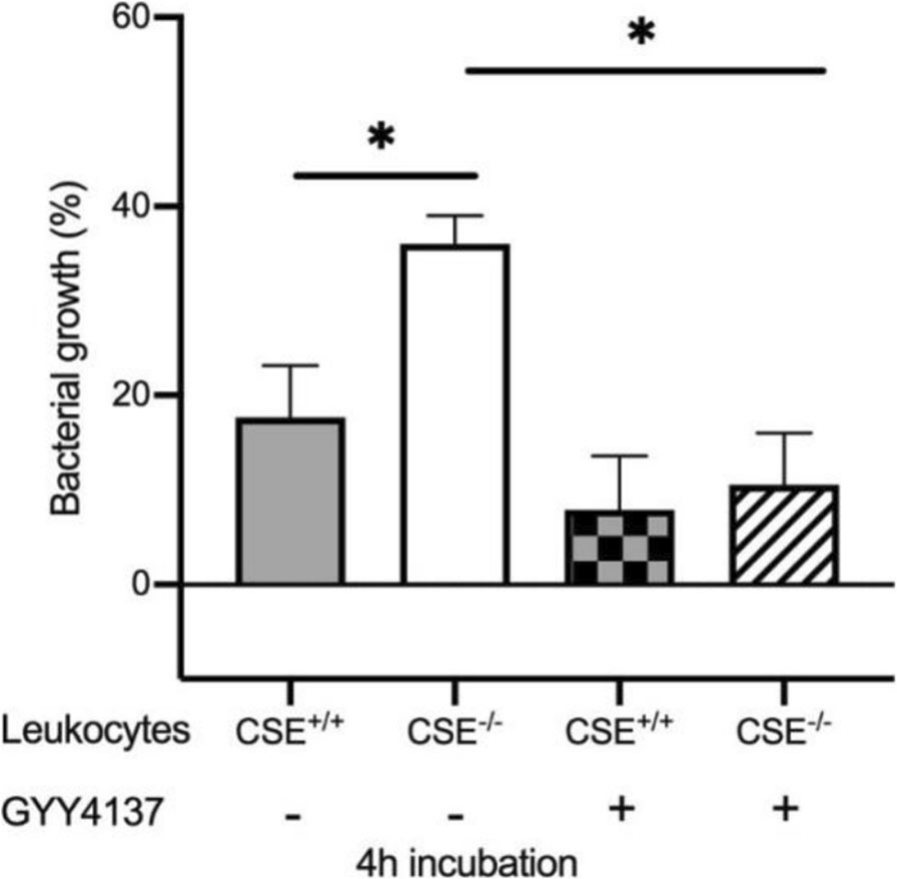
Effect of hydrogen sulfide on the killing of multi-drug resistant Pseudomonas aeruginosa by total leukocytes in vitro

## P458 Brain tight junction protein expression in sepsis in an autopsy series

### KE Erikson^1^, HT Tuominen^2^, MV Vakkala^3^, JL Liisanantti^4^, TK Karttunen^2^, HS Syrjälä^5^, TA Ala-kokko^4^

#### ^1^The North Estonia Medical Centre, Centre of Intensive Care, Tallinn, Estonia; ^2^Oulu University Hospital, Department of Pathology, Oulu, Finland; ^3^Oulu University Hospital, Department of Anesthesiology, Oulu, Finland; ^4^Oulu University Hospital, Department of Anesthesiology, Division of Intensive Care Medicine, Oulu, Finland; ^5^Oulu University Hospital, Department of Infection Control, Oulu, Finland

**Introduction:**

Neuroinflammation often develops in sepsis along with increasing permeability of the blood-brain barrier (BBB), which leads to septic encephalopathy [1]. The barrier is formed by tight junction structures between the cerebral endothelial cells [2]. We investigated the expression of tight junction proteins related to endothelial permeability in brain autopsy specimens in critically ill patients deceased with sepsis, and analyzed the relationship of BBB damage and measures systemic inflammation and systemic organ dysfunction.

**Methods:**

Case series included all adult patients deceased with sepsis in the years 2007- 2015 with brain specimens taken at autopsy available**.** Specimens were categorized according to anatomical location (cerebrum, hippocampus, cerebellum). The immunohistochemical stainings were performed for occludin, ZO-1 and claudin. Patients were categorized as having BBB damage if there was no expression of occludin in the endothelium of cerebral microvessels.

**Results:**

38% (18/47) developed multiple organ failure before death. 74.5% (35/47) had septic shock. The deceased with BBB damage had higher SOFA maximum scores (16 vs.14, p=0.04), and had more often procalcitonin levels above 10 (56 % vs.28 %, p=0.045). BBB damage in cerebellum was more common in cases with c reactive protein above 100 mg/L as compared with CRP less than 100 (69% vs. 31 %, p=0.025). Absence of ZO-1 expression in cerebral meningeal samples associated with BBB damage (17 % vs. 0 %, p=0.046). Positive blood cultures (n = 22) were associated to absence of ZO-1 expression in cerebellar glial cells (92 % vs. 44 %, p=0.018).

**Conclusions:**

In fatal sepsis, damaged BBB defined as loss of cerebral endothelial expression of occludin (Figure 1) is related with severe organ dysfunction and systemic inflammation. Loss of ZO-1 in endothelial cells associates with BBB damage, and sepsis contributes to ZO-1 loss in cerebellar glial cells.

**References:**

1. Tietz S. J Cell Biol 209:493-506, 2015

2. Davies DC. J Anatomy 200:639-646, 2002.

**Fig. 1 (abstract P458).**
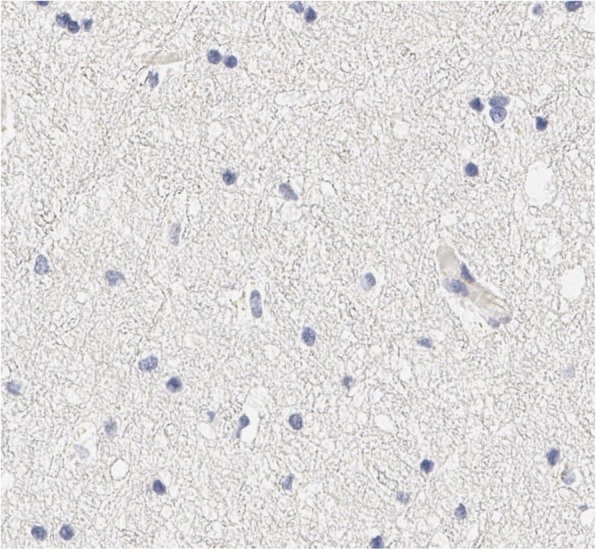
No expression of occludin

## P459 Oxylipins in septic shock: a post-hoc analysis of the VANISH randomized clinical trial

### TN Jones, F Al-Beidh, AC Gordon, DB Antcliffe

#### Imperial College London, Division of Anaesthetics, Pain Medicine and Intensive Care, Department of Surgery and Cancer, London, United Kingdom

**Introduction:**

Oxylipins are oxidative breakdown products of cell membrane fatty acids. Animal models have demonstrated that various vasoactive oxylipin pathways may be implicated in septic shock pathophysiology but these have been poorly studied in humans.

**Methods:**

Oxylipin profiling was performed on serum samples collected on enrolment to the VANISH (Vasopressin vs. Norepinephrine as Initial Therapy in Septic Shock) trial. Samples were analysed with liquid chromatography-mass spectrometry. Patients were followed up until 28 days.

**Results:**

Samples were collected from 154 of 409 (37.7%) patients on inclusion to the trial and 39 (25.3%) had died by 28 days. Non-survivors were found to have higher levels of a number of oxylipins including: 14,15-dihydroxyeicosatrienoic acid (DHET) (p<0.01), 11,12-DHET (p=0.03), 15(S)-hydroxyeicosatetraenoic acid (p=0.02), 14-hydroxyoctadeca-pentaenoic acid (p=0.04) but lower levels of the precursor eicosapentaenoic acid (p=0.012). When corrected for multiple comparisons with the Benjamini-Hochberg test, only 14,15-DHET remained significant (p=0.025). Although there was a difference in median 14,15-DHET levels between survivors and non-survivors, many values were below the level of detection (n=84/154 (54.5%)). As such, we also analysed 14-15-DHET as a binary variable (Figure 1). Patients with detectable 14,15-DHET were more likely to die (HR 2.4 [95% CI 1.2-4.6], p<0.01) and have a higher median lactate (p =0.01) and total SOFA score (p<0.01) than those patients where baseline 14,15-DHET was undetectable.

**Conclusions:**

Our study suggests the oxylipin 14,15-DHET may be associated with septic shock severity and 28-day mortality. These results are consistent with the known vasodilatory actions of this class of oxylipin. More work is needed to confirm its exact role in septic shock and whether this pathway is amenable to therapeutic intervention.

**Fig. 1 (abstract P459).**
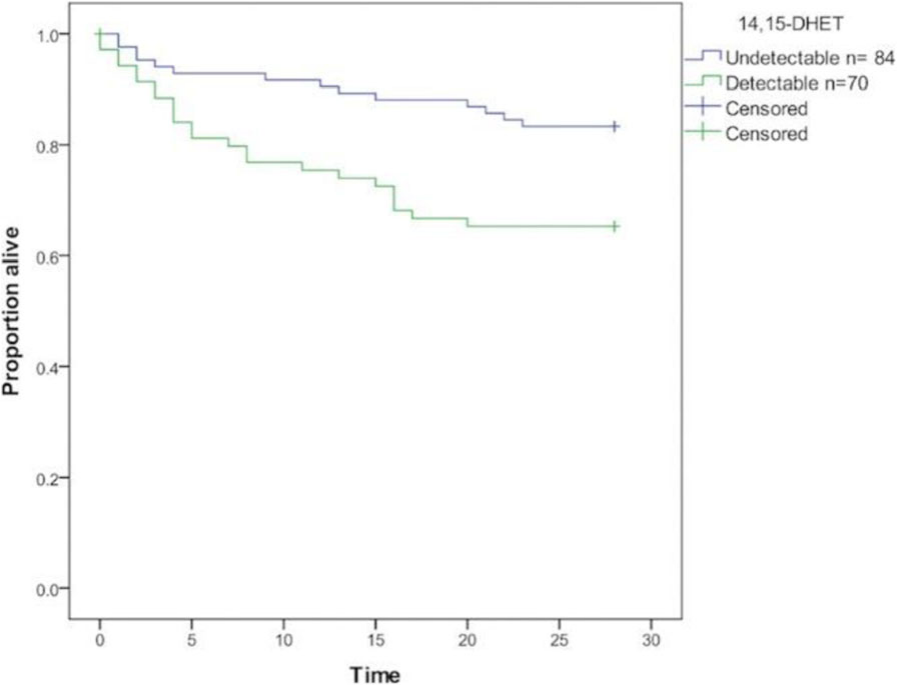
Survival curve for patients with vs without detectable levels of the oxylipin 14,15-DHET. Followed up until 28 days

## P460 Lithium chloride influence neutrophil activation in vitro

### A Kuzovlev, O Grebenchikov, A Shabanov, I Kasatkina, V Moroz

#### Federal research and clinical center of intensive care medicine and rehabilitology, Moscow, Russia

**Introduction:**

Activation of neutrophils is a mandatory stage and a sensitive marker of systemic inflammatory conditions that can lead to the development of multiorgan failure. The aim of the study was to investigate into the antiinflammatory effects of lithium chloride on human neutrophils in vitro.

**Methods:**

Study was carried out on neutrophils isolated from the blood of 5 healthy donors. 50% of neutrophils were activated by 100 mkM fMLP, 50% - by 100 ng/ml lipopolysaccharide (LPS); then their activity was evaluated by fluorescent antibodies to CD11b and CD66b degranulation markers. Intact and activated neutrophils were treated with a solution of lithium chloride (9 mmol). Immunoblotting was used to assess GSK3b activity in neutrophils. Mann-Whitney criterion and p<0.05 were used for statistics.

**Results:**

Lithium chloride 9 mmol decreased the level of expression of CD11b on intact neutrophils by 16% (p=0.07), CD66b by 15% (p=0.07). fMLP increased CD11b expression on neutrophils by 2.6 times (p=0.0007), CD66b by 2.5 times (p=0, 0022). Addition of lithium chloride solution to fMLP activated neutrophils reduced the expression of CD11b (p=0.0317) and CD66b (p=0.0079). LPS increased CD11b and CD 66b expression by 2.1 times (p=0.0007, p=0.0022, respectively); addition of lithium chloride reduced the expression of CD11b (p=0,0317) and CD66b (p=0.0079) on neutrophils. fMLP led to a dephosphorylation of GSK-3b by 47% (p<0.05), lithium chloride increased its phosphorylation by 387% (p <0.05). Adding lithium chloride to activated fMLP neutrophils restored the level of GSK-3b phosphorylation by 277% compared to controls (p<0.05).

**Conclusions:**

Lithium chloride modulates the inflammatory activation of neutrophils by bacterial components through the phosphorylation of GSK3b in neutrophils.

## P461 Human host immune responses to lipopolysaccharide: A comparison study between in vivo endotoxemia model and ex vivo lipopolysaccharide stimulations using an immune profiling panel

### DM Tawfik^1^, JM Lankelma^2^, L Vachot^3^, E Cerrato^3^, A Pachot^3^, WJ Wiersinga^2^, J Textoris^3^

#### ^1^bioMerieux, Medical Diagnostic Discovery Department (MD3), Lyon, France; ^2^Amsterdam UMC, Amsterdam, Netherlands; ^3^bioMerieux, Lyon, France

**Introduction:**

Sepsis, a leading cause of mortality among critically-ill patients in the ICU, recently recognized by the WHO as a global health burden. Patients that suffer from sepsis exhibit an early hyper-inflammatory immune response which can lead to organ failure and death. In our study, we assessed the immune modulations in the human *in vivo* endotoxemia model and compared it to *ex vivo* lipopolysaccharides (LPS) stimulation using 38 transcriptomic markers.

**Methods:**

Eight healthy volunteers were challenged with intravenous LPS *in vivo*. In parallel, blood from another 8 volunteers was challenged with LPS *ex vivo*. Blood was collected before and after 4 hours of LPS challenge and tested with the Immune Profiling Panel (IPP) prototype using the FilmArray® system.

**Results:**

The use of IPP showed that markers from the innate immunity dominated the response to LPS *in vivo*, mainly markers related to monocytes and neutrophils. Comparing the two models, *in vivo* and *ex vivo*, revealed that most of the markers were modulated in a similar pattern (68%). Some cytokine markers such as *TNF*, *IFN-γ* and *IL-1β* were under-expressed *ex vivo* compared to *in vivo*. T-cell markers were either unchanged or up-modulated *ex vivo*, compared to a down-modulation *in vivo*. Interestingly, markers related to neutrophils were expressed in opposite directions, which might be due to the presence of cell recruitment and feedback loops *in vivo*.

**Conclusions:**

The majority of IPP markers showed similar patterns of expression post-LPS challenge in both models, except for several markers related to neutrophils and T-cells. The IPP tool was able to capture the early immune response in the human *in vivo* endotoxemia model, which is a translational model mimicking immune host response in septic patients.

## P462 Are baseline levels of soluble Mer and Gas6 predictors of mortality and organ failure in septic patients? The Need-Speed trial database

### F Gavelli, L Molinari, L Salmi, M Baldrighi, F Patrucco, M Bellan, PP Sainaghi, GC Avanzi, LM Castello

#### Università degli Studi del Piemonte Orientale, Department of Translational Medicine, Novara, Italy

**Introduction:**

Serum levels of tyrosine kinase receptor Mer and its ligand Gas6 predict mortality in septic patients in the intensive care unit. However, whether their early measurement at emergency department (ED) presentation also predicts mortality and organ failure still needs to be clarified.

**Methods:**

In this multicentre observational study, septic patients admitted to 5 Italian EDs were included [1]. At ED presentation blood samples were taken for routine biochemical analyses and serum Mer and Gas6 measurement. Urinalyses, blood gas analyses and chest X-ray were routinely performed. Mortality at 7 and 30 days, as well as the presence of organ damage such as acute kidney injury (AKI), thrombocytopenia, PT-INR derangement and sepsis-induced coagulopathy (SIC) were evaluated according to baseline levels of Mer and Gas6.

**Results:**

890 septic patients were enrolled between March 2013 and March 2015. 7 and 30-day mortality were 9.7% and 19.9%, respectively. Mer and Gas6 baseline levels were 31.1[23.2–43.5]ng/mL and 8.3[4.0–14.4]ng/mL each, and no difference was observed between survivors and non-survivors, at both 7 and 30 days (p>0.05). AKI patients showed higher Mer and Gas6 levels compared to the non-AKI ones (9.8[4.1–17.8] *vs.* 7.9[3.8–12.9]ng/mL and 34.8[26.4–47.5] *vs.* 29.8[22.1–41.6]ng/mL, respectively; p<0.01). However, at the multivariate analysis neither Mer nor Gas6 were confirmed as AKI predictors. On the contrary, both Mer and Gas6 independently predicted thrombocytopenia in septic patients (OR 1.01[1.00-1.02] and 1.04[1.02-1.06], respectively). Moreover, Mer emerged as an independent factor for developing both PT-INR derangement (OR 1.03[1.00-1.06]) and SIC (OR 1.05[1.02-1.07]) (p<0.001).

**Conclusions:**

In conclusion, neither Mer nor Gas6 are early predictors of mortality in septic patients at ED presentation. However, Mer independently predicted the development of SIC, thrombocytopenia and PT-INR derangement in this population.

**References:**

1. Mearelli F et al. Crit Care Med 46:1421-1429, 2018.

## P463 Glycocalyx shedding correlates with positive fluid balance and respiratory failure in patients with septic shock

### N Takeyama, Y Kajita, T Terajima, H Mori, T Irahara, M Tsuda, H Kano

#### Aichi Medical University, Department of Emergency and Critical Care Medicine, Aichi, Japan

**Introduction:**

Endothelial hyperpermeability would play a major role in septic shock related organ failure. The aim of this study is to clarify the relationship between glycocalyx shedding and respiratory failure, SOFA score, plasma angiopoietin (Ang)-2 level and patient survival.

**Methods:**

Plasma samples were collected from 30 septic shock patients from admission to ICU discharge and 10 healthy volunteers. Plasma syndecan (Syn)-1 and Ang-2 were measured and clinical data was also collected. Septic shock patients were classified into 3 groups according to the time-course change of Syn-1 levels. Excess Syn-1 (>400 ng/ml) during 0 to 3 days and remaining high following 4 to 7 days were assigned to Group I. Excess Ang-2 during 0 to 3 days and decreased following 4 to 7 days were assigned to Group II. Moderate increase (<400 ng/ml) during 0 to 7 days were assigned to Group III.

**Results:**

Plasma Syn-1 levels are positively associated with increased Ang-2 levels (r2=0.41, P= 0.005), suggesting that Ang-2 is involved in endothelial hyperpermeability. Fluid balance and ventilator-free days (VFD) are significantly increased in Group I as compared with Group III. SOFA score, Apache II and patient outcome does not show any differences between Groups I, II, and III.

**Conclusions:**

The positive correlation between glycocalyx shedding and fluid balance indicates plasma Syn-1 may be a valuable marker for endothelial hyperpermeability. The negative correlation between glycocalyx shedding and VFD indicates plasma Syn-1 may be a valuable marker for respiratory failure. The plasma level of Syn-1 for prognosis and organ failure excluding ARDS in patients with septic shock requires further investigation.

## P464 Serial procalcitonin measurements in the intensive care unit at Hiroshima University Hospital

### K Hosokawa, S Yamaga, M Fujino, K Ota, N Shime

#### Hiroshima University Hospital, Department of Emergency and Critical Care Medicine, Hiroshima, Japan

**Introduction:**

Serum procalcitonin (PCT) is a promising biomarker for differentiating bacterial infections from other inflammatory states. Moreover, including serial PCT measurements in the management of acute respiratory infection reduces the duration of antibiotic therapy without increasing the mortality. However, limited real-world information is available regarding the use of PCT in intensive care units (ICUs).

**Methods:**

We extracted and analysed data from January 1 to December 31, 2018 from all the orders and results of PCT measurements in the ICU (26 beds) at Hiroshima University Hospital.

**Results:**

A total of 1,252 PCT measurements from 409 ICU patients were included. In 170 patients, PCT was tested ≥3 times during a single ICU stay. Serial PCT measurements showed a fade-out pattern (76 [45%] patients), a second day-peaked decrease pattern (35 [21%] patients), and a series of negative patterns (30 [18%] patients). Compared to patients who demonstrated the fade-out pattern, those who demonstrated the second day-peaked decrease pattern had higher mortality rates (3% vs. 20%, p < 0.01).

**Conclusions:**

Approximately one-third patients in the ICU who had decreasing serial PCT values demonstrated the second day-peaked decrease pattern. Since this group of patients had poorer survival, further studies are needed to clarify the association between a late rise in PCT levels and delayed therapeutic intervention.

## P465 The early diagnostics of the sepsis of newborns on mechanical ventilation

### M Pukhtinskaya^1^, V Estrin^2^

#### ^1^State Medical University, Department of Anesthesiology and Critical Care Medicine, Rostov on don, Russia; ^2^State Medical University, Rostov on don, Russia

**Introduction:**

Sepsis diagnostics during the pre-clinical stage remains a most complex issue. Research objective: increasing the efficiency of sepsis diagnostics.

**Methods:**

The research was performed on 200 full-term newborns; no clinical signs of bacterial infection were diagnosed. On the 1, 5, 20 days the plasmà concentration of IL-1ß, IL-6, IL-8, TNF-α, G-CSF, sFas, FGF, NO was determined by capture ELISA; CD3CD19, CD3CD4, CD3CD8, CD69, CD71, CD95, HLA-DR, CD34, CD14, CD3CD56, lymphocytes in apoptosis - immunophenotype analysis. By applying the statistical cluster population analysis of the immunological criteria under study we have evaluated the feasibility of sepsis diagnostics at the admission to the intensive therapy unit. The diagnostic rule for sepsis has been formulated By applying the "decision tree" approach to the "R" statistic medium.

**Results:**

The cluster analysis confirms the presence of two clusters (presence of absence of sepsis: these two components explain the 60.81% of the point variability). The diagnostic rule for the early diagnostics of sepsis is as follows: disease develops providing during the first 48 hours CD95≥16.8%, NO≤9.6 mkmol/l or CD95≤16.8%, CD34≤0.2%, CD69≥4.12% or CD95≤16.8%, CD34≤0.2%, CD69≤4.12% and lymphocytes AnnexinV-FITC+PI-≥12.3%. 45 newborns featured the confirmed sepsis development. The accuracy of this diagnostics amounts to 95.41%; sensitivity to 97.06%; specificity to 94.67%; diagnostic false positive share to 5.33%; diagnostic false positive share to 2.94%; positive result accuracy to 89.19%; negative result accuracy to 98.61%.

**Conclusions:**

The aggregate determination of CD95, CD69, AnnexinV-FITC+ PI-, CD34 and the plasma concentration of NO enables the pre-clinical diagnostics of sepsis development.

## P466 Efficacy of pancreatic stone protein in diagnosis of infection in adults: a systemic review and metaanalysis of raw patient data

### J Prazak^1^, P Egimann^2^, I Irincheva^3^, MJ Llewelyn^4^, D Stolz^5^, LG De Guadiana-Romualdo^6^, R Graf^7^, T Reding^7^, HJ Klein^8^, YA Que^1^

#### ^1^Inselspital, Bern University Hospital, University of Bern, Department of Intensive Care Medicine, Bern, Switzerland; ^2^University Hospital Medical Center (CHUV) and University of Lausanne, Department of Intensive Care Medicine, Lausanne, Switzerland; ^3^Inselspital, Bern University Hospital, University of Bern, Clinical Trial Unit, Bern, Switzerland; ^4^Brighton and Sussex Medical School, Falmer, United Kingdom; ^5^University Hospital Basel, Clinic of Pulmonary Medicine and Respiratory Cell Research, Basel, Switzerland; ^6^Santa Lucia Hospital, Clinical Chemistry Laboratory, Cartagena, Spain; ^7^University Hospital Zurich, Department of Visceral and Transplantation Surgery, Zurich, Switzerland; ^8^University Hospital Zurich, Department of Plastic Surgery and Hand Surgery, Burn Center Zurich, Zurich, Switzerland

**Introduction:**

Pancreatic stone protein (PSP) has shown promise as a biomarker of infection however, its diagnostic potential has not been systematically evaluated. We performed a systematic review and meta-analysis of available data on PSP to evaluate its value for detecting infection in adults and determining a plasma or serum threshold value.

**Methods:**

The PubMed and Cochrane Library database were searched for studies on PSP in adult patients and their raw data were analyzed to estimate the best PSP cut-off value that could detect infected patients using the Youden’s index. The cut-off sensitivity, specificity, positive predictive value (PPV) and negative predictive value (NPV) were computed and compared to those for procalcitonin (PCT) and C-reactive protein (CRP). Finally, we explored the potential value of a model combining all three biomarkers to detect infection.

**Results:**

From a total of 44 potentially eligible published studies, 5 containing 631 patients were included in quantitative analysis. Among them, 370 patients suffered from a clinically confirmed infection. The median [IQR ] PSP values of infected versus uninfected patients were 81.8 [31.19, 237.75] versus 19.2 [12.66, 33.4] ng/ml respectively, compared to 150 [83.75, 229.32] versus 64 [18.1, 124] mg/l for CRP and 0.9 [0.3, 4.4] versus 0.3 [0.07, 0.5] ng/ml for PCT. When the PSP cut-off value was fixed to 44.2ng/ml for infection versus non-infection, the ROC AUC for PSP reached 0.81 (0.78, 0.84) with a sensitivity 0.66 (0.62, 0.71), specificity 0.83 (0.79, 0.88) PPV 0.85 (0.81, 0.89) and NPV 0.64 (0.58, 0.69). Using a joint model including PSP and CRP, the ROC AUC reaches 0.90 (0.87, 0.92) with respective higher sensitivity 0.82 (0.78, 0.86) and specificity 0.85 (0.79, 0.90) for discriminating infection from non-infection.

**Conclusions:**

In adult patients, a PSP threshold of 44.2ng/ml identifies patients with infection. The combination of PSP with CRP increased further its ability to discriminate between infection and non-infection.

## P467 Clinical correlation of neutrophil CD64, PCT and CRP in ICU patients with sepsis/septic shock

### R Patnaik^1^, A Azim^1^, V Agarwal^2^, P Mishra^3^

#### ^1^Sanjay Gandhi Postgraduate Institute of Medical Sciences, Critical Care Medicine, Lucknow, Uttar Pradesh, India; ^2^Sanjay Gandhi Postgraduate Institute of Medical Sciences, Clinical Immunology, Lucknow, Uttar Pradesh, India; ^3^Sanjay Gandhi Postgraduate Institute of Medical Sciences, Biostatistics, Lucknow, Uttar Pradesh, India

**Introduction:**

Neutrophil CD64 is evolving as a prognostic biomarker. The primary objective was to correlate serial levels of CD64, PCT (Procalcitonin) and CRP (C-reactive protein) with mortality during ICU stay.

**Methods:**

It is a prospective observational study carried out after Institutional Ethics Committee approval in a 20 bedded mixed ICU from December 2017 onwards. Adult patients of >18 years with diagnosis of sepsis or septic shock (as per sepsis 3 criteria) at admission were included. Demographic and clinical parameters were noted at admission. Biomarker levels were measured serially at admission, on day 4 and day 8 of ICU stay along with clinical and laboratory parameters. Appropriate statistical tests were used using SPSS 23. CD 64 was expressed as % age of neutrophils expressing positivity.

**Results:**

Sixty patients were analyzed. All parameters were compared between survivors and non survivors. Demographics were comparable. Most common source of sepsis was lungs and majority were admitted due to medical reason. Non-survivors had significantly increased number of days with septic shock. At day 8 median values of all the biomarkers and the SOFA score were significantly higher in the non-survivor group (p<0.05). There was a decreasing trend of all 3 biomarkers and SOFA score amongst survivors. On multivariate logistic regression analysis, increased CD64 and CRP levels between baseline and day 8, increased days with septic shock and increased SOFA score at baseline were significant (p<0.05) predictors of mortality. Highest area under the ROC curve was obtained for number of days with septic shock (0.857) followed by increased CD64 between baseline and day 8 (0.798). Though serial PCT levels significantly increased amongst non-survivors, it did not predict mortality.

**Conclusions:**

Serial level of biomarkers in ICU patients may predict mortality. Larger trials are needed to confirm the results.

## P468 High STREM-1 levels and low monocyte HLA-DR expression are associated with nosocomial infections and mortality in septic shock patients

### A Olivier^1^, L Jolly^1^, G Monneret^2^, T Rimmele^3^, M Salcedo-Magguilli^1^, JJ Garaud^1^, JM Grouin^4^, M Derive^1^, F Venet^2^

#### ^1^INOTREM, Vandoeuvre-les-Nancy, France; ^2^Hospices Civils de Lyon, Immunology Laboratory, Lyon, France; ^3^Hospices Civils de Lyon, Anesthesia and Critical Care Medicine Department, Lyon, France; ^4^University of Rouen, INSERM U1219, Rouen, France

**Introduction:**

TREM-1 is an innate immune receptor which activation promotes the development of exacerbated inflammatory response in septic shock. Plasma levels of soluble TREM-1, a marker of TREM-1 pathway activation, are correlated with patients’ outcome. The role of TREM-1 in the development of immunosuppression in septic shock patients has never been assessed. So far, decreased monocyte HLA-DR expression (mHLA-DR) is the most studied marker of immune alterations in septic shock.

**Methods:**

Plasma sTREM-1 levels were retrospectively measured at Day 1-2, 3-4 and 6-8 in 116 septic shock patients from the IMMUNOSEPSIS cohort (NCT02803346), included between 01/2016 and 12/2018, using a validated ELISA method. The associations between sTREM-1, mHLA-DR, 28-day survival status, and occurrence of ICU-acquired nosocomial infection (NI) were assessed.

**Results:**

Neither sTREM-1 nor mHLA-DR levels at D1/2 were associated with the occurrence of ICU-acquired NI. However, 28-day mortality was significantly higher in patients with D1-2 sTREM-1 value superior to the median (39.6% vs 11.3%, p=0.0103; median=539 pg/mL). A significant inverse correlation was found between mHLA-DR at D6-8 and sTREM-1 at D1-2 (Sp -0.378, p<0.0001) and at D6-8 (Sp -0.382, p<0.0001). At D6-8, when stratifying patients based on sTREM-1 (400pg/mL) and mHLA-DR (5000 AB/C), patients combining elevated sTREM-1 and low mHLA-DR presented with significantly higher 28-day mortality (47.6% vs 8.7%, p = 0.0003, Chi-squared test) and NI incidence (31.8 vs 12%, p=0.044) compared with patients with low sTREM-1 / high mHLA-DR.

**Conclusions:**

This study shows for the first time that TREM-1 pathway activation is associated with septic shock-induced immunosuppression, as shown by an inverse correlation between sTREM-1 at baseline and mHLA-DR expression at D6-8. Persisting high sTREM-1 values and low mHLA-DR expression in septic shock patients are significantly associated with higher rate of ICU-acquired infection and mortality.

## P469 Added value of serial bio-adrenomedullin measurement in addition to lactate for the prognosis of septic patients admitted to ICU

### A Blet^1^, C De Roquetaillade^1^, O Hartmann^2^, J Schulte^2^, J Struck^2^, PF Laterre^3^, E Gayat^1^, A Mebazaa^1^, BG Chousterman^4^

#### ^1^Hôpital Lariboisière, Paris, France; ^2^Shpingotec, Hennigsdorf, Germany; ^3^St Luc university hospital, Brussels, Belgium; ^4^Hôpital Lariboisière, Département d´Anesthésie-Réanimation, Paris, France

**Introduction:**

Sepsis mortality remains high [1]. The Surviving Sepsis Campaign (SSC) recommends to guide resuscitation on normalization of lactate levels [2], however this is debated [3]. We have shown that plasma levels of bio-adrenomedullin (bio-ADM) were associated with patient outcome during sepsis [4]. We therefore aimed to evaluate the added value of bio-ADM to lactate measurement in the AdrenOSS cohort.

**Methods:**

This is a post-hoc analysis of the Adrenomedullin and Outcome in Severe Sepsis and Septic Shock (AdrenOSS) cohort study. The AdrenOSS study is a prospective observational study conducted in twenty-four centers and included 583 septic patients [4]. We studied the relationship between the association of initial evolution of lactate plasma levels and bio-ADM level at 24h and outcome in patients for whom both markers were available at admission and one day later (“24h”). Bio-ADM levels below 70 pg/mL were considered as low, and high if greater than 70 pg/mL [4].

**Results:**

In patients with high lactate levels (>2 mmol/L) at admission (n=328), lactate normalization (<2 mmol/L) at 24h was associated with better outcome than in patients with persistently high lactate at 24h (28-day mortality 15.9% vs 41.9% respectively, HR 3.3 [2.0-5.3], p<0.001) (Figure 1). Among patients with decreasing lactate, high and low bio-ADM levels at 24h identified patients with different outcomes (28-day mortality 7% vs 26% for low vs high bio-ADM respectively, HR 4.4 [1.6-11.7], p<0.005). High and low bio-ADM levels at 24h also differentiated outcome of patients with persistently elevated lactate (HR 4.5 [1.6-12.3], p<0.005). In patients with low initial lactate, neither lactate or bio-ADM had no added prognostic.

**Conclusions:**

Our data suggest that measurement of bio-ADM in addition to lactate may help physicians to refine risk stratification and therefore to guide resuscitation during sepsis.

**References:**

1. Fleischmann C et al. m J Respir Crit Care Med 193:259-72, 2016

2. Levy MM et al. Crit Care Med 46:997-1000, 2018

3. Hernandez G et al. Intensive Care Med 45:82-85, 2019

4. Mebazaa A et al. Crit Care 22:354, 2018

**Fig. 1 (abstract P469).**
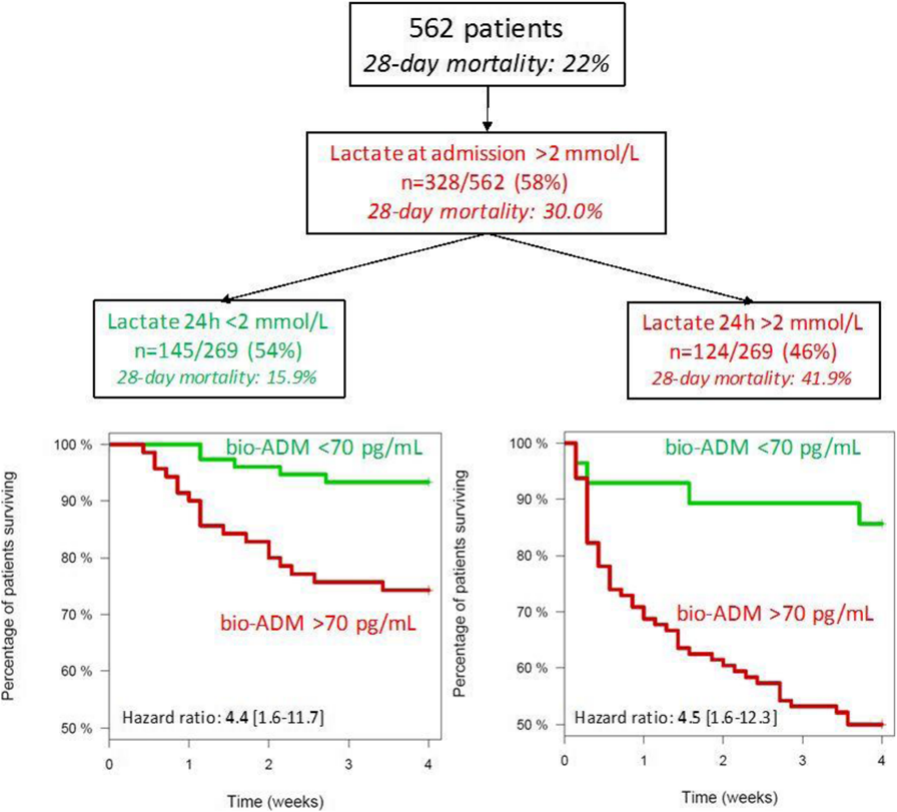
Impact of 24h lactate and bio-ADM values in patients with elevated lactate level at admission. The green curve in the left KM-plot illustrates data from 75 patients with 5 events; the red curve 70 patients with 18 events. The green curve in the right KM-plot illustrates data from 28 patients with 4 events; the red curve 96 patients with 48 events. Of note, differences in numbers between admission (n=328) and 24h (n=269) is related to initial mortality

## P470 The effect of fluid replacement in sepsis, severe sepsis and septic shock in first 24 hrs in clot quality and microstructure

### S Pillai^1^, G Davies^2^, M Lawrence^2^, J Whitley^2^, PR Williams^3^, K Morris^4^, PA Evans^2^

#### ^1^Welsh Centre for Emergency Medicine Research, Emergency Department, Morriston Hospital, Swansea, United Kingdom; ^2^Welsh Centre for Emergency Medicine Research, Morriston Hospital, Swansea, United Kingdom; ^3^Swansea University, Swansea, United Kingdom; ^4^Cardiff Metropolitan University, Swansea, United Kingdom

**Introduction:**

The inflammatory response in sepsis can lead to a spectrum of coagulation system defects [1]. Sepsis and severe sepsis is associated with a hypercoagulable state where the clot microstructure is known to be a tight and highly elastic clot, which is potentially resistant to fibrinolysis (Figure 1). Conversely, septic shock is associated with a hypocoagulable state where the clot microstructure is loose and structurally weak. The study aim to investigate the effect of fluid resuscitation and replacement in clot microstructure over 24 hours.

**Methods:**

100 patients (50 sepsis, 20 severe sepsis and 30 septic shock) were included in the study. All these patients received standard fluid replacement therapy with crystalloids. Blood samples were collected at 0 hours, 4 hours and 24 hours. Clot microstructure, standard markers of coagulation and inflammatory markers were measured.

**Results:**

In sepsis group following fluid administration, the d_f_ reduced initially and then remained stable (1.78- 0 hours, 1.74- 4 hours, 1.73- 24 hours, normal d_f_ range 1.73 ± 0.04). In severe sepsis group, the d_f_ reduced initially, then increased (1.80- 0 hours, 1.71- 4 hours, 1.76- 24 hours) and in septic shock, the df was very low to start with and there were only slight increase with fluid administration (1.66- 0 hours, 1.68- 4 hours, 1.67- 24 hours).

**Conclusions:**

The hypercoagulable state and clot quality in both sepsis and severe sepsis group improved with fluid resuscitation, however despite an early improvement in clot quality, ongoing fluid resuscitation resulted in markedly reduced functional clot with very low clot strength and functionality. This study demonstrates that d_f_ as a marker of clot quality and function may have potential in fluid and component replacement in critical illness and injury.

**References:**

1. Davies et al. Intensive Care Med 42:1990-1998, 2016.

**Fig. 1 (abstract P470).**
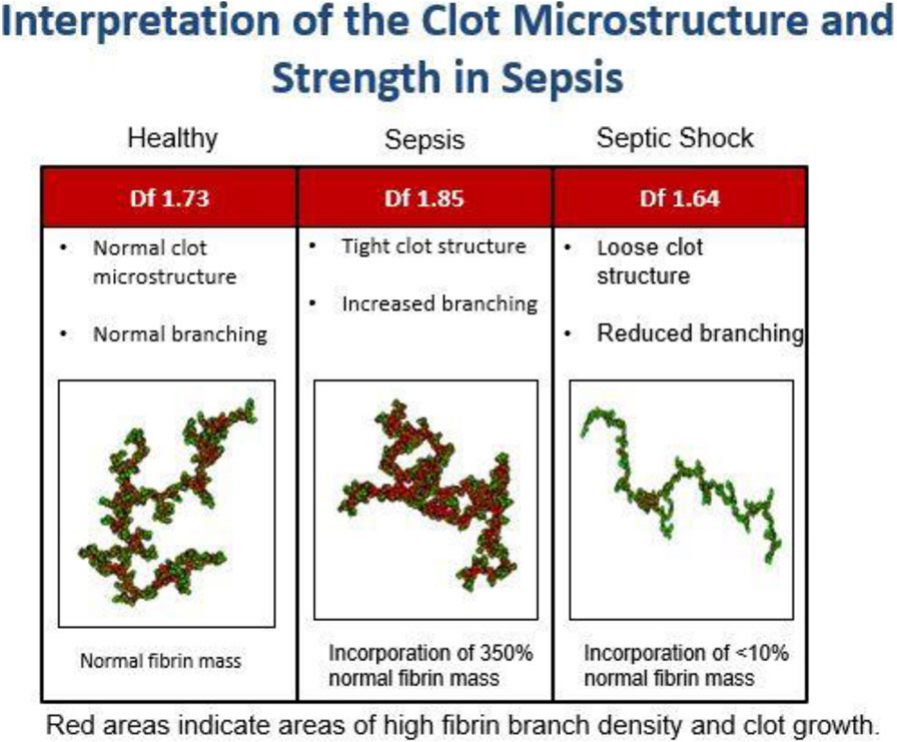
Computer model of clot microstructure in healthy, sepsis and septic shock

## P471 Plasmatic levels of adrenomedullin during extracorporeal membrane oxygenation in patients with severe respiratory failure

### G Grasselli^1^, M Panigada^1^, S De Falco^1^, C Novembrino^2^, F Cea^2^, A Di Modugno^2^, E Cipriani^1^, A Cucino^1^, A Pesenti^1^

#### ^1^Fondazione IRCCS Ca´ Granda Ospedale Maggiore Policlinico, Intensive Care Unit, Milano, Italy; ^2^Fondazione IRCCS Ca´ Granda Ospedale Maggiore Policlinico, Clinical Laboratoy, Milano, Italy

**Introduction:**

Adrenomedullin (AM) is a peptide synthesized in vascular endothelial cells and cleared by the lungs. The use of AM as an inflammatory biomarker and his predictive value has been studied in critically ill patients, but not yet in veno-venous extracorporeal membrane oxygenation (ECMO). The purpose of this study was to describe the plasmatic levels of AM in patients supported with ECMO for acute respiratory failure

**Methods:**

AM (normal values <0.55 nmol/L) was measured at 5 time points: immediately before (T0), 24-h (T1) and 72-h after (T3) ECMO initiation and immediately before (T4) and 72-h (T5) after ECMO removal, in consecutive patients with severe respiratory failure supported with ECMO enrolled in the GATRA study (NCT03208270) at Fondazione IRCCS Ca’ Granda – Policlinico of Milan. Data are reported as median (25^th^ - 75^th^ percentile). Statistical analysis was performed using logistic and random effects regression models (to account for repeated measurements within individuals)

**Results:**

A total of 131 measurements were taken in 32 consecutive patients. AM (nmol/L) decreased along the course of ECMO: T0=2.0 (1.5-6.4), T2=2.0 (1.5-6.4), T3=1.6 (1.1-3.1), T4=1.3 (0.8-2.0), T5=0.9 (0.6-2.1) (mean diff.= -0.65, 95%: CI -0.96, -0.35). AM was lower in patients with viral compared to bacterial ARDS (mean diff.= -2.7, 95%CI -5.2, -0.2) (Figure 1). AM was higher in more severe patients (SOFA>= 10, N=14) compared to less severe patients (sofa<10, N=18): 5.7±4.8 vs 1.4±0.8 nmol/L, respectively p<0.001. Basal values of AM could not predict mortality at 28 days (OR=0.8, 95%CI: 0.5-1.2) after conditioning for SOFA score and respiratory failure etiology

**Conclusions:**

AM plasmatic values seem to be higher in more severe patients and in patients with bacterial ARDS. AM decreased along the ECMO course but could not predict mortality in our group of patients

**Fig. 1 (abstract P471).**
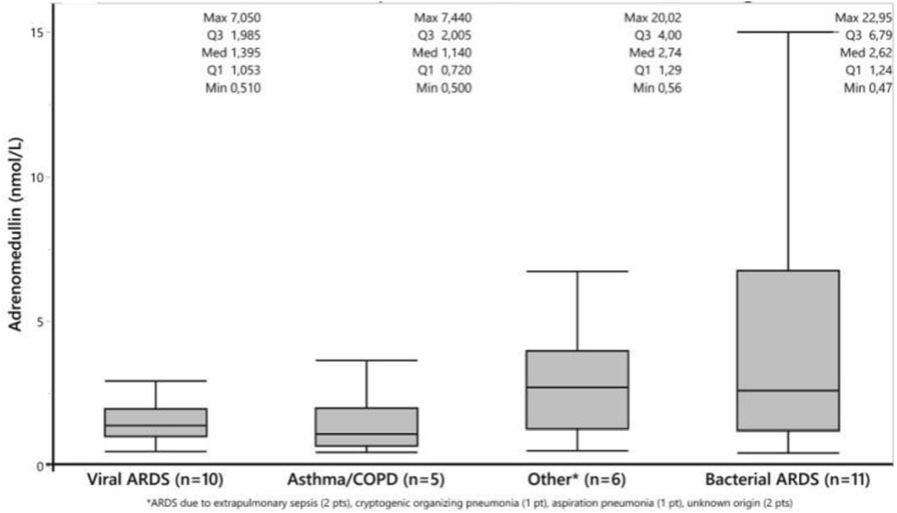
Plasmatic adrenomedullin during ECMO

## P472 Extracellular vesicles are associated with C-reactive protein during sepsis

### B Fendl, R Weiss, T Eichhorn, V Weber

#### Danube University Krems, Department for Biomedical Research, Krems, Austria

**Introduction:**

We characterized the association of C-reactive protein (CRP) with extracellular vesicles (EVs) in plasma from sepsis patients and assessed a commercial CRP adsorbent (Pentrasorb, Pentracor, Hennigsdorf, Germany) to deplete free and EV-associated CRP. In addition, we characterized the potential pro-inflammatory effects of EV-bound CRP on monocytes and endothelial cells.

**Methods:**

The association of EVs with CRP was characterized by flow cytometry and Western Blotting. Plasma CRP levels were quantified using ELISA. To deplete CRP, plasma was incubated with Pentrasorb (10 vol%) for 60 min. Monocytes and human umbilical vein endothelial cells (HUVECs) were stimulated with isolated EVs (20,000 g, 30 min). Monocyte IL-8 secretion was quantified by ELISA; the activation of HUVECs was assessed by their expression of ICAM-1 and E-selectin using confocal microscopy.

**Results:**

Septic plasma (n=30) contained 227.0±88.6 mg/L CRP *vs.* 0.7±0.4 mg/L for healthy controls (n=5). Both, total EVs and CRP^+^ EVs were significantly elevated in septic plasma as compared to healthy controls (14,732±14,657 EVs/μL with 45.9±17.2% CRP^+^ EVs *vs.* 3,741±2,328 EVs/μL with 0.2±0.2% CRP^+^ EVs). Incubation of septic plasma with Pentrasorb resulted in depletion of free CRP (247.2±72.6 mg/L before *vs.* 1.8±0.7 mg/L after adsorption) as well as in a significant reduction in CRP^+^ EVs (15,053±3,992 EVs/μL with 61.0±5.0% CRP^+^ EVs before *vs.* 6,097±1,973 EVs/μL with 1.8±1.3% CRP^+^ EVs after adsorption; n=3). Septic EVs induced a significant release of IL-8 in monocytes as compared to EVs from healthy donors (3,409.0±3,545 pg/mL, n=7 *vs.* 1,333.0±202.9 pg/mL, n=4). EVs from CRP- depleted septic plasma induced significantly lower IL-8 levels. HUVEC ICAM-1 or E-selectin expression, however, did not increase upon stimulation with septic EVs.

**Conclusions:**

Treatment of septic plasma with Pentrasorb efficiently removes free CRP and detaches CRP from the EV surface, resulting in reduced proinflammatory effects.

## P473 Extracellular vesicles from activated platelets induce a shift towards proinflammatory monocyte subsets

### B Fendl^1^, R Weiss^1^, T Eichhorn^1^, A Spittler^2^, V Weber^1^

#### ^1^Danube University Krems, Department for Biomedical Research, Krems, Austria; ^2^Medical University of Vienna, Core Facility Flow Cytometry & Surgical Research Laboratories, Vienna, Austria

**Introduction:**

Circulating monocytes comprise classical (CM, CD14^++^CD16^-^), intermediate (IM, CD14^++^CD16^+^), and non-classical (NCM, CD14^+^CD16^++^) subsets. Changes in subset distribution, specifically a shift towards proinflammatory CD16^+^ monocytes, have been described in various pathologies including sepsis. We analyzed the distribution of monocyte subsets following monocyte isolation from whole blood and the potential influence of platelets and platelet-derived extracellular vesicles (EVs) on monocyte subset distribution.

**Methods:**

Monocyte subsets were characterized by flow cytometry after monocyte isolation by density gradient centrifugation and negative depletion of non-monocytes using two different monocyte isolation protocols (both from Miltenyi Biotec). The association of monocytes with platelets (CD41^+^) and platelet-derived EVs (CD41^+^lactadherin^+^) was assessed by flow cytometry.

**Results:**

Monocyte subset distribution was comparable for both isolation protocols (87.5±4.9% *vs.* 83.7±3.3% CM, 4.8±2.2% *vs.* 5.4±2.8% IM, 7.7±5.8% *vs.* 10.9±5.0% NCM for protocol I *vs.* protocol II; n=4) and did not differ from the distribution in whole blood. Isolated monocytes contained residual platelets and platelet-derived EVs. Overnight storage of isolated monocytes, but not of whole blood, led to a significant increase in IM (86.4±6.2% *vs.* 50.5±11.8% CM, 5.4±2.6% *vs.* 47.1±13.4% IM, and 8.2±4.2% *vs.* 2.4±2.0% NCM at 0h *vs.* 15h). Flow cytometry confirmed the association of monocytes with platelets and platelet-derived EVs as well as the uptake of EVs by monocytes.

**Conclusions:**

Storage of isolated monocytes induces a shift towards CD16 expressing proinflammatory monocytes, which seems to be mediated by residual platelets and platelet-derived EVs. It remains to be clarified whether EVs released from activated platelets can also trigger a shift towards proinflammatory, intermediate monocytes *in vivo*.

## P474 Kinetics of calprotectin, procalcitonin and C-reactive protein in healthy volunteers administered intravenous endotoxin

### JN Fullerton^1^, A Havelka^2^, E Segre^1^, RP De Maeyer^1^, AA Maini^1^, DW Gilroy^1^

#### ^1^University College, Centre for Clinical Pharmacology, Division of Medicine, London, United Kingdom; ^2^Karolinska Institute, Department of Molecular Medicine and Surgery & Gentian Diagnostics AB, Stockholm, Sweden

**Introduction:**

Early recognition of bacterial infection and sepsis is a key step for initiation of antibiotic treatment. Calprotectin is released by neutrophils upon their activation and may thus act as a biomarker of inflammation. This study sought to characterize the kinetics of calprotectin and compare them to established biomarkers following intravenous endotoxin challenge.

**Methods:**

Healthy male volunteers (n=10, mean age 26 years) were administered a bolus injection of 2ng/kg endotoxin (CCRE, E.coli O:113 EC-6). Blood was collected at baseline, 1, 1.5, 2, 4, 6, 8, 24, 48, 72hours and 7 days post injection. Plasma calprotectin was analyzed with a particle enhanced turbidimetric immunoassay (PETIA, Gentian Diagnostics). CRP was analyzed via The Doctors Laboratory, London. Procalcitonin was determined via ELISA (Abcam). Ethical approval was provided by UCL Research Ethics Committee (5060/001). Paired parametric analyses were performed and data displayed as mean +/- 95% CI.

**Results:**

Plasma calprotectin concentration began to increase 1.5hours after endotoxin administration, was significantly higher than baseline by 2 hours (356.7ng/mL vs. 737ng/mL, p <0.01), peaked at 4 hours (mean 1373ng/mL, Figure 1) and normalized by 24 hrs. Calprotectin peaked earlier than comparator soluble mediators (procalcitonin 8hrs, CRP, 24hrs) and exhibited 100% sensitivity; all participants demonstrating a minimum 2-fold increase from baseline (mean 3.84x). Calprotectin displayed greater baseline variability (SD 147.9ng/mL) than either CRP or procalcitonin.

**Conclusions:**

Our results indicate the potential of plasma calprotectin as a biomarker for bacterial infection. It increases earlier and peaks more rapidly than standard biomarkers. Whilst higher baseline variability was observed, all participants exhibited a significant increase from baseline to peak concentration. Plasma calprotectin warrants further investigation as a tool to permit rapid diagnosis and treatment of infection.

**Fig. 1 (abstract P474).**
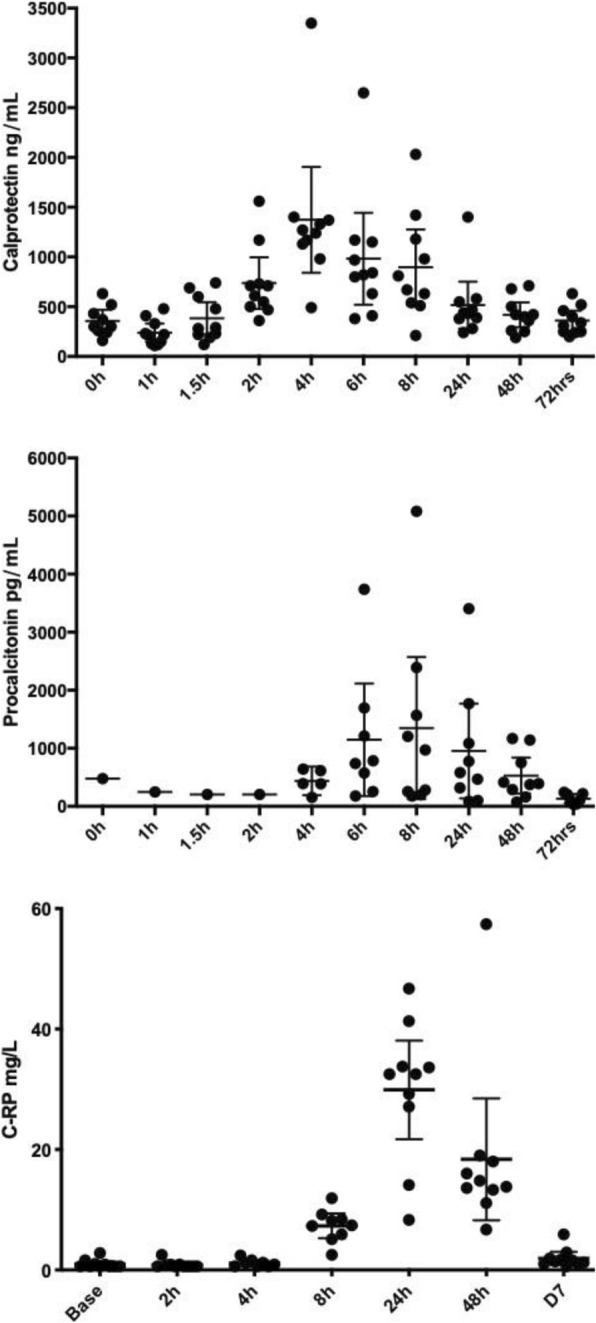
Results

## P475 An analysis of the prognostic ability of the white blood cell count, neutrophil:lymphocyte ratio and C-reactive protein in patients with septic shock

### M Chotalia^1^, M Bangash^2^, T Matthews^2^, D Parekh^2^, J Patel^2^

#### ^1^University Hospitals Birmingham, NHS Foundation Trust, Critical Care and Anaesthesia, Birmingham, United Kingdom; ^2^University Hospitals Birmingham, NHS Foundation Trust, Birmingham, United Kingdom

**Introduction:**

This study analyses the prognostic ability of white blood cell count (WBC), neutrophil:lymphocyte ratio (NLR) and C-reactive protein (CRP). Hypo- and hyperimmune responses have been associated with increased mortality from septic shock [1].

**Methods:**

Patients with septic shock (Sepsis 3.0) admitted to Queen Elizabeth Hospital Birmingham, between December 2017 and July 2019 were included. The primary outcome was 90-day mortality. Data was tested for normality and presented as median (IQR) and analysed using a Mann Whitney U test. Categorical data was presented as % and analysed using a chi-squared test. A p value of < 0.05 was used to determine significance. A multivariate binary logistic regression analysis was conducted using age, APACHE II, charlson comorbidity index, performance status, and initial lactate as covariates. A Hosmer Lemeshow test of >0.05 indicated good fit.

**Results:**

474 patients were admitted with septic shock. The majority (61%) were male, with a median age of 64 (55-75) and a 90-day mortality of 37%. On day 1, WBC was lower in patients who died compared to patients who survived (9 [7-15] vs. 13 [9-21]; p = 0.005). NLR  (8 [3-16] vs. 16 [5-33]; p = 0.001) and CRP (99 [31-177] vs. 170 [91-266]; p <0.0001) were also lower in patients who died compared to survivors. A low WBC on day 1 (<4) was associated with an increased mortality compared to WBC ≥ 4 (45% vs. 35%; p = 0.01).  Multivariate logistic regression analysis identified that day 1 WBC (OR 0.965 [0.925-1.07]; p = 0.1), NLR (OR 0.99 [0.97-1.01]; p = 0.69) and CRP (OR 0.97 [0.95-1.02]; p = 0.93) were not independently associated with mortality.

**Conclusions:**

Patients who died of septic shock had a lower WBC, NLR and CRP response early on compared to survivors.  This may represent early immunoparesis that allows infection to propagate unchecked. However, this was not independently associated with mortality when confounding factors were accounted for.

**References:**

1. Riché F et al. Crit Care 19:439, 2015

## P476 Itaconic acid, other mitochondrial and microbial metabolites in different stages of sepsis

### N Beloborodova^1^, A Pautova^1^, A Sergeev^1^, N Fedotcheva^2^

#### ^1^Federal Research and Clinical Center of Intensive Care Medicine and Rehabilitology, Negovsky Research Institute of General Reanimatology, Moscow, Russia; ^2^Institute of Theoretical and Experimental Biophysics (Russian Academy of Sciences), Pushchino, Russia

**Introduction:**

A specific metabolite of mitochondria – itaconic acid is formed upon pro-inflammatory activation. The attempts of various researches to find the itaconic acid in peripherical blood of patients with sepsis were unsuccessful [1]. Some phenylcarboxylic acids (PhCAs) are known to be microbial metabolites and sepsis biomarkers; they also affect the mitochondrial functions [2].

**Methods:**

Concentrations of PhCAs (phenyllactic, p-hydroxyphenylacetic, p-hydroxyphenyllactic acids) and mitochondrial metabolites (succinic, itaconic acids) in 48 serum samples from 8 patients on the 1^st^ day of diagnosis of sepsis and 35 serum samples from 22 patients with late stages of sepsis (SEPSIS-3) were measured by gas chromatography–mass spectrometry; control group – 20 donors.

**Results:**

Itaconic acid was found in low concentrations (0.5–2.3 μM) only at early stage of sepsis. The multiple increase in levels of PhCAs and mitochondrial metabolites were detected in patients with late stage of sepsis in comparison with early stage and donors, *p<0.001*. Increased succinic acid (up to 100–1000 μM) concentration is the result of succinate dehydrogenase inhibition by microbial metabolism intermediates (PhCAs), which was confirmed by *in vitro* experiments in isolated mitochondria (Fig.1).

**Conclusions:**

Itaconic acid may be a promising marker in early stage of sepsis, which needs to be proved.

**Acknowledgements:** Supported by the Russian Science Foundation Grant 15-15-00110-P.

**References:**

1. Meiser J. Oncotarget 9:32098–32107, 2018

2. Fedotcheva N. Toxycol Lett 180: 182-188, 2008.

**Fig. 1 (abstract P476).**
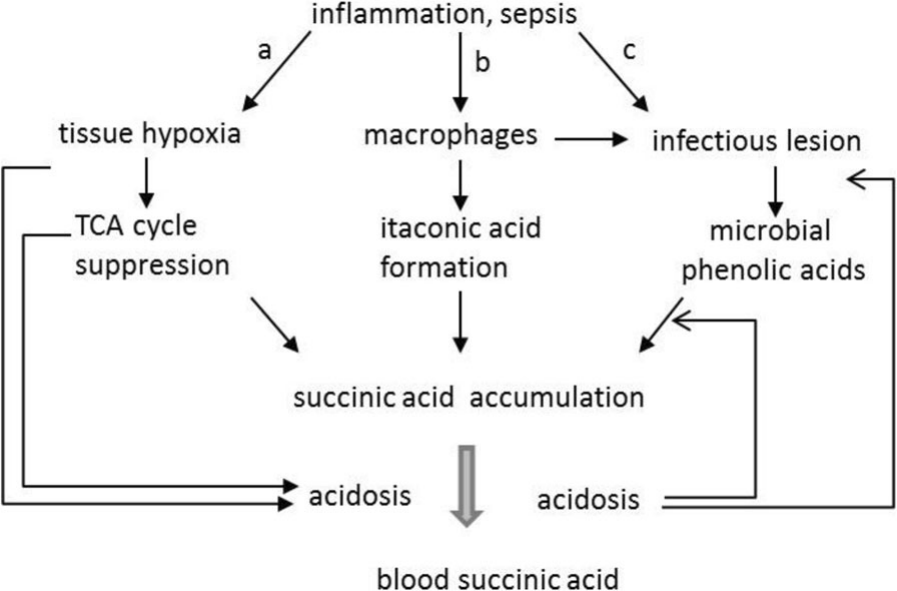
The mitochondrial and microbial metabolites contributing to an increase in the level of succinic acid in the blood of septic patients

## P477 Plasma-calprotectin compared with routine biomarkers for prediction of early severe event in sepsis

### ÅP Parke^1^, DY Yu^2^, CU Unge^3^, JC Sunden-Cullberg^4^

#### ^1^Karolinska Institutet, Department of Medicin, Karolinska Hopital Huddinge, Huddinge, Sweden; ^2^Karolinska Institutet, Department of Laboratory Medicin, Huddinge, Sweden; ^3^Karolinska Institutet, Department of Medicin Huddinge, Huddinge, Sweden; ^4^Karolinska Institutet, Depratment of Medicin, Huddinge, Huddinge, Sweden

**Introduction:**

Prediction of severe events in clinical sepsis is challenging. For such prediction we aimed to compare the novel biomarker calprotectin in plasma, with routine biomarkers.

**Methods:**

In a prospective study, blood samples were collected from consecutive patients who triggered the sepsis alert in the emergency department in our hospital. C-reactive protein (CRP), procalcitonin, neutrophils, and lymphocytes were analysed according to routine practice. P-calprotectin was analysed using a specific particle enhanced turbidimetric assay (Gentian Diagnostics AS). The composite endpoint, which was termed severe event, was defined as death or admission to the intensive care unit (ICU)/high dependency unit (HDU) within 48 hours from arrival.

**Results:**

The study included 367 patients with written informed consent, of whom 335 were considered to have infection (defined as obtained blood culture and subsequent antibiotic therapy for at least 4 days or until discharge or death), and 32 had no infection. Seventy-four patients (22%) with infection developed a severe event. Mean p-calprotectin was 2.99 mg/L (standard deviation (SD) 2.10) among patients with infection and 2.35 mg/L (SD 2.64) among patients without infection (p=0.02). In patients with infection mean p-calprotectin was 3.81 mg/L (SD 3.18) among those with and 2.75 mg/L (SD 2.50) among those without a severe event (p=0.006). Analysis of area under the receiver-operating characteristic (ROC) curve for prediction of severe events showed superiority for p-calprotectin compared with procalcitonin and neutrophil-lymphocyte-ratio, both regarding all sepsis alert cases and regarding the patients with infection (p<0.05 for all comparisons), Fig 1. In addition, there was a trend toward superior performance compared to CRP (p=0.10 and 0.15).

**Conclusions:**

In sepsis alert patients, p-calprotectin was elevated in those who subsequently developed severe events. P-calprotectin was superior to traditional biomarkers for prediction of severe events.

**Fig. 1 (abstract P477).**
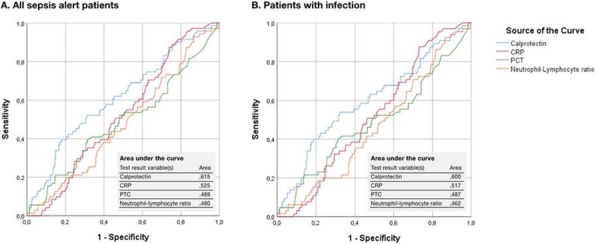
Prediction of early severe event

## P478 A mRNA host response signature accurately distinguishes bacterial and viral infections among emergency department patients

### A Safarika^1^, J Wacker^2^, N Solomonidi^1^, G Giannikopoulos^3^, IM Koutelidakis^4^, O Liesenfeld^2^, T Sweeney^5^, EJ Giamarellos-Bourboulis^1^

#### ^1^14th Department of Internal Medicine, National and Kapodistrian University of Athens,, Athens, Greece; ^2^Inflammatix, Clinical Affairs, Burlingame, United States; ^3^3Department of Internal Medicine, Syros General Hospital,, 3Department of Internal Medicine, Syros General Hospital,, Syros, Greece; ^4^Aristotle University of Thessaloniki, Thessaloniki, Greece; ^5^Inflammatix, Burlingame, United States

**Introduction:**

Rapid diagnosis of acute infections and sepsis is critical in Emergency Departments (EDs). Current tests have slow turnaround times, low sensitivities, and/or signals from contaminant or commensal organisms. Empirical antimicrobial treatment may result in severe adverse events and contributes to antimicrobial resistance. Diagnostics to distinguish bacterial from viral infections and noninfectious etiologies support clinicians in efforts toward antimicrobial stewardship.

**Methods:**

In a prospective, non-interventional study in the EDs of 6 sites in Greece (PROMPT study NCT03295825), we evaluated HostDx Sepsis, a host response test for suspected acute infections and suspected sepsis. HostDx Sepsis measures 29 human mRNA targets and employs advanced machine learning to differentiate patients with bacterial and viral infections, and noninfectious etiologies. Adult patients presenting with suspected acute infection and at least one vital sign change were enrolled. Whole blood RNA was quantified using NanoString nCounter. Predicted probabilities of bacterial and viral infection were calculated (BVN-1 algorithm). Patients were adjudicated in a retrospective chart review by 3 independent infectious disease specialists blinded to HostDx Sepsis results.

**Results:**

Among 396 patients adjudicated as bacterial (56), viral (45), noninfected (1), or indeterminate (294) the Area Under the Receiver Operating Characteristics (AUROC) of HostDx Sepsis for predicting bacterial vs. viral/non-infected patients was 0.92, and AUROC for viral vs. bacterial/non-infected patients was 0.87 (Fig.1).

**Conclusions:**

Our results indicate that HostDx Sepsis distinguishes bacterial from viral infections and other etiologies with high accuracy. HostDx Sepsis is currently developed as a rapid point-of-care device with a turnaround-time of less than 30 minutes. HostDx Sepsis may therefore assist ED doctors in making appropriate treatment decisions earlier, towards the ultimate goal of antimicrobial stewardship.

**Fig. 1 (abstract P478).**
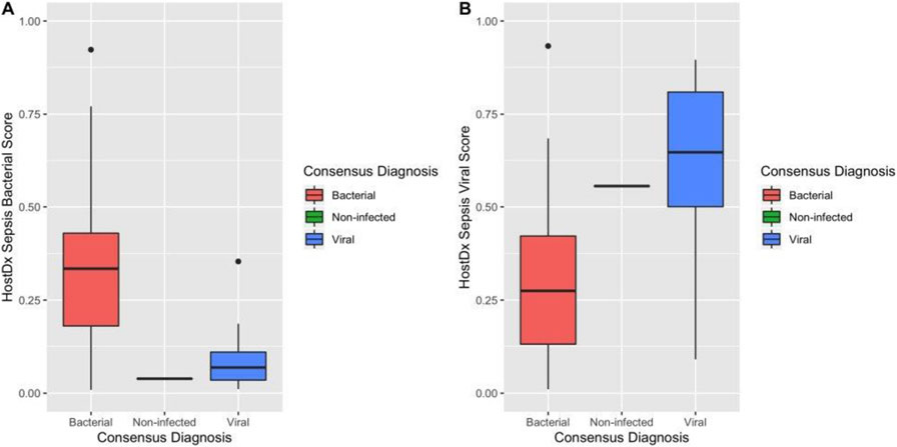
HostDx Sepsis scores for (A) bacterial and (B) viral infection for patients with bacterial, viral, and non-infected status as determined by consensus physician adjudication

## P479 A rapid microfluidic assay of leukocyte deformability demonstrates diagnostic utility for sepsis early in an emergency department (ED) course

### H O´Neal, jr.^1^, A Shah^2^, C Thomas^3^, M Musso^4^, D Hamer^4^, R Sheybani^2^, T Caffery^3^, C Alexander^2^, H Seng^2^, H Tse^2^

#### ^1^Louisiana State University Health Sciences Center New Orleans, Pulmonary and Critical Care, Baton Rouge, United States; ^2^CytoVale, Inc., San Francisco, United States; ^3^Louisiana State University Health Sciences Center New Orleans, Baton Rouge, United States; ^4^Our Lady of the Lake Regional Medical Center, Baton Rouge, United States

**Introduction:**

We studied the diagnostic value of a leukocyte deformability assay that rapidly quantifies the immune activation signatures of sepsis in an undifferentiated population of adults presenting to the ED. ED clinicians must balance the benefits of early intervention against the risks of indiscriminate use of resource-intensive interventions. There are no currently available rapid diagnostics with acceptable performance to achieve this balance.

**Methods:**

We prospectively enrolled adult patients within 5 hours of presentation with signs of suspicion of infection in two EDs in the USA.  EDTA-anticoagulated blood was drawn and analyzed using deformability cytometry [1]. Procalcitonin (PCT) levels were also measured. Patients were retrospectively adjudicated for Sepsis-3 by physician committee using the entire medical record. Diagnostic performance characteristics and receiver operating curves were used to examine the diagnostic performance of the assay as well as PCT.

**Results:**

Of the 190 patients enrolled, 17.4% were adjudicated as septic. The leukocyte deformability assay demonstrated 91% sensitivity, 68% specificity, and 97% Negative Predictive Value for a single cutoff. The AUC was 0.86 (Figure 1). PCT with a cutoff of 0.5 ng/mL had 55% sensitivity, 87% specificity, and 90% Negative Predictive Value. The AUC for PCT (as continuous variable) was 0.8.

**Conclusions:**

The leukocyte deformability assay of immune activation signatures demonstrated superior diagnostic performance for sepsis when compared to PCT. The assay’s diagnostic performance and rapid turnaround time of 5 minutes may positively impact patient outcomes while minimizing indiscriminate use of valuable resources in the ED.

**References:**

1. Crawford K et al. Am J Respir Crit Care Med 198 :280-282., 2018

**Fig. 1 (abstract P479).**
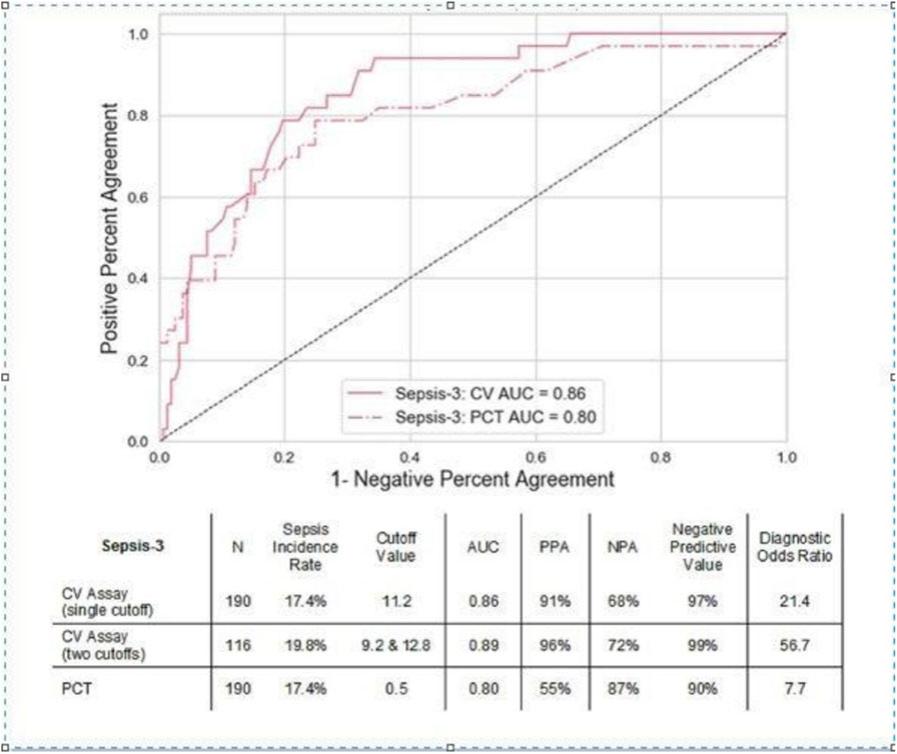
Receiver operating characteristic curve for the CytoVale (CV) assay and PCT

## P480 Midregional proadrenomedullin as a biomarker of microcirculatory and organ disfunction: prospective observational study

### P Giaccaglia^1^, R Domizi^2^, J Montomoli^2^, C Scorcella^3^, A Carsetti^3^, E Damiani^2^, E Casarotta^2^, C D´Angelo^2^, S Bolognini^2^, A Donati^2^

#### ^1^Marche Politechnic University, Anaesthesia and Intensive Care Unit, Ancona, Italy; ^2^Marche Politechnic University, Ancona, Italy; ^3^AOU Ospedali Riuniti, Ancona, Italy

**Introduction:**

It is already known in literature that high levels of midregional proadrenomedullin (MRproADM) are related with organ disfunction in infections despite of source and pathogens [1]. Similarly, microcirculatory impairment has been reported in sepsis. We examine the correlation between microcirculatory disfunction and MRproADM as a sign of early organ failure.

**Methods:**

We included 20 consecutive adult patients with suspected infection, sepsis or septic shock admitted to our Intensive Care Unit (ICU) as first hospital admission with an expected ICU stay of > 24hours. MRproADM was measured daily during the first five consecutive days and sublingual microcirculation was assessed with Incident Dark Field (IDF) technology at T1, T2, and T5. We collected information on SAPS II, APACHE scores, and SOFA score for each timepoint.

**Results:**

Ten patients had septic shock, 5 sepsis and 5 infection. Three patients died during ICU stay. A MRproADM clearance of 20% or more between T1 and T2 was found associated with the improvement of MFI (Mann-Whitney U test, median increase 12.35% versus 2.23%, p=0.005) (Figure 1). A MRproADM >1.42 nmol/l at the ICU admission was associated with a worse SOFA score at all the timepoint. Moreover, MRproADM levels at admission was found significantly related with ICU mortality (AUC 0.941 [0.819-1]; p=0.017). MRproADM shown no relation with absolute value of MFI.

**Conclusions:**

The study shows a good correlation between the clearance of the biomarker and the improvement in MFI. Moreover, our results support previous findings on the prognostic value of MRproADM in terms of SOFA and ICU-mortality.

**References:**

1. Önal U et al. Healthcare 6:110, 2018.

**Fig. 1 (abstract P480).**
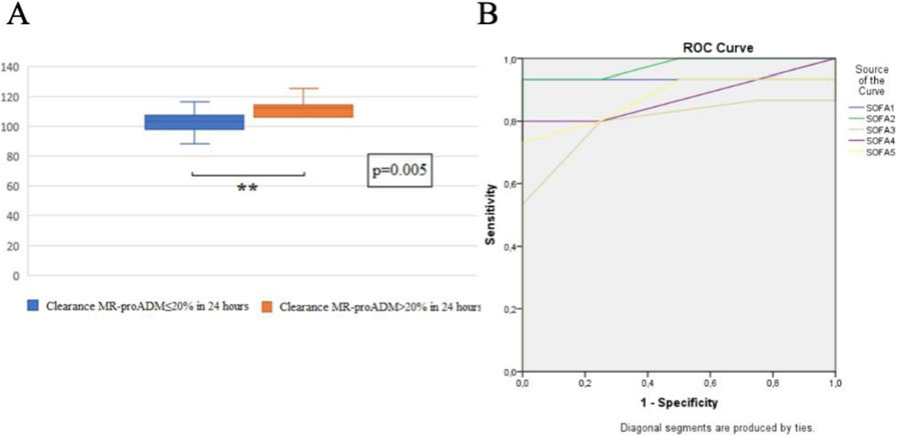
A): Percentage of variation for MFI of small vessels in the first 24 hours of evaluation, in the 2 groups of patients (clearance of MR-proADM inferior-to-equal or higher than 20%). P=0.005 B): ROC curves - SOFA score day1-to-day5 in patients with MR-proADM >1.42 nmol/l at admission in ICU. SOFA-D1: AUC 0.933 [0.807-1], p=0.009; SOFA-D2: AUC 0.975 [0.911-1], p=0.004; SOFA-D3: AUC 0.8 [0.601-0.99], p=0.072; SOFA-D4: AUC 0.875 [0.718-1], p=0.024; SOFA-D5: AUC 0.875 [0.716-1], p=0.024. SOFA score = Sequential Organ Failure Assessment score

## P481 Clinical performance of a rapid sepsis test on a near-patient molecular testing platform

### R Brandon^1^, J Kirk^2^, T Yager^2^, S Cermelli^2^, R Davis^2^, D Sampson^2^, P Sillekens^3^, I Keuleers^3^, T Vanhoey^3^

#### ^1^Immunexpress, Seattle, United States; ^2^Immunexpress, Immunexpress, Seattle, United States; ^3^Biocartis NV, Biocartis, Mechelen, Belgium

**Introduction:**

The purpose of this study was to clinically validate a new, rapid version of the SeptiCyte™ assay on a near-patient testing platform (Biocartis Idylla™). SeptiCyte™ LAB is the first-in-class sepsis diagnostic to gain FDA-clearance but has a complex workflow and a turnaround time (TaT) of ~ 6 hours. The assay in Idylla™ cartridge format is called SeptiCyte™ RAPID.

**Methods:**

SeptiCyte™ LAB was translated to the Biocartis Idylla™ near-patient testing platform and analytically validated. For this study, 0.9mL of peripheral blood PAXgene^TM^ solution from previously collected patient samples was pipetted directly into the cartridge and inserted into the Idylla™ reader. Patients were part of an independent cohort (N=200) from Intensive Care Units located in the USA and Europe. SeptiCyte™ RAPID results were reported as a SeptiScore™ between 0 and 10 with higher scores representing higher probability of sepsis. Assay performance determined included technician hands-on-time (HoT), assay TaT, failure rates, and Area Under ROC Curve based on comparison to retrospective physician diagnosis.

**Results:**

Average HoT was 2 minutes, and average TaT was 65 minutes. Clinical samples could be processed immediately with SeptiCyte™ RAPID and did not require 2 hour pre-incubation of PAXgene blood, greatly improving TaT. Correlation of SeptiScore™ values between LAB and RAPID, based upon a subset of samples run on both platforms, was very high (R^2^>0.97). Estimated ROC AUC performance for discriminating sepsis from non-infectious systemic inflammation (NISI/SIRS) was similar to that previously reported for SeptiCyte™ LAB.

**Conclusions:**

This is the first demonstration of a validated, fully-integrated, rapid, reproducible, near-patient, immune-response sepsis diagnostic, providing actionable results ~ 1 hr, to differentiate sepsis from non-infectious systemic inflammation / SIRS.

## P482 Accuracy of Septicyte™ for diagnosis of sepsis across a broad range of patients

### R Brandon^1^, K Navalkar^2^, D Sampson^2^, R Davis^2^, T Yager^2^

#### ^1^Immunexpress, Seattle, United States; ^2^Immunexpress, Immunexpress, Seattle, United States

**Introduction:**

The purpose of the study was to demonstrate sepsis diagnostic performance of the biomarkers of SeptiCyte™ in subjects other than critically ill adults, and in hospital locations other than ICU. SeptiCyte™ LAB was the first immune-response sepsis diagnostic assay to gain FDA-clearance (K163260) and, as part of gaining this clearance, clinical validation was performed on adult patients admitted to intensive care (ICU) only [1]. We therefore performed an *in silico* analysis across a broad range of patients using the SeptiCyte™ host immune response biomarkers and algorithm.

**Methods:**

Peripheral blood gene expression data, including public and private datasets, were chosen based on quality, annotation, and clinical context for the intended use of SeptiCyte™. Multiple comparisons were performed within datasets to better understand the diagnostic performance in certain cohorts including healthy subjects. Diagnostic performance was determined using Area Under Curve (AUC).

**Results:**

Table 1 shows some characteristics of the selected datasets and patients, including number of datasets (N=22) and comparisons (N=55), number of cases (N=2234) and controls (N=2089) used in comparisons, patient category and hospital location. SeptiCyte™ AUCs for the three groups of adults, adult / pediatric and pediatric / neonates were 0.88, 0.85, and 0.87 respectively, which is similar to that previously reported (0.82 – 0.89) [1].

**Conclusions:**

These results suggest that the SeptiCyte™ signature has diagnostic utility beyond adults suspected of sepsis and admitted to ICU. This signature has now been translated to the near-patient testing platform Biocartis Idylla™ (as SeptiCyte™ RAPID) which promises rapid (~1 hour) diagnosis of sepsis in a broad patient population following further validation.

**References:**

1 Miller III RR et al. Am J Rrespir Crit Care Med 198: 903–913, 2018.

**Table 1 (abstract P482).** Numbers of datasets and comparisons, and patient types used in the analysis

# Datasets / Comparisons	# Case / Controls	Patients	Location	Mean AUC
13 / 35	1640 / 987	Adults	ICU, Ward, ED	0.88
2 / 4	177 / 513	Adults / Pediatric	Ward	0.85
7 / 16	417 / 589	Pediatric / Neonates	ICU, Ward, ED	0.87
Totals				
22 / 55	2234 / 2089			0.87

## P483 Open heart surgery interferes with vascular protective sphingosine-1-phosphate

### G Greiwe^1^, M Winkler^2^

#### ^1^University Medical Center Hamburg-Eppendorf, Department of Anesthesiology, Hamburg, Germany; ^2^University Medicine Göttingen, Department of Anesthesiology and Intensive Care Medicine, Göttingen, Germany

**Introduction:**

Especially extracorporeal cardio pulmonary bypass (CPB) is known to induce severe inflammation. Postoperative inflammation is associated with a sepsis like syndrome including endothelial barrier disruption, volume depletion and hypotension. Sphingosine-1-phosphate (S1P) is a signaling lipid regulating permeability and vascular tone. In septic humans decreased serum-S1P levels could be identified as marker for sepsis severity. We addressed three main issues: (1) Are serum-S1P levels affected by cardiac surgery? (2) Are potential alterations of serum-S1P levels related to changes of acute-phase proteins, S1P sources or carrier? (3) Is the invasiveness of the surgery a factor that may influence serum-S1P levels?

**Methods:**

46 elective major cardiac surgery patients were prospectively enrolled in this study. Serum samples were drawn pre-, post- procedure and on day 1 and day 4 after surgery. We analyzed S1Pand its potential sources: Red blood cells (RBC) and platelets. We further quantified levels of other inflammatory markers and documented other clinical parameters.

**Results:**

Median serum-S1P levels in all patients before the procedure were 0.77 (IQR 0.61-0.99) nmol/ml. Serum-S1P levels decrease after surgery, whereas all other inflammatory markers increase. Serum-S1P levels dropped by 58% in the on-pump and 31% in the off-pump group. Changes of serum-S1P levels are associated with S1P sources and carriers: albumin, HDL and vWF:AG activity. Patients with a full recovery of their serum-S1P levels after surgery compared to their individual baseline presented with a lower SOFA score (P>0.05) and shorter ICU stay (P<0.05).

**Conclusions:**

Serum-S1P levels are disrupted by open heart surgery and levels might be negatively affected by endothelial injury or loss of S1P sources. Low serum-S1P levels may contribute to prolonged ICU stay and worse clinical status. Future studies may investigate the beneficial effects of S1P administration during cardiac surgery.

## P484 Role of neutrophil CD64 (nCD64) and monocytic HLA-DR (mHLA-DR) as newer biomarkers of sepsis management in adult patients admitted in ICU

### K Pandey^1^, SS Nath^1^, M Tripathi^1^, D Malviya^1^, M Harjai^1^, NP Awasthi^2^

#### ^1^Dr. Ram Manohar Lohia Institute of Medical Sciences, Department of Anesthesia and Critical Care, Lucknow, India; ^2^Dr. Ram Manohar Lohia Institute of Medical Sciences, Department of Pathology, Lucknow, India

**Introduction:**

The aim of study is to measure and correlate the expression of nCD64, mHLA-DR, PCT (procalcitonin) and qCRP (quantitative C-reactive protein) to predict development of sepsis and its outcome.

**Methods:**

In this tertiary centre based longitudinal cohort study, a total 110 patients were enrolled in whom sepsis was suspected on the basis of clinical diagnosis and supported by lab investigations. They were divided into two groups sepsis/case and non-sepsis/control. Disease severity in ICU was assessed by Sequential organ failure score (SOFA). Blood samples for routine lab investigations and biomarkers were taken at the time of admission in ICU before administration of first dose of antibiotics at time D_0_/D_1._ Assessment of biomarkers was done simultaneously with TLC at D_0_/D_1_, D_3_ and during follow up of patients till their final outcome.

**Results:**

There was no significant (p>0.05) mean change in PCT, qCRP, SOFA, nCD64, mHLA-DR from Day 1 to Day 3, however, mean change was higher among cases than controls.On comparison of mHLA-DR between the groups across time periods, mHLA-DR was significantly (p=0.0001) lower among septic patients than controls at both Day 1 and Day 3. All biomarker correctly predicted cases among different percentage of patients with different sensitivity and specificity. There was no significant (p>0.05) association of mortality with the study biomarkers except for PCT.

**Conclusions:**

In our study, diagnostic value of PCT in differentiating sepsis from non-sepsis was similar to nCD64 among all biomarkers studied. No advantage of nCD64 or mHLA-DR was found over PCT in diagnosis and correlation with disease progression and mortality.

## P485 AQP4 polymorphism in sepsis: a pilot study in ICU setting

### VM Pisarev^1^, AG Chumachenko^1^, EK Grigoriev^1^, NA Karpun^2^

#### ^1^Federal Research and Clinical Center of Intensive Care Medicine and Rehabilitology, V.A.Negovsky Institute of General Reanimatology, Moscow, Russia; ^2^V.P. Demikhov City Clinical Hospital, Moscow, Russia

**Introduction:**

AQP4 is a water channel protein contributing to astrocyte and immune cells migration, blood-brain barrier maintenance and cell survival [1-2]. AQP4 genetic variants represent biomarkers associating with outcome after traumatic brain injury and intracerebral hemorrhage [3-4]. Linking AQP4 genetic polymorphism to the course of sepsis has not been studied.

**Methods:**

Study cohort included 124 ICU patients diagnosed according to SEPSIS-3 consensus. AQP4 rs11661256 polymorphism was studied by analyzing PCR products in a 2% agarose gel using an AQP4 specific polynucleotide tetraprimer set. Data were analyzed by log rank test (MedCalc 18.11.3), and odds ratios/hazard ratios were computed. Statistical significance was determined by Fisher test (FT) or Mann-Whitney test.

**Results:**

23 of 124 sepsis patients had the minor mutation A for SNP rs11661256 located within the regulatory 3’ region of the *AQP4* gene. Septic shock occurred more frequently in homozygotic carriers of *AQP4* C allele *vs.* patients with AA or CA genotype: OR=3.75 (95%CI: 1.47-9.56), P=0.006 (FT). Lethality in septic shock patients, n=85, significantly increased compared to sepsis patients with no shock, n=39 (82% vs. 20%, P=0.001, FT). Maximum SOFA values were significantly lower in patients with minor allele A compared to CC carriers of (9.6 vs. 12.0, respectively, P=0.008). In post-surgery group of patients, carriers of AC or AA genotypes had significantly increased survival compared to patients with CC genotypes: Chi-square=5.804; HR=0.455 (95%CI: 0.24 -0.863) for lethality; P=0.016 (Figure 1).

**Conclusions:**

Association of minor allele A of AQP4 SNP rs11661256 with survival in sepsis patients seems secondary to linking the SNP to decreased development of multiorgan failure and septic shock that contribute to mortality.

**References:**

1. Tang Y. et al. Exp Neurol 223:485-95, 2010.

2. Nicosia M. et al., Neurol Res 37:657-61, 2015

3. Appelboom G et al. Neurol Res 37:657-61, 2015

4. Dardiotis et al. Neurosci Lett 696:156-161, 2019

**Fig. 1 (abstract P485).**
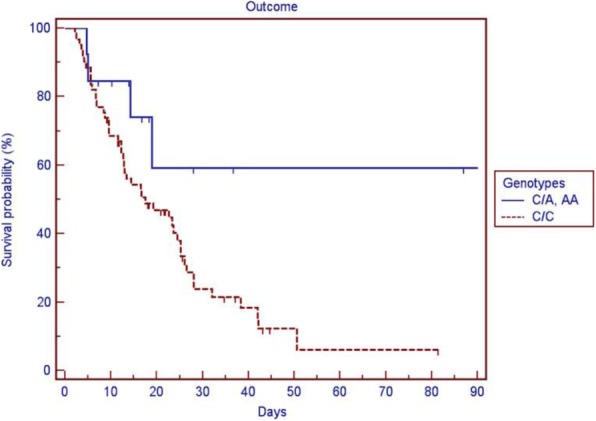
Genotypes AA and AC SNP rs11661256 AQP4 associate with increased survival in sepsis

## P486 Validation of presepsin as a biomarker of sepsis in comparison to procalcitonin, IL-6 and IL-8

### V Chantziara^1^, F Kaminari^1^, C Sklavou^1^, S Fortis^2^, P Kogionou^2^, S Perez^2^, A Efthymiou^1^

#### ^1^Saint Savvas Hospital, ICU, Athens, Greece; ^2^Saint Savvas Hospital, Cancer Immunology and Immunotherapy Center, Athens, Greece

**Introduction:**

Sepsis is an everyday challenge for the intensivist and biomarkers are useful tools for identification and treatment of this syndrome. We sought to validate presepsin as a biomarker of sepsis in comparison to PCT(procalcitonin) and Interleukins (IL-6,IL-8).

**Methods:**

We enrolled 25 patients,18 men and 7 women average age 58 (39.5-74) years old, APACHE II 16 (13.5-20.5), SAPS II 48(40.5-58.5), SOFA 8 (6.5-9). 11 patients were septic on admission (according to Surviving Sepsis Campaign: International guidelines for Management of Sepsis and Septic Shock: 2016), 9 had a septic episode during their hospitalization in the ICU while 5 patients never endured sepsis. We measured presepsin, procalcitonin, IL-6, IL-8 during sepsis and on remission.

**Results:**

All septic patients had increased values of presepsin, PCT, IL-6 and IL-8 during sepsis with a cutoff value for presepsin 800pg/ml, while the values of these biomarkers were significantly decreased during remission or in comparison to non-septic patients(presepsin p = 0.002, PCT p≤ 0.001, IL-6 p≤ 0.001, IL-8 p= 0.004. All patients who were not septic survived while among septic patients 8 died (40% mortality). Presepsin correlated significantly with PCT, IL-6 and IL-8 (p<0.05).

**Conclusions:**

Presepsin is a valid biomarker of sepsis and correlates significantly with all the other values of PCT, IL-6 and IL-8.

## P487 Prospective validation of clinical sepsis phenotypes

### K Demerle^1^, JN Kennedy^1^, OM Peck Palmer^2^, DC Angus^1^, CW Seymour^1^

#### ^1^University of Pittsburgh, Clinical Research, Investigation, and Systems Modeling of Acute Illness (CRISMA), Pittsburgh, United States; ^2^University of Pittsburgh, Division of Clinical Chemistry in the Section of Laboratory Medicine, Pittsburgh, United States

**Introduction:**

Clinical sepsis phenotypes are proposed at hospital presentation. These phenotypes, biomarker profiles, and outcomes are not yet reproduced in prospective data. Even less is known about the biologic mechanism the drives these distinct groups. Thus, we sought to validate clinical phenotypes and to determine markers of innate immunity, coagulation, tolerance and tissue damage in a prospective cohort.

**Methods:**

We prospectively studied patients with Sepsis-3 criteria within 6 hours of presentation at 12 hospitals in Pennsylvania (2018-2019) using automated electronic alerts. Using 29 clinical variables, we predicted phenotypes (*α, β, γ, δ*) for each patient using Euclidean distance anchored to published SENECA phenotype centroids. Discarded blood was analyzed in a subset (n=160) for markers of innate immunity (e.g. IL-6, IL-10), coagulation (e.g antithrombin III, e-selectin), tolerance (e.g. HO-1, IGFBP7), and tissue damage (e.g. serum lactate, bicarbonate)

**Results:**

Among 549 patients, *α*-type was present in 146 (27%), *β*-type in 140 (25%), *γ*-type in 168 (31%) and *δ*-type in 95 (17%, Figure 1a). On average, *β*-type was older and more comorbid (mean 73, SD 9 yrs; mean Elixhauser 4.6, SD 2.2) with renal dysfunction (median creatinine 1.8 [IQR 1.1 – 2.9] mg/dL, p<0.01 all). The *δ*-type had more acidosis (mean HCO3^-^ 20.1, SD 4.7 mEq/L), higher serum lactate (median 1.8 [IQR 1.0-3.5] mmol/L, p <0.01 both) and inpatient mortality (13%, Figure 1b). The *γ*- and *δ*-type had greater markers of innate immunity and abnormal coagulation (e.g IL-6, ICAM p<0.01 both), while markers of increased tissue damage (lactate) and poor tolerance (HO-1) were present in *δ*-type, compared to *α*-type (Figure 1c).

**Conclusions:**

The distribution and characteristics of clinical sepsis phenotypes were reproduced in a prospective validation cohort. Similar to the SENECA study, distinct biomarker profiles of tissue damage, innate immunity and poor tolerance were present for the *δ*-type.

**Fig. 1 (abstract P487).**
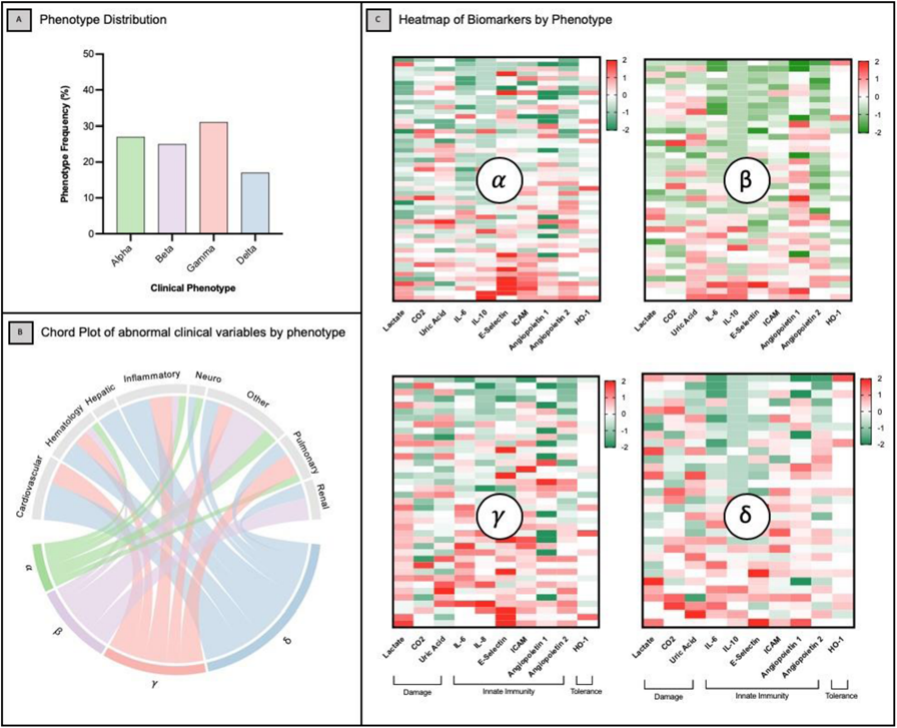
(A) Phenotype distribution (green: α-type, purple: β-type, red: γ-type, blue: δ-type) (B) Chord plot with ribbons connecting individual phenotype to an organ system if the group mean is greater or lesser than the overall mean for the entire cohort. (C) Heatmap showing the log of the fold change of the median biomarker value (column) per patient (row) for various markers of the septic host response grouped by those reflecting tissue damage, innate immunity and tolerance by phenotype. Red represents greater median biomarker value for that phenotype compared to the median of the entire study, while green represents lower values of the biomarker compared to the median of the entire study. White cells are those in which the biomarker was not measured

## P488 HIPEC influences CRP kinetics?

### A Ricardo^1^, J Silvestre^1^, N Abecassis^2^, R Marques^1^, N Candeias^1^, J Nunes^1^

#### ^1^Hospital Lusiadas Lisboa, Intensive Care Unit, Lisboa, Portugal; ^2^Hospital Lusiadas Lisboa, Surgical department, Lisboa, Portugal

**Introduction:**

The effect that neoadjuvant chemotherapy and hyperthermic intraperitoneal chemotherapy (HIPEC) may have in the postoperative kinetics of biomarkers remains unknow. Some studies demonstrate that neoadjuvant chemotherapy and HIPEC do not invalidate the use of inflammatory markers in postoperative patient monitoring, but none have compared biomarkers kinetics between patients who underwent HIPEC or only cytoreduction surgery. Our main purpose was to identify a difference pattern in C-reactive protein (CRP).

**Methods:**

We conducted a single-center observational study from January 2015 to November 2019, including all patients who underwent cytoreductive surgery with or without HIPEC. CRP was measured daily until seven post-operative day. We compared patients with and without HIPEC.

**Results:**

A total of 19 patients were included, 15 were female. Mean age was 63 yrs (44-76). No clinical and demographical differences were observed between groups. No documented infection was found.  After surgery CRP increased markedly in both groups. CRP time-course from the day of surgery onwards was significantly different in HIPEC patients (9.78 ± 3.95 mg/dL vs 14.80 ± 5.63 mg/dL; p=0.035). Multiple comparisons between HIPEC and non HIPEC patients were performed and CRP concentration was significantly different on the 5th and 7th POD (Figure 1). No differences were found in other biomarkers (leucocytes and platelets) neither in body temperature.

**Conclusions:**

After a major elective surgical insult CRP levels markedly increase independently of HIPEC. Serum CRP time-course showed a higher pattern in HIPEC patients despite no infection detected.

**Fig. 1 (abstract P488).**
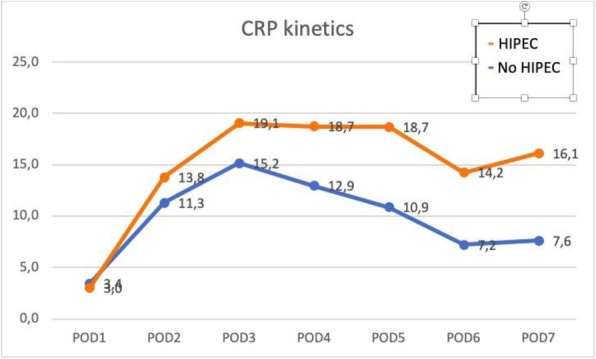
CRP kinetics in cytoreductive surgery

## P489 Decreased thrombin generation potential is associated with increased thrombin generation markers in sepsis associated coagulopathy

### D Hoppensteadt^1^, F Siddiqui^1^, E Bontekoe^1^, R Laddu^1^, R Matthew^2^, E Brailovsky^3^, J Fareed.^1^

#### ^1^Loyola University Medical Center, Pathology, Maywood, United States; ^2^University of Utah, Cardiovascular Institute, Utah, United States; ^3^Loyola University Medical Center, Cardiovascular Institute, Maywood, United States

**Introduction:**

Sepsis associated coagulopathy (SAC) is commonly seen in patients which leads to dysfunctional hemostasis in which uncontrolled protease generation results in the consumption of clotting factors. The purpose of this study is to determine the thrombin generation potential of baseline blood samples obtained from SAC patients and demonstrate their relevance to thrombin generation markers.

**Methods:**

Baseline citrated blood samples were prospectively collected from 49 patients with SAC at the University of Utah clinic. Citrated normal controls (n=50) were obtained from George King Biomedical (Overland Park, KS). Thrombin generation studies were carried out using a flourogenic substrate method. TAT and F1.2 were measured using ELISA methods (Seimens, Indianapolis, IN).  Functional antithrombin levels were measured using a chromogenic substrate method.

**Results:**

The peak thrombin levels and AUC levels were lower in the SAC patients in comparison to higher levels observed in the normal plasma (Table 1). The SAC group showed much longer lag time in comparison to the normal group. Wide variations in the results were observed in these parameters in the SAC group. The F1.2 and TAT levels in the SAC group were much higher in comparison to the normal. The functional antithrombin levels were decreased in the SAC group.

**Conclusions:**

These results validate that thrombin generation markers such as F1.2 and TAT are elevated in patients with SAC. However, thrombin generation parameters are significantly decreased in this group in comparison to normal. This may be due to the consumption of prothrombin due to the activation of the coagulation system. Thus, persistent thrombin generation with simultaneous consumption of clotting factors such as prothrombin contributes to the consumption coagulopathy observed in sepsis patients.

**Table 1 (abstract P489).** Comparison of thrombin generation parameters and markers in normal and SAC plasma samples

Parameters/Markers	Normal Plasma (n=50)	SAC Samples (n=49)
Peak Thrombin (nM)	160 ± 10.78	82 ± 40
Lag Time (min)	2.7 ± 0.20	4.1 ± 2.05
Area Under Curve (nM*min)	700 ± 17.76	561 ± 283
F1.2 (pmol)	570.0 ± 48	210 ± 25
TAT (ng/ml)	27.9 ± 5.1	2.8 ± 0.8
AT (%)	100 ± 20	64.0 ± 11

## P490 Procalcitonin guided blood culture management in the intensive care unit

### H Van leeuwen^1^, M Houterman^2^, R Bosboom^2^, D Hoogeveen^2^

#### ^1^Rijnstate Ziekenhuis, ICU, Arnhem, Netherlands; ^2^Rijnstate Ziekenhuis, Arnhem, Netherlands

**Introduction:**

Procalcitonin (PCT) is used in the ICU as an inflammatory marker to monitor bacterial infections and guide antibiotic therapy. Whether PCT can predict bacteremia and therefore could prevent expenses attached to bloodcultures is unknown . We investigated whether PCT can predict the outcome of blood cultures in the ICU and reduce expences.

**Methods:**

A single centre observational cohort study was performed in a Dutch community teaching hospital . Adult patients who were staying in the ICU and were suspected of bacteremia were included. Simultaneously with drawing of blood cultures, samples for PCT measurement were obtained. Expenses for PCT measurement and bloodcultures were calculated.

**Results:**

In the study period of one year, a total of 120 patients were included. Three patients were excluded because of incomplete data. Out of the 117 included patients, ten patients had positive blood cultures. There was a significant difference in PCT levels between patients who had positive bloodcultures versus patients with negative bloodcultures (8.01 ng/ml vs 0.71 ng/ml) (Figure 1). The negative predictive value for negative blood cultures is 97% when PCT is below 2ng/ml, There was no difference in CRP levels between the two groups (148 mg/l vs 179 mg/l, p= 0.83).A set of negative blood cultures in our centre costs 35 euros. Positive blood cultures however costs significantly more depending on the micro-organisms found. PCT only costs 8.50 euros per measurement. So when blood cultures are omitted when the PCT level is below 2ng/ml, a cost reduction of 38% can be achieved.

**Conclusions:**

A PCT value below 2 ng/ml is a good predictor of a negative blood cultures in ICU patients suspected of bacteremia. PCT guided bloodculture management in these patients could lead to a significant cost reduction

**Fig. 1 (abstract P490).**
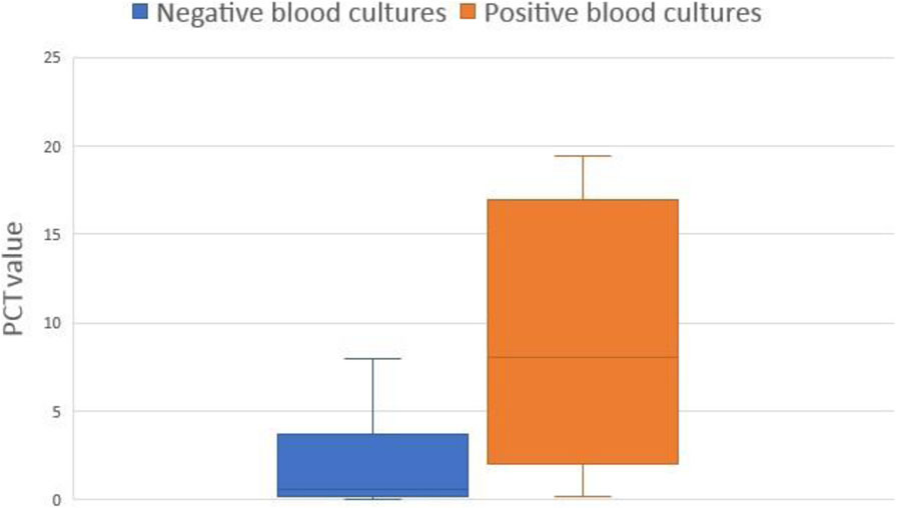
PCT values in patients with positive blood cultures and patients with negative blood cultures

## P491 Circulating plasma cell-free DNA and oxidized cell-free DNA as predictive biomarkers of sepsis in neuroresuscitation ICU patients

### VM Pisarev^1^, AG Chumachenko^1^, EV Elisina^1^, SV Kostyuk^2^, ES Ershova^2^

#### ^1^Federal Research and Clinical Center of Intensive Care Medicine and Rehabilitology, V.A.Negovsky Institute of General Reanimatology, Moscow, Russia; ^2^Research Centre for Medical Genetics, Moscow, Russia

**Introduction:**

Level of cfDNA in plasma is a promising prognostic candidate biomarker in critical illness [1]. Oxidized cfDNA (ocfDNA) have not been studied as a biomarker although its functional role in cellular stress have attracted attention of researches [2]. The goal of our study was to assess the early prognostic value of plasma cfDNA/ocfDNA for sepsis in a NICU setting.

**Methods:**

The cohort included 115 NICU patients diagnosed with stroke, intracerebral hemorrhage (ICH), anoxia, encephalopathy. cfDNA was isolated from day 1 plasma and stained with PicoGreen. Oxidized DNA was determined using DNA immunoblotting with anti-8-oxo-desoxiguanosine antibodies. Genotyping of allelic variants of the TLR9 rs352162 gene was performed using a PCR and designed allele-specific tetraprimers followed by electrophoretic separation of the products Statistics was performed by the Fisher test and Mann-Whitney test.

**Results:**

Sepsis was diagnosed by SEPSIS-3 criteria in 35 patients (30.4%). Average NISU staying was 8,8±11,1 days. Circulating DNA plasma levels on day 1 predicted the future sepsis development (Figure 1): OR for cfDNA was 7.54 (95%CI: 3.03-18.76), P<0.001; OR for ocfDNA was 5.57 (95%CI: 1.64-18.97), P=0.008. Power of both performed tests with alpha=0.05: 1.0. Log rank test demonstrated better predictive value of cfDNA vs. ocfDNA (Figure). Concentrations of cfDNA, but not ocfDNA, on day 1 significantly positively correlated with maximum SOFA values during hospitalization, day 3 and pre-outcome leukocyte count and neutrophil-to-lymphocyte ratios in a limited cohort of NISU patients with TLR9 rs352162 CC genotype and not in other patients with genotype TLR9 CT+TT.

**Conclusions:**

Increased level of plasma cfDNA better then ocfDNA predicts sepsis development in NISU. Further studies are warranted to clarify the possible utility of TLR9 rs352162 polymorphism determining for sepsis risk stratification early on NISU admittance.

**References:**

1. Chornenki J et al. Intensive Care Med Exp. 7:29, 2019

2. Filev AD et al. Oxid Med Cell Longev 2019:1245749, 2019

**Fig. 1 (abstract P491).**
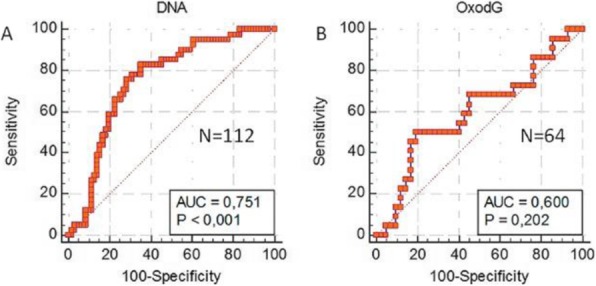
Circulating DNA not oxiDNA predicts sepsis development

## P492 Heparin binding protein as an early diagnostic and prognostic tool in patients with suspected infection

### A Safarika^1^, K Katsaros^2^, N Melachroinopoulos^1^, N Tsokos^3^, P Koutoukas^4^, EJ Giamarellos-Bourboulis^5^

#### ^1^National and Kapodistrian University of Athens, Athens, Greece; ^2^Nafplion General Hospital, Nafplion, Greece; ^3^Halkida General Hospital, Halkida, Greece; ^4^Sparti General Hospital, Sparti, Greece, ^5^National and Kapodistrian University of Athens, 4th Department of Internal Medicine, Athens, Greece

**Introduction:**

Heparin binding protein (HBP) is released from activated neutrophils upon stimulation of b2 integrins. This pro-inflammatory effect generates the hypothesis that it can be a sepsis biomarker for patients admitted at the emergency department (ED)

**Methods:**

The PROMPT study (ClinicalTrials.gov NCT03295825) took place at the ED of six Greek hospitals. Participants were admitted with suspected acute infection and at least one vital sign change. HBP was measured by an enzyme immunosorbent assay in plasma. Sepsis was diagnosed by the Sepsis-3 criteria. The primary study endpoint was the sensitivity for the diagnosis of sepsis. Outcome prediction was the secondary endpoint.

**Results:**

A total of 371 patients were enrolled; 166 had sepsis. The most common infections among patients without and with sepsis were upper respiratory tract infections in 30.2% and 1.2%; community-acquired pneumonia in 6.8% and 28.3%; and acute pyelonephritis in 9.3% and 28.3%. Median HBP was 24.0 and 32.7 ng/ml respectively (p: 0.027). Following analysis of the area under the curve (AUC) it was found that the best discriminatory cut-off for sepsis was 19.8ng/ml. The comparative diagnostic performance of HBP versus qSOFA score is shown in Figure 1. The odds ratio for sepsis with HBP above 19.80 ng/ml was 2.07 (p: 0.001). At the same cut-off point the sensitivity, specificity, positive predictive value (PPV) and negative predictive value (NPV) for the prediction of early death after 72 hours was 100%, 35.7%, 4.1% and 100% respectively.

**Conclusions:**

HBP is more sensitive but less specific than qSOFA for the diagnosis of sepsis in the ED. The rule-out prediction of early death seems the great merit.

**Fig. 1 (abstract P492).**
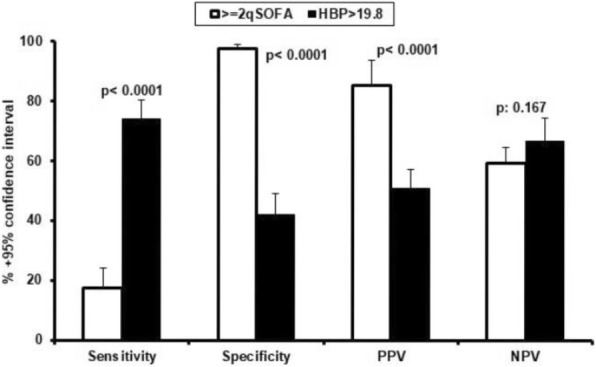
The comparative diagnostic performance of HBP versus qSOFA score

## P493 Chronobiological and recurrence quantification analysis of temperature rhythmicity in critically ill patients

### V Papaioannou^1^, E Sertaridou^1^, I Chouvarda^2^, I Pneumatikos^1^

#### ^1^University Hospital of Alexandroupolis, Intensive Care Unit, Alexandroupolis, Greece; ^2^Aristotle University of Thessaloniki, Faculty of Medicine, Laboratory of Computing, Medical Informatics and Biomedical Imaging Technologies, Thessaloniki, Greece

**Introduction:**

Rhythmicity and complexity of several circadian biomarkers, such as melatonin, cortisol and temperature have been found to be modified by critical illness.

**Methods:**

We examined the potential alterations of core body temperature (CBT) fluctuations and complexity in three groups (N=21): patients with septic shock upon ICU admission (Group A, N=10), patients who developed septic shock at ICU hospitalization (Group B, N= 6) and controls (Group C, N=5). The hourly, average CBT was computed for 24 h upon ICU admission and discharge in Groups A and C, as well as during septic shock onset in Group B. Cosinor analysis of CBT curves was performed leading to the estimation of mesor (mean value), amplitude (the difference between peak and mean values) and acrophase (phase shift of maximum values in hours). Complexity of CBT signals was evaluated with Recurrence Quantification Analysis (RQA).

**Results:**

No significant alterations in any circadian feature within groups were found, except for amplitude. Controls exhibited increased entry CBT amplitude (0.45 ± 0.19) compared to Groups A (0.28 ± 0.18, p < 0.05) and B (0.32 ± 0.13, p < 0.05). Higher entry CBT amplitude in Groups B and C was related with lower SAPS II (r = -0.72 and -0.84, p < 0.003) and APACHE II scores (r = -0.70 and -0.6, p < 0.003) respectively, reduced ICU and hospital stay in Group B (r = -0.62 and -0.64, p < 0.003) and entry SOFA score in Group C (r = -0.82, p < 0.003). Recovery CBT time series appeared more periodic in relation with ICU entry, for all groups. A more random CBT signals pattern upon admission was related with higher severity of illness and extension of ICU stay for all groups.

**Conclusions:**

Reduced CBT fluctuations upon ICU admission was found to more severely ill patients with worse clinical outcomes, while the more periodic CBT patterns were correlated with high CBT rhythmicity and better outcome.

## P494 Sex differences in the epidemiology and mortality of sepsis. The HUNT study

### RM Mohus^1^, MK Moen^2^, T Rogne^3^, TI Nilsen^4^, BO Åsvold^5^, JK Damås^6^, LT Gustad^7^, E Solligård^8^

#### ^1^Clinic of Anaesthesia and Intensive care, St. Olavs hospital, Trondheim, Norway; ^2^Clinic of Anaesthesia and Intensive care, St. Olavs hospital, Clinic of Anaesthesia and Intensive care, St. Olavs hospital, Trondheim, Norway; ^3^Department of Circulation and Medical Imaging, NTNU, Department of Circulation and Medical Imaging, NTNU, Trondheim, Trondheim, Norway; ^4^Department of Public Health and Nursing, NTNU, Departement of Public Health and Nursing, Trondheim, Norway; ^5^Department of Public Health and Nursing, NTNU, Department of public health and nursing., Trondheim, Norway; ^6^Department of Clincal and Molecular Medicine, NTNU, Department of Clincal and Molecular Medicine, Trondheim, Norway; ^7^Clinic of Internal Medicine and Rehabilitation, Levanger Hospital, Helse Nord-Trøndelag Hospital Trust, Clinic of Internal Medicine and Rehabilitation, Levanger Hospital, Helse Nord-Trøndelag Hospital Trust, Levanger, Norway; ^8^Department of Circulation and Medical Imaging, NTNU, Department of Circulation and Medical Imaging, Trondheim, Norway

**Introduction:**

The impact of sex on sepsis incidence and mortality have been elucidated in previous studies, and sex is increasingly recognized as one key factor in sepsis [1]. Some studies indicate that women have better immunologic responses to infections [2]. Later investigations assume this advantage is linked to immune modulating genes located on the X-chromosome [3]. The purpose of this study is to reveal sex differences in incidence of and mortality of sepsis in a large population-based cohort.

**Methods:**

64049 adult participants in the HUNT2 study (1995-97) were followed from inclusion through end of 2011. Incident bloodstream infections (BSI) from all local and regional hospitals in Nord-Trøndelag county were identified through linkage with the Mid-Norway Sepsis Register, which includes prospectively registered information on BSI used as a specific indicator of sepsis. We estimated age-adjusted cumulative incidence of first-time BSI and compared the risk of a first-time BSI and BSI mortality in men and women using age-adjusted Cox proportional hazard regression.

**Results:**

During a median follow-up of 14.8 years 1840 individuals experienced at least one episode of BSI, and 396 died within 30 days after a BSI. Cumulative incidence and cumulative mortality curves are shown in Fig. 1a and 1b. BSI risk was higher among men (HR 1.39 95%CI 1.26–1.52), also BSI mortality was higher among men (HR 1.79 95%CI 1.46-2.19). In analyses of the most common bacteria, men showed higher risk of BSI caused by *S. aureus* (HR 2.09 95%CI 1.58-2.75) and *S. pneumoniae* (HR 1.36 95%CI 1.05-1.76), while *E. coli* did not show sex differences (HR 0.97 95%CI 0.83-1.13).

**Conclusions:**

Male sex is associated with increased risk of BSI and BSI mortality caused by Gram-positive bacteria. Sepsis is a global burden, and it us of urgent importance to reveal the aetiology of this sex disparity in sepsis.

**References:**

1. Sakr Y et al. Crit Care 17:R50, 2013

2. Restif O et al. Proc R Soc 277:2247-2255, 2010

3. Schurz H et al. Hum Genomics 13:2, 2019

**Fig. 1 (abstract P494).**
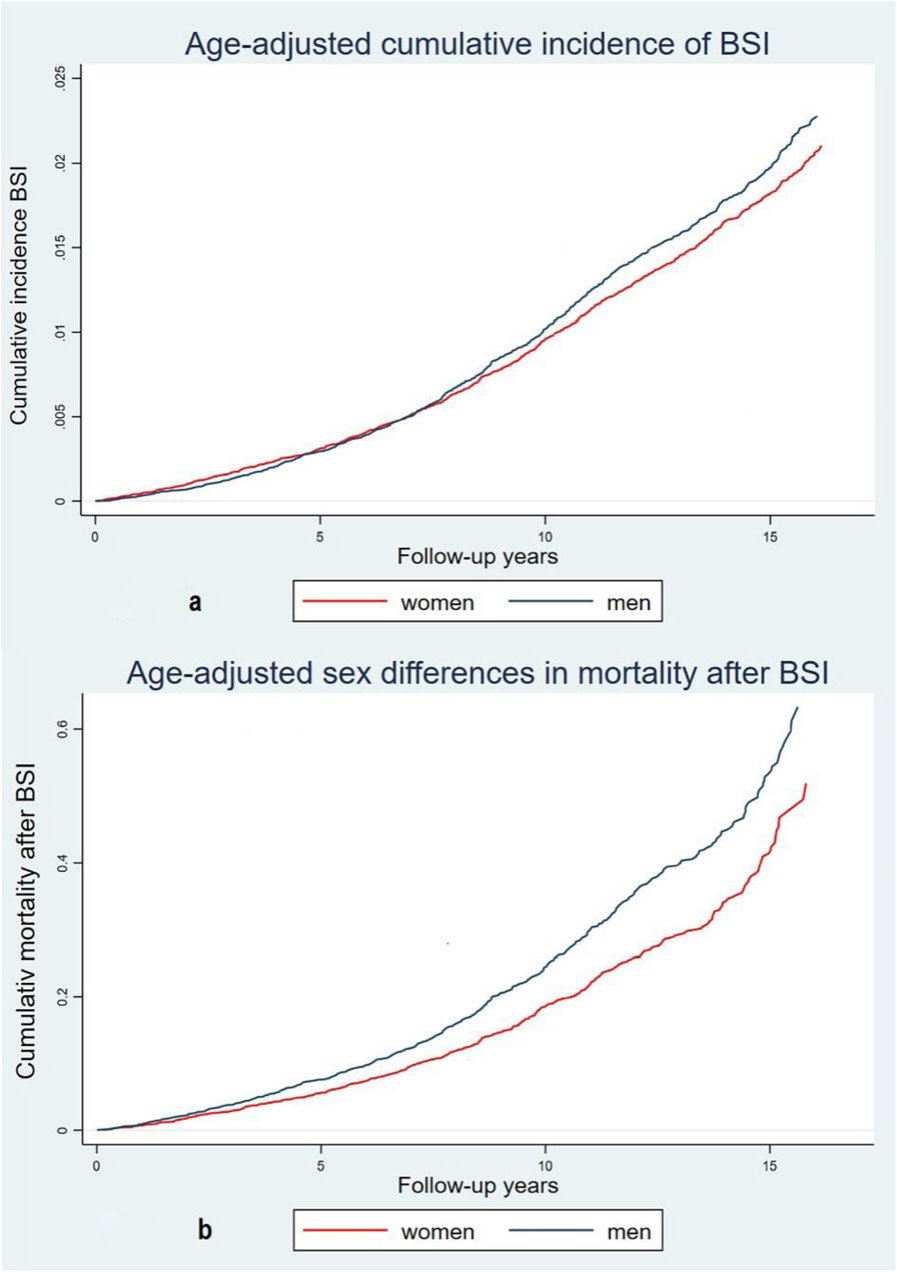
a Age-adjuste cumulative incidence of BSI among men and women. Fig 1b Age-adjusted cumulative mortality in BSI patients, among men and women

## P495 Incidence of hospital-acquired infection in sepsis patients – results from a retrospective cohort study

### A Schettler^1^, M Spoden^2^, L Wedekind^3^, N Rose^1^, A Freytag^4^, P Schlattmann^3^, K Reinhart^5^, C Günster^2^, CS Hartog^5^, C Fleischmann-Struzek^1^

#### ^1^Jena University Hospital, Center for Sepsis Control and Care, Jena, Germany; ^2^Wissenschaftliches Institut der AOK, Forschungsbereich Qualitaets- und Versorgungsforschung, Berlin, Germany; ^3^Jena University Hospital, Institute for Medical Statistics, Computer Science and Data Science, Jena, Germany; ^4^Jena University Hospital, Institute of General Practice and Family Medicine, Jena, Germany; ^5^Charité Universitätsmedizin, Department of Anesthesiology and Operative Intensive Care Medicine, Berlin, Germany

**Introduction:**

The proportion of hospital-acquired infections (HAI) among sepsis patients is unknown in Germany. Systematic differences in HAI foci between sepsis patients with and without ICU treatment are insufficiently described.

**Methods:**

Retrospective cohort study based on nationwide health claims data of the German statutory health insurance AOK. Incident inpatient sepsis cases were identified in 2013/2014 among insured persons >15y without preceding sepsis in 24 months prior to index hospitalization. Sepsis was defined according to explicit sepsis ICD-10-codes (incl. severe sepsis/septic shock). HAI were defined based on specific ICD-10-codes for surgical site infection, catheter-associated urinary tract infection (CAUTI), *Clostridium difficile* (*C. diff*) infections, nosocomial pneumonia, central-line associated bloodstream infection (CLABSI) and other nosocomial infections. ICU treatment was identified by OPS-codes for intensive care complex treatment.

**Results:**

Among 23.011.601 individuals, 159.691 received inpatient treatment for sepsis. 41% had severe sepsis. 21% of sepsis and 28% of severe sepsis patients had an explicitly coded HAI. The proportion of HAI was higher in patients that received ICU-treatment than in patients without ICU-treatment (35% in ICU/14% in Non-ICU sepsis, 37% in ICU/17% in Non-ICU severe sepsis patients). Tab. 1 shows the foci of explicitly coded HAI. Nosocomial pneumonia was the most common HAI in all patient groups. CLABSI occurred more frequently in ICU-treated patients; 21% were affected. CAUTI and *C. diff* infections were more common among Non-ICU-treated sepsis patients. More than one quarter of Non-ICU-treated sepsis patients had a *C. diff* infection.

**Conclusions:**

HAI are common causes of sepsis and pose a significant healthcare burden. The proportion of patients affected and the distribution of foci differ between Non-ICU- and ICU-treated sepsis patients with important implications for sepsis management within hospitals.

**Table 1 (abstract P495).** Foci of explicitly coded HAI in sepsis patients

	ICU-treated Sepsis (n=54,319)	Non-ICU- treated Sepsis (n=105,372)	ICU-treated Severe Sepsis (n=34,999)	Non-ICU-treated Severe Sepsis (n=29,993)
Explicitly coded HAI, n, Among them:	19,091	14,312	12,897	5,206
Nosocomial pneumonia, n, %	10,300 (54%)	4,758 (33%)	7,380 (57%)	2,091 (40%)
Surgical site infection, n, %	1,934 (10%)	1,862 (13%)	1,177 (9%)	533 (10%)
CLABSI, n, %	3,960 (21%)	2,033 (14%)	2,565 (20%)	613 (12%)
CAUTI, n, %	1,124 (6%)	1,416 (10%)	824 (6.4%)	545 (10%)
Clostridium difficile infection, n, %	3,107 (16%)	3,851 (27%)	2,107 (16%)	1,450 (28%)
Other nosocomial infections, n, %	3,825 (20%)	1,904 (13%)	2,478 (19%)	638 (12%)

## P496 Impact of sepsis protocol triggered by Ramathibodi Early Warning Score (REWS) in IPD sepsis on clinical outcomes

### S Matupumanon^1^, Y Sutherasan^2^, D Junhasawasdikul^2^, P Theerawit^3^

#### ^1^Faculty of medicine Ramathibodi hospital, Department of Medicine Ramathibodi Hospital, Mahidol University, Bangkok, Thailand; ^2^Faculty of medicine Ramathibodi hospital, Division of Pulmonary and Pulmonary Critical Care Medicine, Department of Medicine Ramathibodi Hospital, Mahidol University, Bangkok, Thailand; ^3^Faculty of medicine Ramathibodi hospital, Division of Critical Care Medicine, Department of Medicine Ramathibodi Hospital, Mahidol University, Bangkok, Thailand

**Introduction:**

Sepsis is now early identified and managed during triage in the emergency department. However, there is less focus on the effect of patients’ management at the ward level. We aim to evaluate the impact of the implementation of the sepsis protocol on clinical outcomes in in-patients with new-onset sepsis.

**Methods:**

We conducted a prospective observational cohort study among adult medical patients admitted to the general wards in a university hospital. A 25-month pre-protocol period (August 2016 to August 2018) was assigned to a control group, and a 14- month protocol period (September 2018 to October 2019) was allocated to a protocol group. An in-patient sepsis protocol comprised nurse-initiated sepsis protocol by Ramathibodi early warning score (REWS)≥ 2 plus suspected infection, prompt antibiotic, lactate measurement, and fluid resuscitation was implemented.

**Results:**

282 patients were evaluated (141 pre-implementation, 141 post-implementation). 95.7% of sepsis patients have REWS ≥2. More patients in protocol period had lactate measured [90 pts (63.8%) vs. 46 pts (32.6%), P=0.00] and fluid management [51 pts (36.2%) vs.22 pts (15.6%), P=0.00]. More patients in protocol period received antibiotics within 1 hr of presentation compared with pre-protocol period [80 pts (56.7%) vs.53 pts (37.6%), P=0.00]. The length of ICU stay was shorter in sepsis patients in the protocol period [8 days (3, 16.5) vs.10 days (5, 20.5), P<0.05]. There was no difference for in-hospital mortality between both groups (Table 1).

**Conclusions:**

The implementation of in-hospital sepsis protocol was associated with significant improvement in patients’ outcomes, namely lactate measurement, starting antibiotic within 1 hr, fluid management, and the shorter length of ICU stay.

**Table 1 (abstract P496).** Sepsis managements and outcomes

Sepsis managements and outcomes	Pre-protocol n=141	Post-protocol n=141	P
Initial Laboratory measurement n(%)	138(97.9)	140(99.3)	0.32
Initial lactate measurement n(%)	46(32.6)	90(63.8)	0.00
Blood culture taking before antibioticsn(%)	137(97.2)	140(99.3)	0.18
Time to antibiotics less than 1 hour(%)	53(37.6)	80(56.7)	0.00
Fluid management n(%)	22(15.6)	51(36.2)	0.00
ICU Length of stay,days, median (IQR)	10.0 (5.0 – 20.5)	8.0 (3.0 – 16.5)	0.01
In-hospital mortality, n(%)	51(36.2)	44(31.2)	0.39

## P497 ICU routine nursing procedures interfere with cerebral hemodynamics in a prolonged porcine fecal peritonitis model

### SL Liu^1^, DC Casoni^2^, W Z’Graggen^3^, D Bervini^3^, D Berger^1^, SJ Jakob^1^

#### ^1^Inselspital, Department of Intensive Care Medicine, Bern, Switzerland; ^2^2Experimental Surgery Facility, Department for BioMedical Research, University of Bern, Experimental Surgery Facility, Department for BioMedical Research, Bern, Switzerland; ^3^Inselspital, Department of Neurosurgery, Bern, Switzerland

**Introduction:**

Routine nursing procedures (NP) can interfere with blood pressure and cardiac output and may therefore alter cerebral hemodynamics in critical illness. This may be risk factor of sepsis-associated encephalopathy.

**Methods:**

20 sedated and mechanically ventilated pigs were randomized to fecal peritonitis or controls (n=10, each). After 8 hours of untreated peritonitis, the animals were resuscitated for 76 hours (resuscitation period). NP [assessment of sedation (AS), tracheal suctioning (TS), change in body position (CP), lung recruitment maneuver (RM)] were performed at baseline and 8h, 32h, 56h and 72h after start of RP. Systemic and cerebral hemodynamics and O_2_ saturations were recorded continuously.

**Results:**

After sepsis induction, mean arterial pressure (MAP) decreased by 15 (7-22) mmHg, cardiac output by 1.3 (0.7-2.2) L/min and arterial lactate increased by 0.2 (0.1-0.5) mmol/L. Intracranial pressure (ICP) was not affected. In controls, NP decreased MAP (max. 23±11mmHg) and increased ICP (max. 3.0±1.4mmHg). Cerebral perfusion pressure (CPP, max. 26±11mmHg) and carotid arterial flow decreased (max. 79±27ml/min). Thus, cerebral O_2_ delivery (DO_2_, max. 0.58±0.33ml/min/kg) and superior sagittal sinus O_2_ saturation (SssO_2_, max. 20±15%) decreased markedly, despite maintained arterial O_2_ saturation (all p<0.05). The alterations recovered within 1 min after end of NP. The effects of NP on CPP, DO_2_ and SssO_2_ were attenuated during the resuscitation period. NP with most impact on SssO_2_ were TS, CP and RM. In septic animals, NP led to similar changes as in controls. Higher SssO_2_ values in sepsis – both before and after NP - were the result of numerically higher cerebral DO_2_ and lower O_2_ consumption (VO_2_).

**Conclusions:**

NP impaired cerebral O_2_ transport similarly in control and in septic animals, but the changes were short lasting and attenuated during the course of treatment. In septic animals, cerebral VO_2_ was numerically lower than in controls, despite numerically higher DO_2_.

## P498 Elevated renin and acute respiratory distress syndrome in patients with vasodilatory shock treated with angiotensin II

### LW Busse^1^, C Galloway^2^

#### ^1^Emory University, Department of Medicine, Atlanta, United States; ^2^La Jolla Pharmaceutical Company, Medical Director, Critical Care, San Diego, United States

**Introduction:**

Elevated renin is associated with an increased risk of death in patients with vasodilatory shock (VS). Recent data show that patients with VS and elevated renin levels have improved survival when treated with angiotensin II (Ang II) + standard care (SC) vs placebo + SC. Patients with acute respiratory distress syndrome (ARDS) can develop angiotensin-converting enzyme (ACE) defects that can lead to elevated renin levels and insufficient endogenous Ang II production. We hypothesized that patients with severe ARDS and elevated renin shock would have improved survival when treated with Ang II + SC vs placebo + SC.

**Methods:**

In the randomized, placebo-controlled, double-blind ATHOS-3 study, 321 patients with severe VS receiving >0.2 μg/kg/min of norepinephrine or the equivalent were randomized to intravenous Ang II (n=163) or placebo (n=158). In a post hoc analysis, we assessed the subset of patients with elevated renin (defined as a renin level greater than the median value of the overall ATHOS-3 population) and ARDS (defined by a PaO2/FiO2 ratio <300) at the time of randomization. Survival to 28 days was compared between the Ang II group (n=41) and the placebo group (n=61).

**Results:**

In patients with elevated renin and ARDS, baseline age, Acute Physiology and Chronic Health Evaluation II score, and blood pressure were similar in the Ang II and placebo groups. The median serum renin level was 459.5 pg/ml (IQR: 285.8-1036.0) compared to the normal range for serum renin: 5-58 pg/ml. A significantly higher proportion of patients receiving Ang II survived to day 28 compared to those in the placebo group (51% vs 31%; p=0.01).

**Conclusions:**

Elevated renin identified patients with VS and ARDS who were most likely to gain a survival benefit from Ang II. Elevated renin is likely caused by an ACE defect and may describe an important subset of patients with a biotype that responds well to Ang II therapy.

## P499 Elevated renin and acute kidney injury in patients with vasodilatory shock treated with angiotensin II

### M Ostermann^1^, C Galloway^2^

#### ^1^Guy’s & St Thomas’ Hospital, Dept of Critical Care, London, United Kingdom; ^2^La Jolla Pharmaceutical Company, Medical Director, Critical Care, San Diego, United States

**Introduction:**

Elevated renin levels have been shown to be associated with an increased risk of death and more severe acute kidney injury (AKI) in patients with vasodilatory shock (VS). Recent data show that patients with VS and elevated renin levels have improved survival when treated with angiotensin II (Ang II) + standard care (SC) vs placebo (PBO) + SC. We hypothesized that VS patients with severe AKI and elevated renin levels would have improved survival and enhanced renal recovery with Ang II treatment.

**Methods:**

In the randomized, PBO-controlled, double-blind ATHOS-3 study, 321 patients with severe VS received >0.2 μg/kg/min of norepinephrine or the equivalent and were randomized to intravenous Ang II + SC (n=163) or PBO + SC (n=158). In a post hoc analysis, we assessed the subset of patients with elevated renin (defined as a renin level greater than the median value of the overall ATHOS-3 population) and severe AKI (defined as those with AKI requiring renal replacement therapy [RRT] at baseline). Survival and renal recovery were assessed in patients treated with Ang II + SC (n=45) and PBO + SC (n=60).

**Results:**

In patients with elevated renin and severe AKI, baseline age, Acute Physiology and Chronic Health Evaluation II score, and blood pressure were similar between Ang II + SC vs PBO + SC. The median baseline serum renin level in the whole group was 352.5 pg/ml (IQR: 115.9-785.4; normal range for serum renin: 5-58 pg/ml). A significantly higher proportion of patients receiving Ang II + SC vs PBO + SC survived to day 28 (43% vs 22%, respectively; p=0.03). Ang II recipients also had a higher rate of discontinuation from RRT by day 7 (43% vs 12%; p=0.04).

**Conclusions:**

In this study, elevated-renin shock patients with AKI treated with Ang II + SC gained a survival benefit and earlier discontinuation from RRT compared to those receiving PBO + SC. Elevated renin is likely caused by an angiotensin-converting enzyme defect and may identify those patients with a biotype that responds well to Ang II therapy.

## P500 Machine-learning methods for predicting the progression of sepsis-induced coagulopathy

### D Hasegawa^1^, K Yamakawa^2^, K Nishida^3^, O Nishida^4^

#### ^1^Fujita Health University School of Medicine, Anesthesiology and critical care medicine, Toyoake, Japan; ^2^Osaka General Medical Center, Division of Trauma and Surgical Critical Care, Osaka, Japan; ^3^Nagoya University Graduate School of Medicine, Department of Biostatistics, Nagoya, Japan; ^4^Fujita Health University School of Medicine, Department of Anesthesiology and Critical Care Medicine, Toyoake, Japan

**Introduction:**

Most clinical trials conclude the ineffective use of anticoagulation for sepsis-induced coagulopathy [1]. However, *post hoc* analyses of randomized control trials report positive results [2], suggesting anticoagulation is effective in specific populations exhibiting coagulopathy. Further, anticoagulants should be administered in the early phase [3]; however, methods for precisely predicting the progression of sepsis-induced coagulopathy are not established. This study aimed to create and evaluate a prediction model of coagulopathy progression using machine-learning techniques.

**Methods:**

We performed a subgroup analysis of data from a retrospective cohort study involving adult septic patients in 40 Japanese institutions from January 2011 to December 2013 and used the Japanese Association for Acute Medicine disseminated intravascular coagulation (DIC) score as a DIC severity index test. The predictive ability of ΔDIC ([DIC score on Day 3] – [DIC score on Day 1]) was evaluated using various statistical methods. Using variables available at the outset, we compared the predictive ability of Random Forest (RF) and Support Vector Machine (SVM) with that of multiple linear regression analysis.

**Results:**

A total of 1110 adults with sepsis were included in the analysis. The Root Mean Square Error in ΔDIC score for the multiple linear regression analysis model was 2.1168 compared with values of 1.6508 and 1.9394 for RF and SVM, respectively. Thus, the RF method predicted the progression of sepsis-induced coagulopathy more accurately than multiple linear regression analysis.

**Conclusions:**

RF, a machine-learning technique, was superior to multiple linear regression analysis in predicting the progression of sepsis-induced coagulopathy. This prediction model might enable us to use anticoagulation in an early phase.

**References:**

1. Vincent JL et al. JAMA 321:1993–2002, 2019

2. Yamakawa K et al. Crit Care 23:302, 2019

3. Hasegawa D et al. Crit Care 23:231, 2019

## P501 A multicenter randomized controlled study on landiolol for the treatment of sepsis-related tachyarrhythmia: subanalysis of the J-Land 3S study

### O Nishida^1^, Y Kakihana^2^, M Okajima^3^, T Taniguchi^3^, H Morimatsu^4^, H Ogura^5^, Y Yamada^6^, T Nagano^7^, E Morishima^8^, N Matsuda^9^

#### ^1^Fujita Health University School of Medicine, Department of Anesthesiology and Critical Care Medicine, Toyoake, Japan; ^2^Kagoshima University Graduate School of Medical and Dental Sciences, Department of Emergency and Intensive Care Medicine, Kagoshima, Japan; ^3^University Hospital, Kanazawa University, Intensive Care Unit, Kanazawa, Japan; ^4^Okayama University Graduate School of Medicine, Dentistry and Pharmaceutical Sciences, Department of Anesthesiology and Resuscitology, Okayama, Japan; ^5^Osaka University Graduate School of Medicine, Department of Traumatology and Acute Critical Medicine, Osaka, Japan; ^6^The University of Tokyo Hospital, Department of Anesthesiology and Pain Relief Center, Tokyo, Japan; ^7^Ono Pharmaceutical Co., Ltd., Clinical Development Planning, Osaka, Japan; ^8^Ono Pharmaceutical Co., Ltd., Data Science, Osaka, Japan; ^9^Nagoya University Graduate School of Medicine, Department of Emergency & Critical Care Medicine, Nagoya, Japan

**Introduction:**

This study examined the efficacy and safety of landiolol, an ultra-short-acting β1-blocker, for treating sepsis-related tachyarrhythmia, according to patient background characteristics.

**Methods:**

The J-Land 3S study (JapicCTI-173767) was conducted in patients with sepsis, diagnosed according to the Sepsis-3 criteria, and tachyarrhythmia (atrial fibrillation, atrial flutter, or sinus tachyarrhythmia). The patients had a mean heart rate of ≥100 beats/min and required catecholamine administration to maintain a mean blood pressure of ≥65 mmHg. The efficacy endpoint was the percentage of patients whose heart rate could be controlled within 60–94 beats/min at 24 h of registration. The safety endpoint was the incidence of adverse events within 168 h of registration. Subgroup analyses of efficacy and safety were performed after stratifying the patients according to various patient background characteristics.

**Results:**

A total of 151 patients were randomized, 76 to landiolol and 75 to the control group. The efficacy endpoint, percentage of patients with a heart rate of 60–94 beats/min at 24 h of registration, was significantly higher in the landiolol group (54.7% vs 33.3%; Mantel–Haenszel test: p = 0.0031). The incidence of adverse events was 63.6% and 59.5% in the landiolol and control groups, respectively, and there was no difference between the two groups. Most adverse events were related to sepsis or septic shock. The subgroup analyses showed that no patient background characteristic clearly affected the efficacy and safety of landiolol.

**Conclusions:**

Landiolol is a well tolerated and effective therapeutic agent for controlling heart rate in patients with sepsis-related tachyarrhythmias; its safety and efficacy were not affected by the patient background characteristics investigated.

## P502 Tissue oxygenation monitoring in sepsis

### R Marinova, AT Temelkov

#### UMHAT Alexandrovska, Anesthesiology and intensive care, Sofia, Bulgaria

**Introduction:**

Near-infrared Spectroscopy (NIRS) was proposed as a concept in the end of 20th century. This method offers noninvasive monitoring of oxy- and deoxyhemoglobin in tissues.NIRS could be measured on the thenar or forehead within few santimeters of the skin. It was first applied as a monitoring in cardiovascular surgery.

Patients with sepsis have changes in the microcirculation which are important target for therapy. Invasive monitoring of oxygen delivery and consumption has been used in patients with sepsis but as every invasive technique such a monitoring hides risks. NIRS offers a noninvasive method for tissue oxygenation monitoring (StO2) and could be useful in patients with sepsis and septic shock. The aim of the study is to compare noninvasive tissue oxygenation monitoring with hemodinamic monitoring and lactate values in patients with sepsis

**Methods:**

The study includes 19 critically ill patients in ICU of UMHAT Alexandrovska, Sofia. 10 of the patients fullfil the criteria for septic state. The other 9 patients do not have sepsis. In both group of patients are measured tissue oxygenation with INVIOS monitor, mean arterial pressure, oxygen saturation in mixed venous blood and lactate values during 72 h after ICU admission.

**Results:**

Patients with sepsis are reported with significantly lower values of tissue oxygenation, compared to patients without sepsis. The values of tissue oxygenation correlate well with the mixed venous blood oxygenation, mean arterial pressure and lactate values but not significantly with APACHE scores.

**Conclusions:**

NIRS when used for tissue oxygenation monitoring correlates well with the hemodinamic monitoring and lacate values in patients with sepsis and could be used as an noninvasive monitoring for guiding teurapeutic strategies. Tissue oxygenation monitoring has no linear correlation with the severity of illness in patients with sepsis and could not be reccomended as a guidance in the early ressuscitating stage of sepsis. Further investiganions in these field are needed.

## P503 Digital phenomenology of early sepsis care

### EB Brant^1^, JN Kennedy^1^, IJ Barbash^2^, CH Chang^3^, DC Angus^1^, CW Seymour^1^

#### ^1^University of Pittsburgh School of Medicine, Department of Critical Care Medicine, Pittsburgh, United States; ^2^University of Pittsburgh School of Medicine, Division of Pulmonary, Allergy and Critical Care Medicine, Pittsburgh, United States; ^3^University of Pittsburgh School of Medicine, Department of Biostatistics, Pittsburgh, United States

**Introduction:**

Digitization of health data enables development of virtual care providers. In early sepsis care, development of such technologies, through reinforcement learning and other strategies, first requires understanding the sequence of vital sign or laboratory measurements and treatments. Thus, we explored patterns of data availability and intravenous (IV) fluid/vasopressor administration in a sepsis cohort.

**Methods:**

We analyzed a retrospective cohort of electronic health records from adult sepsis patients at 12 UPMC hospitals from 2010 to 2014. We defined Sepsis-3 by i.) suspected infection (e.g., administration of antibiotics or body fluid culture) & ii.) organ dysfunction (e.g., 2 or more SOFA points) in the first 6 hours of care. Data were organized by hour and included vital signs, lab values, and treatments (e.g., total hourly IV fluids (mL) and norepinephrine equivalent dose). For each hour we describe, i.) available data elements, ii.) presence of Sepsis-3, and iii.) treatment patterns.

**Results:**

We studied 71,272 sepsis patients (median age 68, [IQR, 56-81] years; 34,384 [48% male]. During Hour 1, vital signs were measured in 70% (n=49,890), lab values (e.g. complete blood count) in 51% (n= 36,348), fluid culture in 46% (n=32,785) and serum lactate in 10% (n=7,172, Fig1A). By Hour 6, most patients had vital signs (99%; n=70,559), basic labs (88%; n=62,719), fluid cultures (94%, n= 66,995), while serum lactate was completed in 24% (n=17,818). These data contributed to Sepsis-3 criteria in Hour 1 in 78% (n=55,592) and 100% by hour 5 (Fig1B). Amidst this changing background, IV fluids were given in 17% (mean volume, 166 mL [SD, 411]) and vasopressors in 1% (n=712) in Hour 1. By Hour 6, all received fluids, (mean volume, 1170 mL [SD, 979]), and 80% (n=57,018) received a vasopressor (Fig1C).

**Conclusions:**

Early sepsis care patterns are variable. IV fluids were given during early hours, when uncertainty about sepsis was greatest, while vasopressors were administered after Sepsis-3 elements were present.

**Fig. 1 (abstract P503).**
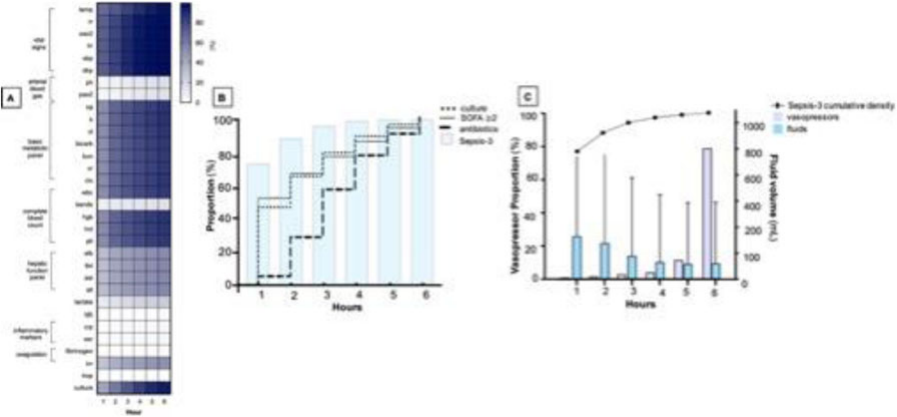
(A) Heatmap of available data elements per hour. X-axis represents hour after hospital arrival; y-axis represents physiologic or laboratory variable. Dark blue corresponds to no missing variables; white corresponds to 100% missing. (B) Time-to-event plot of Sepsis-3 elements, overlaid on cumulative proportion of patients with Sepsis-3. X-axis is hour after hospital arrival. (C) Patterns of IV fluid and vasopressor prescriptions per hour. X-axis represents hour after hospital arrival. Left y-axis corresponds with line graph and purple bars, which represent the cumulative density of sepsis patients and proportion of patients receiving vasopressors, respectively. Right y-axis corresponds with blue bars and represents mean IV fluid volume administered per hour; error bars represent standard deviation

## P504 Effects of abdominal negative pressure treatment on splanchnic hemodynamics and liver and kidney function in a porcine fecal peritonitis model

### SL Liu^1^, BS Schnüriger^2^, DC Casoni^3^, AK Kohler^2^, SJ Jakob^1^

#### ^1^Inselspital, Department of Intensive Care Medicine, Bern, Switzerland; ^2^Inselspital, Department of Visceral Surgery and Medicine, Bern, Switzerland; ^3^Department for Biomedical Research, Experimental Surgery Facility, Department for Biomedical Research, Bern, Switzerland

**Introduction:**

The effects of abdominal negative pressure treatment (NPT) on splanchnic hemodynamics after abdominal surgery for sepsis are not well known.

**Methods:**

Twenty mechanically ventilated pigs underwent surgery for splanchnic hemodynamic monitoring under general anesthesia, followed by analgo-sedation and randomization to fecal peritonitis or controls with/without NPT (ABThera, KCI, US) (n=5/group). After 8 hours of untreated peritonitis, animals were resuscitated w/wo NPT for 76 hours (resuscitation period, RP). Splanchnic hemodynamics and laboratory parameters were measured at baseline (BL, start of RP), and 24h, 48h and 72h after start of RP. Two/three-way RM-ANOVA or mixed-effects analysis, and Student *t* tests were performed.

**Results:**

NPT in controls had no effect. After sepsis induction, mean arterial pressure (MAP) decreased by 15 (7-22) mmHg, cardiac output (CO) by 1.3 (0.7-2.2) L/min, and arterial lactate increased by 0.2 (0.1-0.5) mmol/L. Sepsis and resuscitation was associated with increasing hepatic and renal arterial flows (p≤0.002, both), and increasing prothrombin time [max. increase 1.4 (0.3-2.9) sec], aspartate aminotransferase [max. increase 70 (49-79) IU] and creatinine [max. increase 72 (59-85) μmol/L; all p≤0.005]. MAP, CO, or hepatic/renal hemodynamics and functions were not affected by NPT. Nevertheless, NPT in sepsis resulted in numerically less noradrenaline administration (0.3±0.3 ug/min/kg in sepsis with NPT vs. 0.8±0.7 ug/min/kg without NPT, p=0.310) and positive fluid balance (2.8±0.4 ml/h/kg with NPT vs. 3.1±0.4 ml/h/kg without, p=0.241).

**Conclusions:**

In our experimental fecal peritonitis model, NPT did neither impair splanchnic hemodynamics nor abdominal organ function. Whether NPT helps to reduce noradrenaline and volume administration in abdominal sepsis should be evaluated in further studies.

## P505 Association between a C-reactive protein gene polymorphism (rs2794521) with the risk of develop septic shock in postsurgical patients of major abdominal surgery

### P Martínez-paz^1^, A Fadrique-Fuentes^2^, M Aragón-Camino^3^, M Heredia-Rodríguez^4^, E Gómez-Sánchez^5^, M Lorenzo-López^5^, E Gómez-Pesquera^5^, H Gonzalo-Benito^6^, E García-Morán^7^, E Tamayo^1^

#### ^1^Faculty of Medicine, University of Valladolid, Department of Surgery, Valladolid, Spain; ^2^Hospital of Medina del Campo, Anesthesiology and Reanimation Sevice, Medina del Campo, Spain; ^3^Clinic University Hospital of Valladolid, Anesthesiology and Reanimation Service, Valladolid, Spain; ^4^University Hospital of Salamanca, Anesthesiology and Reanimation Service, Salamanca, Spain; ^5^Clinic University Hospital of Valladolid, Anesthesiology and Reanimation Sevice, Valladolid, Spain; ^6^Instituto de Estudios en Ciencias de la Salud de Castilla y León, Instituto de Estudios en Ciencias de la Salud de Castilla y León, Valladolid, Spain; ^7^Clinic University Hospital of Valladolid, Cardiology Service, Valladolid, Spain

**Introduction:**

Shock is the most common cause of death in the postsurgical ICU, including septic shock and hypovolemic shock, reaching the 50–60% mortality in septic shock. The inadequate response of the immune system to the infection triggers a potent inflammatory cascade, where the C-Reactive Protein (CRP) is an essential key in the amplification and maintenance of this cascade. The gene encoding to CRP is located on the proximal long arm of human chromosome 1 (1q32). The GT polymorphism in the promoter sequence of *CRP* gene (rs2794521) has been associated with invasive pneumococcal disease. Thus, we analyze the relationship between rs2794521 polymorphism and the risk of developing septic shock in postsurgical patients.

**Methods:**

An observational, retrospective and single-center study was conducted on a sample of Caucasian patients undergoing major abdominal surgery, of which one part developed septic shock and another part developed systemic inflammatory response syndrome, who were used as control. The rs2794521 polymorphism was analyzed by the Sequenom´s MassARRAY platform and a recessive inheritance model was selected (CC vs TT/CT).

**Results:**

The possible association between the CC recessive form of the rs279451 polymorphism and the septic shock risk was analyzed, demonstrating a statistically significant relationship (p=0.02) between both conditions. Among patients who developed septic shock, 79.2% presented a recessive inheritance pattern while 54.5% showed the CT/TT genotype. On the other hand, those patients with the recessive form of the rs279451 polymorphism were selected and a statistical analysis was performed comparing those patients who developed septic shock from those who did not develop it, obtaining a statistically significant relationship (p=0.036) between the presence of the recessive form of polymorphism and the likelihood of developing septic shock.

**Conclusions:**

The recessive form of rs279451 polymorphism is a risk factor for septic shock in post-operative patients of major abdominal surgery.

## P506 A novel protein-binding RNA aptamer, Apta-1 - a new therapeutic tool to combat sepsis

### L Jedlina^1^, O Olin^1^, A Bylock^1^, V Casslén^1^, M Eriksson^2^, M Brewinska-Olchowik^3^, K Piwocka^3^, M Lindstam^1^

#### ^1^Aptahem AB, Malmo, Sweden; ^2^Section of Anesthesiology and Intensive Care, Department of Surgical Sciences, Uppsala University, Uppsala, Sweden; ^3^Laboratory of Cytometry, Nencki Institute of Experimental biology PAS, Warsaw, Poland

**Introduction:**

Sepsis remains one of the major causes of morbidity with mortality rates as high as 50 % worldwide, representing significant clinical challenge to confront highly intangible therapeutic needs. RNA-based structures are emerging as versatile tools encompassing a variety of functions capable to bypass the current protein- and cell-based therapies. RNA aptamers act as disease-associated protein antagonists. Here, the effects of an aptamer, Apta-1, were evaluated in animal models that mimic systemic inflammation in humans.

**Methods:**

High dose of LPS endotoxin was used to induce systemic inflammation in mice and in non-human primate animal models. Apta-1 was administered intravenously in two doses post LPS infection. Animals were monitored and blood samples collected up to 72 hours after Apta-1 administration. Healthy- and LPS-only treated animals served as control groups. Complex analyses of clinical parameters, hematology, serum biochemistry, inflammation and tissue damage markers were performed.

**Results:**

Apta-1 increased survival of endotoxin challenged animals up to 80% in a dose-dependent manner and exerted profound effects on well-being and recovery of healthy eating habits.  Administration of Apta-1 led to delayed coagulation and enhanced fibrinolysis; maintained the complement cascade activated while preventing it from further amplification. Expression of pro-inflammatory cytokines was reduced while anti-inflammatory increased. Endogenous pro-inflammatory molecules (DAMPs), secreted from injured cells, were preserved at healthy level in animals treated with Apta-1.

**Conclusions:**

Systemic inflammation and sepsis lead to severe dysregulation of several arms/axis of innate immune response. Our studies showed that Apta-1 affects various components of this system and restores the organism’s control over its dysregulated immune response. Thus, Apta-1 might be a promising potential therapeutic candidate to treat life-threatening conditions such sepsis.

## P507 Organ failures & mortality in surgical septic shock

### S Kongsayreepong^1^, K Jongsiriyunyong^2^

#### ^1^Siriraj Hospital, Anesthesiology & Critical Care, Bangkok, Thailand; ^2^Siriraj Hospital, Bangkok, Thailand

**Introduction:**

Surgical septic shock is associated with high morbidity & mortality especially when associated with organ dysfunction. The aim of this study was to study the incidence & predictors of organ failure & mortality in surgical septic shock on admission to the SICU after surgical source control

**Methods:**

This prospective observational study was done in 220 (age≥18 yrs) septic shock pts on admission to the SICU after surgical source control between Oct 2014-Sep 2019. Pts with preadmission ARDS, ESRD, stroke&delirium were excluded. Study data included: pts demographic, comorbidities, ASA class, source of infection, time of first antibiotic, shock reversal & source control, used of NE or other vasopressor/inotrope,steroid,intraop resuscitation & complications. First 72 hr data included admission& average Hb, serum albumin& lactate; fluid/blood replacement & balanced; organ failure as stroke, delirium,ARDS & AKI (KEDIGO criteria), 7days& 28days mortality.

**Results:**

Among the study pts (age 67.9±14.5 yrs), 82% was ASA III, admission SOFA score 2.9 ±2.1, 82% were cancer. 66% had intraabdominal infection, 38%, 6%, 3%,14 & 22%had AKI, ARDS,delirium,7 & 28 days mortality subsequently. Eight patients develped cardiac arrest during anesthesia induction. From multivariate analysis showed age >75 yrs, smoker, GFR<40 ml/min, inadequate preop/intraop fluid resuscitation & overused NE/inotrop, admitted Hb<9gm% & serum albumin<3mg/dL were significant predictor of AKI. Pts with 7 days mortality had higher incidence of low EF (<40%)/heart failure, longer (>6hr) time to start & inadequate source control, inadequate pre/intraop resuscitation & overused NE/inotrop, higher SOFA score, antibiotic resistance, later start RRT. Blood purification were used in 4 pts with good outcome.

**Conclusions:**

Pre/intraoperative resuscitation, faster & adequate source control were key component in surgical septic shock

## P508 Methane inhalation therapy in experimental sepsis

### A Rutai, SP Tallósy, B Zsikai, M Poles, D Érces, M Boros, J Kaszaki

#### University of Szeged, Institute of Surgical Research, Szeged, Hungary

**Introduction:**

Several preclinical studies demonstrated beneficial effects for methane (CH_4_) administration in various inflammatory conditions. Our aim was to investigate the consequences of post-treatment with inhaled CH_4_ in a clinically relevant intra-abdominal sepsis model.

**Methods:**

Anesthetized minipigs were subjected to fecal peritonitis (0.6 g/kg, 5-9x10^6^ CFU i.p.; n=22) or sham-operation (sterile saline i.p; n=5). Invasive hemodynamic monitoring with blood gas analyses was started between 16-24 hours, organ dysfunction parameters (PaO_2_/FiO_2_ ratio; mean arterial pressure; lactate, bilirubin, creatinine; urine output and platelet counts) were determined according to a modified porcine-specific Sequential Organ Failure Assessment (ps-SOFA) score system, the perfusion rate (PR) of sublingual microcirculation was measured by incident dark field illumination imaging. The animals were divided into non-treated septic or septic shock groups (n=6-6) and CH_4_-treated septic or septic shock (n=5-5) subgroups, CH_4_ inhalation started from the 18th hr (2.2% CH_4_ in normoxic air; 500 mL/min).

**Results:**

Despite the standardized induction, heterogeneous severity of organ damage was evolved. In septic and septic shock groups the median values of ps-SOFA score reached 5 (4.75-5.65) and 13 (11.75-14), respectively. Septic shock was characterized by significant elevations of creatinine and bilirubin levels, while the platelet count decreased (from 332 to 76 *10^9^/L). Inhalation of CH_4_ increased the sublingual PR by 22% in the septic group, the creatinine and bilirubin levels were decreased by 28% and 80%, respectively. CH_4_ post-treatment significantly decreased the ps-SOFA score (to 1; 0.5-2.75) and resulted in lower values in septic shock group (to 10; 9.5-12.4).

**Conclusions:**

Methane post-treatment effectively influences sepsis-related end organ dysfunction. Up to a severity threshold it may be a promising additional organ protective tool.

**Fundings:** NKFI K120232, NKFIH K116689; GINOP 2.3.2-15-2016-00015, EFOP-3.6.2-16-2017-00006

## P509 Association between vasopressor-dependent sepsis and limb or digit threat

### K Reitz^1^, X Li^2^, J Kennedy^3^, H Gershengorn^4^, M Neal^5^, H Wunsch^6^, H Prescott^7^, CC Chang^3^, D Angus^3^, C Seymour^3^

#### ^1^UPMC, General Surgery and Clinical Research, Investigations and Systems Modeling of Acute Illness (CRISMA), Pittsburgh, United States; ^2^Eli Lilly and Company, Eli Lilly, Indianapolis, United States; ^3^UPMC, Clinical Research, Investigation, and Systems Modeling of Acute Illness (CRISMA) Center, Pittsburgh, United States; ^4^University of Miami Miller School of Medicine, University of Miami Miller School of Medicine, Miami, United States; ^5^UPMC, General Surgery, Pittsburgh, United States; ^6^Sunnybrook Health Science Centre, Department of Critical Care Medicine, Toronto, Canada; ^7^University of Michigan, Division of Pulmonary and Critical Care Medicine, University of Michigan, Ann Arbor, United States

**Introduction:**

Vasoactive medications are commonly used in sepsis treatment but may correlate with peripheral ischemia and the well-publicized complication of limb and digit loss. Yet, the association between limb and digit threat and the intensity, duration, and pattern of vasopressor exposure are unknown.

**Methods:**

We studied adults (2010-2014) at 12 hospitals in an integrated health system who met criteria for Sepsis-3. We identified the time to clinically apparent limb or digit threat using clinical adjudication among those with vasopressor-dependent sepsis (i.e. >1 hour of vasopressors at sepsis onset) who had a surgical evaluation within 28-days of sepsis onset. We defined daily vasopressor intensity as 0 to 4 vasopressors administered. Then, we created a time-dependent model for threat with mortality as a competing risk with a weight function to estimates the varying contribution of vasopressors over time. We determined the sub-distribution hazard (SH) ratio of threat for various patterns of vasopressor exposure and intensity, adjusted for age, baseline risk factors, and sequential organ failure assessment (SOFA) score at sepsis onset.

**Results:**

Of 110,621 adults with sepsis, 13,147 (12%) were vasopressor-dependent (age, 66 [IQR, 56-77]; 7,040 [54%] males; max SOFA score, 8 [SD 5]). Of these, 3,664 (28%) died and 117 (0.9%) had evaluations for limb or digit threat 4 [IQR, 1-8] days after sepsis onset. The model-based weight function showed the contribution of vasopressors to threat was stable over time (Fig 1A). Overall, a 1 unit increase in cumulative vasopressor exposure was associated with risk of threat (SH ratio, 2.60 [95%CI, 1.60-4.23], p<.001). For various patterns of vasopressor exposure, greater intensity associated with increased risk of threat (Fig 1B). Compared to constant exposure, an increasing and peak pattern associated with the greatest SH (Fig 1C).

**Conclusions:**

Cumulative vasopressor exposure was associated with an increased risk-adjusted hazard of limb or digit threat following sepsis.

**Fig. 1 (abstract P509).**
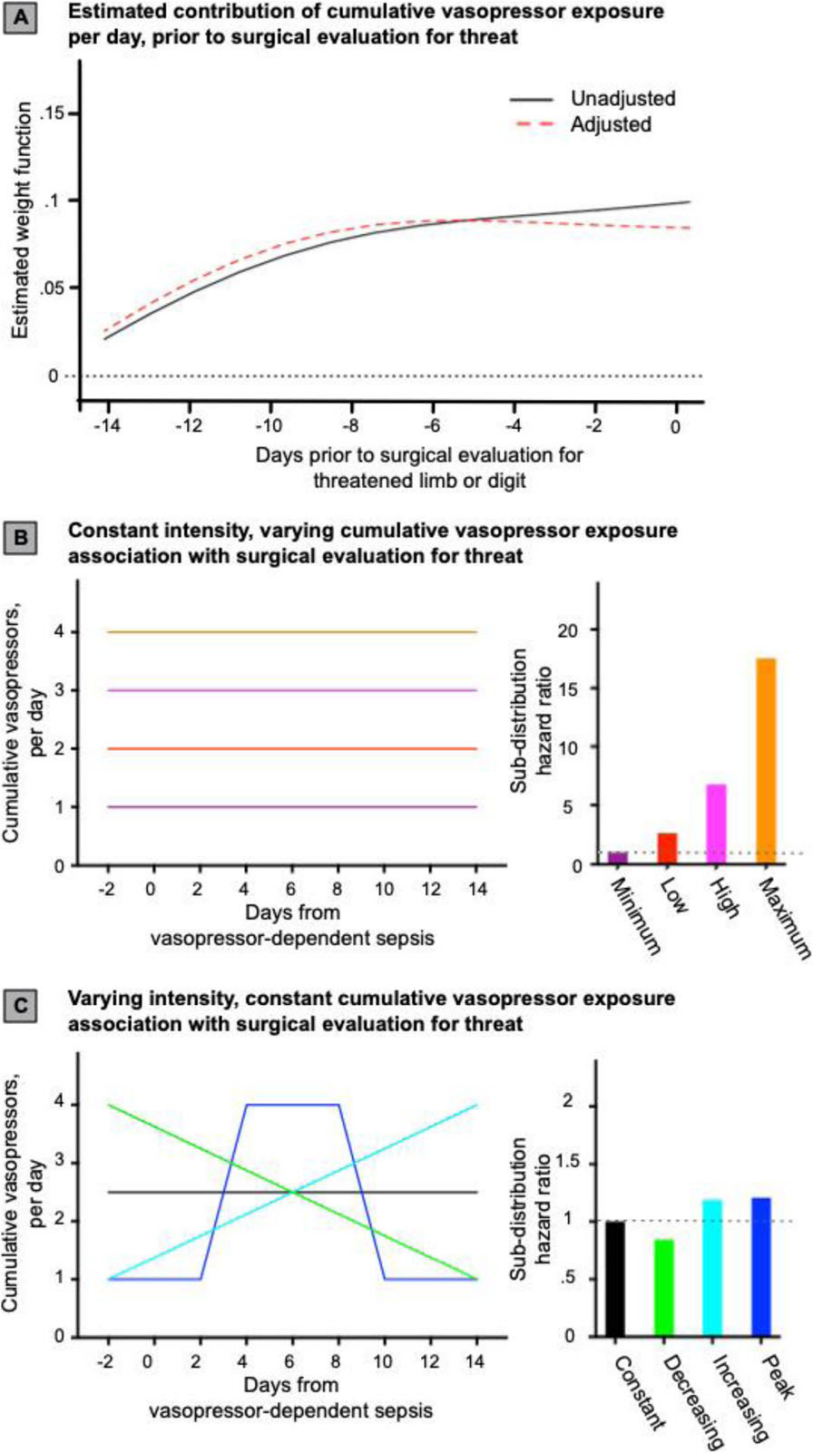
Relationship between vasopressor exposure and limb or digit threat following vasopressor-dependent sepsis. Panel A demonstrates the estimated contribution of daily vasopressor intensity prior to surgical evaluation for limb or digit threat, with mortality as a competing risk. Panel B and C explore the relationship between threat and both cumulative vasopressor exposure and the pattern of exposure following sepsis onset. (B) The maximum cumulative vasopressor exposure was associated with the highest risk of limb or digit threat (SHR 17.5) when compared to reference exposure pattern (SHR 1.0, reference). (C) Increasing (SHR 1.2) and peak (SHR 1.2) patterns of cumulative exposure were associate with an increased SH of limb threat, while a decreasing pattern was associated with a lower risk (SHR 0.8) when compared to constant intensity (SHR 1.0, reference). Abbreviations: SHR: Sub-distribution hazard ratio

## P510 Evaluation of sepsis awareness among various groups in Turkey: a survey study

### S Erel, O Ermis, Ö Nadastepe, L Karabıyık

#### Gazi University School of Medicine, Anesthesiology and Intensive Care, Ankara, Turkey

**Introduction:**

Sepsis is a common life-threatening condition in critically ill patients [1]. Public awareness is important for early recognition of sepsis and improvement of outcomes [2]. We aimed to evaluate sepsis awareness among different groups of people.

**Methods:**

Prospective paper-based surveys were issued between 1st July and 1st August 2019 to patients, the relatives of the patiens, hospital staff and general public who gave consent to participate in the study. The questionnaire included ten questions about demographic informations, occupational informations of hospital stuff and sepsis awareness.

**Results:**

A total of 588 participated in the survey. Of these participants, 87 (14.3%) were patients, 50 (8.5%) were relatives of patients, 134 (22.8%) were physicians, 125 (21.3%) were medical students, 49 (8.3%) were nurses, 51 (8.7%) were other hospital stuff and 92 (%15.6) were other people. Of these participants, 425 (72.3%) had heard of the word “sepsis”. 206 (35.0%) responded correctly regarding the definition of sepsis. 325 (55.3%) of the participants heard the word “sepsis” during their education, but only 53 (9%) heard it through the media. In the groups of high school graduates, university graduates and postgraduates, the rate of hearing the word sepsis and correctly identifying sepsis is significantly higher than the primary school graduates or illiterate groups. (p<0.05). Physicians, nurses and medical students were heard of the word “sepsis” significantly more than other groups (p<0.005). Physicians and medical students responded more accurately to the definition of sepsis than other groups (p<0.05).

**Conclusions:**

Public awareness of sepsis is limited compared to healthcare workers. Increasing public knowledge of sepsis through education and through media may contribute to raising public awareness and improving outcomes.

**References:**

1. Singer M et al. JAMA 315:801-810, 2016.

2. Rubulotta FM et al. Crit Care Med 37:167-170, 2009

## P511 Analysis of acetaminophen metabolizing enzymes gene polymorphisms in critically ill febrile patients with sepsis and septic shock

### C D´Angelo, C Scorcella, R Domizi, E Casarotta, P Giaccaglia, E Damiani, J Montomoli, S Zuccari, S Vannicola, A Donati

#### Università Politecnica delle Marche, Biomedical Science and Public Health, Ancona, Italy

**Introduction:**

UDP-glucuronidation and oxido-reduction are the main pathways involved in the hepatic metabolism of acetaminophen. Polymorphisms rs8330 C>G for Uridine diphosphate-glucuronosyltransferase 1A1 (UGT1A1p) and rs776746 G>A for Cytochrome 3A5 (CYP3A5p) oxidoreductase [1] might potentially affect the efficacy of paracetamol in controlling fever and increase the toxic effects.

**Methods:**

Prospective observational study in 50 febrile (body temperature ≥ 38 °C) patients with sepsis/septic shock. Blood samples were collected before i.v. administration of 1 g standard dose of paracetamol (t0), at 30 minutes (t1) and 2 hours (t2) after the infusion, to measure drug plasma levels. An analysis of polymorphisms was conducted.

**Results:**

The prevalence of the polymorphisms was 36% for UGT1A1p and 8% for CYP3A5p. Peak plasmatic levels in patients with CYP3A5p were higher than wild type(WT) and UGT1A1p at t1(17.6[16.2-21.7] vs 11.4[9.1-14.3] and 10.5[9.3-13.1]mcg/ml; p=0.03 and 0.02 respectively). No significant differences were founded on t2 (Fig.1A). Notably, the three groups received a comparable pro kg dose of acetaminophen. No difference was found between groups in term of toxic effects. Patients carrying the CYP3A5p showed a more pronounced effect on body temperature in respect of WT and UGT1A1p with a delta t° of -0.85[-1.65—0.02] vs -0.4[-0.8—0.05] and -0.45[0.65—0.1]°C respectively, but it does not reach statistical significance (Fig.1B). Only 50% of the patients reach a temperature <38°C at t2 and only 20% <37.5°C.

**Conclusions:**

Polymorphisms in enzymes involved in the metabolism of acetaminophen are relatively common. CYP3A5p seems to lead to higher peak plasmatic concentration and a slightly increased efficacy in fever control. These data could serve as basis for further research on pharmacokinetics of acetaminophen in critically ill patients with well-known increased volume of distribution that could affect clinical efficacy.

**References:**

[1]  Mazaleuskaya et al. Pharmacogenet Genomics 25:416-26, 2015.

**Fig. 1 (abstract P511).**
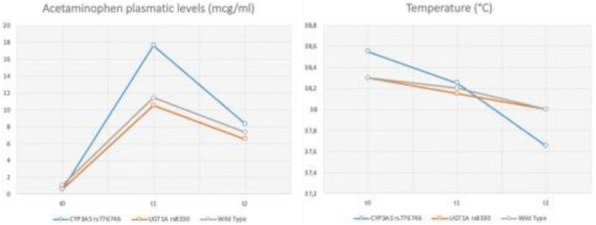
Panel A: variations of acetaminophen plasmatic levels after 30 minutes (t1) and 2 hours (t2) after administration of an iv dose of 1g of paracetamol in WT patients and patients carrying mutation; Panel B: body temperature variations in WT patients and patients carrying mutations

## P512 The association between clinical phenotype cohesiveness and sepsis transitions after presentation

### JN Kennedy^1^, EB Brant^1^, KM DeMerle^2^, CH Chang^3^, S Wang^4^, DC Angus^1^, CW Seymour^5^

#### ^1^University of Pittsburgh, Clinical Research, Investigation, and Systems Modeling of Acute Illness (CRISMA) Center, Department of Critical Care Medicine, Pittsburgh, United States; ^2^University of Pittsburgh, Division of Pulmonary, Allergy and Critical Care Medicine, Pittsburgh, United States; ^3^University of Pittsburgh, Clinical Research, Investigation, and Systems Modeling of Acute Illness (CRISMA) Center, Department of Biostatistics, Pittsburgh, United States; ^4^University of Florida, Division of Quantitative Sciences, Department of Biostatistics, Gainesville, United States; ^5^University of Pittsburgh, Clinical Research, Investigation, and Systems Modeling of Acute Illness (CRISMA) Center, Department of Critical Care Medicine, Department of Emergency Medicine, Pittsburgh, United States

**Introduction:**

Sepsis is common, deadly, and heterogeneous. Prior work proposed clinical phenotypes of sepsis at presentation. However, little is known about how those phenotypes change over time. We explored trajectories of clinical sepsis phenotypes for 24hrs after presentation and contribution of phenotype cohesiveness to subsequent transitions.

**Methods:**

We analyzed a retrospective cohort of adult patients meeting Sepsis-3 criteria within 6hrs of presentation using electronic health data from 12 hospitals in a large health system in Pennsylvania. We predicted clinical phenotypes at 6-hr intervals over 24hrs using Euclidean distance anchored to previously published cluster centroids (α,ß,*y*,∂). We determined phenotype cohesiveness using probability of assignment at presentation, defining core members as ≥90% and marginal as <90% probability. We determined how members transitioned to other phenotypes over 24hrs using t-distributed stochastic neighbor embedding (tSNE) plots and determined the odds (95%CI) of transition.

**Results:**

We studied 37,198 adult sepsis encounters (median age 69, [IQR 57-81]; 51% male, median SOFA at arrival 3, [IQR 2-4]) hospitalized for at least 24hrs. At presentation, encounters were 28% α-type, 30% ß-type, 28% *y*-type, and 14% ∂-type. Phenotype was unchanged over 24hrs among 68% of encounters. Transitions were most common for ∂-type (20% core, 57% marginal) and least common for α-type (11% core, 39% marginal, Figure 1A). Core members were less likely to transition than marginal members for all phenotypes (p<0.01, Figure 1B,1C) The odds of ever transitioning from presenting phenotype increased significantly for marginal members vs. core for all phenotypes (α: OR 5.3, 95%CI 4.7-6.0; ß: OR 2.9, 95%CI 2.6-3.2; *y*: OR 3.6 95%CI 3.2-3.9, ∂: OR 5.2, 95%CI 4.6-5.9, p<0.01 for all).

**Conclusions:**

More than half of patients had stable clinical sepsis phenotypes for 24hrs after presentation. Transitions to other phenotypes were significantly more common among marginal members and those in the ∂-type.

**Fig. 1 (abstract P512).**
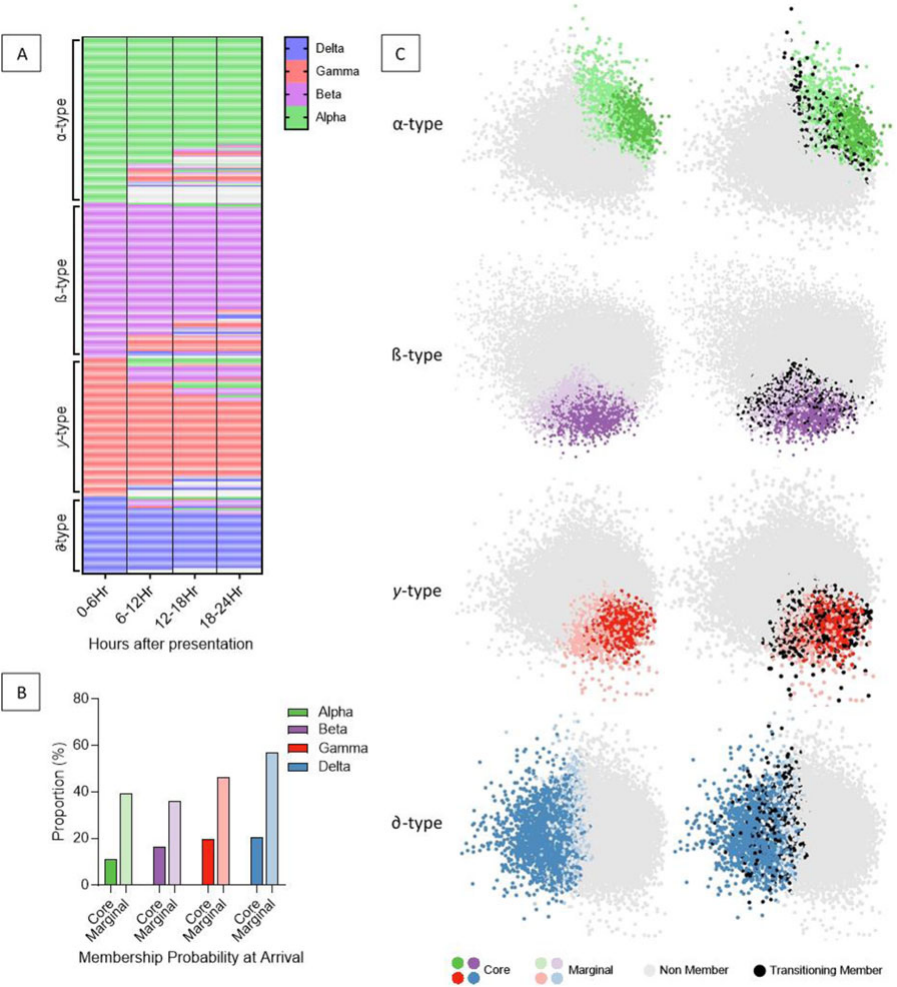
(A) Heap Map showing phenotype assignments at 6-hr intervals for first 24hrs after presentation. (B) Bar graph showing proportion of encounters transitioning from phenotype at presentation within 24hrs, by arrival phenotype assignment and probability of membership. (C) tSNE plots for α-type, ß-type, y-type, and ∂-type, with core (dark), marginal (light), and non-members (grey) in plots on the left and core, marginal, non members, and transitioning members (black) on the right

